# A heuristic derived from analysis of the ion channel structural proteome permits the rapid identification of hydrophobic gates

**DOI:** 10.1073/pnas.1902702116

**Published:** 2019-06-24

**Authors:** Shanlin Rao, Gianni Klesse, Phillip J. Stansfeld, Stephen J. Tucker, Mark S. P. Sansom

**Affiliations:** ^a^Department of Biochemistry, University of Oxford, Oxford OX1 3QU, United Kingdom;; ^b^Clarendon Laboratory, Department of Physics, University of Oxford, Oxford OX1 3PU, United Kingdom;; ^c^OXION Initiative in Ion Channels and Disease, University of Oxford, Oxford OX1 3PT, United Kingdom

**Keywords:** ion channel annotation, water, hydrophobic gating, molecular dynamics, machine learning

## Abstract

Ion channel proteins control ionic flux across biological membranes through conformational changes in their transmembrane pores. An exponentially increasing number of channel structures captured in different conformational states are now being determined; however, these newly resolved structures are commonly classified as either open or closed based solely on the physical dimensions of their pore, and it is now known that more accurate annotation of their conductive state requires additional assessment of the effect of pore hydrophobicity. A narrow hydrophobic gate region may disfavor liquid-phase water, leading to local dewetting, which will form an energetic barrier to water and ion permeation without steric occlusion of the pore. Here we quantify the combined influence of radius and hydrophobicity on pore dewetting by applying molecular dynamics simulations and machine learning to nearly 200 ion channel structures. This allows us to propose a simple simulation-free heuristic model that rapidly and accurately predicts the presence of hydrophobic gates. This not only enables the functional annotation of new channel structures as soon as they are determined, but also may facilitate the design of novel nanopores controlled by hydrophobic gates.

## Significance

Ion channels are nanoscale protein pores in cell membranes. An exponentially increasing number of structures for channels means that computational methods for predicting their functional state are needed. Hydrophobic gates in ion channels result in local dewetting of pores, which functionally closes them to water and ion permeation. We use simulations of water behavior within nearly 200 different ion channel structures to explore how the radius and hydrophobicity of pores determine their hydration vs. dewetting behavior. Machine learning-assisted analysis of these simulations allowed us to propose a simple model for this relationship and present an easy method for rapidly predicting the functional state of new channel structures as they emerge.

Ion channel proteins are water-filled, ion-conducting pores that are key components of biological membranes (1). In a resting or inactivated state, the flow of ions through the pore may be interrupted at 1 or more positions along the channel, known as “gates.” On activation, for example in response to ligand binding or a change in the membrane potential, conformational changes generally lead to expansion of the channel pore at its gate region(s), thereby switching it from a closed (i.e., nonconducting) to an open (conductive) conformation. Ion channels represent attractive therapeutic targets, and there is considerable interest in elucidating the mechanisms underlying this process in many different ion channel families. In most cases, the determination of channel structures in various different conformational states provides the key molecular basis for understanding how this “gating” is regulated, often supported by insights from electrophysiological and other biophysical approaches.

For a newly determined ion channel structure, its conductive state is most frequently inferred by measuring the physical dimensions of its transmembrane pore, displayed as a profile of pore radius along the central axis along with an image of the pore-lining surface. Of the several available methods for doing this, one of the most widely used is the HOLE program (2). Such pore radius profiles provide an indication of the maximum size of ions that might be accommodated within a transmembrane pore, with steric constrictions narrower than the radius of a water molecule (∼0.15 nm) considered potential gates.

**Fig. 1.**
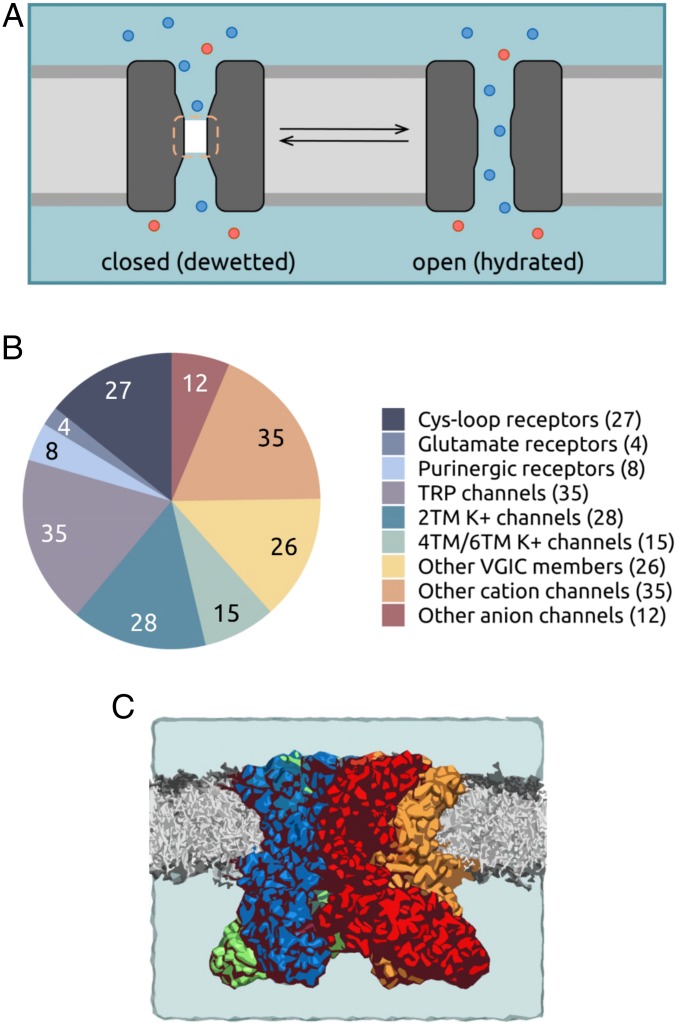
(*A*) Schematic of a hydrophobic gate in an ion channel. A hydrophobic gate region (dashed line) can spontaneously dewet to form a dry (i.e., vapor) state and functionally close the channel. Widening of the gate leads to wetting (i.e., hydration) of the region and a functionally open state. Water is represented by the pale blue background shading; ions, by red and blue spheres. (*B*) Pie chart summarizing the ∼200 channel dataset that forms the basis of the present study. The channels are grouped into 9 broad families: cys-loop receptors, ionotropic glutamate receptors, purinergic receptors, TRP channels, K^+^ channels (2TM, 4TM, and 6TM), other members of the voltage-gated ion channel (VGIC) superfamily, other cation channels, and other anion channels. (*C*) Representation of a typical simulation system used in this study. Shown is the TRPV4 channel in a phospholipid (gray) bilayer. Water molecules and ions are present but omitted for clarity.

The permeation of ions and water through a given region in a subnanometer pore is influenced not only by its radius, but also by the local hydrophobicity of the pore lining. Ion permeation may readily occur through polar regions only slightly larger than the radius of the permeating species, but a hydrophobic pore segment of comparable dimensions may undergo spontaneous dewetting to form a local nanoscale region devoid of water and ions (3–5). Therefore, a particular channel conformation may present an energetic barrier to permeation (i.e., be gated closed) in a hydrophobic region of the pore without requiring full steric occlusion. This is referred to as a hydrophobic gate (3, 6–8), or sometimes as a vapor lock (9). In such cases, the widening and consequent wetting (i.e., hydration) of the hydrophobic gate region enables the passage of ions through the channel (Fig. 1*A*).

The concept of hydrophobic gating in members of the Cys-loop family of ligand-gated ion channels, including the nicotinic acetylcholine receptor (10), GLIC (7, 11), and the 5-HT_3_ receptor (12–14) is now well established. However, experimental and computational evidence of hydrophobic gates and barriers within other ion channels has also emerged, including those for the TWIK-1 K2P channel (15), BK channels (16), the CorA magnesium channel (17), the CRAC channel Orai (18), and members of the transient receptor potential (TRP) channel family (19–22). Hydrophobic gating has also been demonstrated in synthetic nanopores (23, 24).

Computationally, molecular dynamics (MD) simulations of channel structures embedded within a lipid bilayer have aided the functional interpretation of new structures. Such simulations may range in complexity from characterization of free energy landscapes of ion permeation and/or the conformational transitions associated with gating (6, 11) to simpler simulations of water behavior within channel pores (12).

The influence of pore radius and hydrophobicity on the wetting/dewetting (i.e., nanoscale liquid-vapor transition of water inside) of channels has previously been examined by simulation of model nanopores (3, 4, 25). For a uniformly hydrophobic constriction, the dewetted (vapor) state appears stable below a critical pore radius of ∼0.5 nm, but this threshold radius decreases if the hydrophobicity of the pore lining is reduced (26). While channel structures containing hydrophobic regions with radii of ∼0.3 nm have been reported to represent dewetted nonconductive conformations (e.g., ref. 16), our ability to predict this based on structure alone is hampered by a lack of detailed understanding of how pore dewetting depends on the local radius and hydrophobicity, especially in complex biological structures such as ion channels, where a wide range of hydrophobicity profiles are possible via different combinations of pore-lining side chains.

Fortunately, such analysis is now possible due to the recent explosion in structural data available for ion channels. This has arisen mostly from advances in structural biology, especially cryo-electron microscopy (27) and there are now 900 structures of >100 unique ion channel proteins deposited in the Protein Data Bank. Furthermore, improved software (e.g., CHAP; www.channotation.org) is now available for the analysis of channel pore dimensions, hydrophobicity profiles, and simulations of pore wetting/dewetting (28). Thus, the ion channel structural proteome can now be subjected to a systematic examination of hydrophobic gating.

Here we used these improved approaches to quantify the influence of local pore radius and hydrophobicity on channel dewetting via MD simulations of water behavior in nearly 200 ion channel structures (Fig. 1*B*). The results of this systematic analysis provide a structure-based simulation-free heuristic model that allows rapid prediction of the conductive state of new channel structures as soon as they are determined. This method can also facilitate the design of novel nanopores (29) that contain hydrophobic gates (30, 31).

## Results

### Protocol for Channel Simulation and Analysis.

**Fig. 2.**
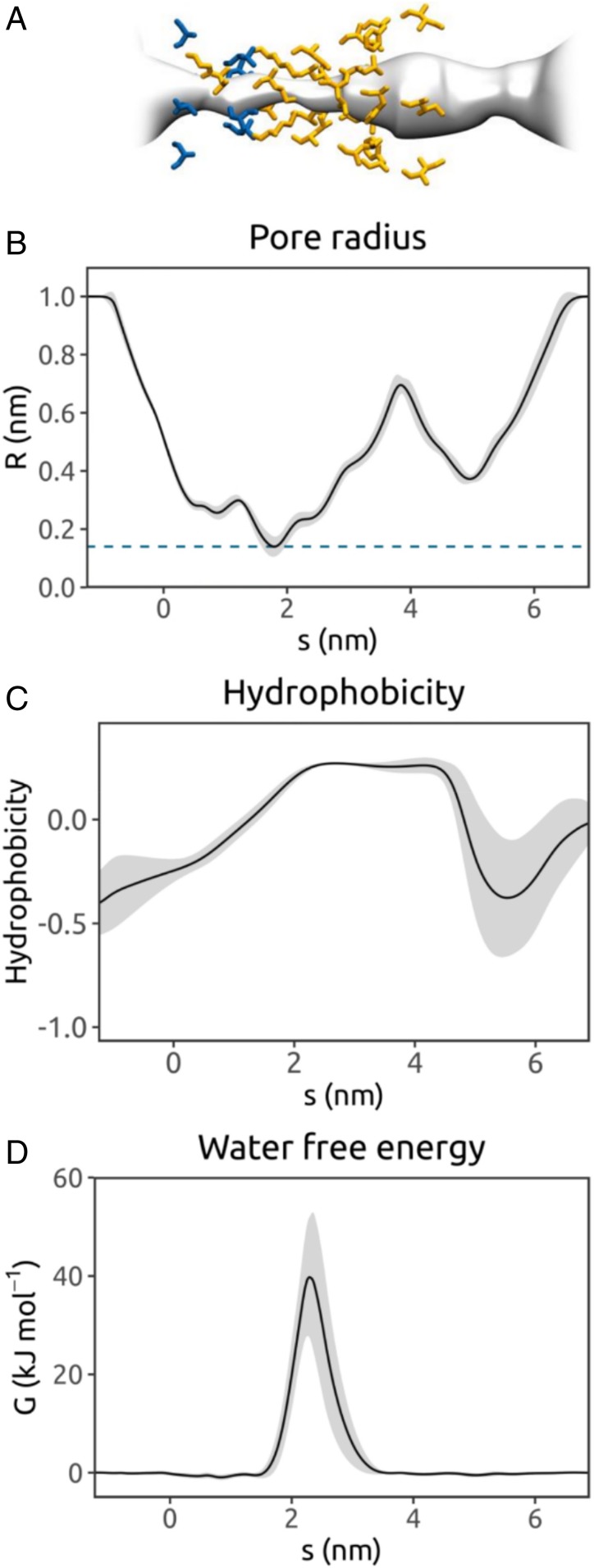
Annotation of an ion channel structure via MD simulations of water within the pore, (*A*) illustrated for the TRPV4 ion channel (PDB ID code 6BBJ) (47). (*B*) Pore radius profile derived from 1 of 3 × 30-ns simulations of the protein embedded in a phospholipid bilayer. The mean radius, calculated using the final 20 ns of the trajectory with a sampling interval of 0.5 ns, is shown as a black line (with the gray band representing the SD over time) as a function of position, *s*, along the pore axis. The pore-lining surface and the pore-lining residues of the channel (hydrophobic in orange; polar in blue) are shown at the top. (*C*) Hydrophobicity profile for the pore-lining side chains as a function of position along the pore axis *s*. The hydrophobicity values are evaluated using a normalized version of the Wimley–White scale (48). (*D*) Free energy for water as a function of position along the pore axis *s*. The free energy profile is obtained from the density of water within the pore as estimated from the MD simulation (details in *Methods*).

Each selected ion channel structure was embedded within a phospholipid bilayer, solvated on either side with 0.15 M NaCl (Fig. 1*C*), and subjected to 3 replicate 30-ns atomistic MD simulations to determine the behavior of water within the transbilayer pore. Using our recently described channel annotation software (CHAP) (28), side chains that line the pore during simulations were identified and time-averaged profiles were calculated for the pore radius, local hydrophobicity, and free energy of water as a function of position along the length of the pore (Fig. 2 and *SI Appendix*, Fig. S1). Therefore, each point along the permeation pathway of a channel structure was associated with corresponding values for 3 variables of interest: pore radius, pore hydrophobicity, and free energy of water at that region of the pore. This enables the dependency of water free energy (and hence pore wetting/dewetting) on local radius and hydrophobicity to be established and averaged across all the channel structures analyzed.

During the simulations of water behavior, positional restraints were applied to backbone atoms of the protein. This was done to preserve the experimentally determined conformational state of the protein while allowing for local side chain flexibility. This is an important consideration, as we are aiming to analyze water behavior within known conformational states of ion channels, not to explore possible conformational changes of those channels by simulation, which would require much longer simulations. Nevertheless, we have previously shown that the presence/absence of such restraints does not significantly alter the free energy profile for water within a given simulation (32). Furthermore, we have also run more extended simulations to establish that 3 × 30 ns provides a robust estimate of the free energy landscape for water within an ion channel, even in the presence of a hydrophobic gate (*SI Appendix*, Fig. S2). Indeed, tracking individual water molecules within a hydrophobic gate region that we hydrate at the start of the simulation reveals that dewetting occurs within the first few nanoseconds (*SI Appendix*, Fig. S3), as we have previously seen for model nanopores with hydrophobic gates (30, 33). Thus, for any individual channel, our simulation protocol enables us to establish whether or not a hydrophobic gate is present and to determine the free energy profile for water within any such gate. Furthermore, the relative simplicity of the process allows us to perform simulation analysis across a dataset of nearly 200 channel structures that are representative of the ion channel structural proteome (*SI Appendix*, Table S1).

### Ion Channel Dataset.

To fully sample the range of radius-hydrophobicity combinations present within the 900 ion channel structures in the Protein Data Bank (PDB), a reduced dataset was manually curated on the basis of structure quality (primarily resolution) and structural redundancy. Structures with a resolution of 5 Å or worse were excluded (*SI Appendix*, Fig. S4), as were those with an incomplete backbone trace in their pore-lining segments. When multiple structures of the same ion channel species and of similar pore conformation (judged on root-mean-square-deviations and pore radius profiles along with visual inspection) were available, only the higher-resolution structures of the wild-type protein were chosen. This yielded a reduced dataset of ∼200 structures (*SI Appendix*, Table S1). The MD trajectory dataset (3 × 30 ns for each structure) formed the basis for the subsequent analysis of pore dewetting behavior.

### Analysis of the Main Energetic Barrier in Each Channel Structure.

**Fig. 3.**
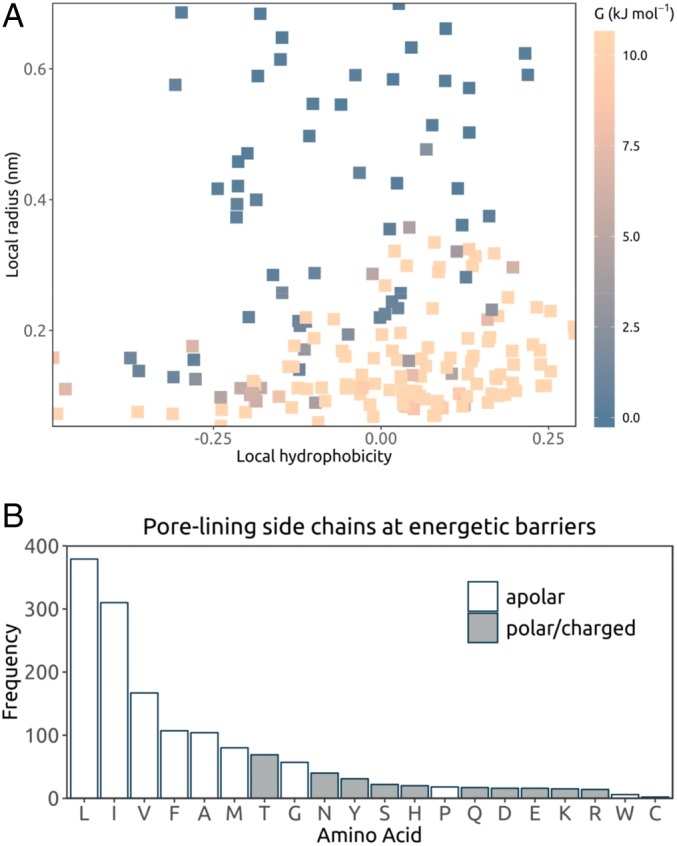
(*A*) Analysis of the main energetic barrier to water permeation in each ion channel structure. Each point corresponds to a single channel structure (details in *SI Appendix*, Table S1), indicating the hydrophobicity and pore radius at the highest barrier in the water free energy profile (averaged across the 3 repeat simulations). The height of the barrier is given by the color scale. Points with energy values outside the range are shown in the same color as the lower or upper boundary. For structures containing a narrow pore-loop selectivity filter (e.g., K^+^ channels), the section on their water free energy profile corresponding to the filter region is excluded when locating the maximum energy point to represent the structure. (*B*) Frequency of the different pore-lining side chains at an energetic barrier in these ion channel structures, that is, where the water free energy is >1.5 RT (3.9 kJ mol^−1^) at the mean position of the residue along the pore axis *s*. Multiple amino acids may occur at each energetic barrier.

Water free energy profiles derived from equilibrium simulations of wetting/dewetting within a channel structure can serve as the basis for functional annotation (12). Any dewetted region presenting an energetic barrier to water is likely to form a barrier to permeation (26), and thus water permeability may serve as a proxy for ion permeability. Based on our simulation dataset, channel structures containing 1 or more functionally closed gates were identified. These corresponded to ∼70% of the ∼200 structures analyzed (*SI Appendix*, Table S1). Fig. 3*A* shows a point for each barrier to water permeability in the (*hydrophobicity*, *radius*) plane, colored by the height of the water free energy barrier (*G*). Significant barriers (*G* >2.6 kJ mol^−1^) were frequently associated with hydrophobic regions. The distribution of water free energy values on the pore hydrophobicity-radius landscape were also in qualitative agreement with predictions from simplified models of hydrophobic gating (4, 26), and in regions with elevated water free energies, hydrophobic aliphatic side chains (leucine, isoleucine, and valine) were most prevalent (Fig. 3*B*).

### Analysis of Residues Lining Ion Channel Pores.

**Fig. 4.**
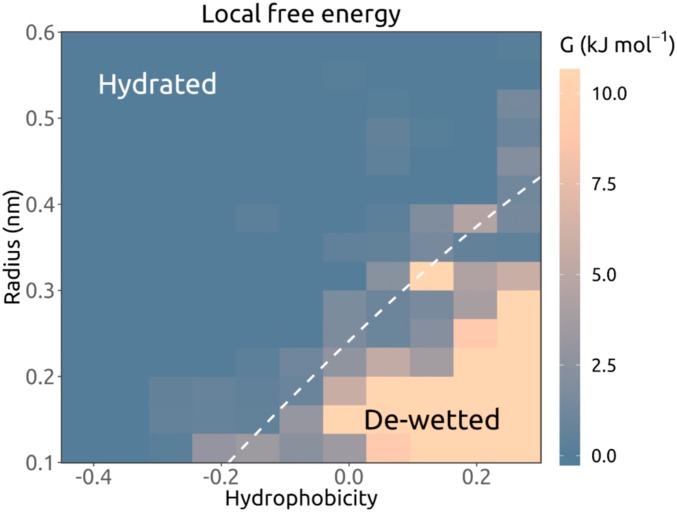
Analysis of all pore-lining residues for a training set of simulated ion channel structures in terms of the local water free energy. From each simulation, mean measurements are noted at the mean position (*s*) along the pore axis (Fig. 2) of any side chain oriented toward the pore for at least 50% of the simulation. The local water free energy is shown as a function of hydrophobicity and pore radius for all occurrences of pore-lining side chains in the simulated structures. Hydrophobicity values are based on the Wimley–White scale, linearly normalized to [−1, 0.34], in relative units. The hydrophobicity-radius grid is colored by mean energy. Regions with energy values outside the color scale range are shown in the same color as the lower or upper bound. The white dashed contour line indicates the 1 RT (2.6 kJ mol^−1^) position as given by a polynomial support vector machine classifier.

The relationship between local pore dimensions, hydrophobicity, and free energy of water at any given position along the channel axis also became clearer when all pore-lining residues in the ∼200 structures were surveyed, rather than just those present at barriers in the water free energy profiles. Examination of the distribution of water density values associated with all pore-lining residues revealed a substantive tail in the distribution corresponding to dewetted regions, that is, those with reduced water density relative to that of bulk water (*SI Appendix*, Fig. S5). When a water free energy landscape in the local pore hydrophobicity-radius plane was constructed, a clear pattern emerged, with the landscape clearly divided into 2 regions, corresponding to wetted and dewetted pores (Fig. 4). In the hydrophobic region of the landscape, an energetic barrier can be encountered in channel regions with radii up to ∼0.4 nm (the radius of a water molecule is ∼0.15 nm; Fig. 4). Conversely, hydrophilic regions of the landscape include pores that become hydrated at much smaller radii (i.e., <0.2 nm). We note that the water free energy landscape is very similar even when alternative hydrophobicity scales are used (*SI Appendix*, Fig. S6). The water free energy landscape was also evaluated using subsets for the dataset from which structures with poorer resolution than 4.5 Å (6 structures) and 4.0 Å (a further 19 structures) were excluded. These exclusions of lower-resolution structures did not significantly change the resultant water free energy landscapes (*SI Appendix*, Fig. S7).

By training a support vector machine (SVM) classifier with a polynomial kernel (34), we found that the hydrophobicity-radius landscape could be divided into 2 regions, corresponding to the likelihood of pore wetting vs. dewetting. These 2 regions are indicated by the dotted line in Fig. 4.

### Heuristic for Predicting the Conductive State.

**Fig. 5.**
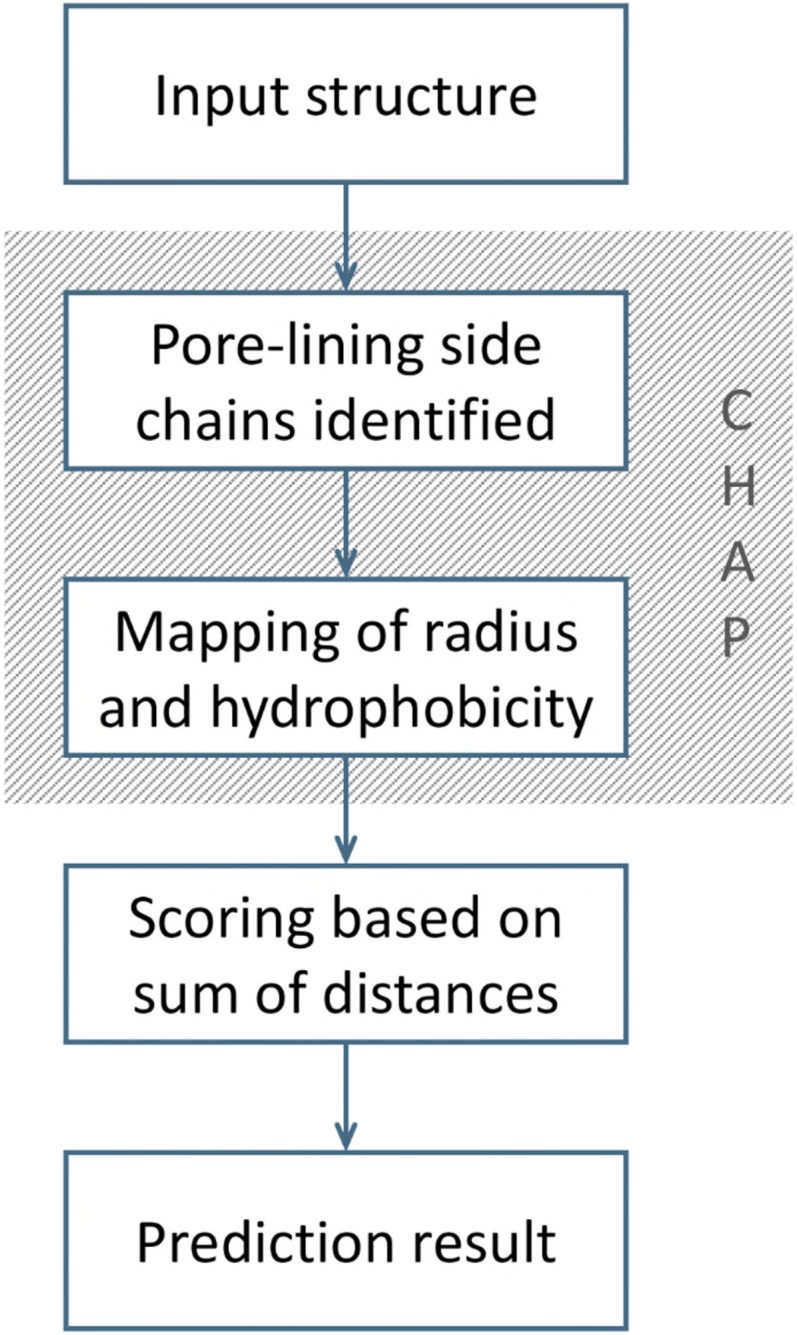
Schematic of a heuristic prediction approach for the permeation state of an ion channel structure based on analysis of hydrophobicity and pore radius profiles, both of which can be derived from a static structure using the CHAP analysis tool (28).

The water free energy landscape derived from the dataset of channel structures and simulations allows us to devise a simple simulation-free heuristic technique for predicting the functional state of ion channel structures, based on the correlation with pore dimensions and hydrophobicity alone (Fig. 5). Having used CHAP to identify pore-lining residues and to estimate pore radius and hydrophobicity profiles, the (*hydrophobicity, radius*) values for the pore-lining residues of a given channel are then mapped onto the landscape described above. By identifying the number of residues for which the corresponding (*hydrophobicity, radius*) points fall below the SVM classification line (i.e., in the dewetted region of the landscape), the likelihood of a channel structure corresponding to a dewetted and thus functionally closed state can be predicted. The sum of shortest distances (Σ*d*) for residue points falling below the SVM open vs. closed classification line (corresponding to the dotted line in Fig. 4) provides a score indicating whether the channel structure is likely to contain a closed gate. As this machine learning-based predictive approach can be performed using automated analysis of a single set of coordinates (28) and does not require MD simulation of the structure, it can also be performed within a few seconds on a standard desktop; for example, the total CPU time was <15 min for CHAP and heuristic prediction for the dataset of ∼200 channel structures (Dataset S1).

**Fig. 6.**
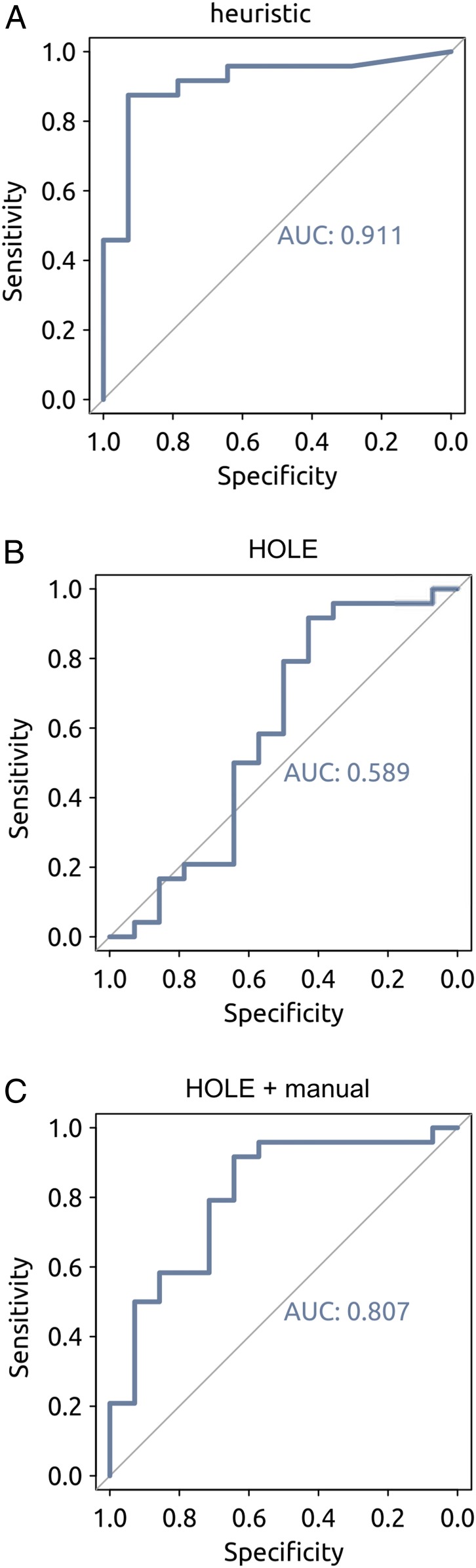
ROC curve analyses based on comparison of simulations of pore wettability (*SI Appendix*, Table S1) and heuristic-based predictions. The heuristic predictions are evaluated against the simulation results for pore wettability and the relationship between sensitivity (i.e., true positive rate) and specificity (i.e., true negative rate) are defined and displayed in the standard fashion. (*A*) ROC curve for the heuristic model presented in the current study, indicating an optimal cutoff of Σ*d* = 0.55 for the heuristic prediction of a closed channel. (*B* and *C*) ROC curve for predictions based on HOLE (*B*) and ROC curve for predictions based on HOLE plus manual correction for channels with K^+^ channel-like selectivity filters (*C*). In each case, the AUC is given.

To evaluate the heuristic prediction, we performed receiver operating characteristic (ROC) curve analysis (35) for the polynomial kernel SVM and also for a linear kernel. For the polynomial kernel the area under the curve (AUC) was 0.91, compared with 0.83 for the linear kernel (*SI Appendix*, Fig. S8), demonstrating the advantage of using the polynormal kernel SVM. We also used ROC curve analysis to compare the current heuristic prediction model with simpler models based on the minimum pore radius as evaluated using HOLE or on the minimum pore radius evaluated using HOLE alongside manual exclusion of those pores with K^+^ channel selectivity filters. As shown in Fig. 6, the AUC values for the heuristic model, for HOLE, and for HOLE with manual exclusion of K^+^ channel filters were 0.91, 0.59, and 0.81 respectively. Therefore, we suggest that our heuristic model is a significant advancement from HOLE-based methods.

**Fig. 7.**
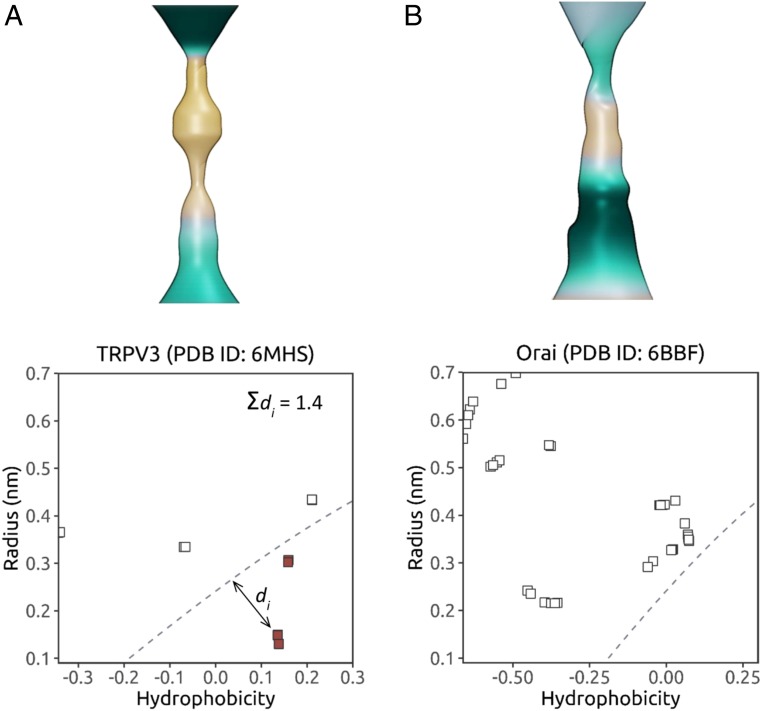
Illustration of the heuristic prediction approach using 2 recent structures of the TRPV3 channel in a nonconductive sensitized conformation (PDB ID code 6MHS) (*A*) and the CRAC channel Orai in an open conformation (PDB ID code 6BBF) (*B*). Pore radius and hydrophobicity surfaces (with the pale-brown color corresponding to maximum hydrophobicity) are shown for the transmembrane domains of both structures. In the 2 graphs, for each pore-lining side chain, the channel pore radius at the residue is plotted against the corresponding local hydrophobicity value. The sum of shortest distances between the dashed (1 RT) contour line and all points falling below it (colored red) is then used as a score for identifying closed gates. A structure is predicted to be in a nonconductive state if it has a value of Σ*d* > 0.55.

We illustrate the effectiveness of this heuristic approach using 2 recent structures (Fig. 7): a structure of the TRPV3 channel in a putative sensitized (but nonconductive) conformation (PDB ID code 6MHS) and a structure of the CRAC channel (Orai) in an open conformation (PDB ID code 6BBF) (36). For the open-state Orai channel, none of the (*hydrophobicity, radius*) points fall below the SVM classification line, and so the structure is predicted to be fully wetted and correspond to a functionally open state of the channel. In marked contrast, for the TRPV3 structure, 12 points were below the SVM line, and the sum of shortest distances for residue points falling below this line was Σ*d* = 1.4, leading to clear prediction of a closed hydrophobic gate for this particular channel conformation. Consistent with these predictions, when these 2 structures were subjected to the complete MD simulation and analysis protocols described above, the resulting water free energy profiles (*SI Appendix*, Fig. S9) confirmed the predictions made by this heuristic model.

As a further test of the ability of the simulation protocol to provide an estimate of the free energy landscape for water within an ion channel, we ran more extended (0.5 µs) simulations of the water in 3 channels (Slo1, PDB ID code 5TJI; GLIC, PDB ID code 3EHZ; and MscS, PDB ID code 2OAU; *SI Appendix*, Fig. S10), 2 of which lay close to the dividing line from the SVM classifier. In each case, the extended (0.5 µs) simulation did not change the free energy profile for water within the channel from the profile calculated from the 3 × 30 ns simulations of the same channel.

## Discussion

**Fig. 8.**
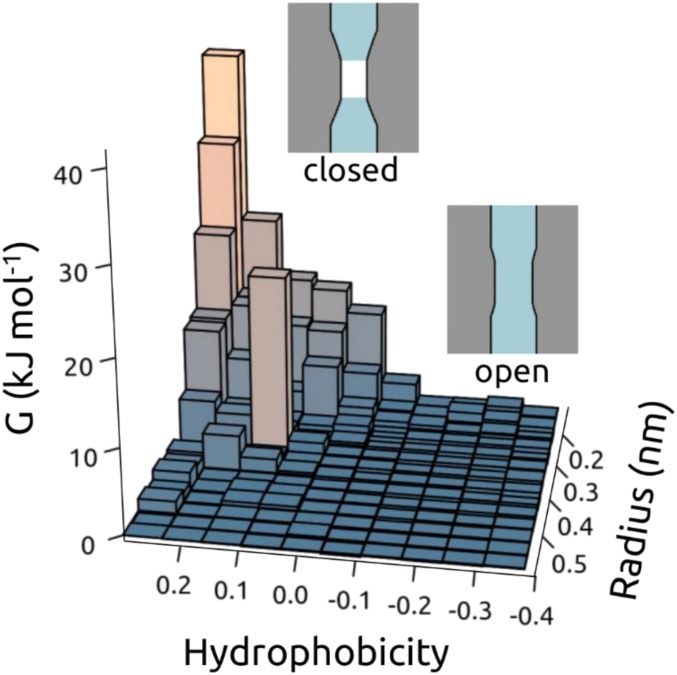
Schematic of hydrophobic gating as a function of (*hydrophobicity, radius*) of the transmembrane pore. The surface shows the free energy of water within a channel as a function of (*hydrophobicity, radius*) corresponding to the data in Fig. 5. Schematic depictions of dewetted (closed) and hydrated (open) channels are shown for the 2 main regions of the data.

We have used MD simulations of pore water density to determine the wetting/dewetting properties of nearly 200 ion channel structures that are representative of the currently available ion channel structural proteome. A systematic analysis of the behavior of pore dewetting within these structures as a function of local radius and hydrophobicity revealed an almost linear dependence of the critical radius for wetting on the local hydrophobicity. Therefore, because pore radius and hydrophobicity profiles can now be readily estimated from any known structure, this analysis enables us to propose a simple simulation-free heuristic model for identifying closed gates in newly determined structures of ion channels or other forms of transmembrane pores (Fig. 8). This model is based on an underlying energy landscape derived from ∼600 simulations of water behavior in ion channels, with a combined duration of ∼18 µs.

It should be noted that pore wetting of a hydrophobic gate is necessary but not always sufficient for ion conduction. However, our method provides a rapid and robust exploratory approach to the functional annotation of novel channel structures. Importantly, this analysis can be performed in a matter of seconds before more detailed simulations and/or experimental studies of the relationship between ion channel structure and function. Furthermore, this method may also facilitate the design and engineering of novel nanopores (29) that contain switchable hydrophobic gates.

## Methods

The WHAT IF server (https://swift.cmbi.umcn.nl/whatif/) (37) was used to model incomplete side chains in the selected channel structures. Channel structures were inserted in a POPC (1-palmitoyl-2-oleoyl-*sn*-glycero-3-phosphocholine) bilayer using a multiscale protocol (38) for generating and equilibrating protein-membrane simulation systems. MD simulations were performed with GROMACS (http://www.gromacs.org/) version 5.1 (39), using the OPLS all-atom protein force field with united-atom lipids (40) and the TIP4P water model (41). The integration time step was 2 fs. A Verlet cutoff scheme was applied, and the particle mesh Ewald method (42) was used to calculate long-range electrostatic interactions. Temperature and pressure were maintained at 37 °C and 1 bar, respectively, using the velocity-rescale thermostat (43) in combination with a semi-isotropic Parrinello and Rahman barostat (44), with coupling constants of τ_T_ = 0.1 ps and τ_P_ = 1 ps. Bonds were constrained using the LINCS algorithm (45). A harmonic restraint with a force constant of 1,000 kJ mol^−1^ nm^−2^ was placed on the protein backbone atoms during simulations. CHAP (www.channotation.org) (28) was used to analyze trajectory frames, with bandwidths of 0.14 nm and 0.45 nm applied for estimating water density and hydrophobicity, respectively, along each channel axis.

SVM classification was conducted using the Caret package (46) in R version 3.4.4 (www.r-project.org). To calculate the ROC curves, the dataset of ∼200 channel structures was randomly partitioned into training:validation:testing sets at a ratio of 64%:16%:20%. The SVM model was trained using repeated 10-fold cross-validation. The resultant SVM classification lines were used to predict simulation outcomes (i.e., whether or not a channel structure is hydrated or contains energetic barrier to water) for the testing set of ∼40 structures via calculation of sum of shortest distances (Σ*d*; Fig. 7) scores. Further documentation of the heuristic method can be found on the CHAP website: https://www.channotation.org/docs/heuristic_method/.

## Supplementary Material

Supplementary File

Supplementary File
