# Supplementary material for: A heuristic derived from analysis of the ion channel structural proteome permits the rapid identification of hydrophobic gates
Source: Proc Natl Acad Sci U S A. 2019 Jun 24;116(28):13989–95. doi: 10.1073/pnas.1902702116 (PMC6628796; doi:10.1073/pnas.1902702116)
Supplement: Supplementary File [file pnas.1902702116.sapp.pdf]

Table S1. Ion channel structures analysed. Any auxiliary subunits, Fab fragments, ligands, ions, and/or water molecules present in a PDB entry were removed prior to molecular dynamics simulations. Large structures that were truncated to include only the transmembrane or pore domain for analysis are indicated by an asterisk (*) next to their PDB ID. Where available, the likely functional state of a channel structure as suggested upon its publication is noted. The height of the main energetic barrier to water (G_{water}) is recorded for each structure, with values coloured red (> 1 RT) or blue (≤ 1 RT).

Protein	PDB	Method	Resolution (Å)	Proposed functional state	G_{water} (kJ mol^{-1})
**Cys-loop receptors**					
5HT3R, *M. musculus*	4PIR	X-ray	3.5		42
5HT3R, *M. musculus*	6BE1	cryo-EM	4.31	closed	44
ELIC, *E. chrysanthemi*	2VL0	X-ray	3.3	closed	90
ELIC, *E. chrysanthemi*	3RQW	X-ray	2.91	closed	79
GABAAR, *H. sapiens*	4COF	X-ray	2.97	desensitised	1
GABAAR, *H. sapiens*	6A96	cryo-EM	3.51	open	0
GABAAR, *H. sapiens*	6D6T	cryo-EM	3.86	desensitised	2
GABAAR, *H. sapiens*	6D6U	cryo-EM	3.92	desensitised	28
GLIC, *G. violaceus*	2XQ7	X-ray	3.4	open	1
GLIC, *G. violaceus*	3EHZ	X-ray	3.1	open	0
GLIC, *G. violaceus*	4F8H	X-ray	2.99		0
GLIC, *G. violaceus*	4HFI	X-ray	2.4	open	0
GLIC, *G. violaceus*	4NPP	X-ray	3.35	open	1
GLIC, *G. violaceus*	4QH5	X-ray	3	open	0
GLIC, *G. violaceus*	5J0Z	X-ray	3.25	desensitised	1
GLIC, *G. violaceus*	5L47	X-ray	3.3	closed	48
GluCl, *C. elegans*	3RHW	X-ray	3.26	open	1
GluCl, *C. elegans*	3RIF	X-ray	3.35	open	1
GluCl, *C. elegans*	4TNV	X-ray	3.6	closed	12
GlyR, *D. rerio*	3JAD	cryo-EM	3.9	closed	4.6
GlyR, *D. rerio*	3JAE	cryo-EM	3.9	open	0
GlyR, *D. rerio*	3JAF	cryo-EM	3.8	desensitised/ intermediate	1
GlyR, *H. sapiens*	5CFB	X-ray	3.04	closed	23
GlyR, *H. sapiens*	5VDH	X-ray	2.85	desensitised/ intermediate	24
nAChR, *T. marmorata*	1OED	EM	4	closed	10
nAChR, *T. marmorata*	2BG9	EM	4	closed	3.6
nAChR, *H. sapiens*	5KXI	X-ray	3.94	desensitised	0
**Ionotropic glutamate receptors**					
GluA, *R. norvegicus*	5WEK*	cryo-EM	4.6	closed	4
GluA, *R. norvegicus*	5WEL*	cryo-EM	4.4	closed	7
GluA, *R. norvegicus*	5WEO*	cryo-EM	4.2	open	1
GluN, *X. laevis*	5UOW*	cryo-EM	4.5	open	1
**Purinergic receptors**					
P2X3, *H. sapiens*	5SVJ	X-ray	2.98	closed	26
P2X3, *H. sapiens*	5SVK	X-ray	2.77	open	0
P2X3, *H. sapiens*	5SVL	X-ray	2.9	desensitised	23
P2X4, *D. rerio*	3H9V	X-ray	3.1	closed	39
P2X4, *D. rerio*	3I5D	X-ray	3.46	closed	43

www.pnas.org/cgi/doi/10.1073/pnas.1902702116

P2X4, *D. rerio*	4DW0	X-ray	2.9	closed	36
P2X4, *D. rerio*	4DW1	X-ray	2.8	open	0
P2X7, *G. gallus*	5XW6	X-ray	3.1	closed	56
**Transient receptor potential (TRP) channels**					
NOMPC, *D. melanogaster*	5VKQ*	cryo-EM	3.55	closed	11
PKD2, *H. sapiens*	5K47	cryo-EM	4.22	closed	10
PKD2, *H. sapiens*	5MKE	cryo-EM	4.3	open	1
PKD2, *H. sapiens*	5MKF	cryo-EM	4.2	closed	13
PKD2, *H. sapiens*	5T4D	cryo-EM	3	closed	32
PKD2, *H. sapiens*	6D1W	cryo-EM	3.54	open	15
PKD2L1, *M. musculus*	5Z1W	cryo-EM	3.38	open	29
TRPA1, *H. sapiens*	3J9P	cryo-EM	4.24	closed	76
TRPM2, *N. vectensis*	6CO7	cryo-EM	3.07	closed	44
TRMP4, *M. musculus*	6BCL	cryo-EM	3.54	closed	9
TRMP4, *M. musculus*	6BCQ	cryo-EM	3.25	closed	10
TRMP4, *H. sapiens*	6BQR	cryo-EM	3.2	closed	8
TRPML1, *H. sapiens*	5WJ5	cryo-EM	3.72	closed	27
TRPML1, *H. sapiens*	5WJ9	cryo-EM	3.49	open	1
TRPML1, *M. musculus*	5WPV	cryo-EM	3.59	closed	9
TRPML3, *C. jacchus*	5W3S	cryo-EM	2.94	closed	7
TRPML3, *H. sapiens*	6AYE	cryo-EM	4.06	closed	16
TRPML3, *H. sapiens*	6AYF	cryo-EM	3.62	open	0
TRPML3, *H. sapiens*	6AYG	cryo-EM	4.65	closed	10
TRPV1, *R. norvegicus*	3J5P	cryo-EM	3.28	closed	9
TRPV1, *R. norvegicus*	3J5Q	cryo-EM	3.8	open	4.9
TRPV1, *R. norvegicus*	3J5R	cryo-EM	4.2	closed	5.2
TRPV1, *R. norvegicus*	5IRX	cryo-EM	2.95	open	1
TRPV1, *R. norvegicus*	5IRZ	cryo-EM	3.28	closed	8
TRPV1, *R. norvegicus*	5IS0	cryo-EM	3.43	closed	9
TRPV2, *O. cuniculus*	5AN8	cryo-EM	3.8	desensitised	67
TRPV2, *O. cuniculus*	6BWJ	X-ray	3.1	closed	73
TRPV2, *R. norvegicus*	5HI9	cryo-EM	4.4	open	38
TRPV4, *X. tropicalis*	6BBJ	cryo-EM	3.8	closed	40
TRPV5, *O. cuniculus*	6B5V	cryo-EM	4.8	closed	21
TRPV6, *R. norvegicus*	5IWK	X-ray	3.25	closed	32
TRPV6, *R. norvegicus*	5IWP	X-ray	3.65	closed	21
TRPV6, *R. norvegicus*	5WO6	X-ray	3.31		20
TRPV6, *H. sapiens*	6BO8	cryo-EM	3.6	open	1
TRPV6, *H. sapiens*	6BOB	cryo-EM	3.9	closed	37
**2TM family K^+ channels**					
GsuK, *G. sulfurreducens*	4GX5	X-ray	3.7	closed	12
K2P1.1, *H. sapiens*	3UKM	X-ray	3.4	open	0
K2P2.1, *M. musculus*	5VKP	X-ray	2.8		0
K2P4.1, *H. sapiens*	4WFE	X-ray	2.5	open	0
K2P10.1, *H. sapiens*	4BW5	X-ray	3.2	open	1
K2P10.1, *H. sapiens*	4XDL	X-ray	3.5	open	1
KcsA, *S. lividans*	1K4C	X-ray	2		34
KcsA, *S. lividans*	2ITD	X-ray	2.7		44
KcsA, *S. lividans*	3EFF	X-ray	3.8	closed	49

KcsA, *S. lividans*	3FB5	X-ray	2.8	partially open	0
KcsA, *S. lividans*	3PJS	X-ray	3.8	open	0
KcsA, *S. lividans*	4UUJ	X-ray	2.4	closed	24
Kir2.2, *G. gallus*	3JYC	X-ray	3.11	closed	20
Kir2.2, *G. gallus*	3SPC	X-ray	2.45	closed	28
Kir2.2, *G. gallus*	3SPI	X-ray	3.31	closed	37
Kir3.2, *M. musculus*	3SYA	X-ray	2.98	closed	3.1
Kir3.2, *M. musculus*	3SYO	X-ray	3.54	closed	0
Kir6.2, *R. norvegicus*	6BAA	cryo-EM	3.63	closed	29
Kir6.2, *H. sapiens*	6C3O	cryo-EM	3.9		37
KirBac1.1, *B. pseudomallei*	2WLL	X-ray	3.65	closed	14
KirBac1.3/Kir3.1, *M. musculus/B. xenovornas*	2QKS	X-ray	2.2	closed	27
KirBac3.1, *M. magnetotacticum*	2WLH	X-ray	3.28	closed	45
KirBac3.1, *M. magnetotacticum*	2WLI	X-ray	3.09	closed	10
KirBac3.1, *M. magnetotacticum*	2WLJ	X-ray	2.6	closed	7
MthK, *M. thermautotrophicus*	1LNQ	X-ray	3.3	open	0
NaK, *B. cereus*	2AHY	X-ray	2.4	closed	20
NaK, *B. cereus*	3E86	X-ray	1.6	open	0
NaK/NavSulP, *B. mycoides/S. pontiacus*	3VOU	X-ray	3.2	closed	59
**4TM or 6TM K^+ channels**					
KCNQ1, *X. laevis*	5VMS	cryo-EM	3.7	closed	89
Kv1.2, *R. norvegicus*	2A79	X-ray	2.9	open	0
Kv1.2, *R. norvegicus*	2R9R	X-ray	2.4	open	0
Kv1.2, *R. norvegicus*	3LUT	X-ray	2.9	open	0
Kv10.1, *R. norvegicus*	5K7L*	cryo-EM	3.78	closed	17
Kv11.1, *H. sapiens*	5VA1*	cryo-EM	3.7	open	0
KvAp, *A. pernix*	1ORQ	X-ray	3.2	open	0
KvLm, *L. monocytogenes*	4H33	X-ray	3.1	closed	14
KvLm, *L. monocytogenes*	4H37	X-ray	3.35	closed	11
MloK1, *M. loti*	3BEH	X-ray	3.1	closed	22
MloK1, *M. loti*	6EO1	cryo-EM	4.5	open	0
Slo1, *A. californica*	5TJ6	cryo-EM	3.5	open	0
Slo1, *A. californica*	5TJI	cryo-EM	3.8		0
Slo2.2, *G. gallus*	5U70	cryo-EM	3.76	open	0
Slo2.2, *G. gallus*	5U76	cryo-EM	3.76	closed	0
**Other members of the voltage-gated ion channel (VGIC) superfamily**					
Cav1.1, *O. cuniculus*	5GJV	cryo-EM	3.6	closed	80
CavAb, *A. butzleri*	4MVM	X-ray	3.20		7
CavAb, *A. butzleri*	5KLB	X-ray	2.7		27
TAX5 (CNG), *C. elegans*	5H3O	cryo-EM	3.5	open	1
LliK (CNG),	5V4S	cryo-EM	4.2	closed	33

L. licerasiae					
HCN1, *H. sapiens*	5U6O	cryo-EM	3.5	closed	28
Hv1, *M. musculus*	3WKV	X-ray	3.45	closed	32
InsP3R1, *R. norvegicus*	3JAV*	cryo-EM	4.7	closed	47
Nav1.4, *E. electricus*	5XSY	cryo-EM	4	open	28
NavAb, *A. butzleri*	4EKW	X-ray	3.21	closed	19
NavAb, *A. butzleri*	4MW8	X-ray	3.26		2.5
NavAb, *A. butzleri*	5EK0	X-ray	3.53	closed	90
NavAb, *A. butzleri*	5VB2	X-ray	3.2	closed	119
NavAb, *A. butzleri*	5VB8	X-ray	2.85	open	6
NavAe1, *A. ehrlichii*	4LTO	X-ray	3.46	closed	85
NavMm, *M. marinus*	4CBC	X-ray	2.66	open	25
NavMm, *M. marinus*	4OXS	X-ray	2.8	open	28
NavPaS, *P. americana*	5X0M	cryo-EM	3.8	closed	41
NavRh, *R. HIMB114*	4DXW	X-ray	3.05	closed	58
RyR1, *O. cuniculus*	3J8H*	cryo-EM	3.8	closed	11
RyR1, *O. cuniculus*	5TB0*	cryo-EM	4.4	closed	8
RyR2, *S. scrofa*	5GO9*	cryo-EM	4.4	closed	12
RyR2, *S. scrofa*	5GOA*	cryo-EM	4.2	open	0
TPC1, *A. thaliana*	5DQQ	X-ray	2.87	closed	39
TPC1, *M. musculus*	6C9A	cryo-EM	3.2	open	2
TPC1, *M. musculus*	6C96	cryo-EM	3.4	closed	56
**Other cation channels**					
ASIC1, *G. gallus*	2QTS	X-ray	1.9	desensitised (-like)	23
ASIC1, *G. gallus*	3S3W	X-ray	2.6	desensitised (-like)	21
ASIC1, *G. gallus*	4NTW	X-ray	2.07	open	1
ASIC1, *G. gallus*	4NTY	X-ray	2.65	open	0
ASIC1, *G. gallus*	4NYK	X-ray	3	desensitised	17
ASIC1, *G. gallus*	5WKU	X-ray	2.95	closed	38
ExbB/ExbD, *E. coli*	5SV0	X-ray	2.6		0
ExbB/ExbD, *E. coli*	5SV1	X-ray	3.5		0
M2, *Influenza A*	3BKD	X-ray	2.05	closed	4.6
M2, *Influenza A*	4QKC	X-ray	1.1		6
M2, *Influenza A*	5JOO	X-ray	1.41		5.9
M2, *Influenza A*	5TTC	X-ray	1.4		5.9
M2, *Influenza A*	5UM1	X-ray	1.45		6
M2, *Influenza B*	2KIX	aq NMR	(NMR)	closed	5.4
MCU, *C. elegans*	5ID3	aq NMR	(NMR)	closed	87
MgtE, *T. thermophilus*	2ZY9	X-ray	2.94	closed	69
MgtE, *T. thermophilus*	4U9N	X-ray	2.2	closed	56
MscL, *M. tuberculosis*	2OAR	X-ray	3.5	closed	46
MscL, *S. aureus*	3HZQ	X-ray	3.82	closed/ intermediate	0
MscL, *M. acetivorans*	4Y7J	X-ray	4.1	closed/ intermediate	0
MscL, *M. acetivorans*	4Y7K	X-ray	3.5	closed	81
MscS, *E. coli*	2OAU	X-ray	3.7	open	15
MscS, *E. coli*	2VV5	X-ray	3.45	open	0
NNT, *T. thermophilus*	5UNI	X-ray	2.2		71

Orai, *D. melanogaster*	4HKR	X-ray	3.35	closed	8
Piezo1, *M. musculus*	6BPZ	cryo-EM	3.8	closed	37
TMEM175, *C. minutus*	5VRE	X-ray	3.30		45
TRIC, *C. elegans*	5EGI	X-ray	3.3		7
TRIC, *C. elegans*	5EIK	X-ray	2.3		12
TRIC, *S. acidocaldarius*	5WUC	X-ray	1.6	closed	71
TRIC, *S. acidocaldarius*	5WUE	X-ray	2.4	open	0
TRIC, *C. psychrerythraea*	5WUF	X-ray	2.40		2.4
YetJ, *B. subtilis*	4PGR	X-ray	1.95	closed	29
YetJ, *B. subtilis*	4PGS	X-ray	2.5	open	4.3
YetJ, *B. subtilis*	4PGU	X-ray	3.40	closed	58
**Other anion channels**					
BEST1, *G. gallus*	4RDQ	X-ray	2.85	open	76
KpBest, *K. pneumoniae*	4WD8	X-ray	2.3	closed	71
CFTR, *H. sapiens*	5UAK	cryo-EM	3.87	closed	15
CFTR, *D. rerio*	5UAR	cryo-EM	3.73	closed	58
CFTR, *D. rerio*	5W81	cryo-EM	3.37	closed	38
CLC-K, *B. taurus*	5TQQ	cryo-EM	3.76		43
TehA, *H. influenzae*	3M71	X-ray	1.2	closed	52
TehA, *H. influenzae*	4YCR	X-ray	2.3		29
TMEM16A, *M. musculus*	5OYB	cryo-EM	3.75	closed	9
TMEM16A, *M. musculus*	5OYG	cryo-EM	4.06	closed	32
TMEM16A, *M. musculus*	6BGI	cryo-EM	3.8	closed	26
TMEM16A, *M. musculus*	6BGJ	cryo-EM	3.8	closed	6

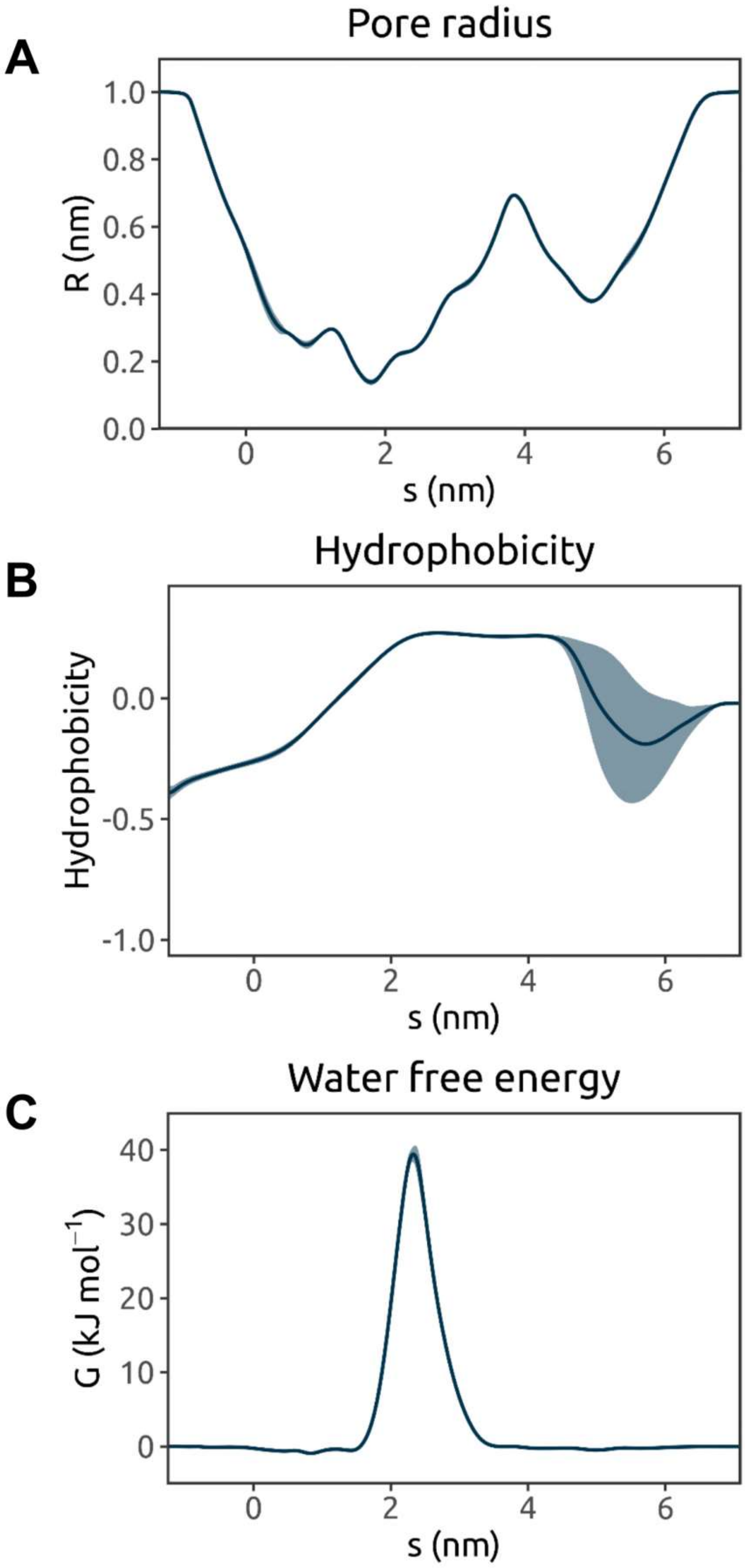

Figure S1. Average pore radius, hydrophobicity, and water free energy profiles derived from 3 x 30 ns MD simulations of water in the TRPV4 ion channel (PDB ID 6BBJ). The one-standard-deviation range is represented by the lighter-coloured band.

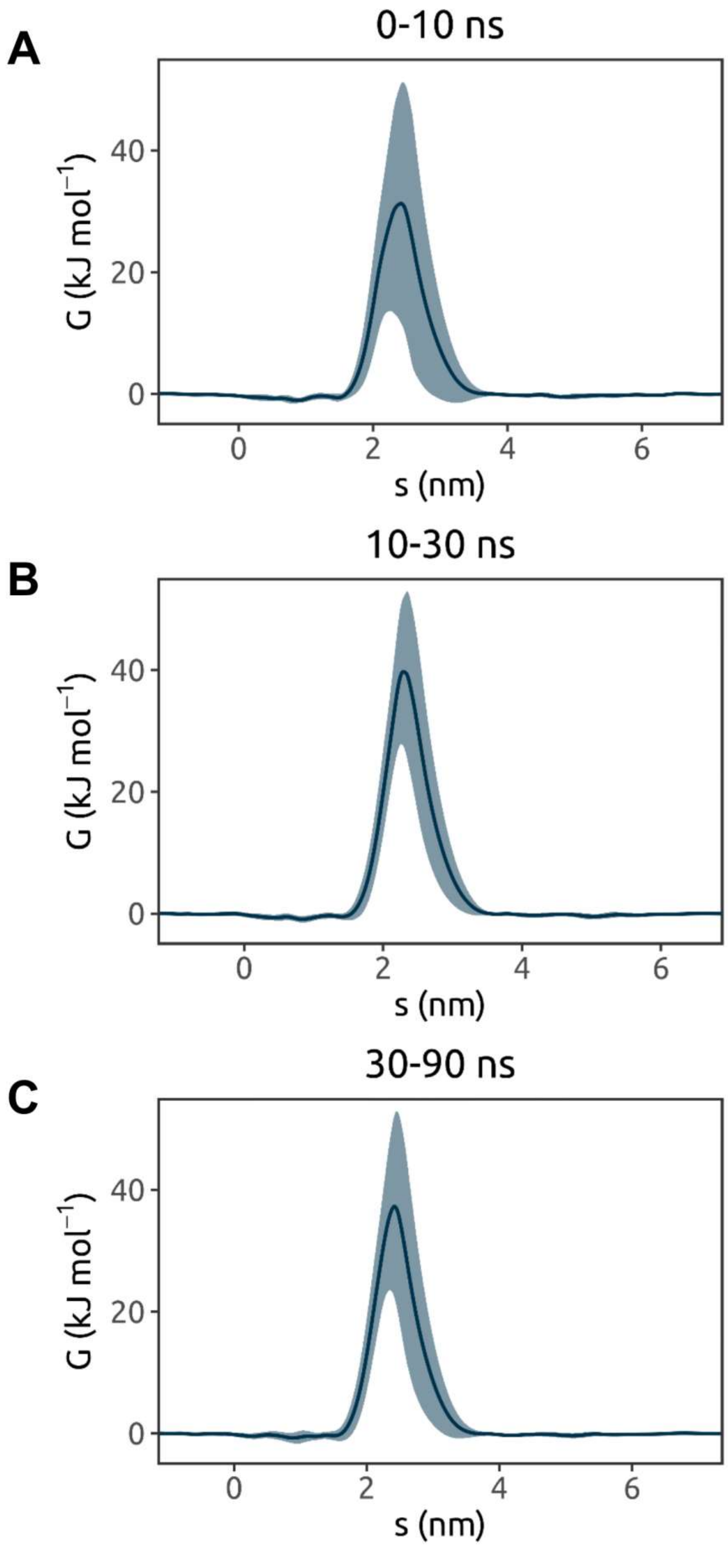

Figure S2. Water free energy profiles derived from different intervals of one 90 ns MD simulation of water in the TRPV4 ion channel (PDB ID 6BBJ), sampling every 0.5 ns. The one-standard-deviation range is represented by the lighter-coloured band.

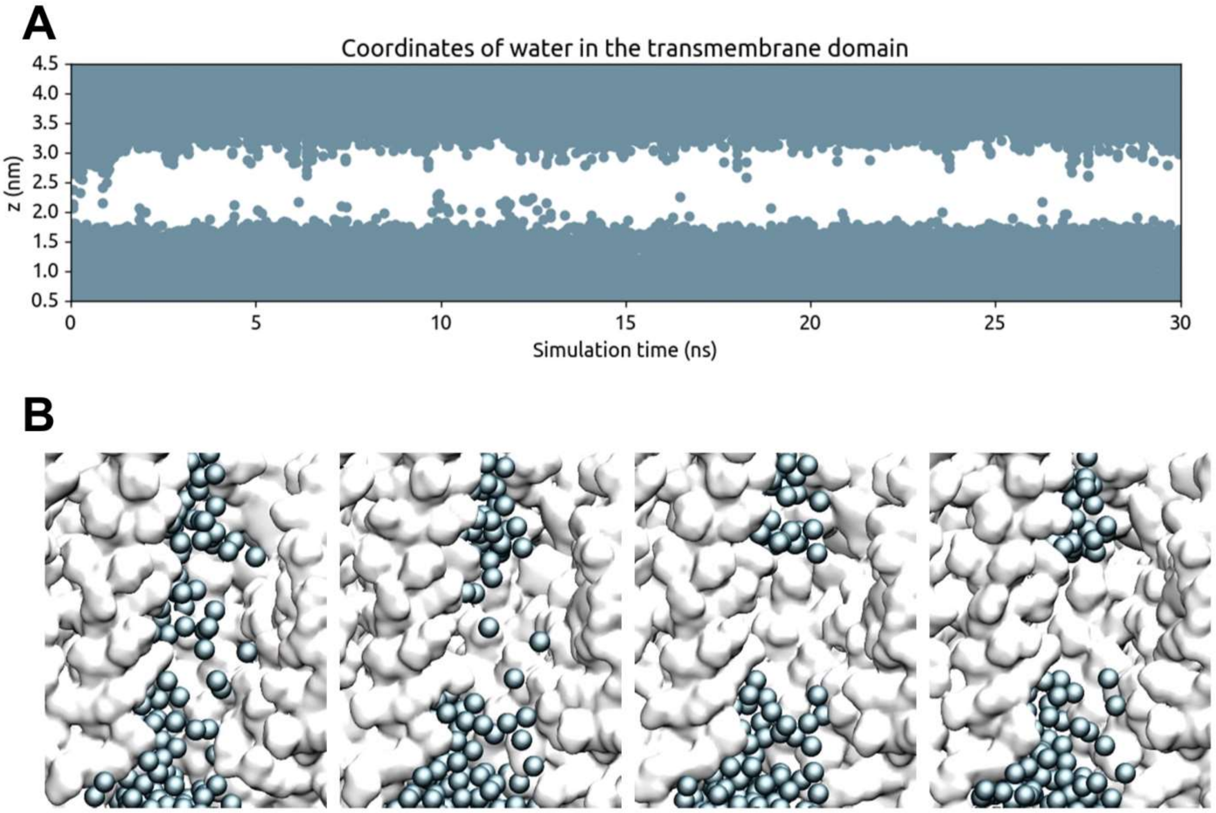

Figure S3. De-wetting of the hydrophobic gate region of the TRPV4 ion channel (PDB ID 6BBJ) in a 30 ns MD simulation. Water molecules are shown as blue spheres, with the protein in a surface representation.

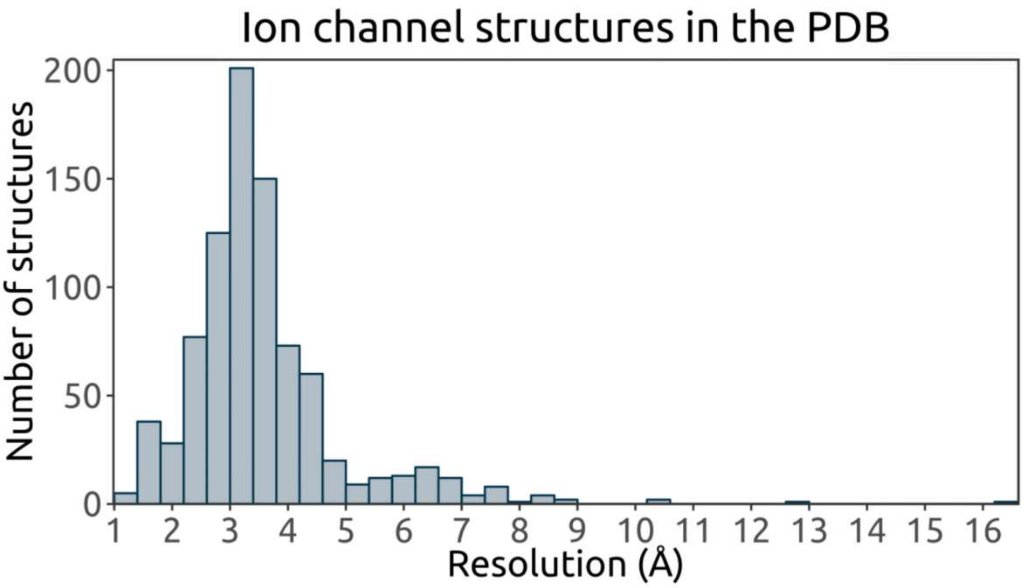

Figure S4. Distribution of resolution of ~860 ion channel structures determined by X-ray crystallography or cryo-electron microscopy in the PDB. ~20 NMR structures have been excluded from this analysis.

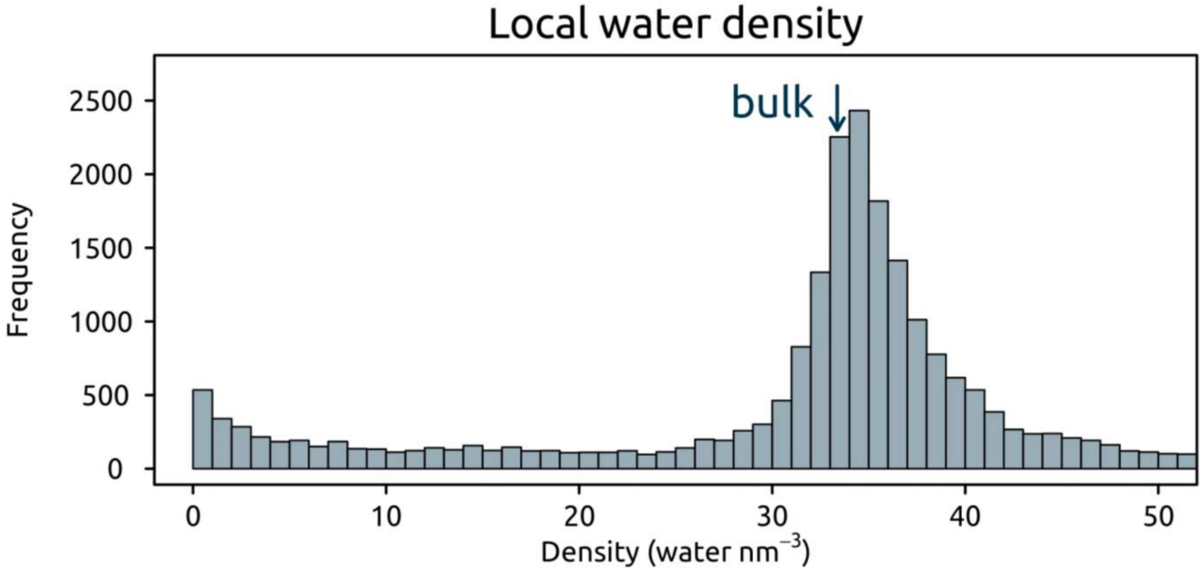

Figure S5. Distribution of local water density values for the dataset of all channel simulation, where the arrow indicates the density of bulk liquid water, at 33 water molecules nm^{-3}.

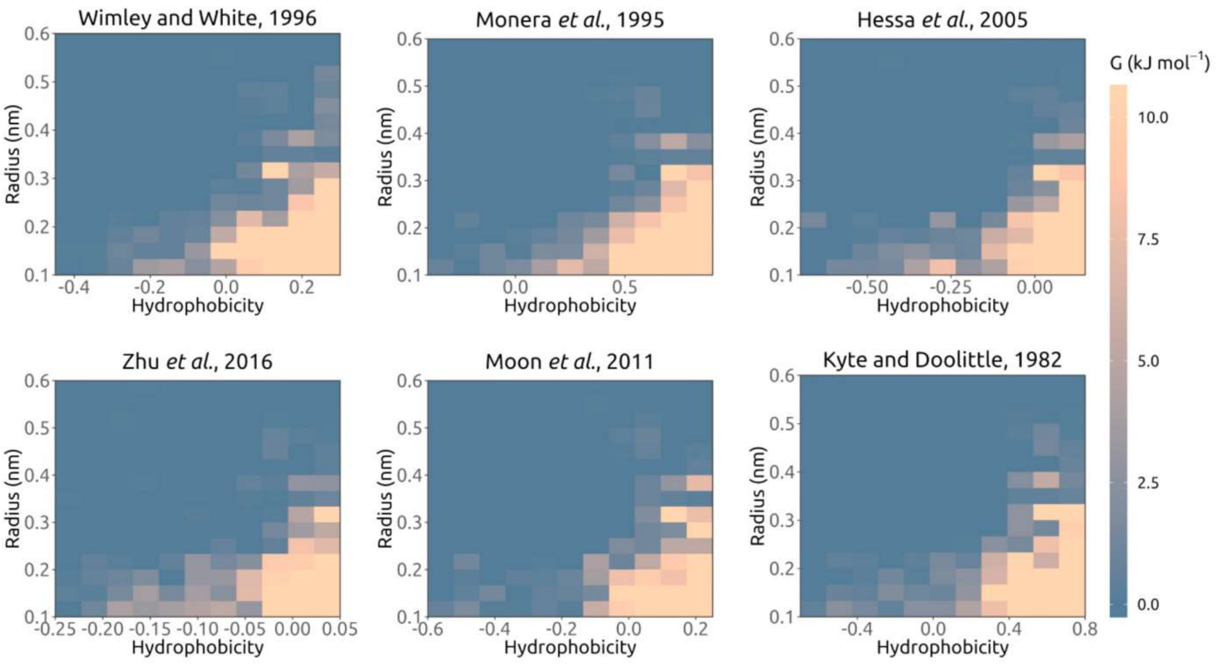

Figure S6. Local free energy as a function of hydrophobicity and pore radius, averaged over all occurrences of pore-lining side chains in the simulated channel structures. Alternative hydrophobicity scales are employed, each linearly normalized such that their respective positions of 0 hydrophobicity are unshifted and the largest absolute value amongst amino acids is equal to 1 (relative) unit, with more positive values indicating greater hydrophobicity. The scales compared are from: (1), (2), (3), (4), (5), and (6).

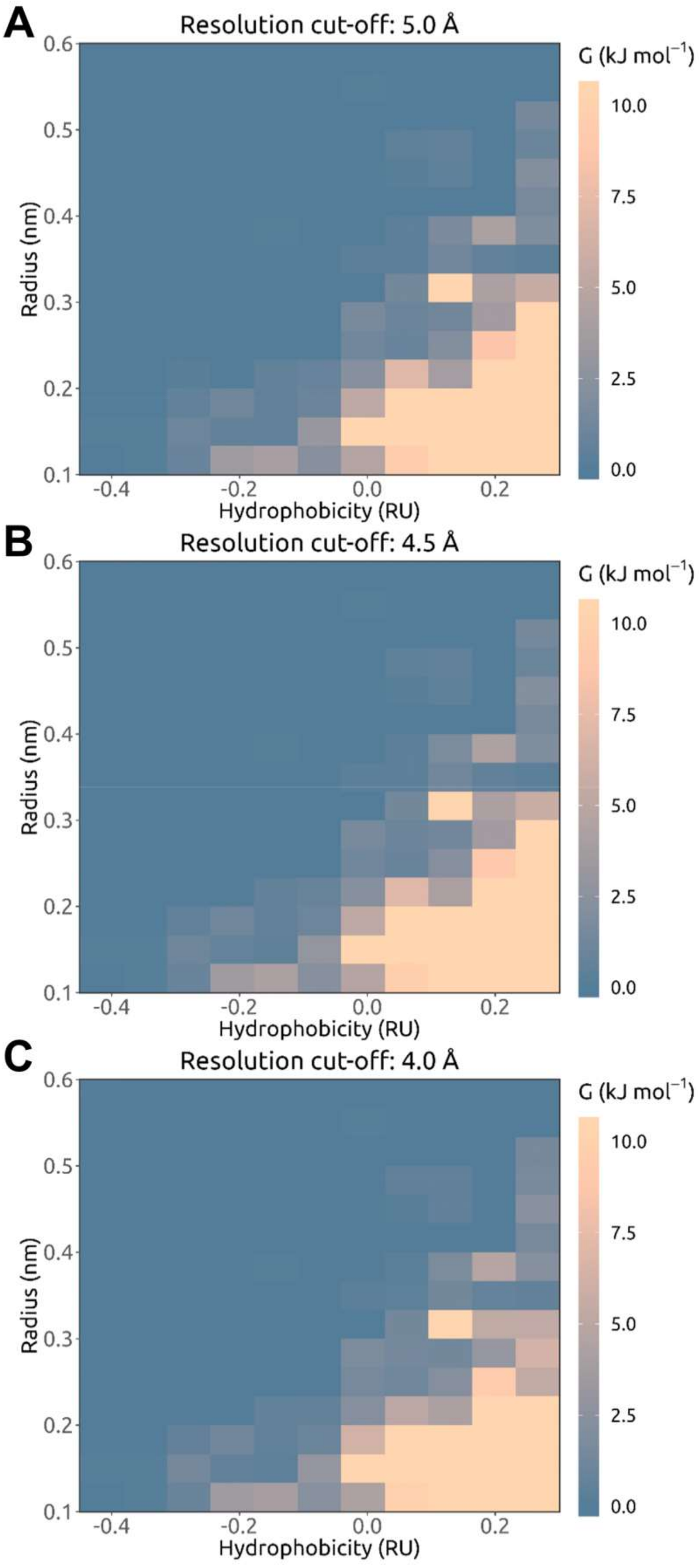

Figure S7. Local free energy as a function of hydrophobicity and pore radius, averaged over all occurrences of pore-lining side chains in (**A**) all simulated channel structures or subsets of simulated structures with resolution better than (**B**) 4.5 Å or (**C**) 4.0 Å.

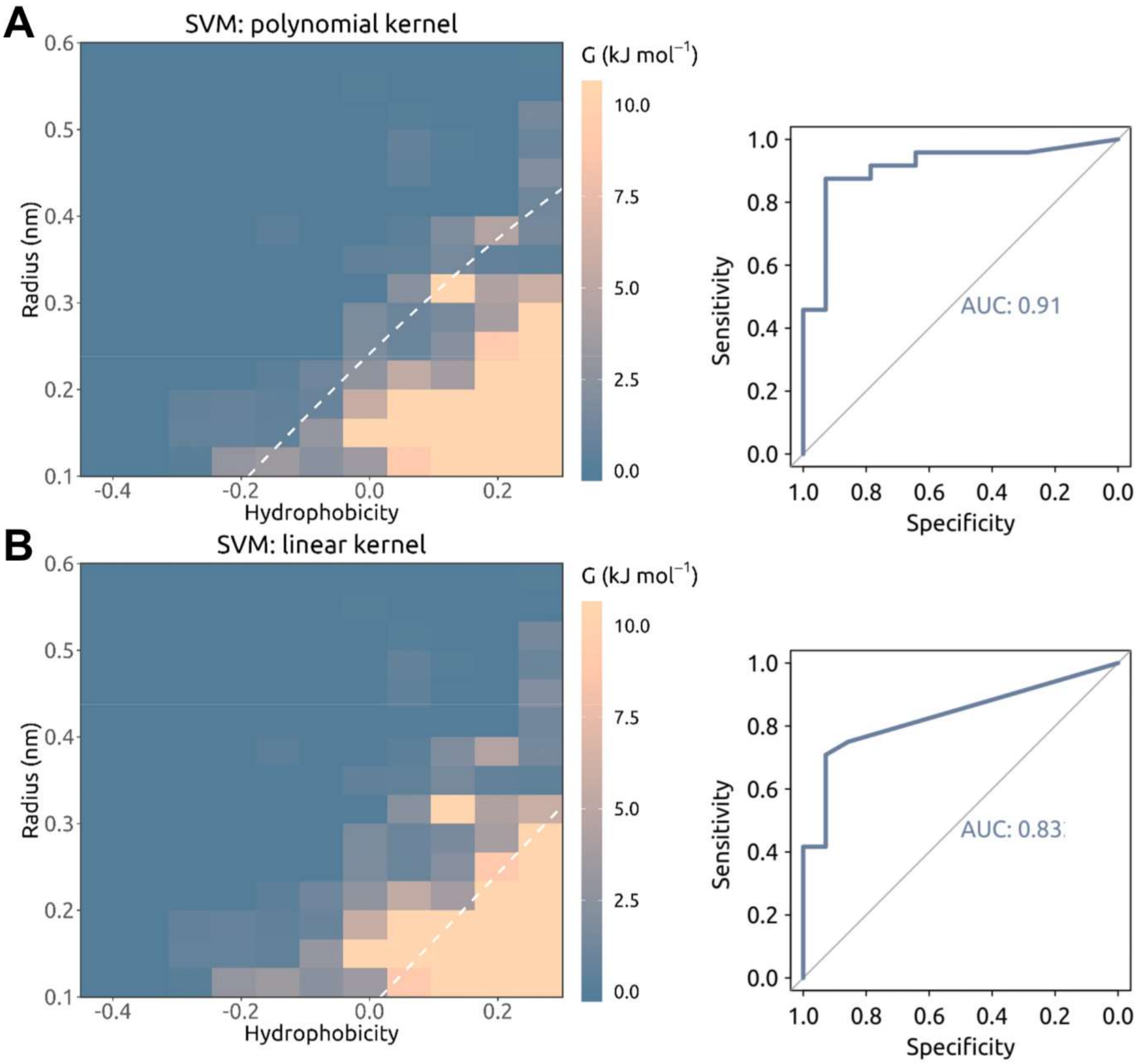

Figure S8. SVM classification of all pore-lining residues in a training set of simulated ion channel structures, using (**A**) a polynomial kernel or (**B**) a linear kernel. Both SVM models were trained using repeated 10-fold cross-validation. The resultant classification lines were used to predict simulation outcomes for a testing set of ~40 structures via calculation of sum-of-shortest-distances scores, with the respective ROC (receiver operating characteristics) curves shown on the right.

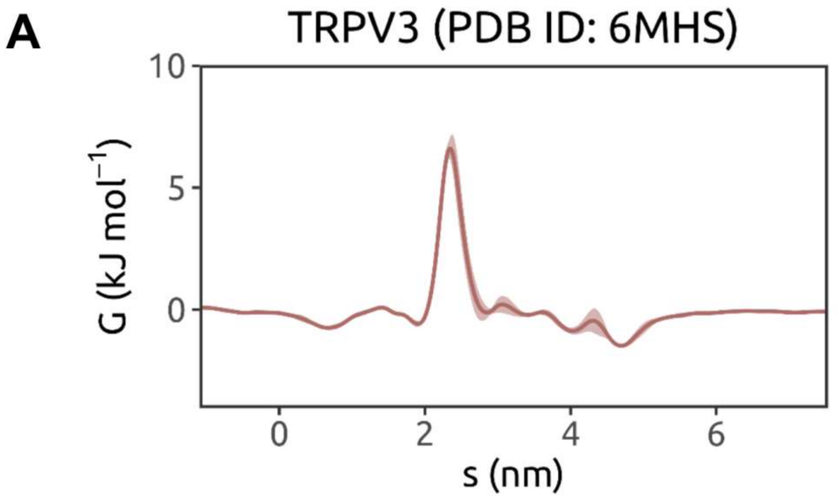

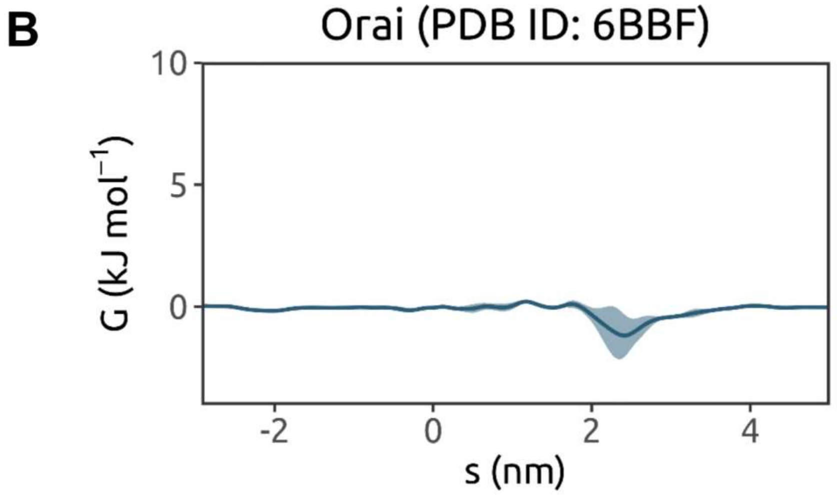

Figure S9. Water free energy profiles derived from triplicate 30 ns MD simulations of water of two recent structures of (**A**) the TRPV3 channel in a non-conductive (sensitized) conformation (PDB ID: 6MHS) and (**B**) the CRAC channel Orai in an open conformation (due to a H206A gain-of-function mutation; PDB ID: 6BBF).

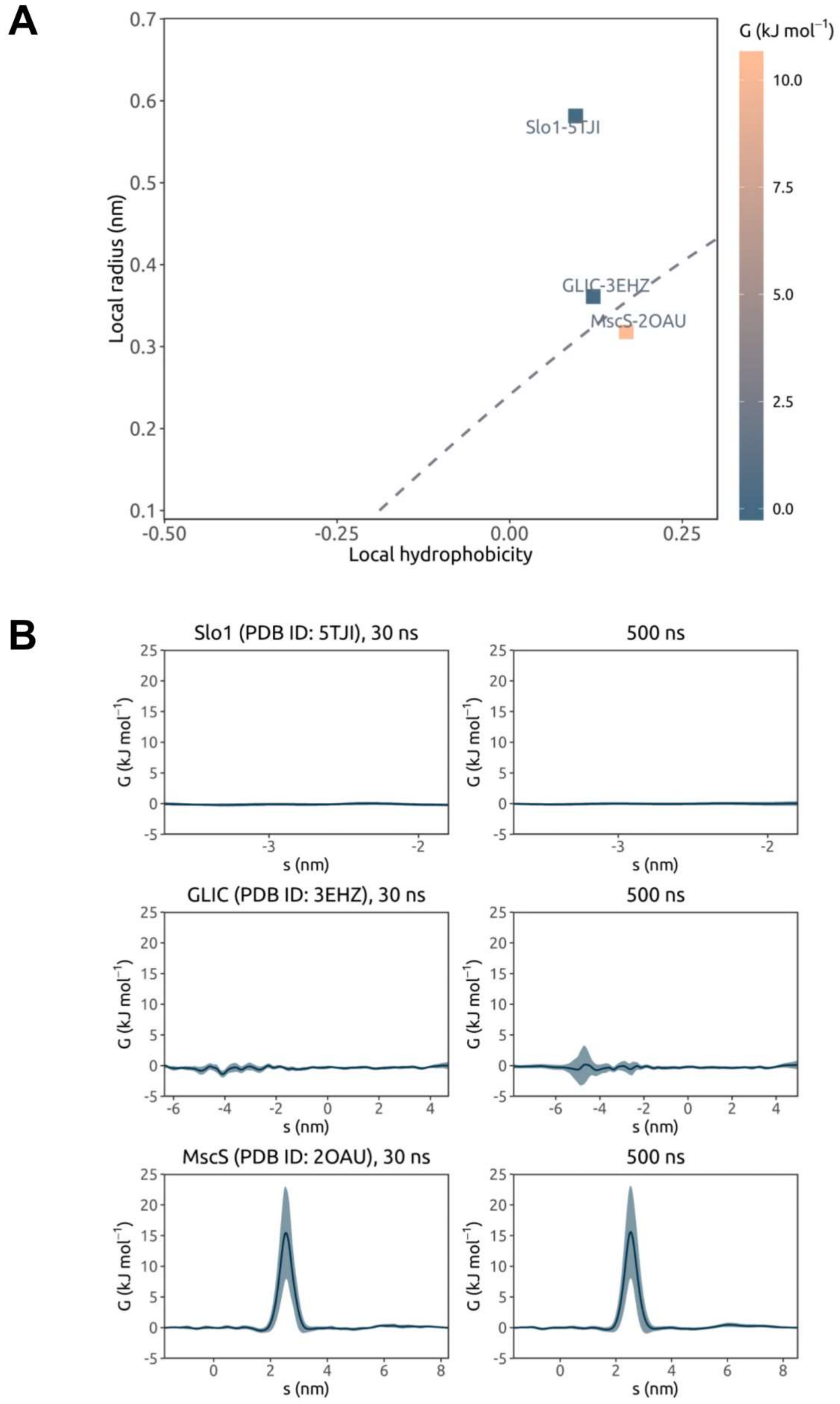

Figure S10. (**A**) Local radius, hydrophobicity, and water free energy at the main energy barrier or putative hydrophobic gate region of three selected ion channel structures, and (**B**) their water free energy profiles derived from 30 ns and 500 ns MD simulations, during which positional restraints were applied to each protein backbone whilst allowing for side chain flexibility.

SI References

1. Wimley CW, White SH (1996) Experimentally determined hydrophobicity scale for proteins at membrane interfaces. *Nature Struct Biol* 3:842-848.
2. Monera OD, et al. (1995) Relationship of sidechain hydrophobicity and alpha-helical propensity on the stability of the single-stranded amphipathic alpha-helix *J Pept Sci* 1:319-329.
3. Hessa T, et al. (2005) Recognition of transmembrane helices by the endoplasmic reticulum translocon *Nature* 433:377-381.
4. Zhu CQ, et al. (2016) Characterizing hydrophobicity of amino acid side chains in a protein environment via measuring contact angle of a water nanodroplet on planar peptide network *Proc Natl Acad Sci USA* 113:12946-12951.
5. Moon CP, Fleming KG (2011) Side-chain hydrophobicity scale derived from transmembrane protein folding into lipid bilayers *Proc Natl Acad Sci USA* 108:10174-10177.
6. Kyte J, Doolittle RF (1982) A simple method for displaying the hydrophobic character of a protein. *J Mol Biol* 157:105-132.
