# Supplementary material for: A heuristic derived from analysis of the ion channel structural proteome permits the rapid identification of hydrophobic gates
Source: Proc Natl Acad Sci U S A. 2019 Jun 24;116(28):13989–95. doi: 10.1073/pnas.1902702116 (PMC6628796; doi:10.1073/pnas.1902702116)
Supplement: Supplementary File [file pnas.1902702116.sd01.pdf]

**Dataset S1. Radius and hydrophobicity profiles for all ion channel structures analysed.** Each channel structure is oriented with the cytoplasmic (or mitochondrial matrix) side in the negative *s*-axis direction. The approximate position of the lipid bilayer membrane, centred at $s = 0$ nm, is indicated by a beige shading. Simulation results are coloured red if the main barrier to water permeation has height > 2.6 kJ $mol^{-1}$ and blue otherwise, with a similar colour divide for the heuristic scores, at a cut-off of 0.55.

www.pnas.org/cgi/doi/10.1073/pnas.1902702116

# 5HT3R (PDB ID: 4PIR)

*Mus musculus*
X−ray (3.5 Å)

Hassaine et al., 2014

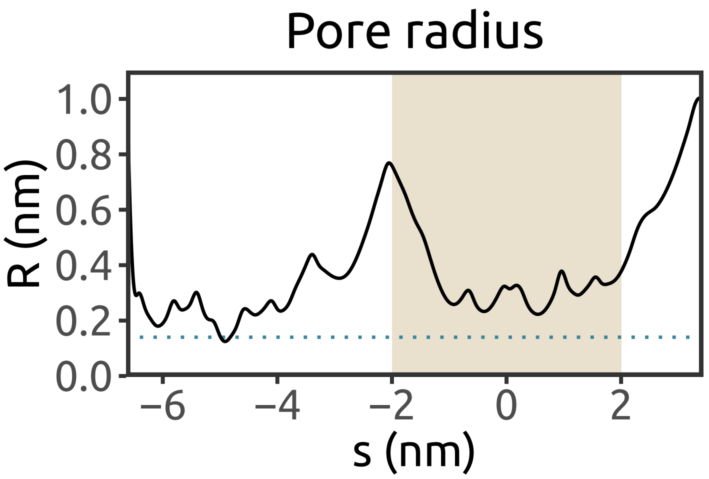


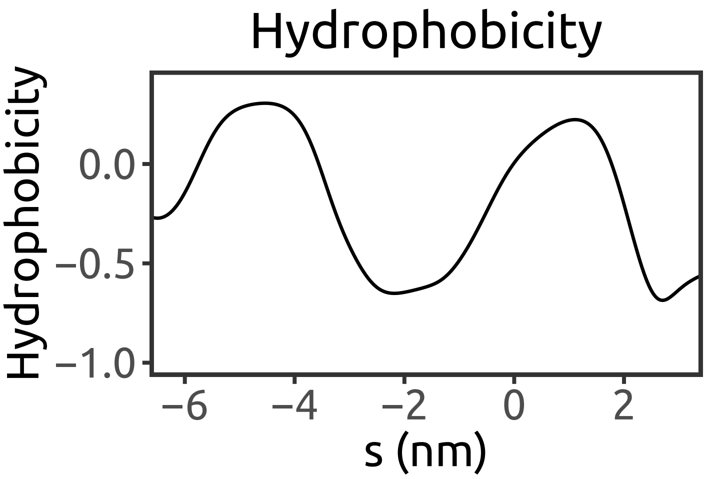


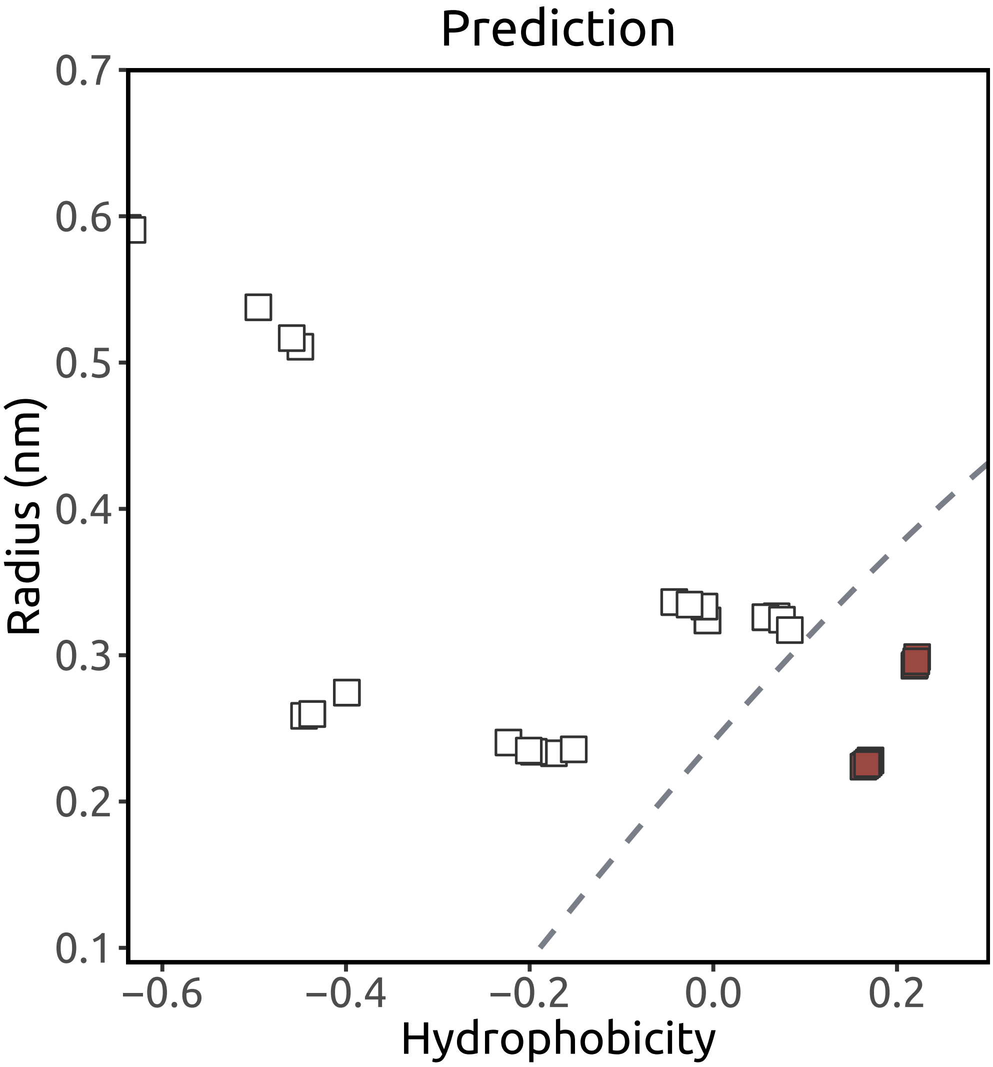


Heuristic score:
0.93 (n = 10)

Simulation result:
barrier to water

# 5HT3R (PDB ID: 6BE1)

*Mus musculus*
cryo−EM (4.31 Å)

Basak et al., 2018

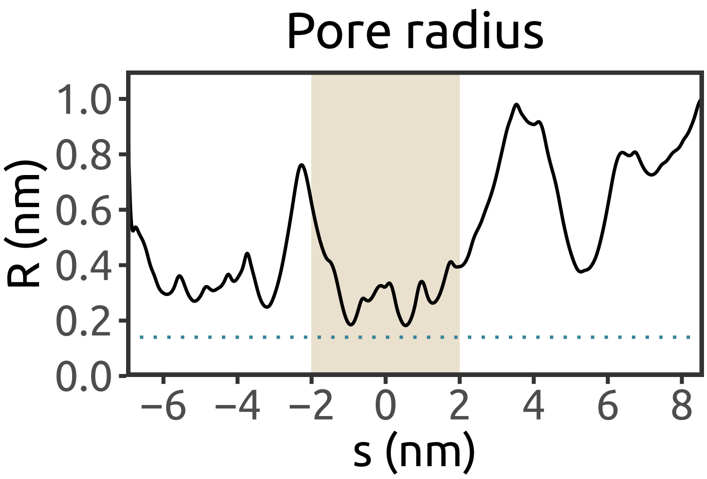


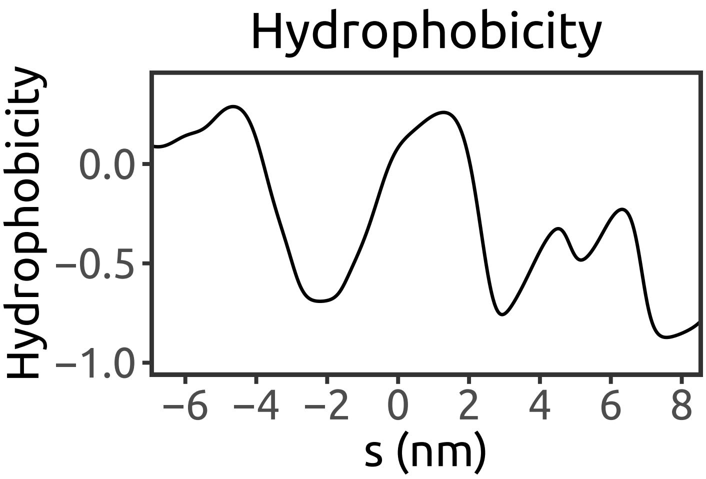


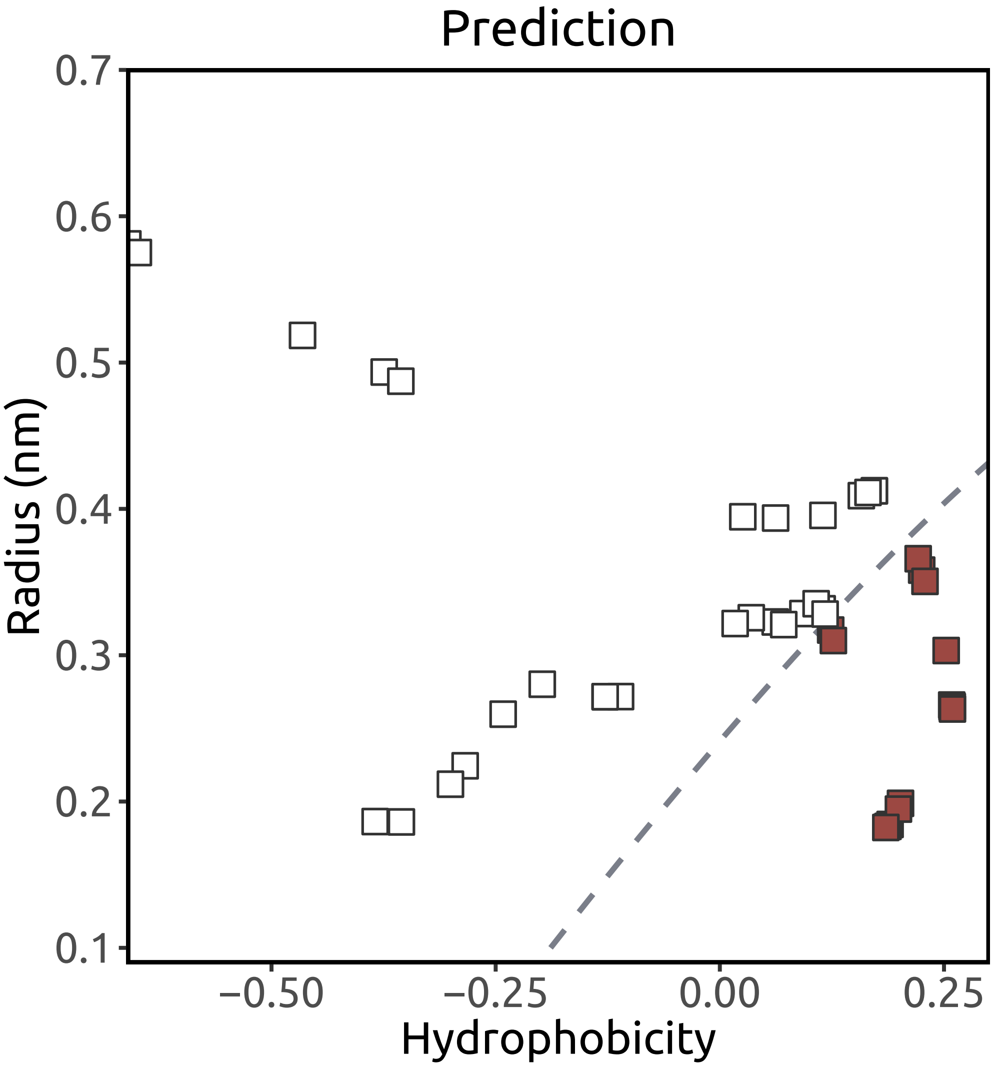


Heuristic score:
1.45 (n = 15)

Simulation result:
barrier to water

# ELIC (PDB ID: 2VL0)

*Erwinia chrysanthemi*

X–ray (3.3 Å)

Hilf & Dutzler, 2008

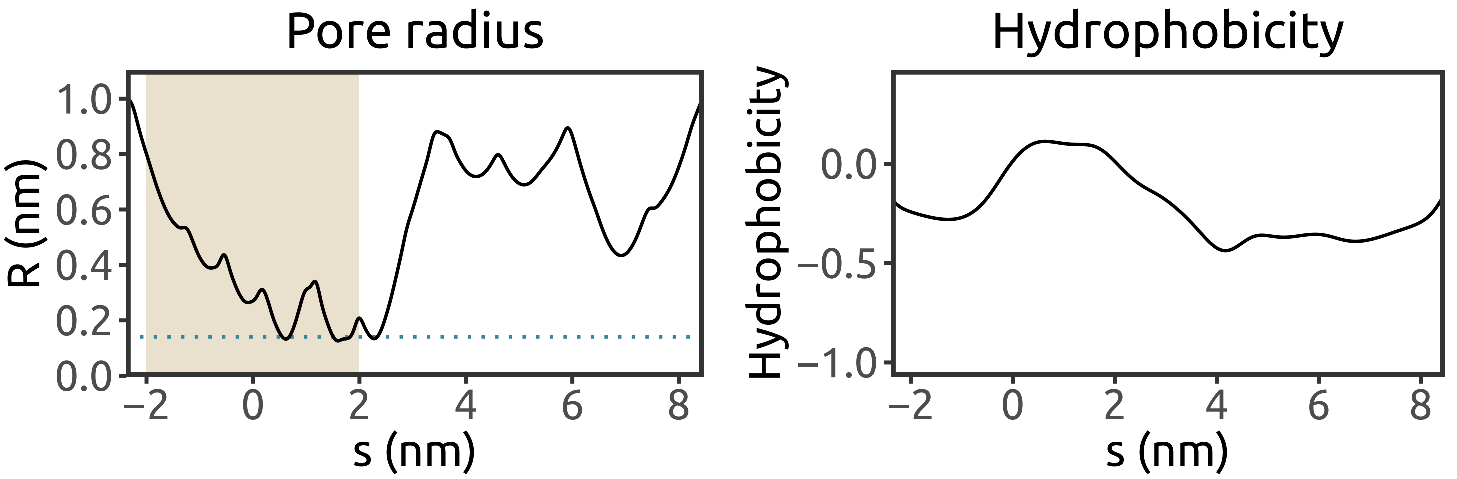


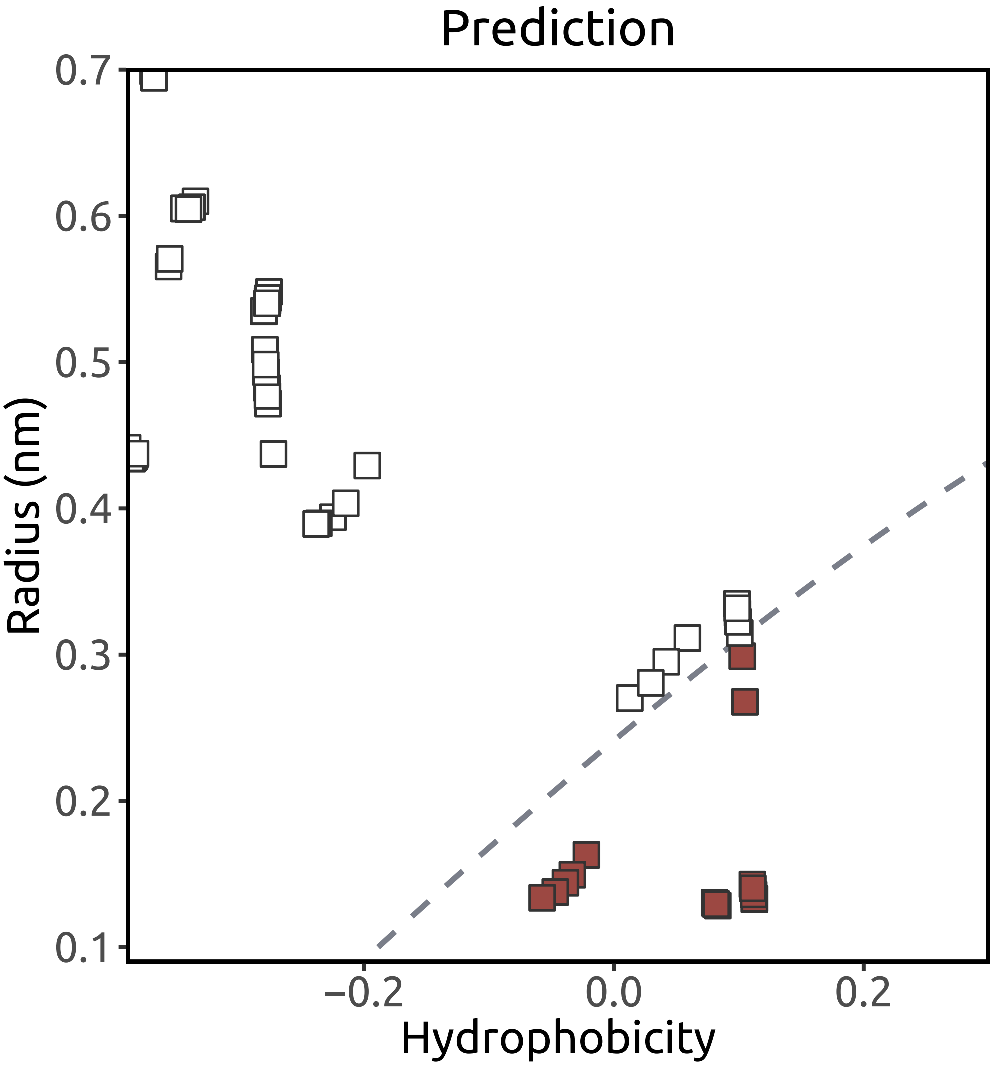


Heuristic score:

1.78 (n = 19)

Simulation result:

barrier to water

# ELIC (PDB ID: 3RQW)

*Erwinia chrysanthemi*

X−ray (2.91 Å)

Pan et al., 2012

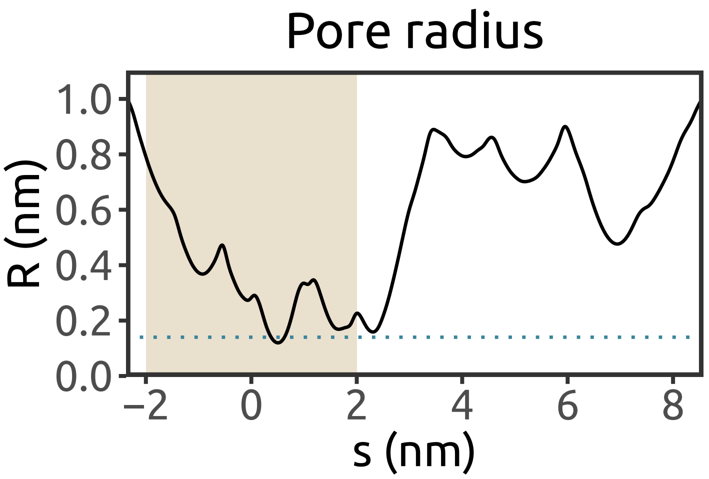


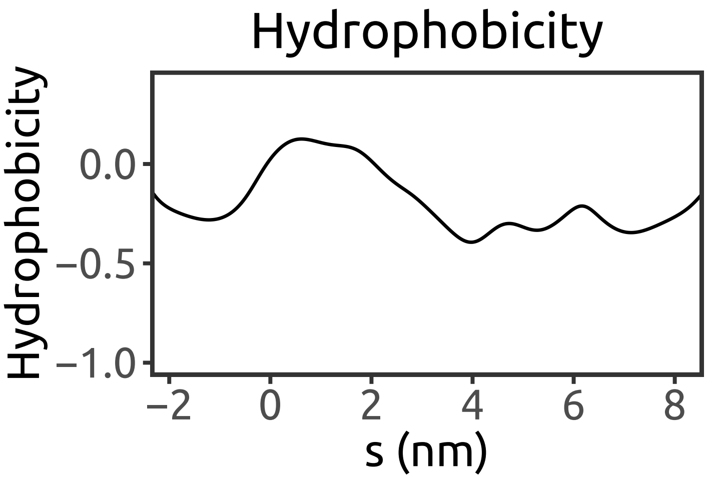


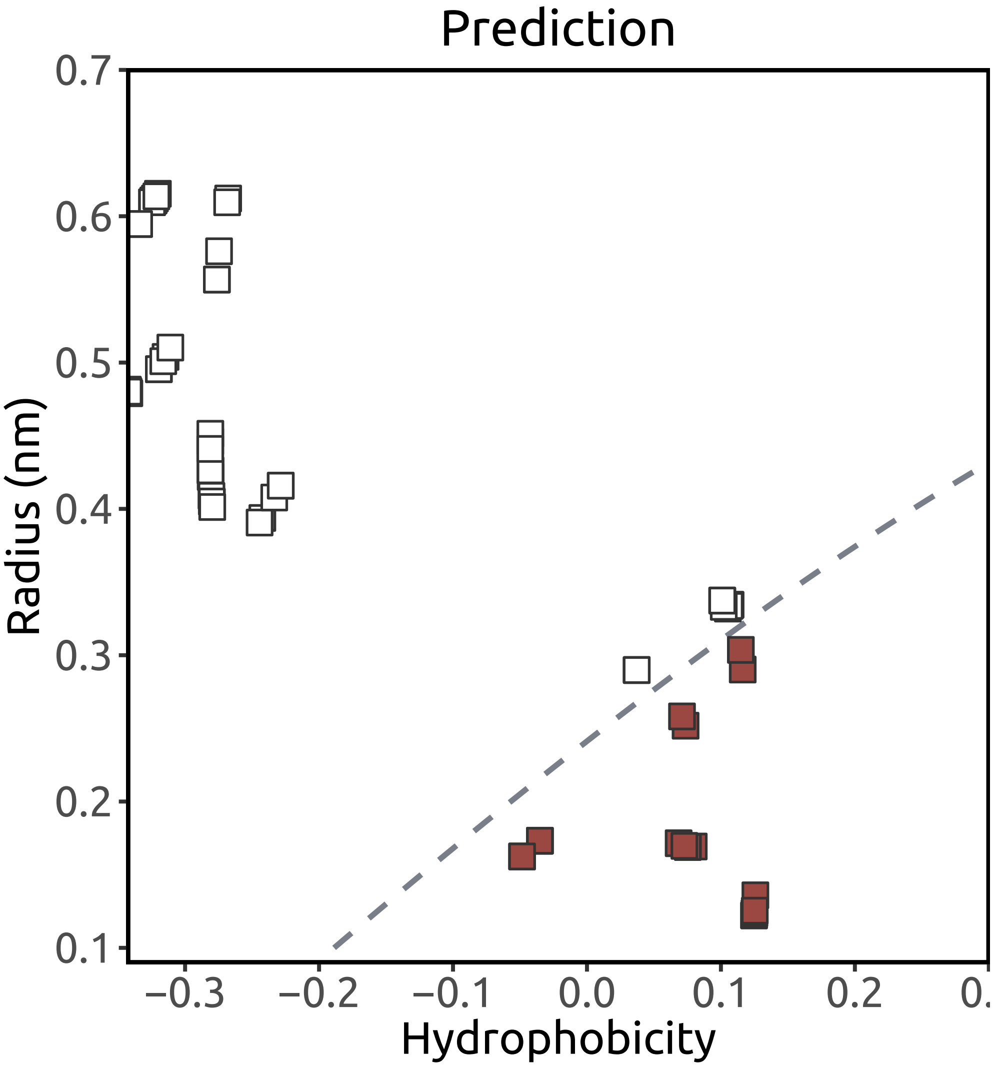


Heuristic score:

1.61 (n = 19)

Simulation result:

barrier to water

# GABAAR (PDB ID: 4COF)

*Homo sapiens*
X−ray (2.97 Å)

Miller et al., 2014

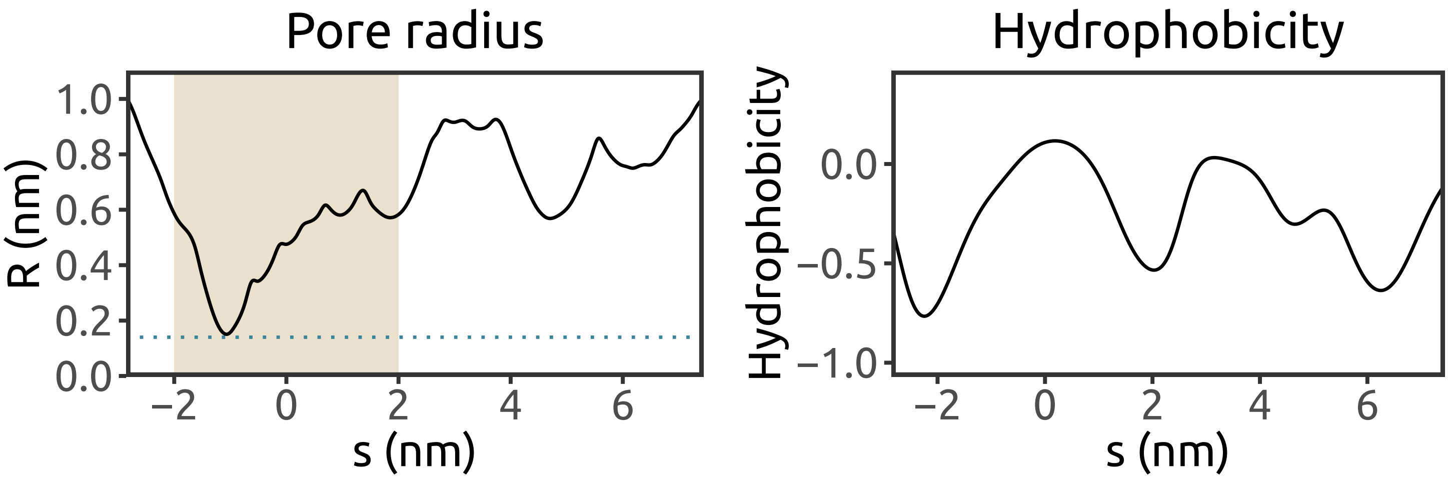


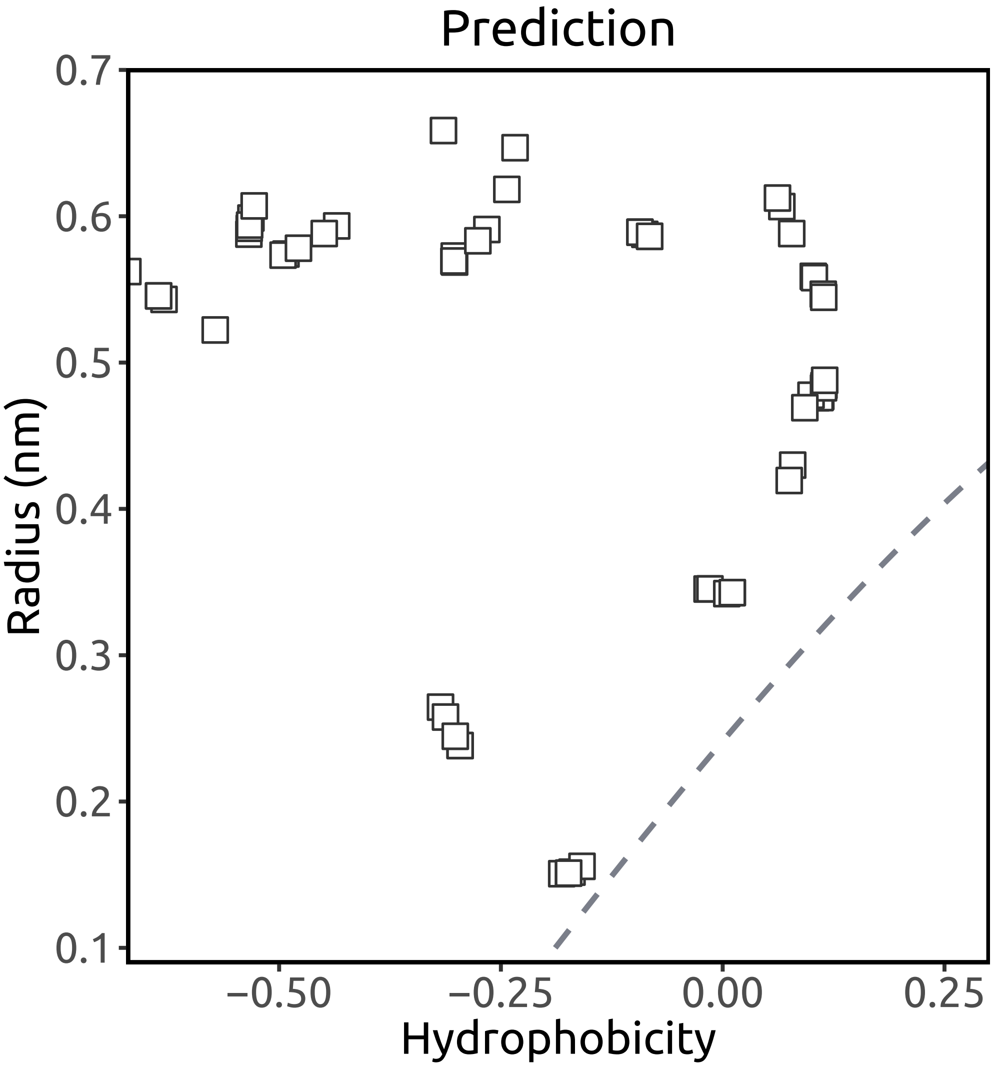


Heuristic score:
0 (n = 0)

Simulation result:
hydrated channel

# GABAAR (PDB ID: 6A96)

*Homo sapiens*
cryo−EM (3.51 Å)

Liu et al., 2018

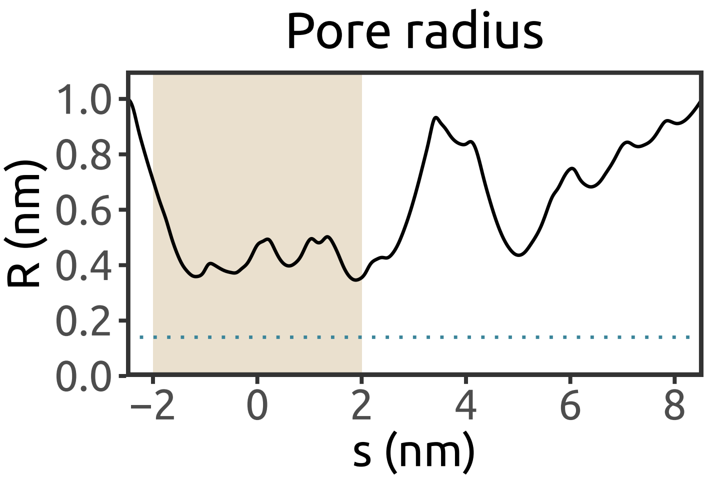


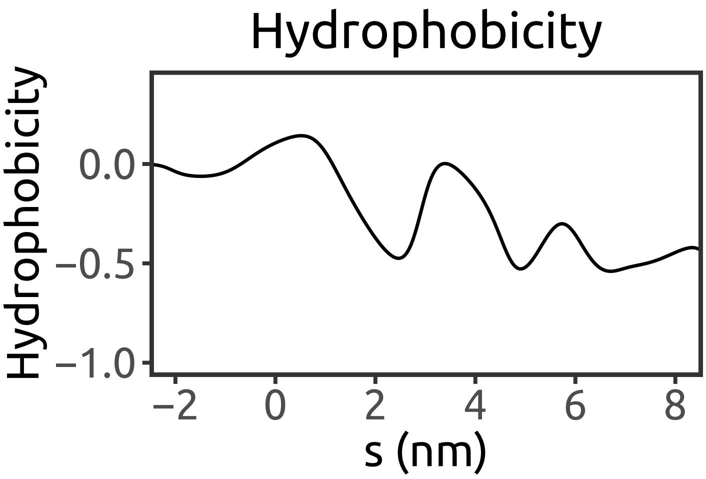


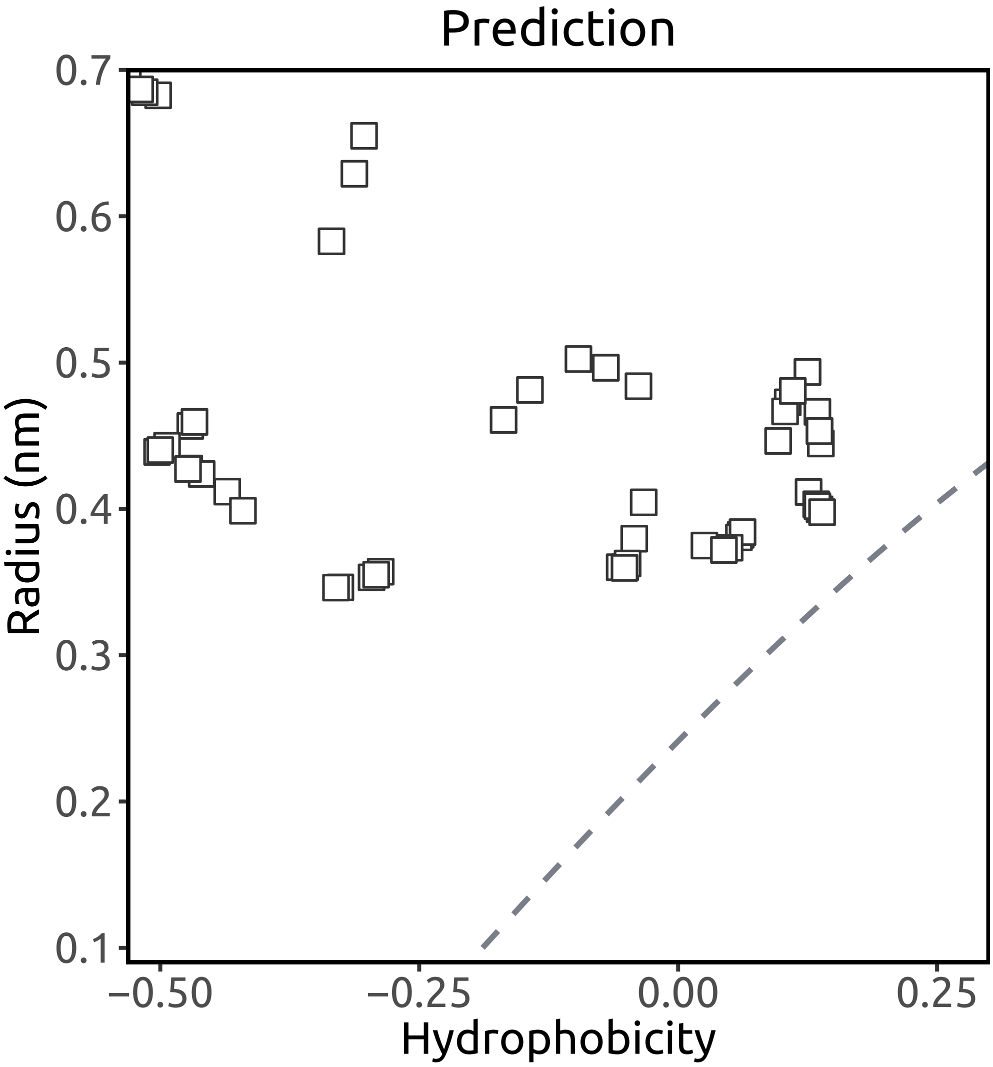


Heuristic score:
0 (n = 0)

Simulation result:
hydrated channel

# GABAAR (PDB ID: 6D6T)

*Homo sapiens*

cryo−EM (3.86 Å)

Zhu et al., 2018

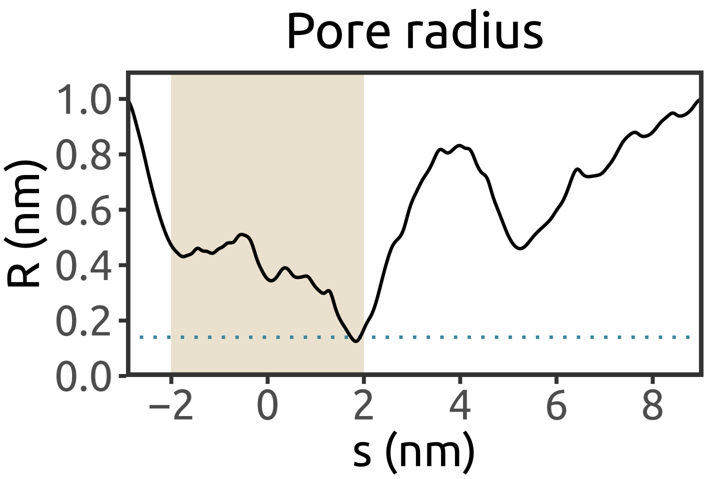


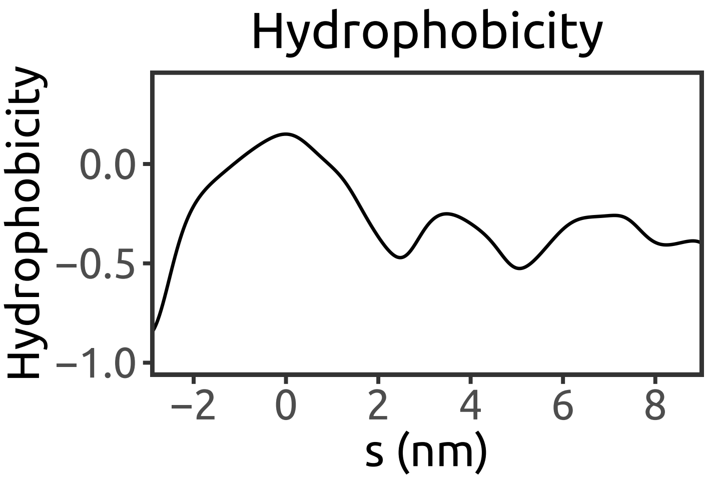


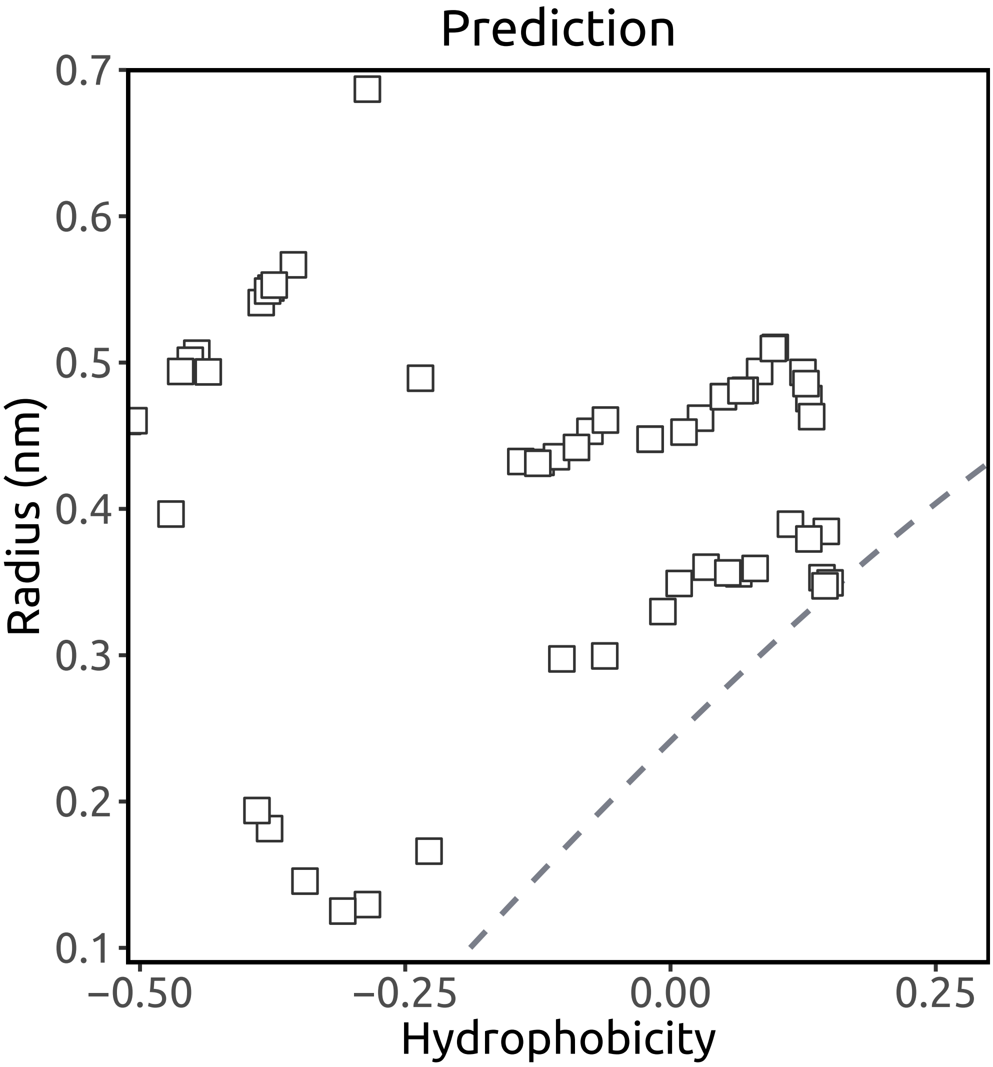


Heuristic score:

0 (n = 0)

Simulation result:

hydrated channel

# GABAAR (PDB ID: 6D6U)

*Homo sapiens*
cryo–EM (3.92 Å)

Zhu et al., 2018

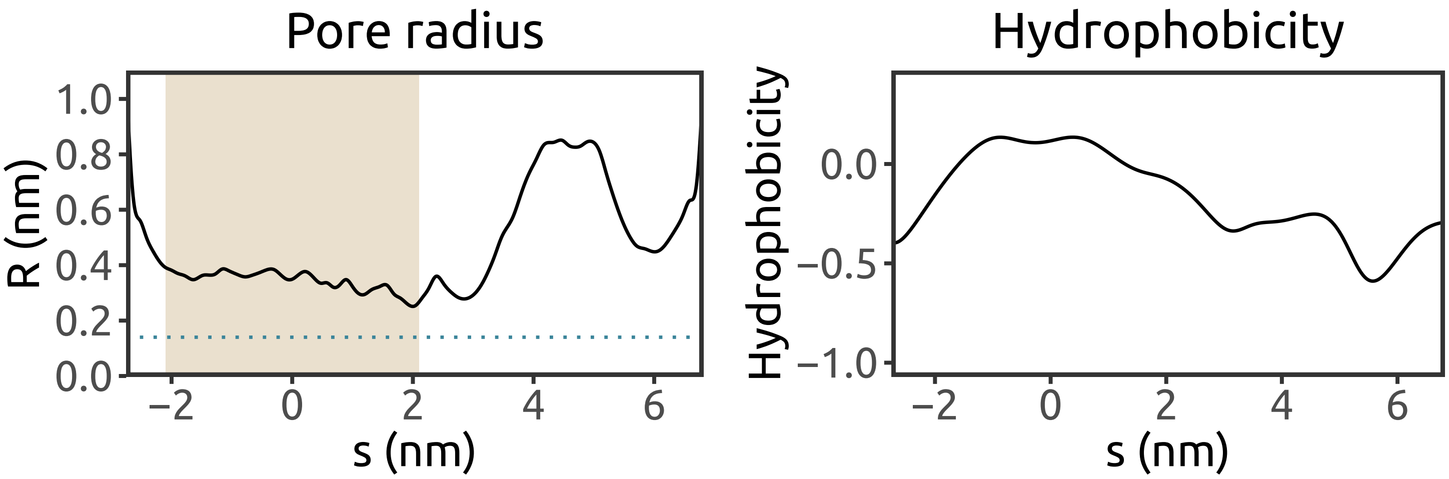


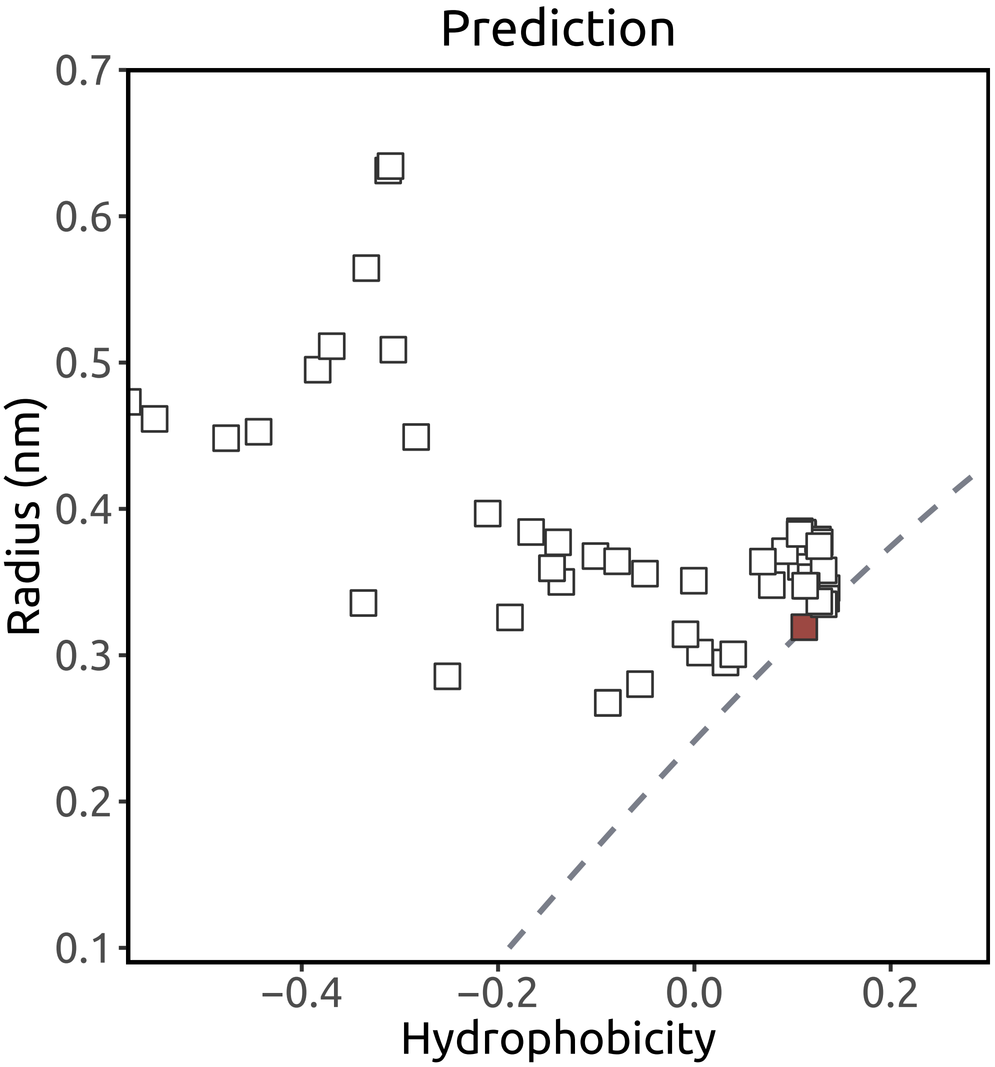


Heuristic score:
0 (n = 1)

Simulation result:
barrier to water

# GLIC (PDB ID: 2XQ7)

*Gloeobacter violaceus*

X–ray (3.4 Å)

Hilf et al., 2010

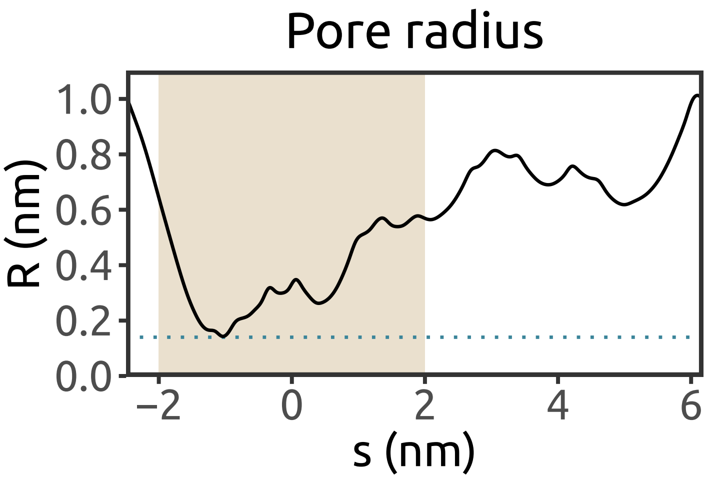


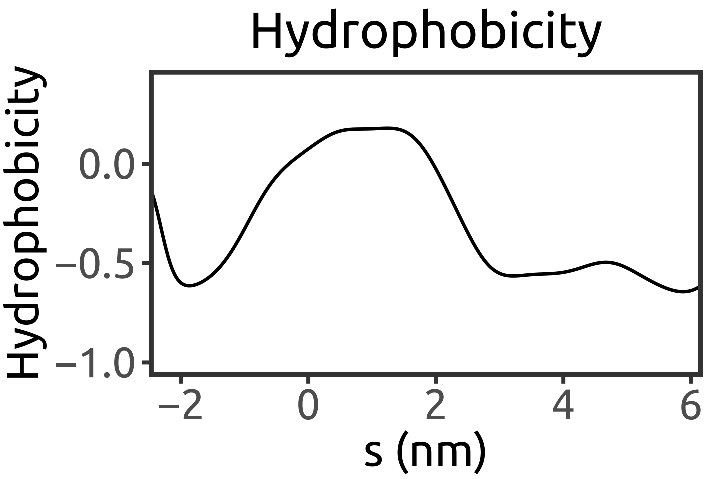


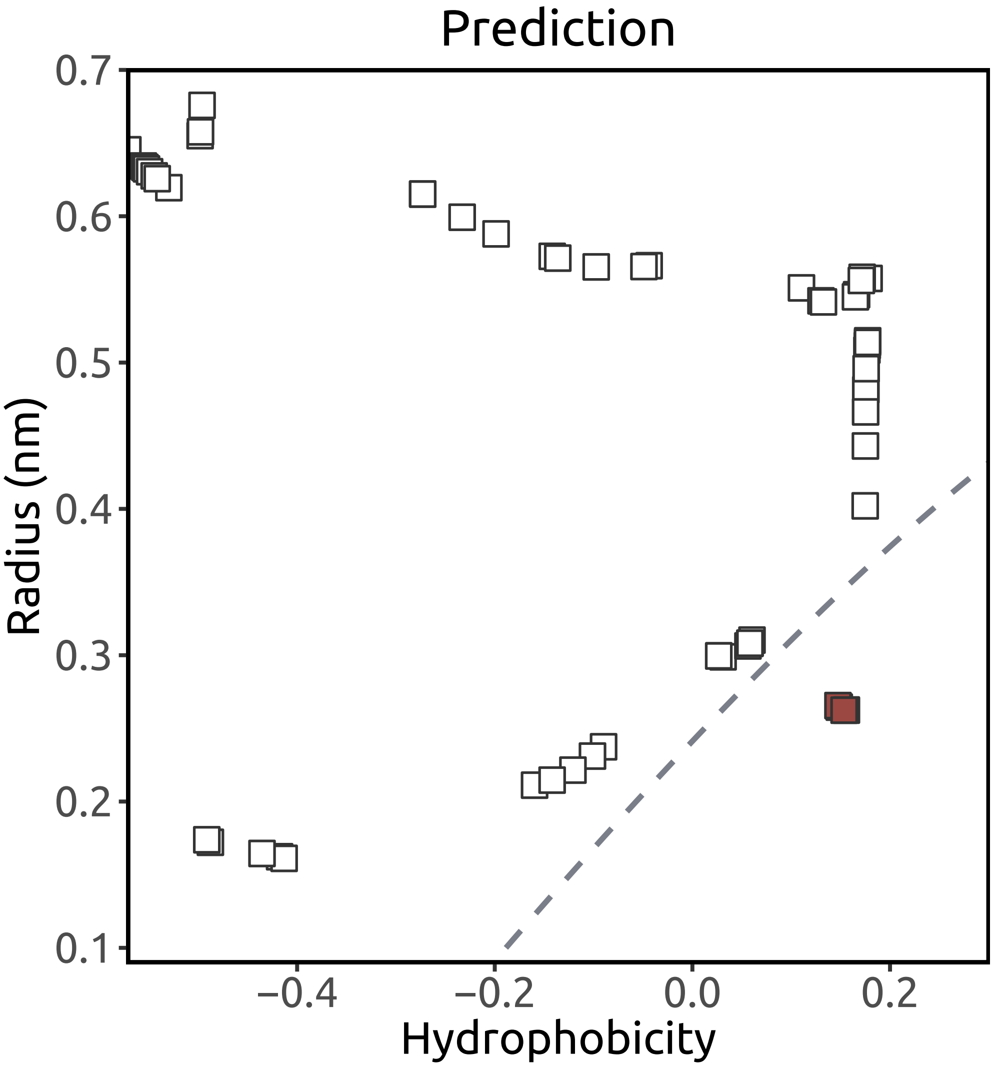


Heuristic score:

0.34 (n = 5)

Simulation result:

hydrated channel

# GLIC (PDB ID: 3EHZ)

*Gloeobacter violaceus*

X–ray (3.1 Å)

Hilf & Dutzler, 2009

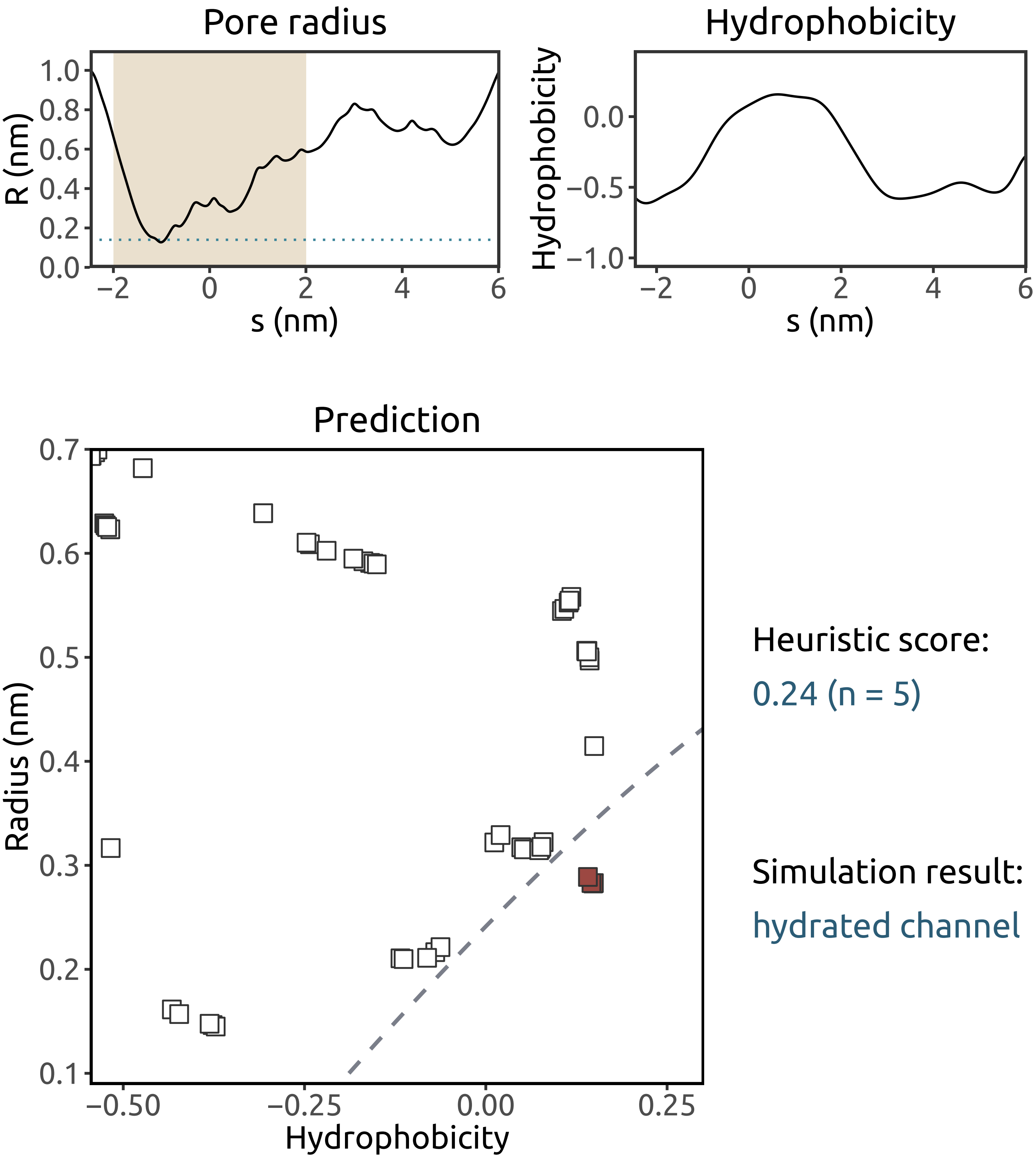


Heuristic score:

0.24 (n = 5)

Simulation result:

hydrated channel

# GLIC (PDB ID: 4F8H)

*Gloeobacter violaceus*
X−ray (2.99 Å)

Pan et al., 2012

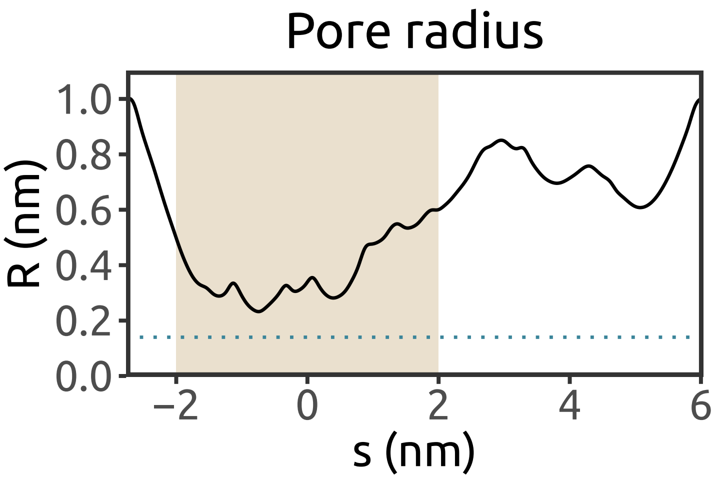


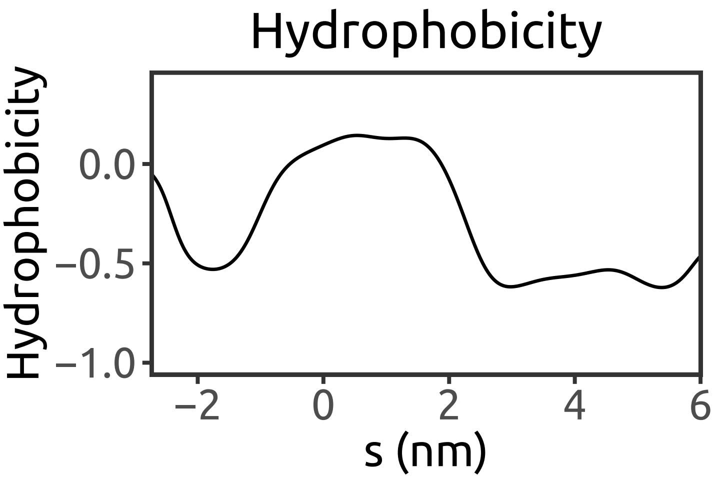


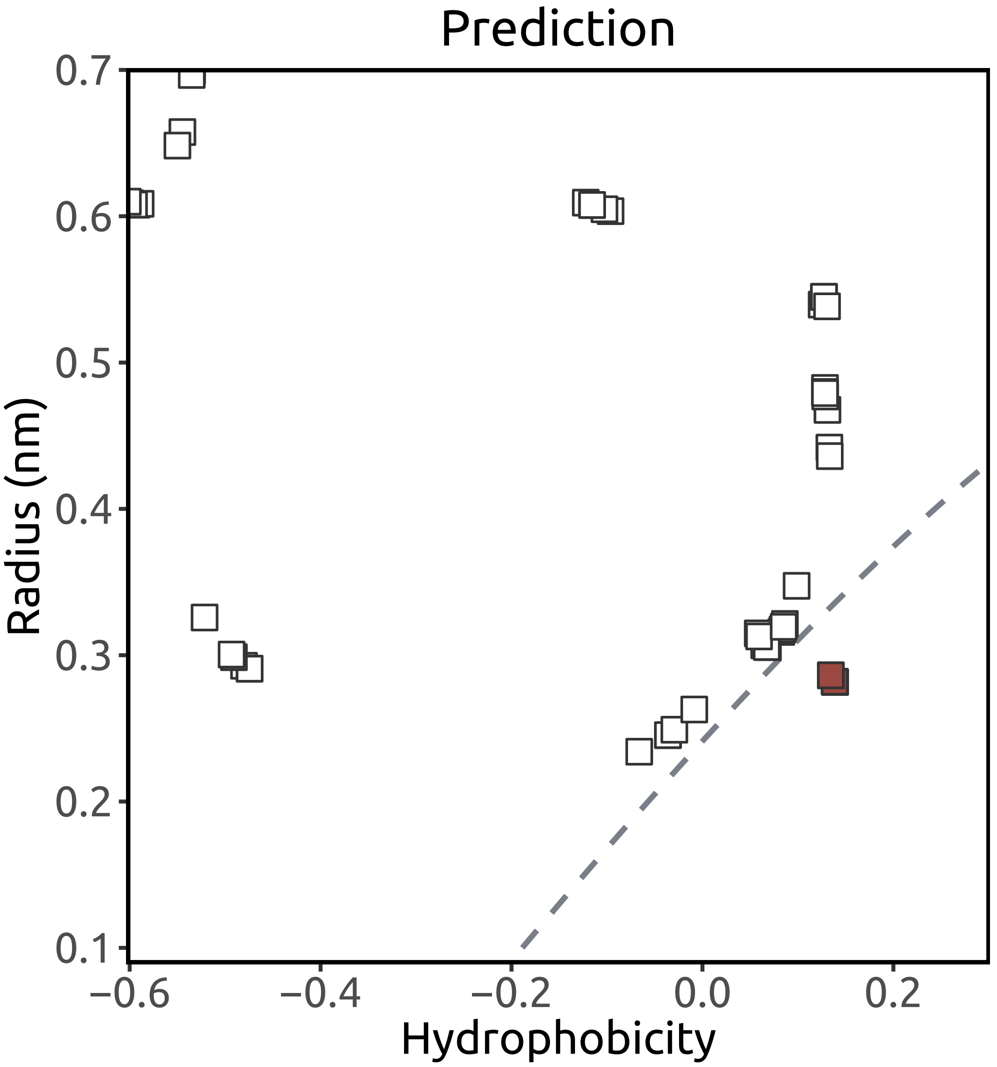


Heuristic score:
0.21 (n = 5)

Simulation result:
hydrated channel

# GLIC (PDB ID: 4HFI)

*Gloeobacter violaceus*

X−ray (2.4 Å)

Sanguet et al., 2013

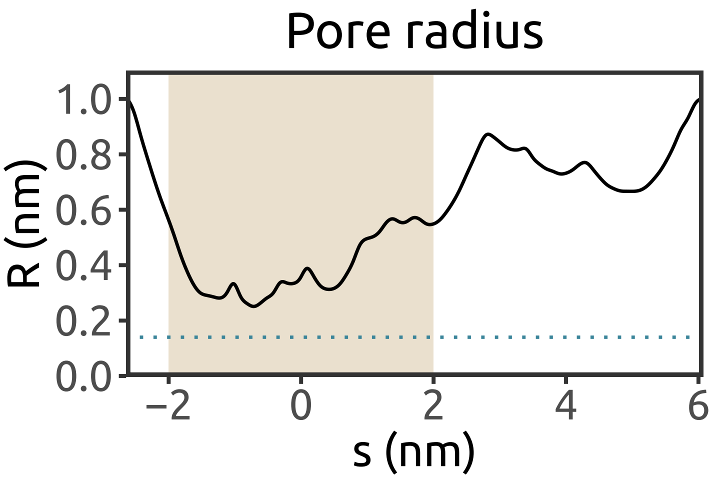


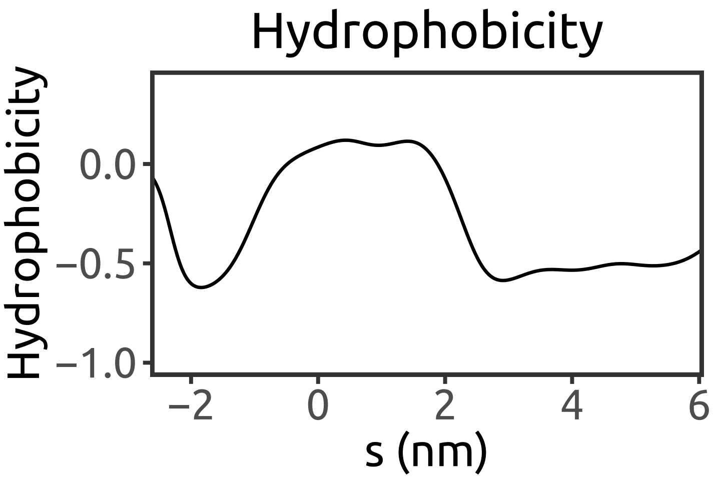


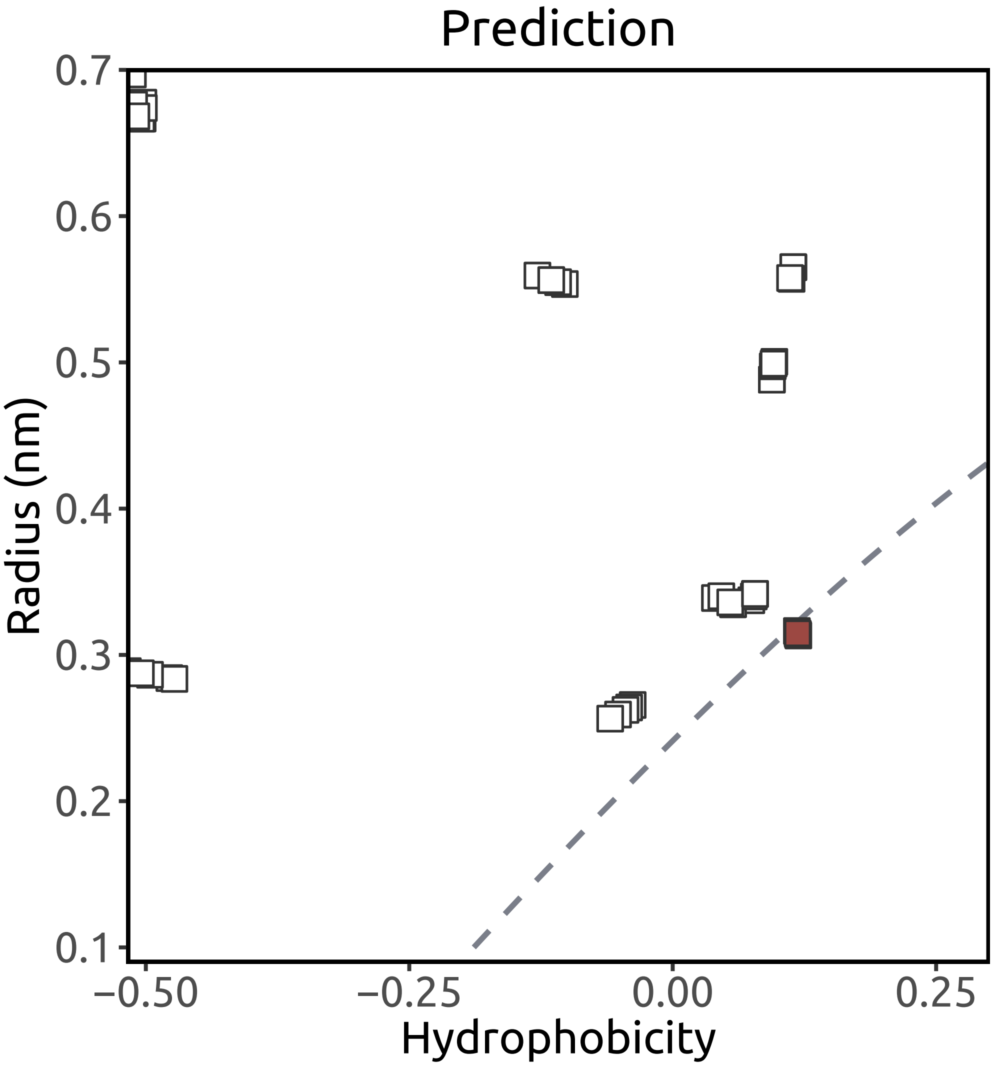


Heuristic score:

0.04 (n = 5)

Simulation result:

hydrated channel

# GLIC (PDB ID: 4NPP)

*Gloeobacter violaceus*

X−ray (3.35 Å)

Sanguet et al., 2014

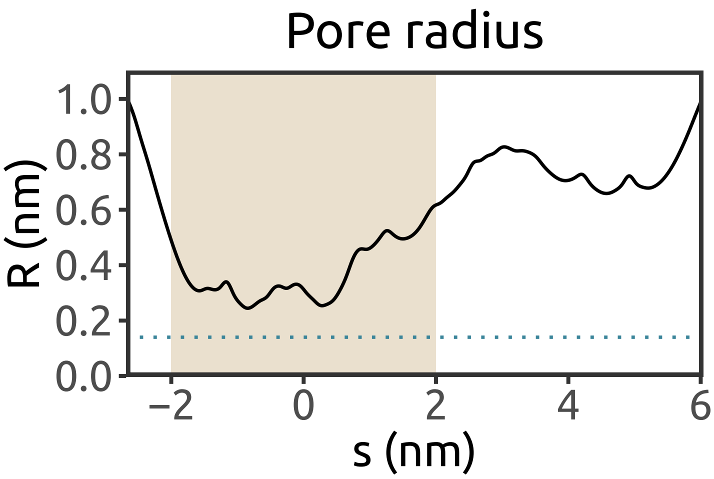


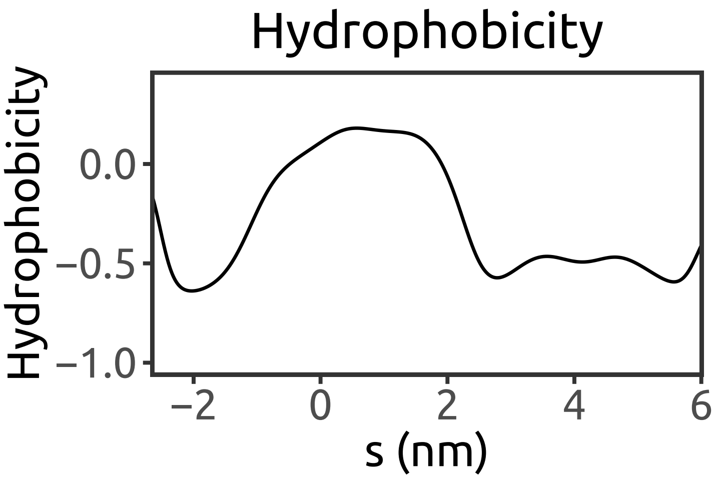


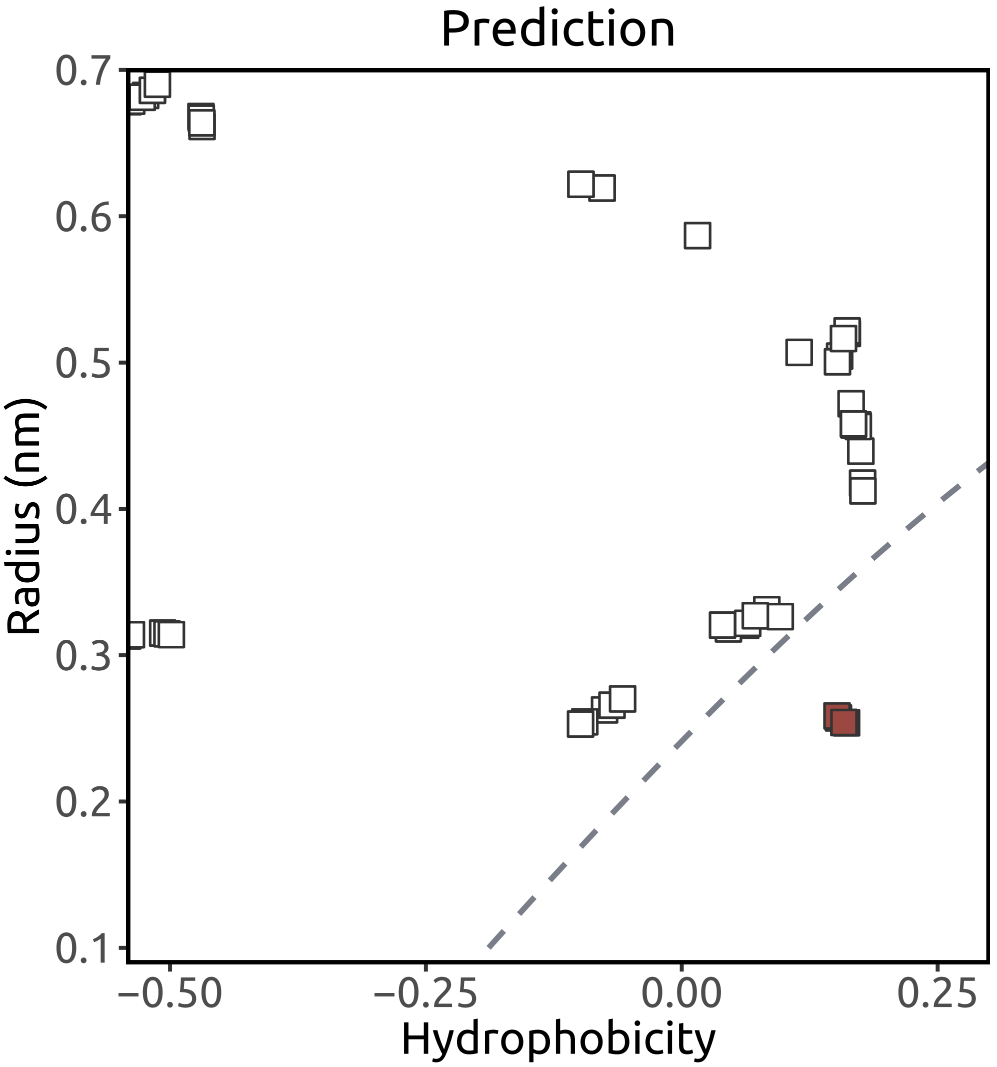


Heuristic score:

0.39 (n = 5)

Simulation result:

hydrated channel

# GLIC (PDB ID: 4QH5)

*Gloeobacter violaceus*
X–ray (3 Å)

Fourati et al., 2015

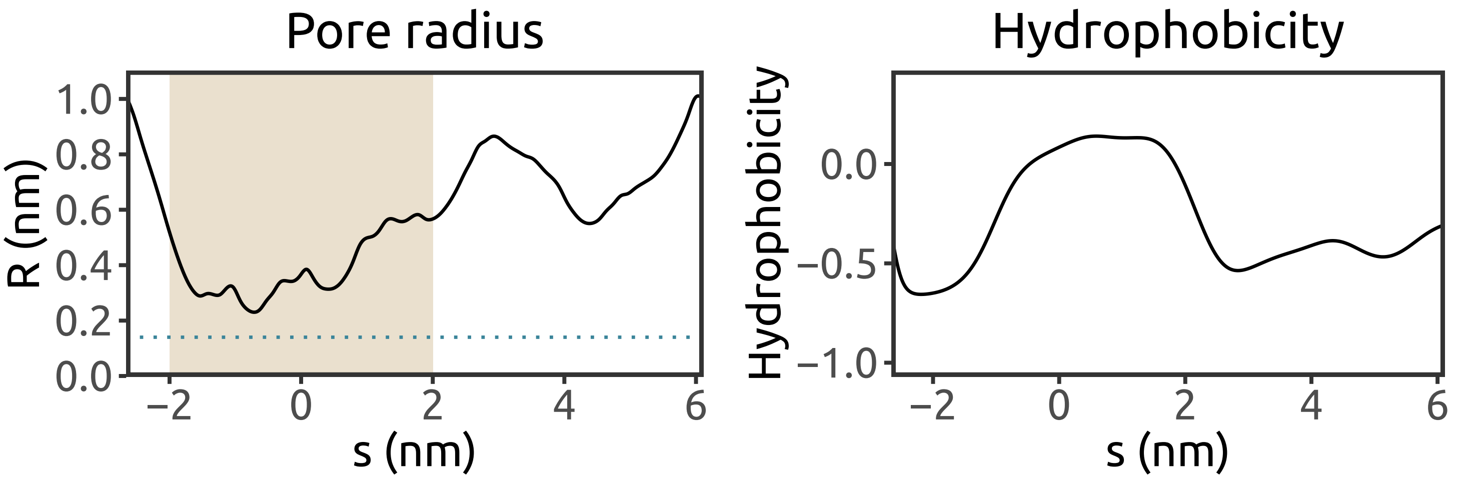


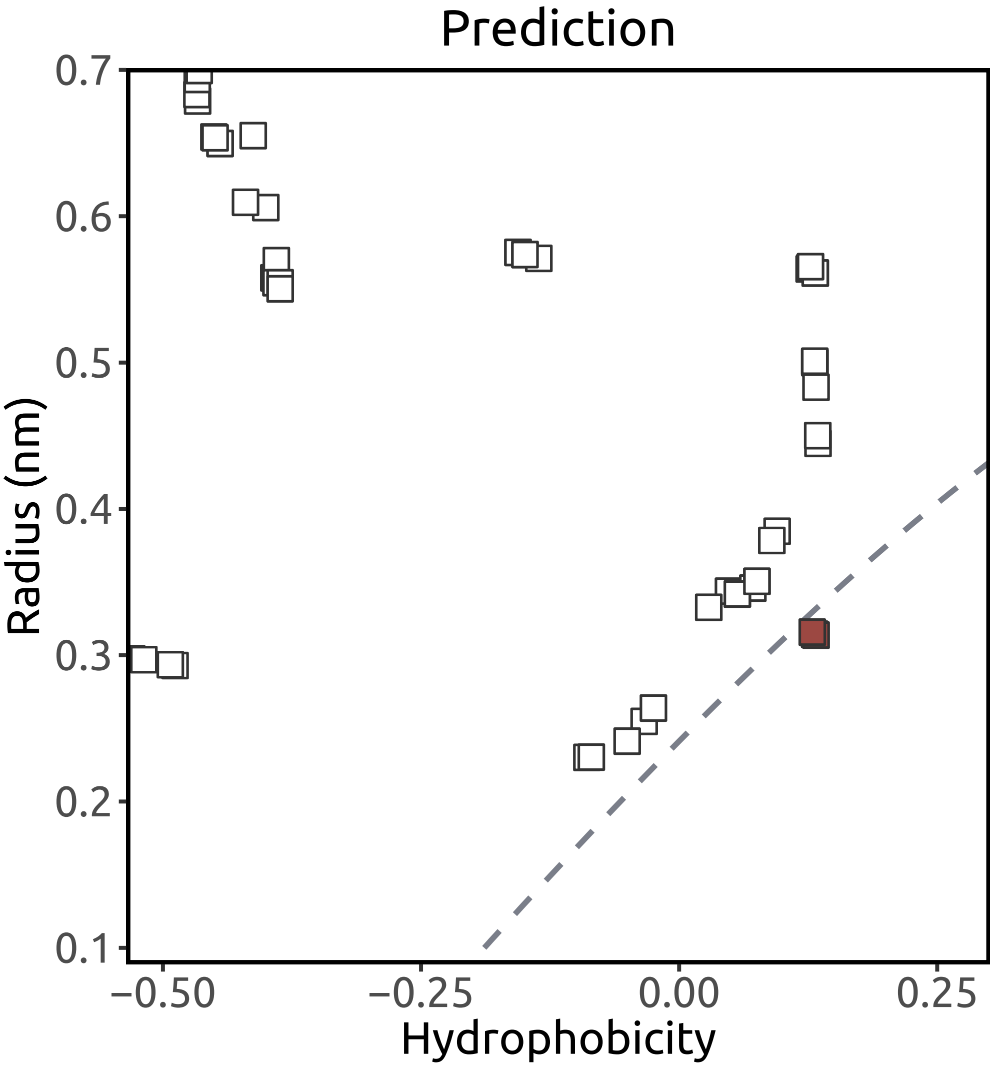


Heuristic score:
0.07 (n = 5)

Simulation result:
hydrated channel

# GLIC (PDB ID: 5J0Z)

*Gloeobacter violaceus*

X−ray (3.25 Å)

Basak et al., 2017

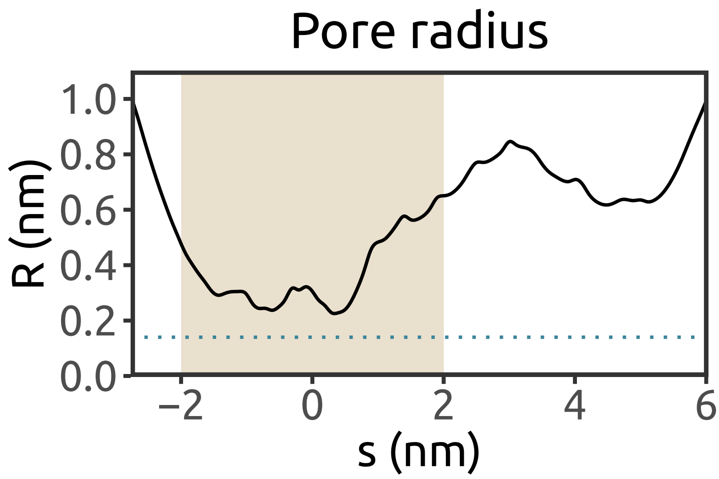


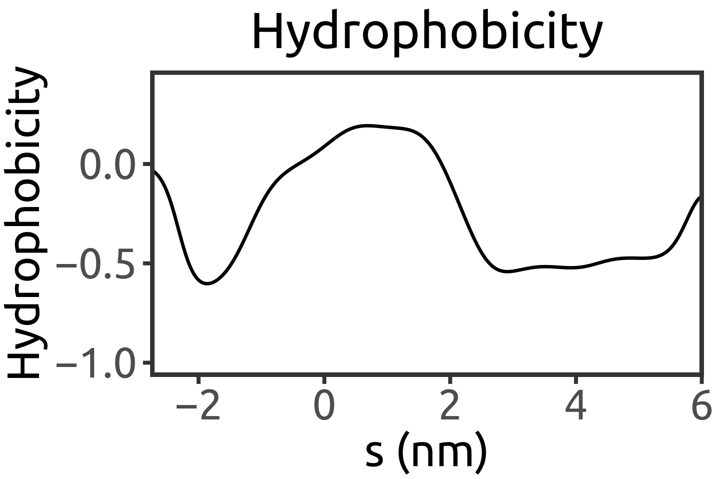


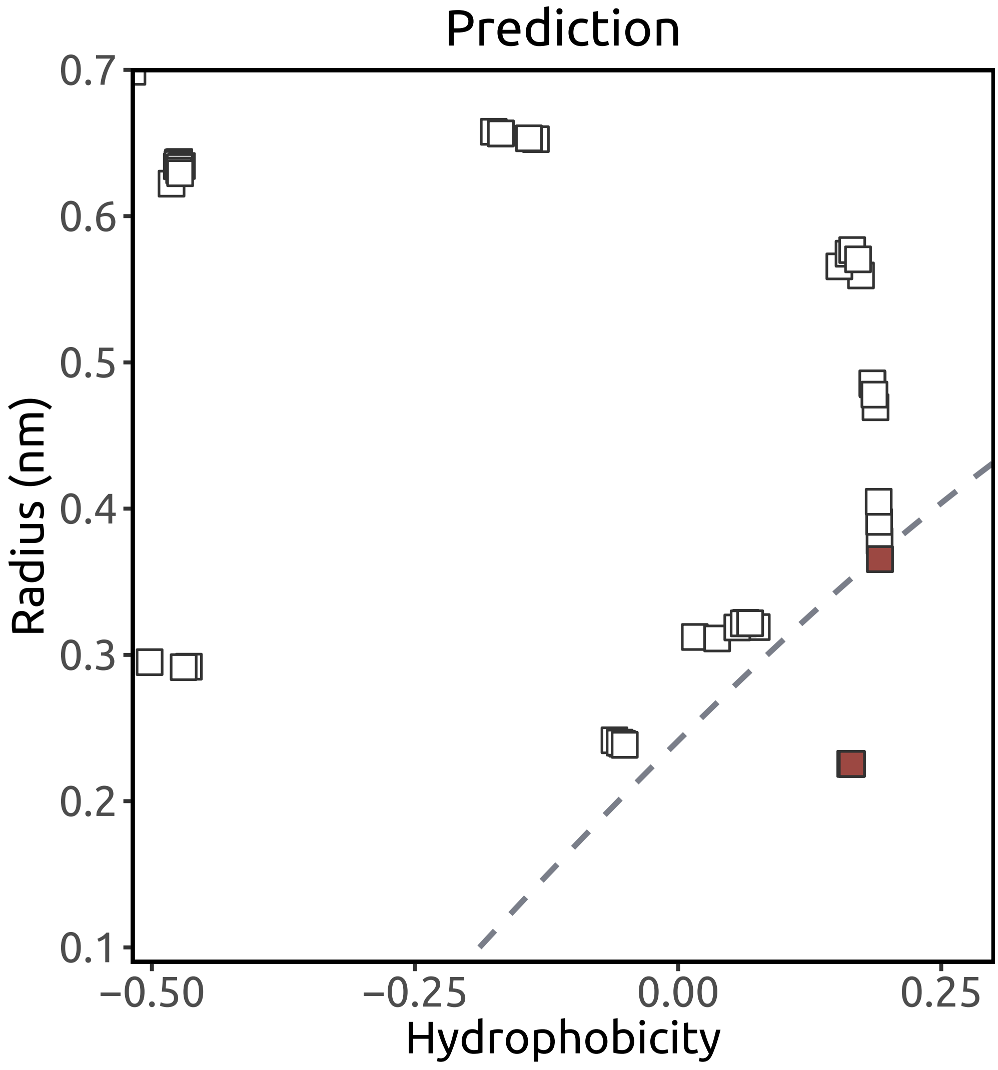


Heuristic score:

0.54 (n = 7)

Simulation result:

hydrated channel

# GLIC (PDB ID: 5L47)

*Gloeobacter violaceus*

X–ray (3.3 Å)

Fourati et al., 2017

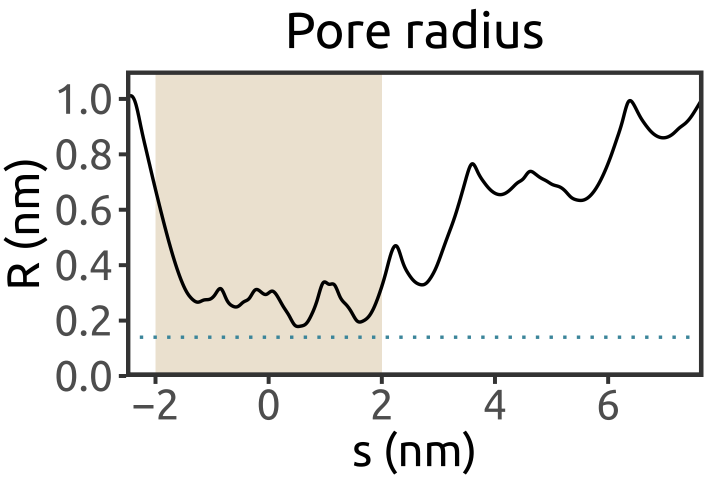


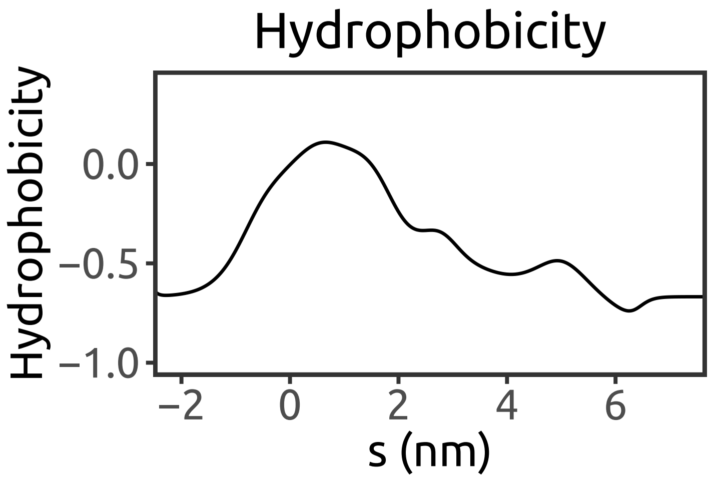


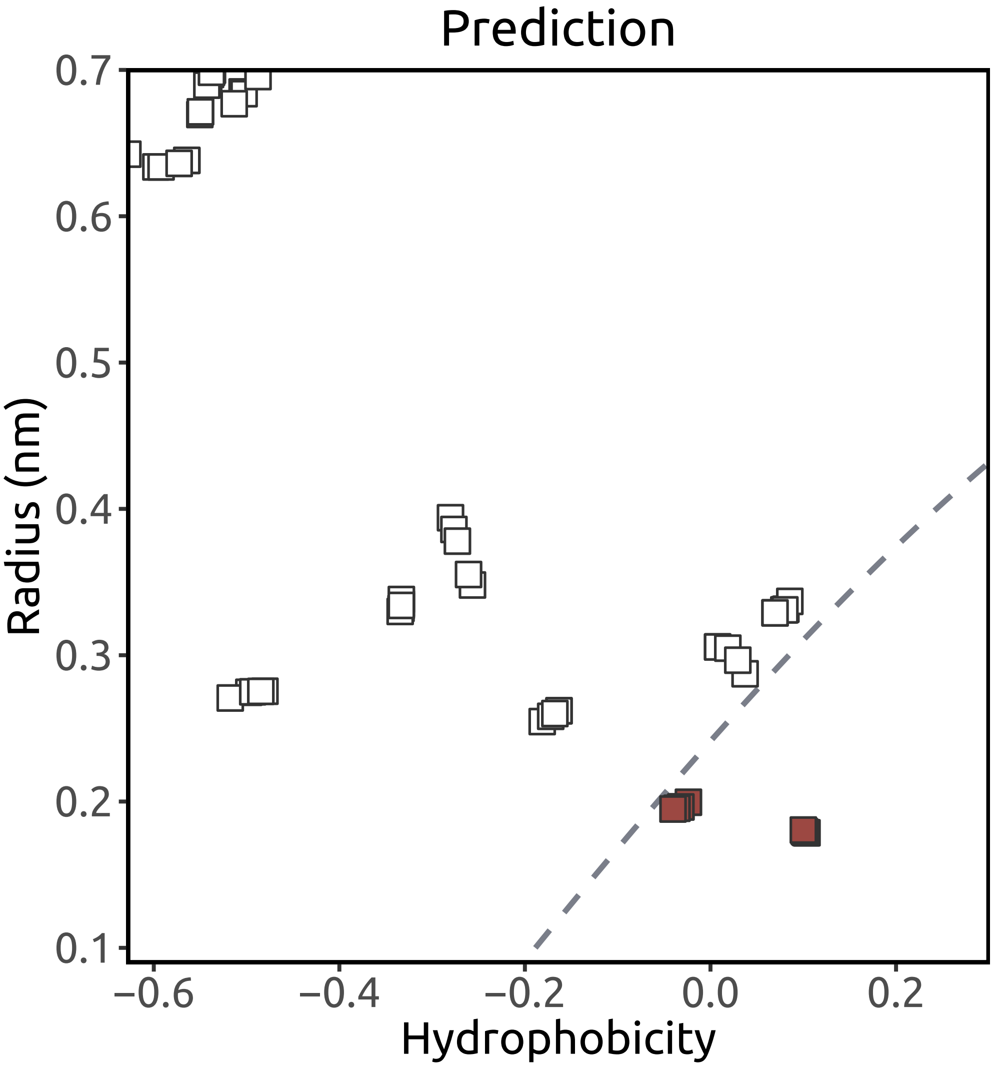


Heuristic score:

0.63 (n = 10)

Simulation result:

barrier to water

# GluCl (PDB ID: 3RHW)

*Caenorhabditis elegans*

X–ray (3.26 Å)

Pan et al., 2012

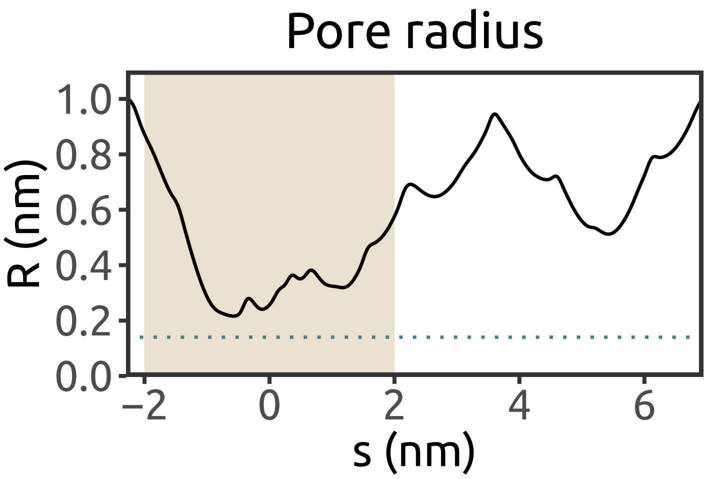


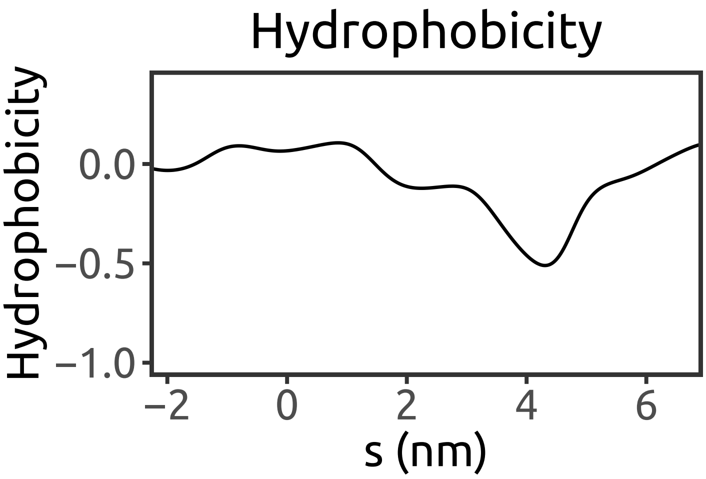


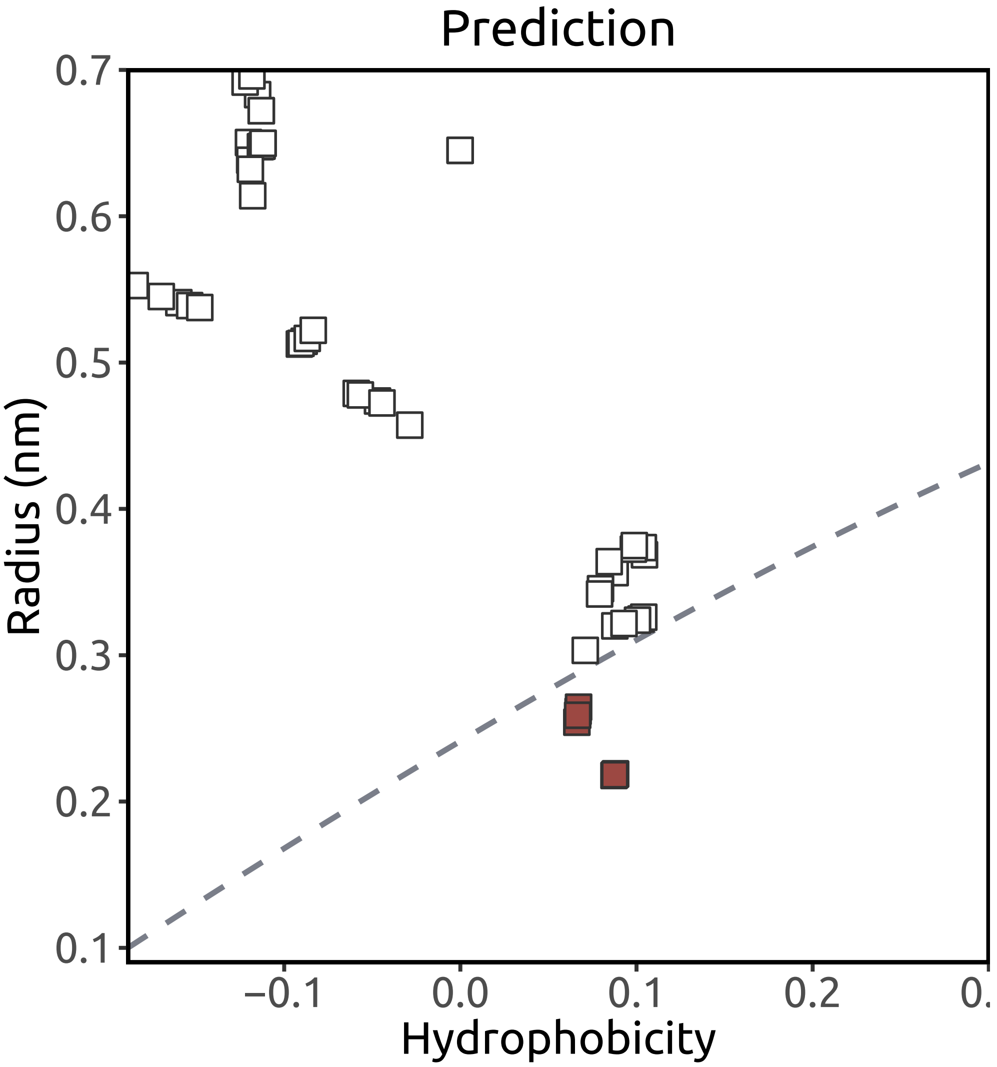


Heuristic score:

0.44 (n = 9)

Simulation result:

hydrated channel

# GluCl (PDB ID: 3RIF)

*Caenorhabditis elegans*

X–ray (3.35 Å)

Hibbs & Gounaux, 2011

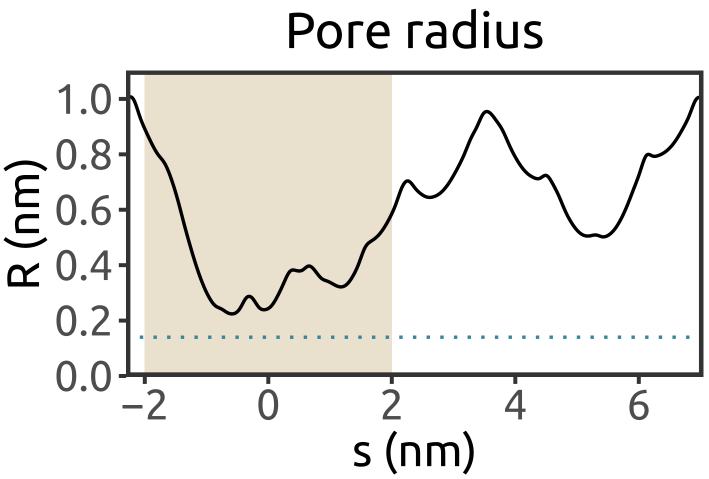


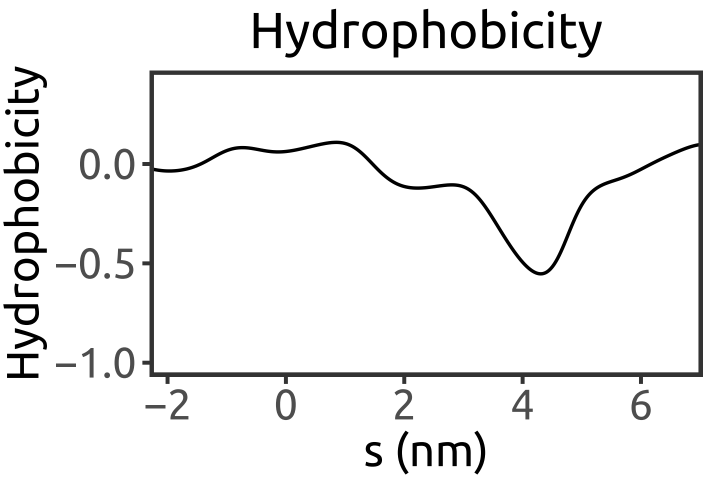


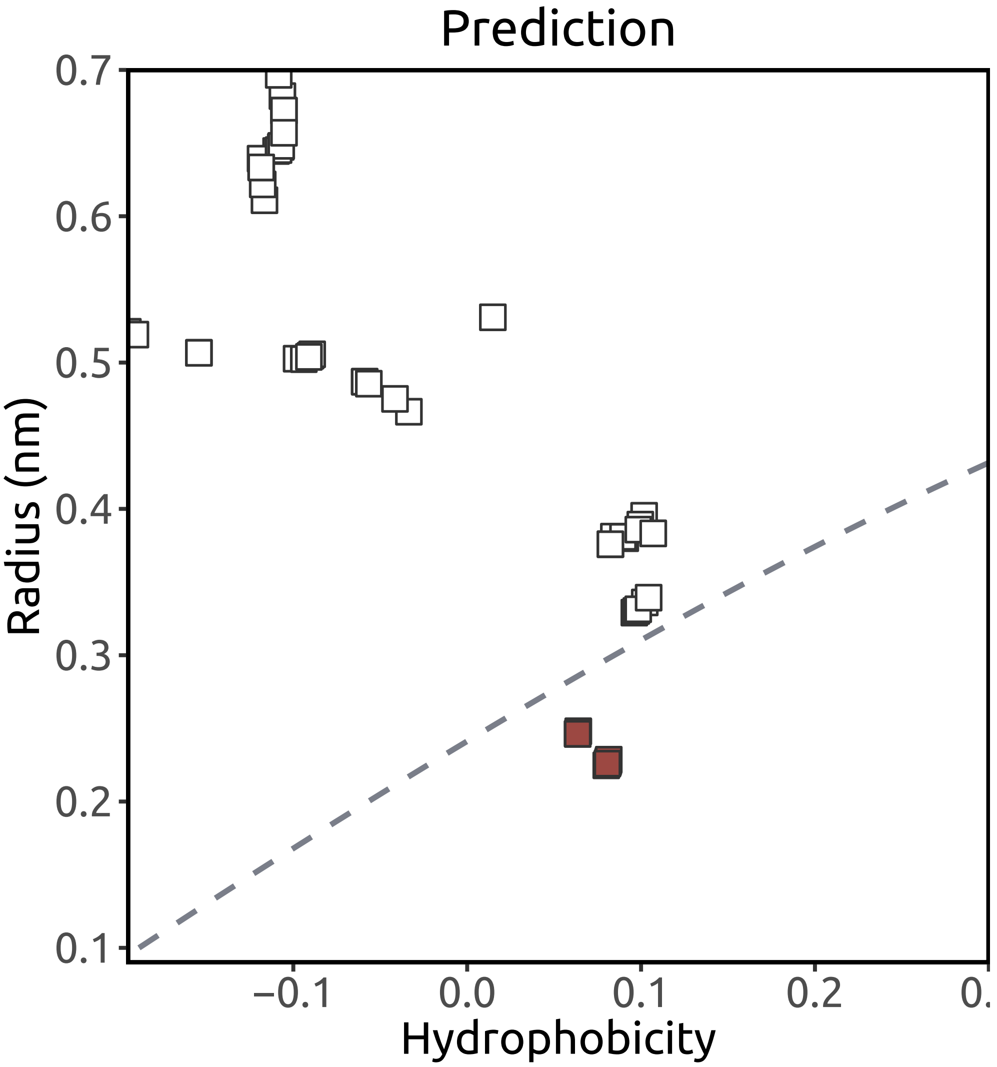


Heuristic score:

0.45 (n = 10)

Simulation result:

hydrated channel

# GluCl (PDB ID: 4TNV)

*Caenorhabditis elegans*

X–ray (3.6 Å)

Althoff et al., 2014

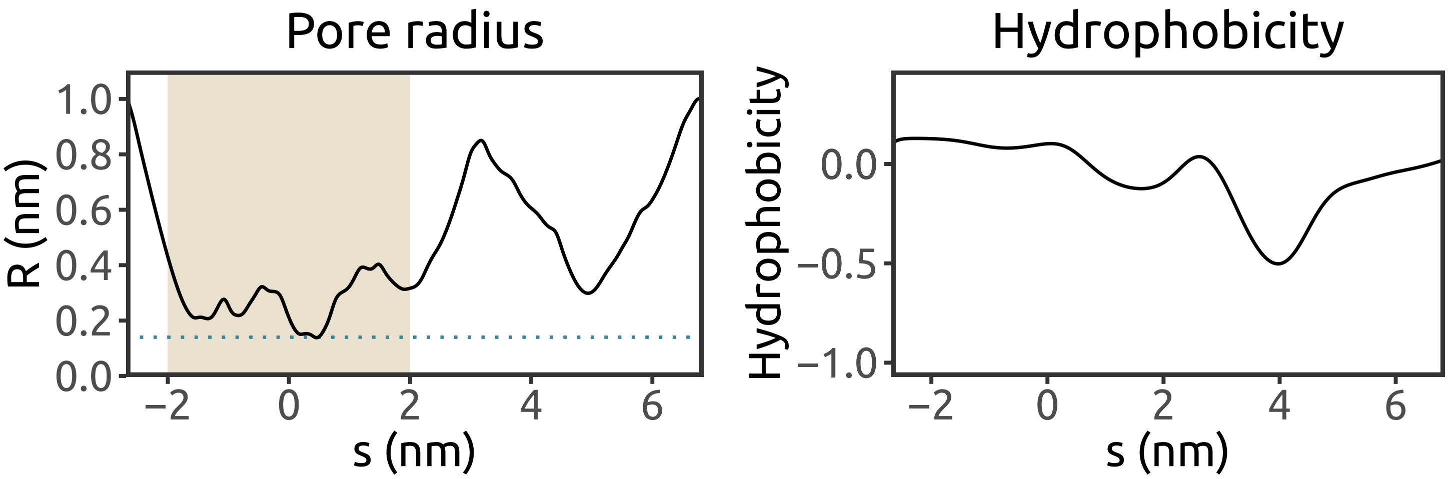


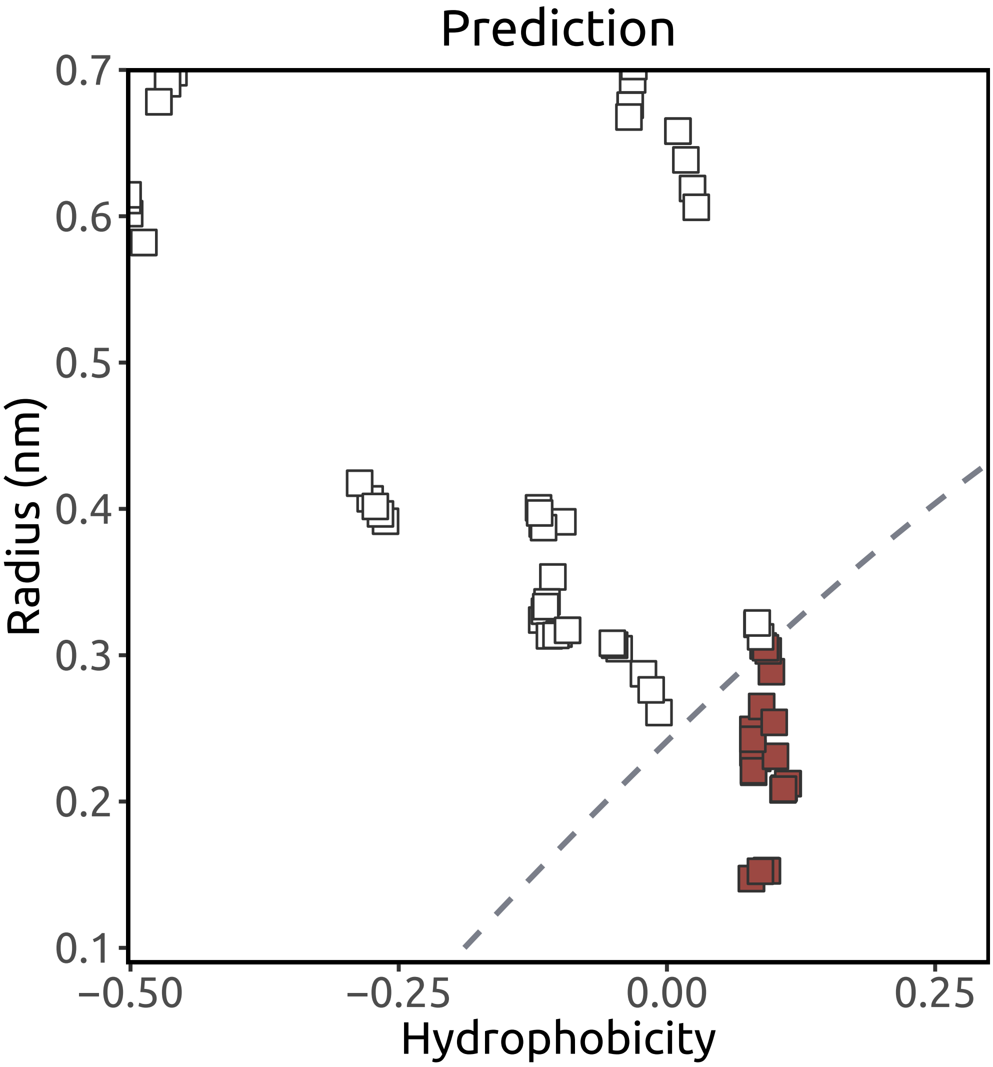


Heuristic score:

1.55 (n = 22)

Simulation result:

barrier to water

# GlyR (PDB ID: 3JAD)

*Danio rerio*

cryo–EM (3.9 Å)

Du et al., 2015

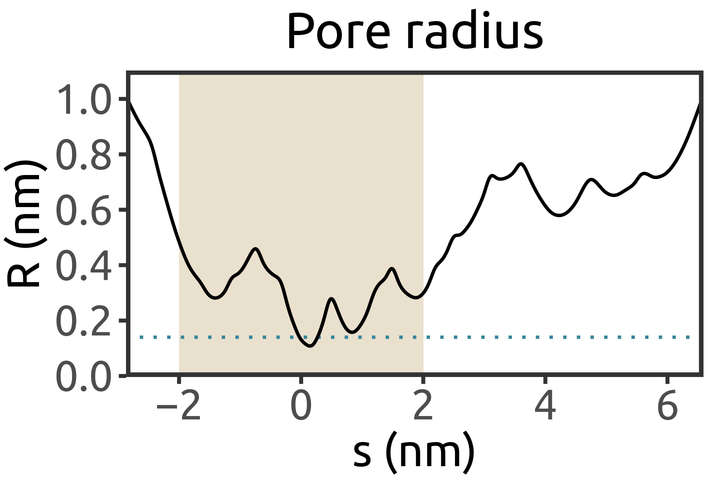


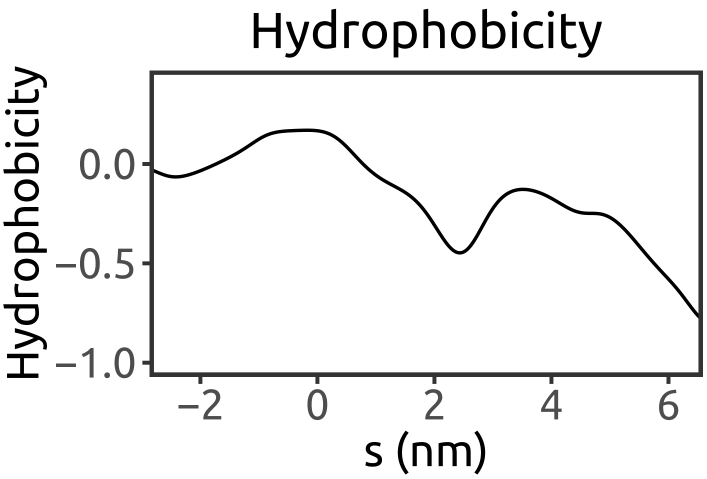


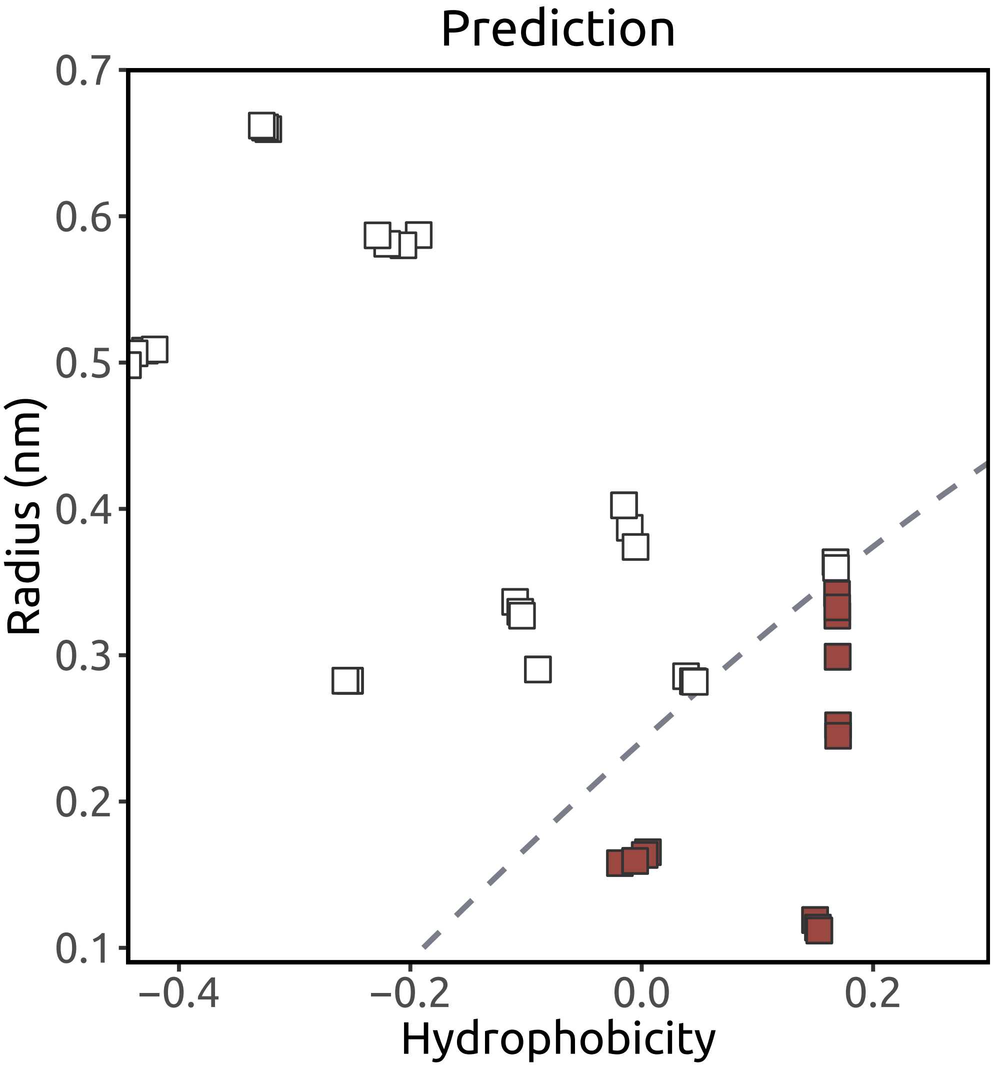


Heuristic score:

1.59 (n = 17)

Simulation result:

barrier to water

# GlyR (PDB ID: 3JAE)

*Danio rerio*

cryo−EM (3.9 Å)

Du et al., 2015

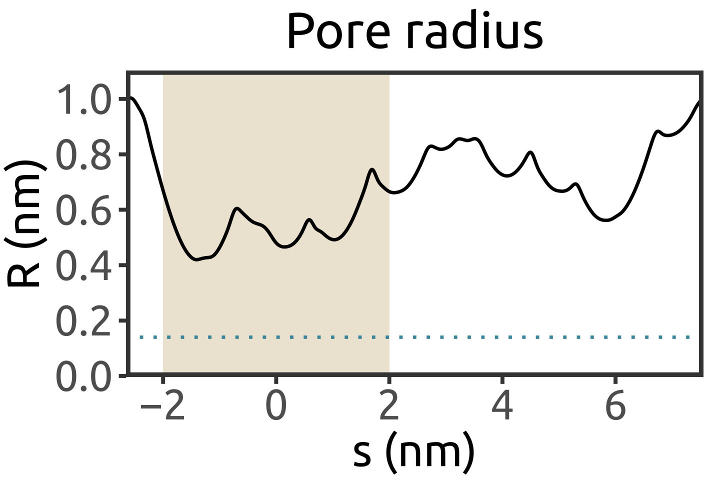


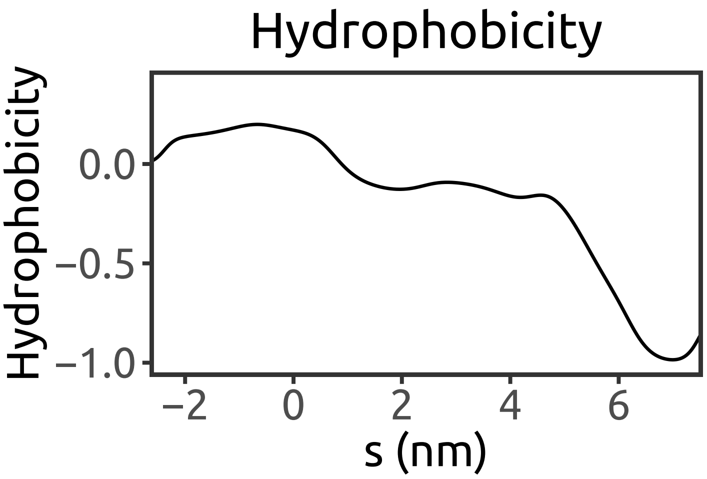


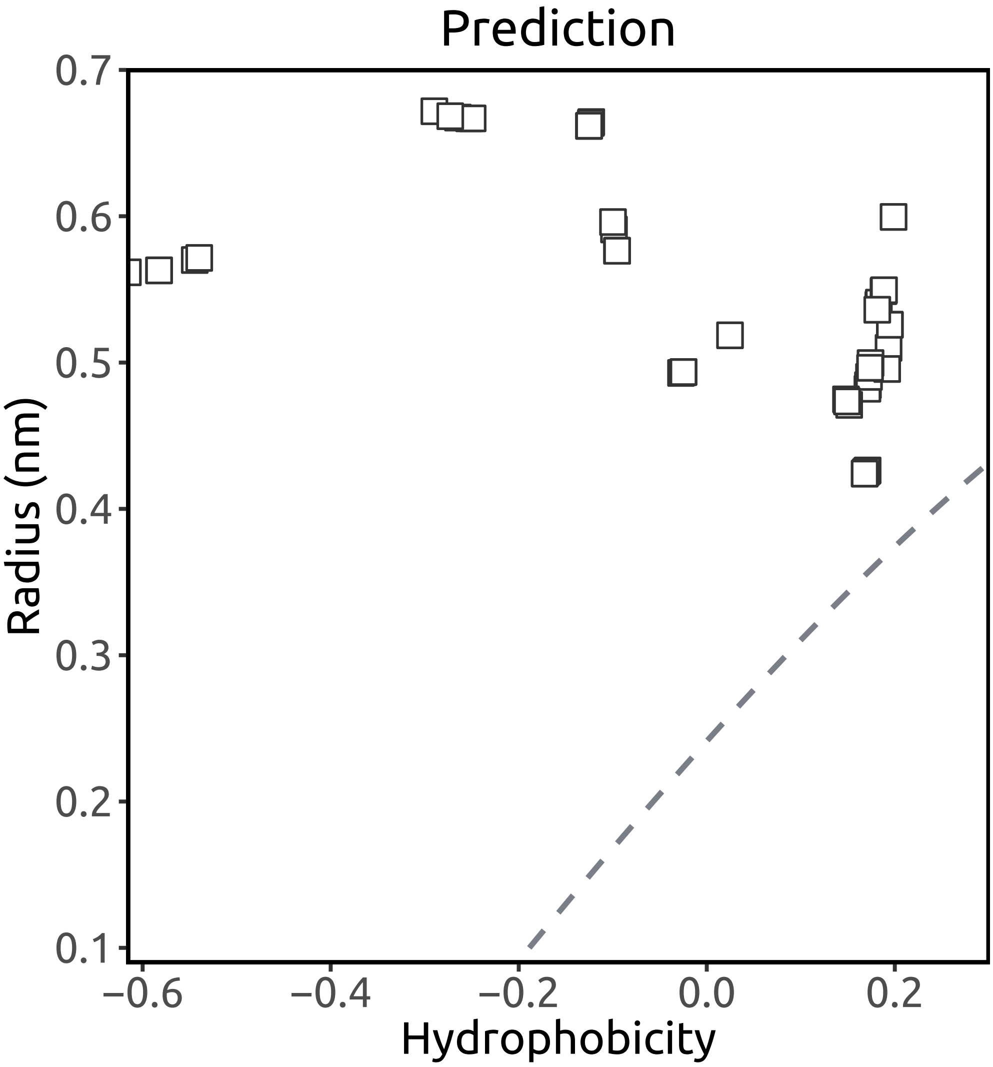


Heuristic score:

0 (n = 0)

Simulation result:

hydrated channel

# GlyR (PDB ID: 3JAF)

*Danio rerio*
cryo–EM (3.8 Å)

Du et al., 2015

## Pore radius

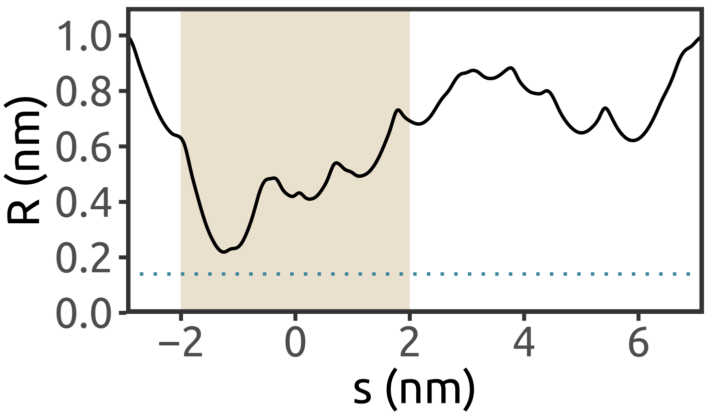


## Hydrophobicity

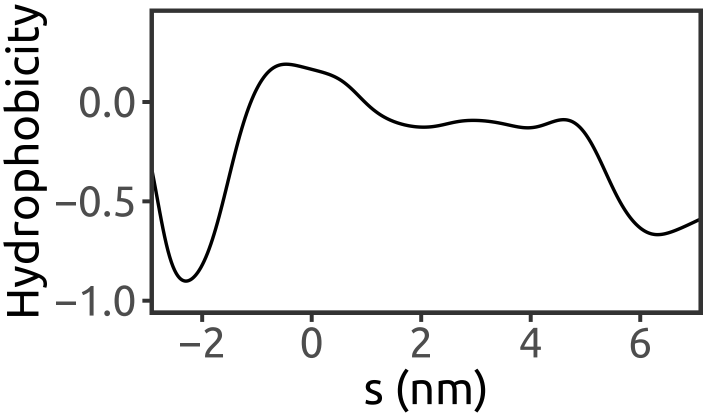


## Prediction

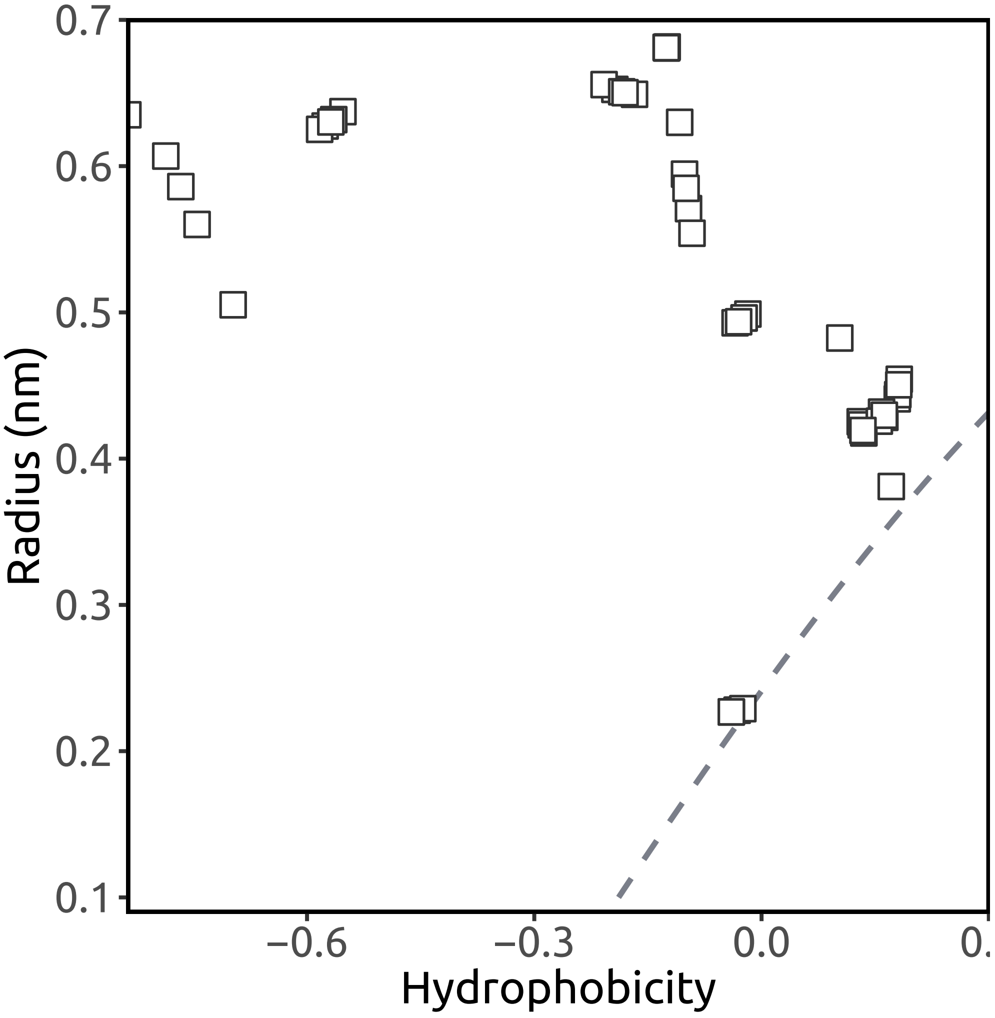


Heuristic score:
0 (n = 0)

Simulation result:
hydrated channel

# GlyR (PDB ID: 5CFB)

*Homo sapiens*

X–ray (3.04 Å)

Huang et al., 2015

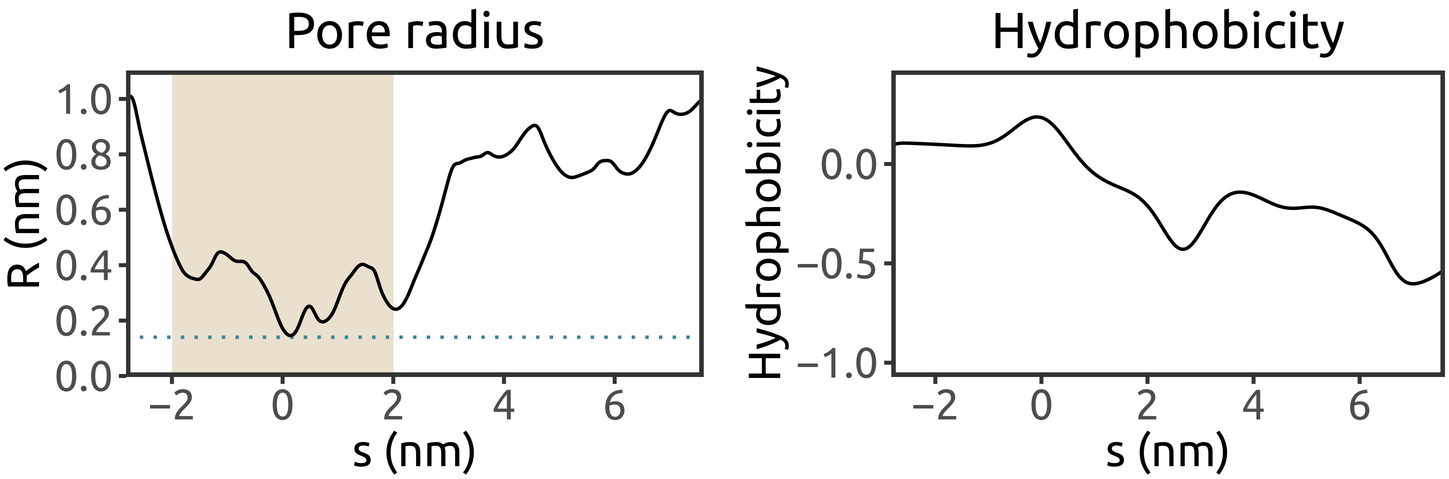


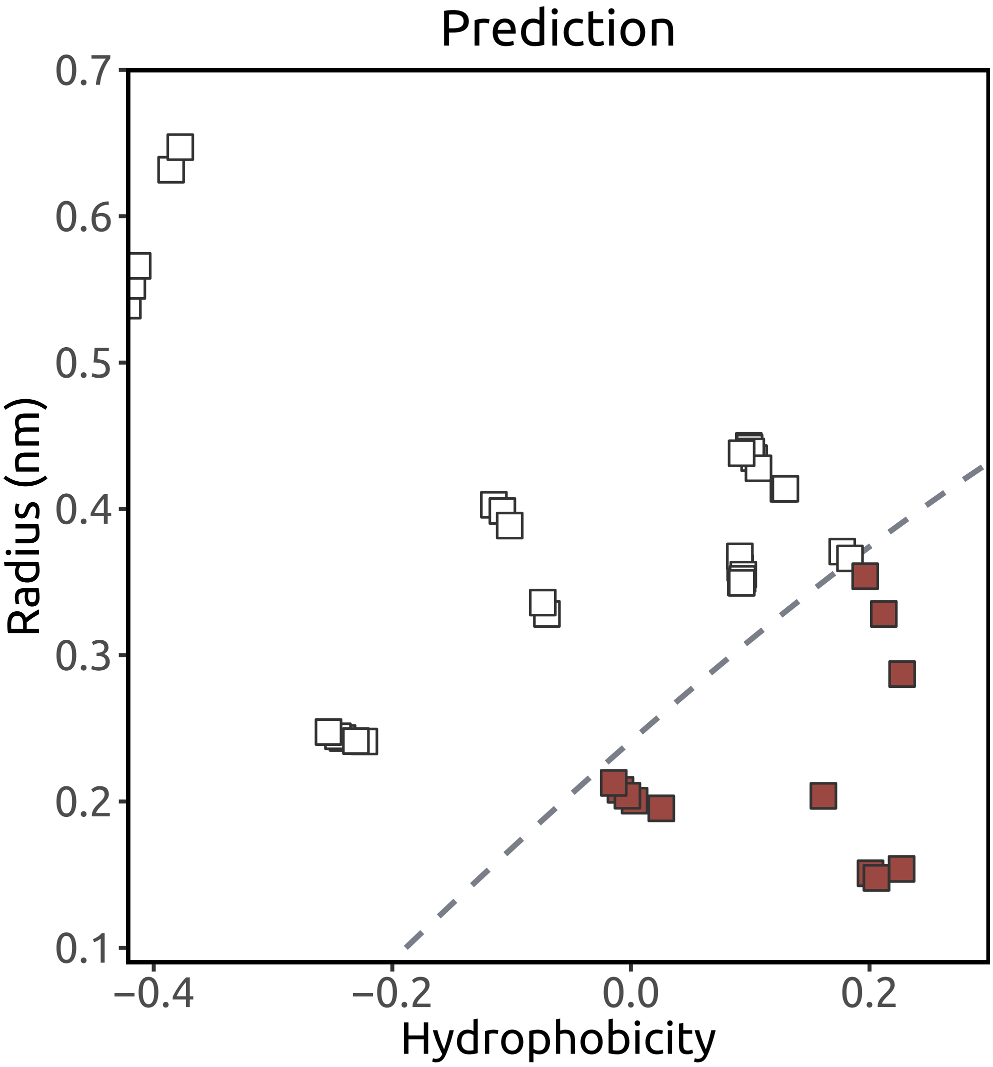


Heuristic score:

1.2 (n = 13)

Simulation result:

barrier to water

# GlyR (PDB ID: 5VDH)

*Homo sapiens*
X–ray (2.85 Å)

Huang et al., 2017

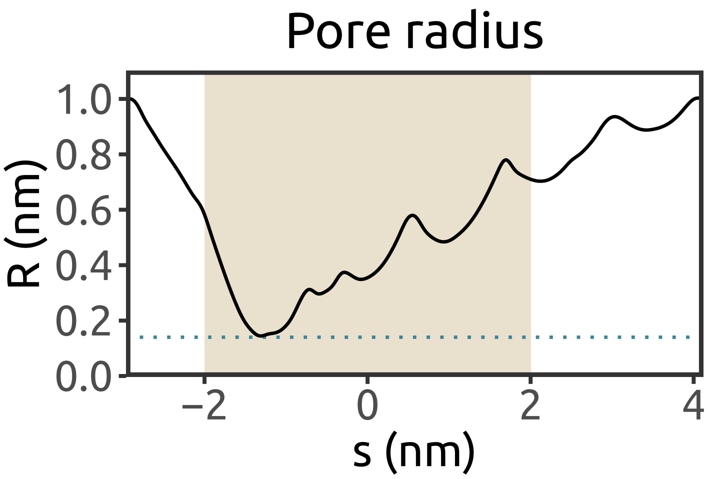


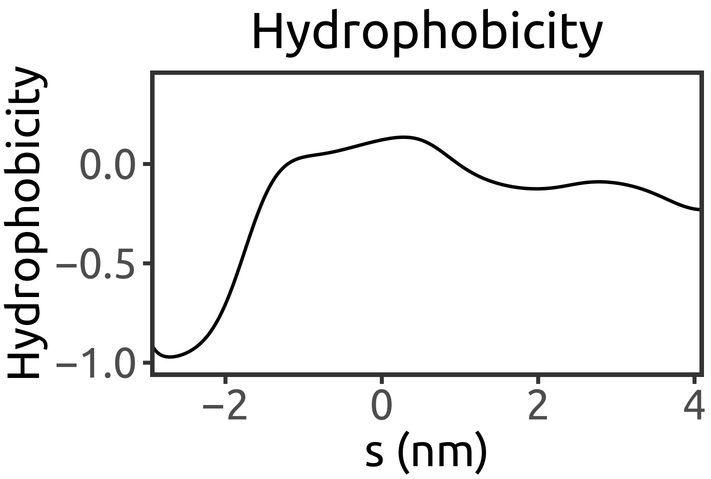


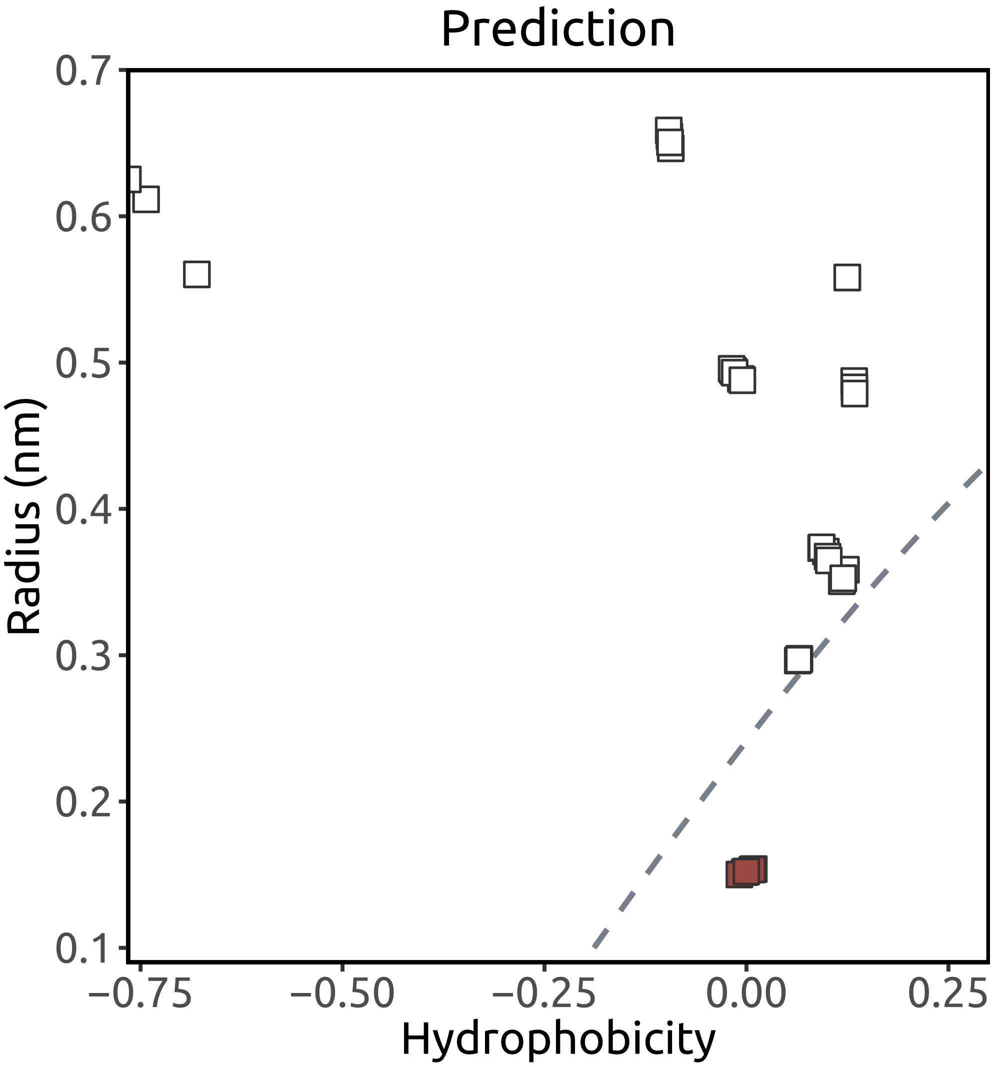


Heuristic score:
0.36 (n = 5)

Simulation result:
barrier to water

# nAChR (PDB ID: 1OED)

*Torpedo marmorata*
EM (4 Å)

Miyazawa et al., 2003

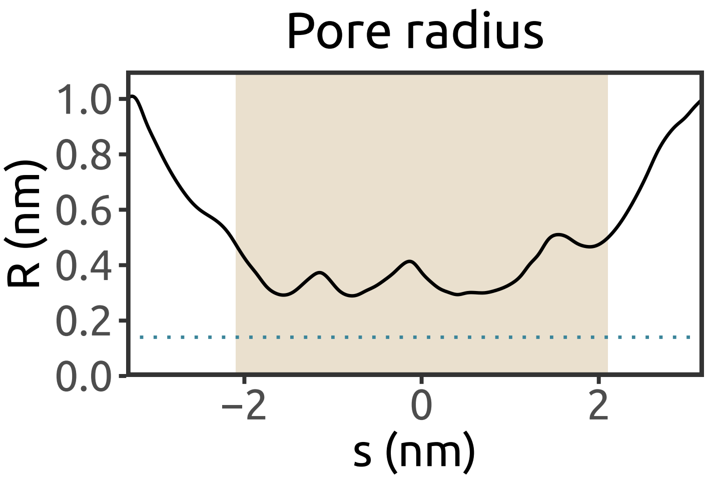


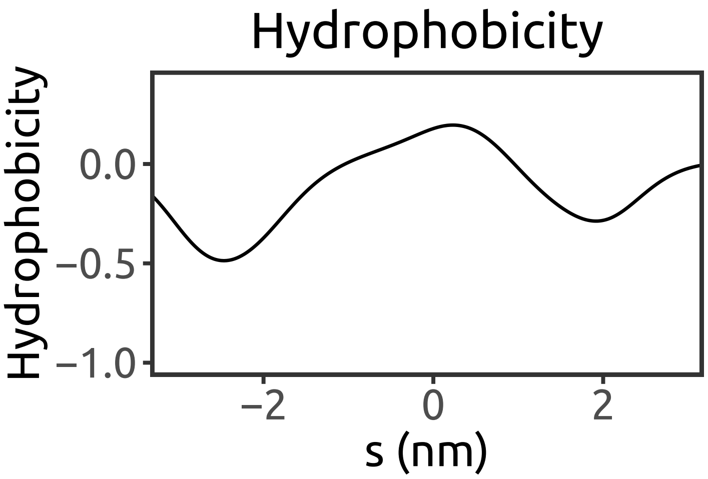


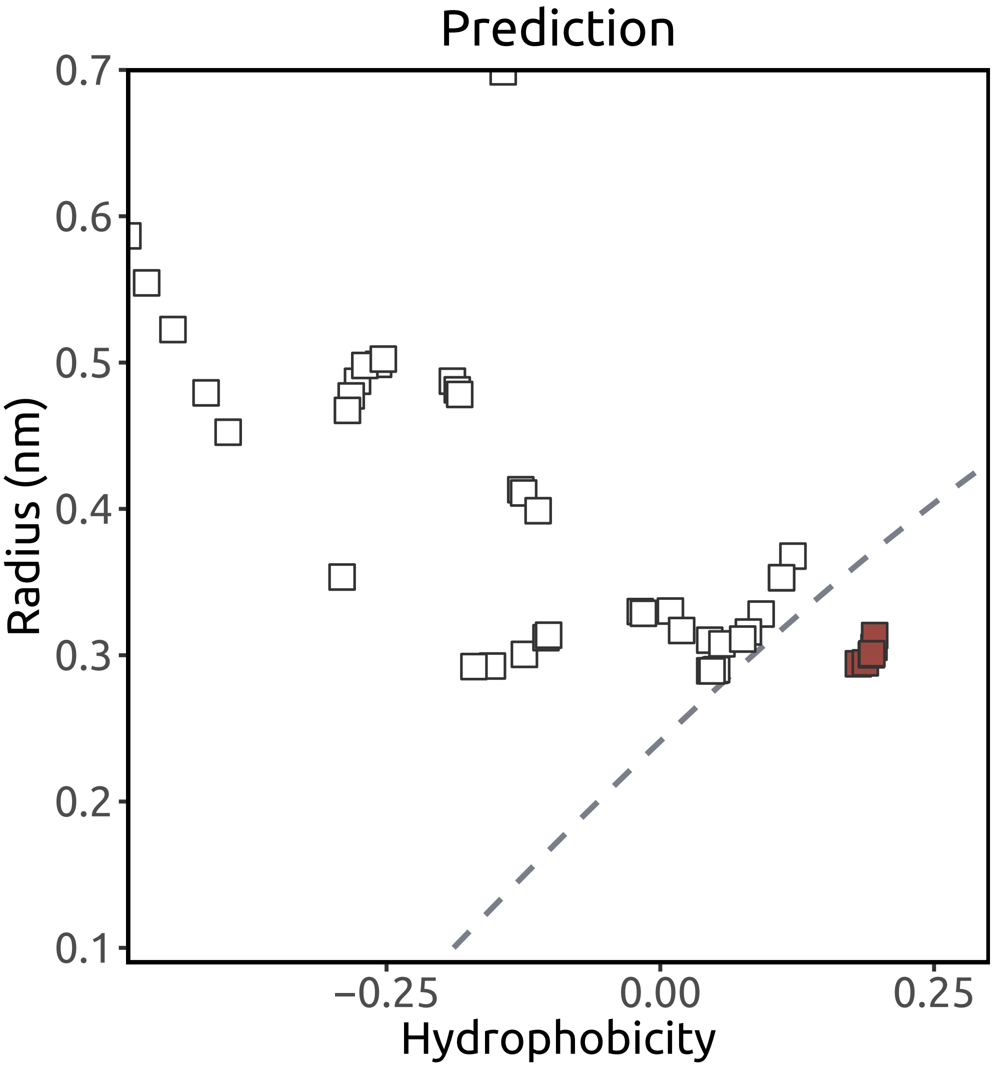


Heuristic score:
0.34 (n = 6)

Simulation result:
barrier to water

# nAChR (PDB ID: 2BG9)

*Torpedo marmorata*

EM (4 Å)

Unwin, 2005

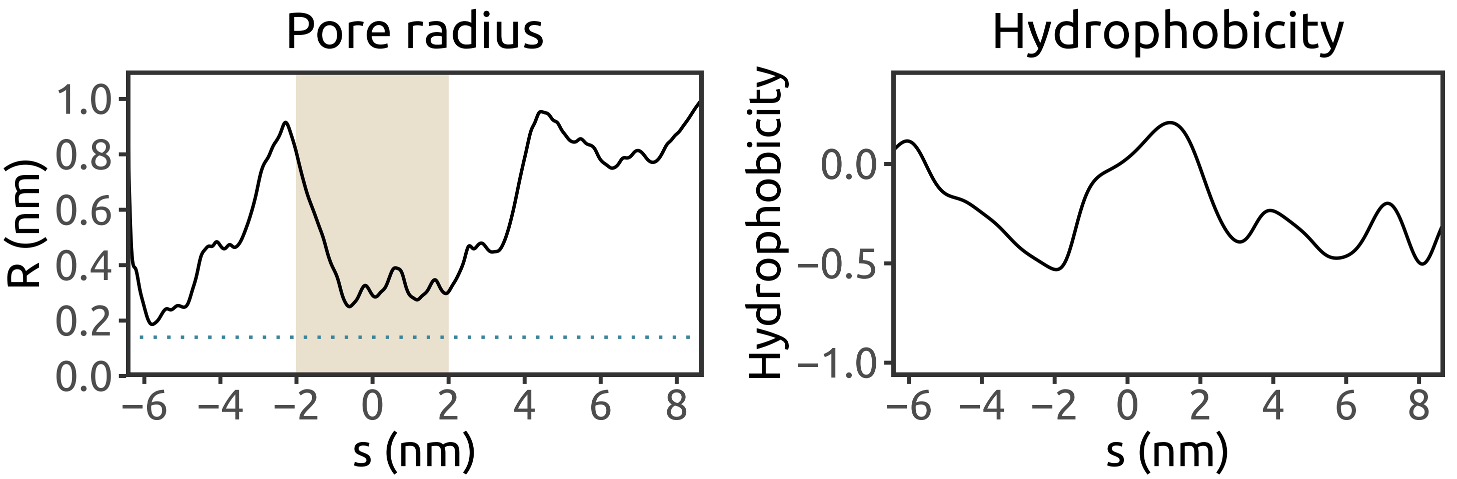


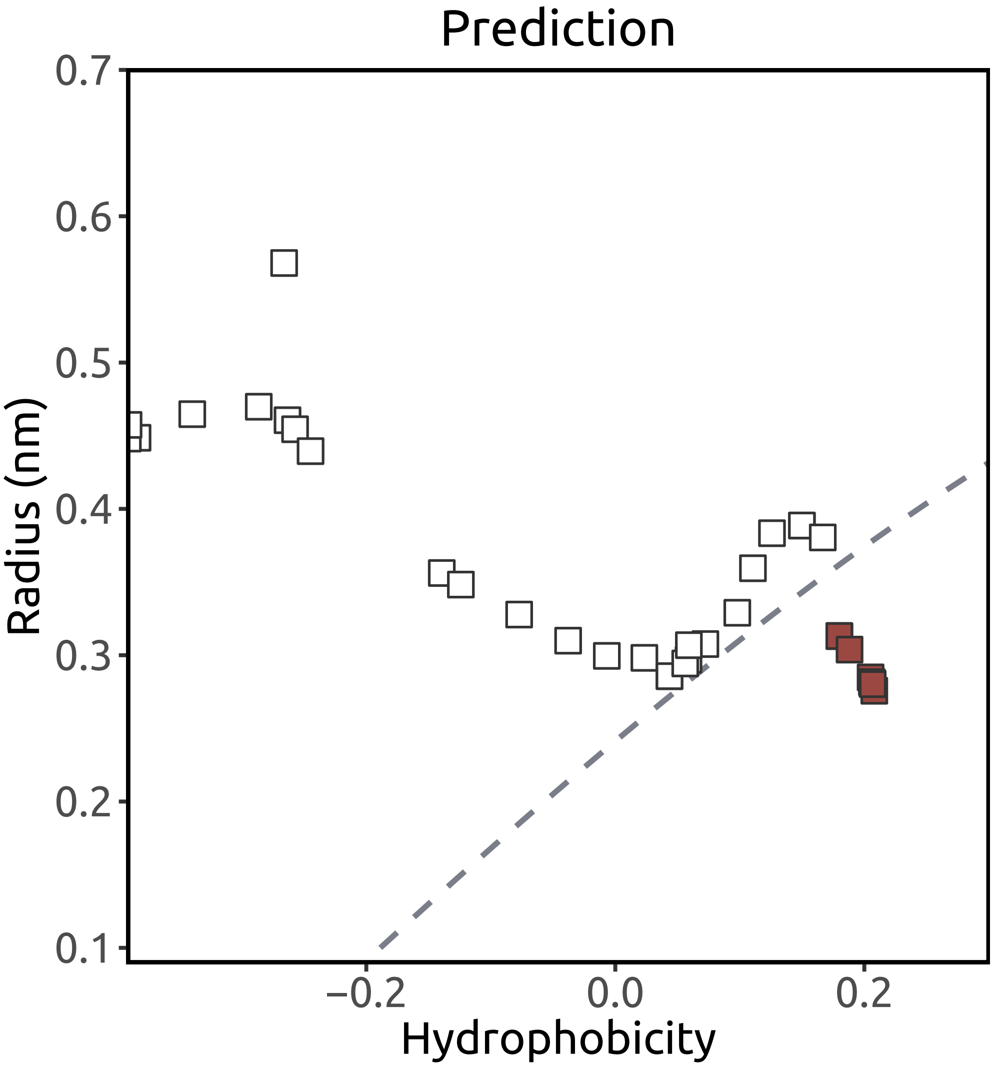


Heuristic score:

0.59 (n = 9)

Simulation result:

barrier to water

# nAChR (PDB ID: 5KXI)

*Homo sapiens*
X−ray (3.94 Å)

Morales−Perez et al., 2016

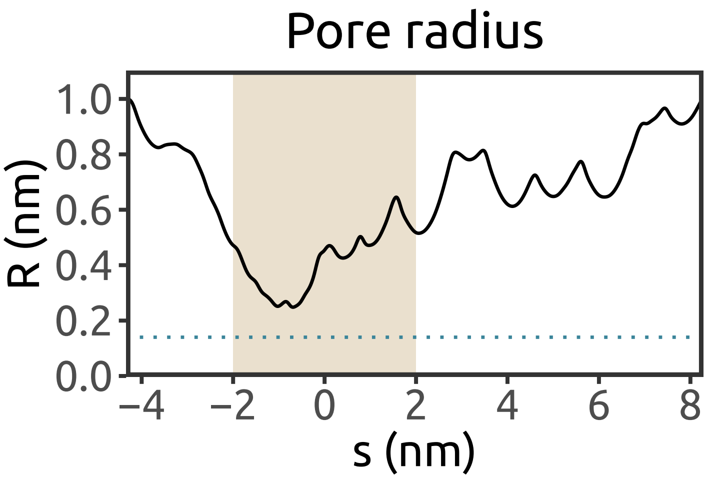


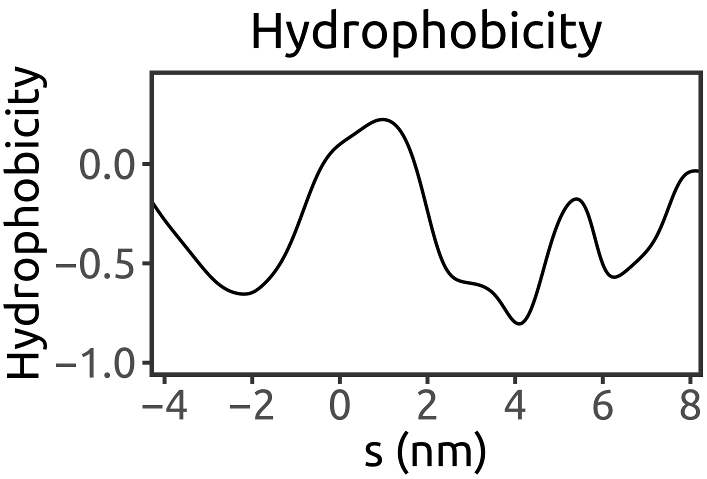


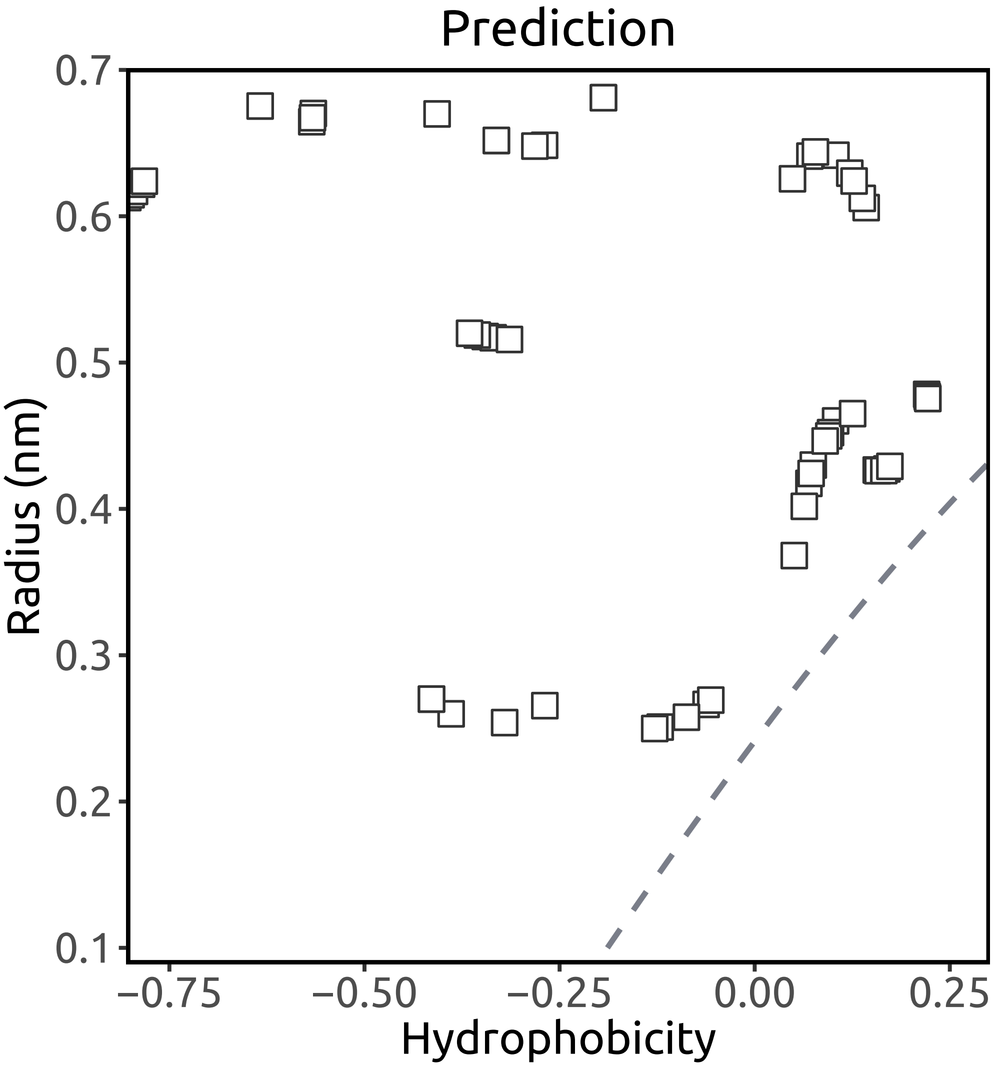


Heuristic score:
0 (n = 0)

Simulation result:
hydrated channel

# GluA (PDB ID: 5WEK)

*Rattus norvegicus*
cryo−EM (4.6 Å)

Twomey et al., 2017

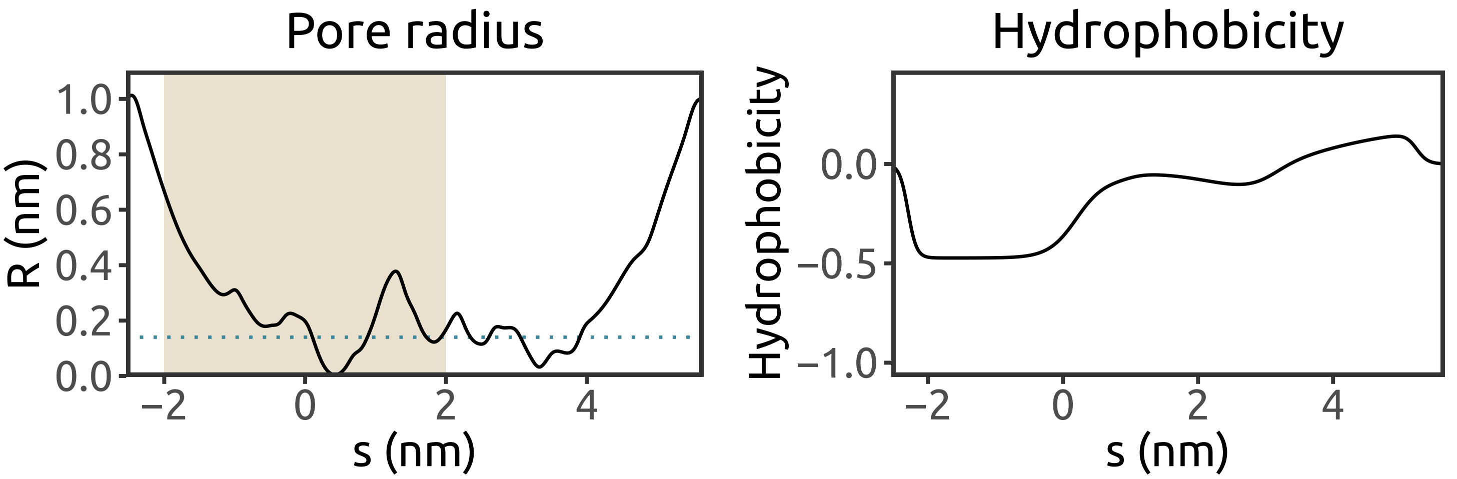


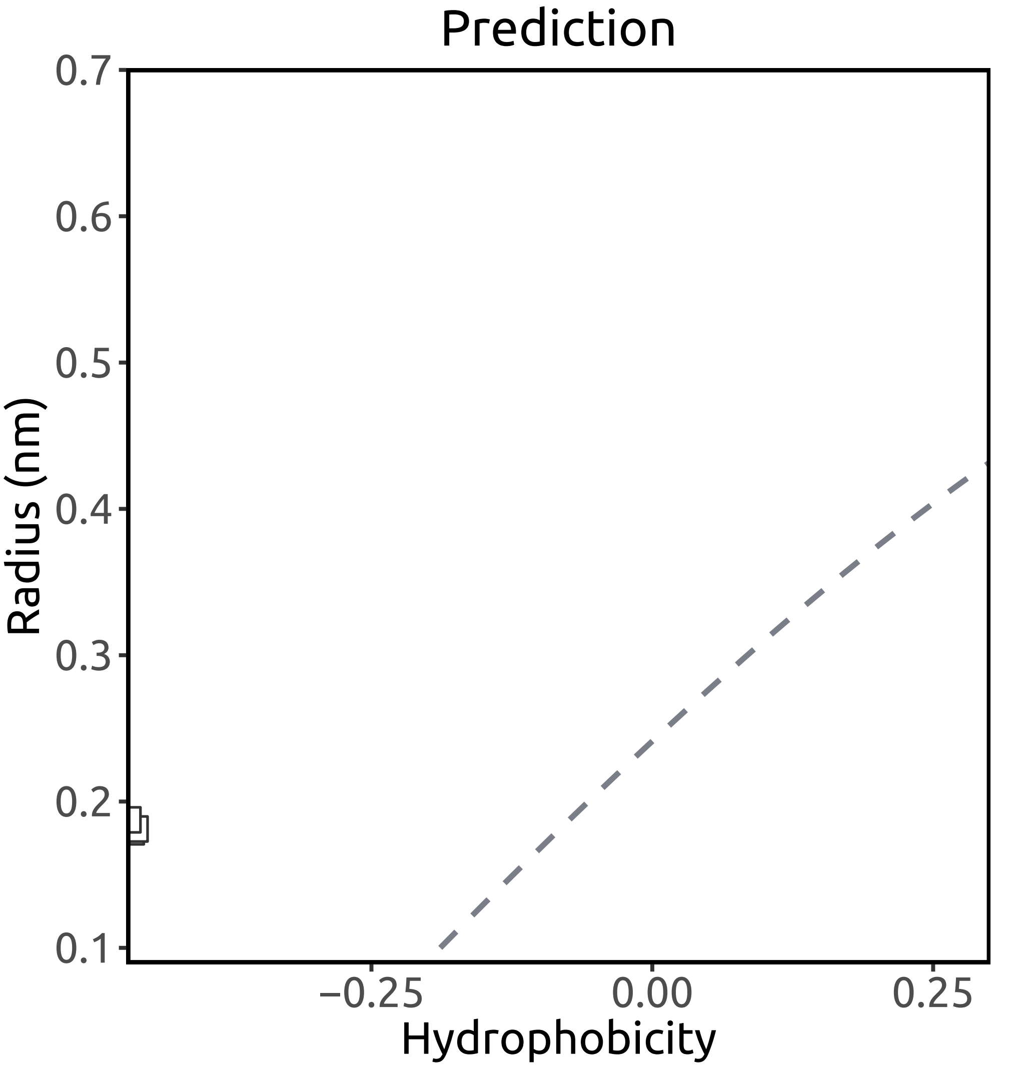


Heuristic score:
0.09 (n = 1)

Simulation result:
barrier to water

# GluA (PDB ID: 5WEL)

*Rattus norvegicus*
cryo−EM (4.4 Å)

Twomey et al., 2017

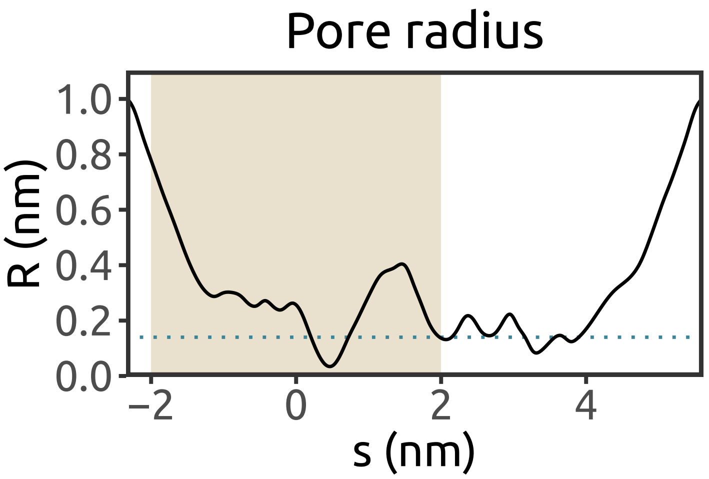


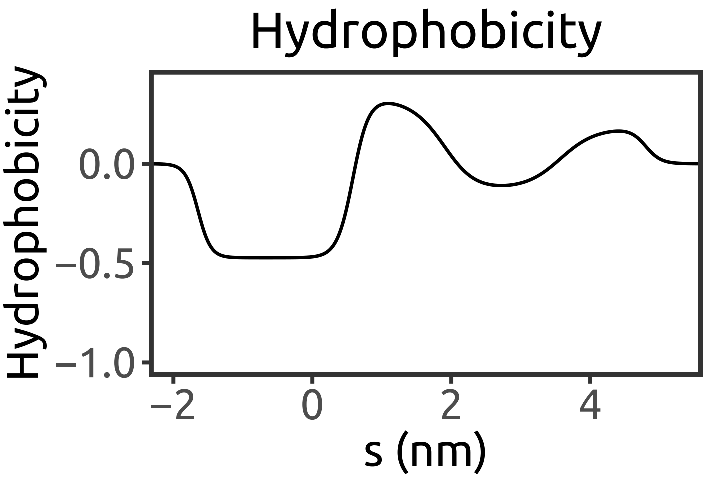


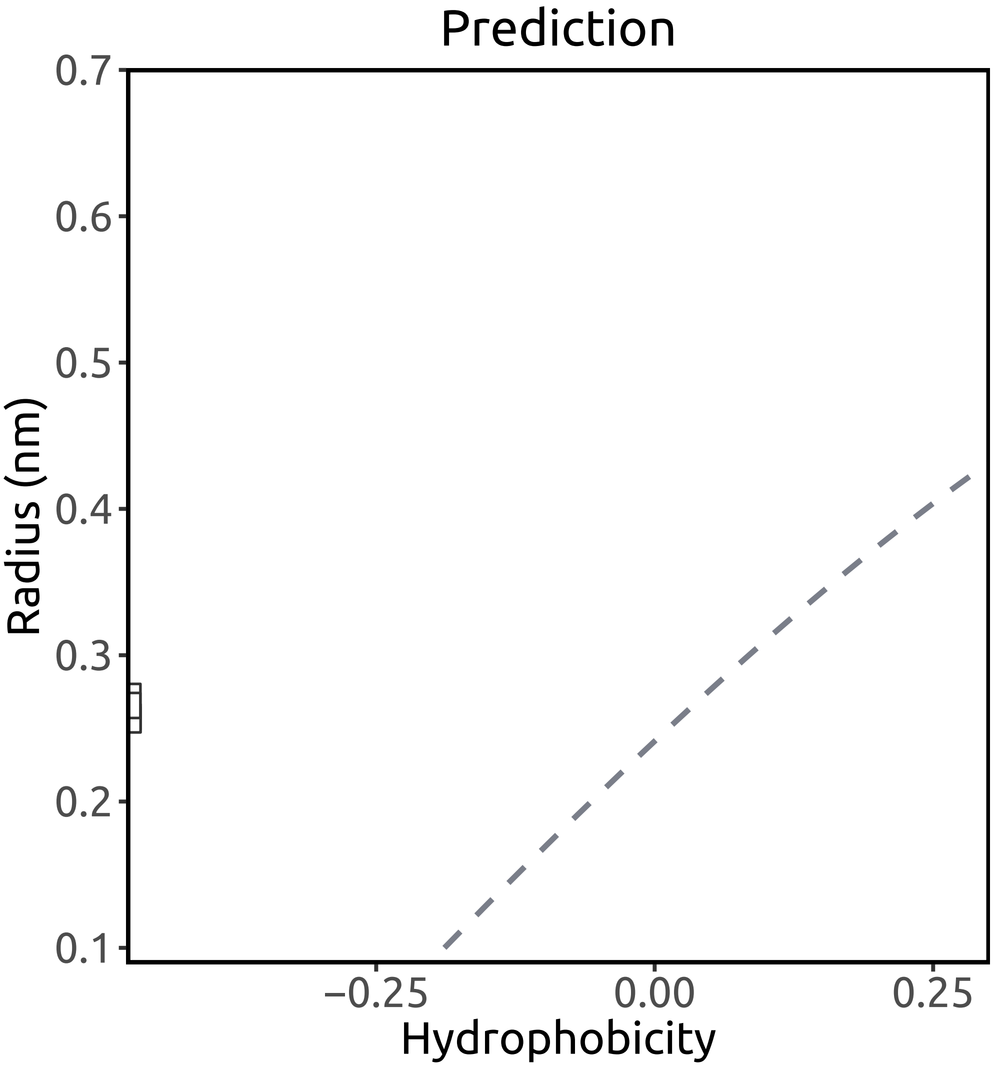


Heuristic score:

0 (n = 0)

Simulation result:

barrier to water

# GluA (PDB ID: 5WEO)

*Rattus norvegicus*
cryo−EM (4.2 Å)

Twomey et al., 2017

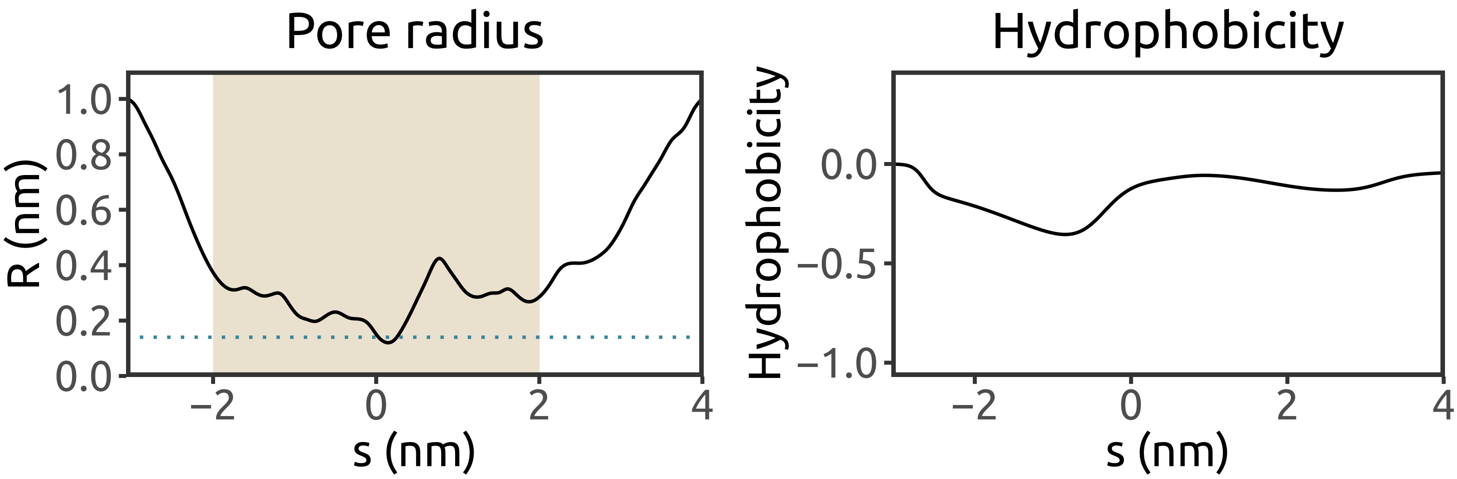


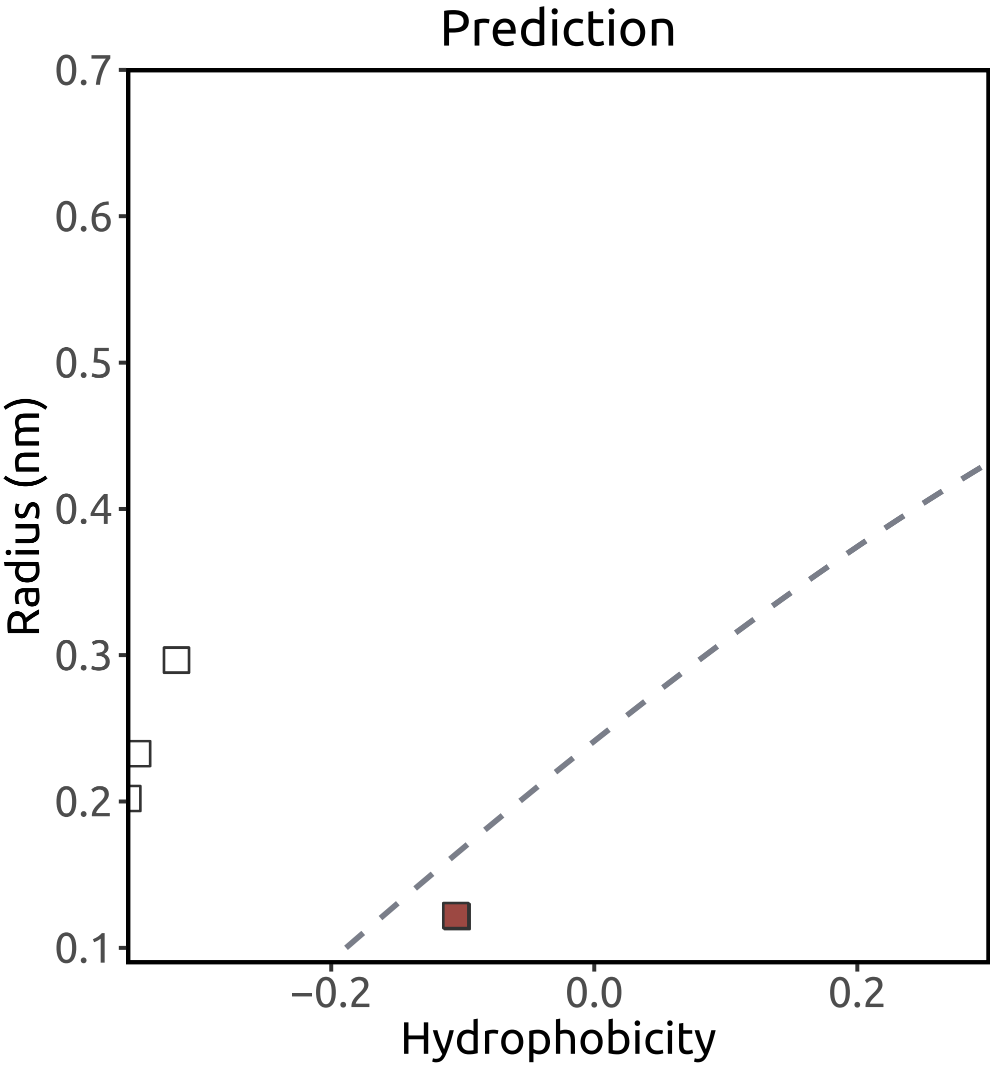


Heuristic score:
0.07 (n = 2)

Simulation result:
hydrated channel

# GluN (PDB ID: 5UOW)

*Xenopus laevis*
cryo–EM (4.5 Å)

Lü et al., 2017

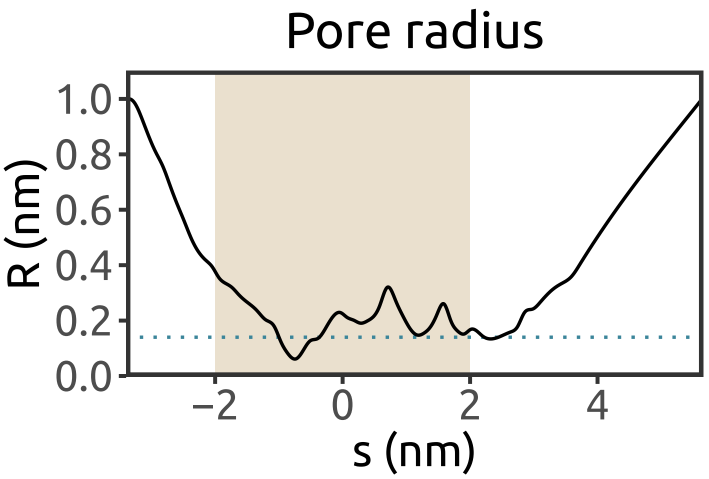


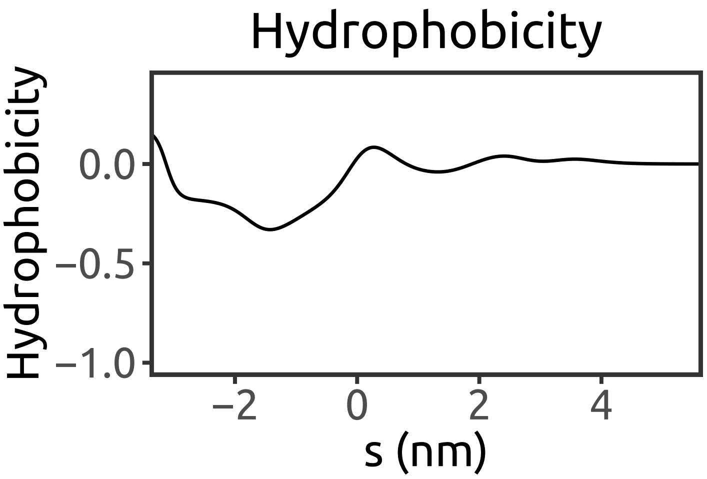


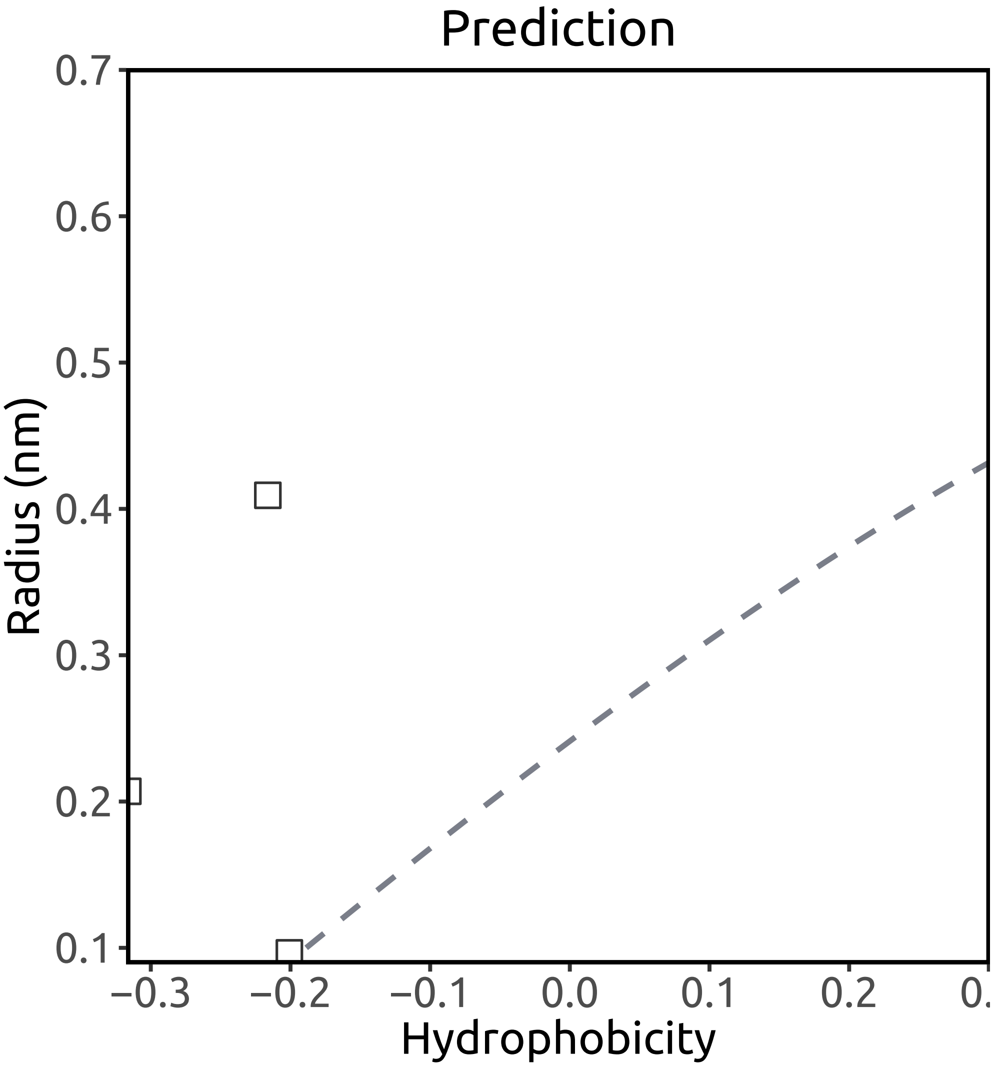


Heuristic score:
0 (n = 0)

Simulation result:
hydrated channel

# P2X3 (PDB ID: 5SVJ)

*Homo sapiens*
X–ray (2.98 Å)

Mansoor et al., 2016

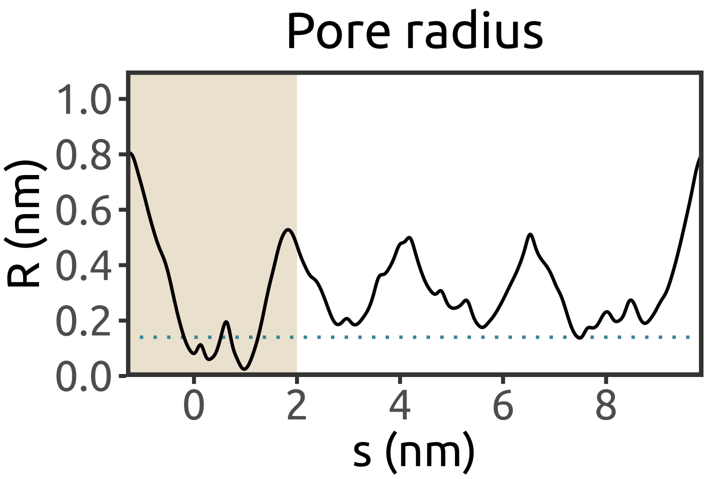


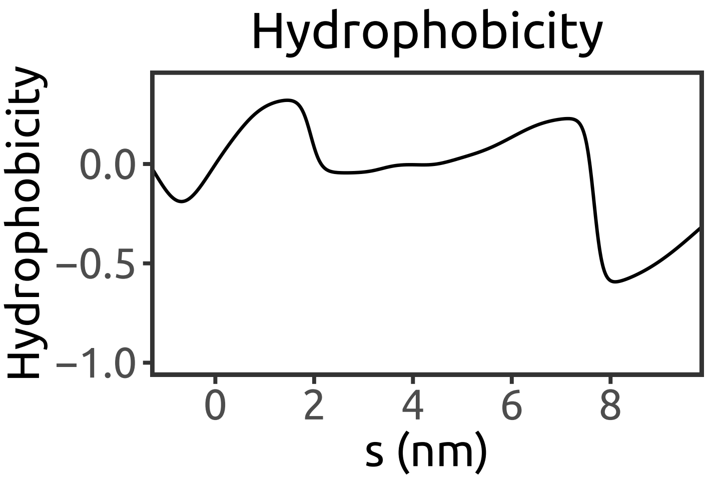


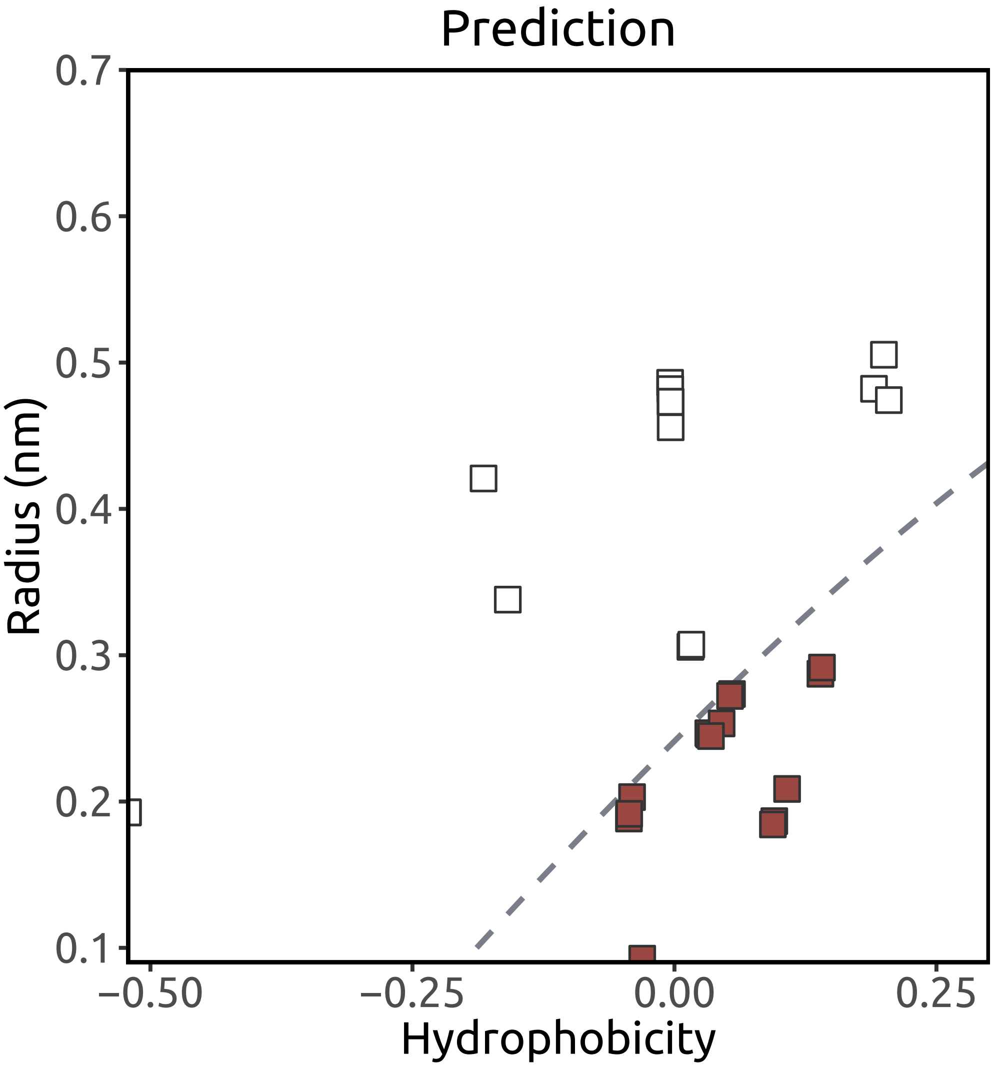


Heuristic score:
2.49 (n = 23)

Simulation result:
barrier to water

# P2X3 (PDB ID: 5SVK)

*Homo sapiens*

X−ray (2.77 Å)

Mansoor et al., 2016

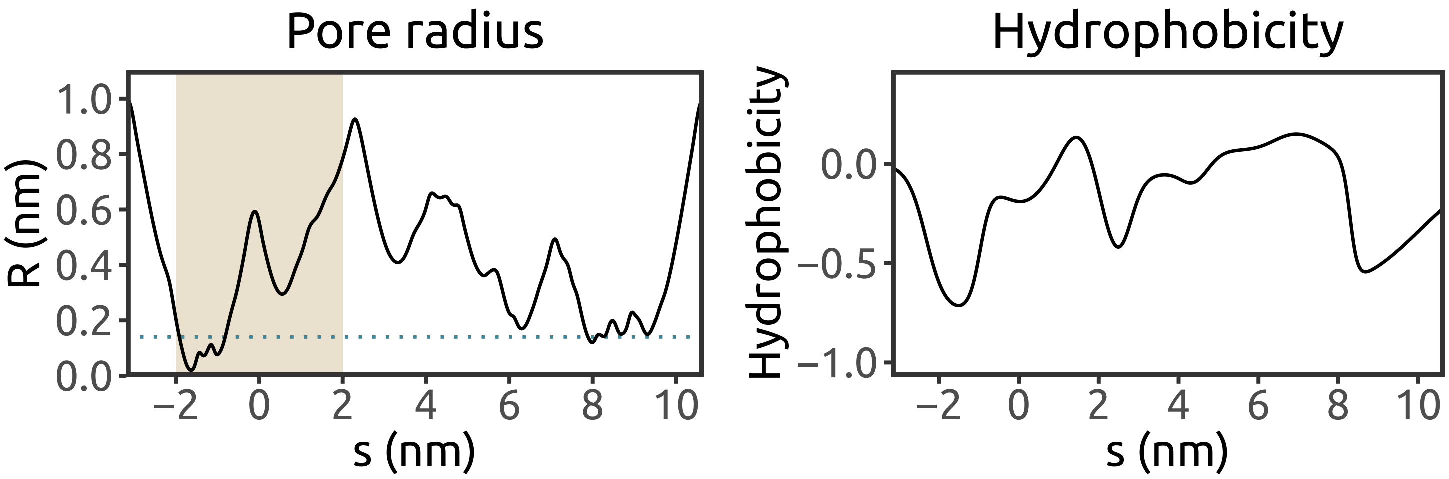


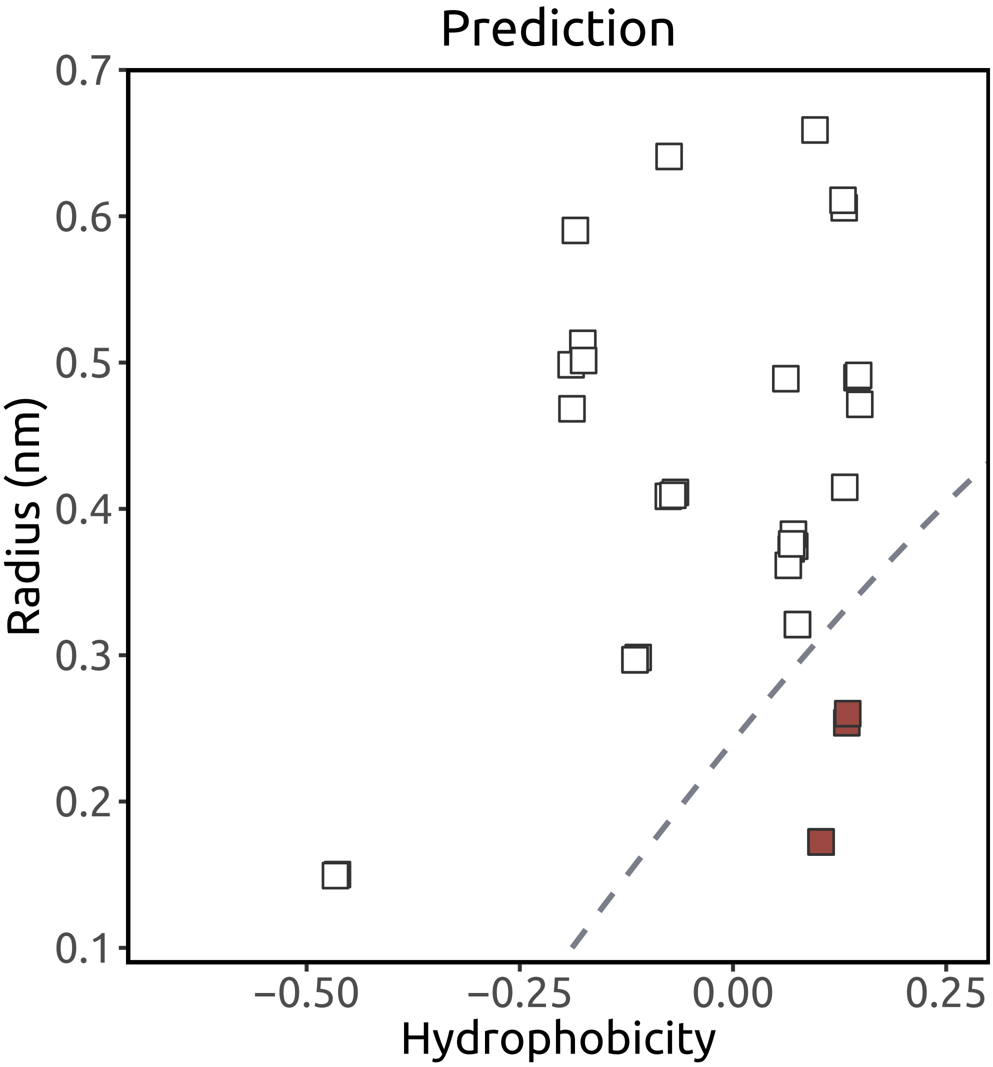


Heuristic score:

0.47 (n = 5)

Simulation result:

barrier to water

# P2X3 (PDB ID: 5SVL)

*Homo sapiens*
X–ray (2.9 Å)

Mansoor et al., 2016

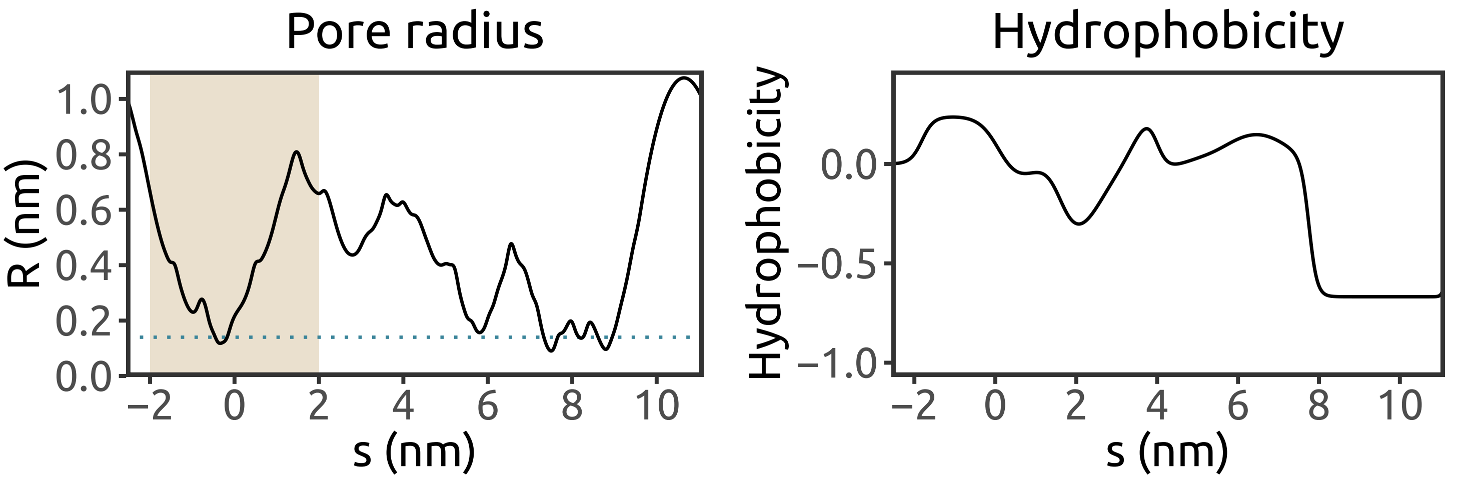


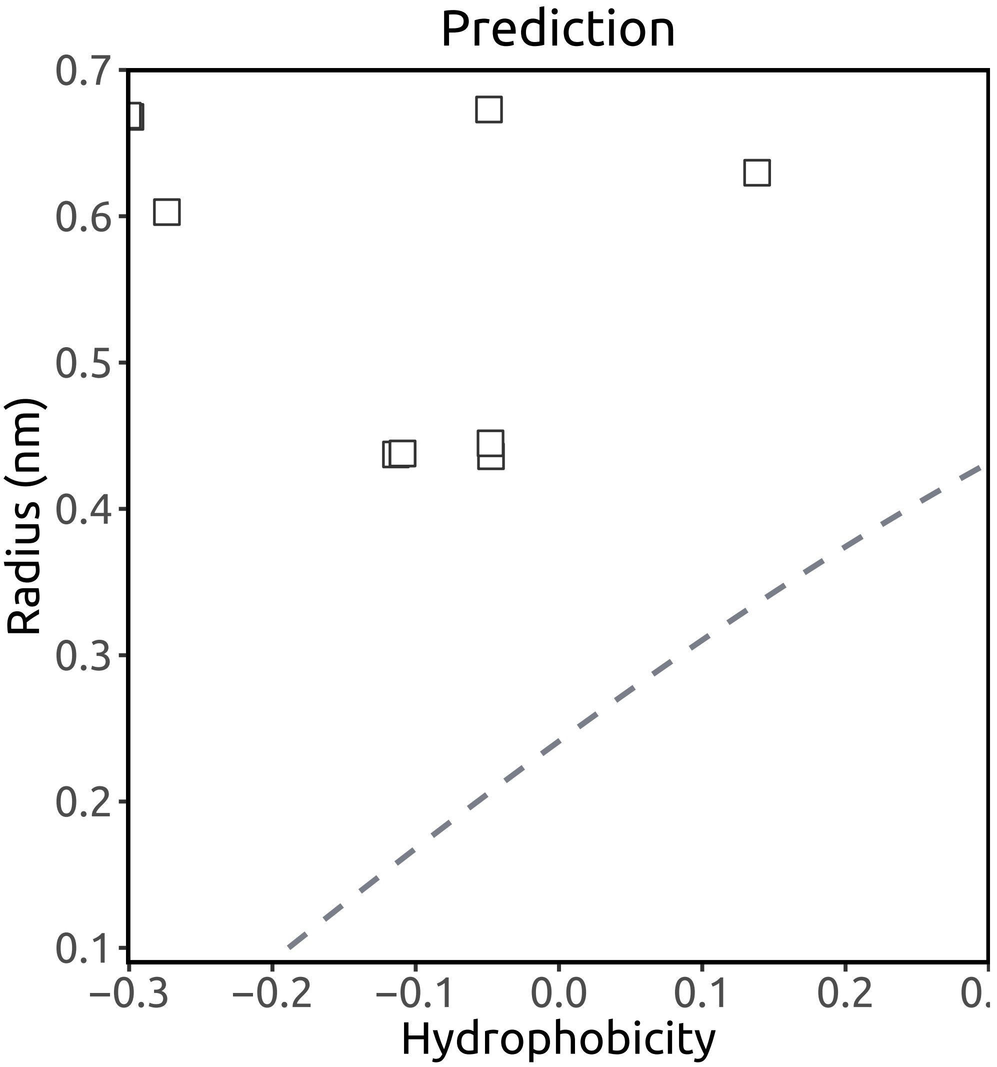


Heuristic score:
0 (n = 0)

Simulation result:
hydrated channel

# P2X4 (PDB ID: 3H9V)

*Danio rerio*
X−ray (3.1 Å)

Kawate et al., 2009

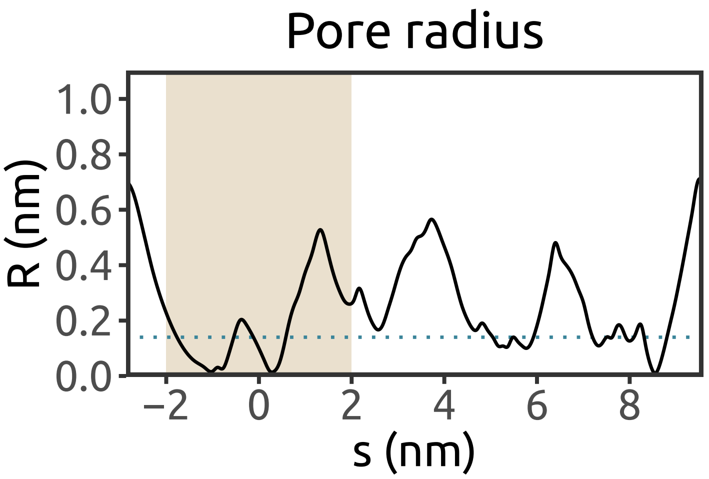


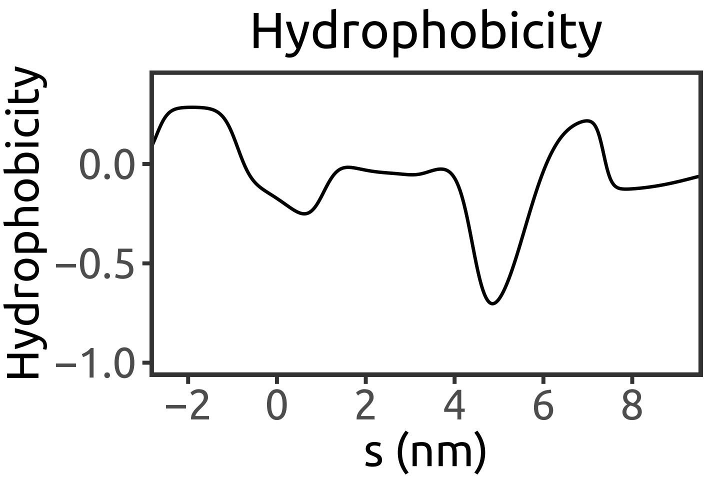


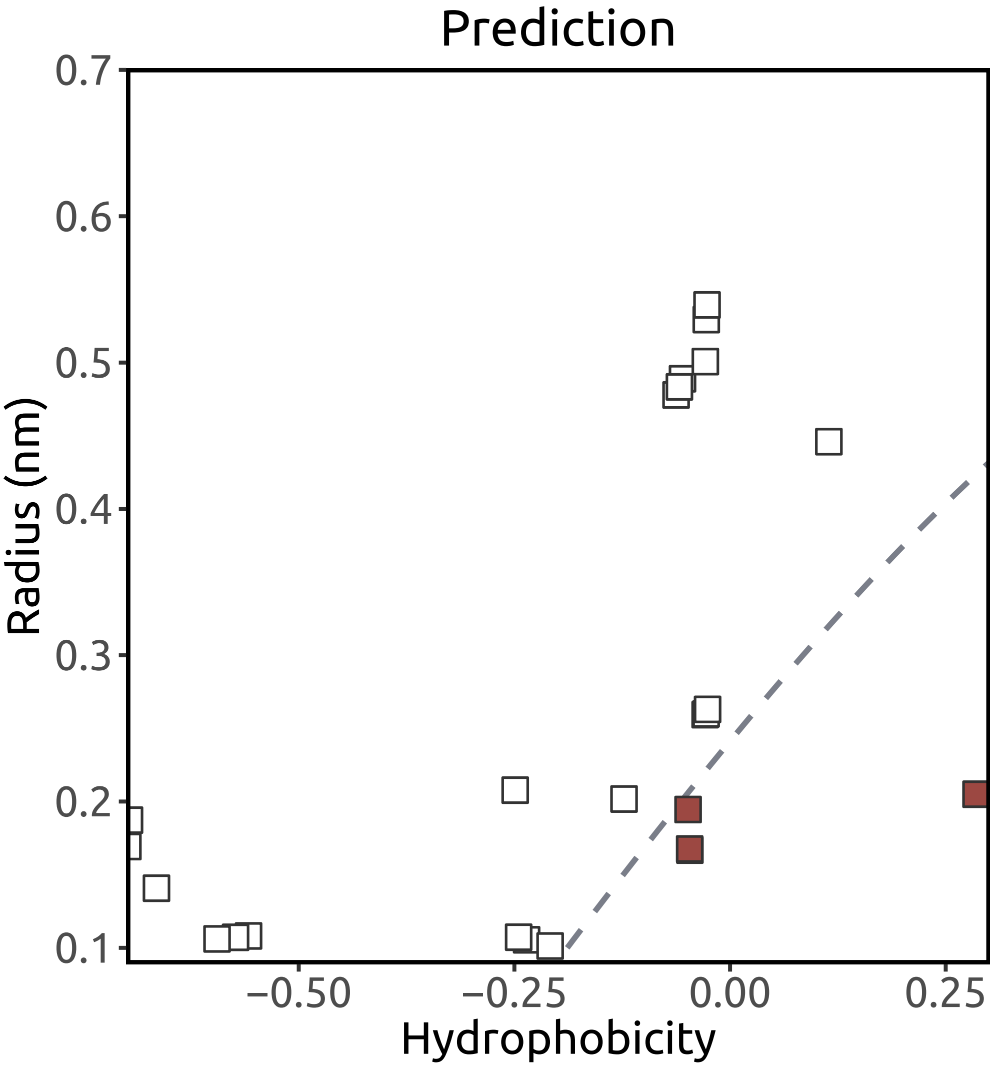


Heuristic score:
0.94 (n = 9)

Simulation result:
barrier to water

# P2X4 (PDB ID: 3I5D)

*Danio rerio*

X−ray (3.46 Å)

Kawate et al., 2009

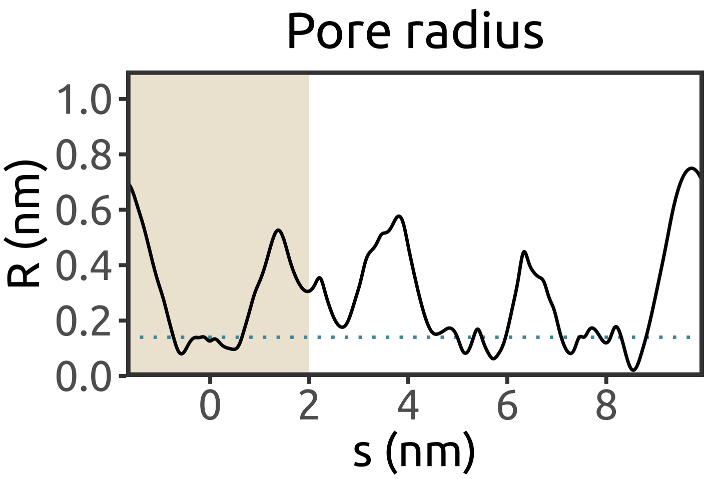


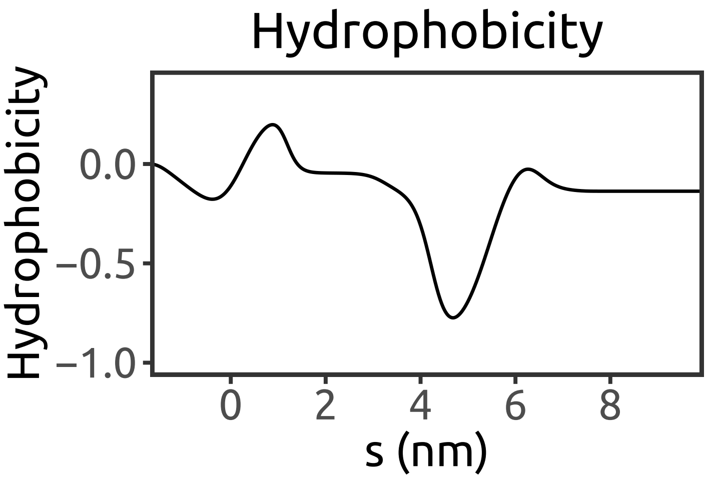


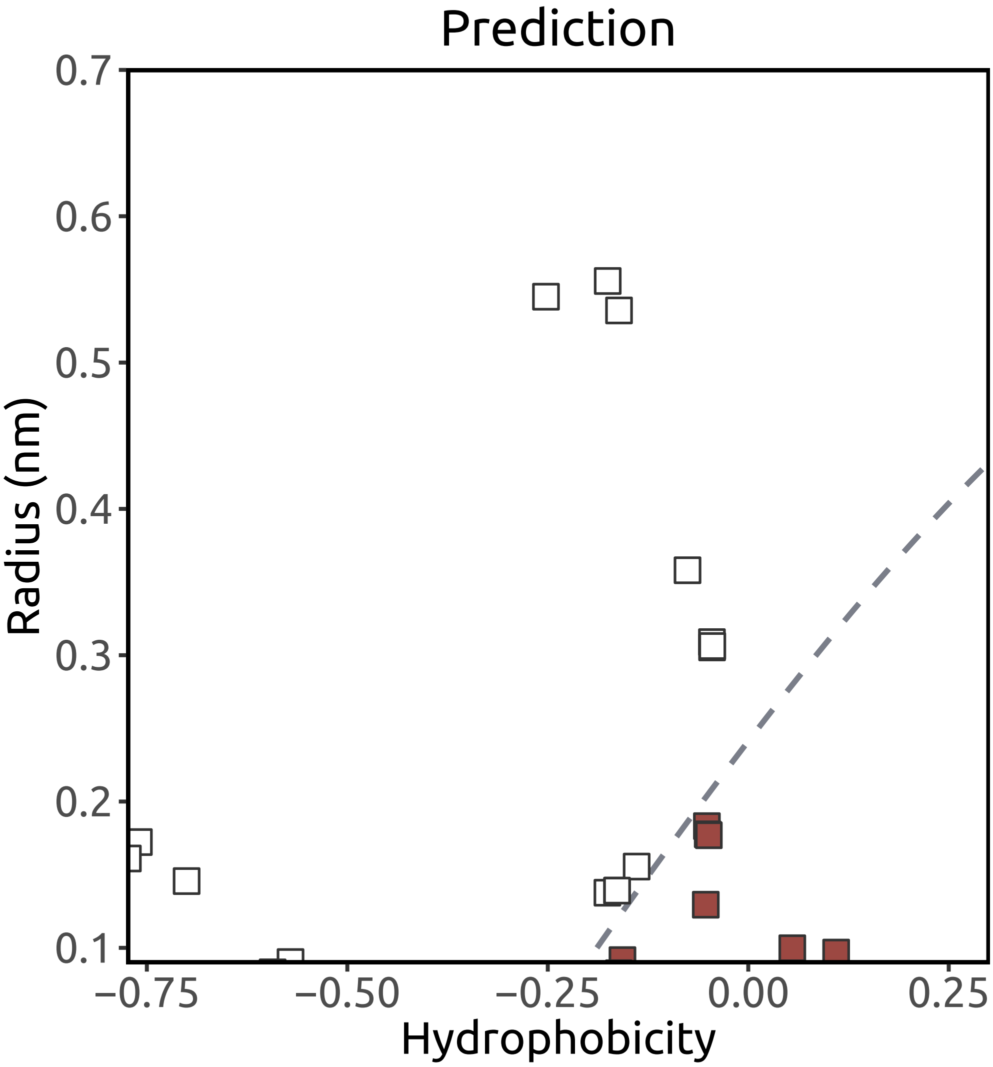


Heuristic score:

0.8 (n = 12)

Simulation result:

barrier to water

# P2X4 (PDB ID: 4DW0)

*Danio rerio*
X−ray (2.9 Å)

Hattori & Gouaux, 2012

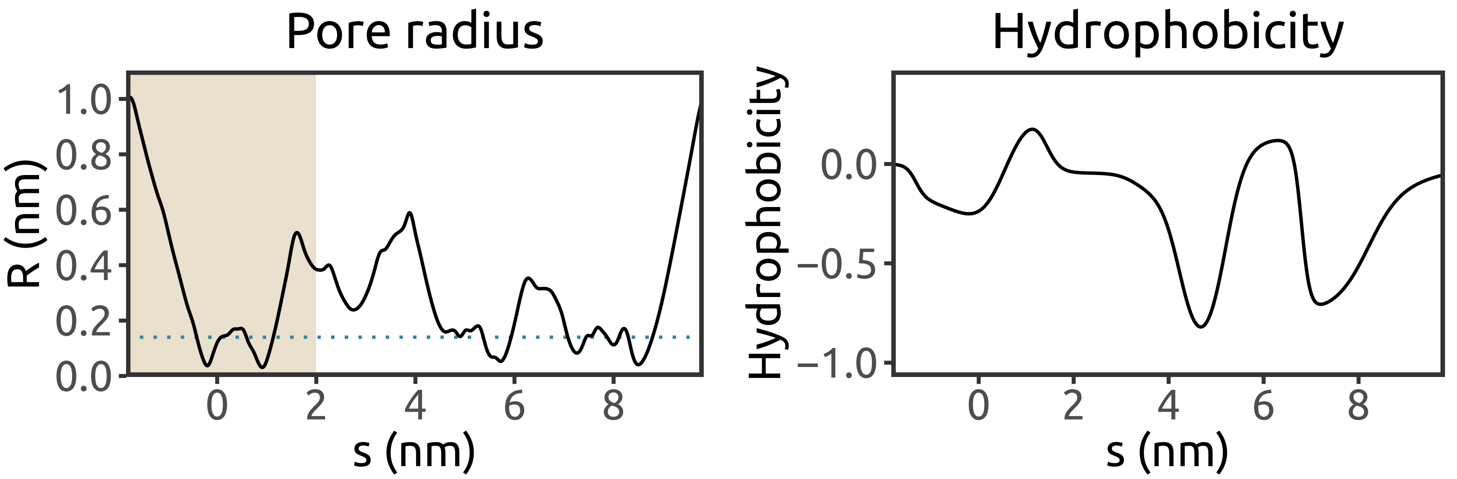


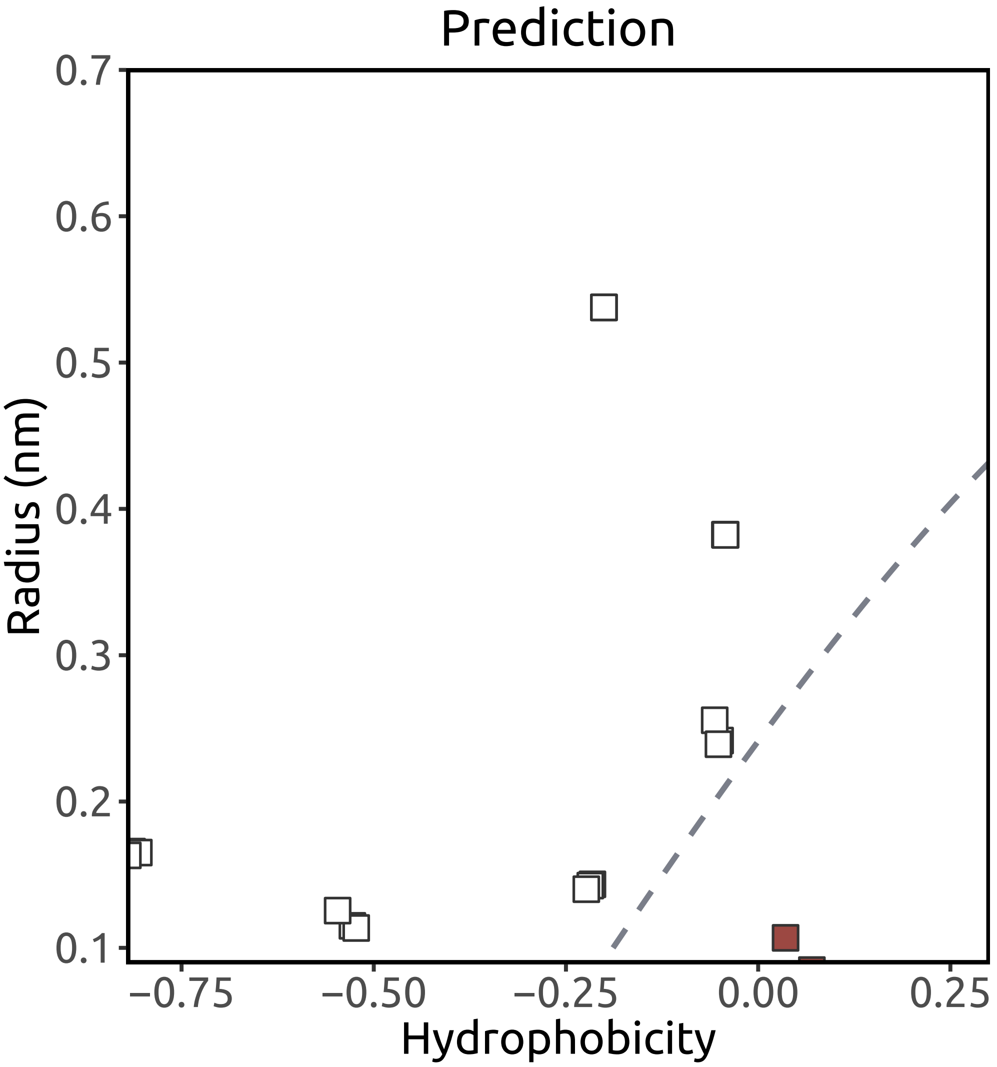


Heuristic score:
0.92 (n = 6)

Simulation result:
barrier to water

# P2X4 (PDB ID: 4DW1)

*Danio rerio*

X−ray (2.8 Å)

Hattori & Gouaux, 2012

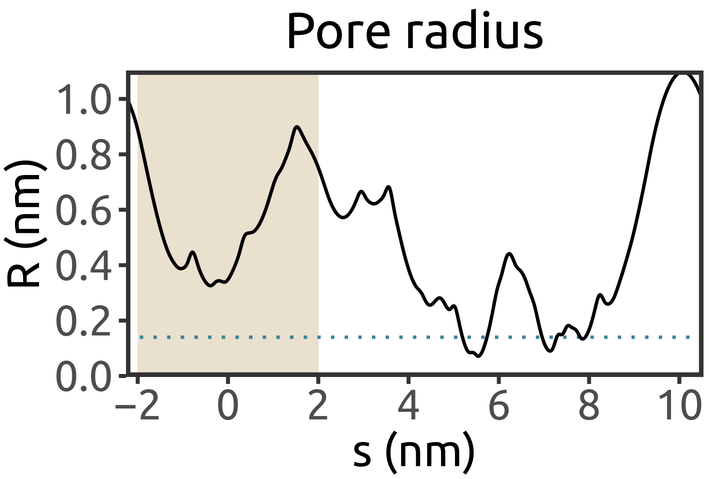


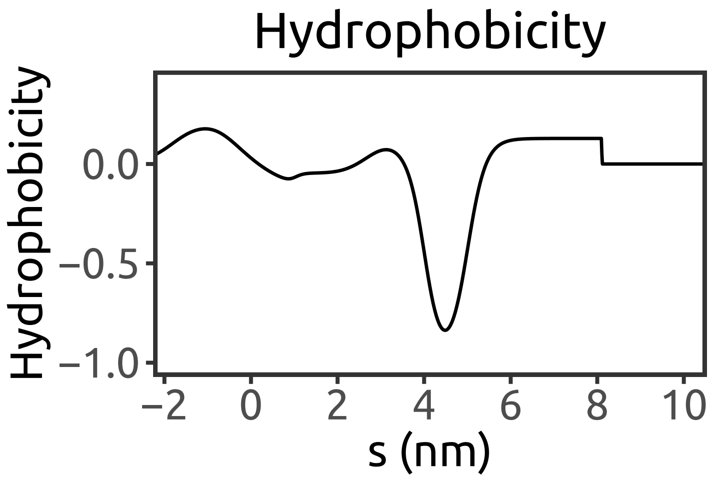


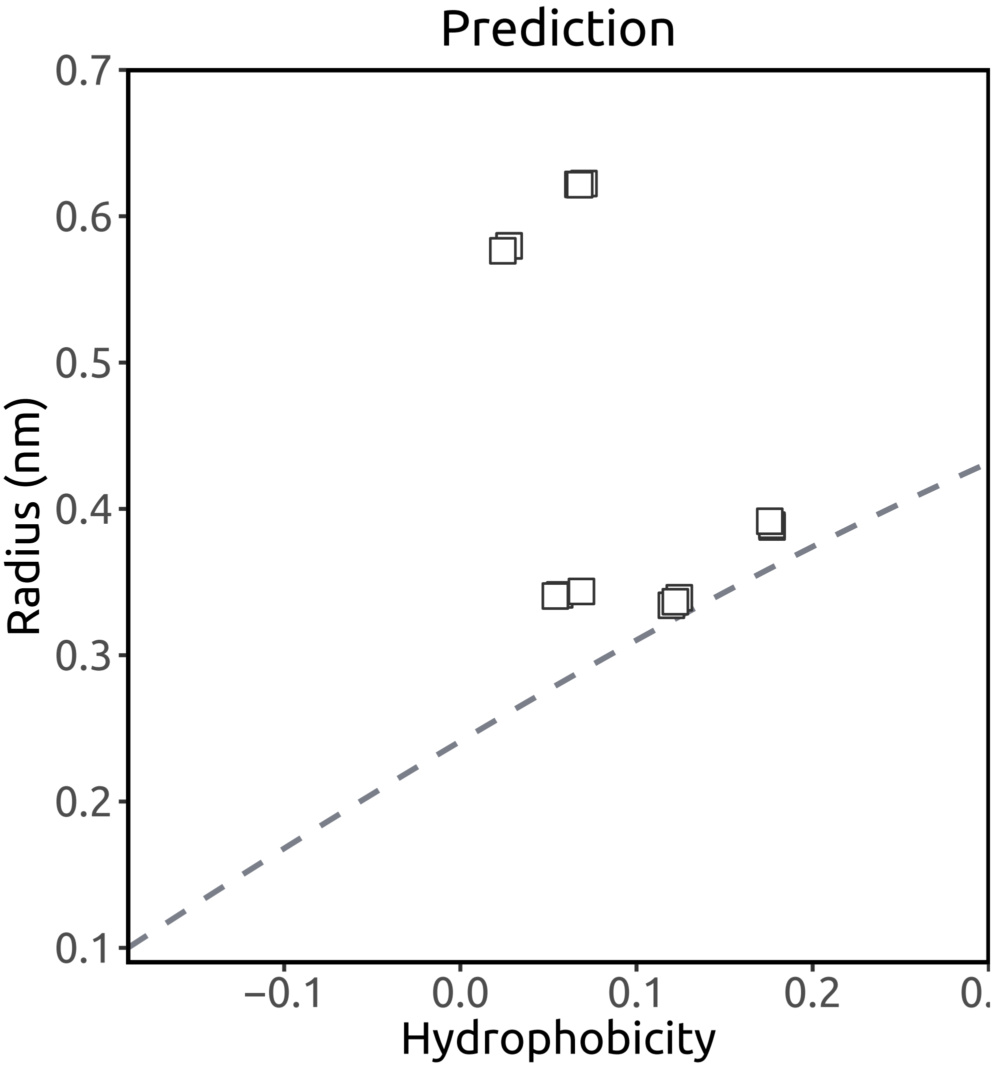


Heuristic score:

0 (n = 0)

Simulation result:

hydrated channel

# P2X7 (PDB ID: 5XW6)

*Gallus gallus*

X−ray (3.1 Å)

Kasuya et al., 2017

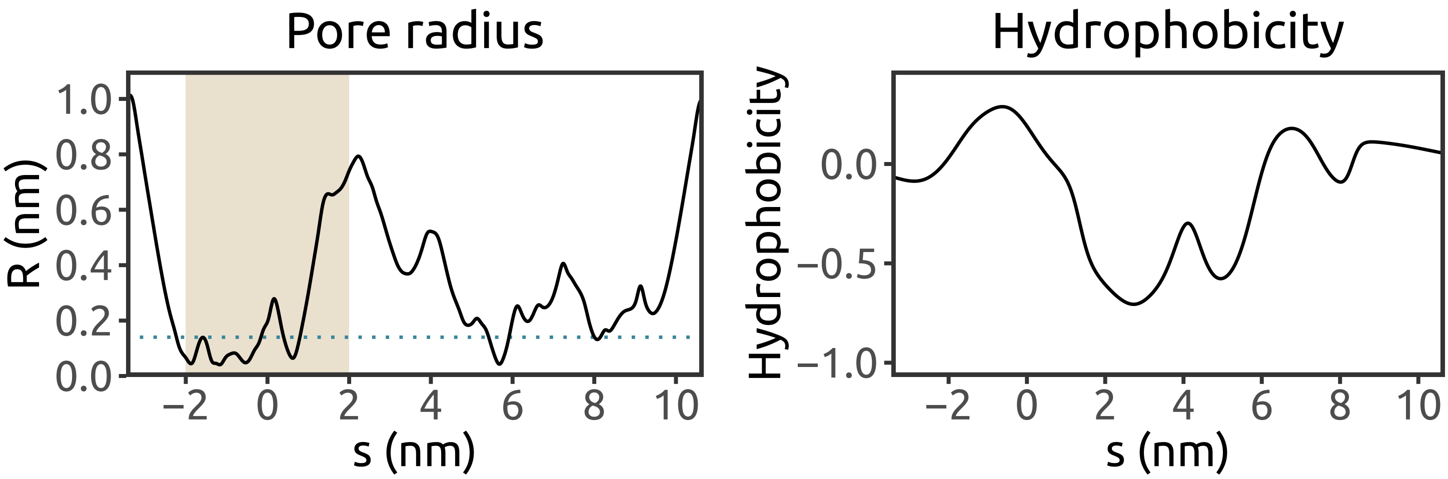


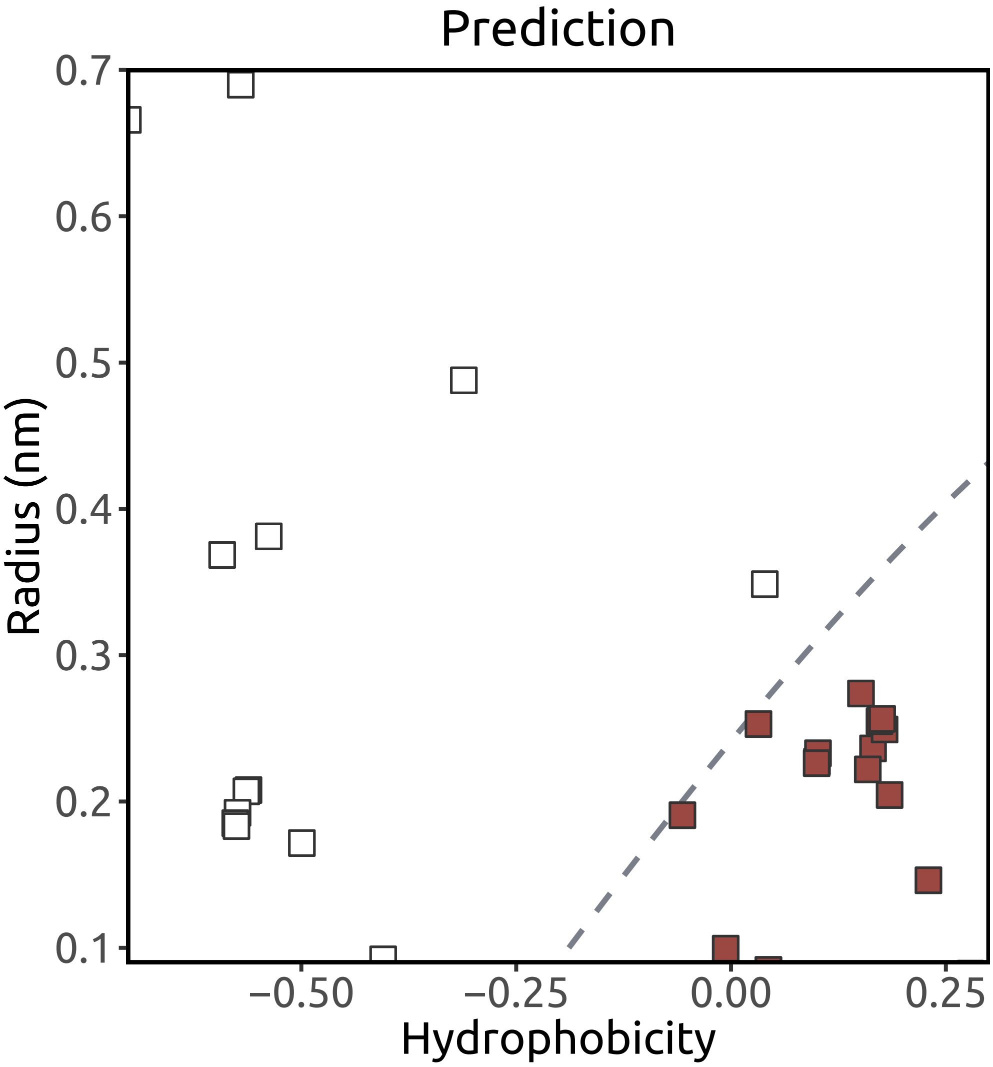


Heuristic score:

3.62 (n = 25)

Simulation result:

barrier to water

# NOMPC (PDB ID: 5VKQ)

*Drosophila melanogaster*
cryo–EM (3.55 Å)

Jin et al., 2017

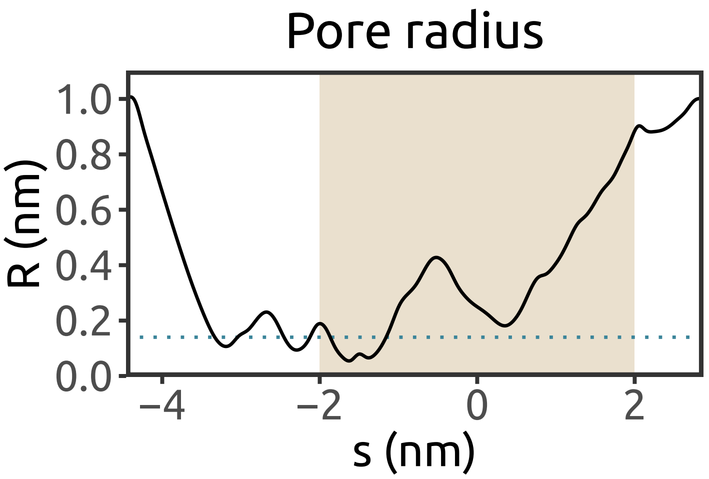


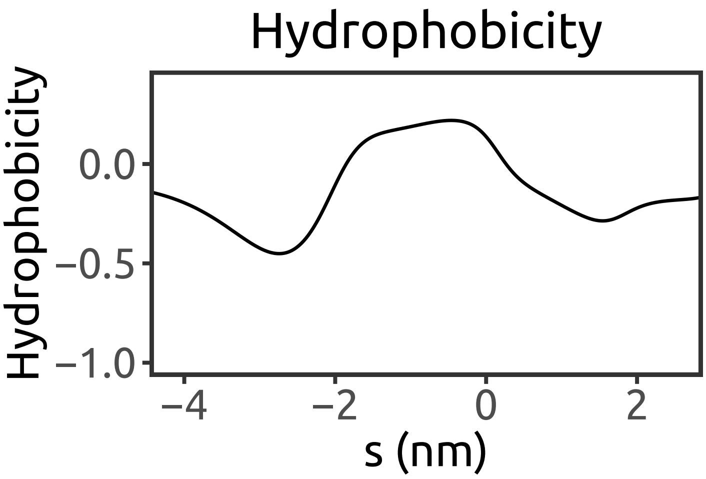


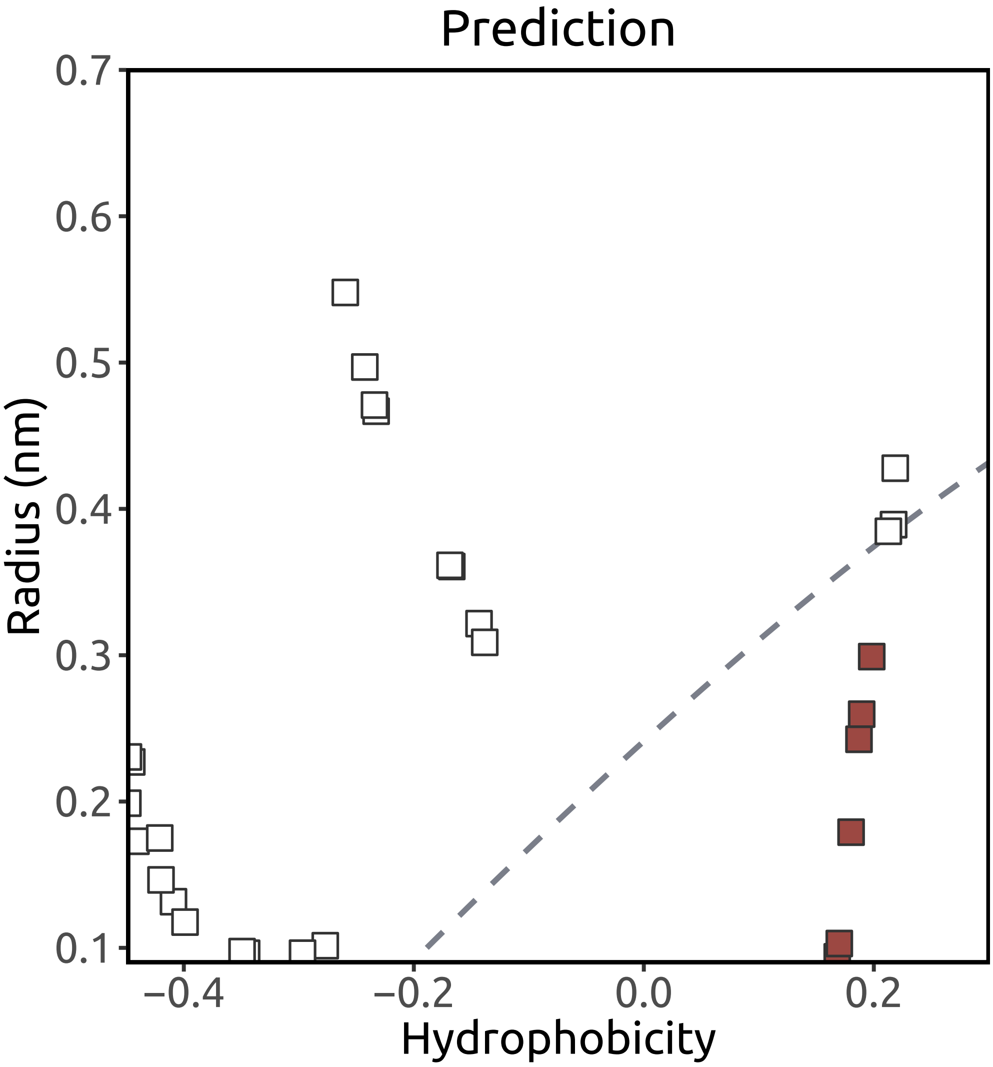


Heuristic score:
2.14 (n = 12)

Simulation result:
barrier to water

# PKD2 (PDB ID: 5K47)

*Homo sapiens*
cryo–EM (4.22 Å)

Grieben et al., 2017

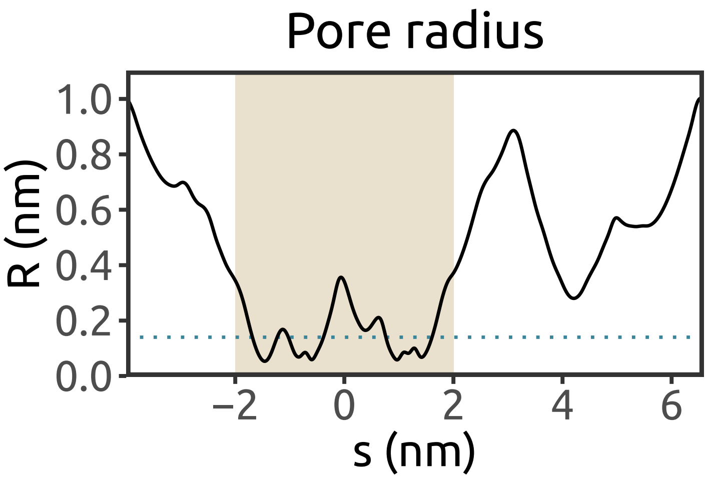


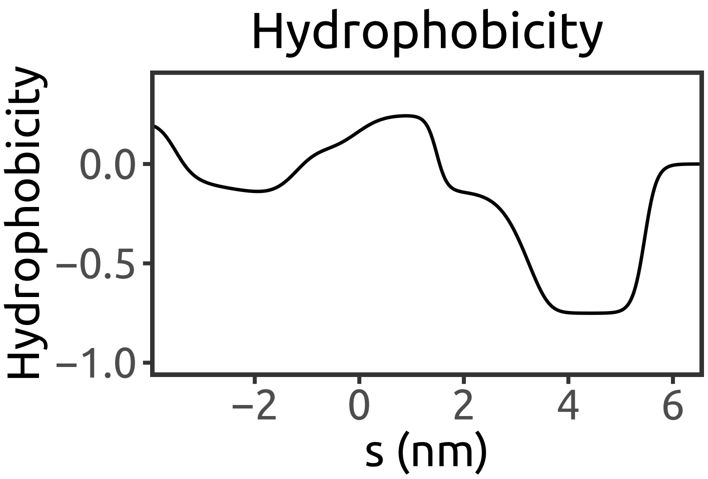


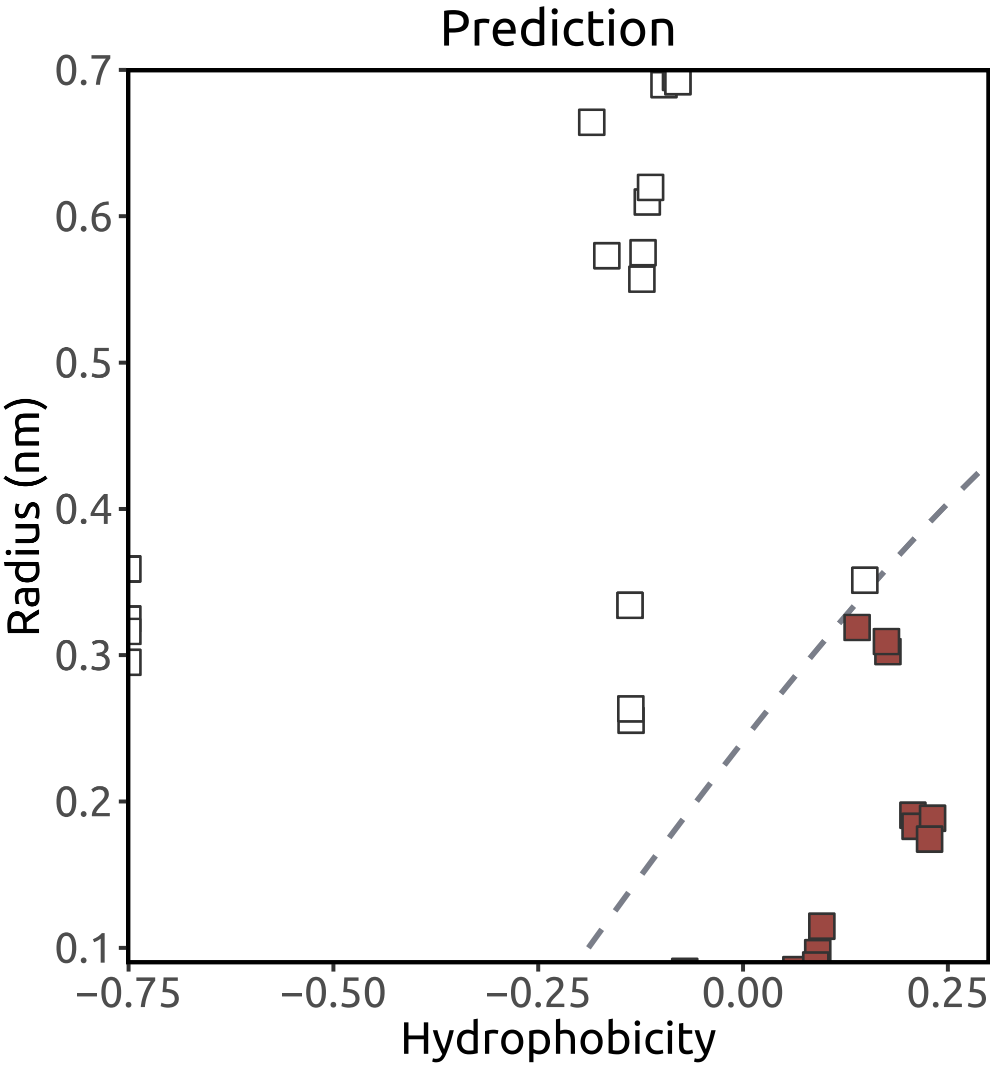


Heuristic score:
2.49 (n = 19)

Simulation result:
barrier to water

# PKD2 (PDB ID: 5MKE)

*Homo sapiens*
cryo−EM (4.3 Å)

Wilkes et al., 2017

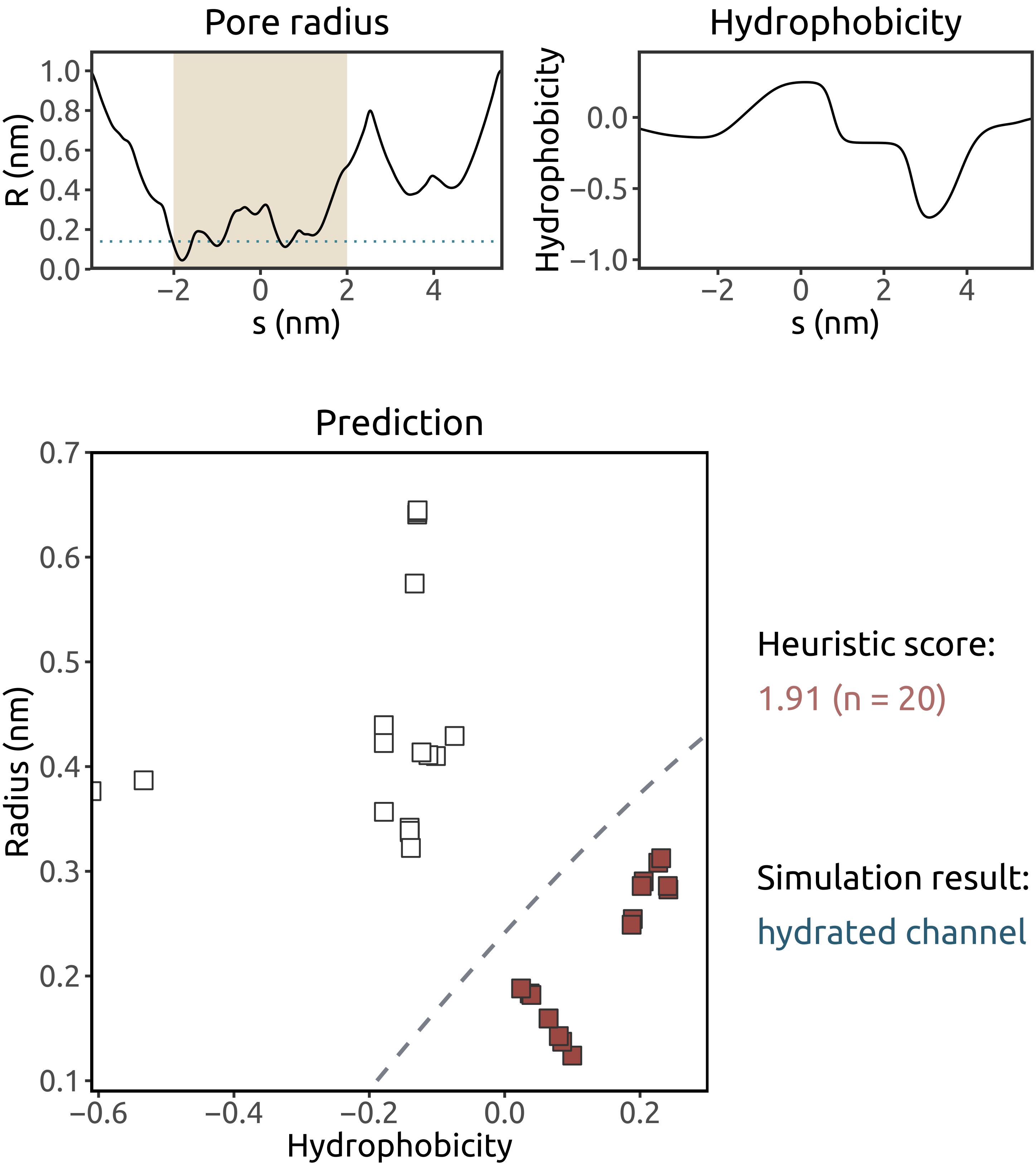


Heuristic score:
1.91 (n = 20)

Simulation result:
hydrated channel

# PKD2 (PDB ID: 5MKF)

*Homo sapiens*
cryo−EM (4.2 Å)

Wilkes et al., 2017

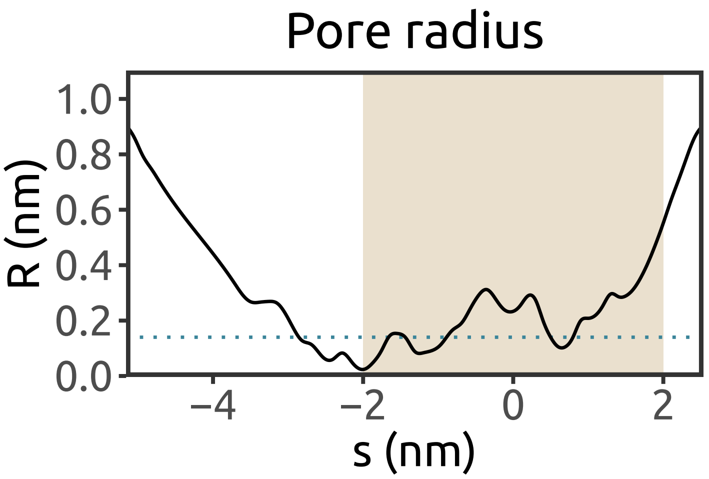


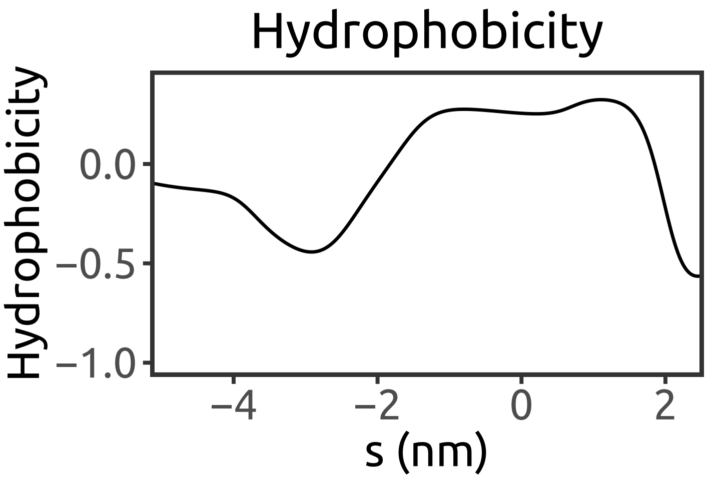


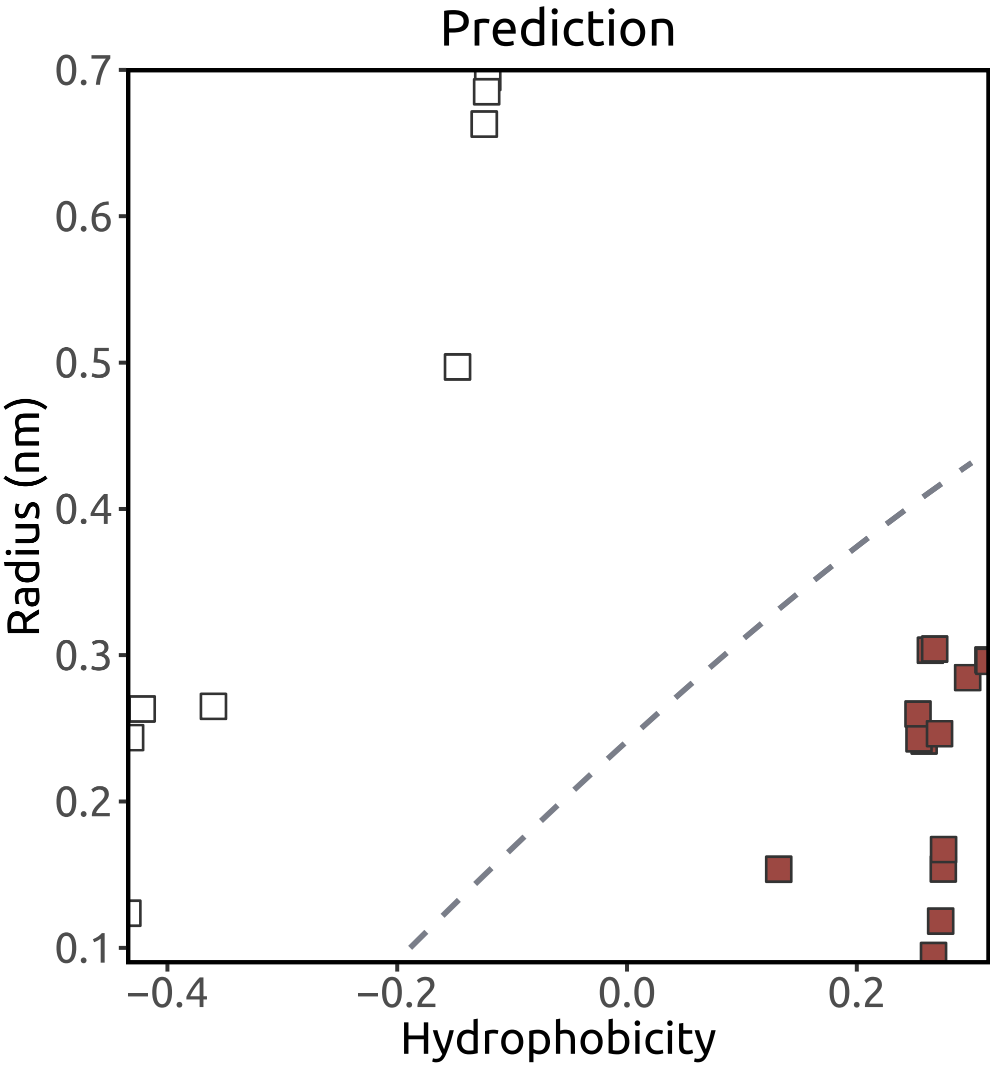


Heuristic score:
2.5 (n = 16)

Simulation result:
barrier to water

# PKD2 (PDB ID: 5T4D)

*Homo sapiens*
cryo−EM (3 Å)

Shen et al., 2016

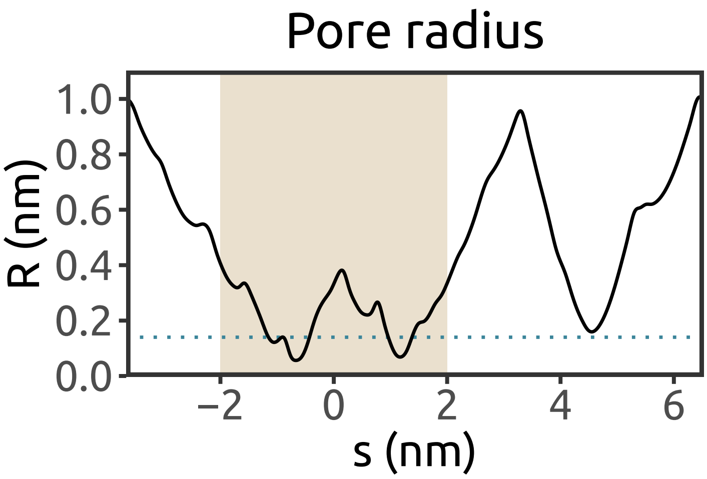


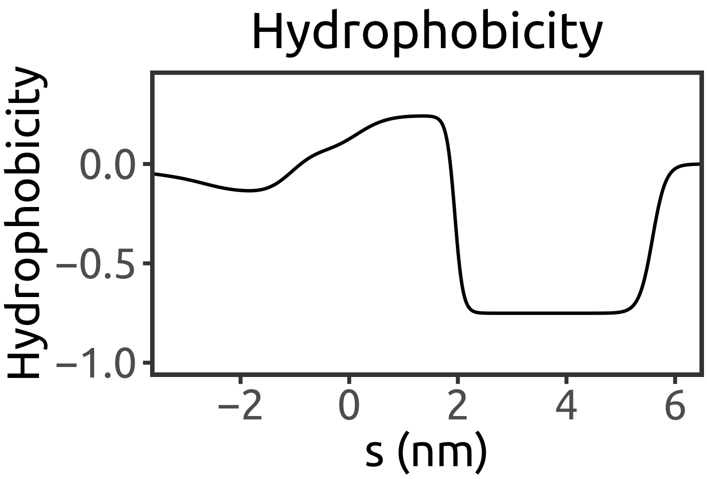


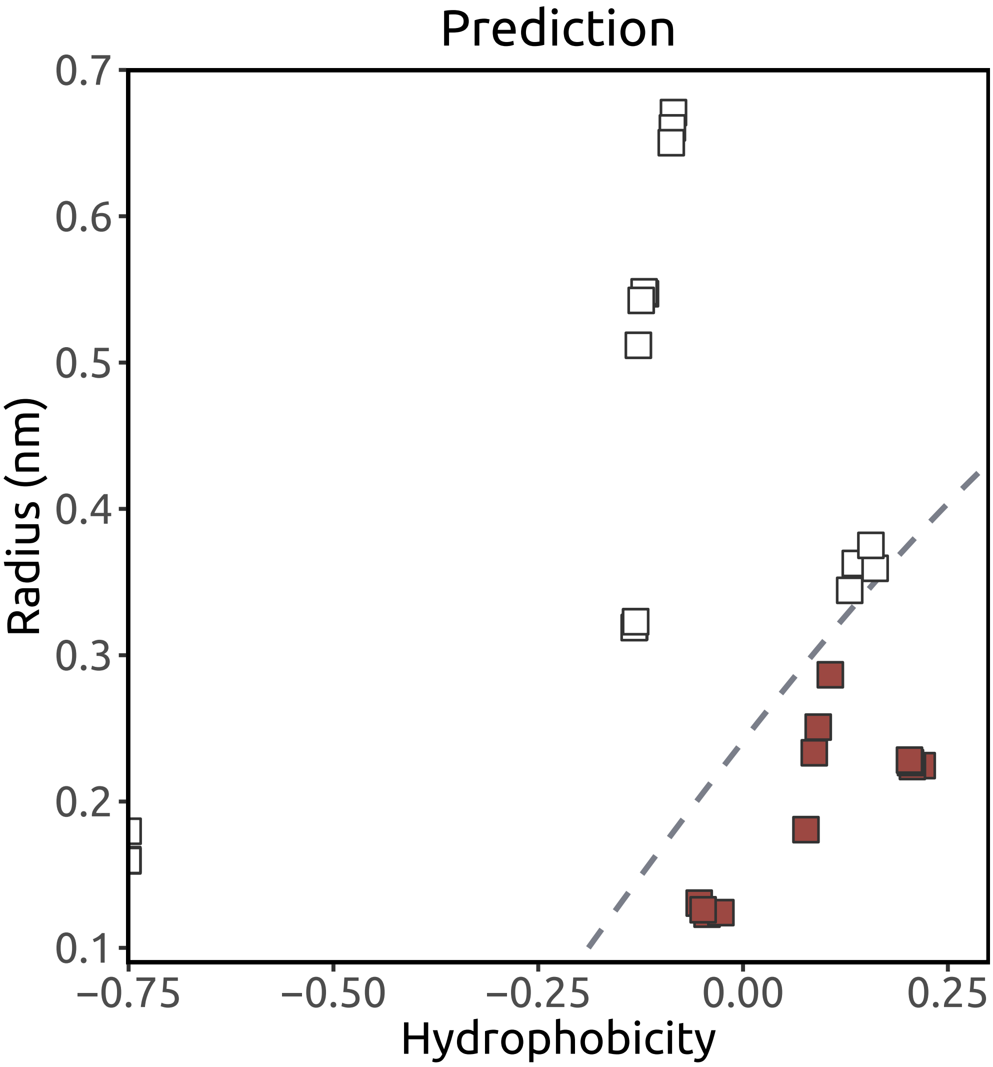


Heuristic score:
1.71 (n = 16)

Simulation result:
barrier to water

# PKD2 (PDB ID: 6D1W)

*Homo sapiens*
cryo−EM (3.54 Å)

Zheng et al., 2018

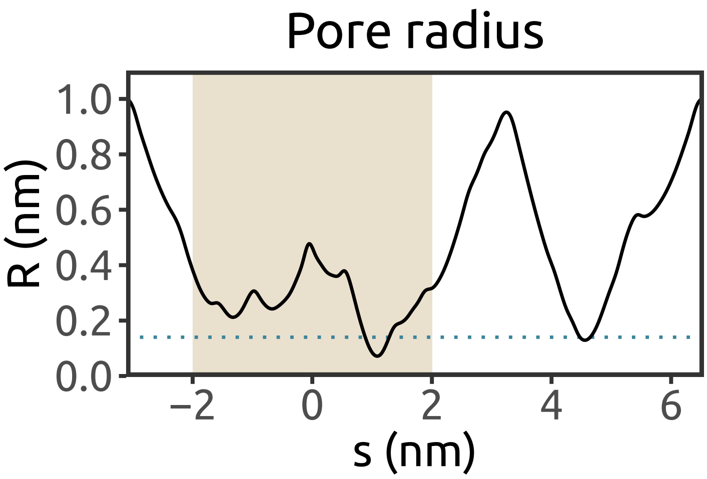


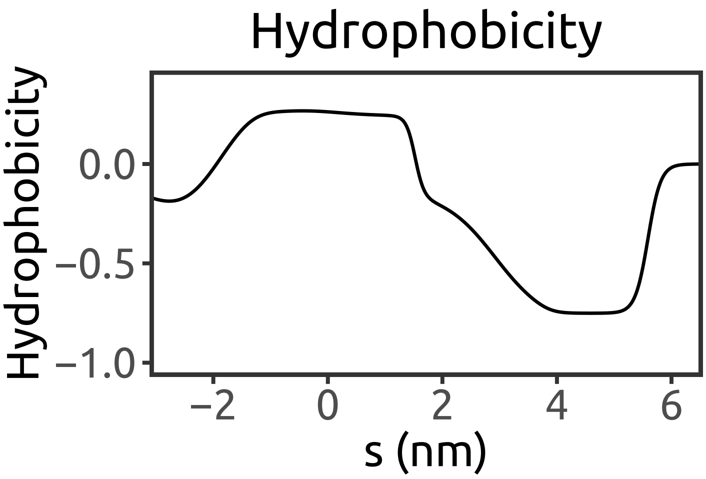


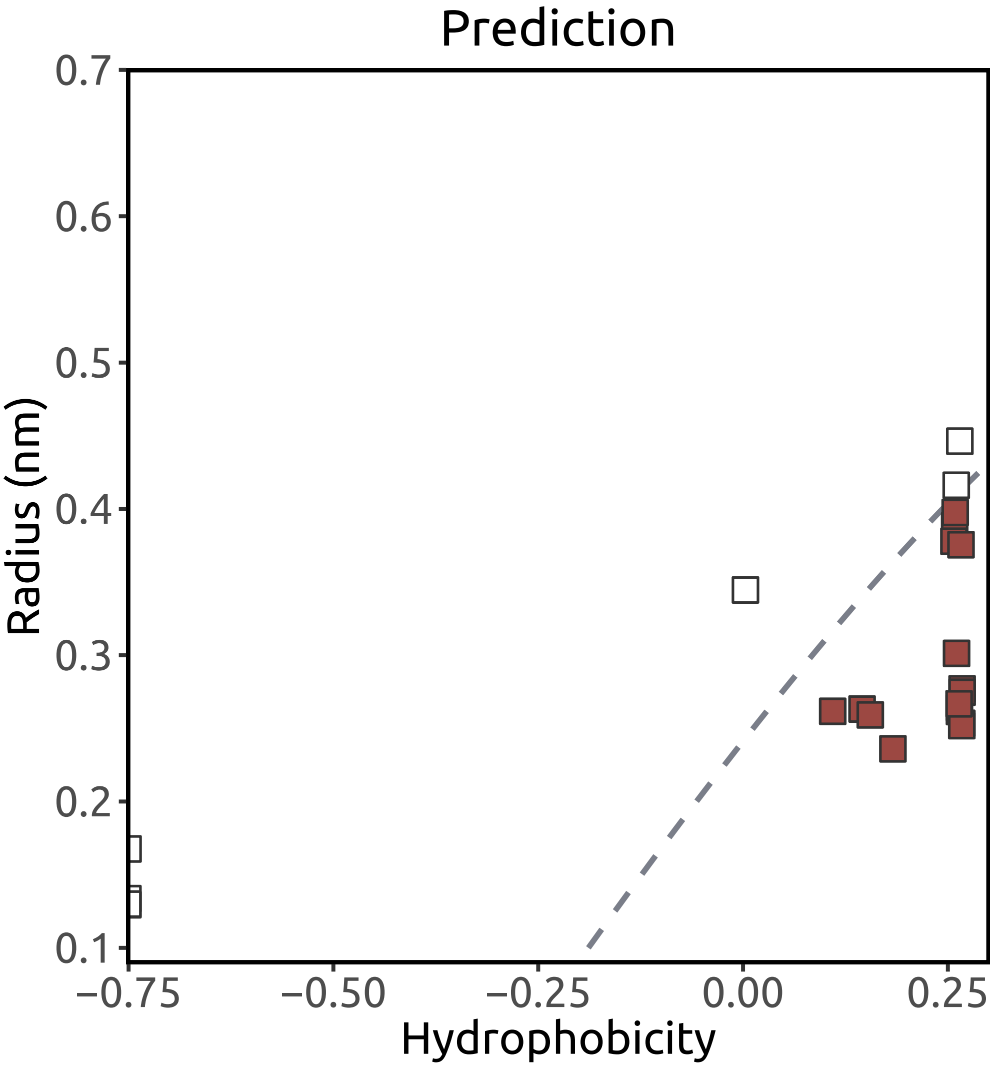


Heuristic score:

1.37 (n = 16)

Simulation result:

barrier to water

# PKD2L1 (PDB ID: 5Z1W)

*Mus musculus*
cryo−EM (3.38 Å)

Su et al., 2018

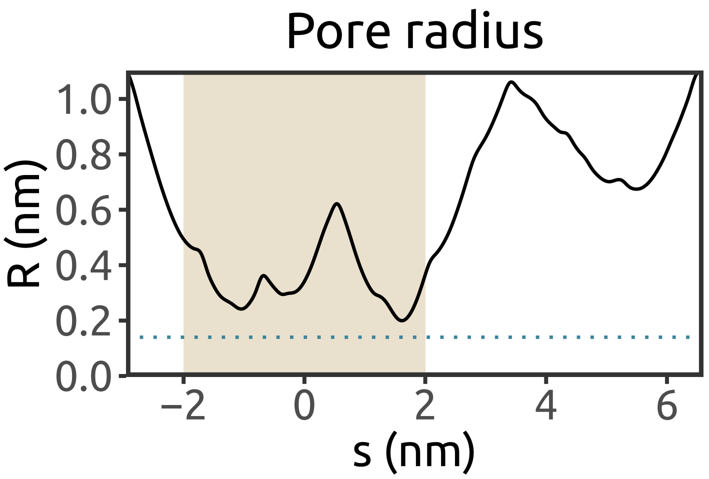


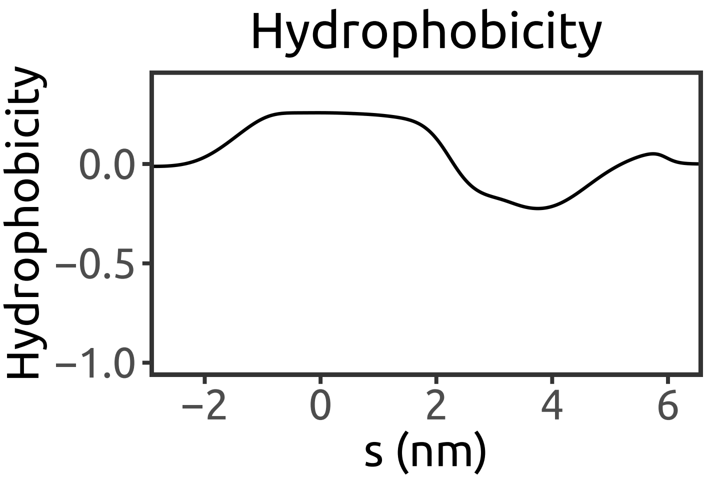


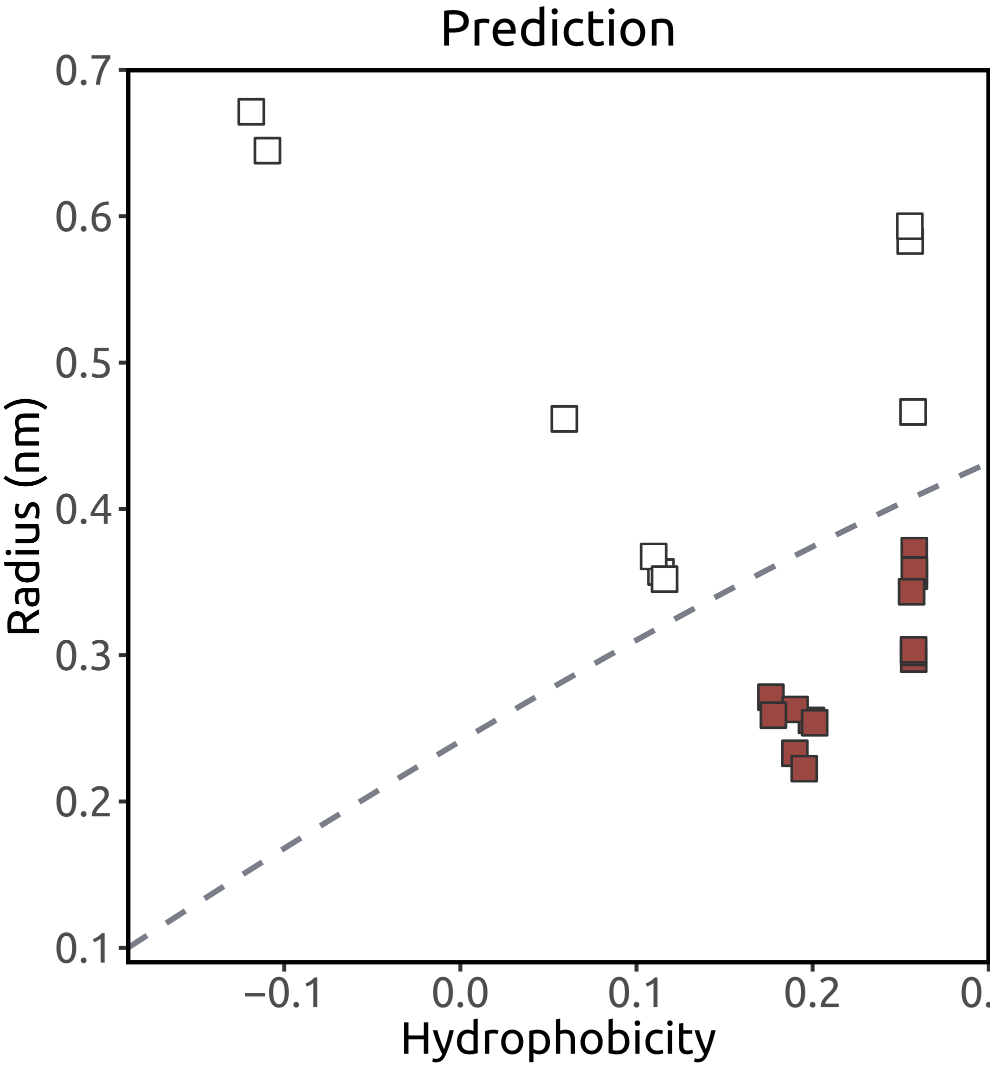


Heuristic score:
1.15 (n = 14)

Simulation result:
barrier to water

# TRPA1 (PDB ID: 3J9P)

*Homo sapiens*
cryo−EM (4.24 Å)

Paulsen et al., 2015

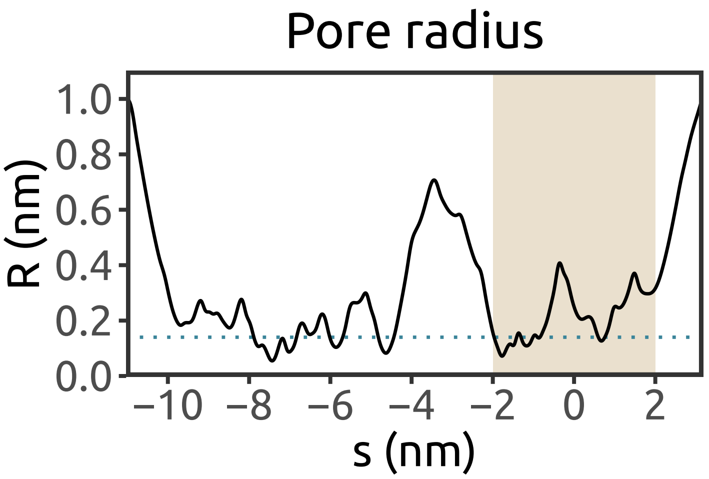


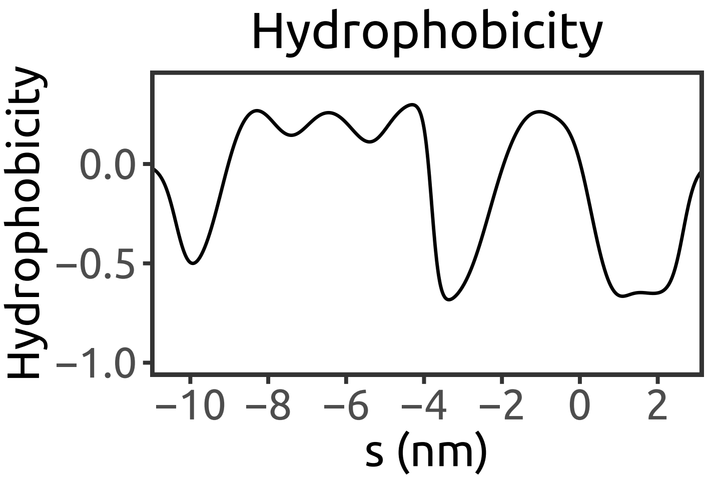


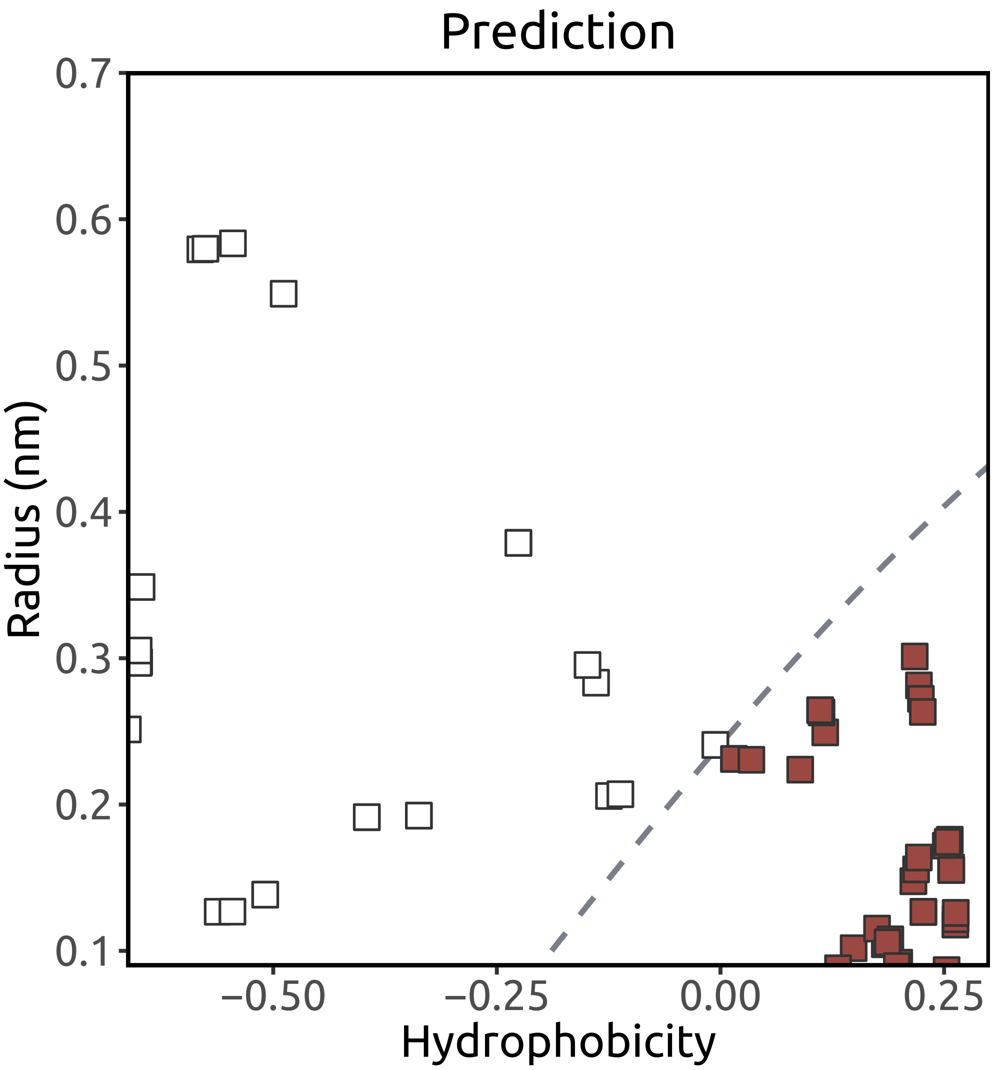


Heuristic score:

8.6 (n = 48)

Simulation result:

barrier to water

# TRPM2 (PDB ID: 6CO7)

*Nematostella vectensis*
cryo−EM (3.07 Å)

Zhang et al., 2018

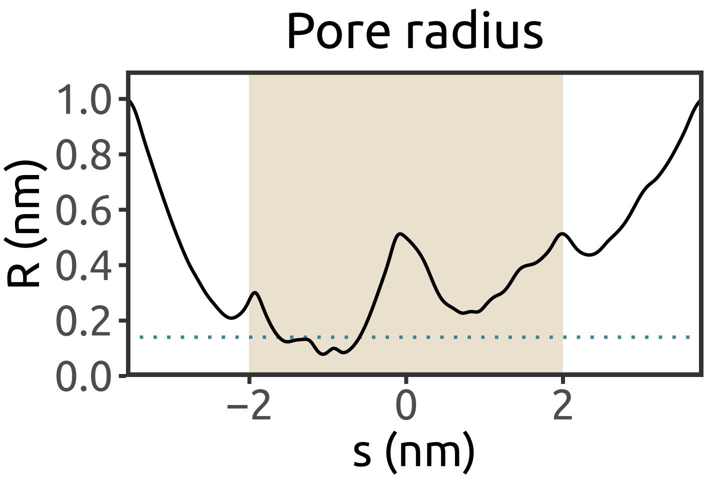


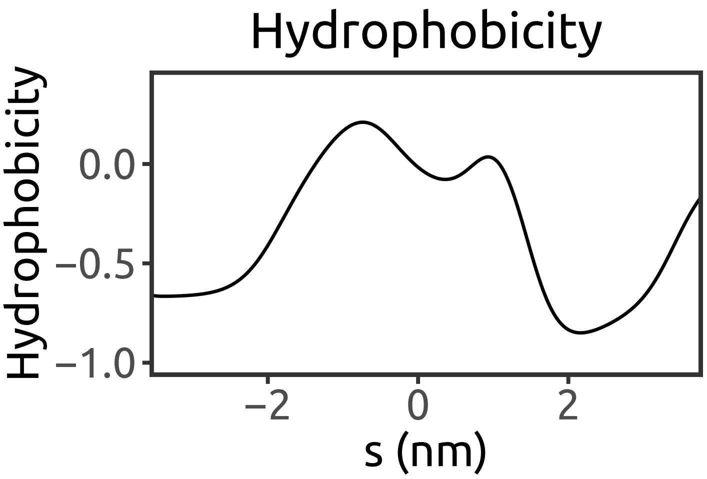


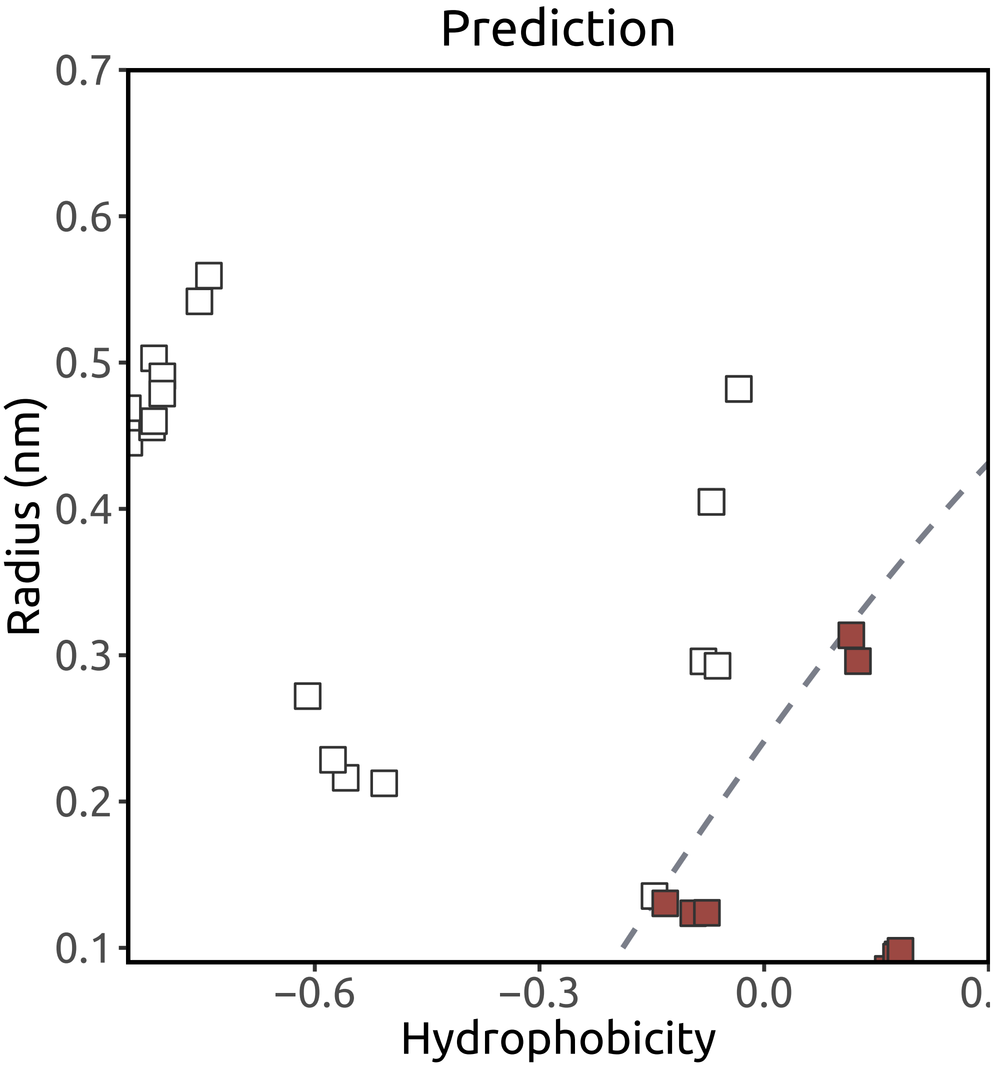


Heuristic score:
1.02 (n = 9)

Simulation result:
barrier to water

# TRPM4 (PDB ID: 6BCL)

*Mus musculus*
cryo−EM (3.54 Å)

Guo et al., 2017

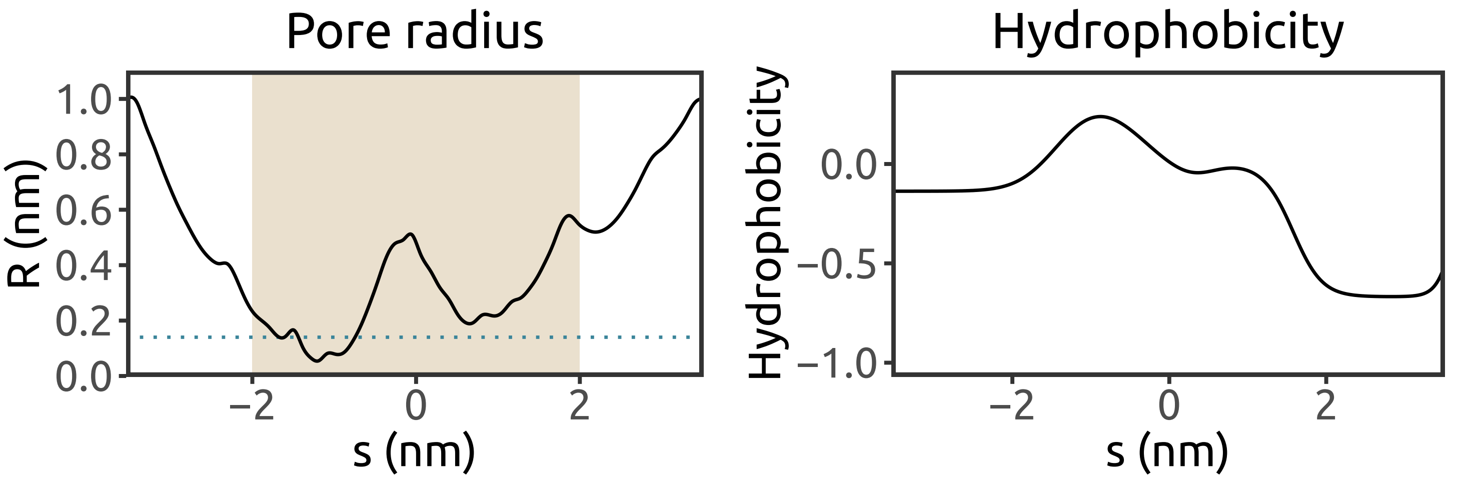


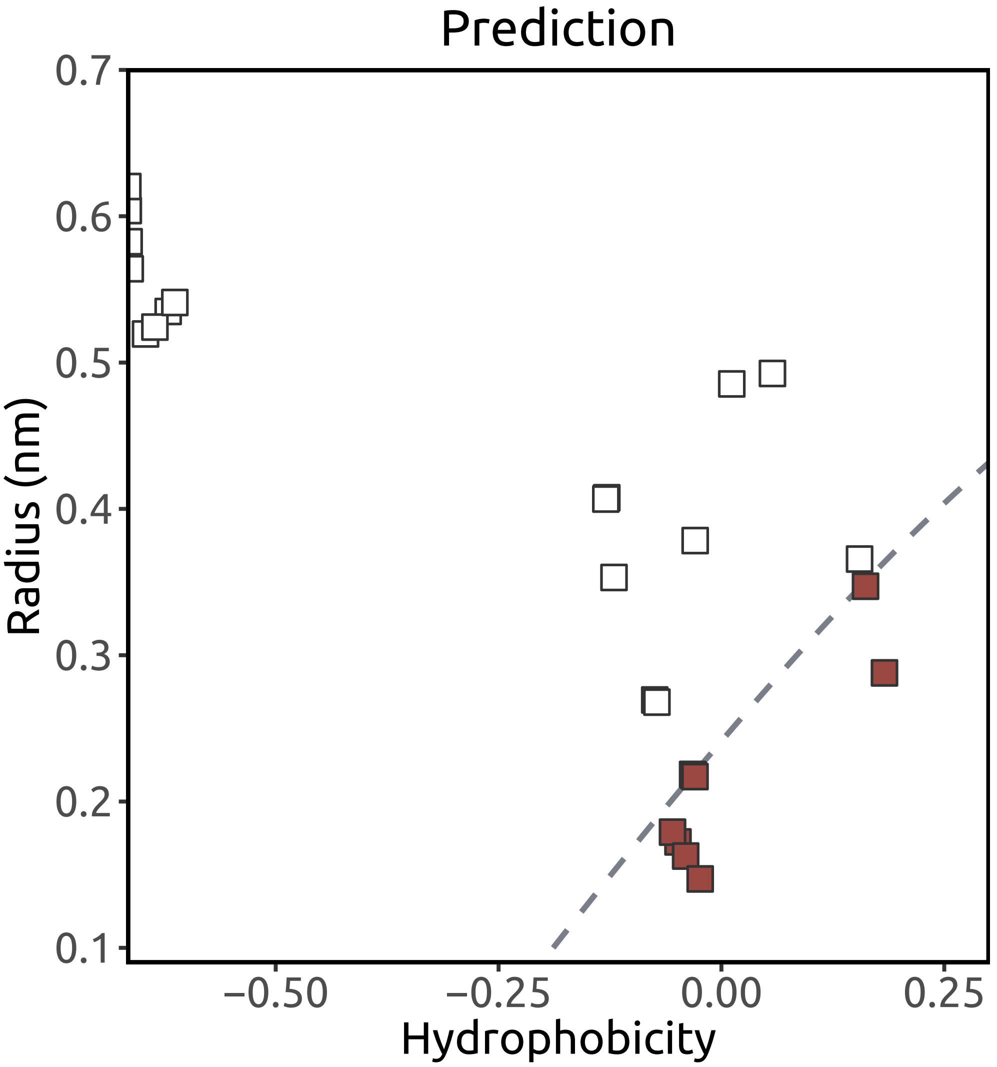


Heuristic score:
1.26 (n = 13)

Simulation result:
barrier to water

# TRPM4 (PDB ID: 6BCQ)

*Mus musculus*
cryo–EM (3.25 Å)

Guo et al., 2017

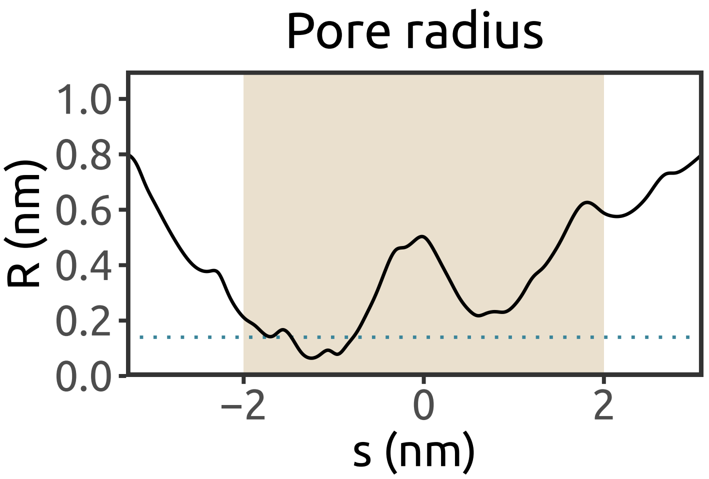


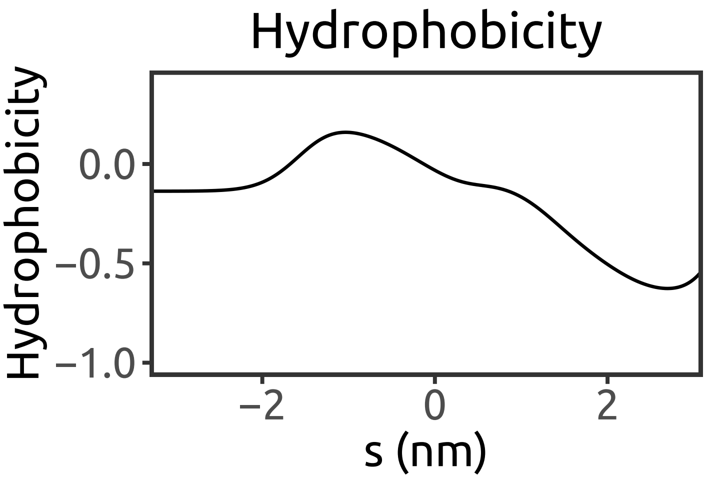


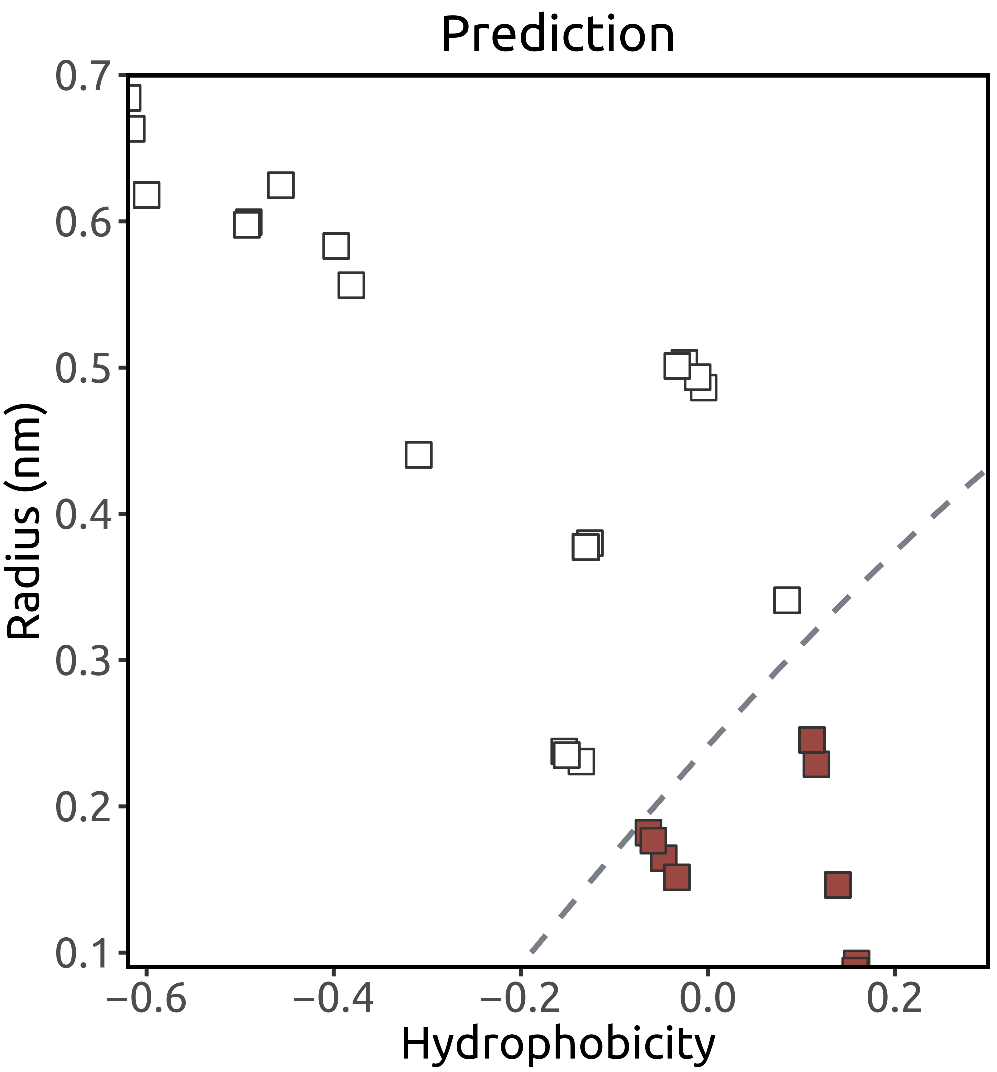


Heuristic score:
1.45 (n = 12)

Simulation result:
barrier to water

# TRPM4 (PDB ID: 6BQR)

*Homo sapiens*
cryo–EM (3.2 Å)

Autzen et al., 2018

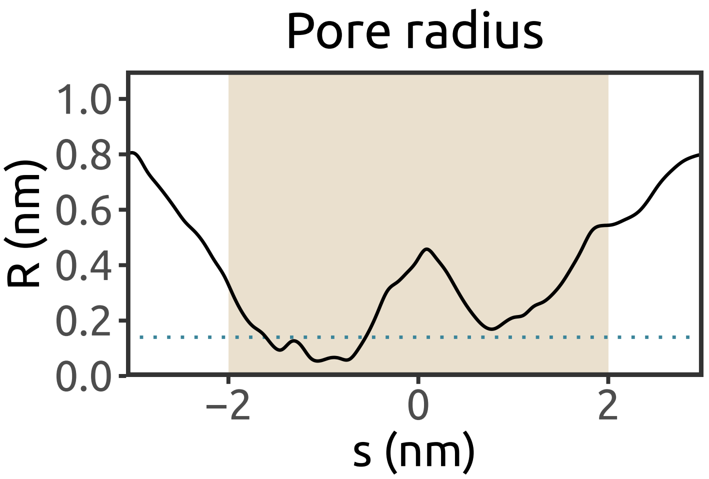


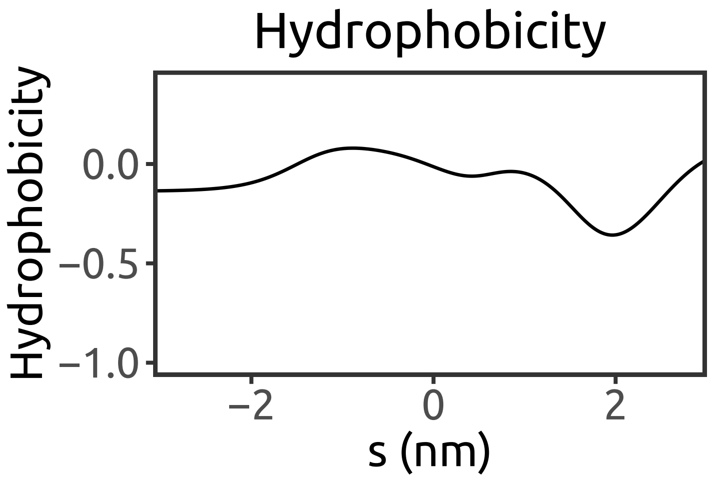


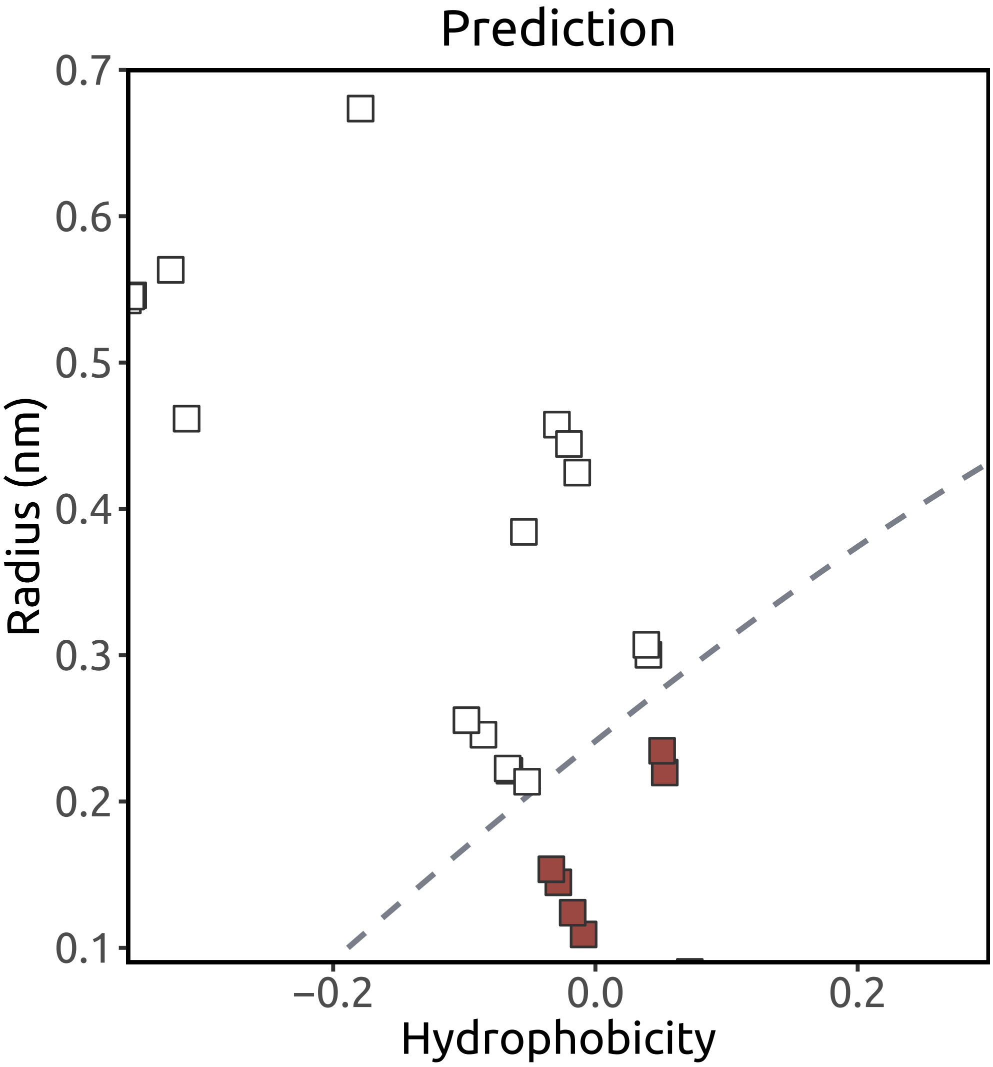


Heuristic score:
1.87 (n = 14)

Simulation result:
barrier to water

# TRPML1 (PDB ID: 5WJ5)

*Homo sapiens*
cryo−EM (3.72 Å)

Schmiege et al., 2017

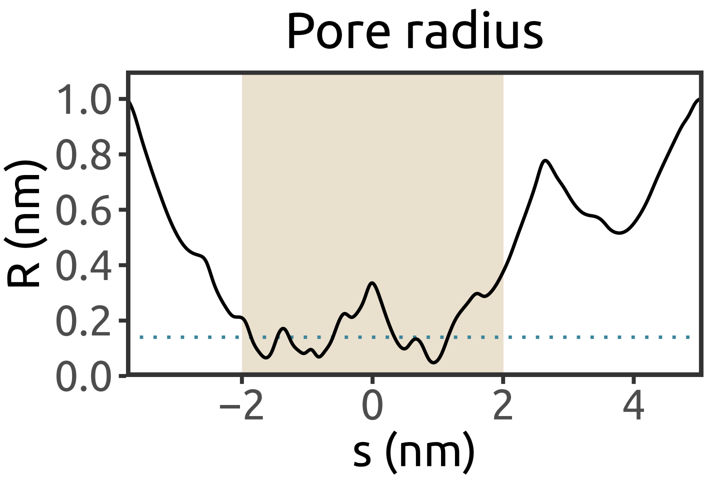


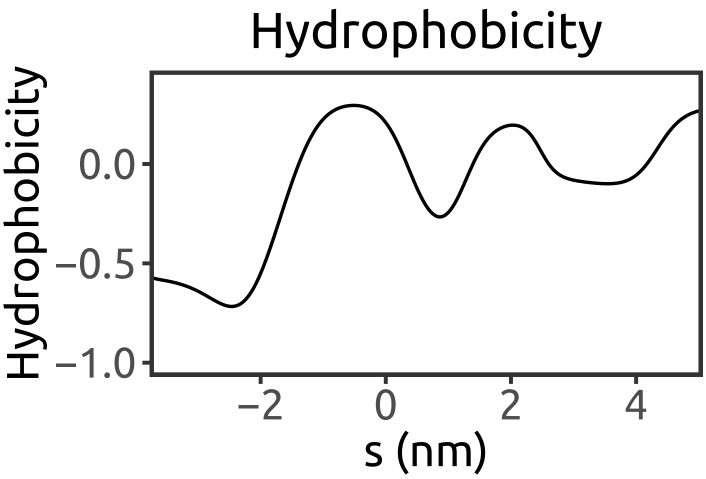


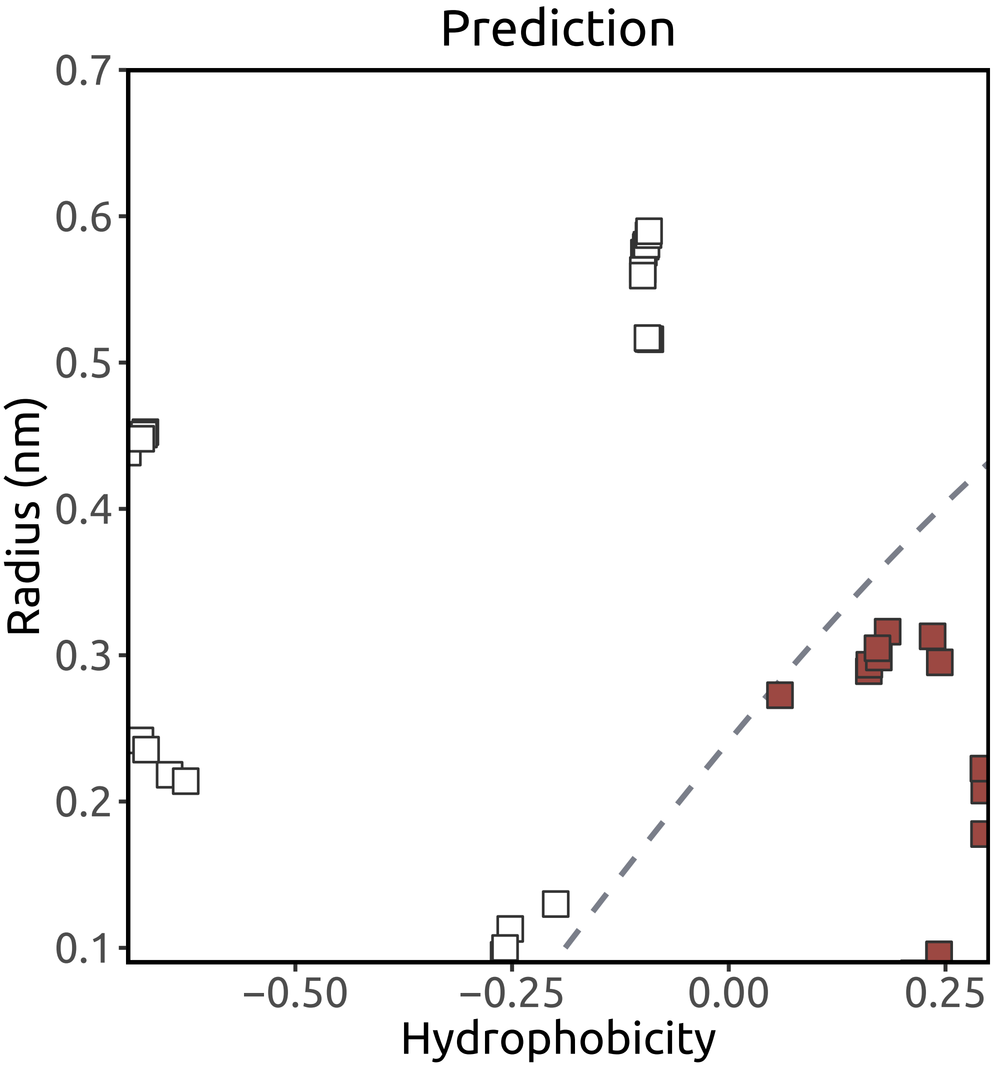


Heuristic score:
2.22 (n = 17)

Simulation result:
barrier to water

# TRPML1 (PDB ID: 5WJ9)

*Homo sapiens*
cryo–EM (3.49 Å)

Schmiege et al., 2017

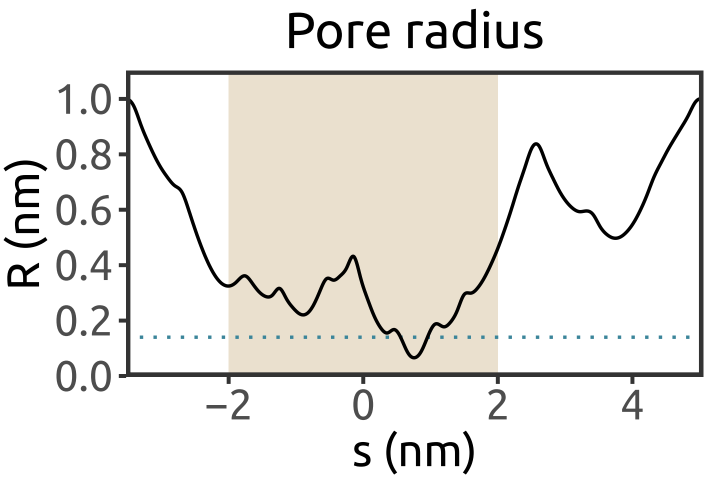


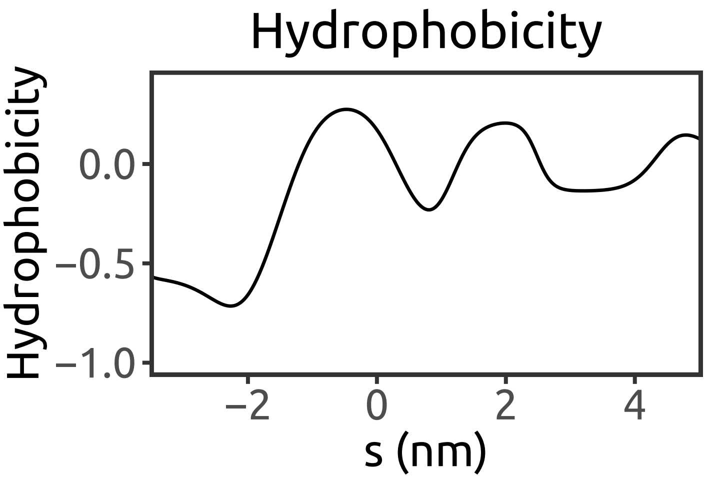


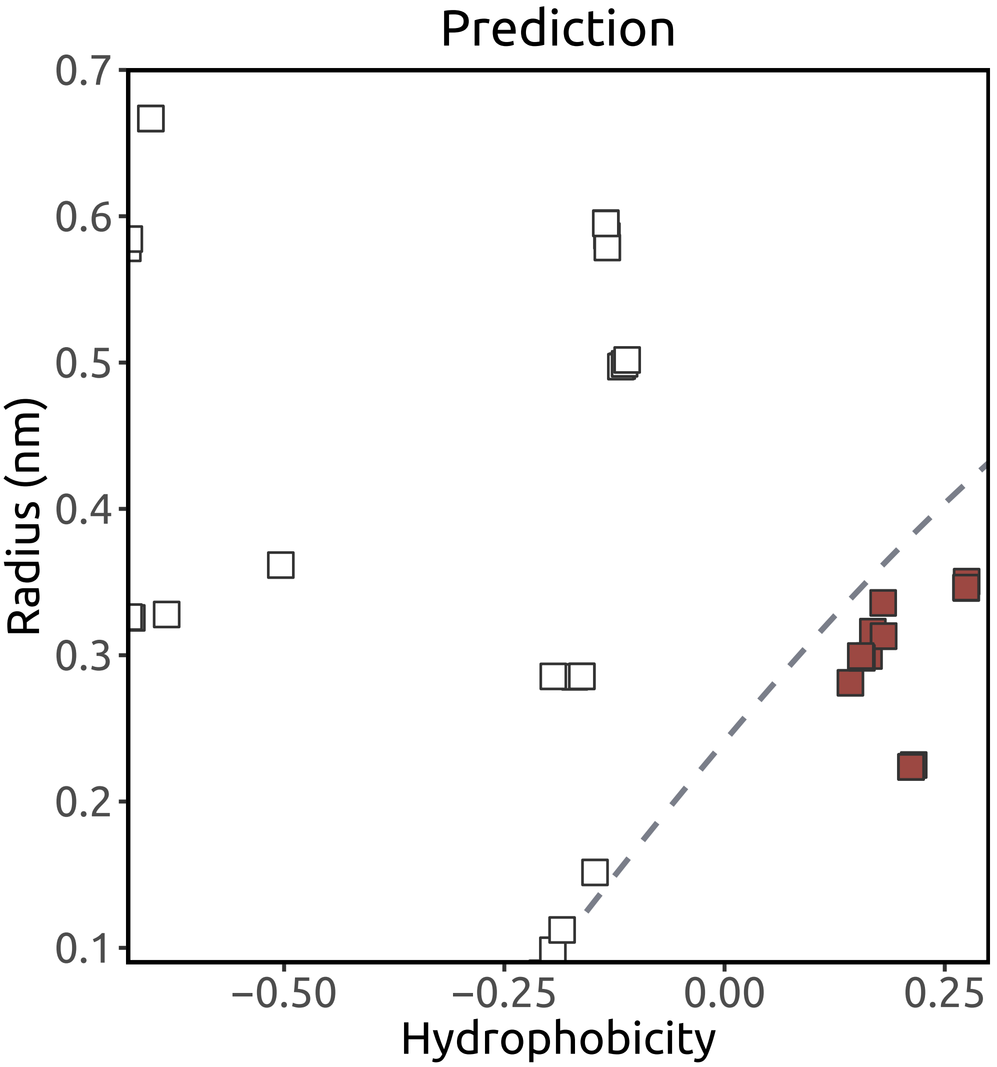


Heuristic score:
1.09 (n = 16)

Simulation result:
hydrated channel

# TRPML1 (PDB ID: 5WPV)

*Mus musculus*
cryo–EM (3.59 Å)

Chen et al., 2017

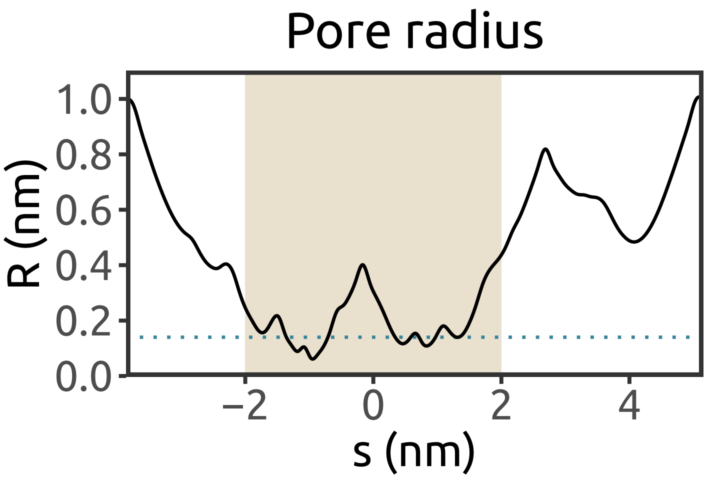


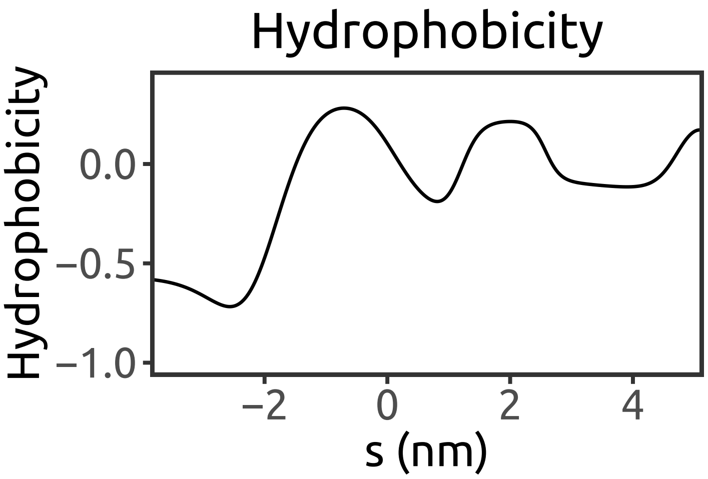


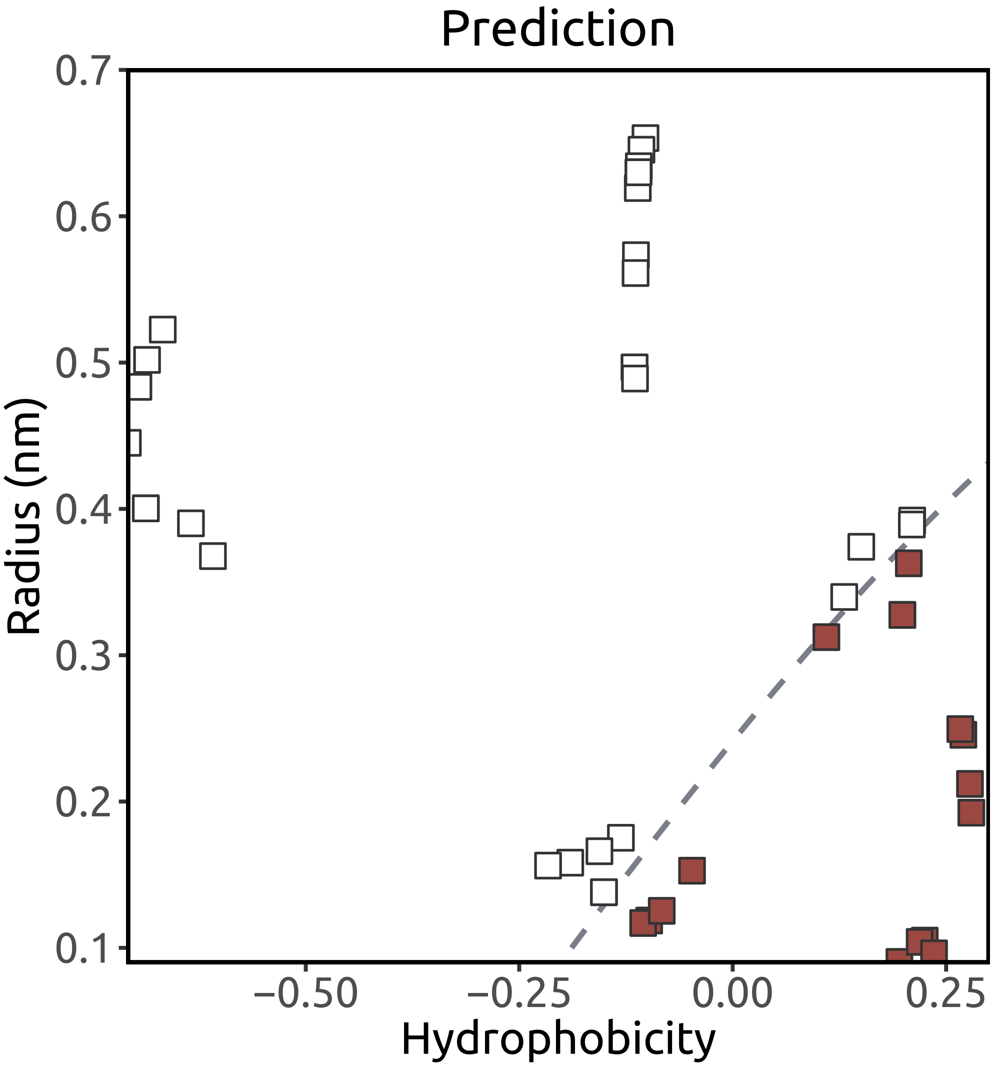


Heuristic score:
1.89 (n = 16)

Simulation result:
barrier to water

# TRPML3 (PDB ID: 5W3S)

*Callithrix jacchus*
cryo−EM (2.94 Å)

Hirschi et al., 2017

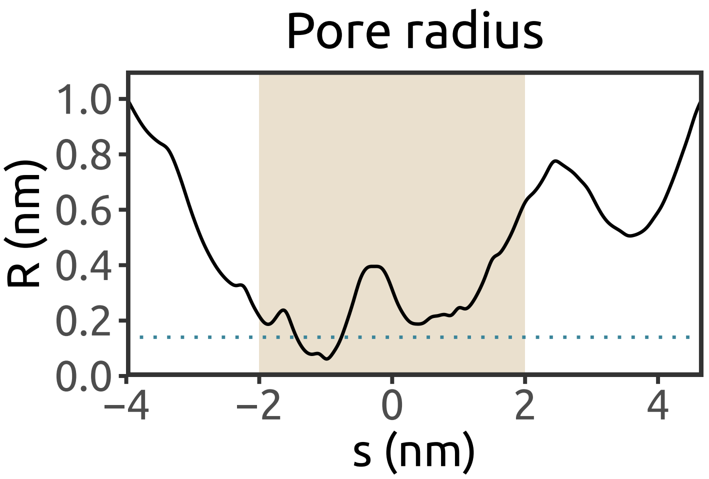


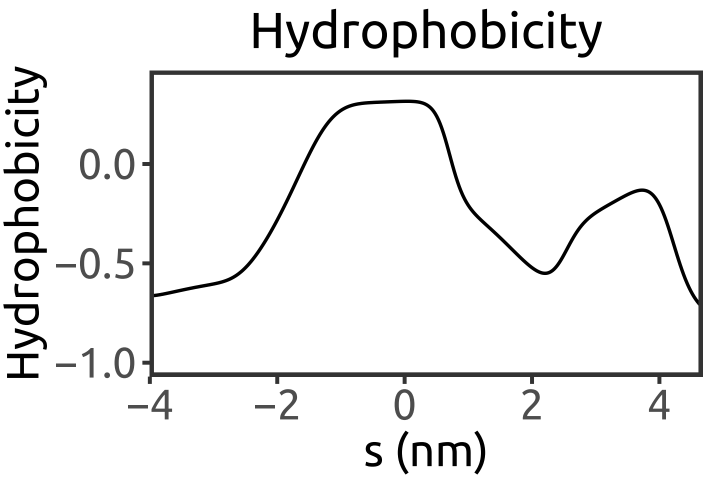


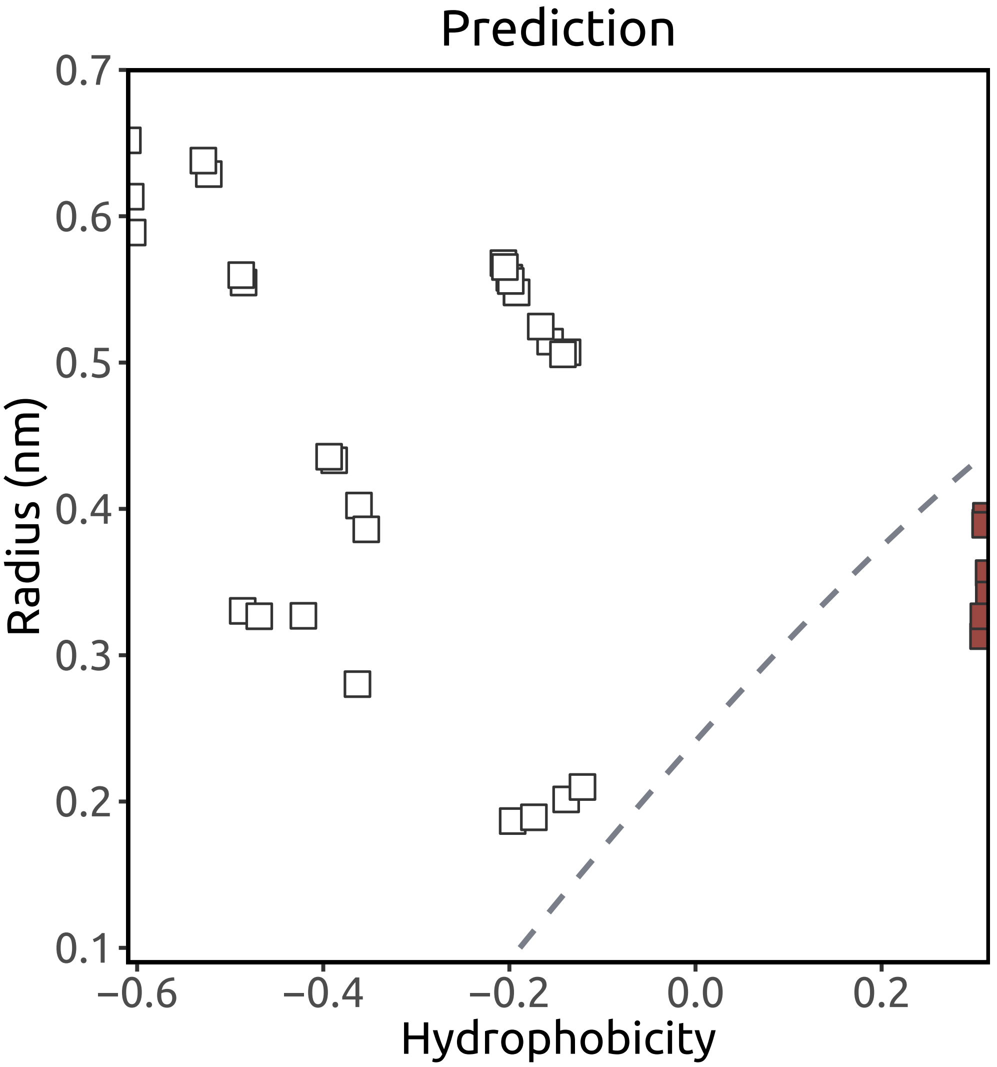


Heuristic score:
1.56 (n = 12)

Simulation result:
barrier to water

# TRPML3 (PDB ID: 6AYE)

*Homo sapiens*
cryo–EM (4.06 Å)

Zhou et al., 2017

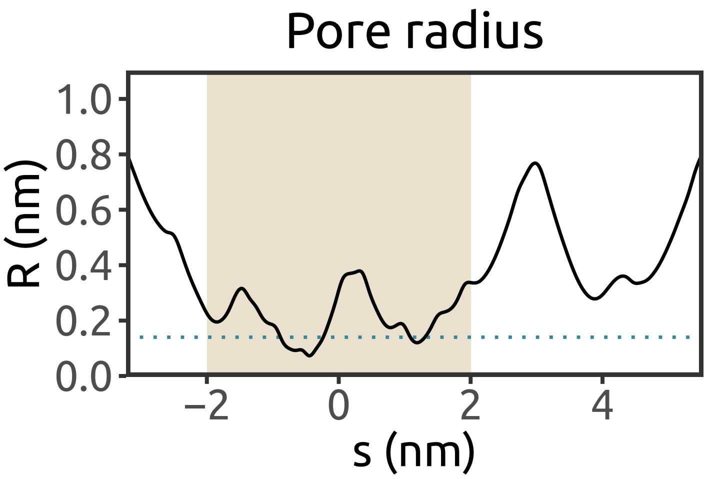


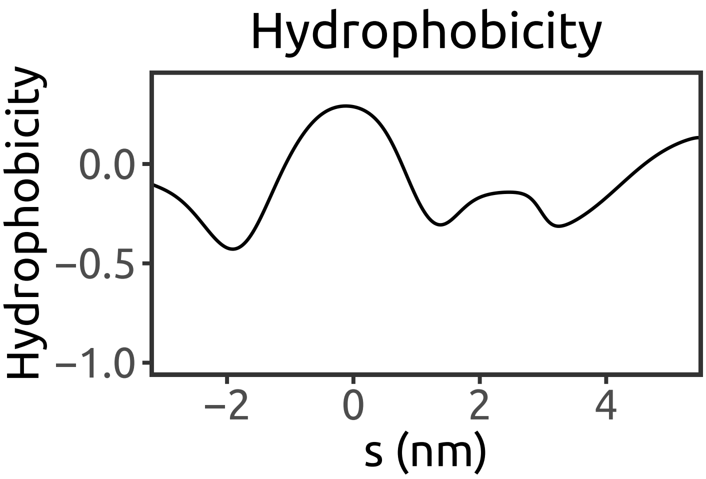


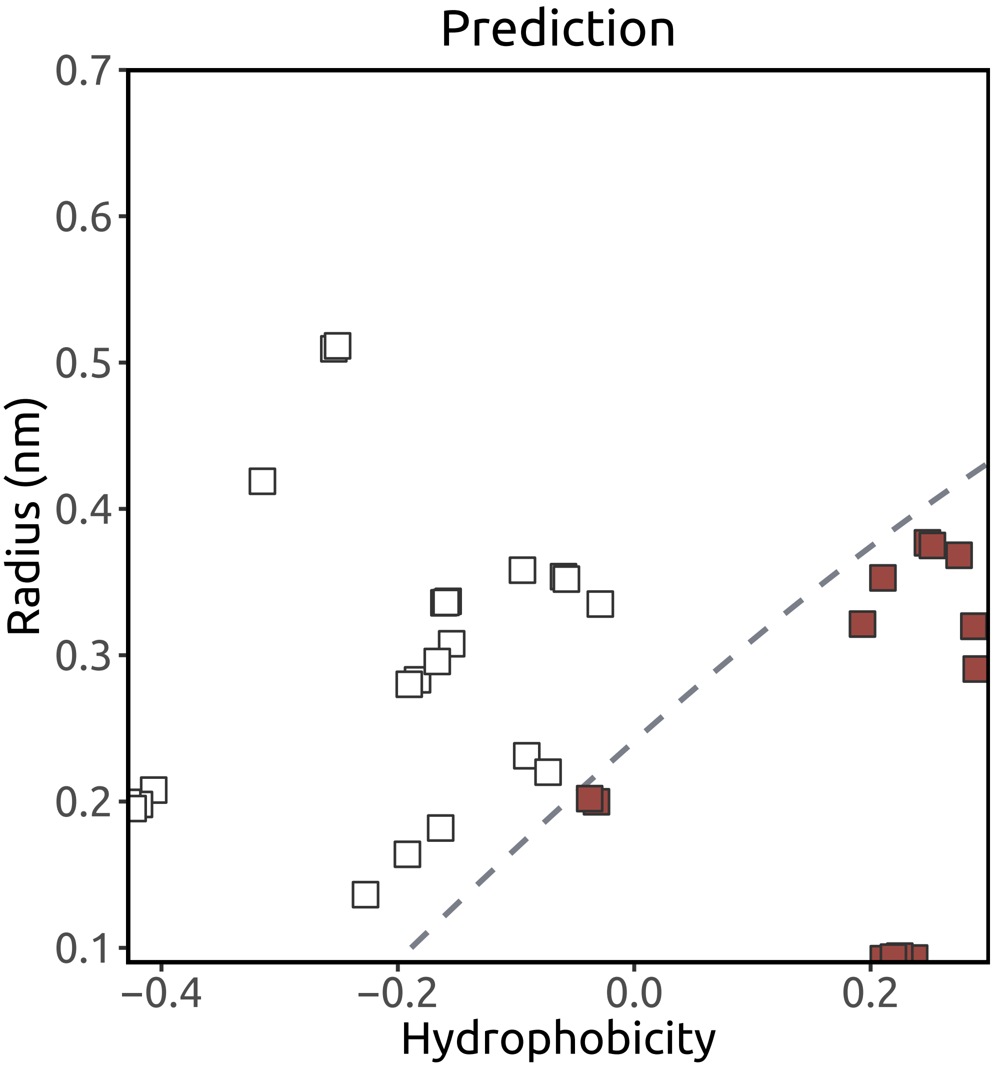


Heuristic score:
1.48 (n = 14)

Simulation result:
barrier to water

# TRPML3 (PDB ID: 6AYF)

*Homo sapiens*
cryo−EM (3.62 Å)

Zhou et al., 2017

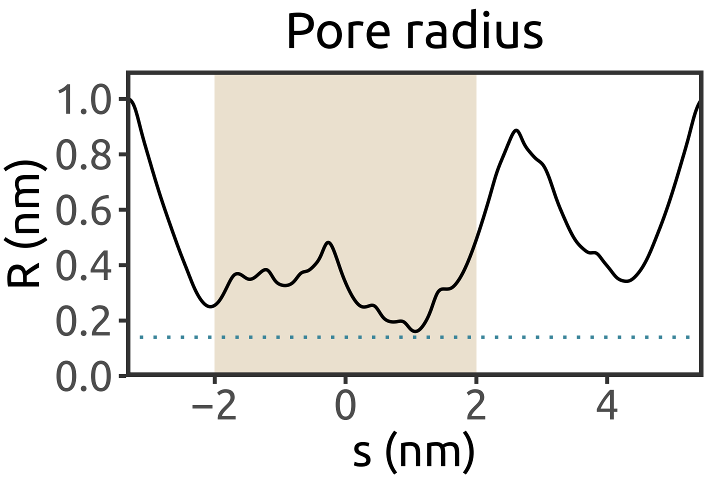


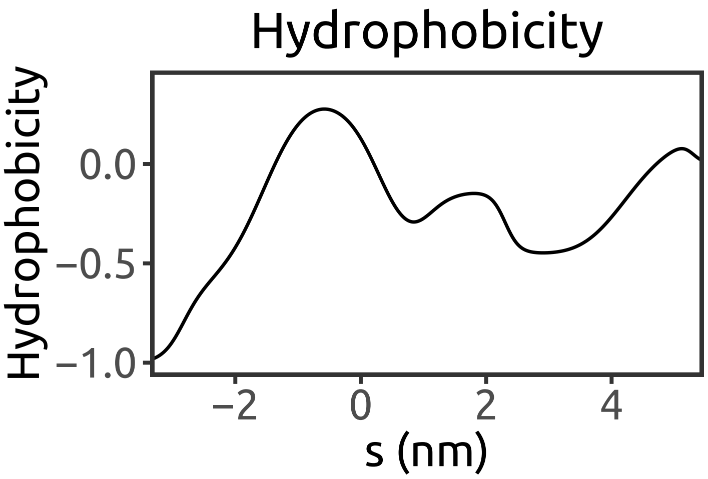


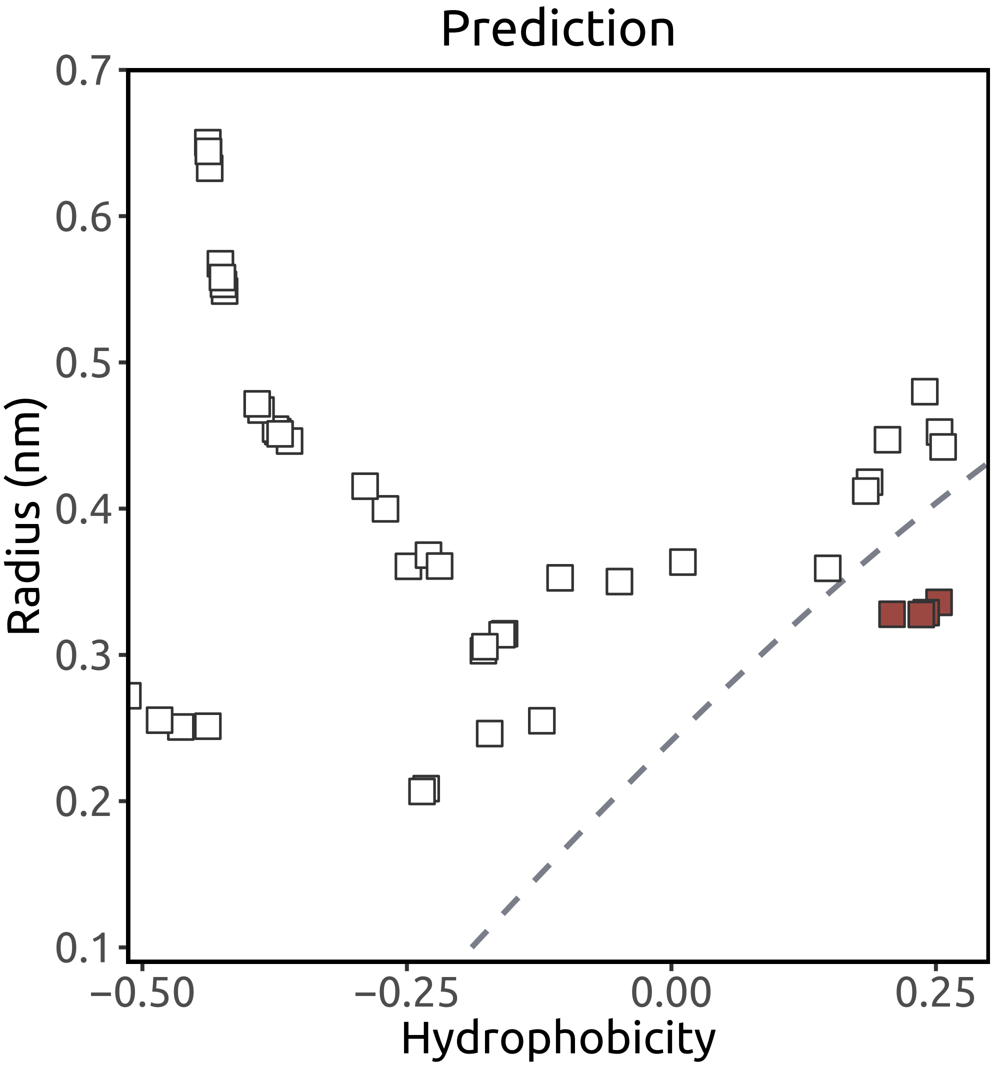


Heuristic score:
0.22 (n = 4)

Simulation result:
hydrated channel

# TRPML3 (PDB ID: 6AYG)

*Homo sapiens*
cryo–EM (4.65 Å)

Zhou et al., 2017

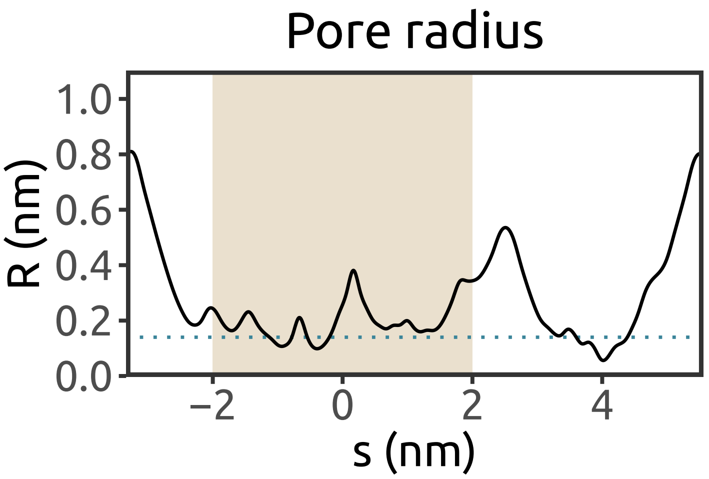


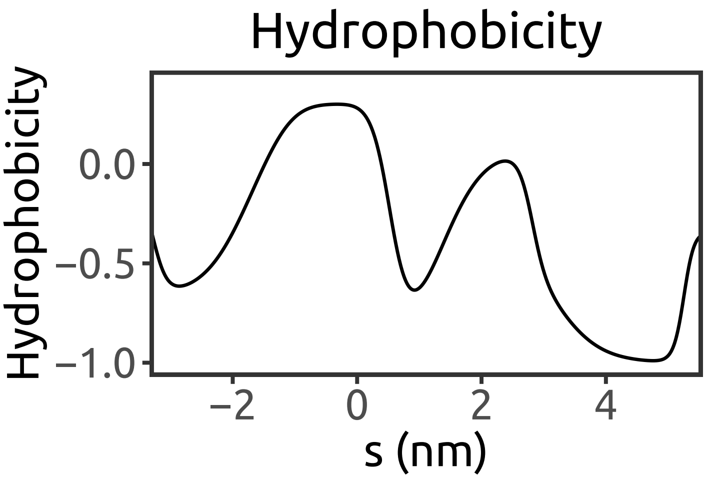


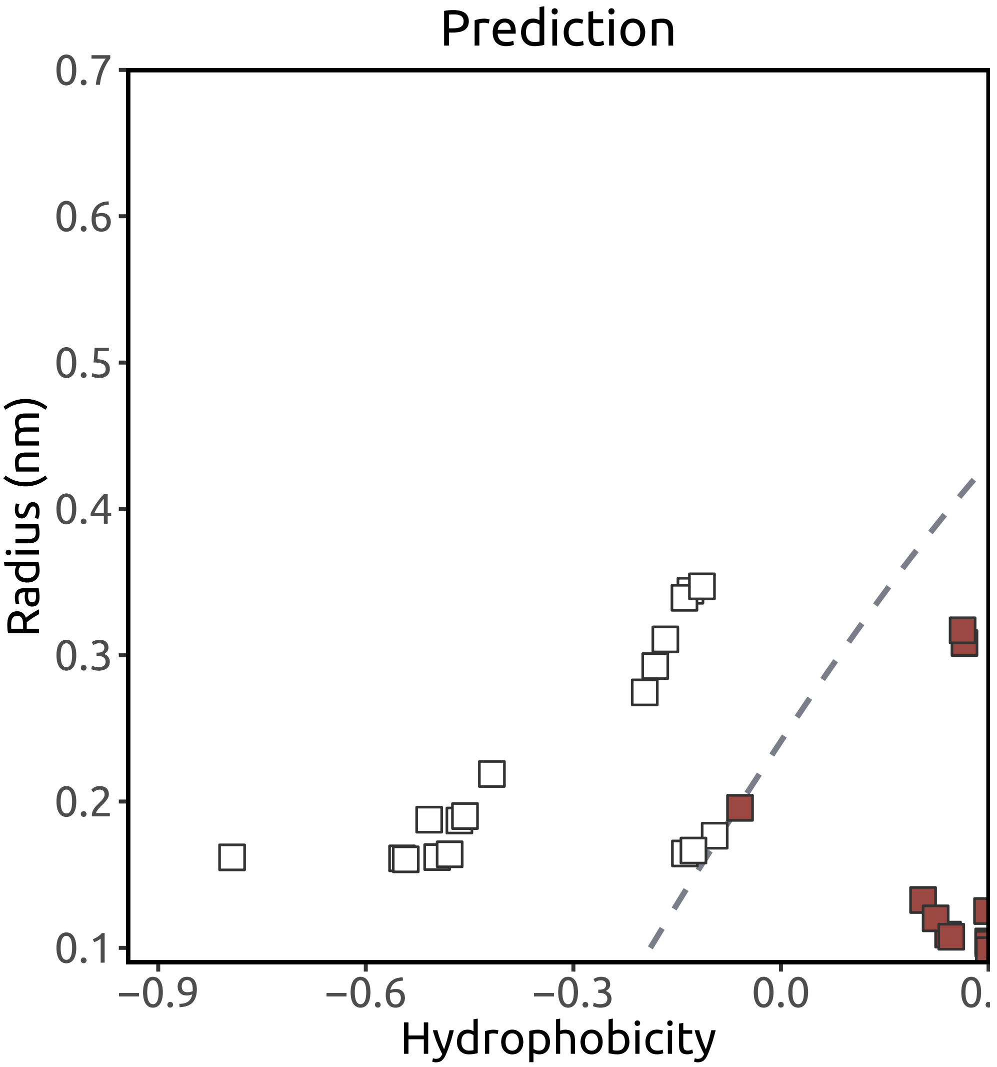


Heuristic score:
2.22 (n = 11)

Simulation result:
barrier to water

# TRPV1 (PDB ID: 3J5P)

*Rattus norvegicus*
cryo–EM (3.28 Å)

Liao et al., 2013

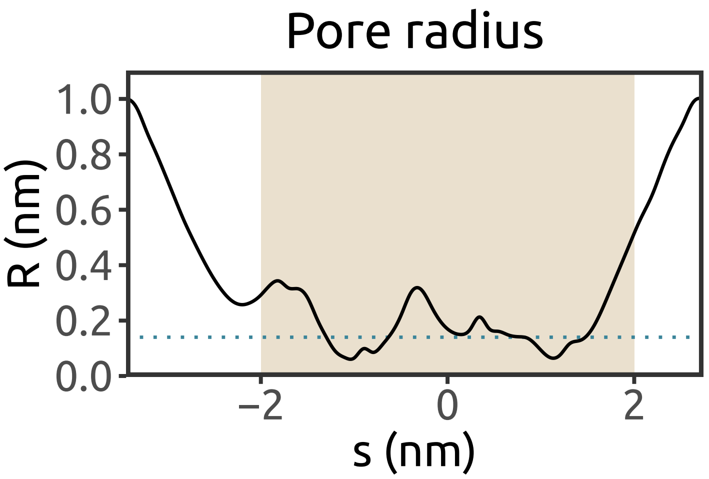


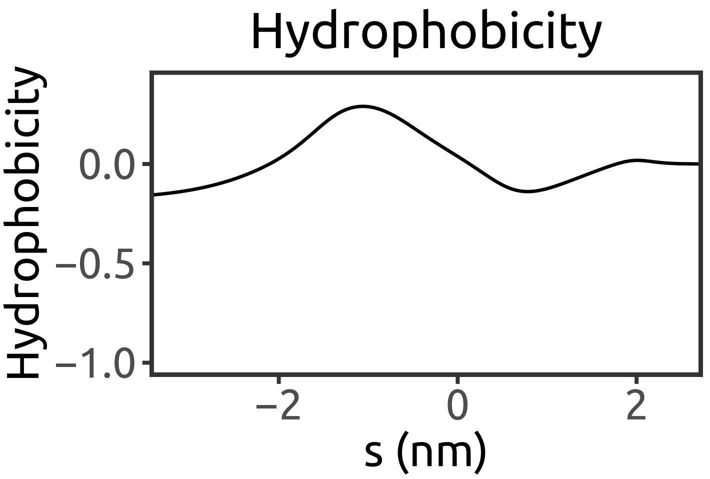


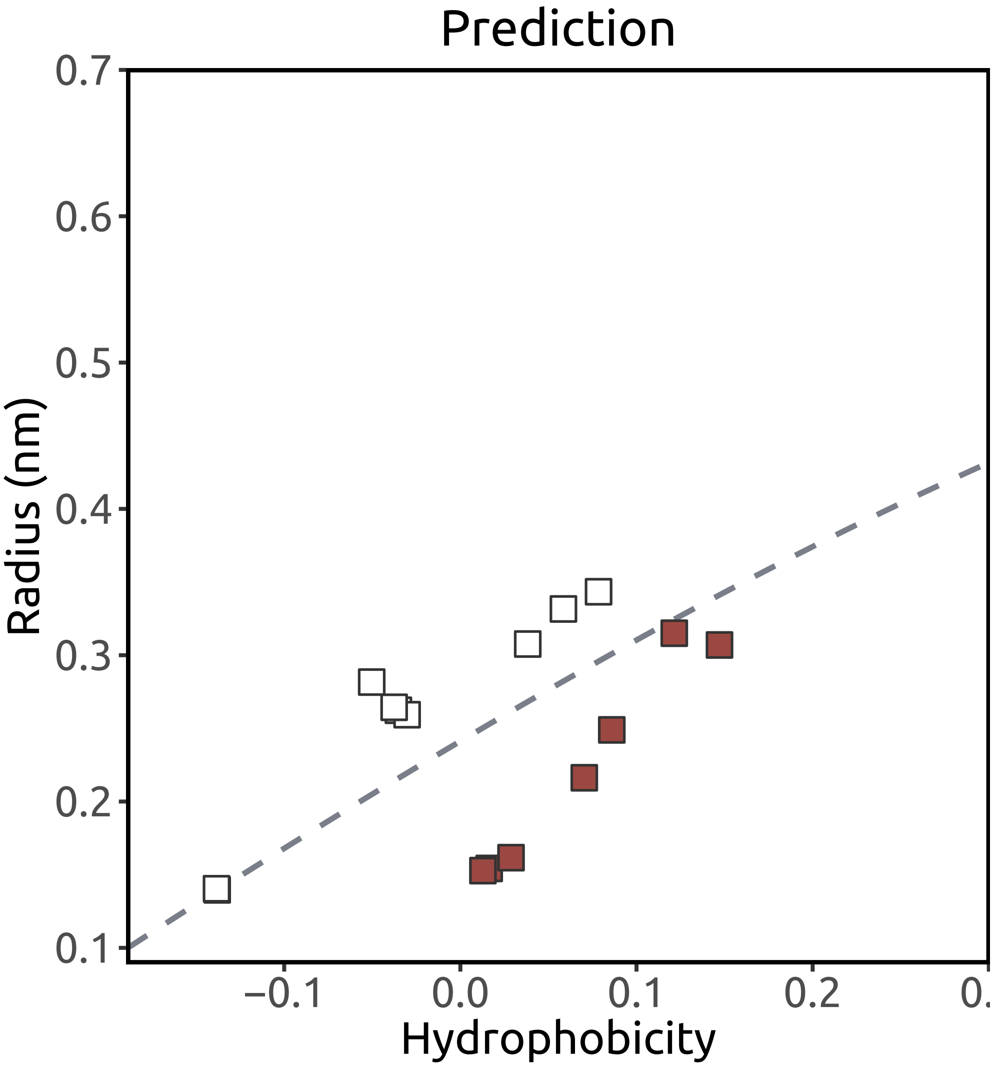


Heuristic score:
1.74 (n = 13)

Simulation result:
barrier to water

# TRPV1 (PDB ID: 3J5Q)

*Rattus norvegicus*
cryo−EM (3.8 Å)

Cao et al., 2013

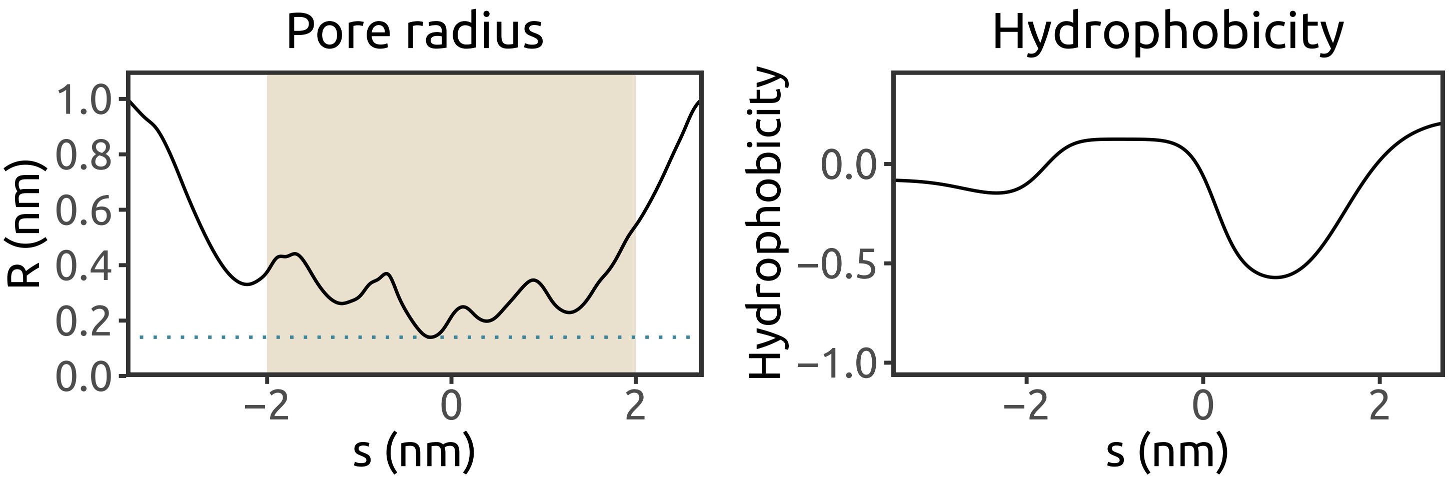


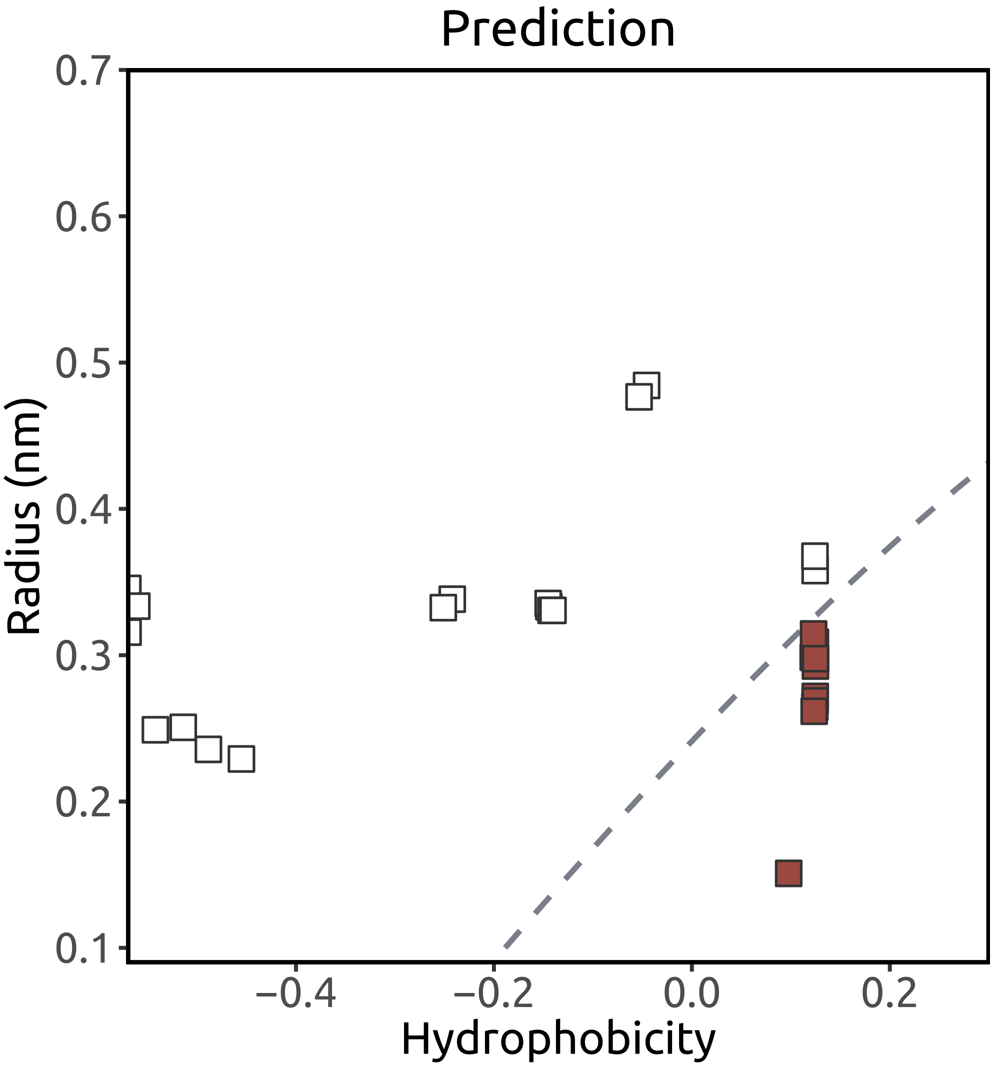


Heuristic score:
0.45 (n = 11)

Simulation result:
barrier to water

# TRPV1 (PDB ID: 3J5R)

*Rattus norvegicus*
cryo−EM (4.2 Å)

Cao et al., 2013

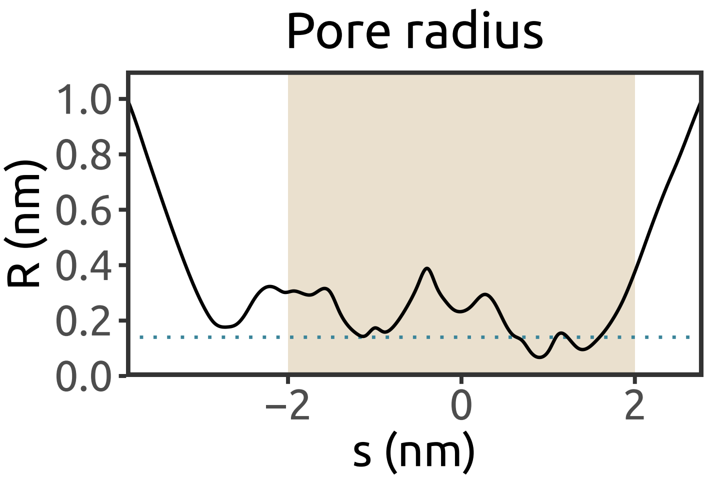


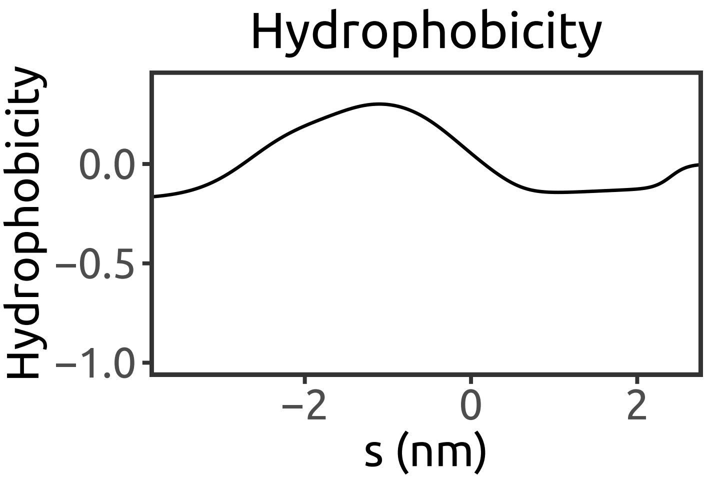


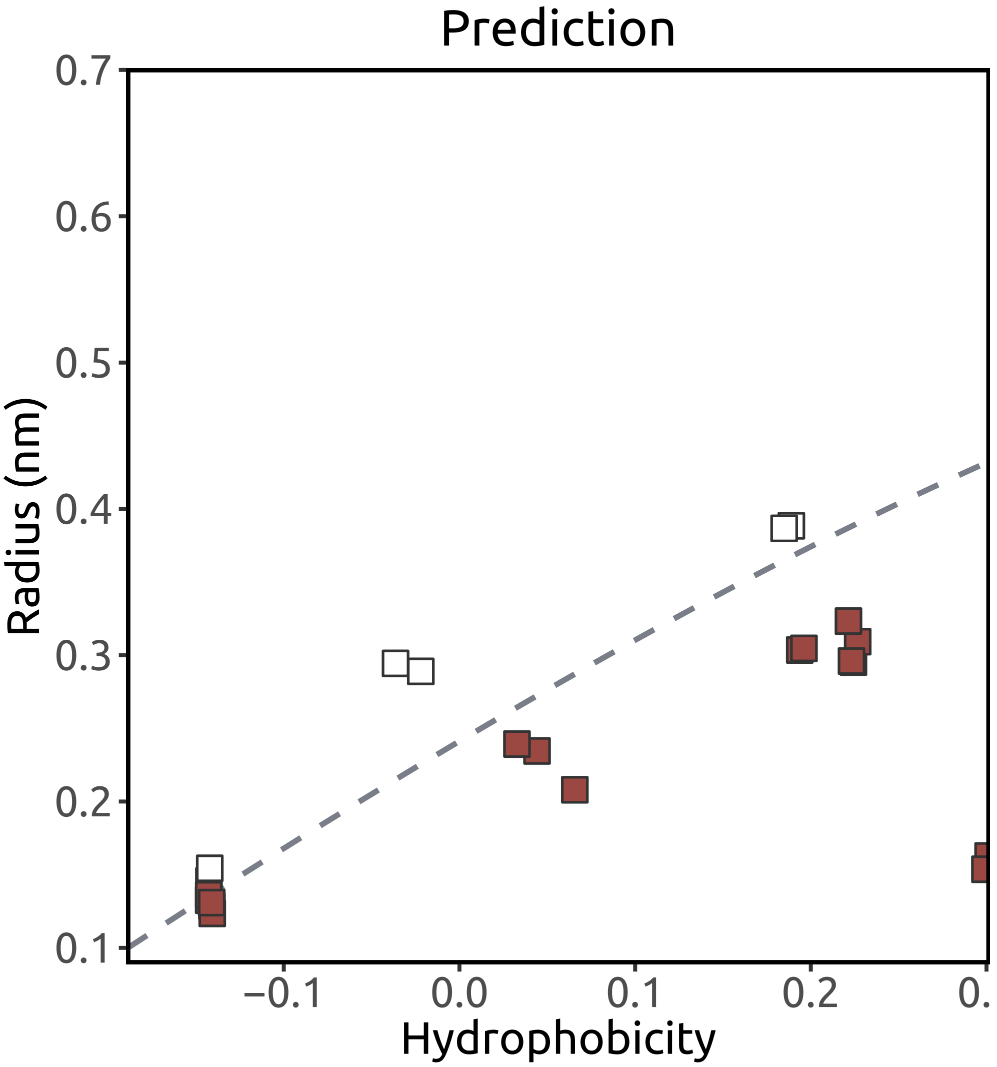


Heuristic score:
1.49 (n = 19)

Simulation result:
barrier to water

# TRPV1 (PDB ID: 5IRX)

*Rattus norvegicus*
cryo–EM (2.95 Å)

Gao et al., 2015

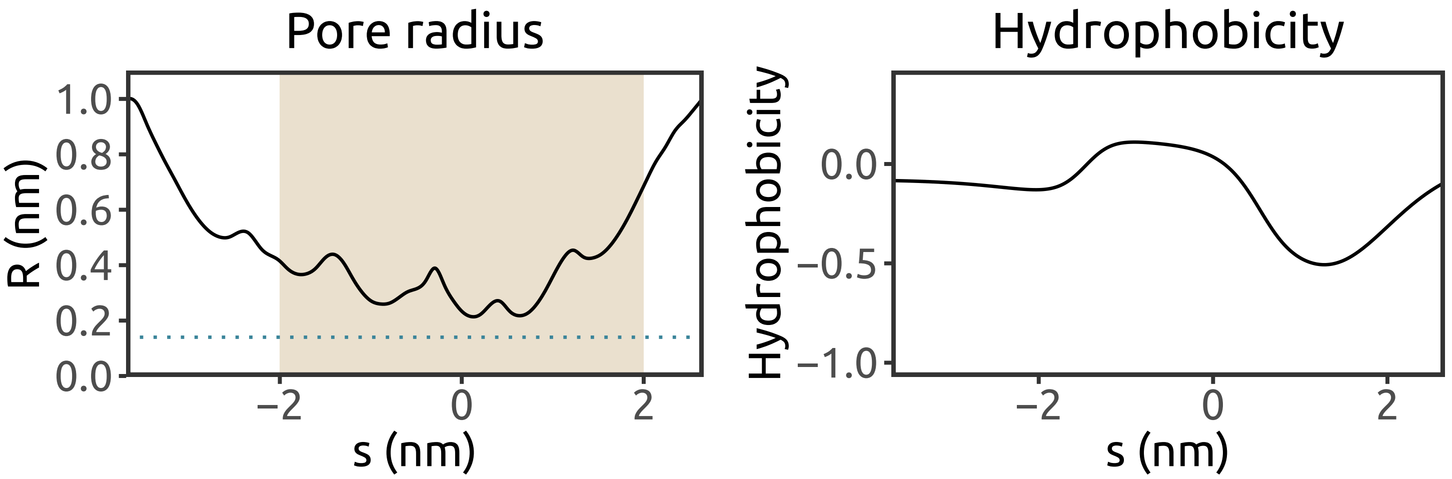


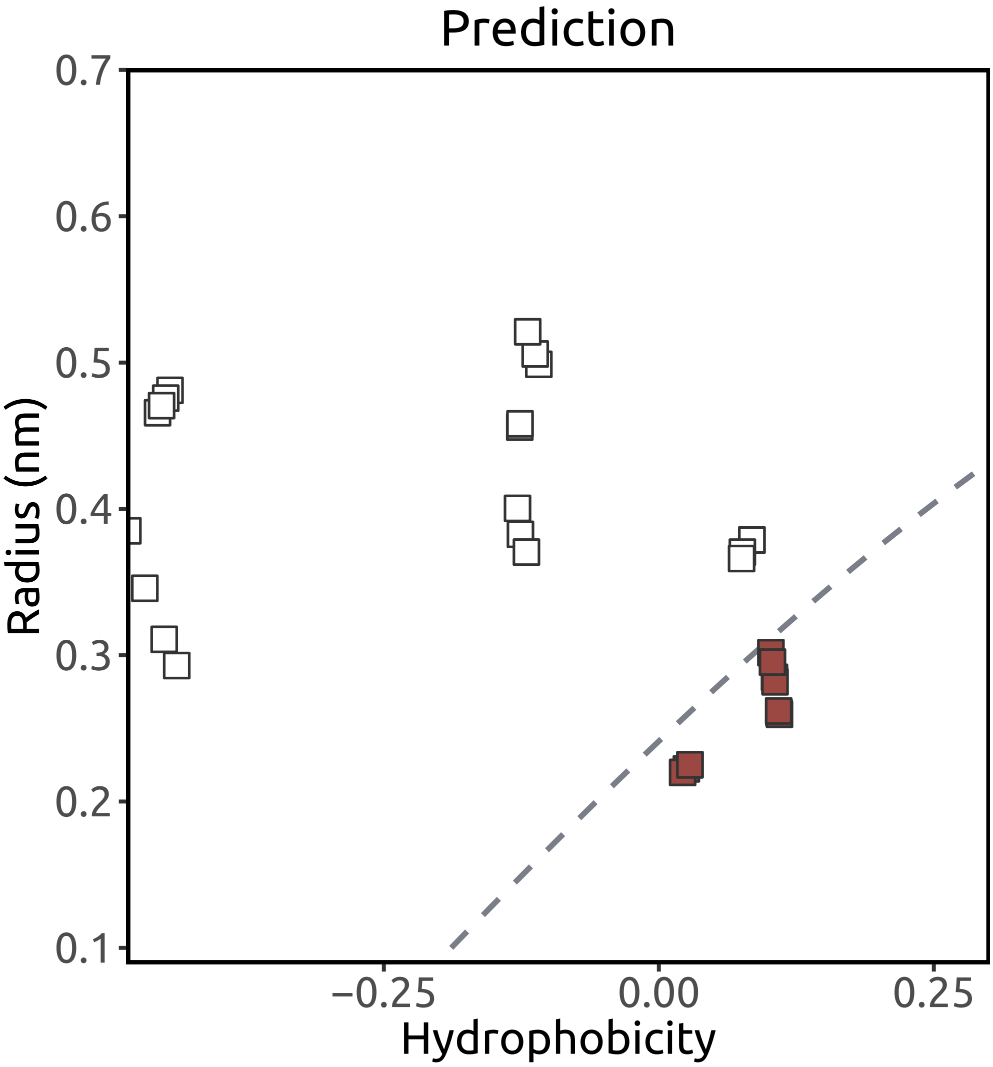


Heuristic score:
0.38 (n = 12)

Simulation result:
hydrated channel

# TRPV1 (PDB ID: 5IRZ)

*Rattus norvegicus*
cryo−EM (3.28 Å)

Gao et al., 2015

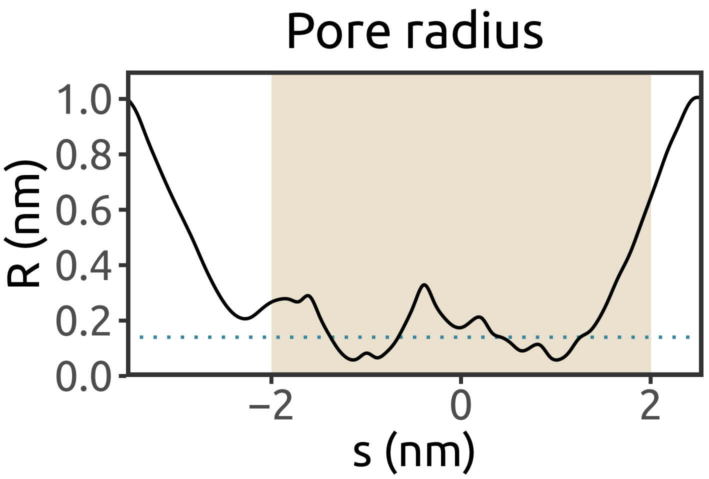


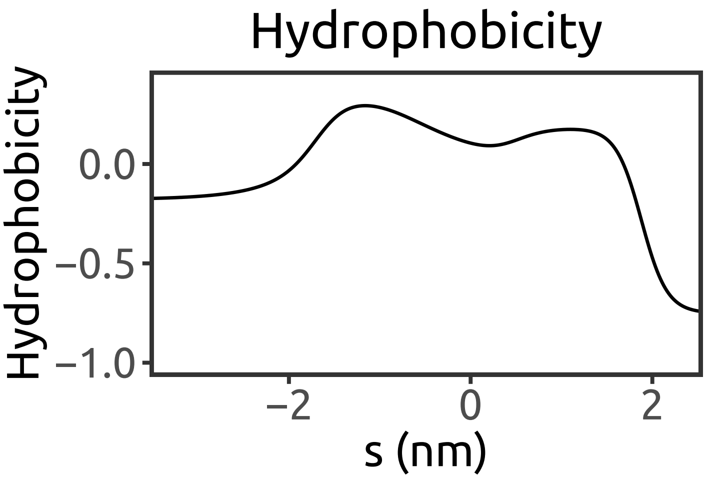


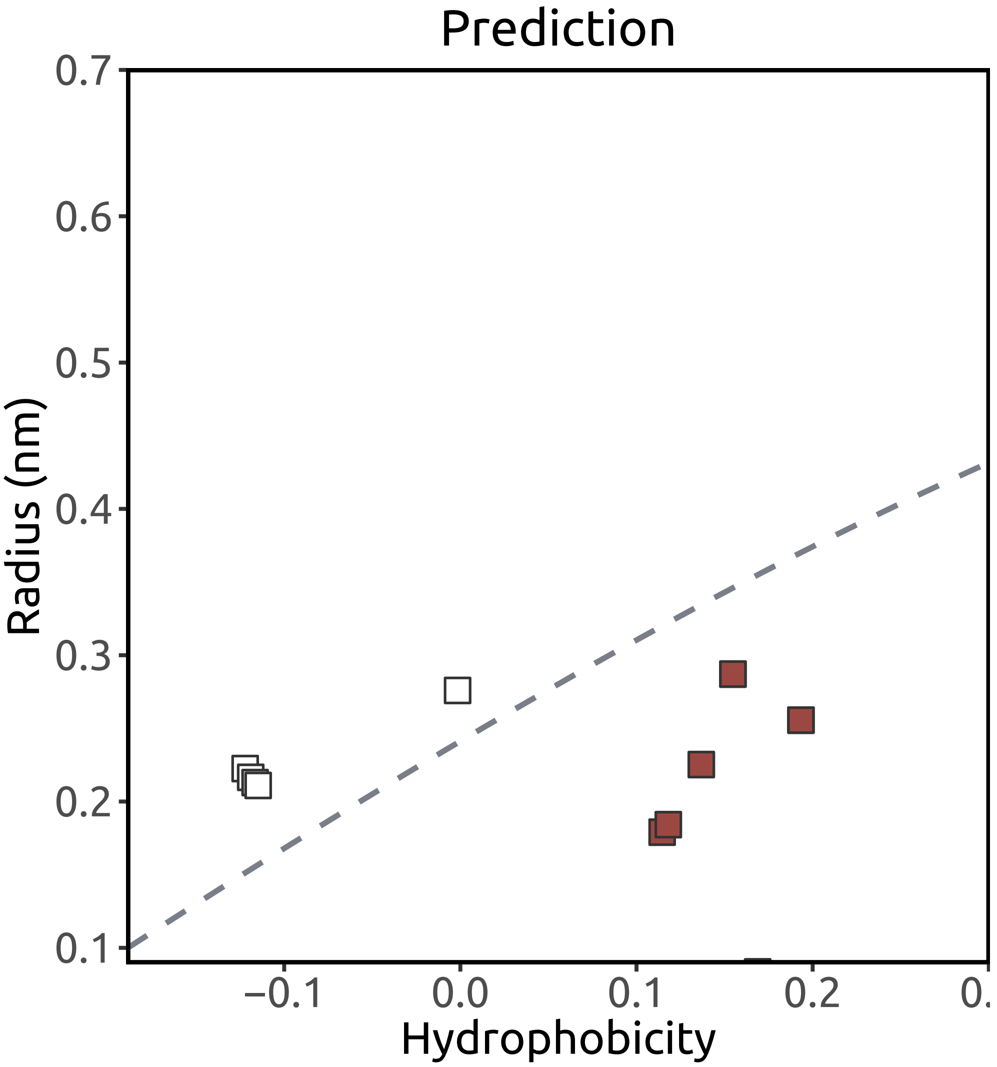


Heuristic score:
2.79 (n = 15)

Simulation result:
barrier to water

# TRPV1 (PDB ID: 5IS0)

*Rattus norvegicus*
cryo−EM (3.43 Å)

Gao et al., 2015

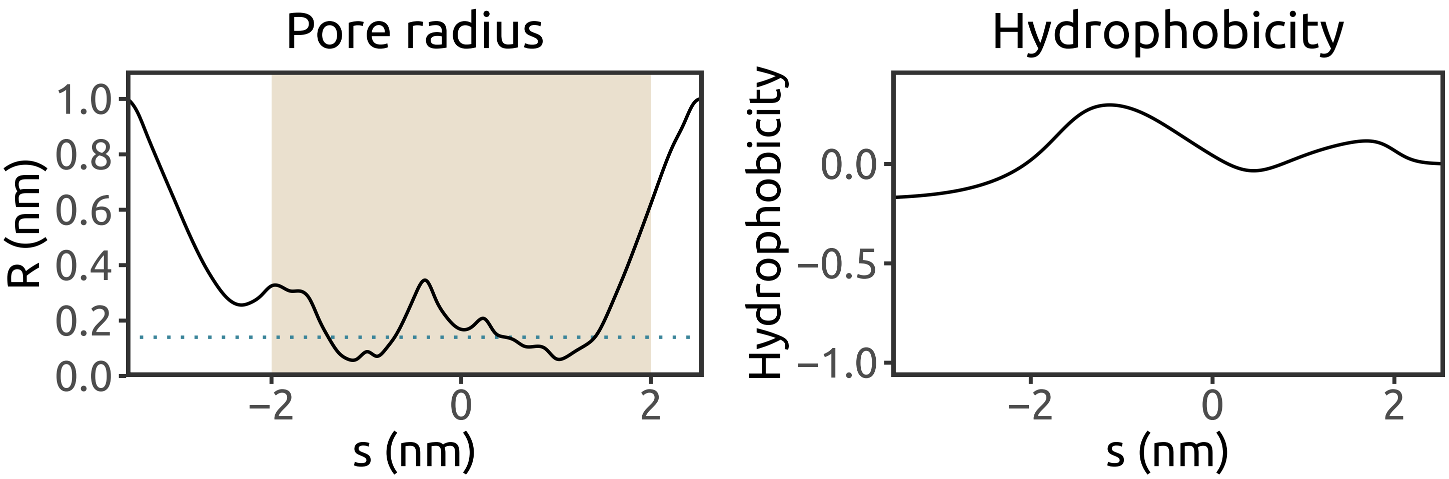


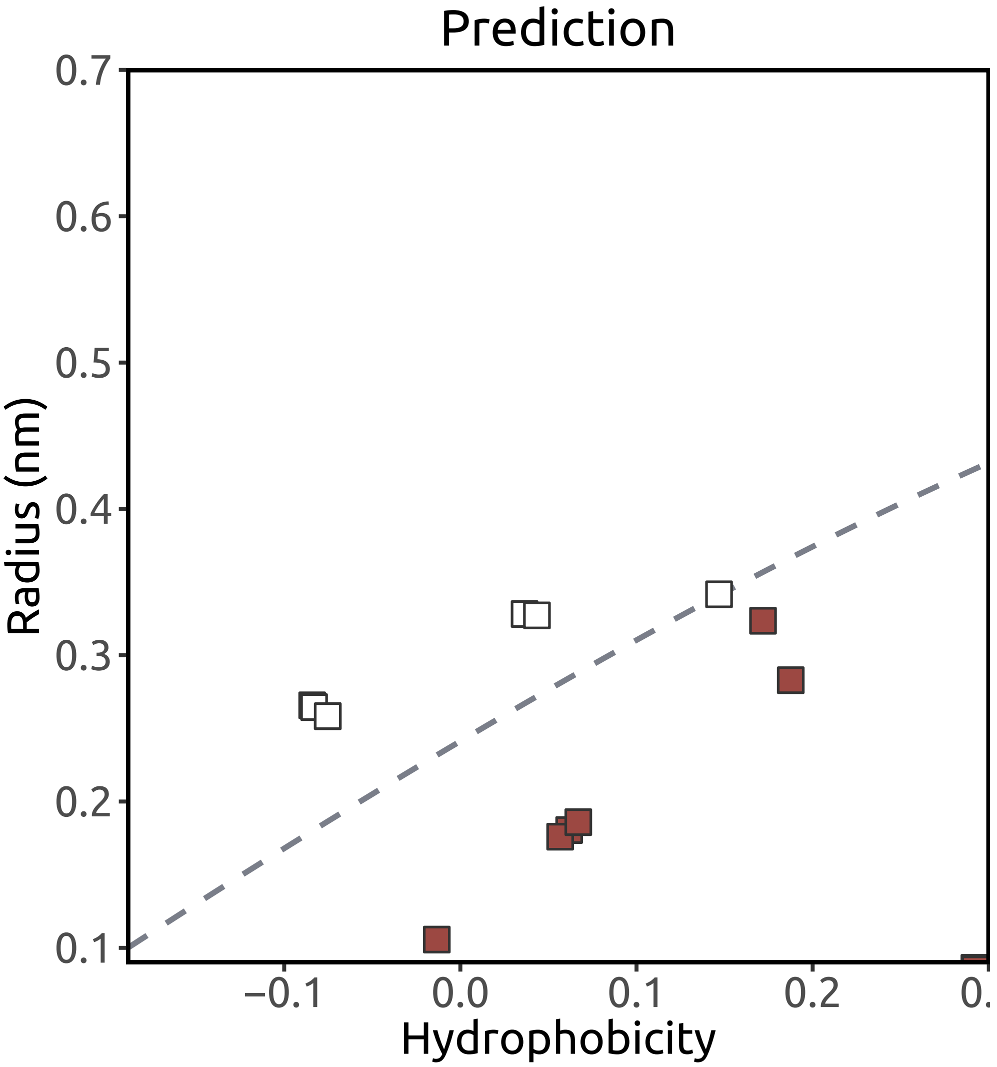


Heuristic score:
2.16 (n = 13)

Simulation result:
barrier to water

# TRPV2 (PDB ID: 5AN8)

*Oryctolagus cuniculus*
cryo−EM (3.8 Å)

Zubcevic et al., 2016

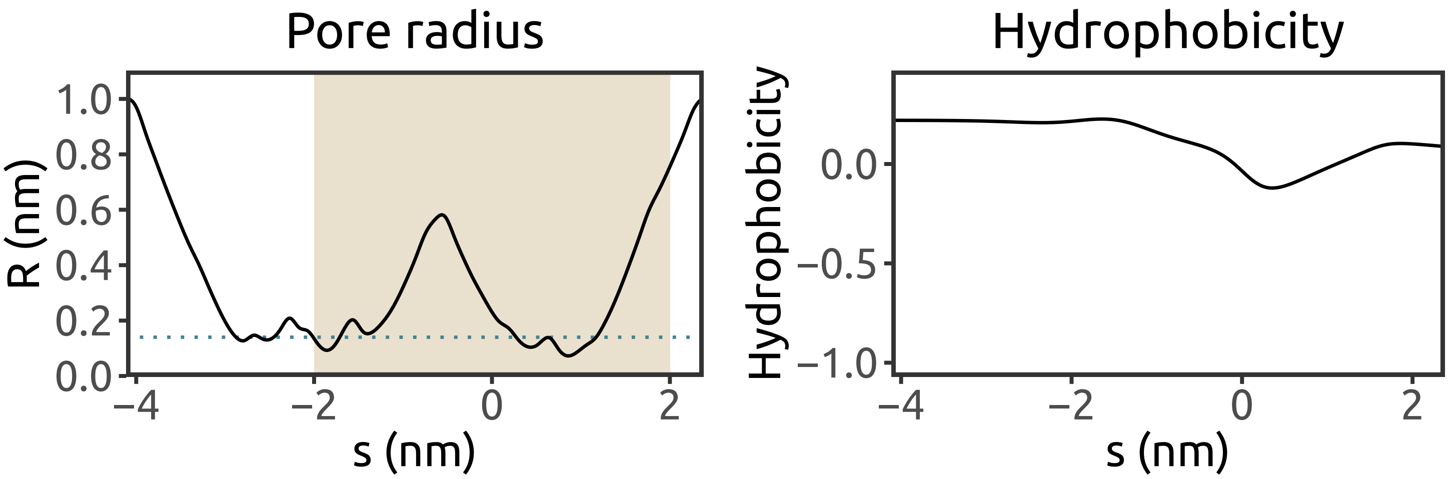


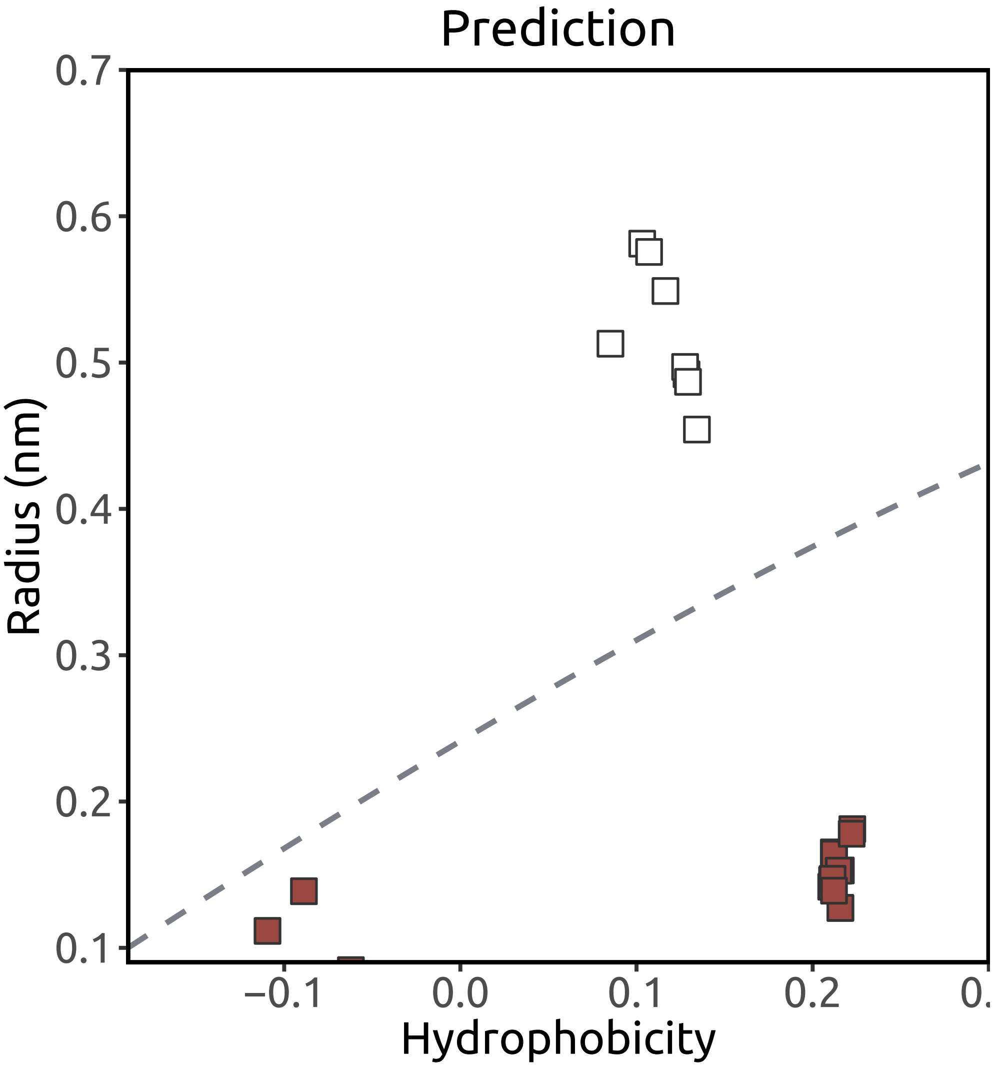


Heuristic score:
2.72 (n = 17)

Simulation result:
barrier to water

# TRPV2 (PDB ID: 5HI9)

*Rattus norvegicus*
cryo–EM (4.4 Å)

Huynh et al., 2016

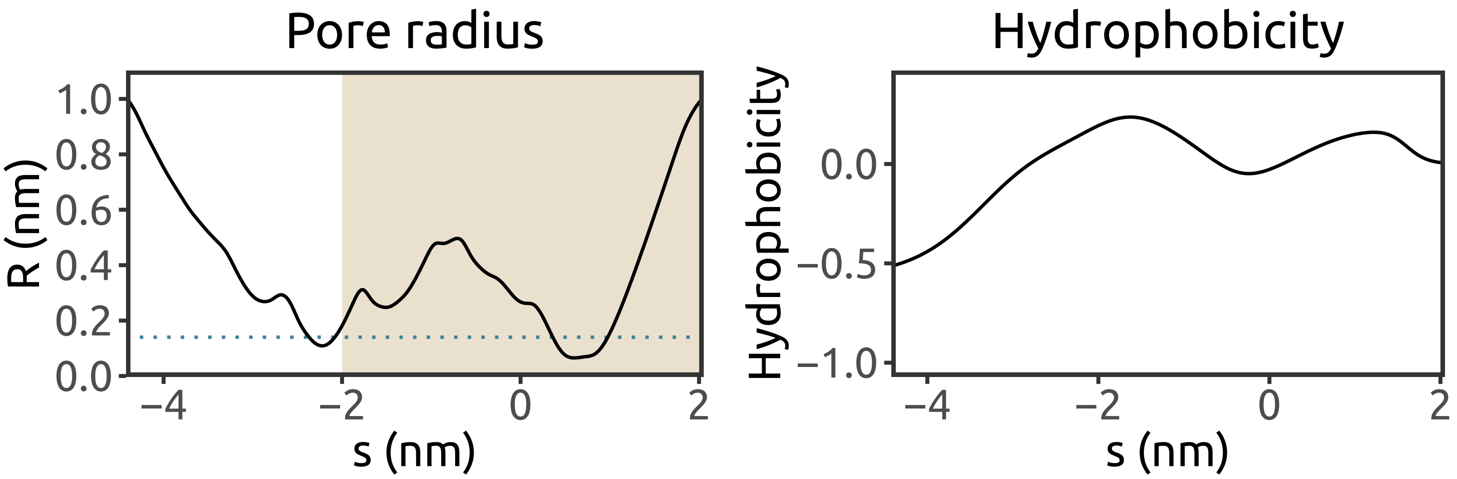


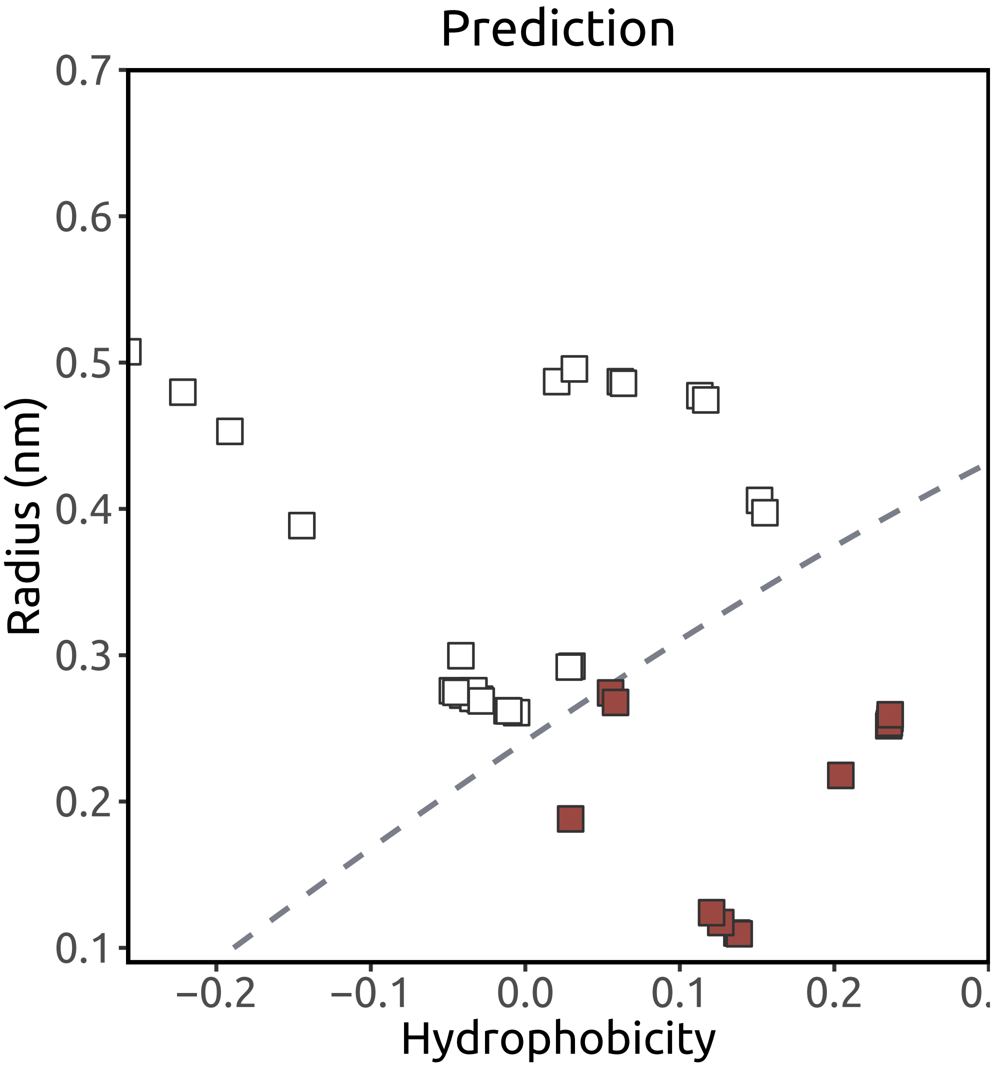


Heuristic score:
2.17 (n = 16)

Simulation result:
barrier to water

# TRPV2 (PDB ID: 6BWJ)

*Oryctolagus cuniculus*
X–ray (3.1 Å)

Zubcevic et al., 2018

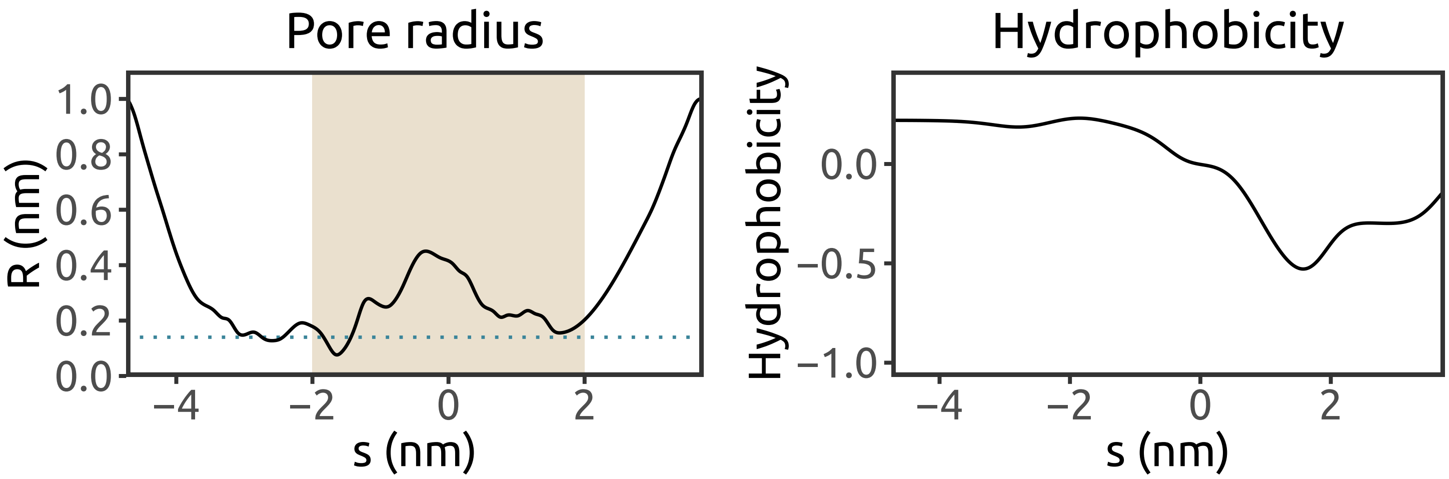


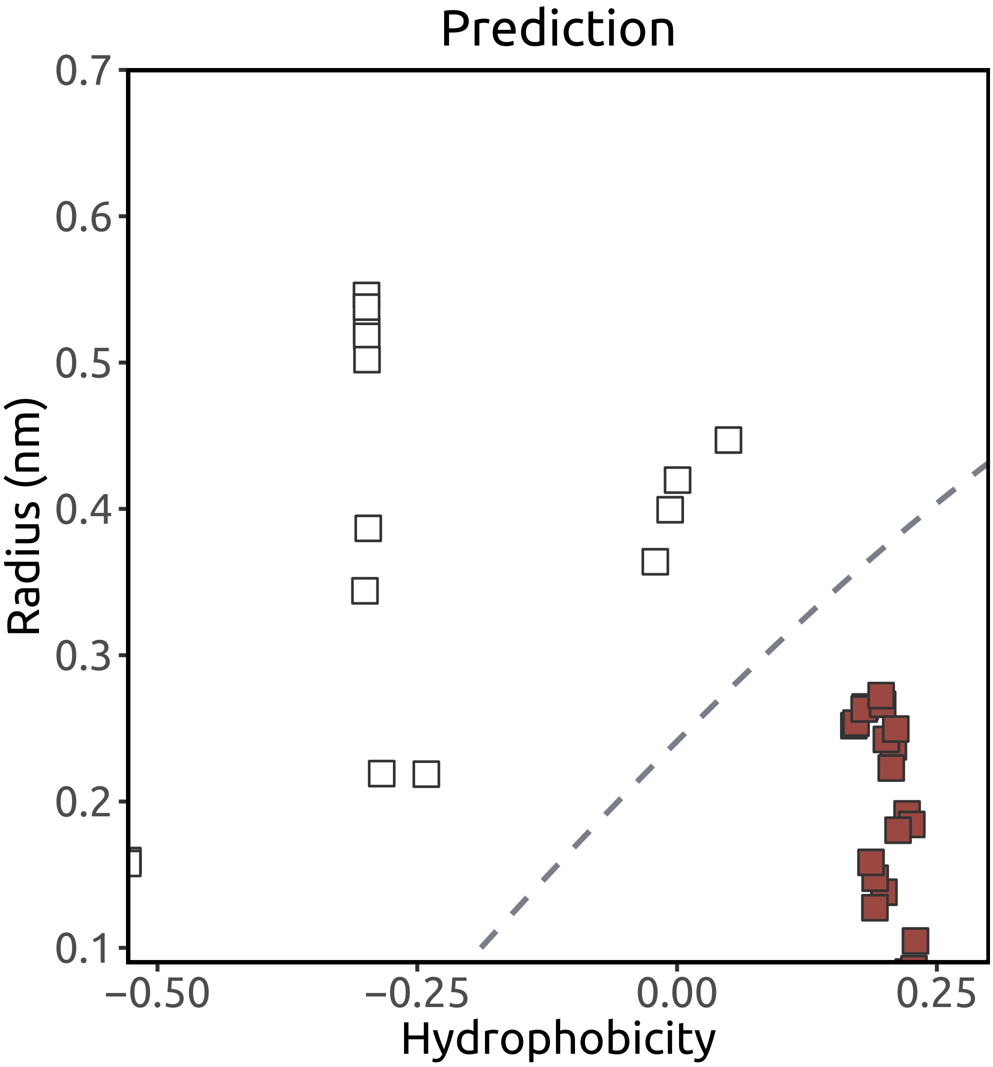


Heuristic score:
3.02 (n = 20)

Simulation result:
barrier to water

# TRPV4 (PDB ID: 6BBJ)

*Xenopus tropicalis*
cryo–EM (3.8 Å)

Deng et al., 2018

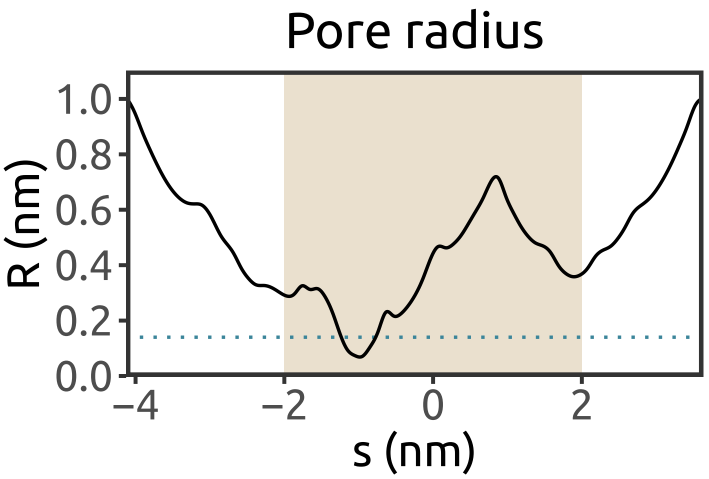


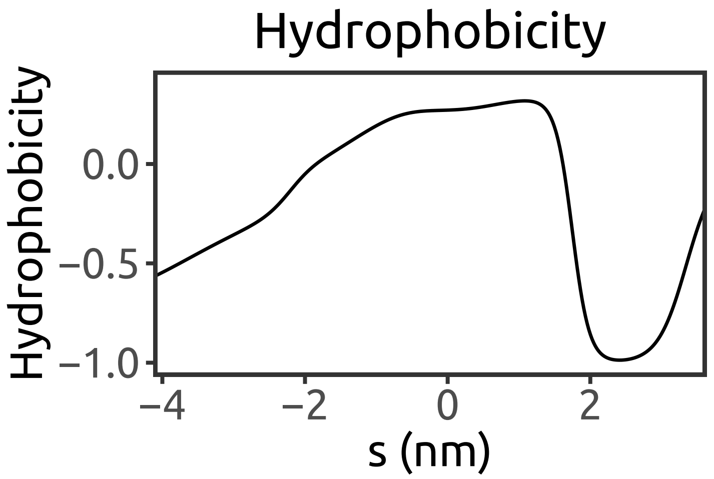


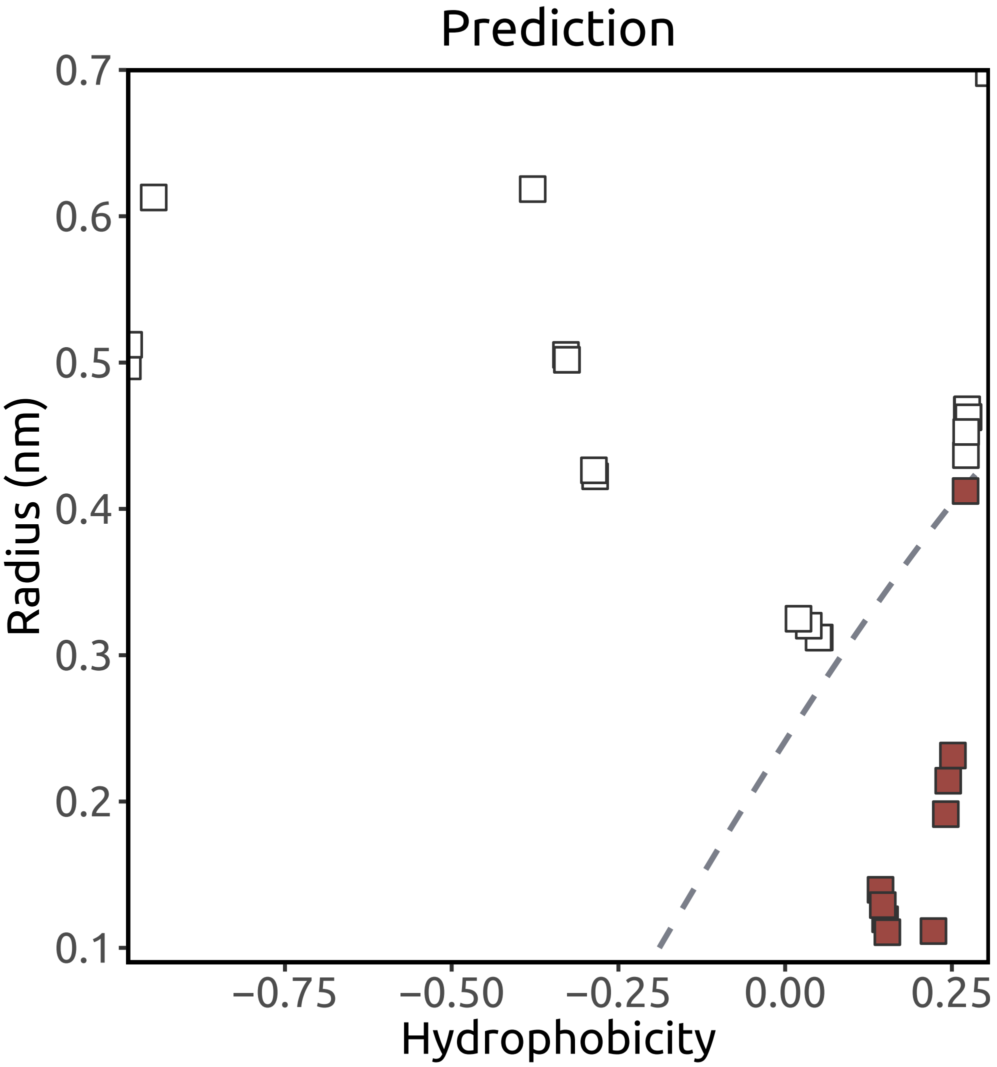


Heuristic score:
1.44 (n = 9)

Simulation result:
barrier to water

# TRPV5 (PDB ID: 6B5V)

*Oryctolagus cuniculus*

cryo–EM (4.8 Å)

Hughes et al., 2018

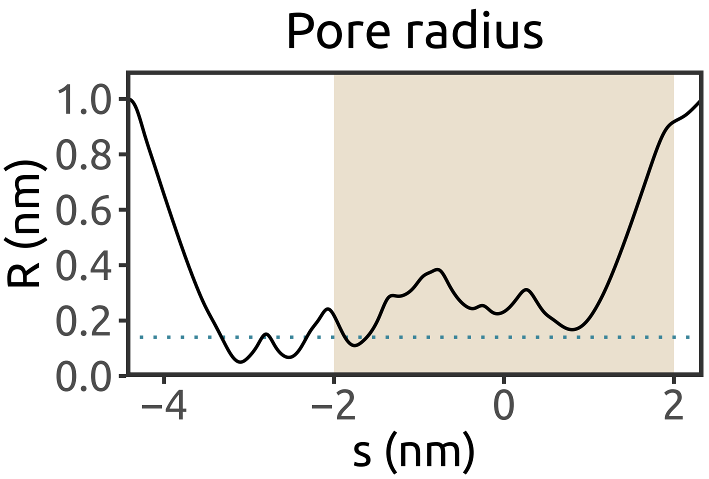


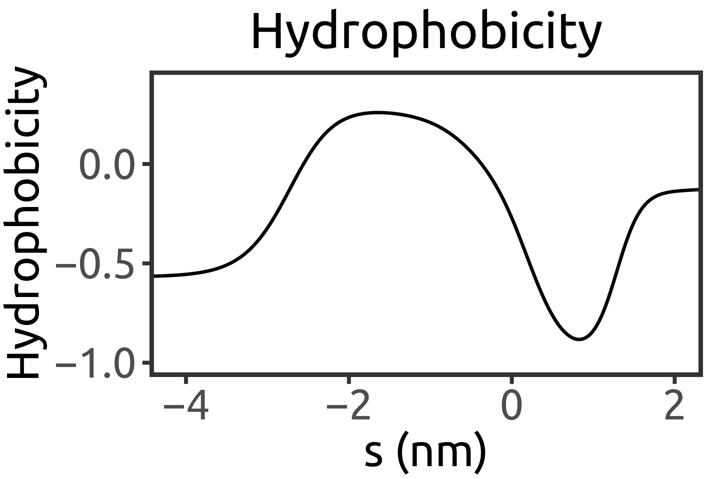


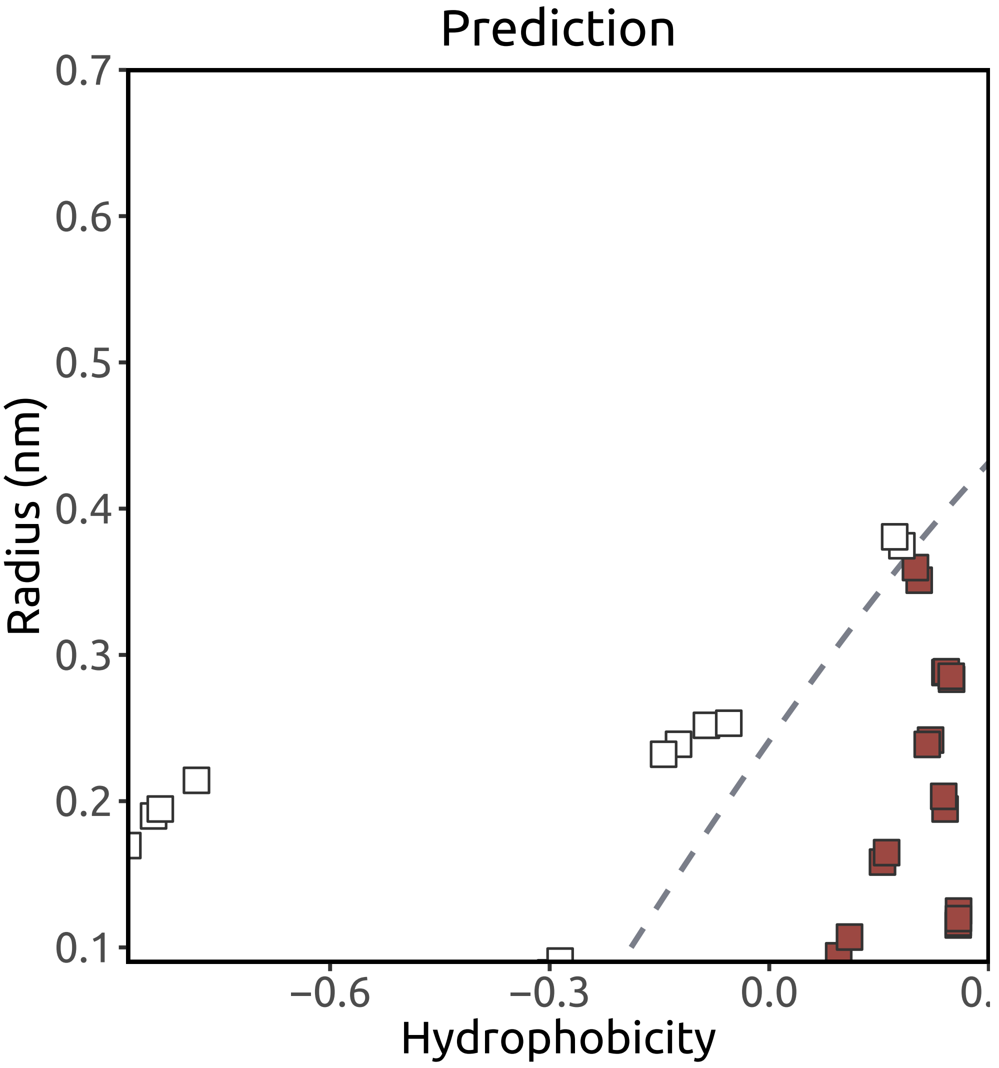


Heuristic score:

2.66 (n = 18)

Simulation result:

barrier to water

# TRPV6 (PDB ID: 5IWK)

*Rattus norvegicus*
X–ray (3.25 Å)

Saotome et al., 2016

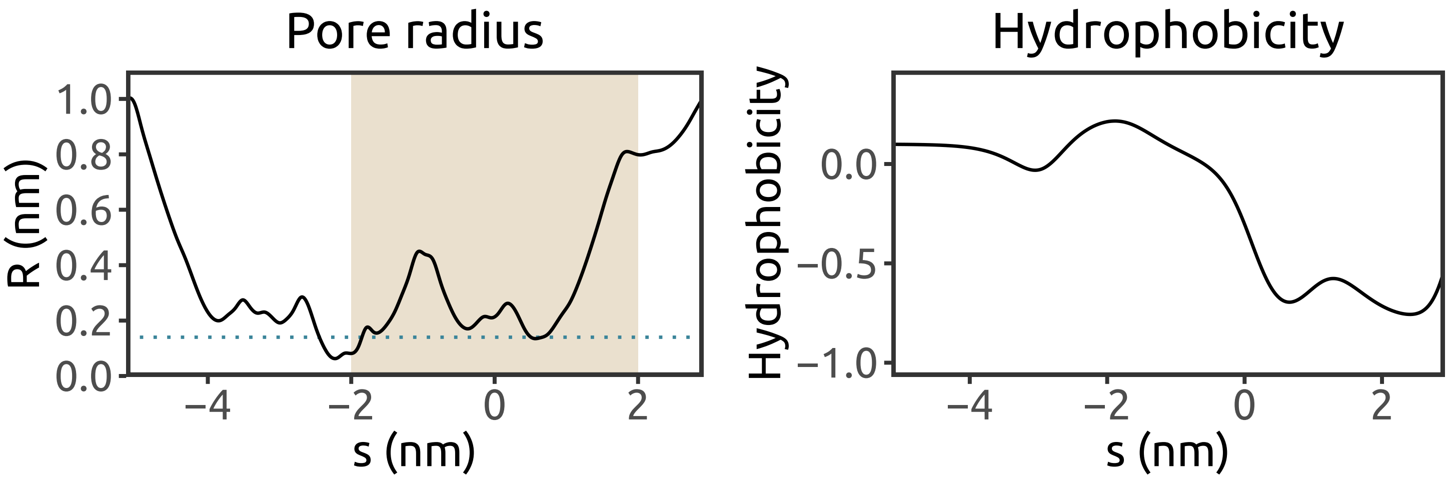


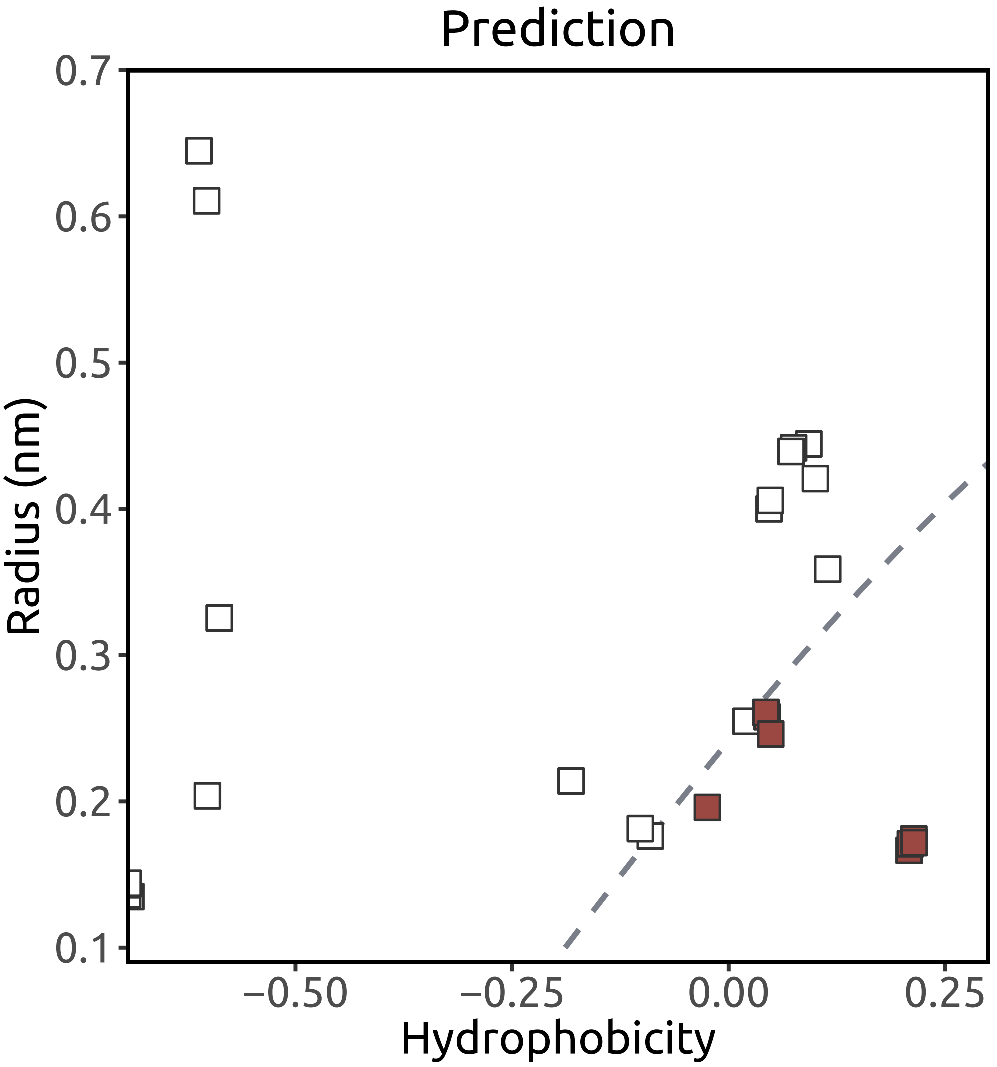


Heuristic score:
1.71 (n = 12)

Simulation result:
barrier to water

# TRPV6 (PDB ID: 5IWP)

*Rattus norvegicus*
X–ray (3.65 Å)

Saotome et al., 2016

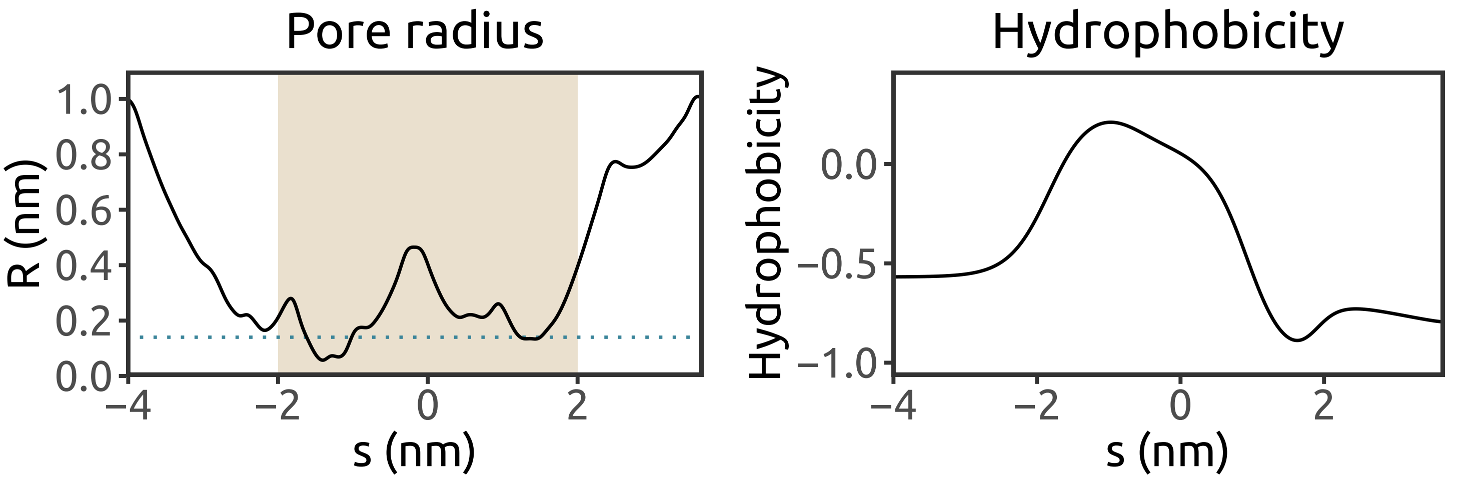


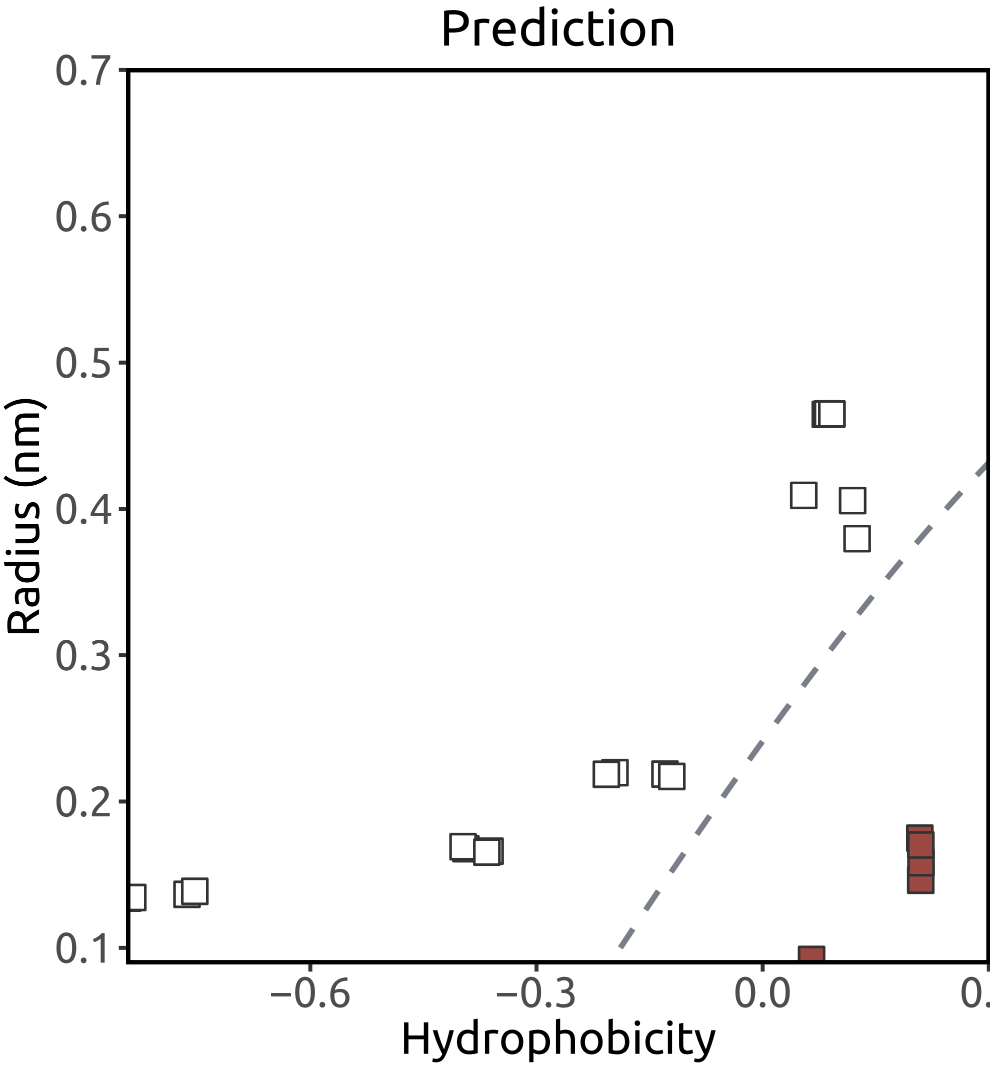


Heuristic score:

1.47 (n = 8)

Simulation result:

barrier to water

# TRPV6 (PDB ID: 5WO6)

*Rattus norvegicus*
X−ray (3.31 Å)

Singh et al., 2017

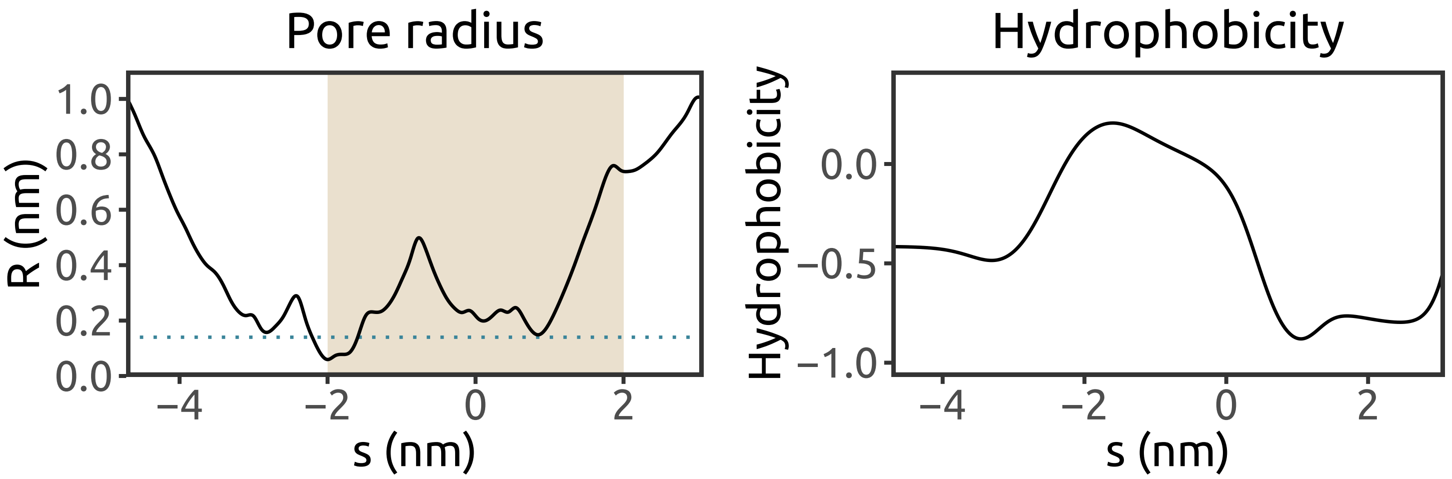


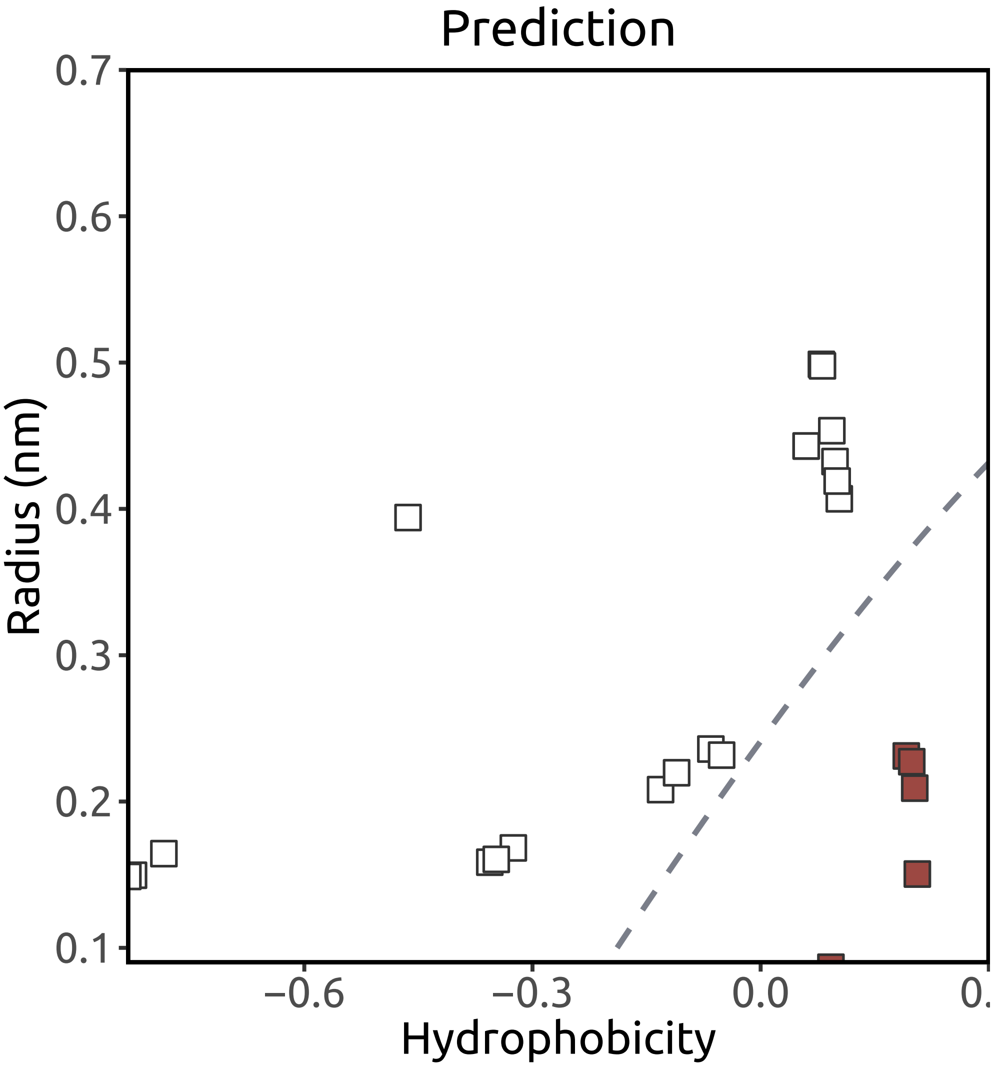


Heuristic score:
1.34 (n = 8)

Simulation result:
barrier to water

# TRPV6 (PDB ID: 6BO8)

*Homo sapiens*
cryo−EM (3.6 Å)

McGoldrick et al., 2018

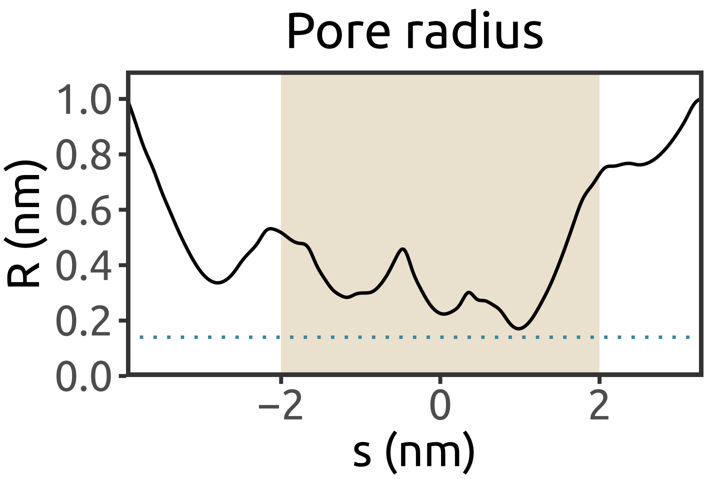


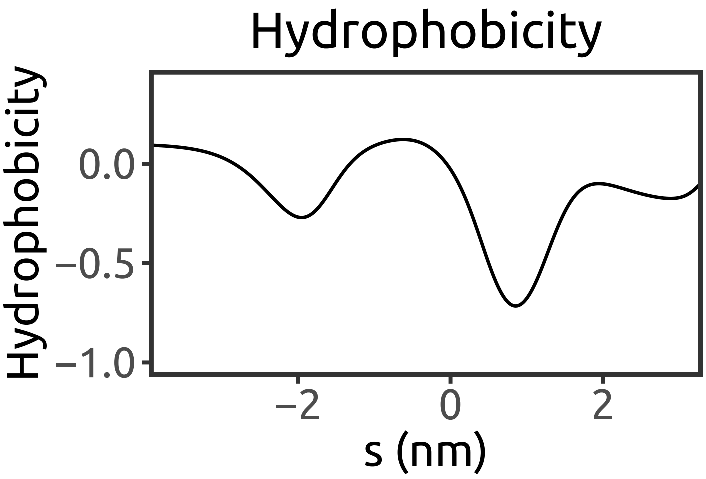


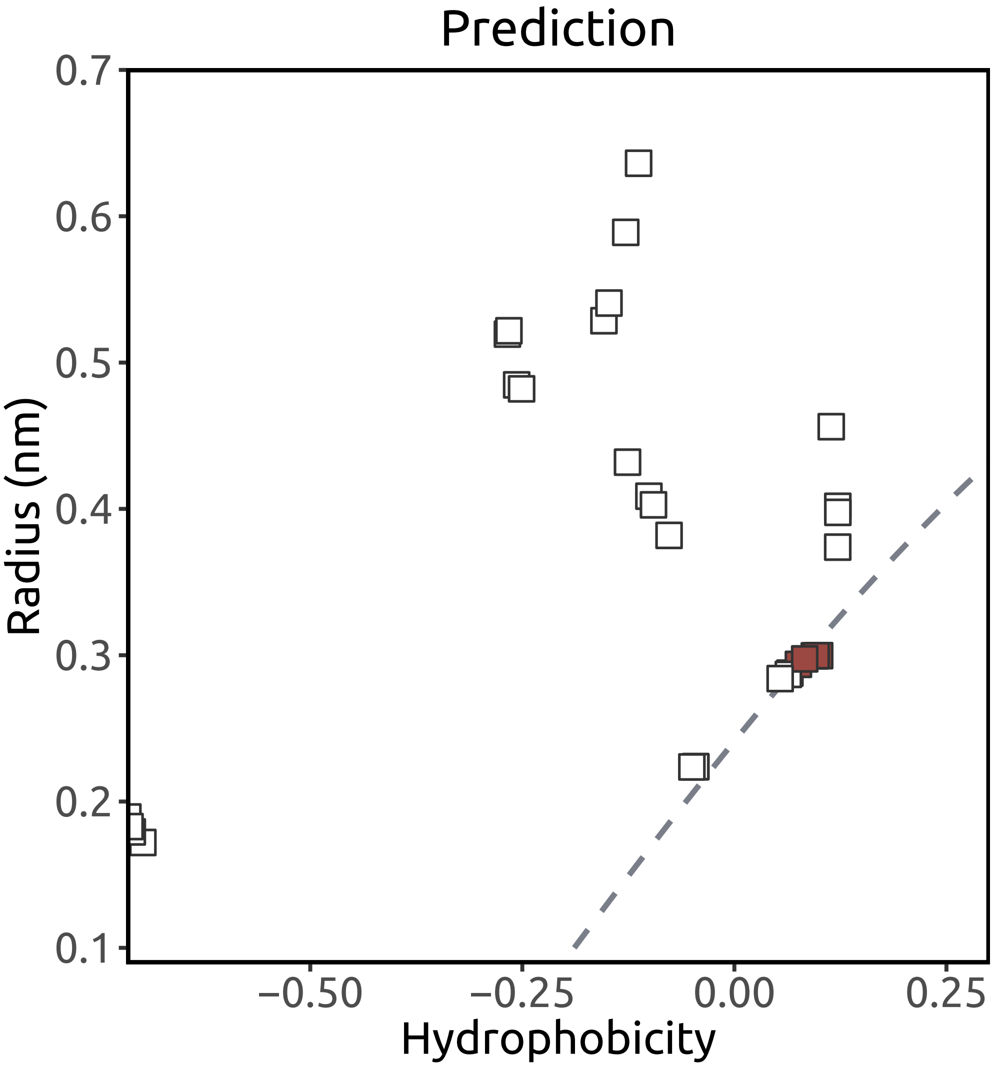


Heuristic score:
0.03 (n = 5)

Simulation result:
hydrated channel

# TRPV6 (PDB ID: 6BOB)

*Homo sapiens*
cryo−EM (3.9 Å)

McGoldrick et al., 2018

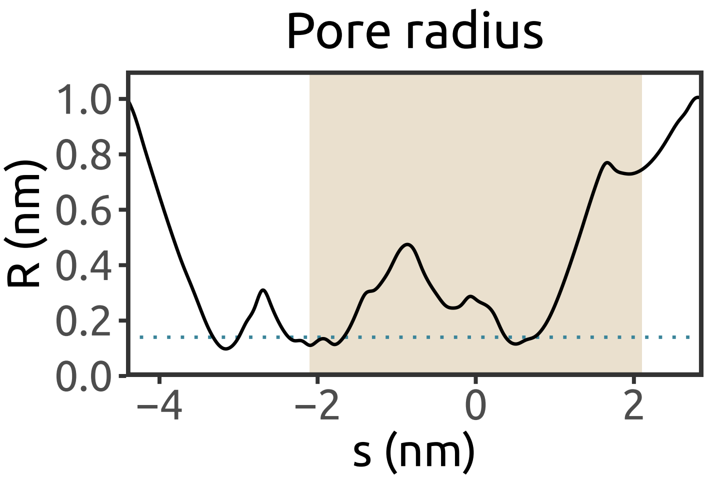


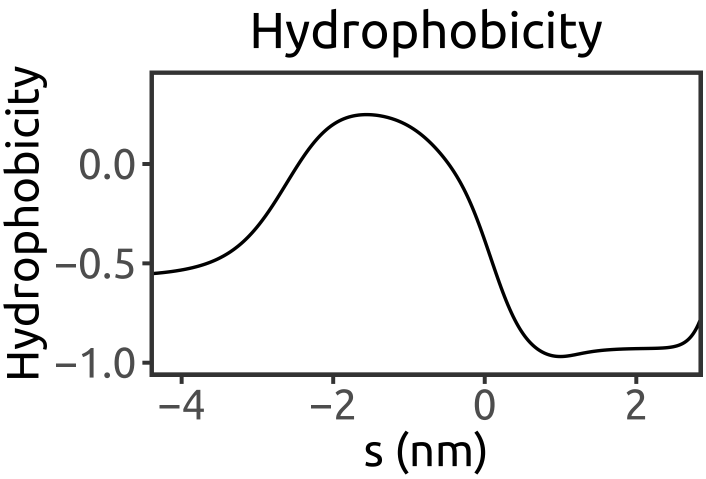


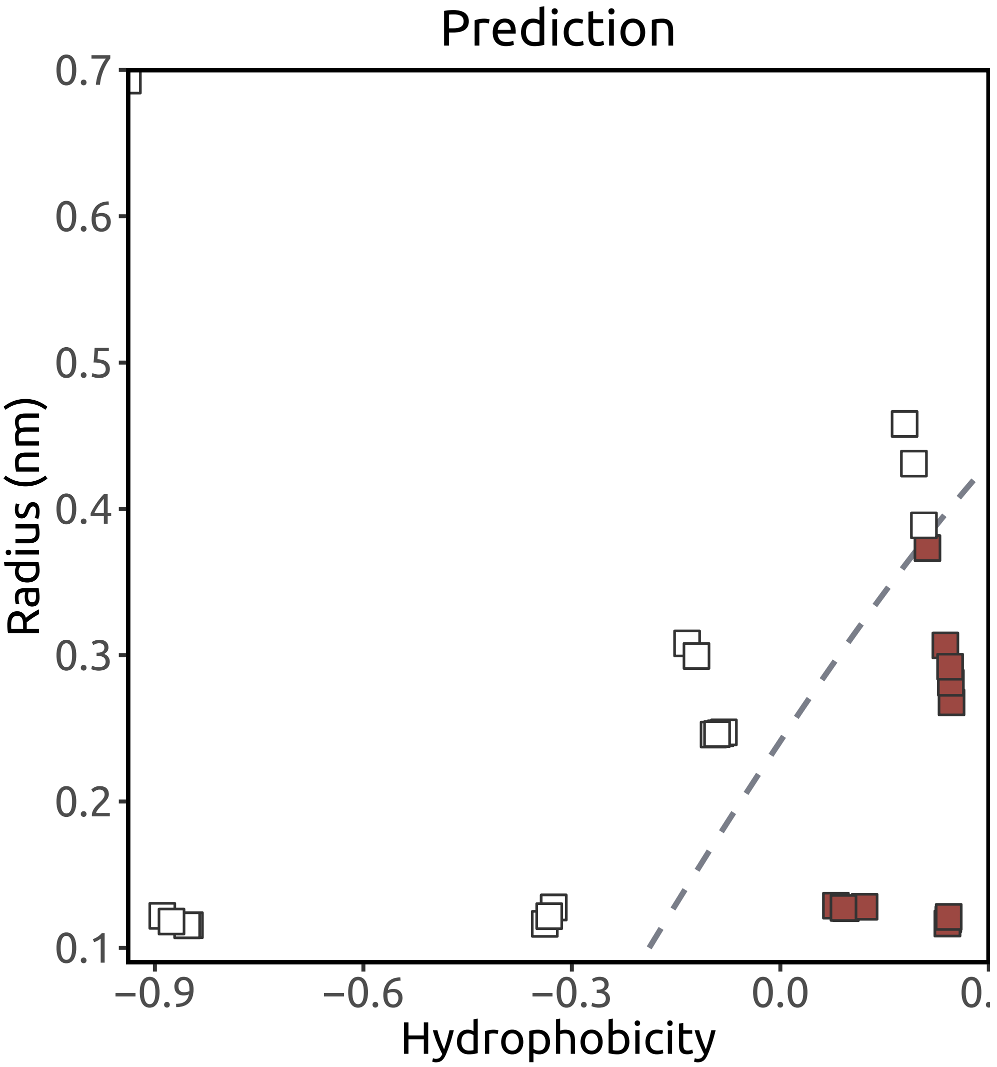


Heuristic score:
1.94 (n = 13)

Simulation result:
barrier to water

# GsuK (PDB ID: 4GX5)

*Geobacter sulfurreducens*
X−ray (3.7 Å)

Kong et al., 2012

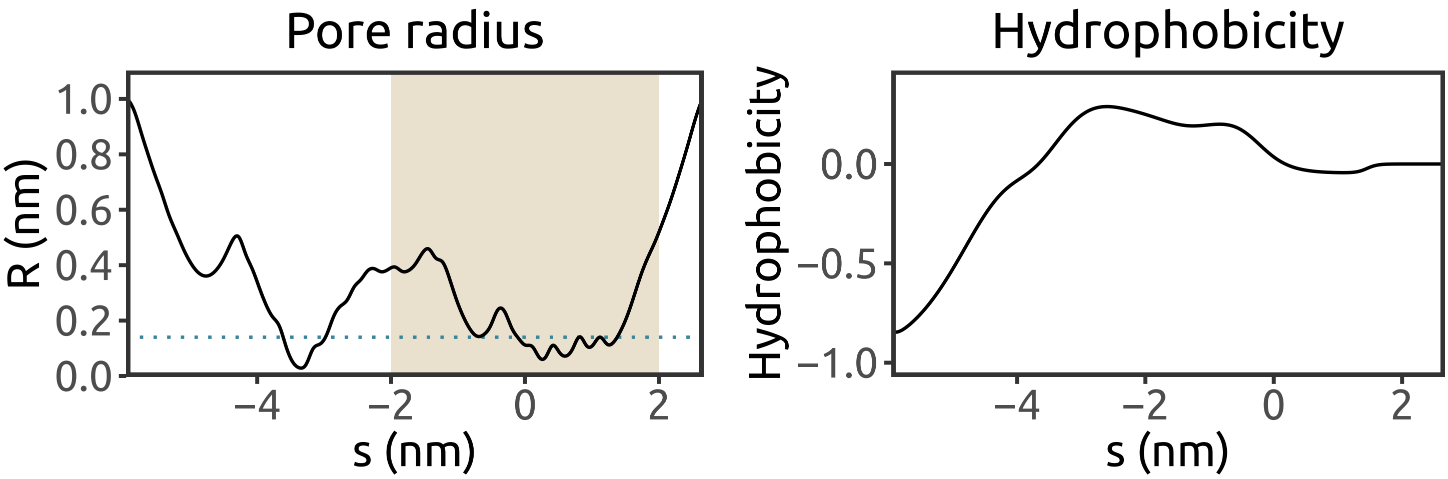


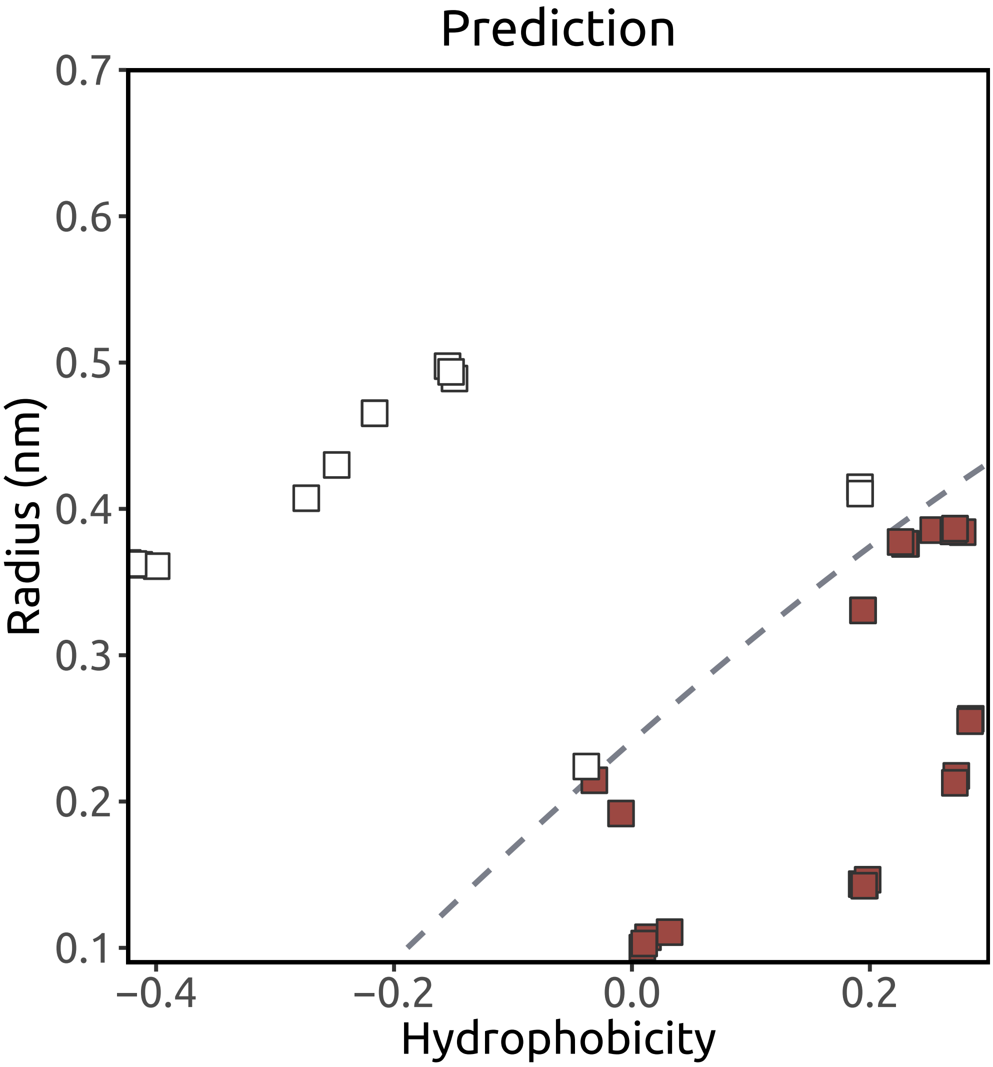


Heuristic score:
3.15 (n = 28)

Simulation result:
barrier to water

# K2P10.1 (PDB ID: 4BW5)

*Homo sapiens*
X–ray (3.2 Å)

Dong et al., 2015

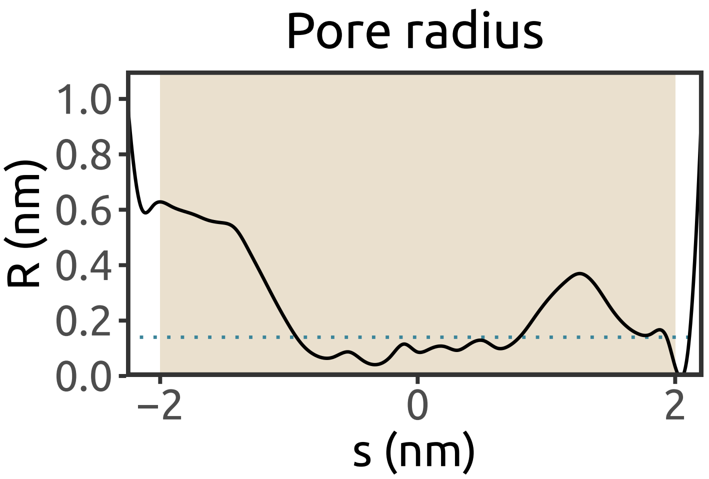


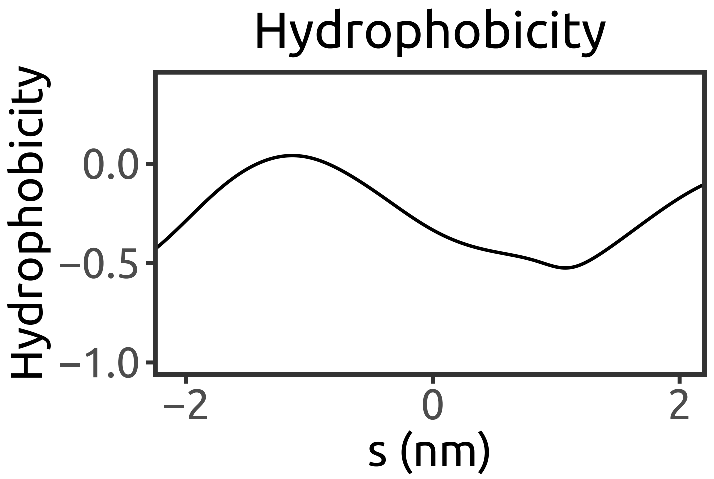


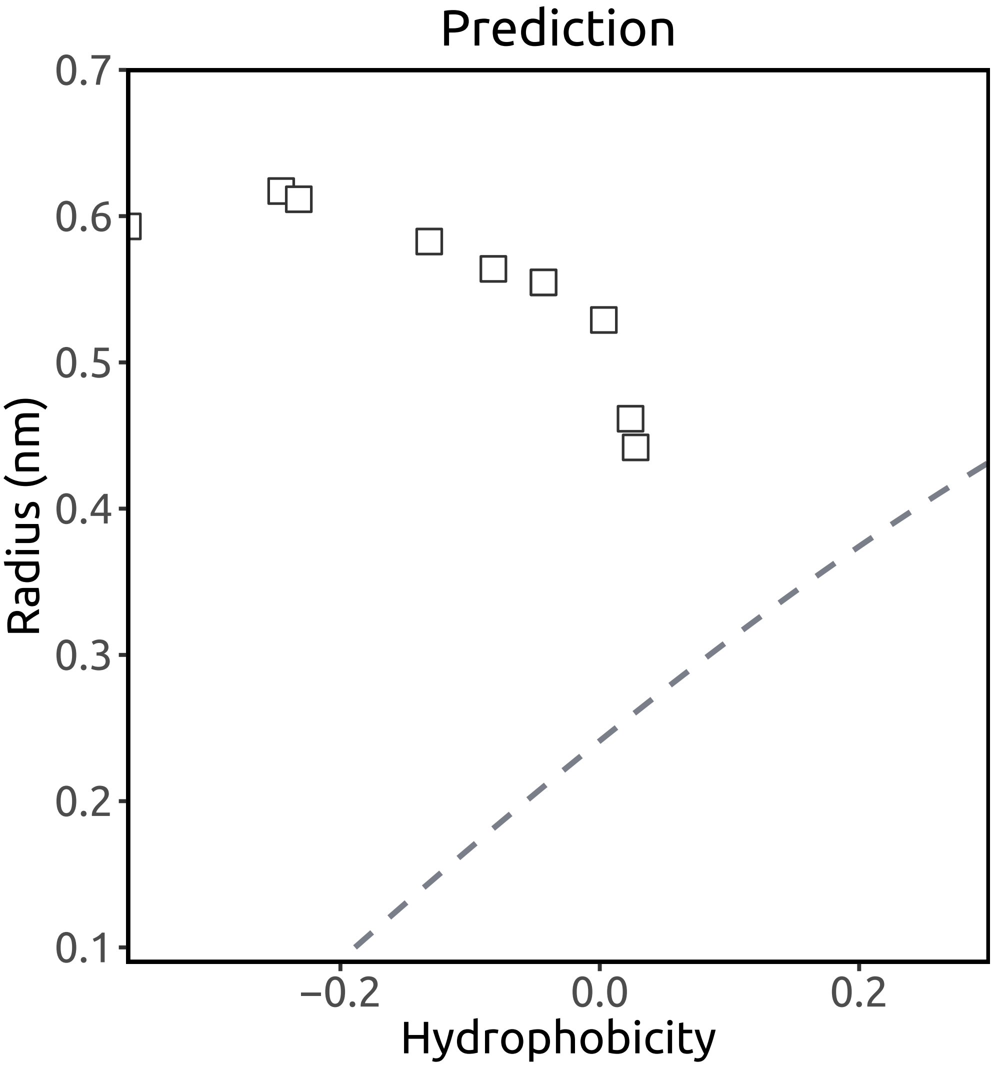


Heuristic score:
0 (n = 0)

Simulation result:
hydrated channel

# K2P10.1 (PDB ID: 4XDL)

*Homo sapiens*
X–ray (3.5 Å)

Dong et al., 2015

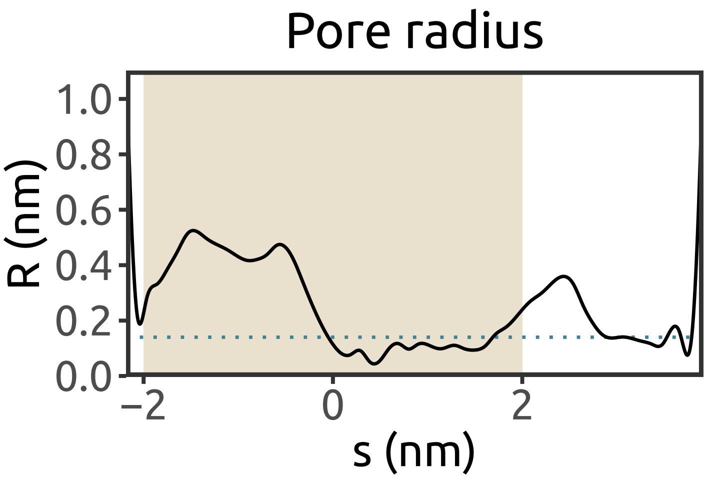


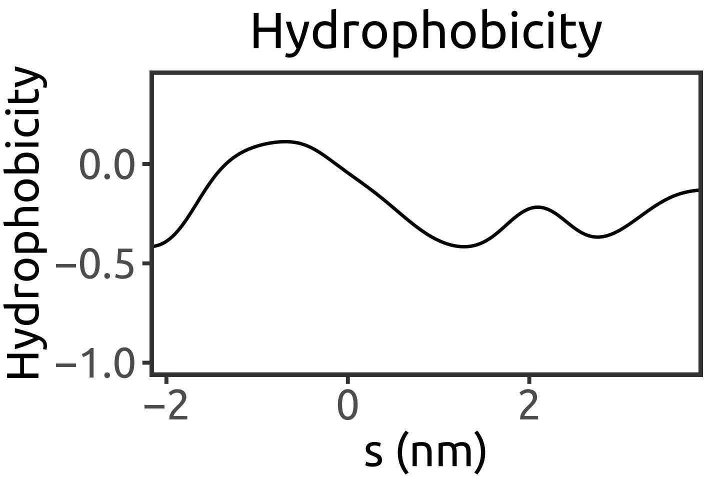


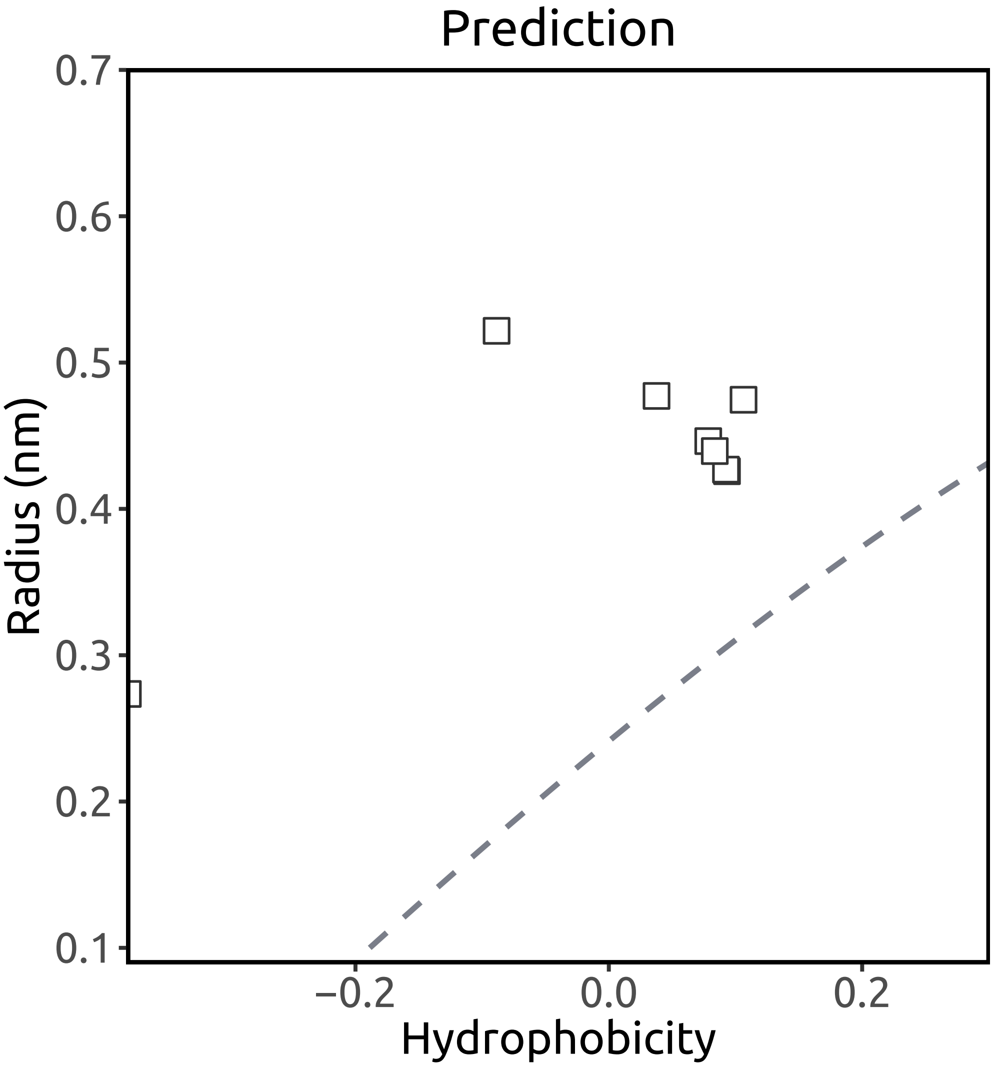


Heuristic score:
0 (n = 0)

Simulation result:
hydrated channel

# K2P1.1 (PDB ID: 3UKM)

*Homo sapiens*

X–ray (3.4 Å)

Miller & Long, 2012

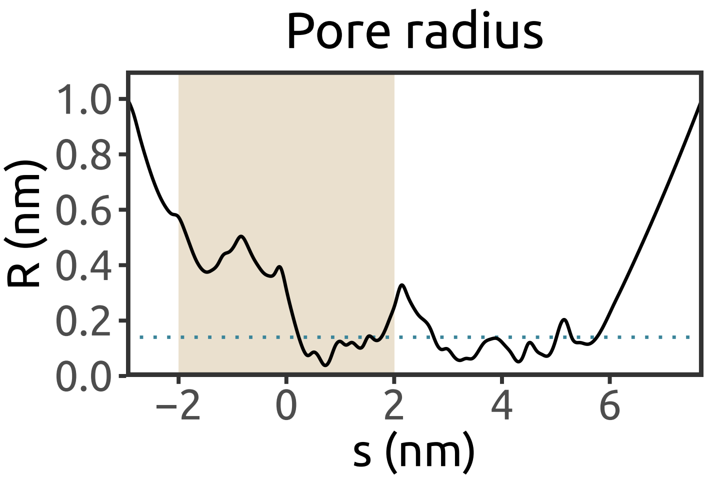


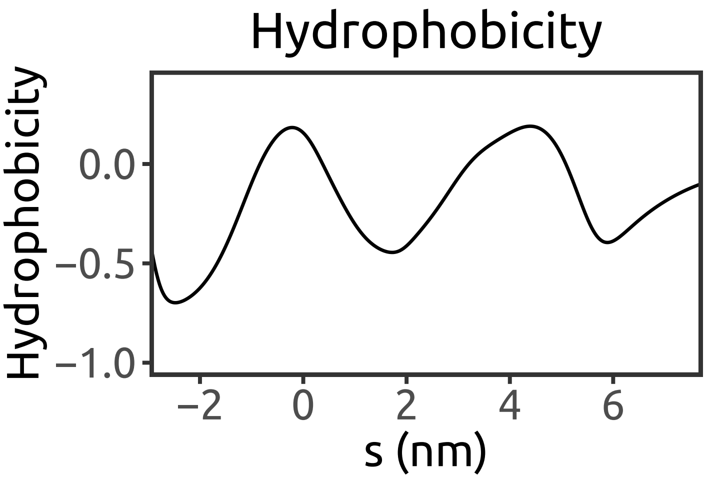


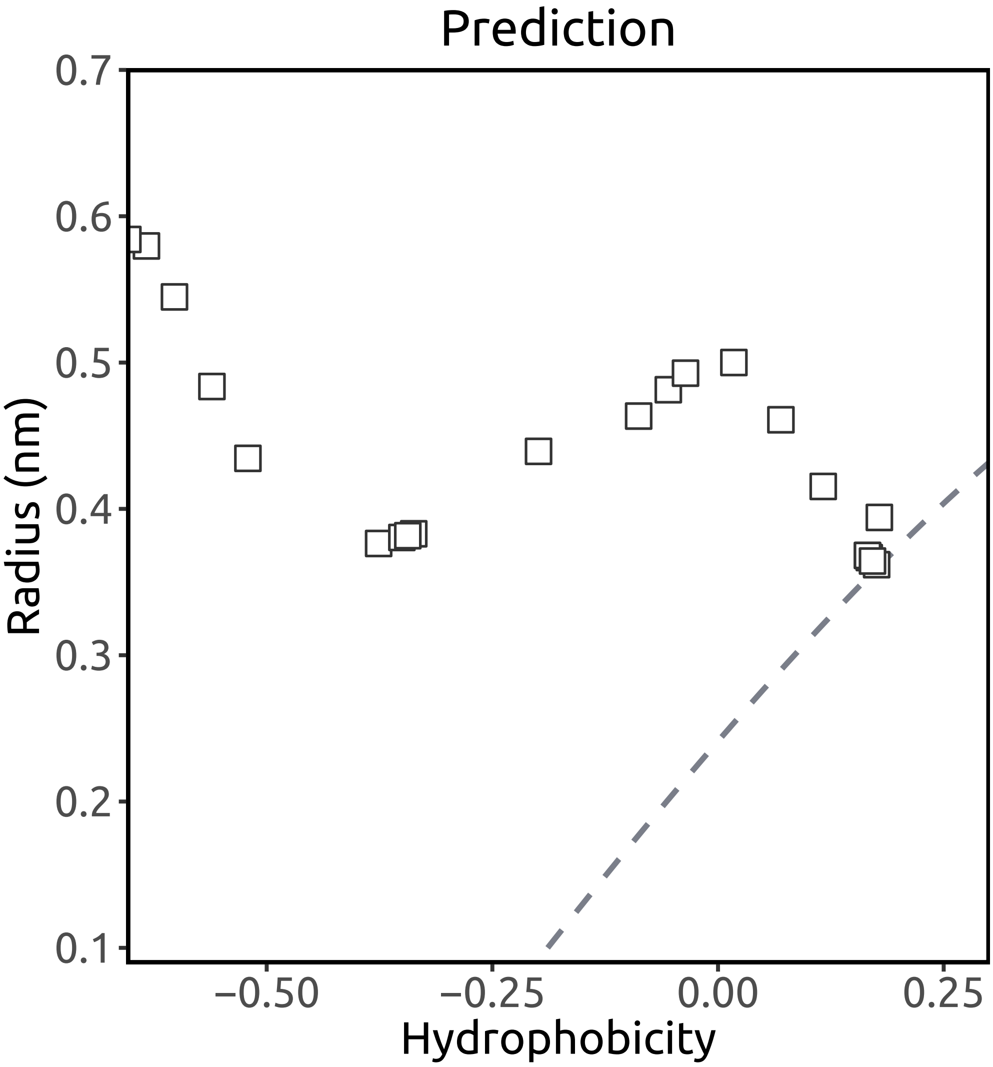


Heuristic score:

0 (n = 0)

Simulation result:

hydrated channel

# K2P2.1 (PDB ID: 5VKP)

*Mus musculus*
X–ray (2.8 Å)

Lolicato et al., 2017

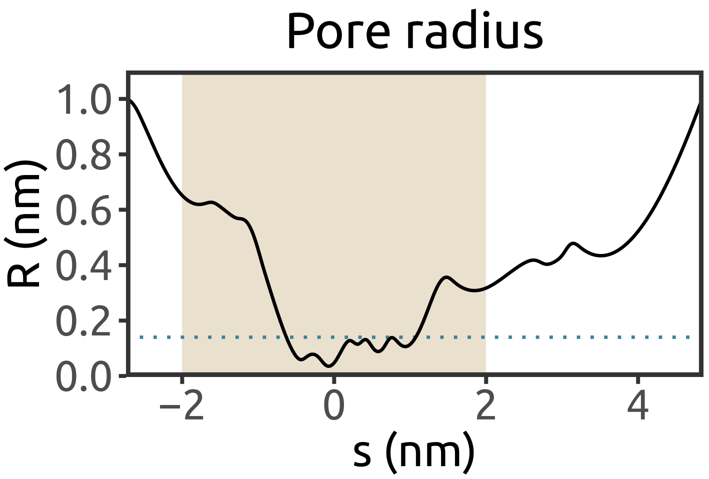


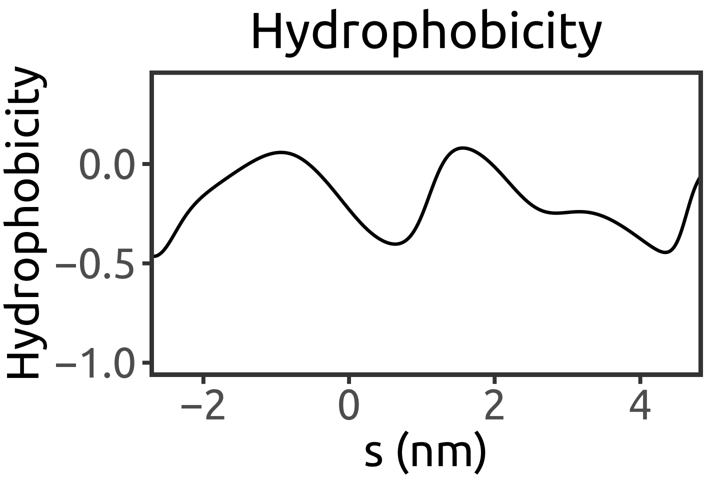


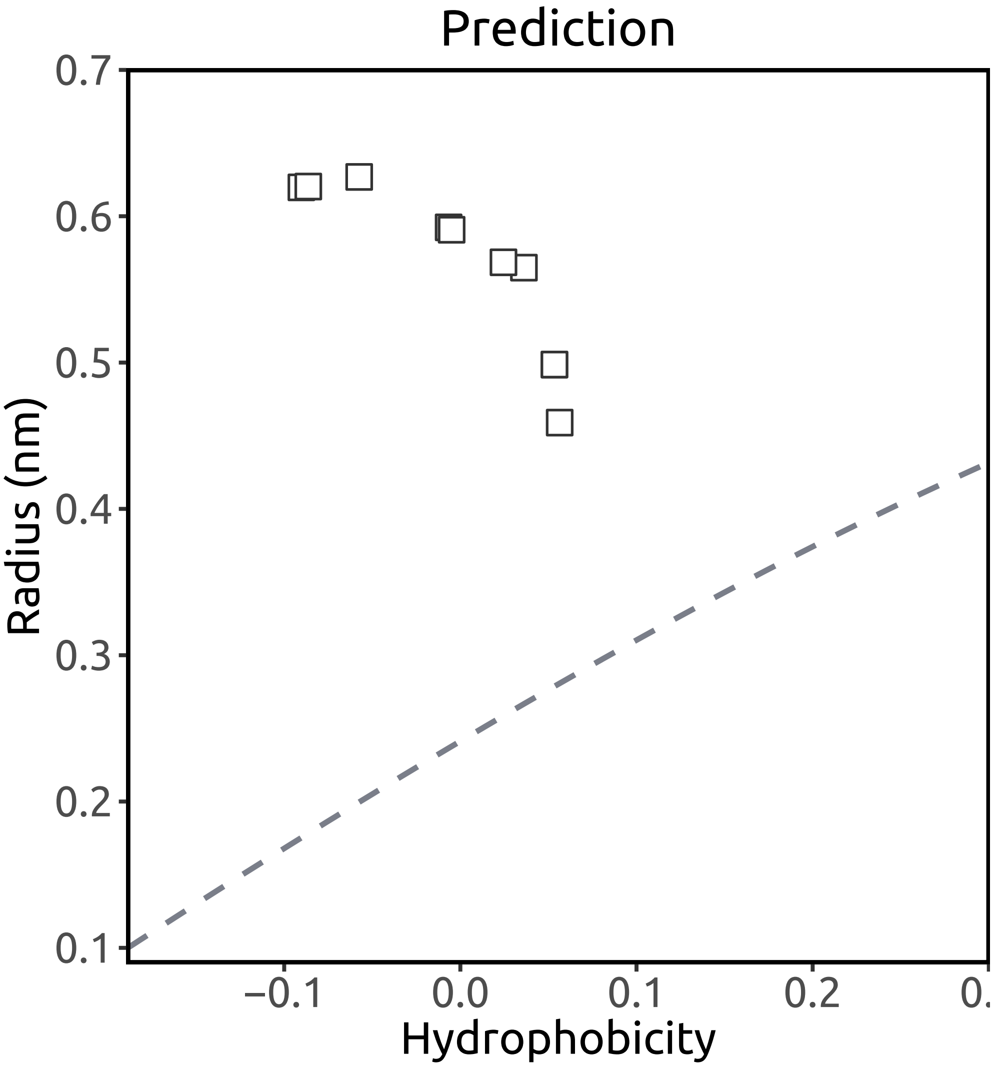


Heuristic score:
0 (n = 0)

Simulation result:
hydrated channel

# K2P4.1 (PDB ID: 4WFE)

*Homo sapiens*

X–ray (2.5 Å)

Brohawn et al., 2014

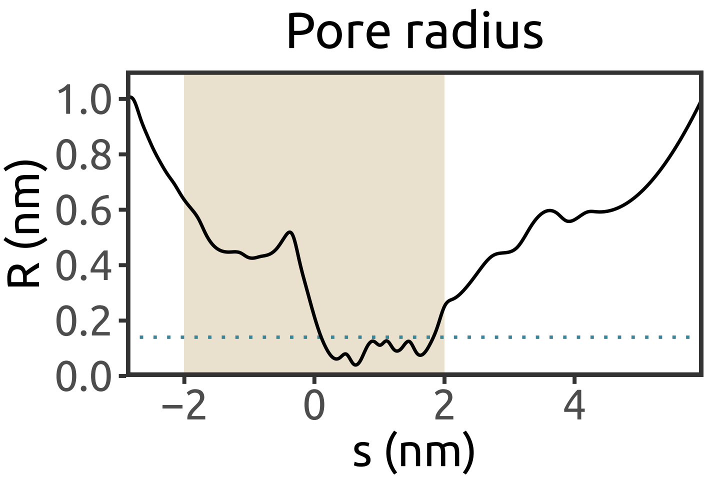


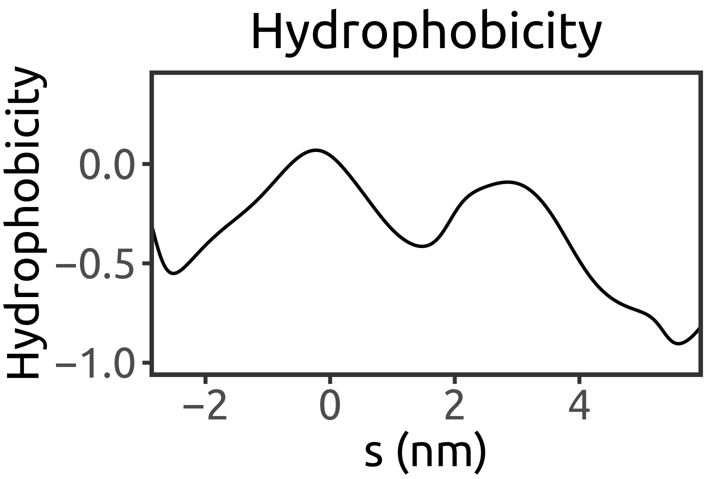


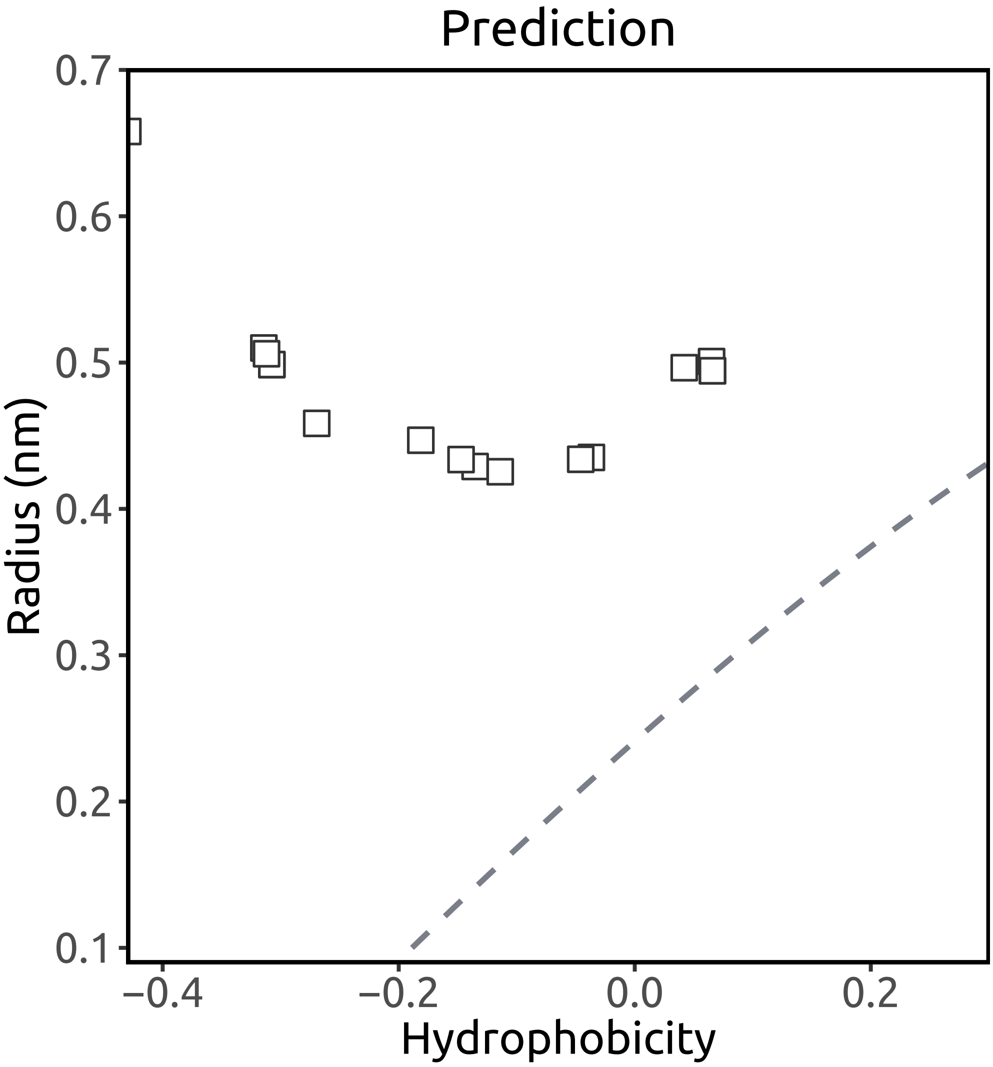


Heuristic score:

0 (n = 0)

Simulation result:

hydrated channel

# KcsA (PDB ID: 1K4C)

*Streptomyces lividans*

X−ray (2 Å)

Zhou et al., 2001

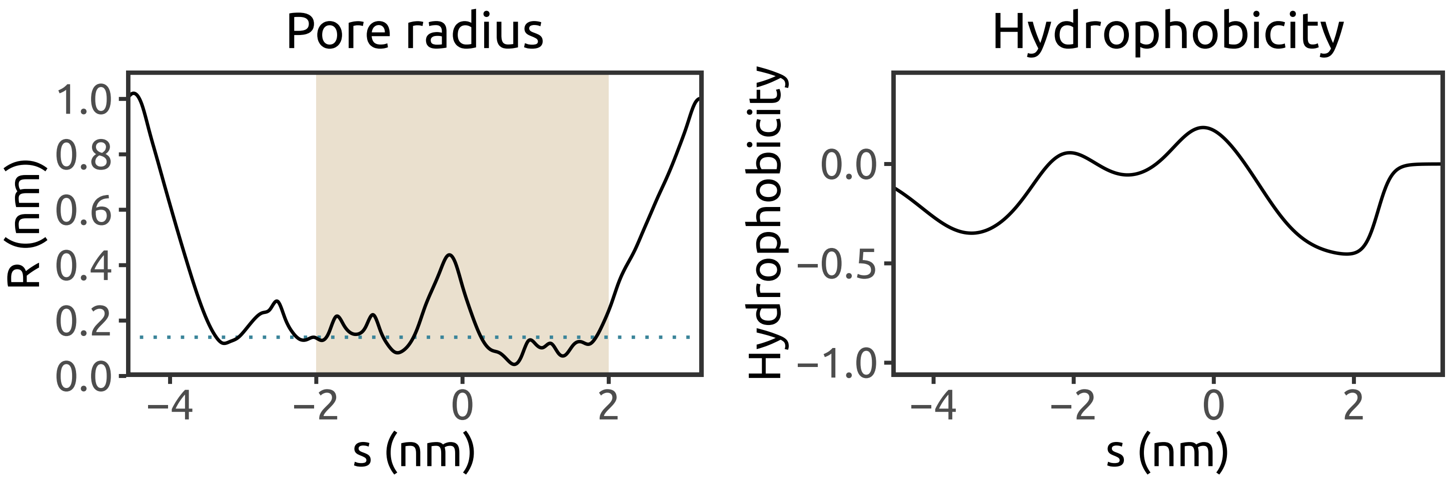


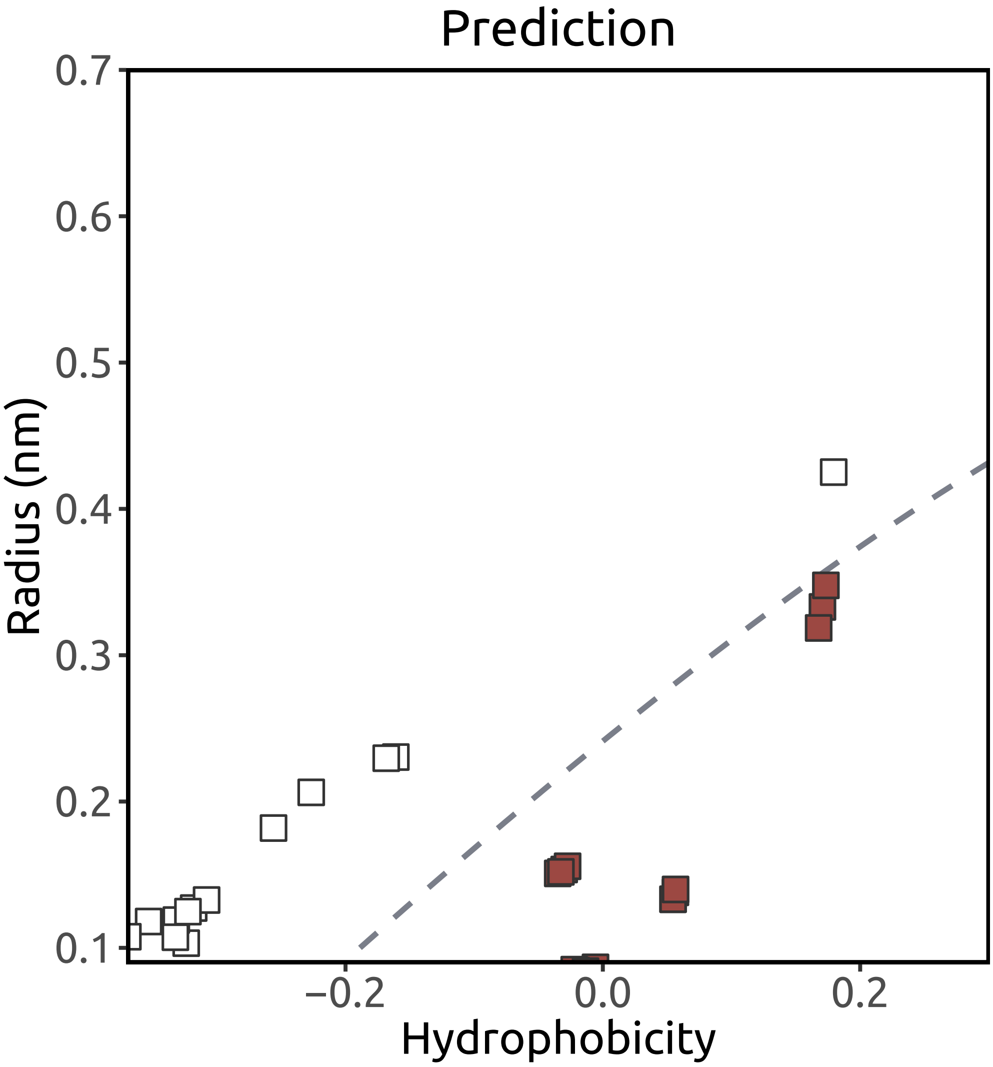


Heuristic score:

1.63 (n = 19)

Simulation result:

barrier to water

# KcsA (PDB ID: 2ITD)

*Streptomyces lividans*
X−ray (2.7 Å)

Lockless et al., 2007

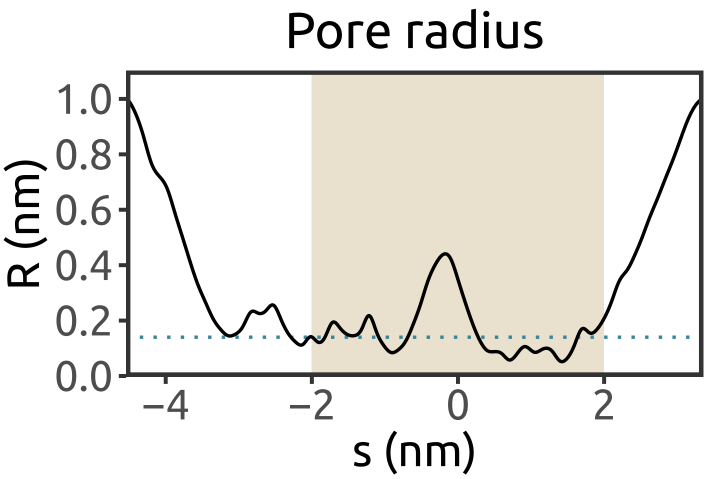


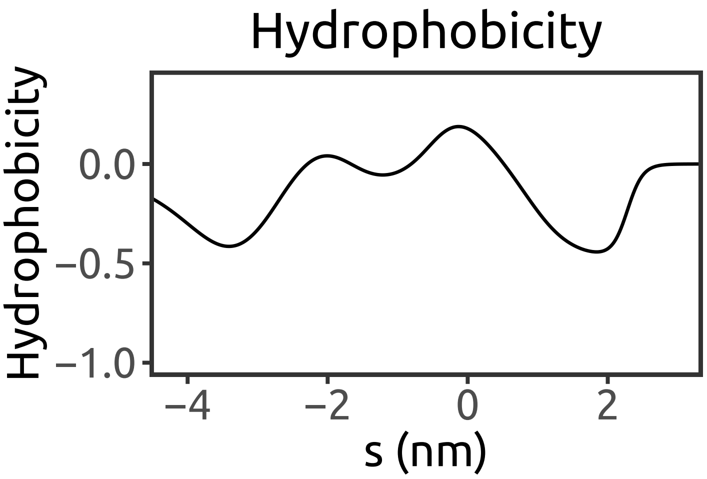


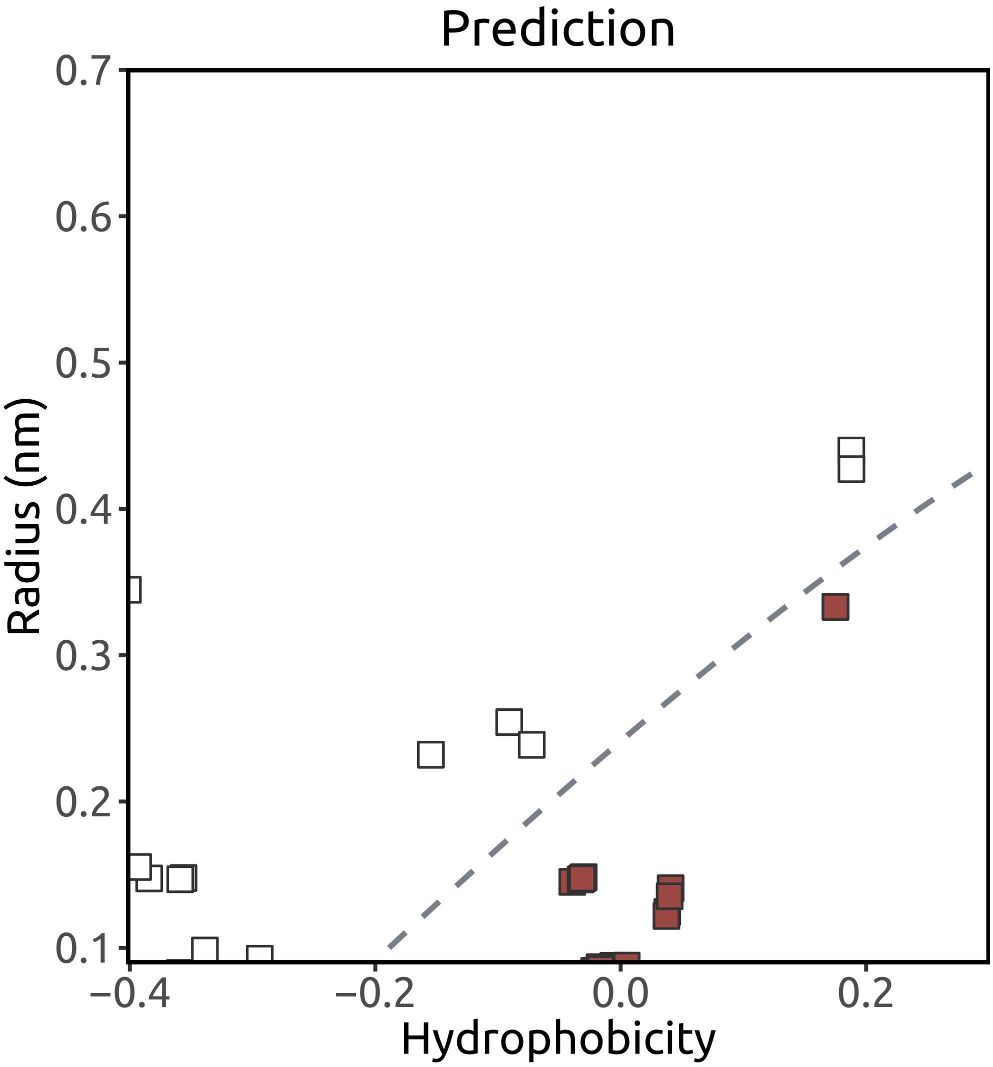


Heuristic score:
1.65 (n = 18)

Simulation result:
barrier to water

# KcsA (PDB ID: 3EFF)

*Streptomyces lividans*

X–ray (3.8 Å)

Uysal et al., 2009

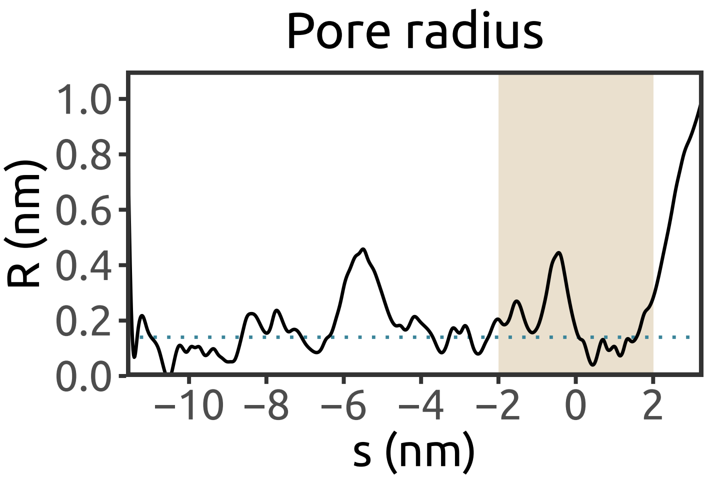


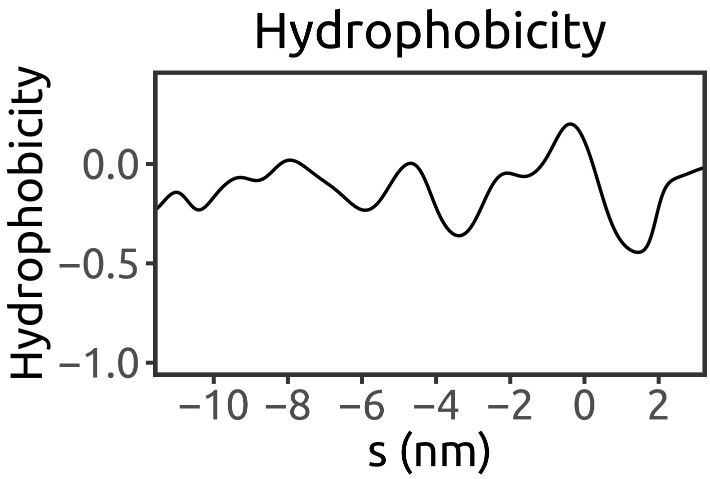


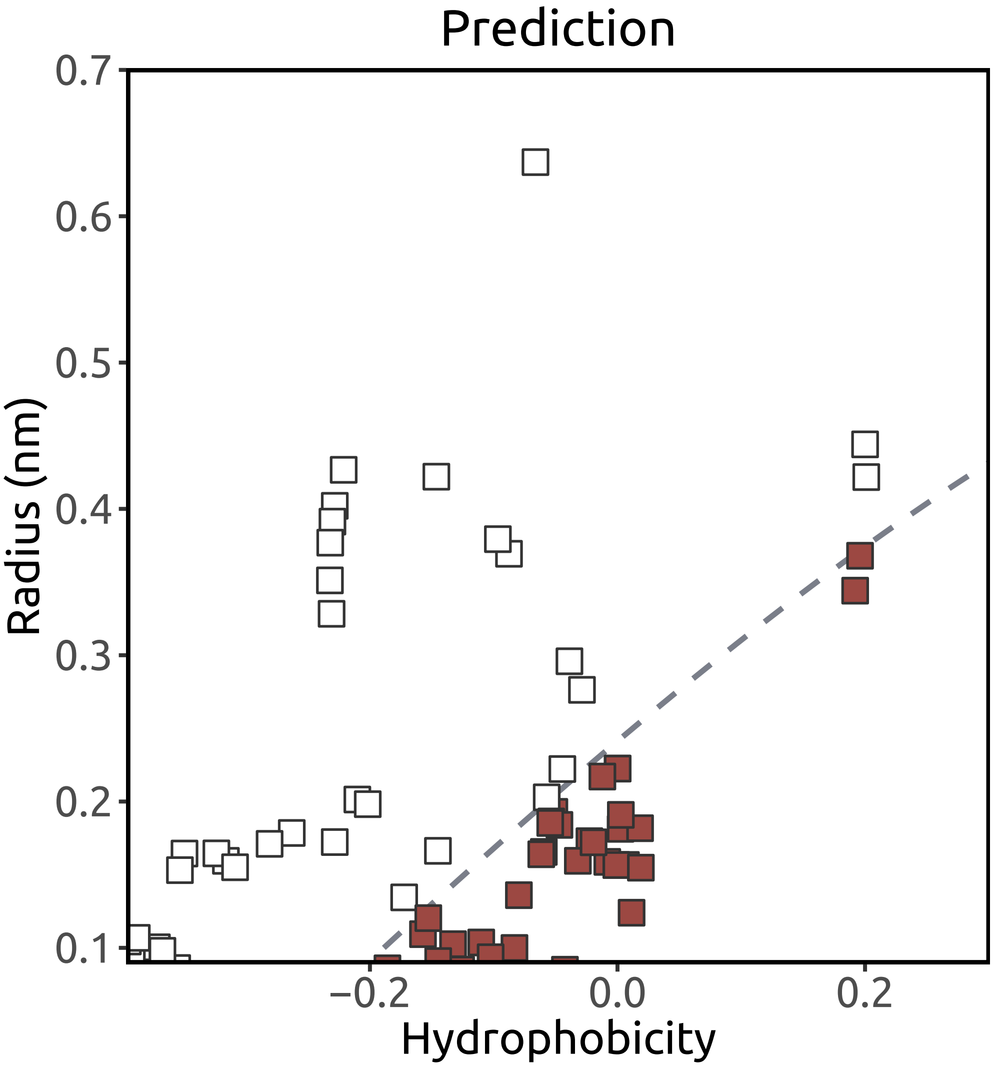


Heuristic score:

1.95 (n = 41)

Simulation result:

barrier to water

# KcsA (PDB ID: 3FB5)

*Streptomyces lividans*

X–ray (2.8 Å)

Cuello et al., 2010

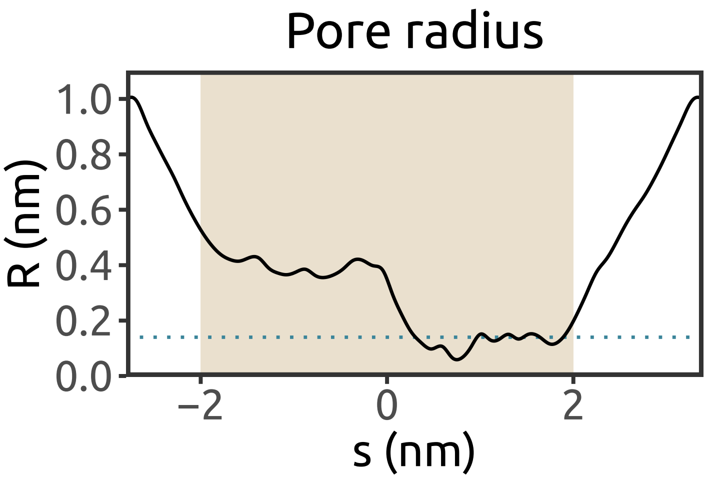


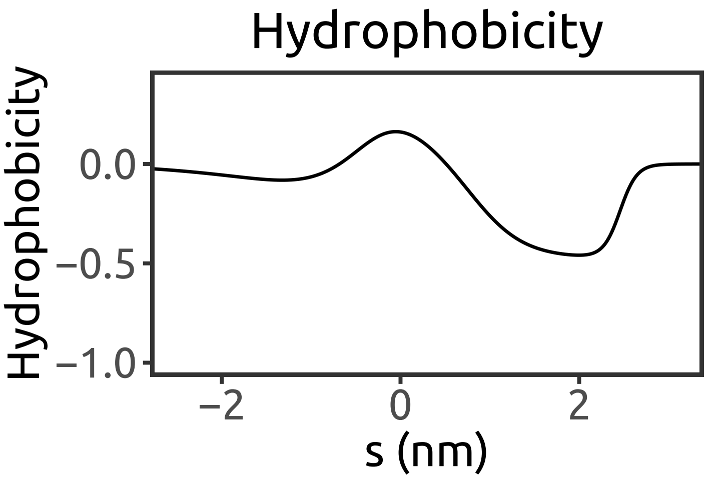


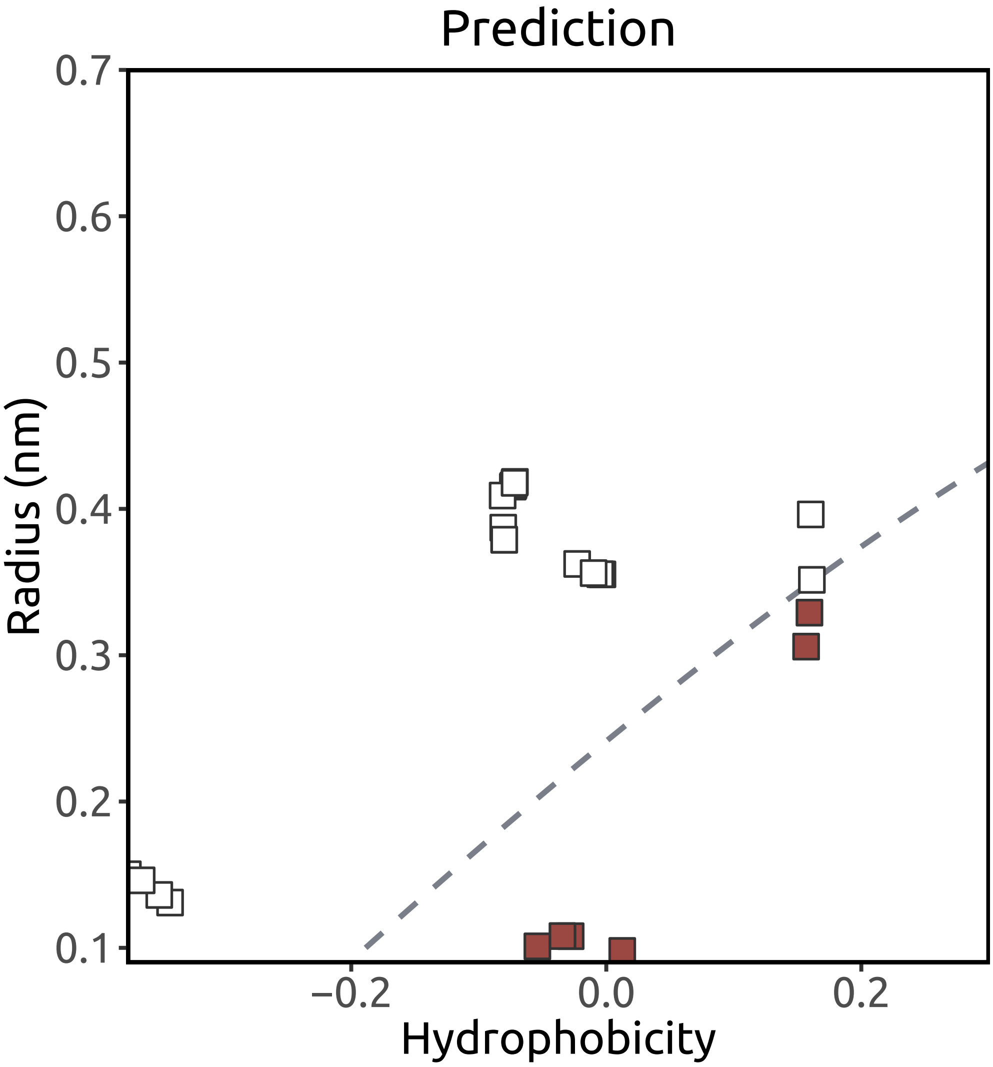


Heuristic score:

0.43 (n = 6)

Simulation result:

hydrated channel

# KcsA (PDB ID: 3PJS)

*Streptomyces lividans*

X–ray (3.8 Å)

Uysal et al., 2011

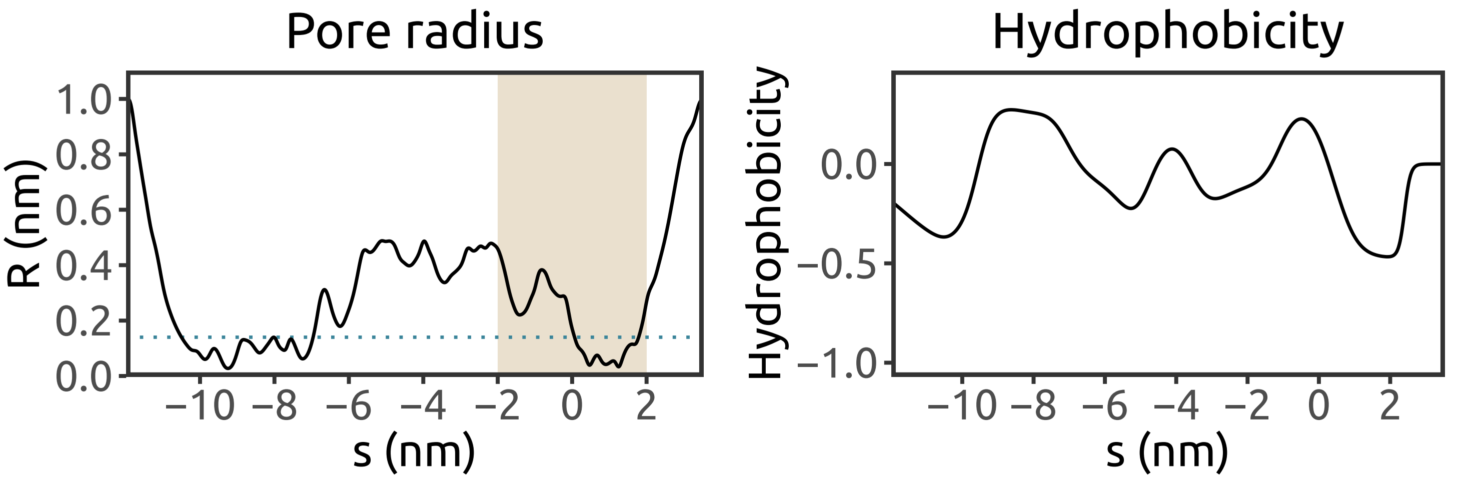


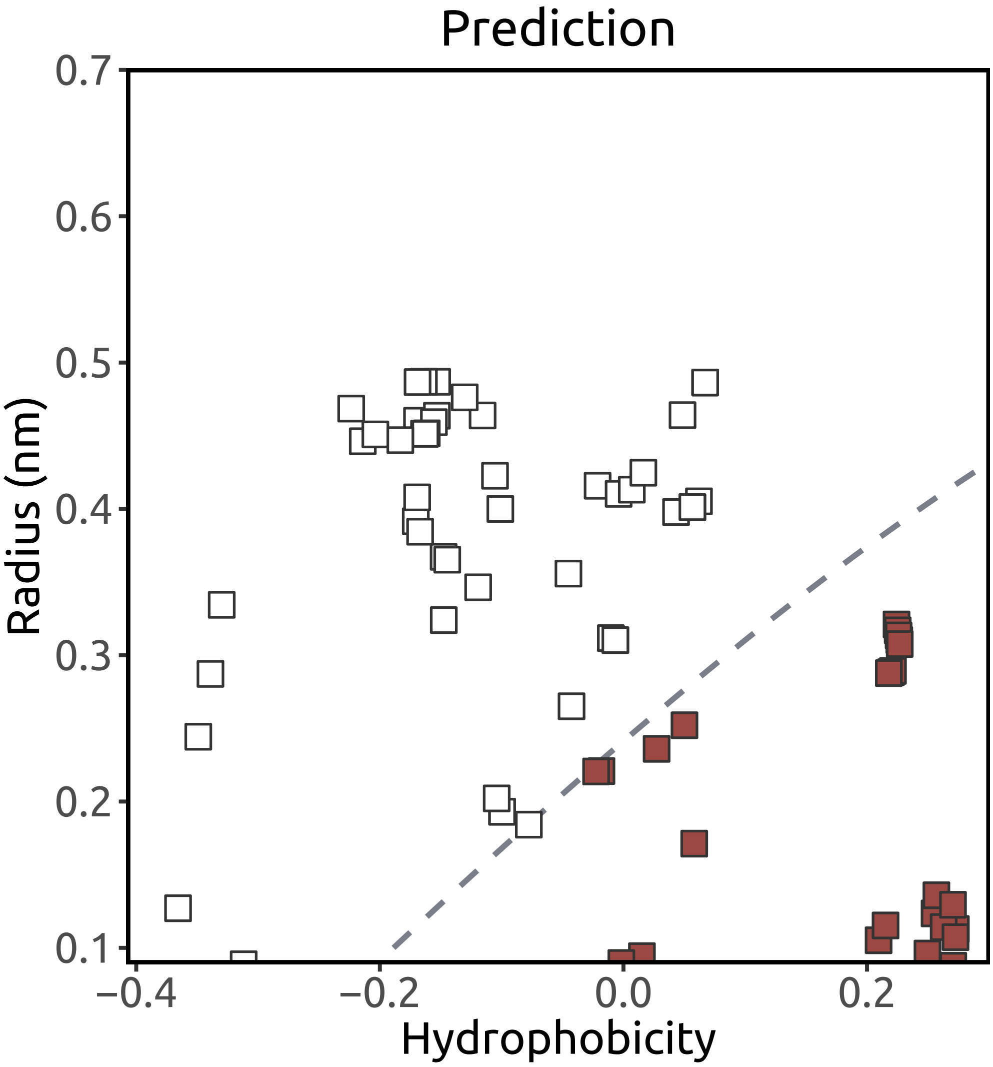


Heuristic score:

5 (n = 32)

Simulation result:

barrier to water

# KcsA (PDB ID: 4UUJ)

*Streptomyces lividans*

X–ray (2.4 Å)

Lenaeus et al., 2014

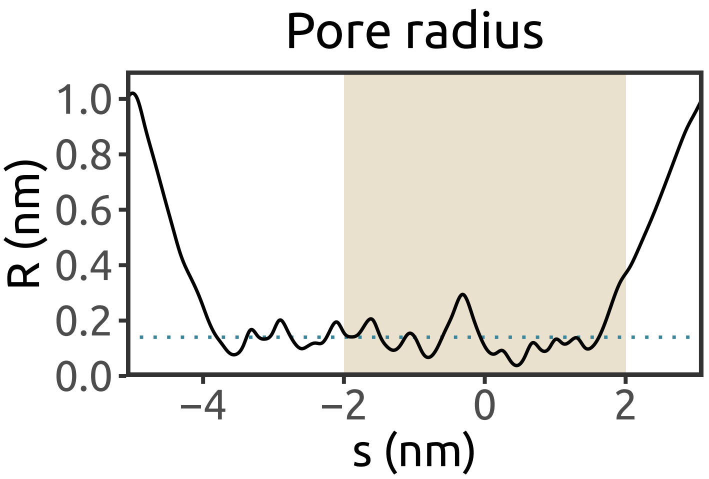


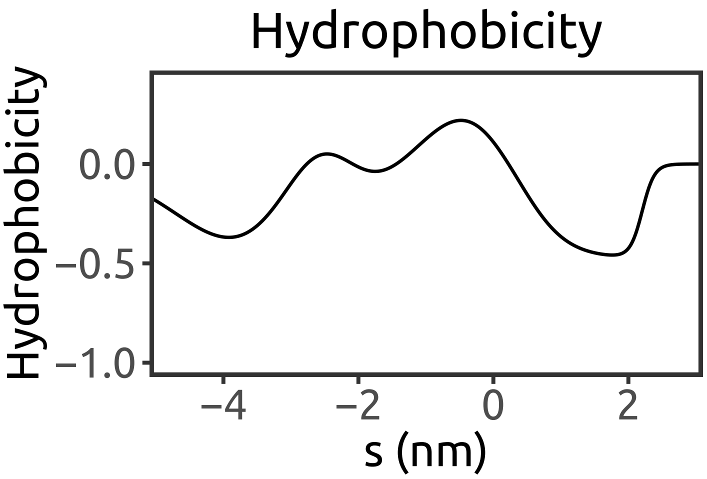


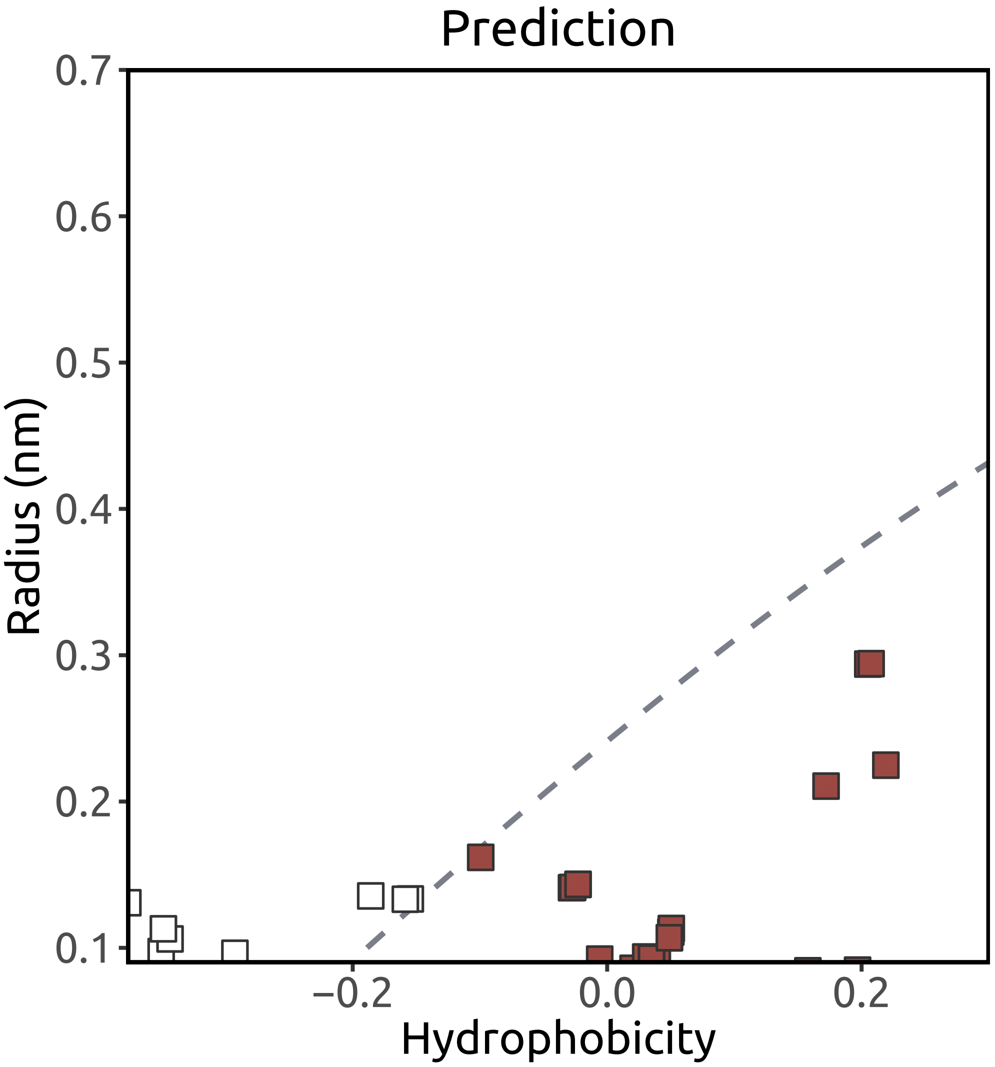


Heuristic score:

3.17 (n = 25)

Simulation result:

barrier to water

# Kir2.2 (PDB ID: 3JYC)

*Gallus gallus*
X−ray (3.11 Å)

Tao et al., 2009

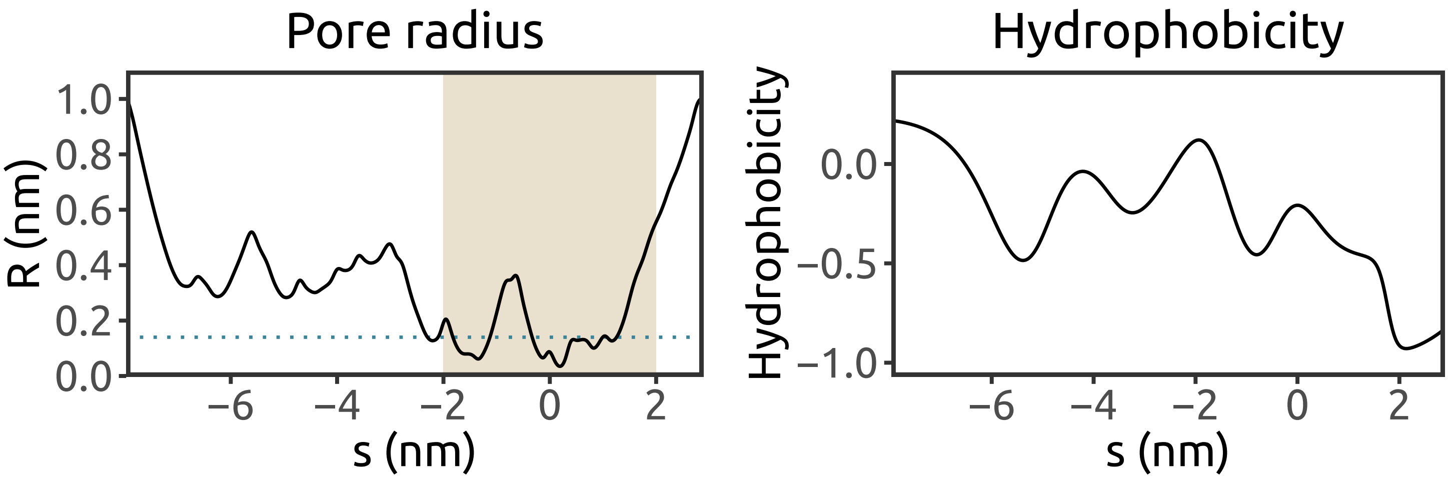


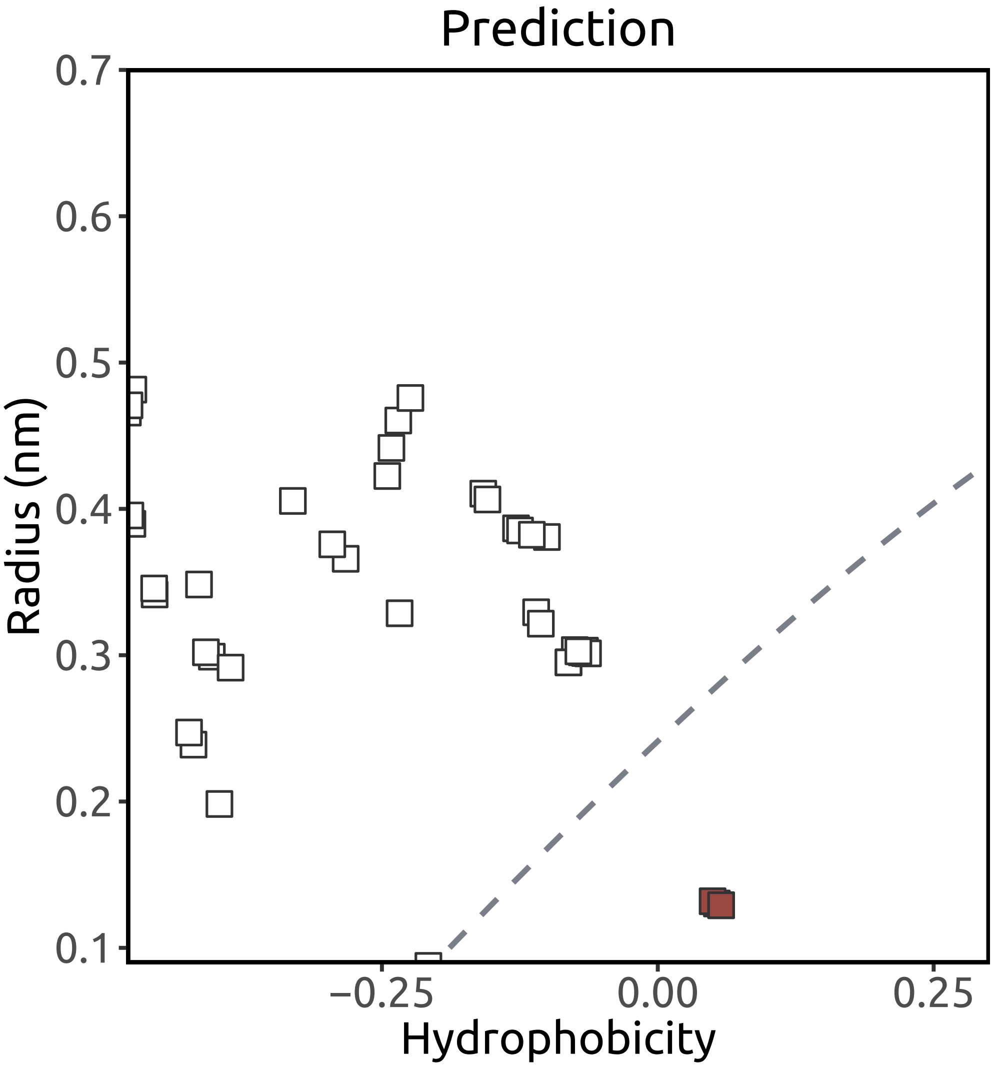


Heuristic score:
0.88 (n = 8)

Simulation result:
barrier to water

# Kir2.2 (PDB ID: 3SPC)

*Gallus gallus*
X–ray (2.45 Å)

Hansen et al., 2011

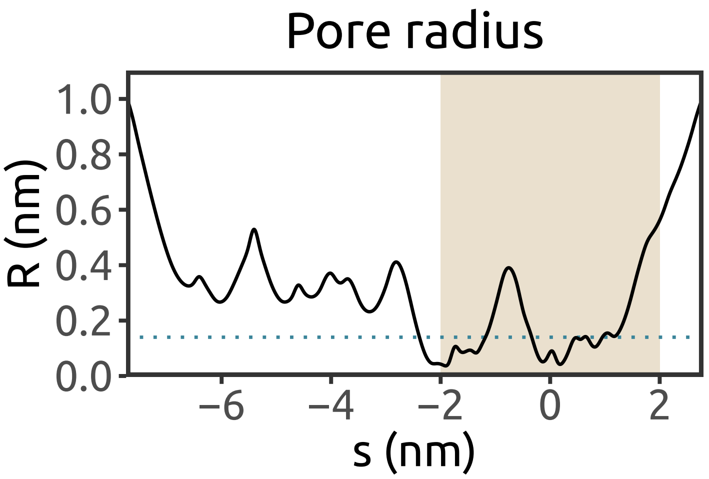


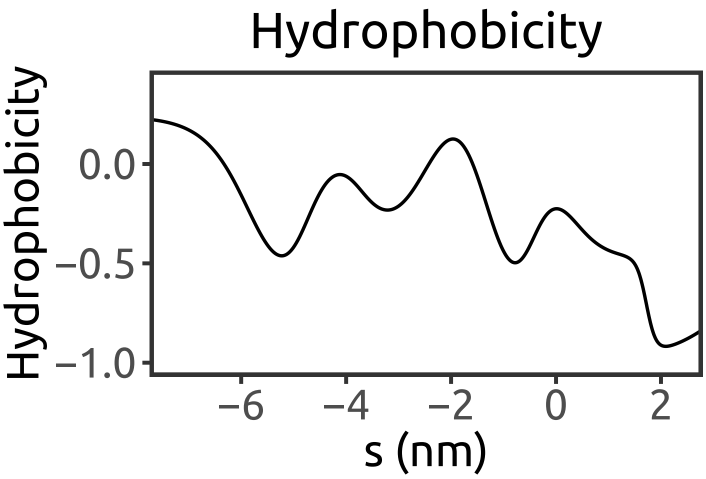


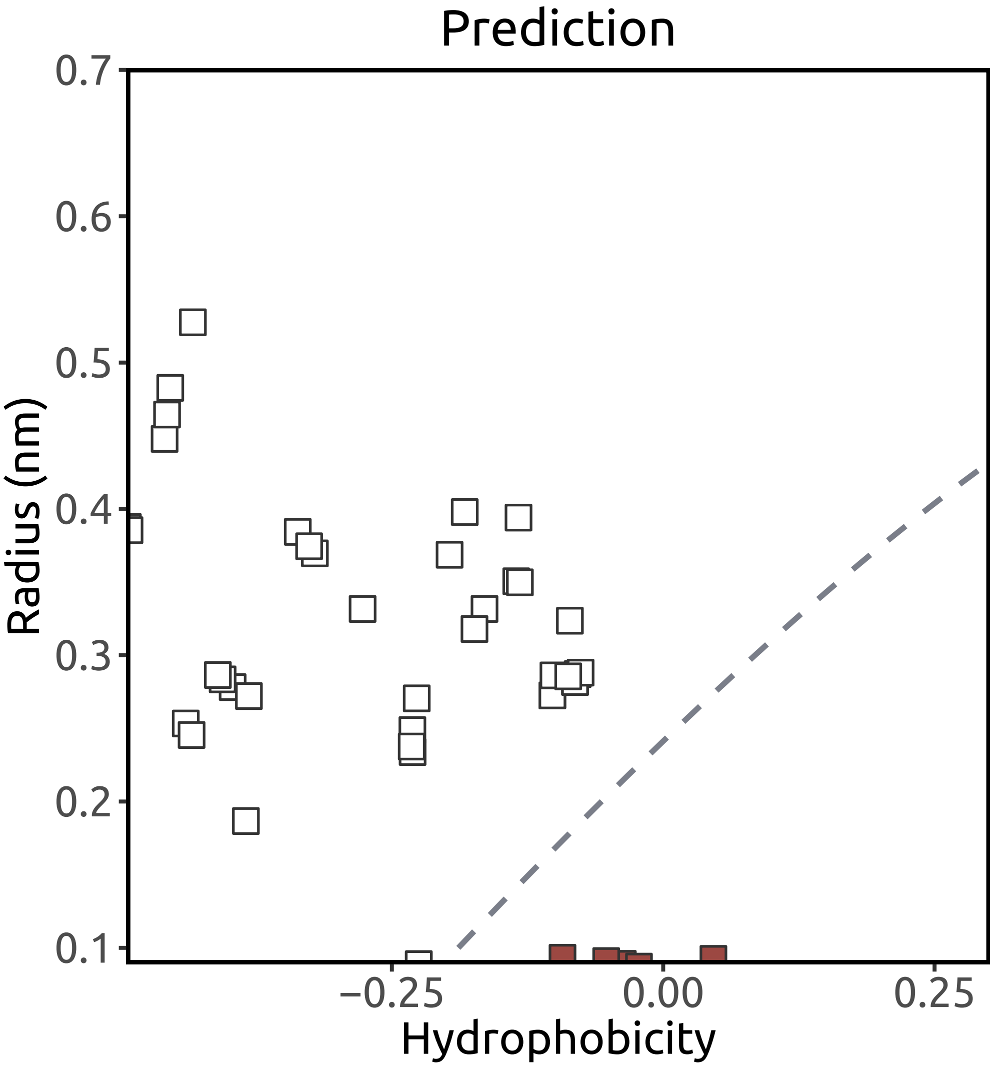


Heuristic score:
1.15 (n = 8)

Simulation result:
barrier to water

# Kir2.2 (PDB ID: 3SPI)

*Gallus gallus*

X–ray (3.31 Å)

Hansen et al., 2011

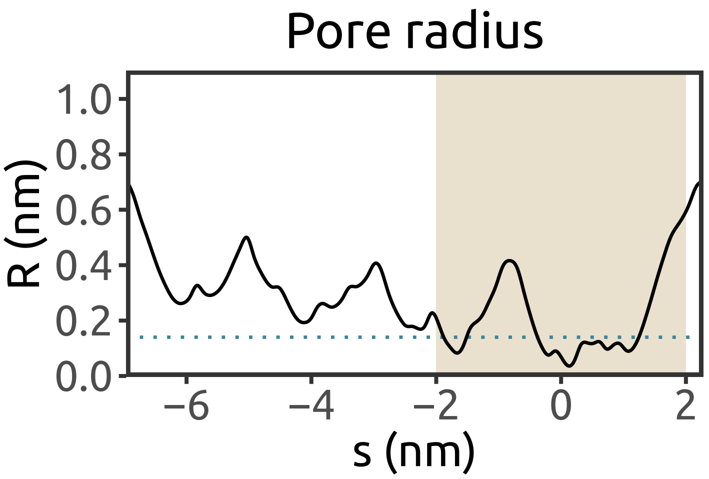


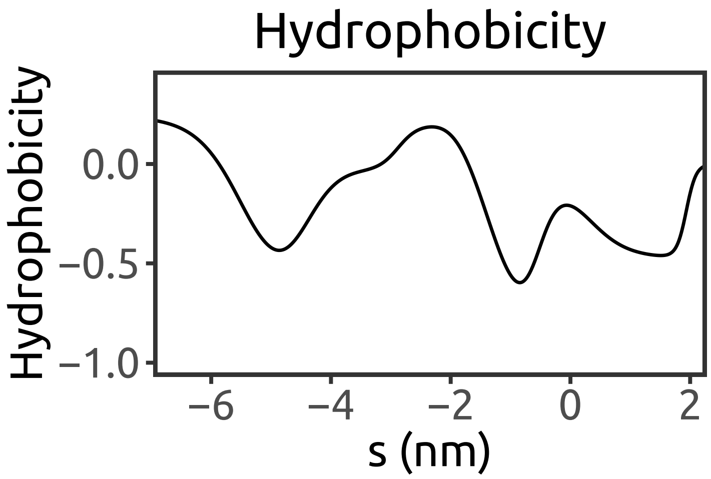


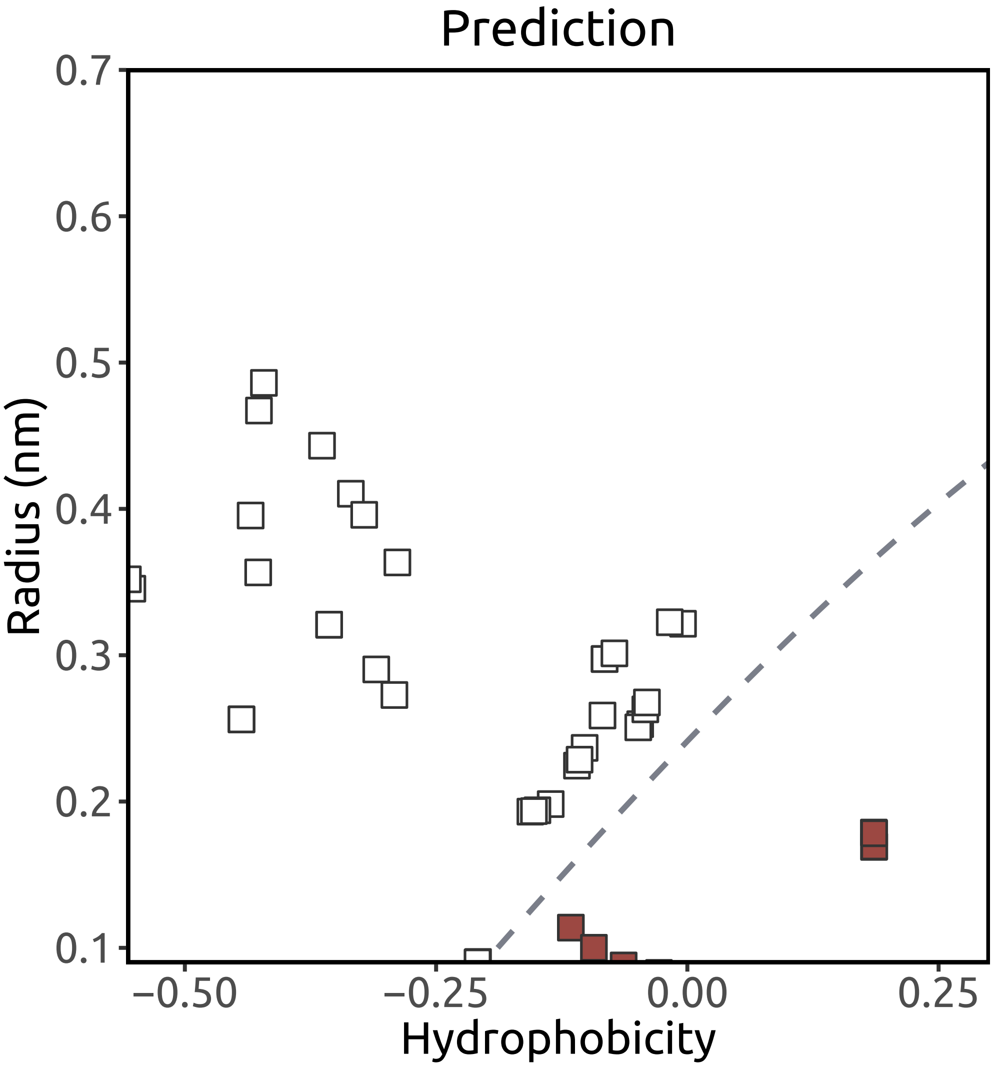


Heuristic score:

0.93 (n = 8)

Simulation result:

barrier to water

# Kir3.2 (PDB ID: 3SYA)

*Mus musculus*
X–ray (2.98 Å)

Whorton & Mackinnon, 2011

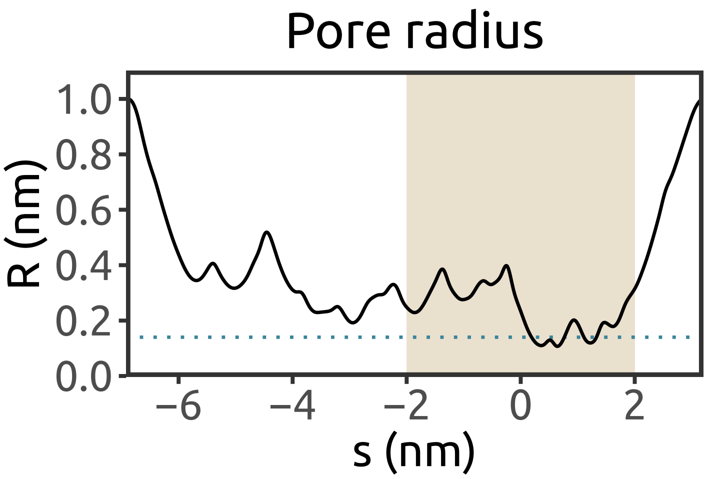


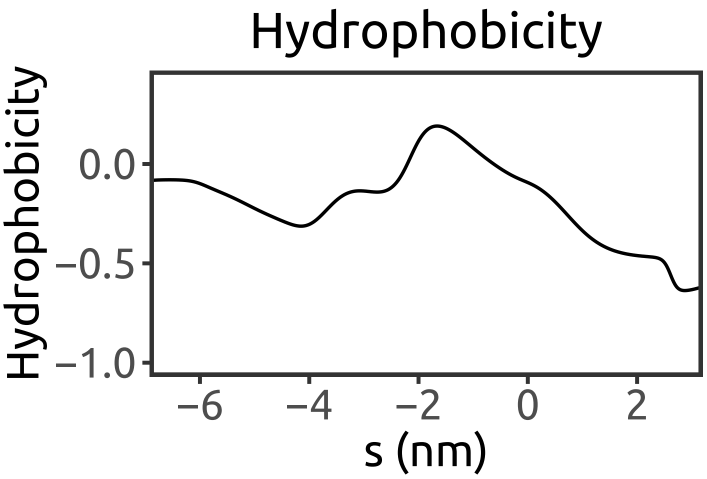


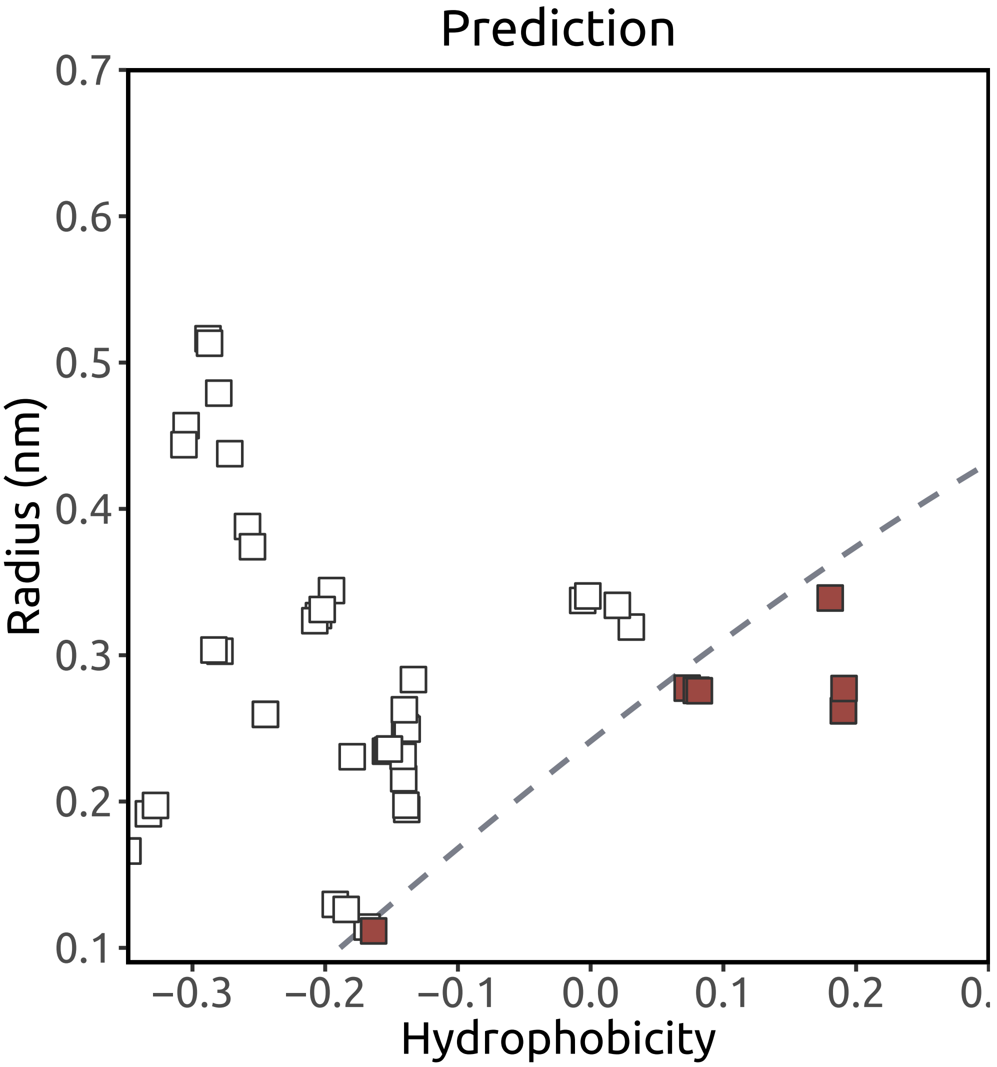


Heuristic score:
0.35 (n = 9)

Simulation result:
barrier to water

# Kir3.2 (PDB ID: 3SYO)

*Mus musculus*

X–ray (3.54 Å)

Whorton & Mackinnon, 2011

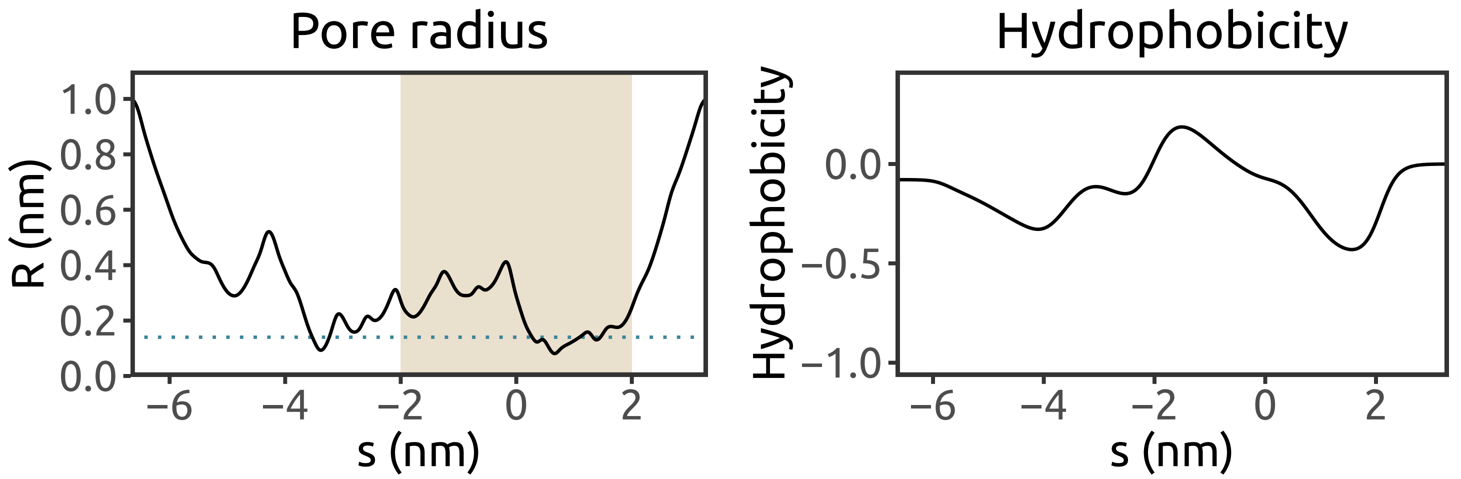


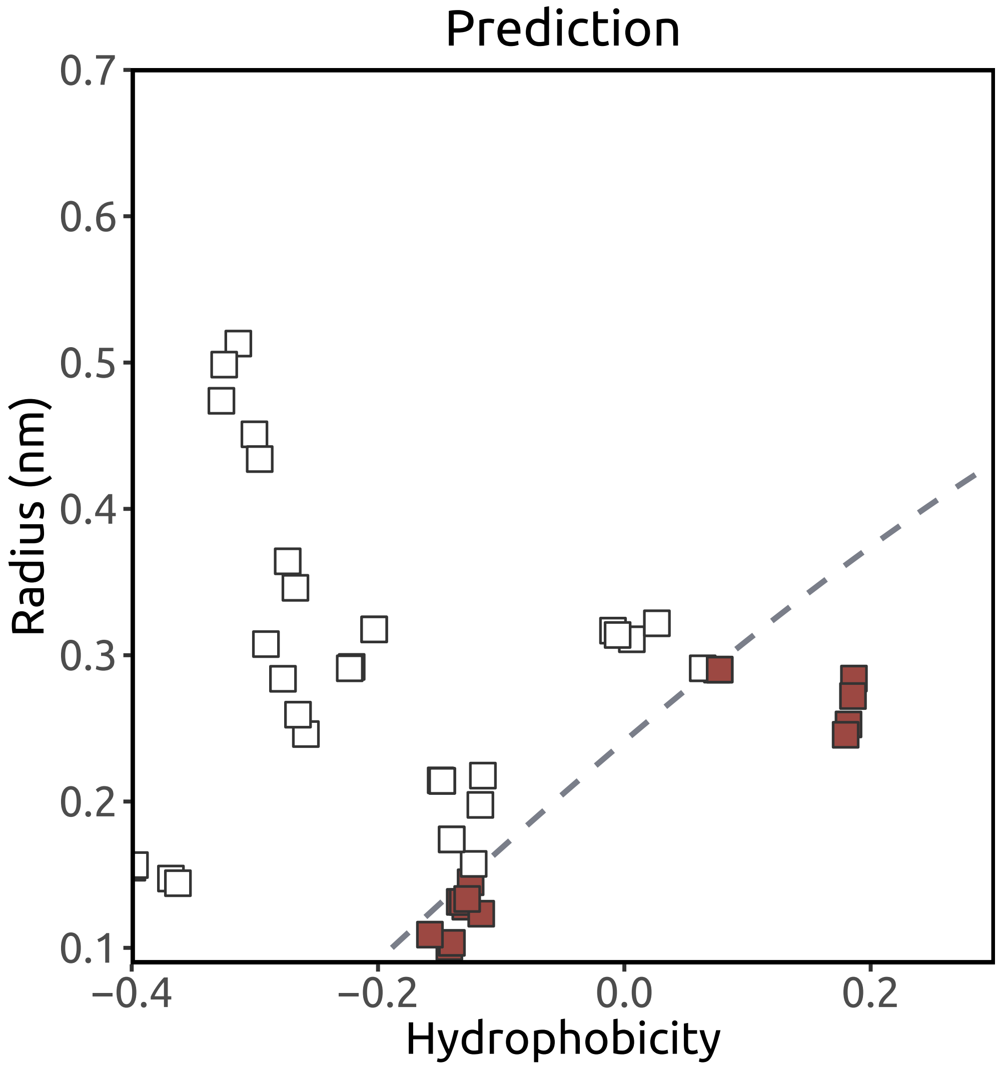


Heuristic score:

0.5 (n = 16)

Simulation result:

hydrated channel

# Kir6.2 (PDB ID: 6BAA)

*Rattus norvegicus*
cryo−EM (3.63 Å)

Martin et al., 2017

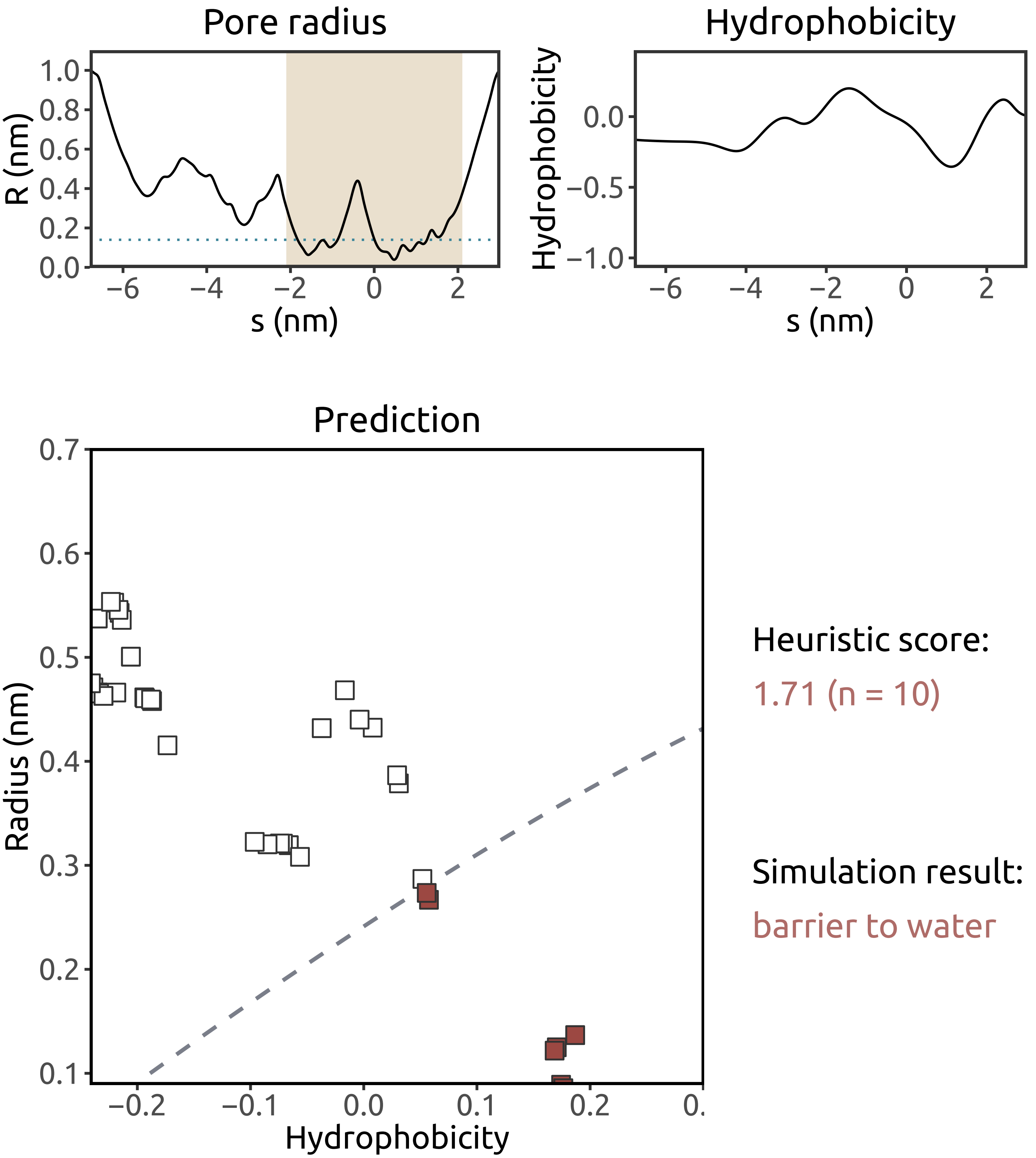


Heuristic score:

1.71 (n = 10)

Simulation result:

barrier to water

# Kir6.2 (PDB ID: 6C3O)

*Homo sapiens*
cryo–EM (3.9 Å)

Lee et al., 2017

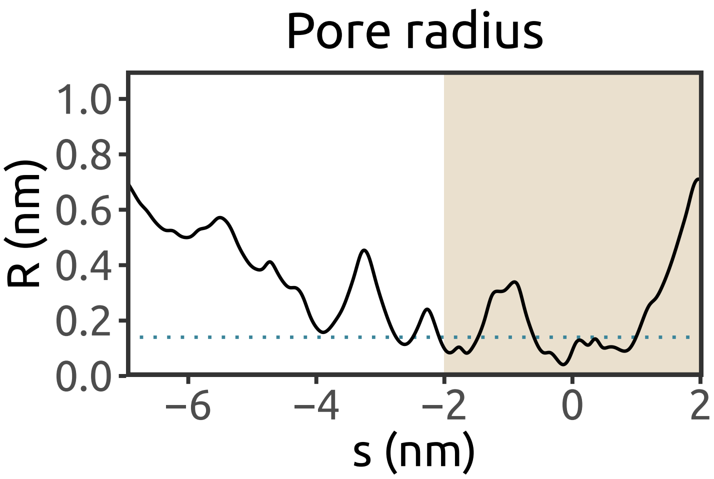


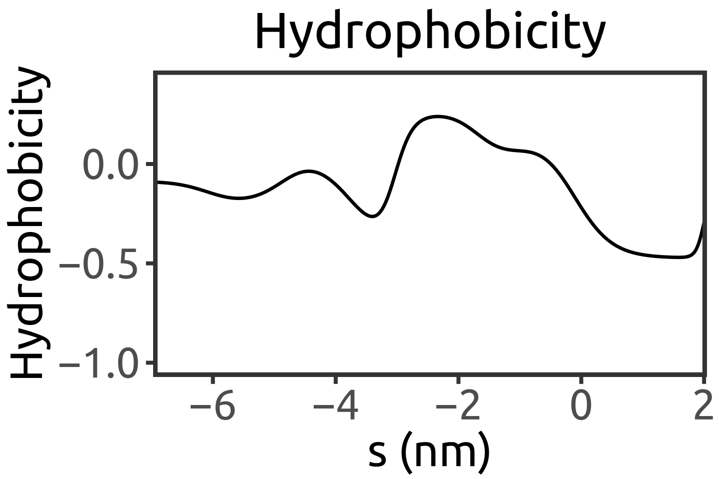


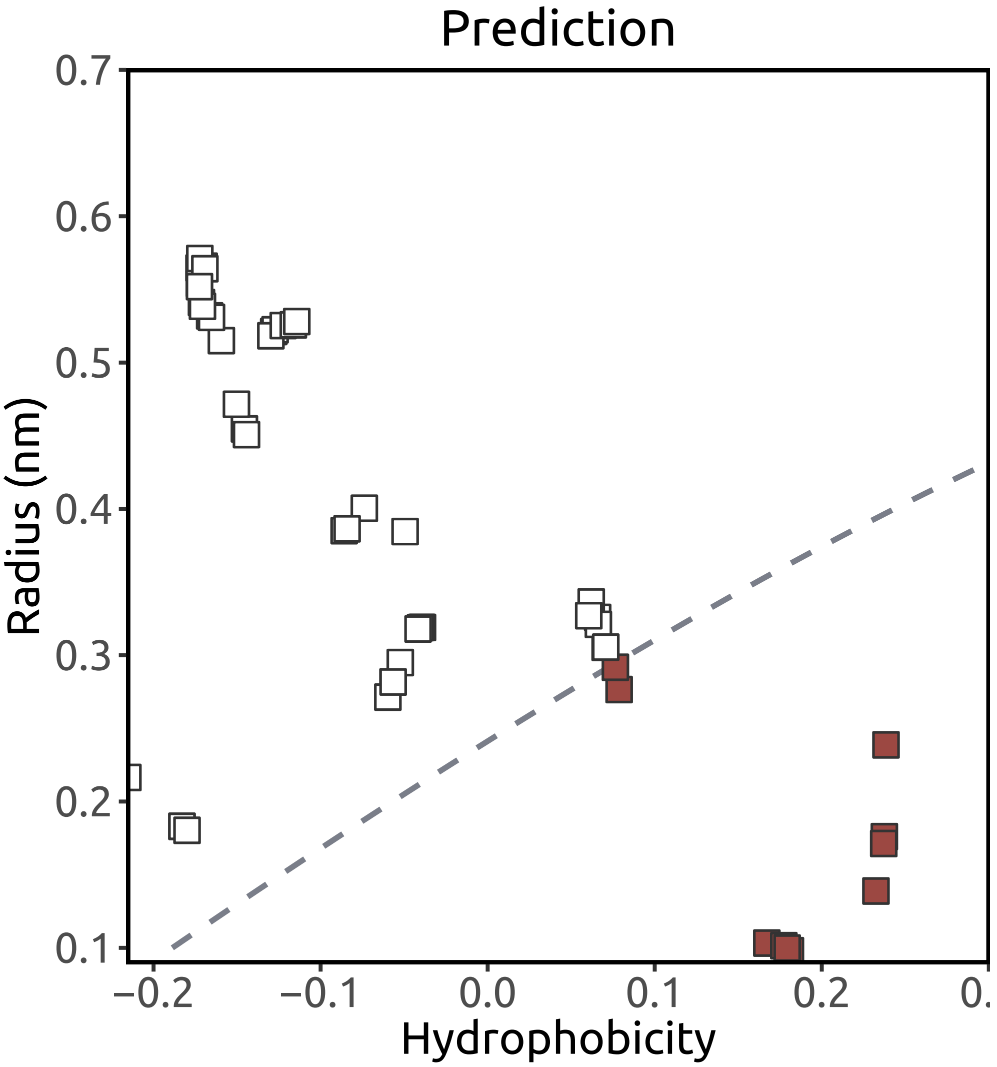


Heuristic score:
1.62 (n = 10)

Simulation result:
barrier to water

# KirBac1.1 (PDB ID: 2WLL)

*Burkholderia pseudomallei*
X–ray (3.65 Å)

Kuo et al., 2003

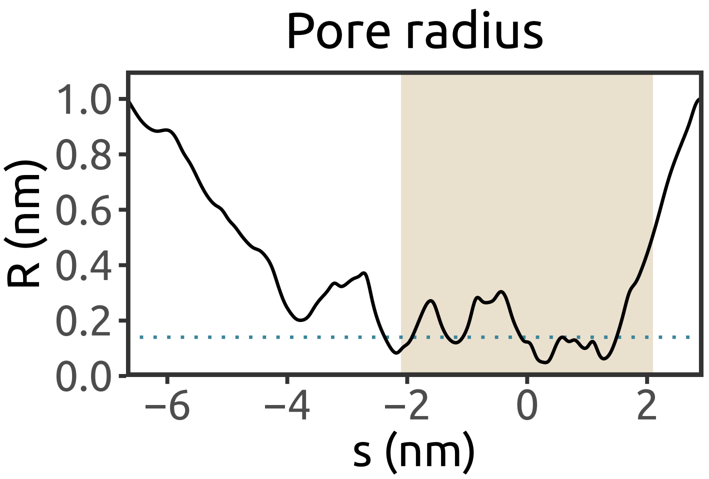


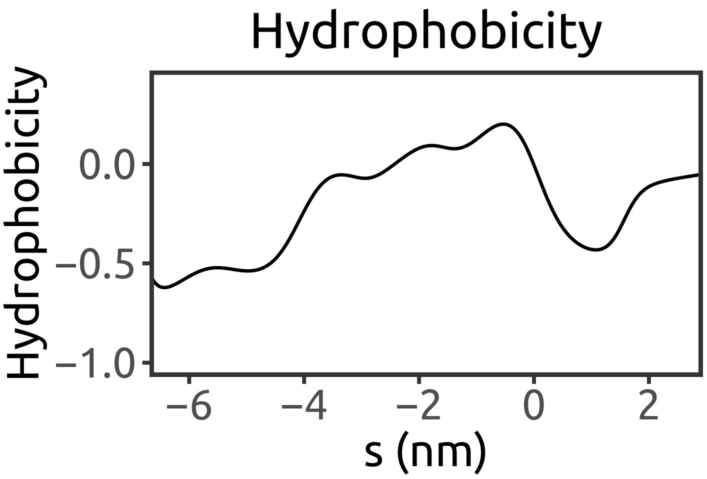


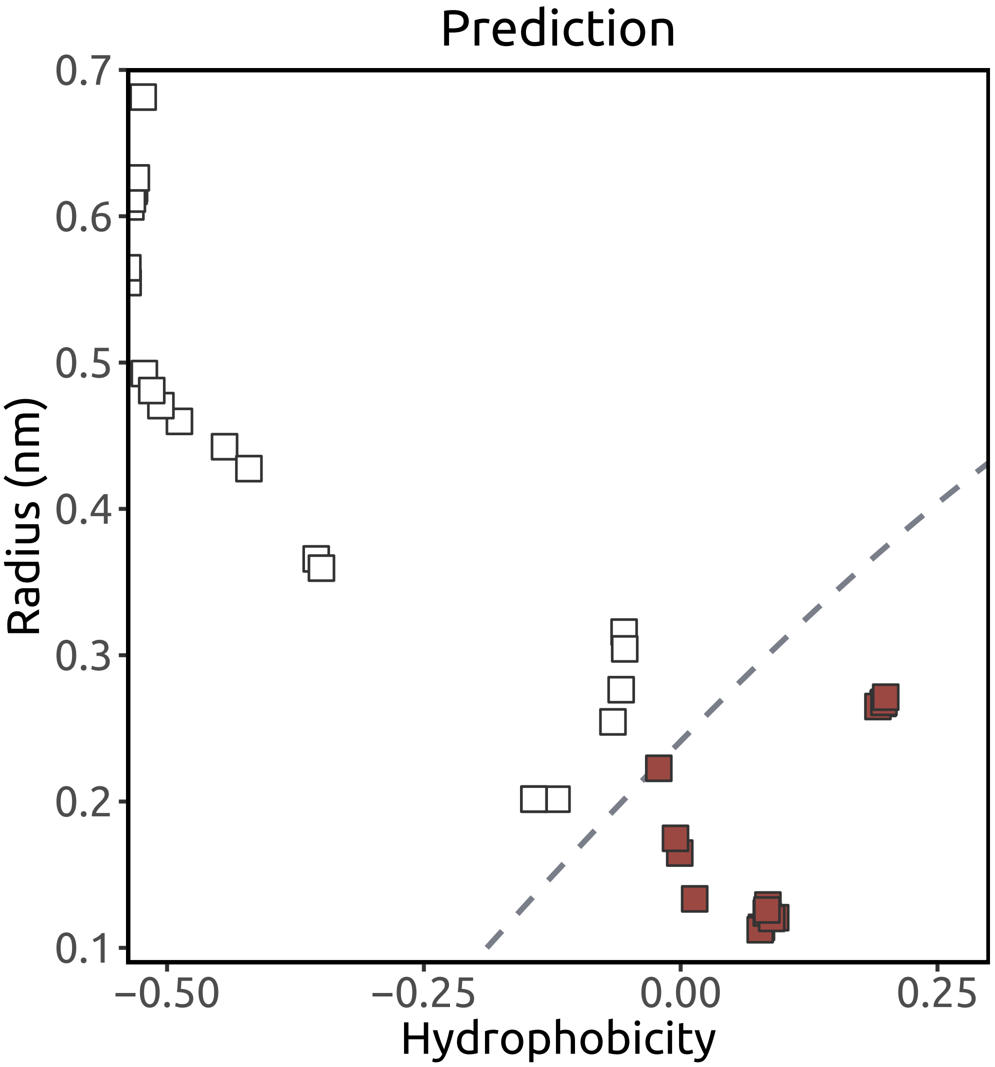


Heuristic score:
1.74 (n = 16)

Simulation result:
barrier to water

# KirBac1.3 (PDB ID: 2QKS)

*M. musculus B. xenovornas*
X−ray (2.2 Å)

Nishida et al., 2007

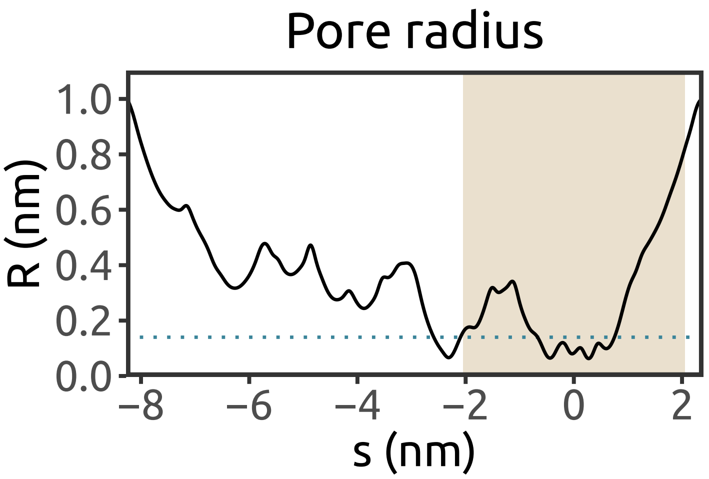


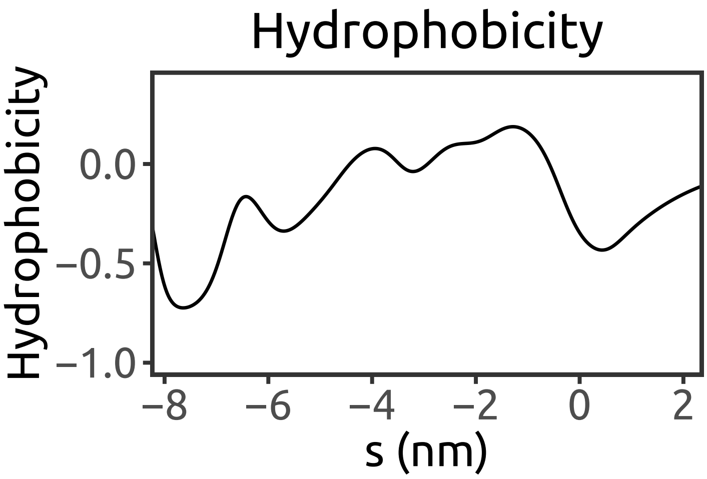


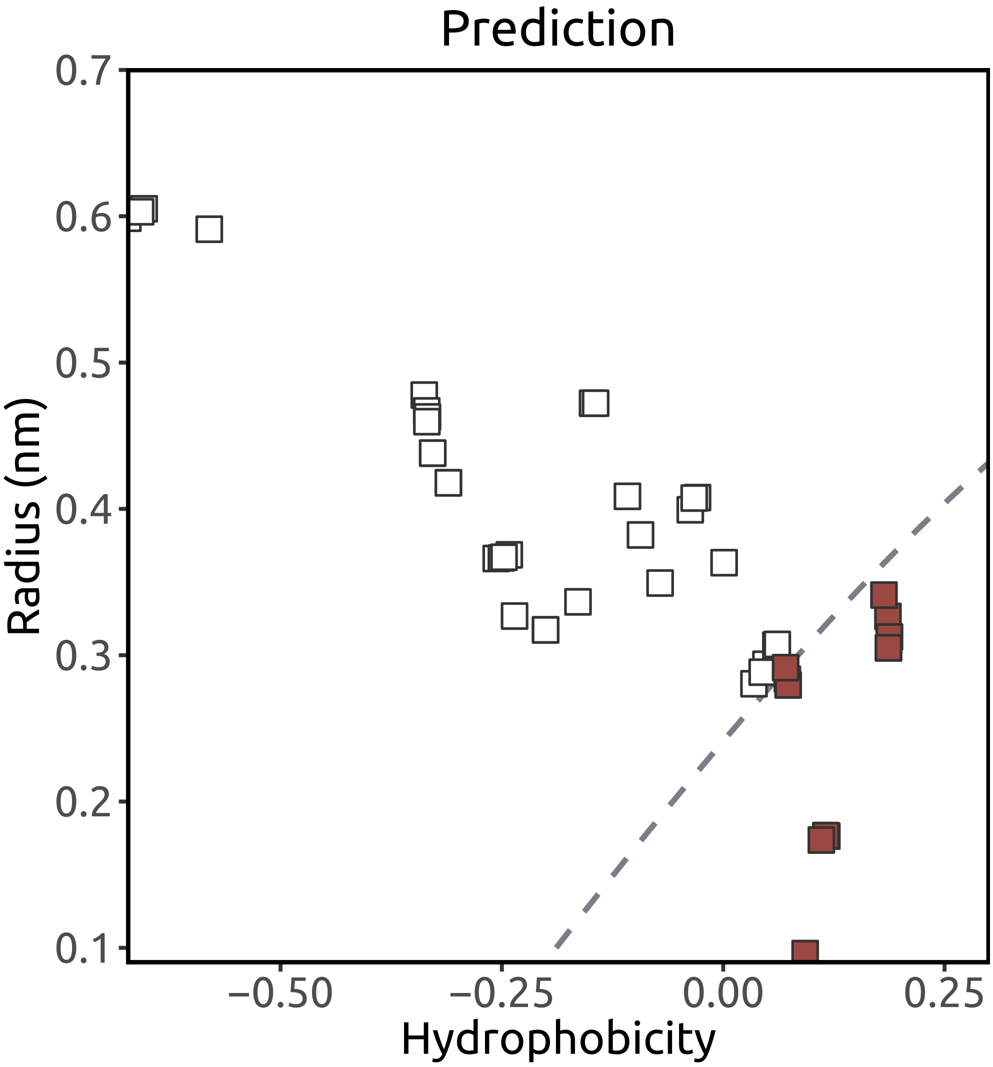


Heuristic score:
1.49 (n = 17)

Simulation result:
barrier to water

# KirBac3.1 (PDB ID: 2WLH)

*M. magnetotacticum*
X–ray (3.28 Å)

Clarke et al., 2010

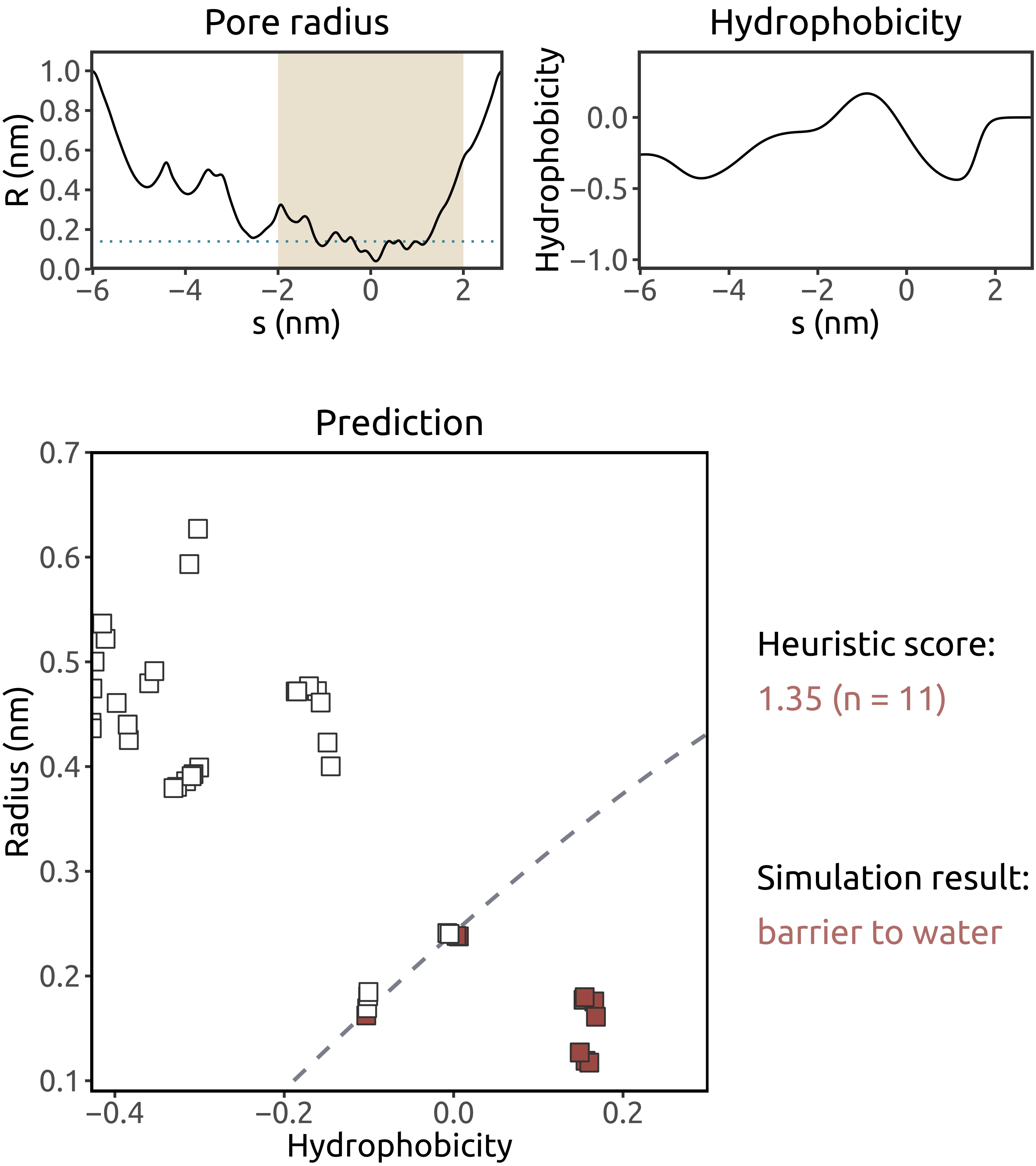


Heuristic score:
1.35 (n = 11)

Simulation result:
barrier to water

# KirBac3.1 (PDB ID: 2WLI)

*M. magnetotacticum*
X–ray (3.09 Å)

Clarke et al., 2010

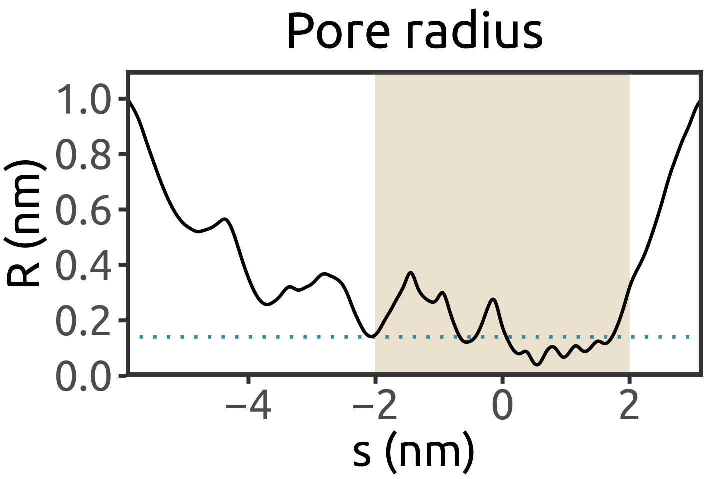


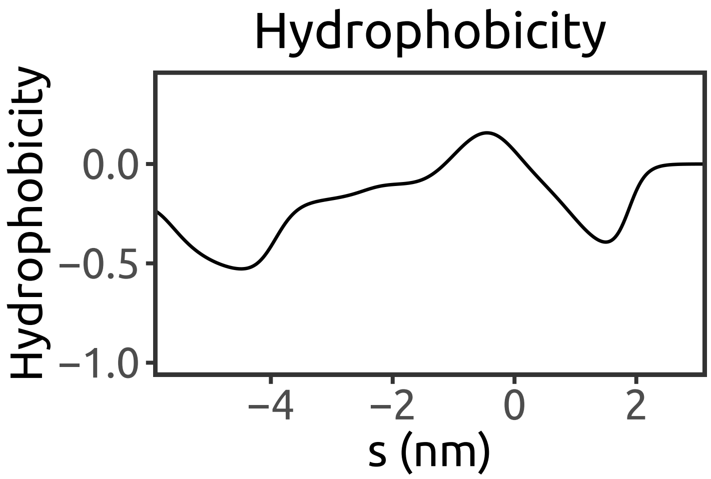


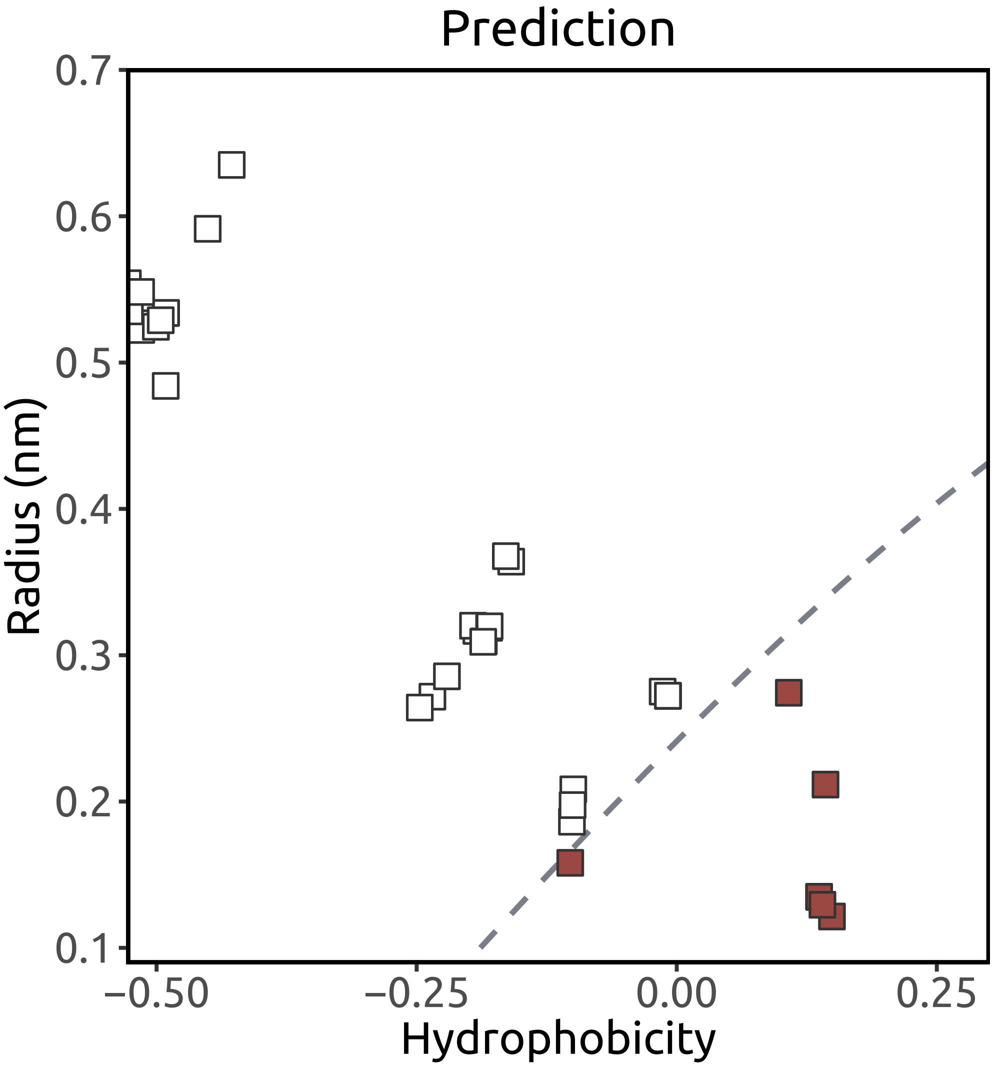


Heuristic score:
0.84 (n = 7)

Simulation result:
barrier to water

# KirBac3.1 (PDB ID: 2WLJ)

*M. magnetotacticum*
X–ray (2.6 Å)

Clarke et al., 2010

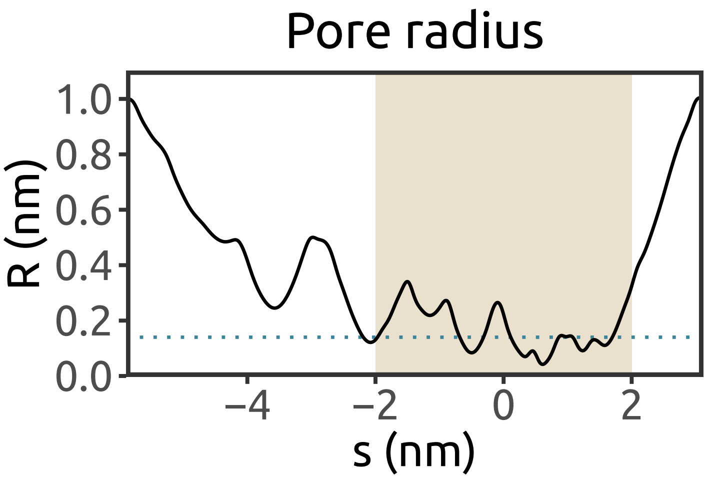


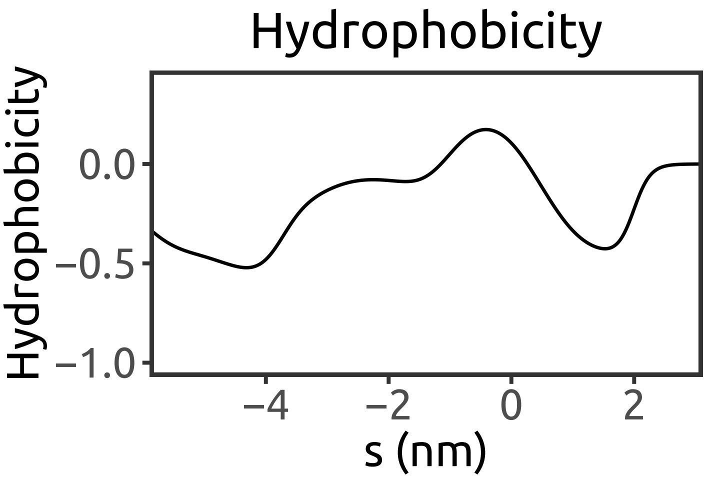


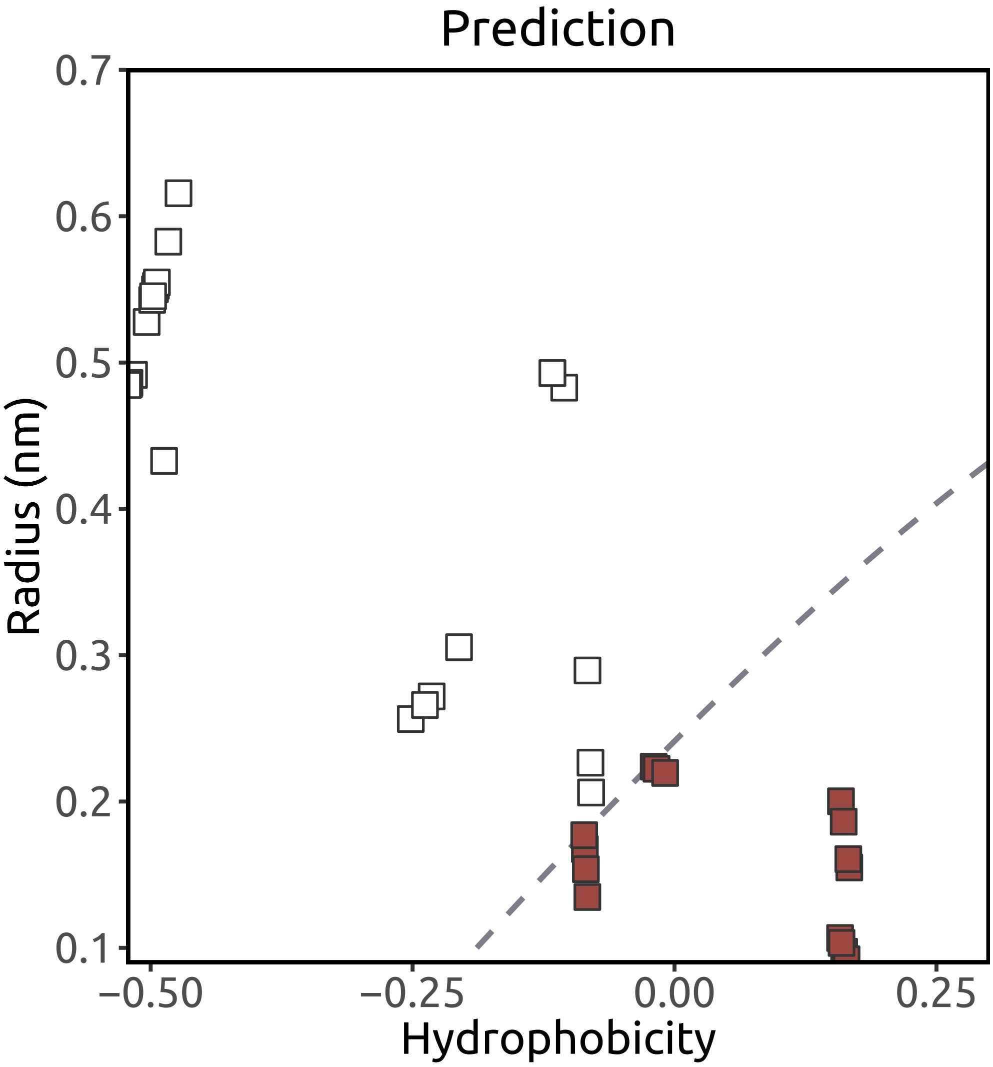


Heuristic score:
1.52 (n = 16)

Simulation result:
barrier to water

# MthK (PDB ID: 1LNQ)

*M. thermautotrophicus*
X–ray (3.3 Å)

Jiang et al., 2002

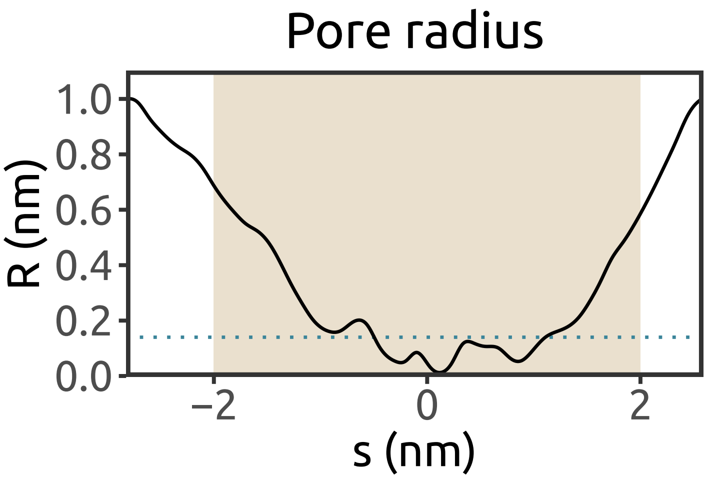


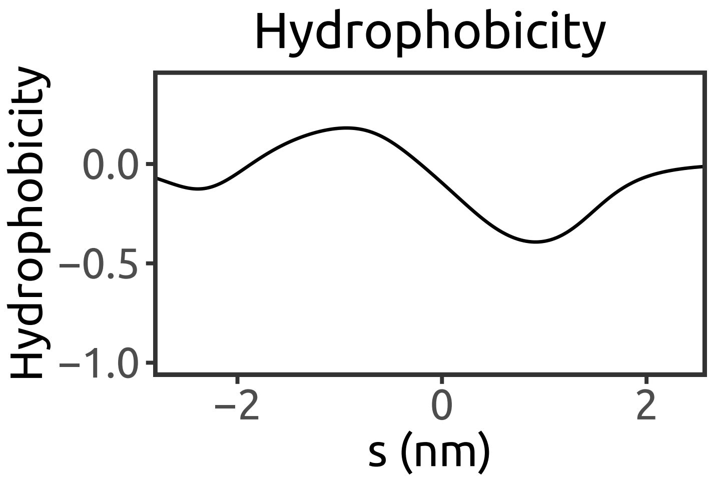


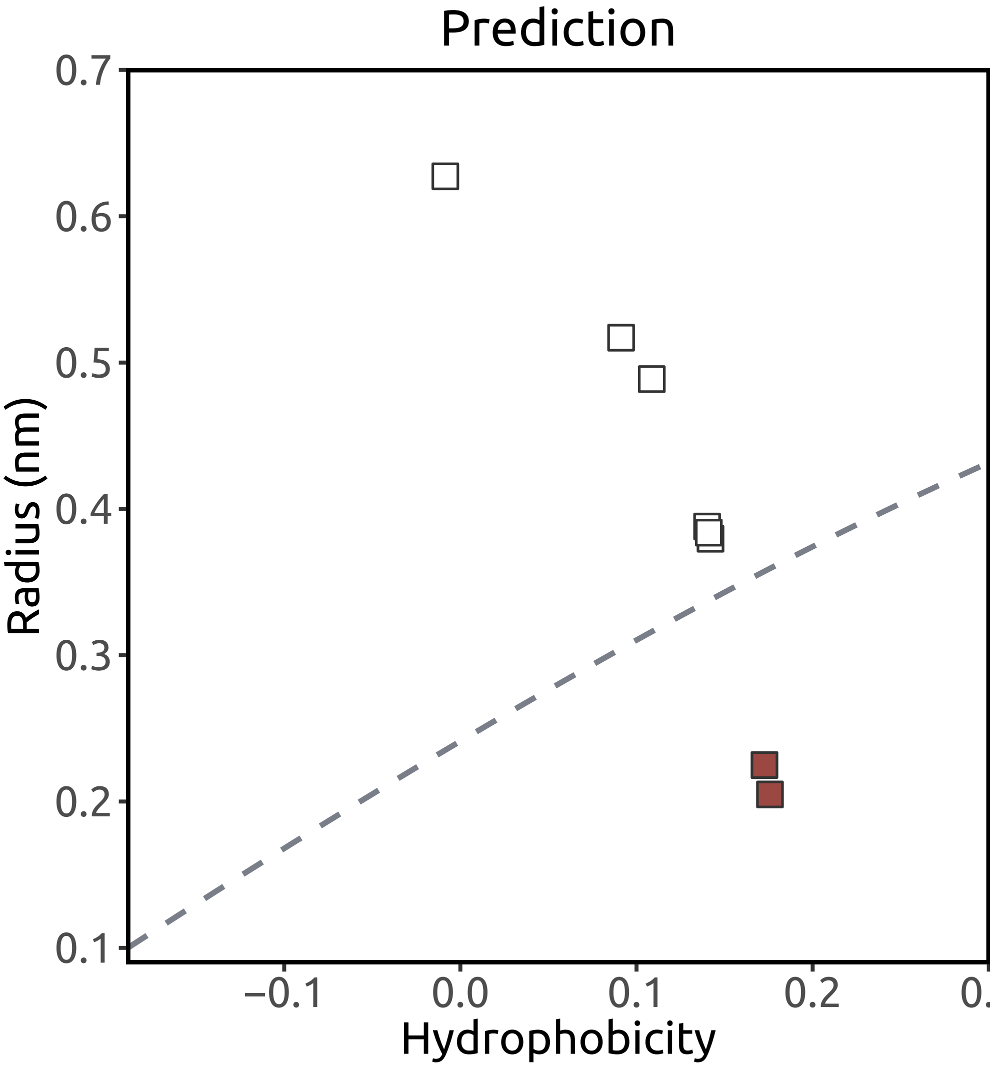


Heuristic score:
0.24 (n = 2)

Simulation result:
hydrated channel

# NaK (PDB ID: 2AHY)

*Bacillus cereus*

X–ray (2.4 Å)

Shi et al., 2006

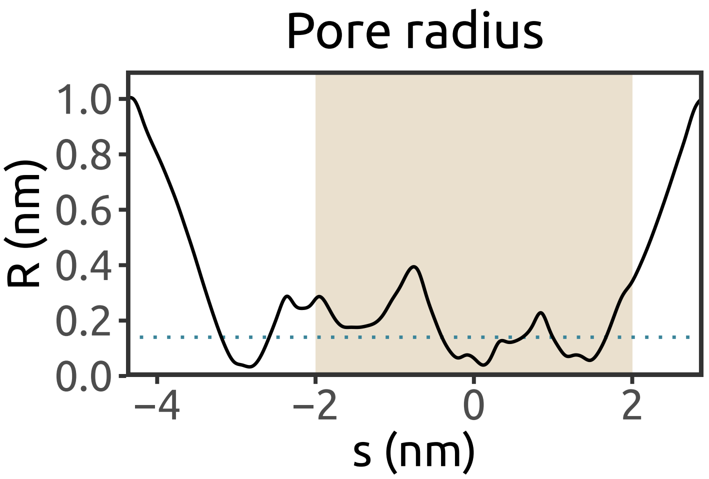


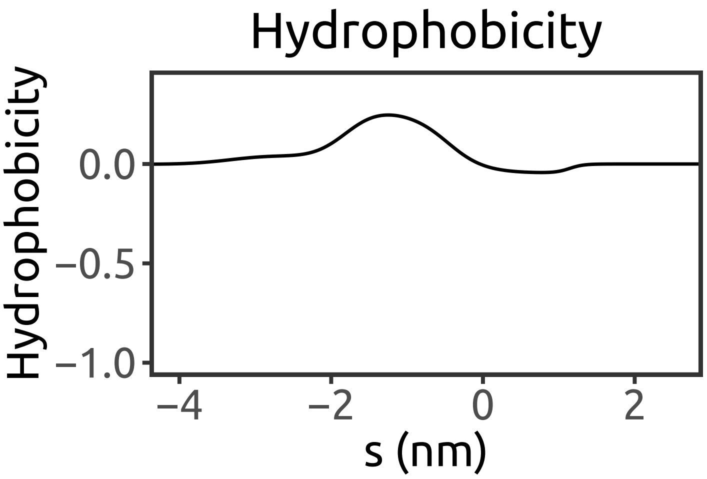


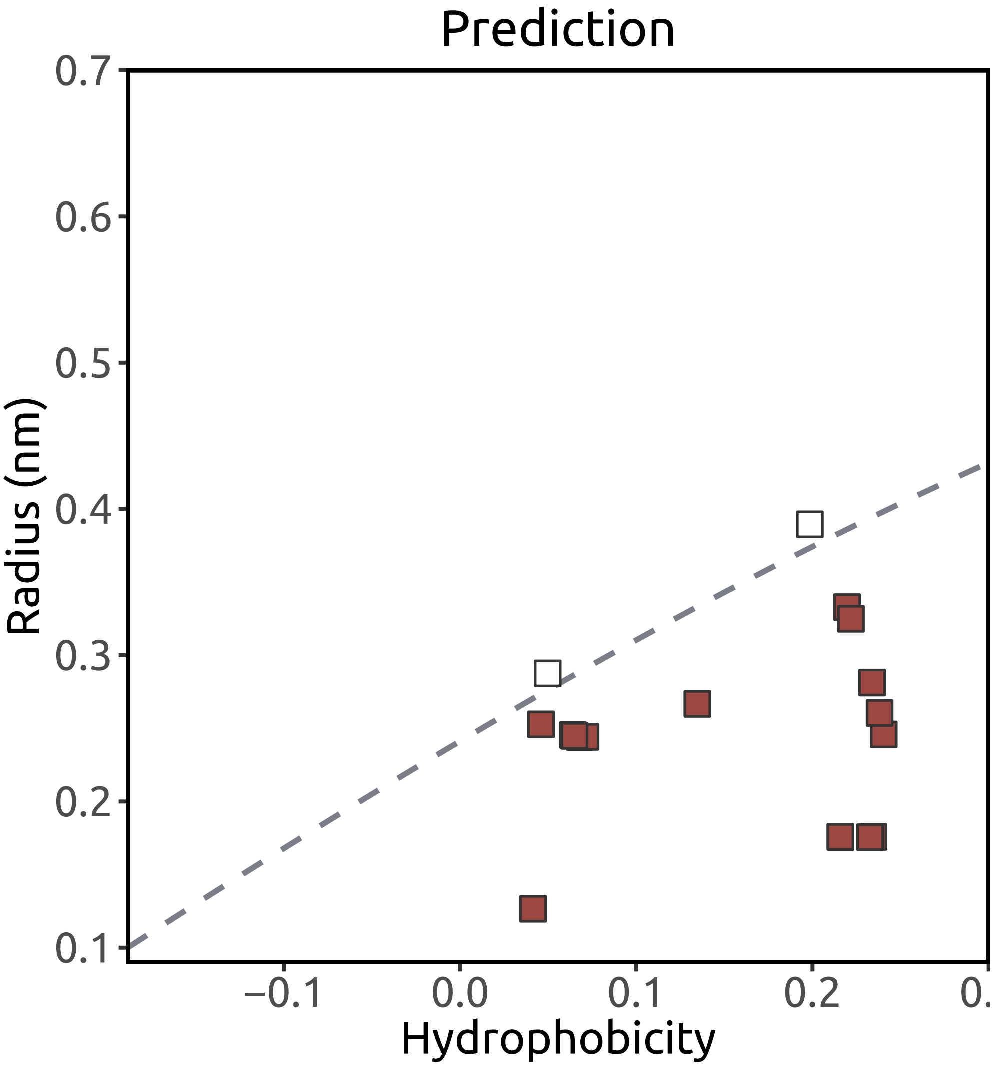


Heuristic score:

2.24 (n = 20)

Simulation result:

barrier to water

# NaK (PDB ID: 3E86)

*Bacillus cereus*

X−ray (1.6 Å)

Alam & Jiang, 2009

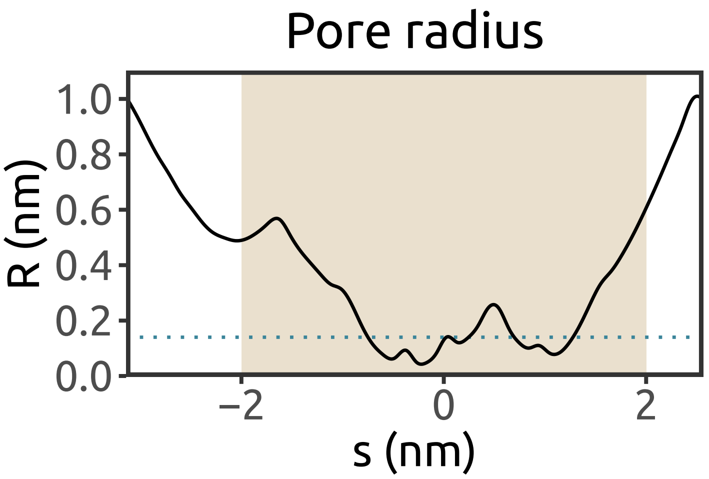


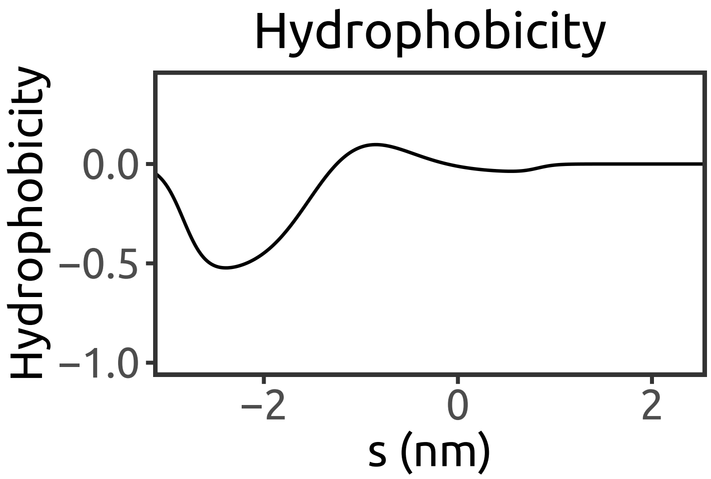


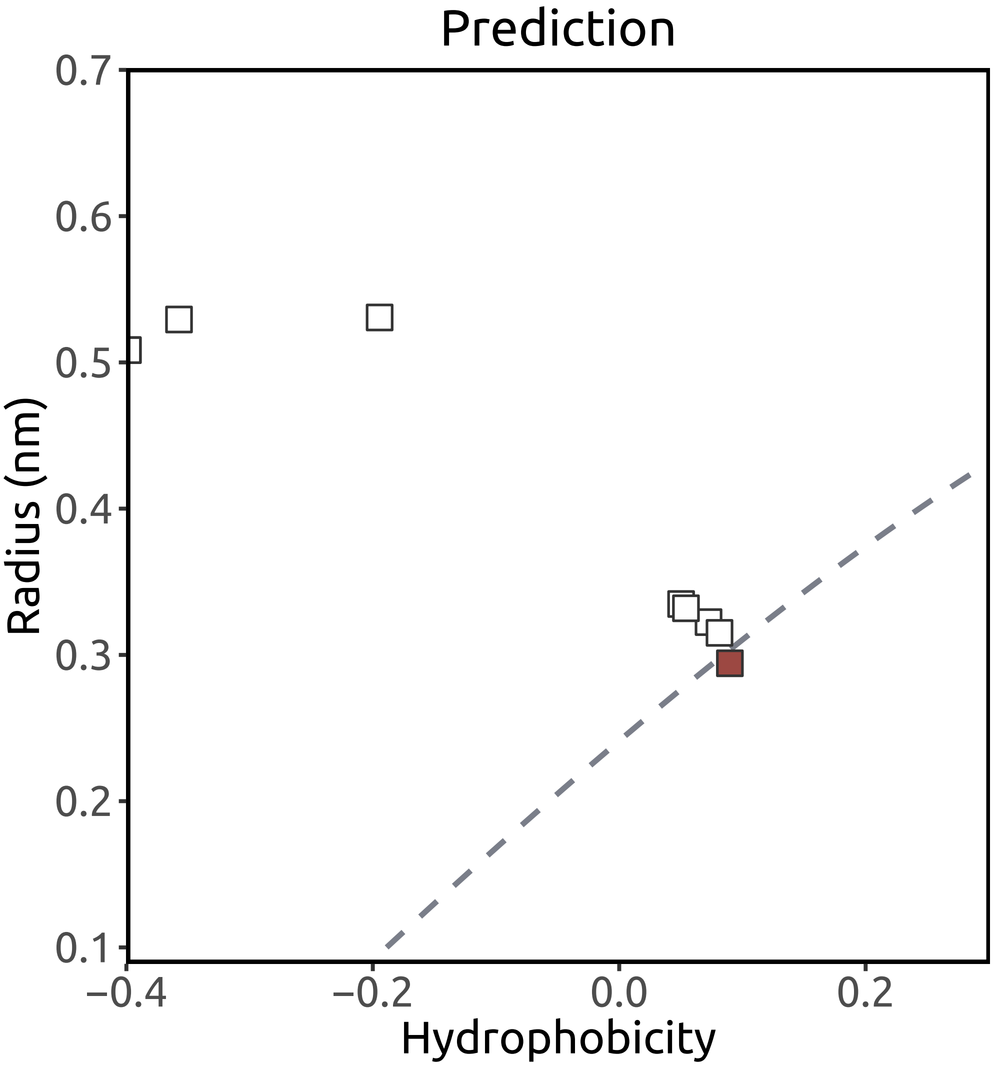


Heuristic score:

0.01 (n = 1)

Simulation result:

hydrated channel

# NaKNav (PDB ID: 3VOU)

*B. mycoides S. pontiacus*

X–ray (3.2 Å)

Irie et al., 2012

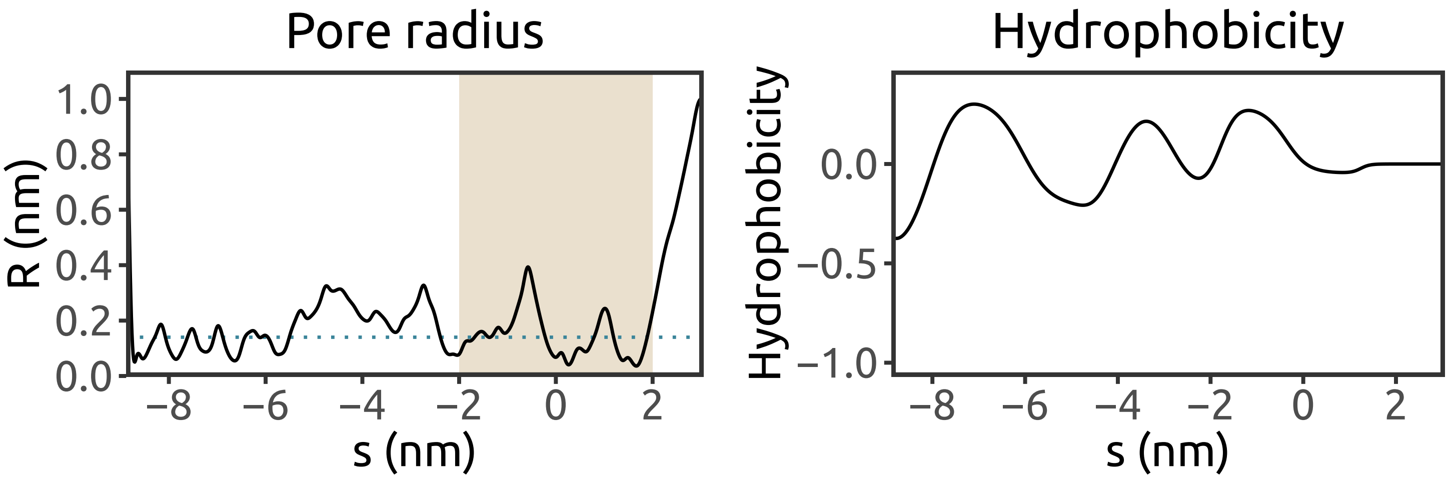


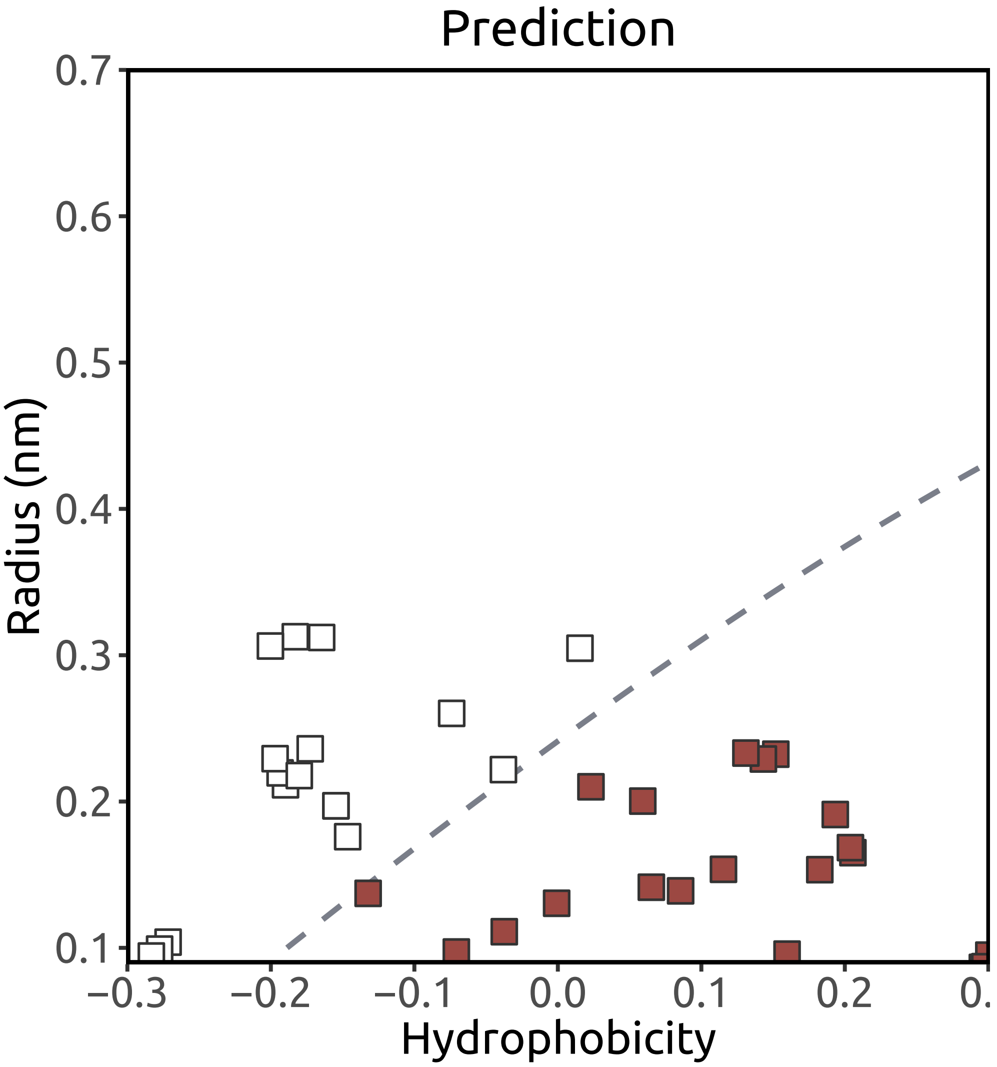


Heuristic score:

4.81 (n = 31)

Simulation result:

barrier to water

# KCNQ1 (PDB ID: 5VMS)

*Xenopus laevis*
cryo–EM (3.7 Å)

Sun & MacKinnon, 2017

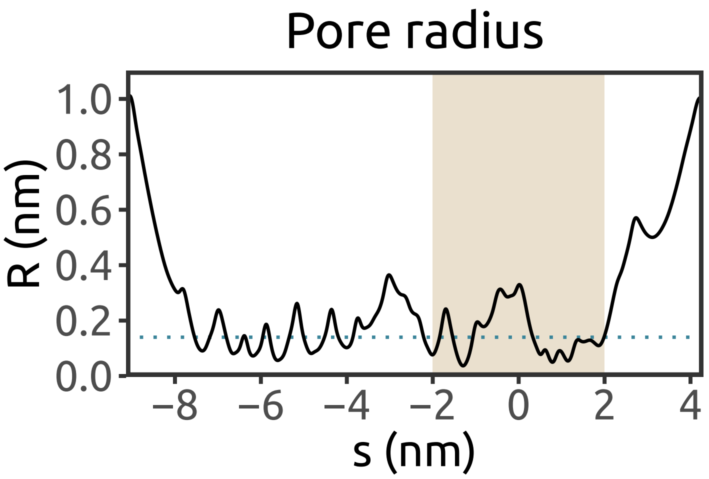


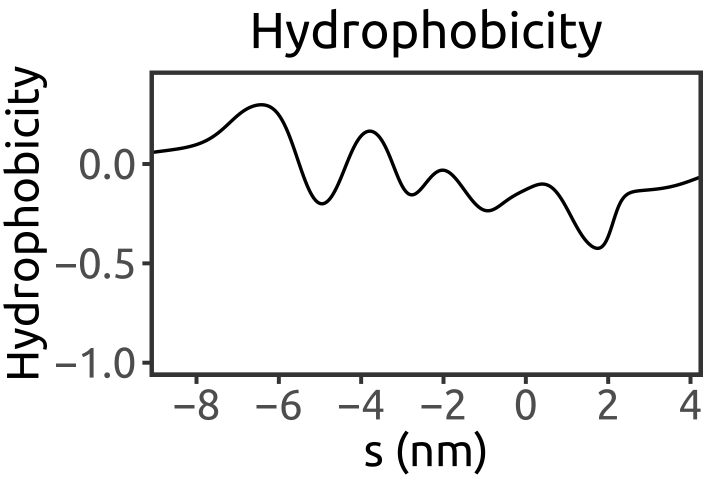


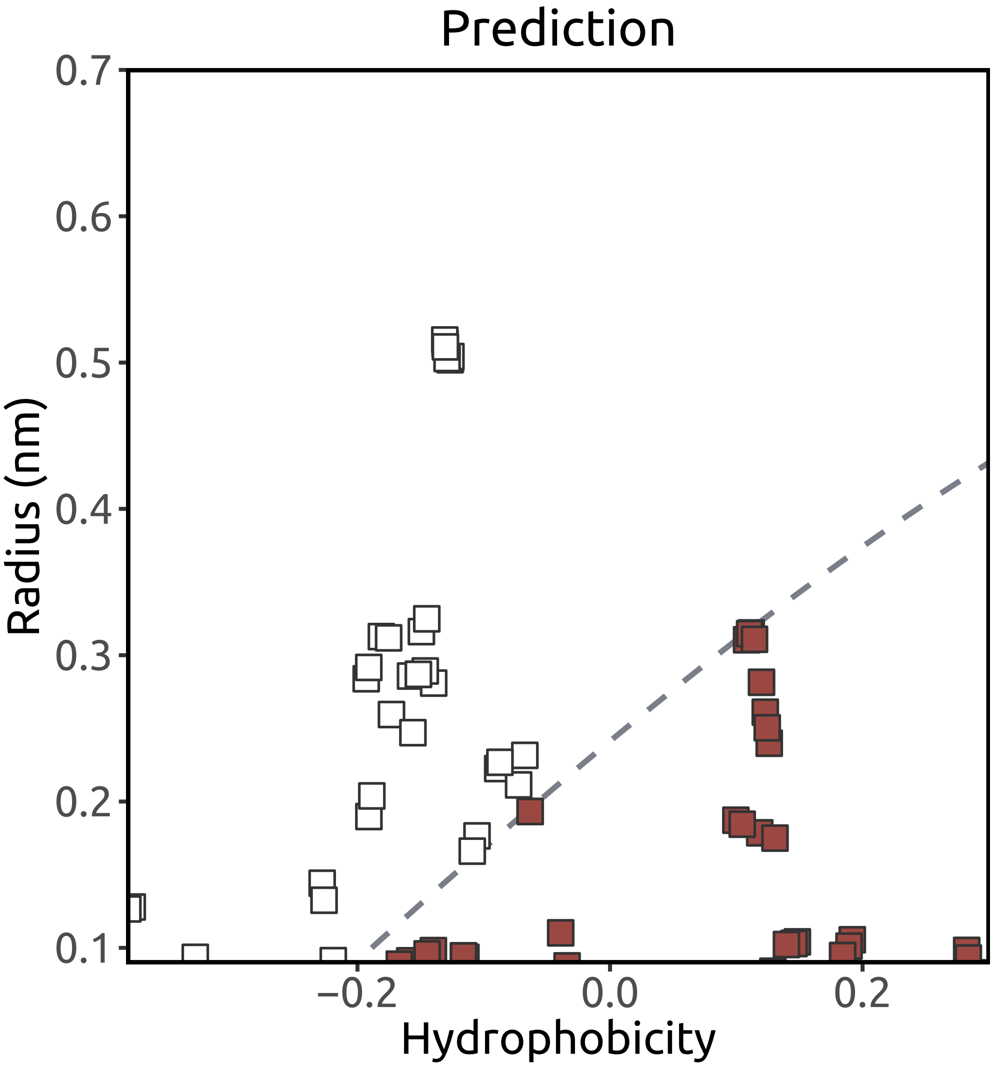


Heuristic score:

6.44 (n = 50)

Simulation result:

barrier to water

# Kv10.1 (PDB ID: 5K7L)

*Rattus norvegicus*
cryo–EM (3.78 Å)

Whicher & MacKinnon, 2016

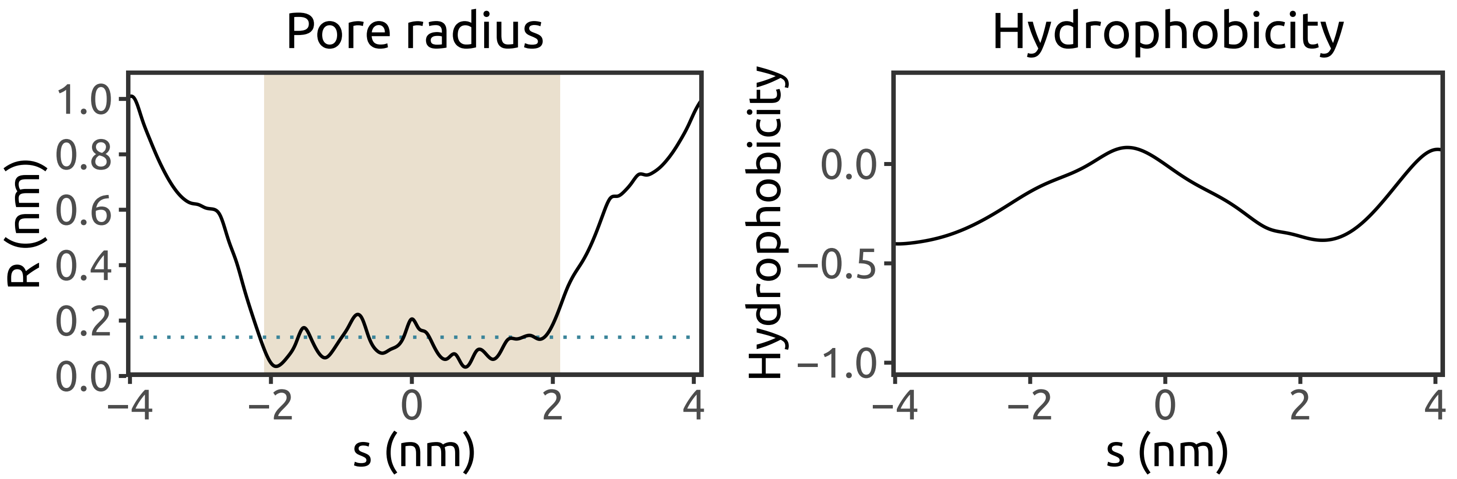


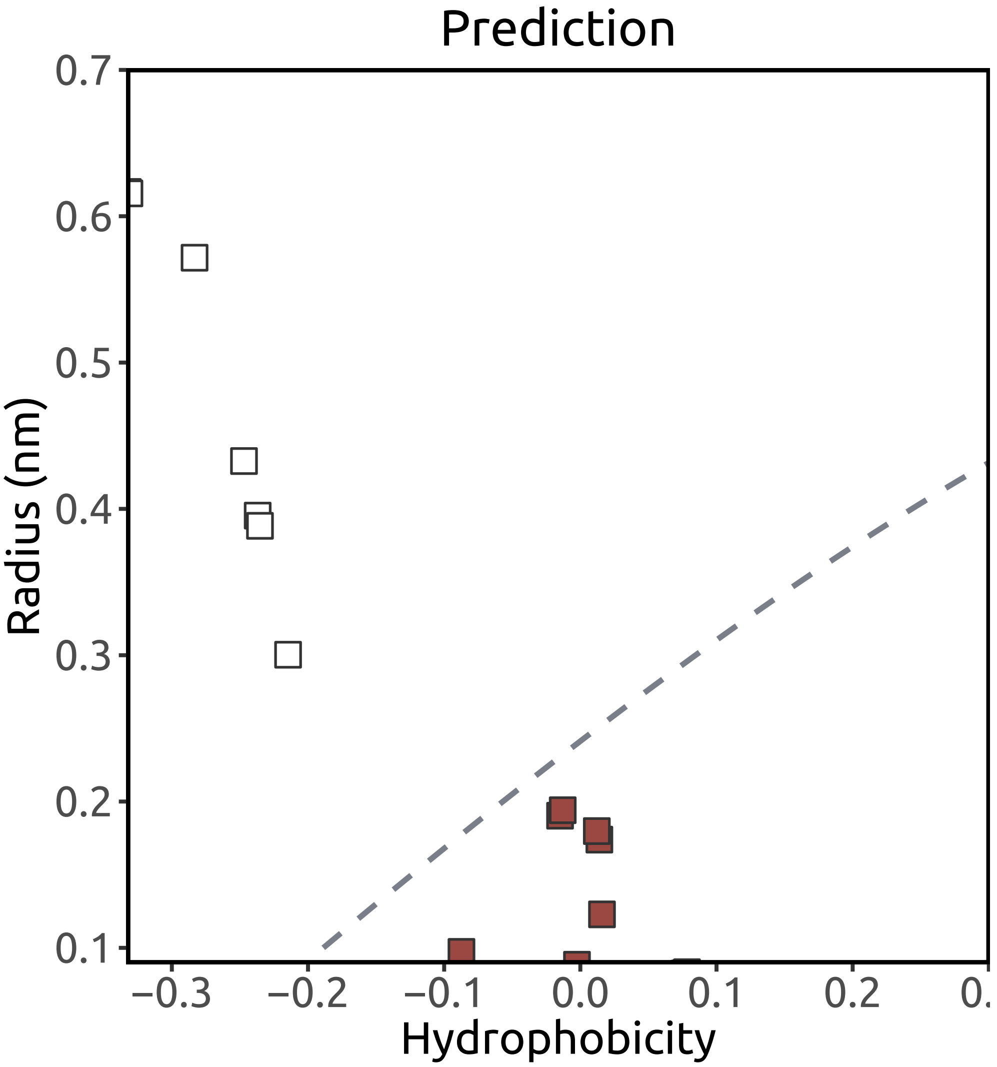


Heuristic score:

1.69 (n = 16)

Simulation result:

barrier to water

# Kv11.1 (PDB ID: 5VA1)

*Homo sapiens*
cryo−EM (3.7 Å)

Wang & MacKinnon, 2017

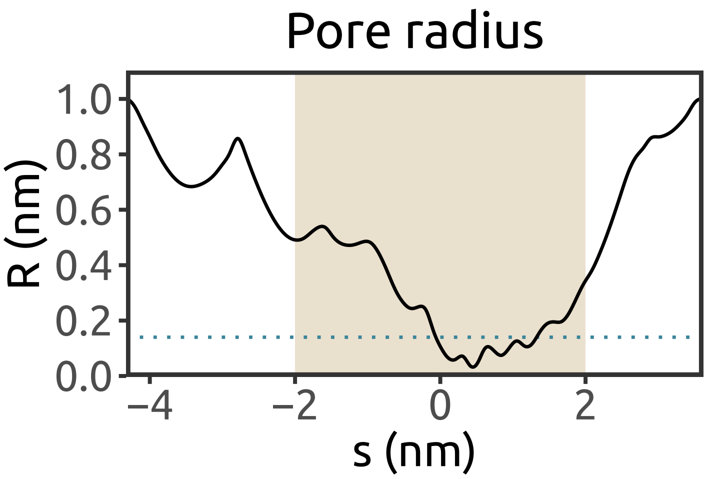


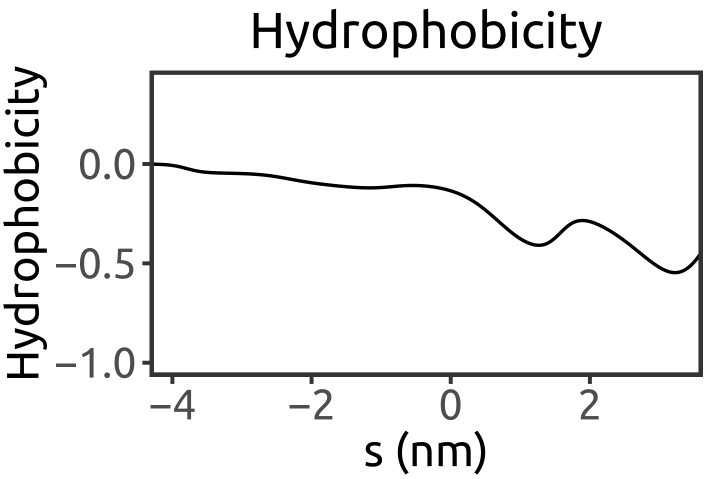


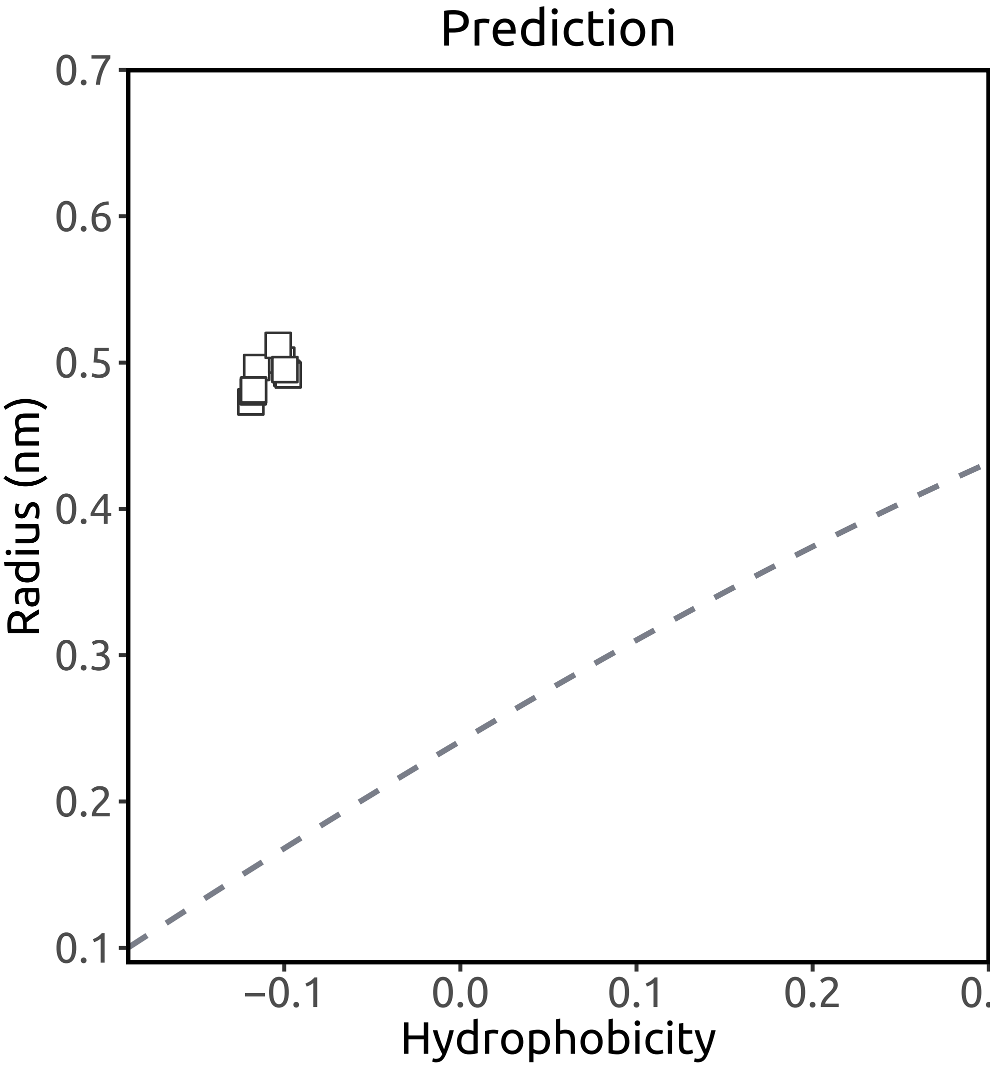


Heuristic score:
0 (n = 0)

Simulation result:
hydrated channel

# Kv1.2 (PDB ID: 2A79)

*Rattus norvegicus*
X−ray (2.9 Å)

Long et al., 2005

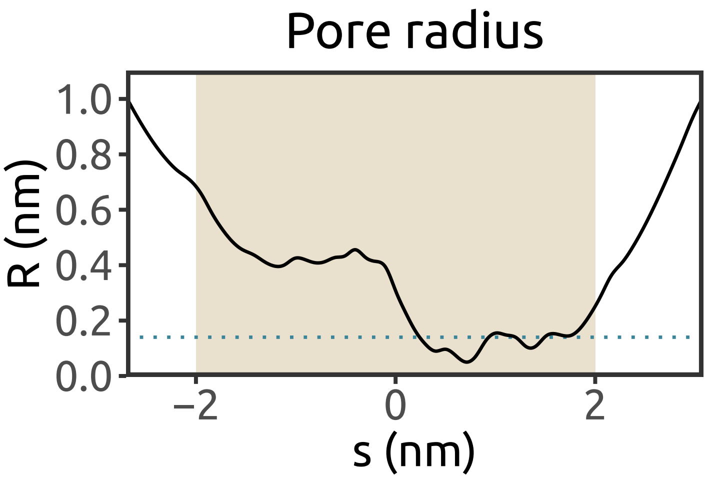


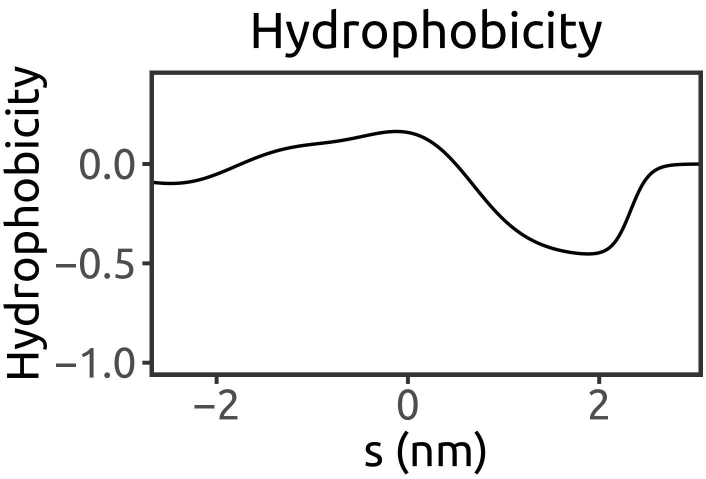


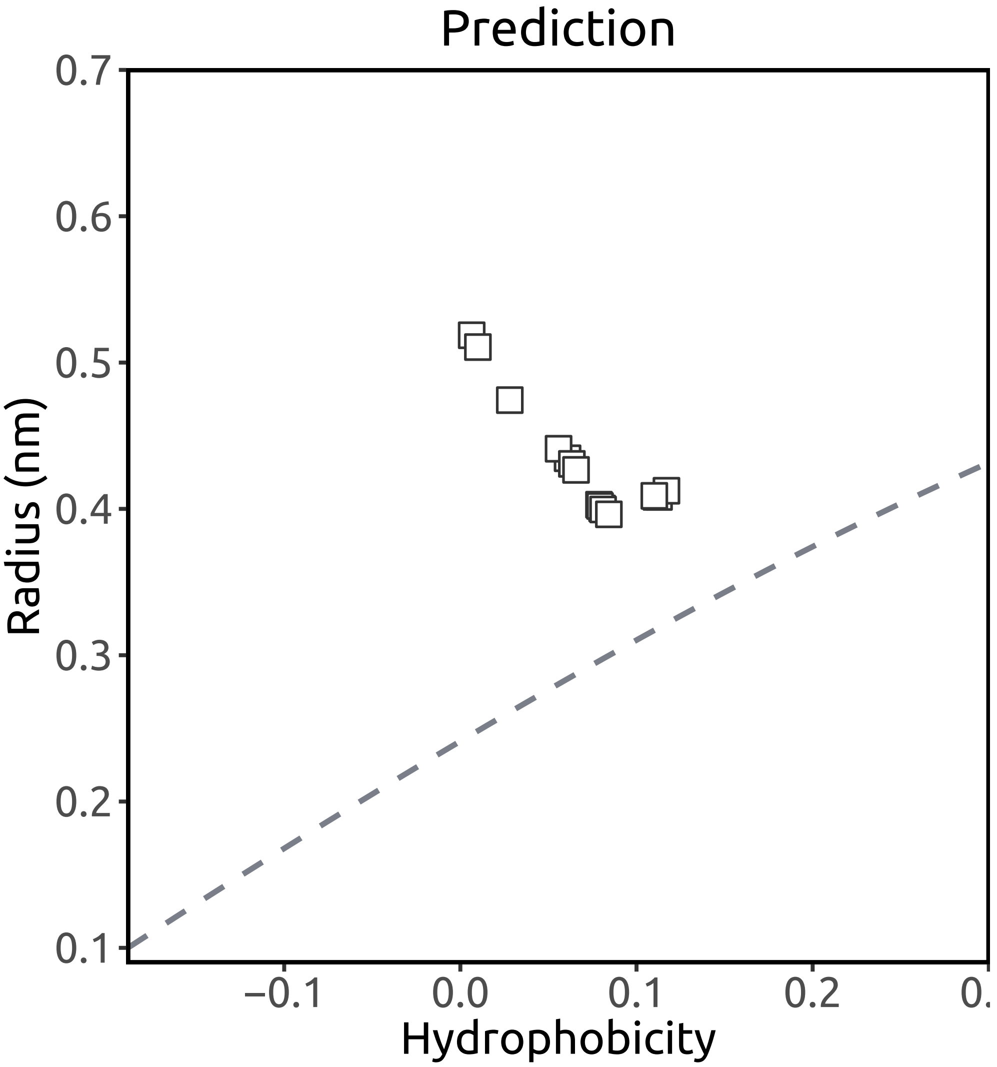


Heuristic score:
0 (n = 0)

Simulation result:
hydrated channel

# Kv1.2 (PDB ID: 2R9R)

*Rattus norvegicus*
X–ray (2.4 Å)

Long et al., 2007

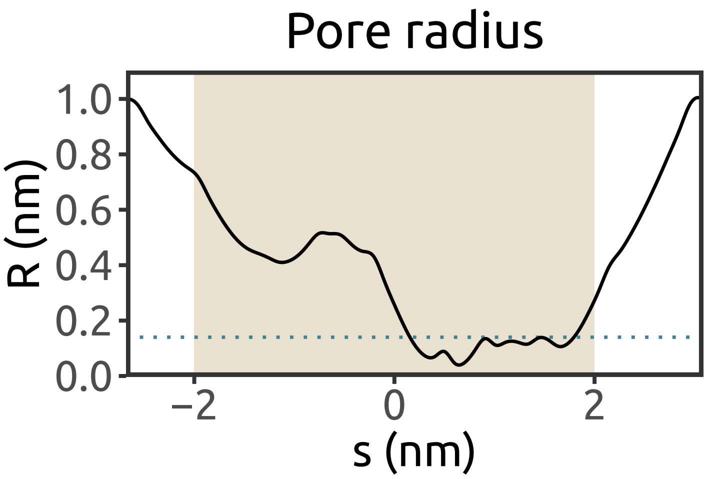


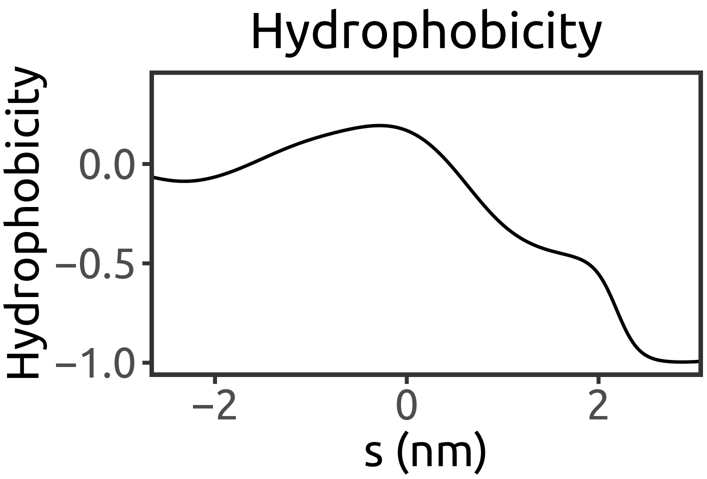


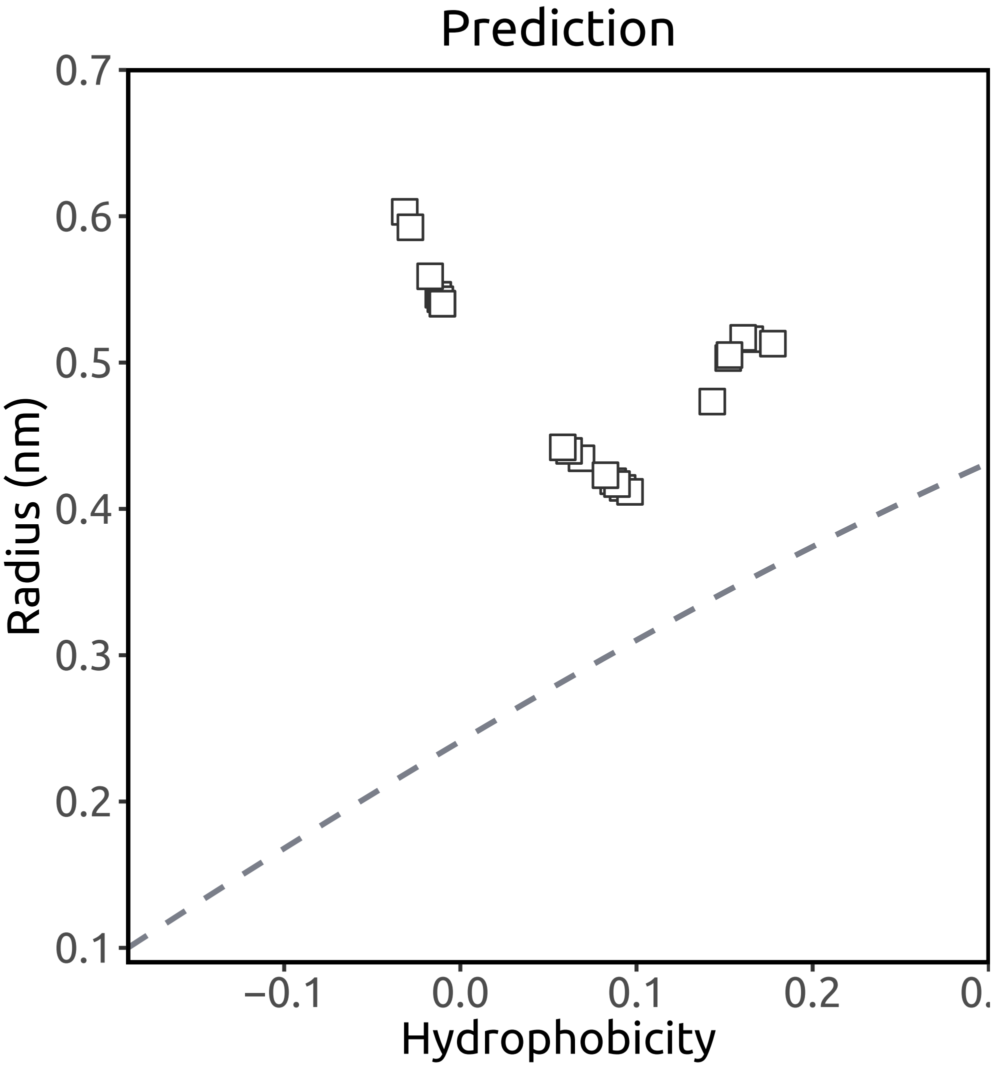


Heuristic score:
0 (n = 0)

Simulation result:
hydrated channel

# Kv1.2 (PDB ID: 3LUT)

*Rattus norvegicus*
X–ray (2.9 Å)

Chen et al., 2010

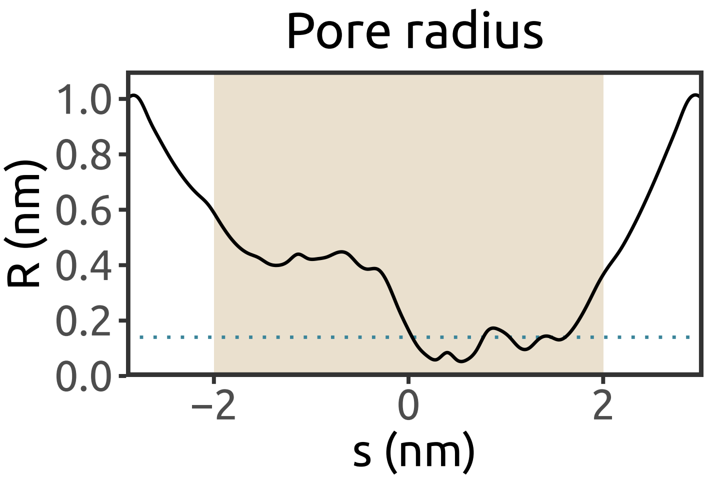


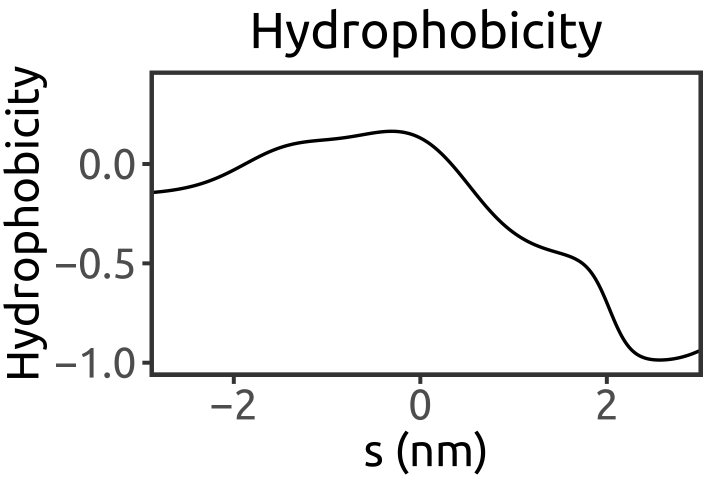


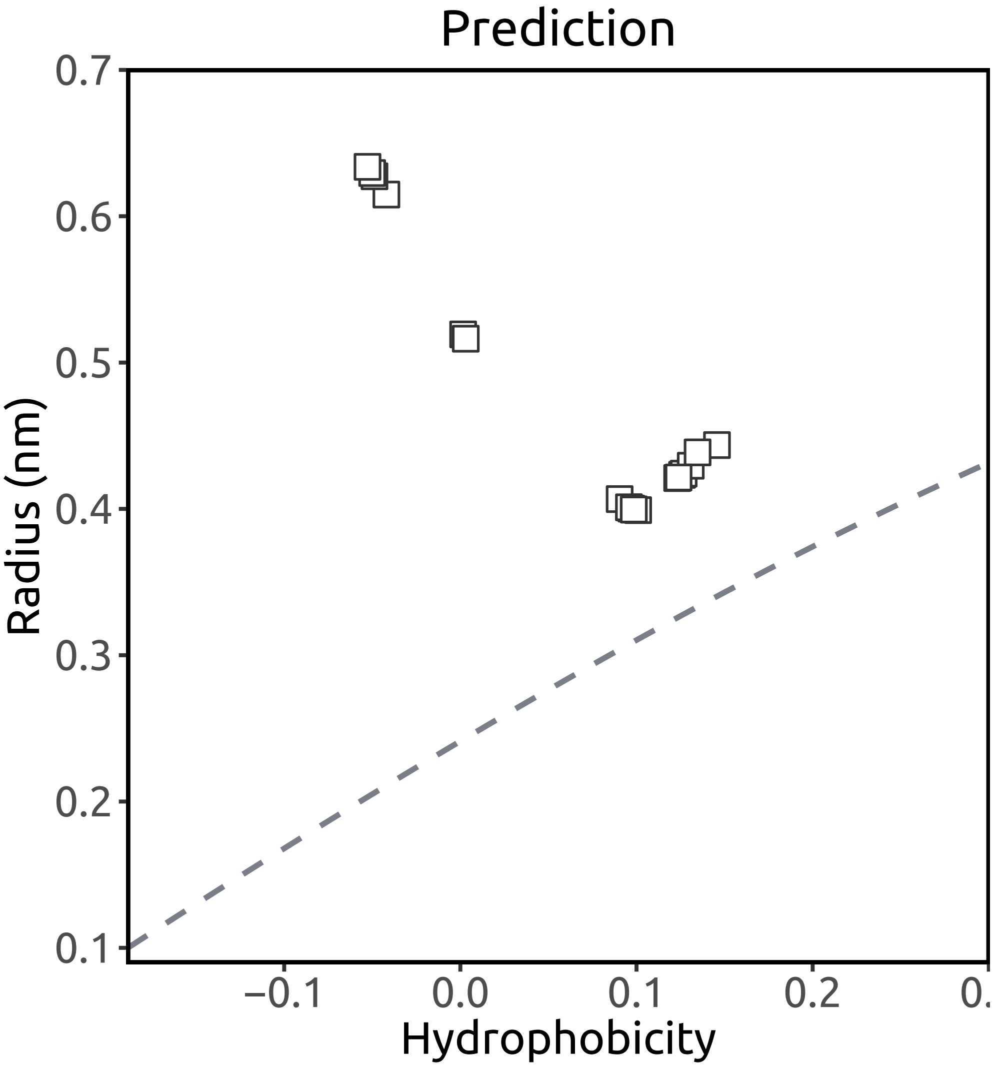


Heuristic score:
0 (n = 0)

Simulation result:
hydrated channel

# KvAp (PDB ID: 1ORQ)

*Aeropyrum pernix*
X–ray (3.2 Å)

Jiang et al., 2003

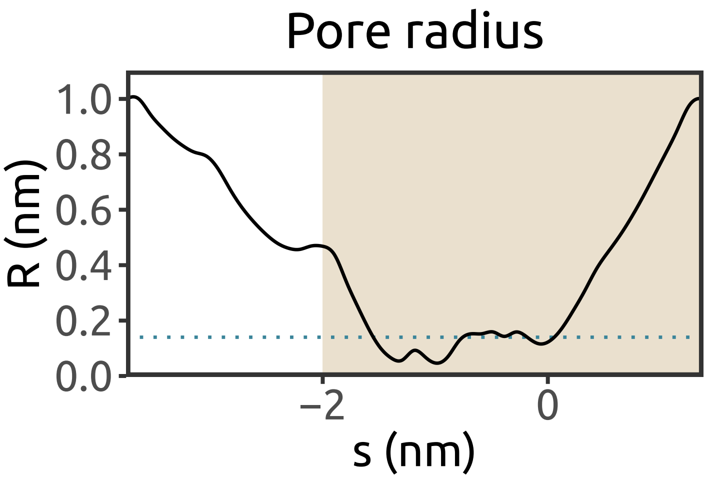


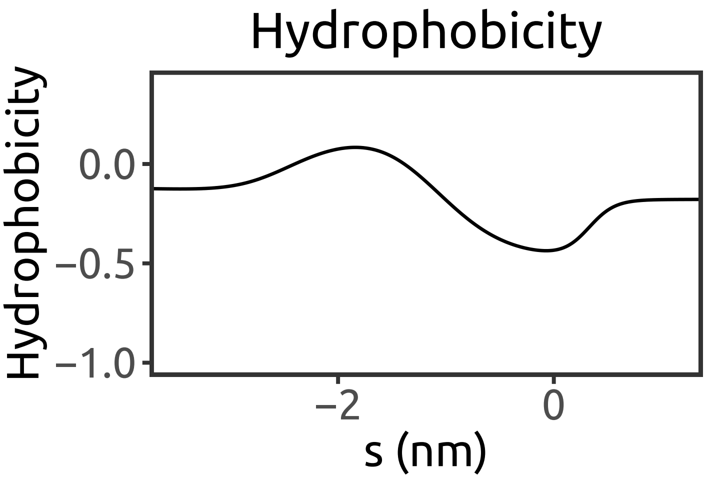


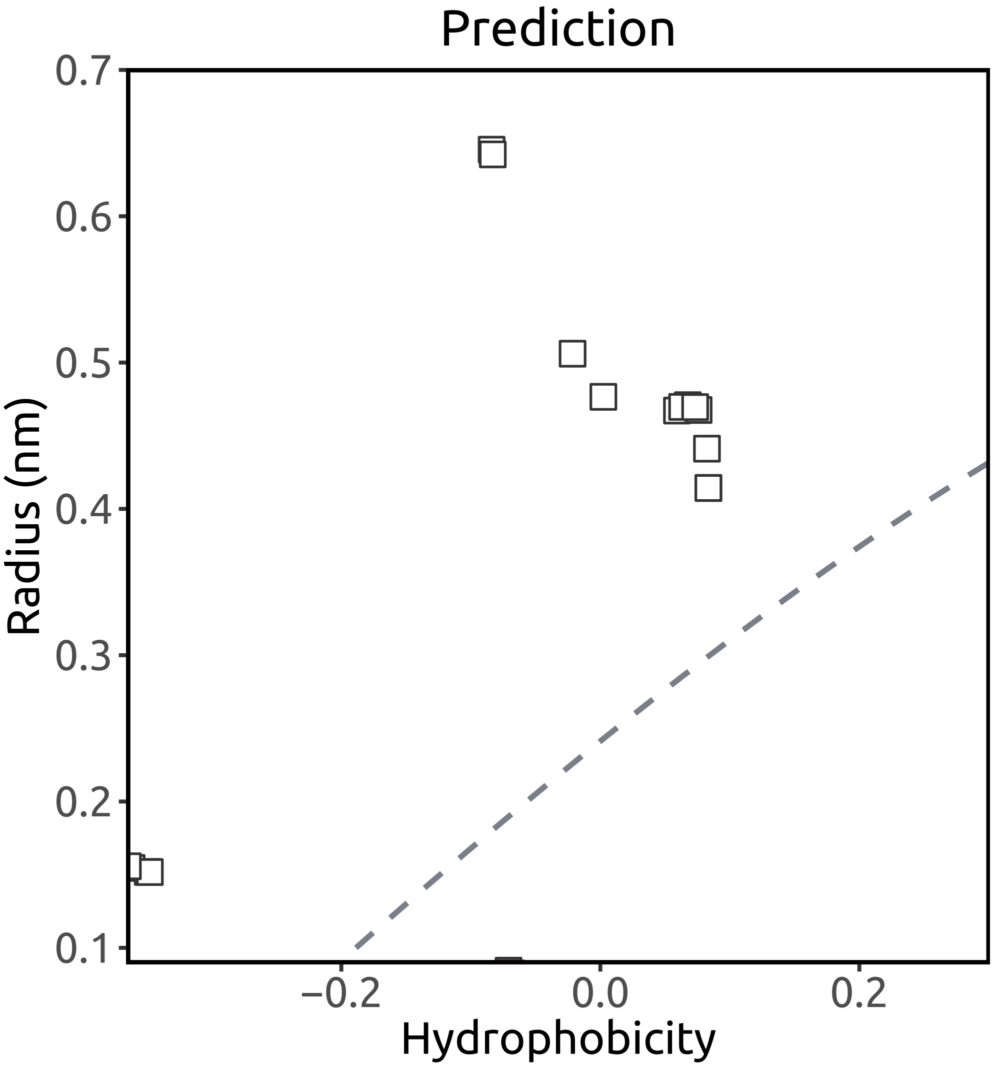


Heuristic score:
0.38 (n = 4)

Simulation result:
barrier to water

# KvLm (PDB ID: 4H33)

*Listeria monocytogenes*
X–ray (3.1 Å)

Santos et al., 2012

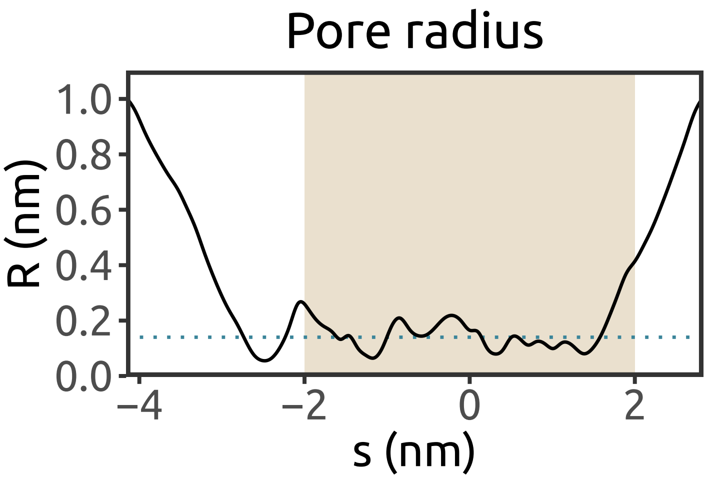


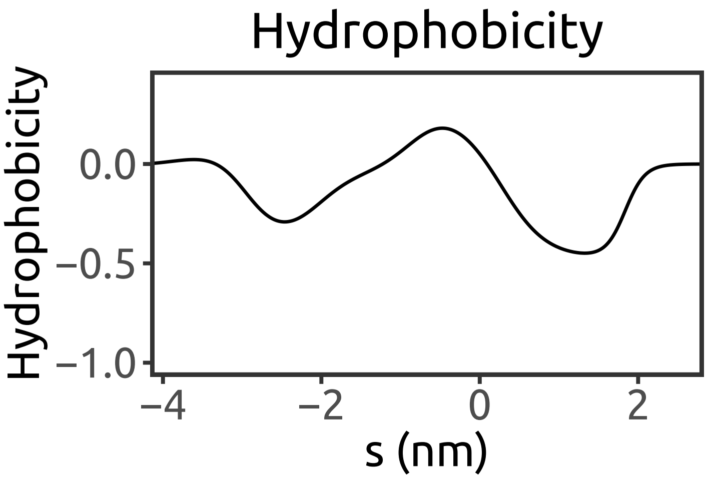


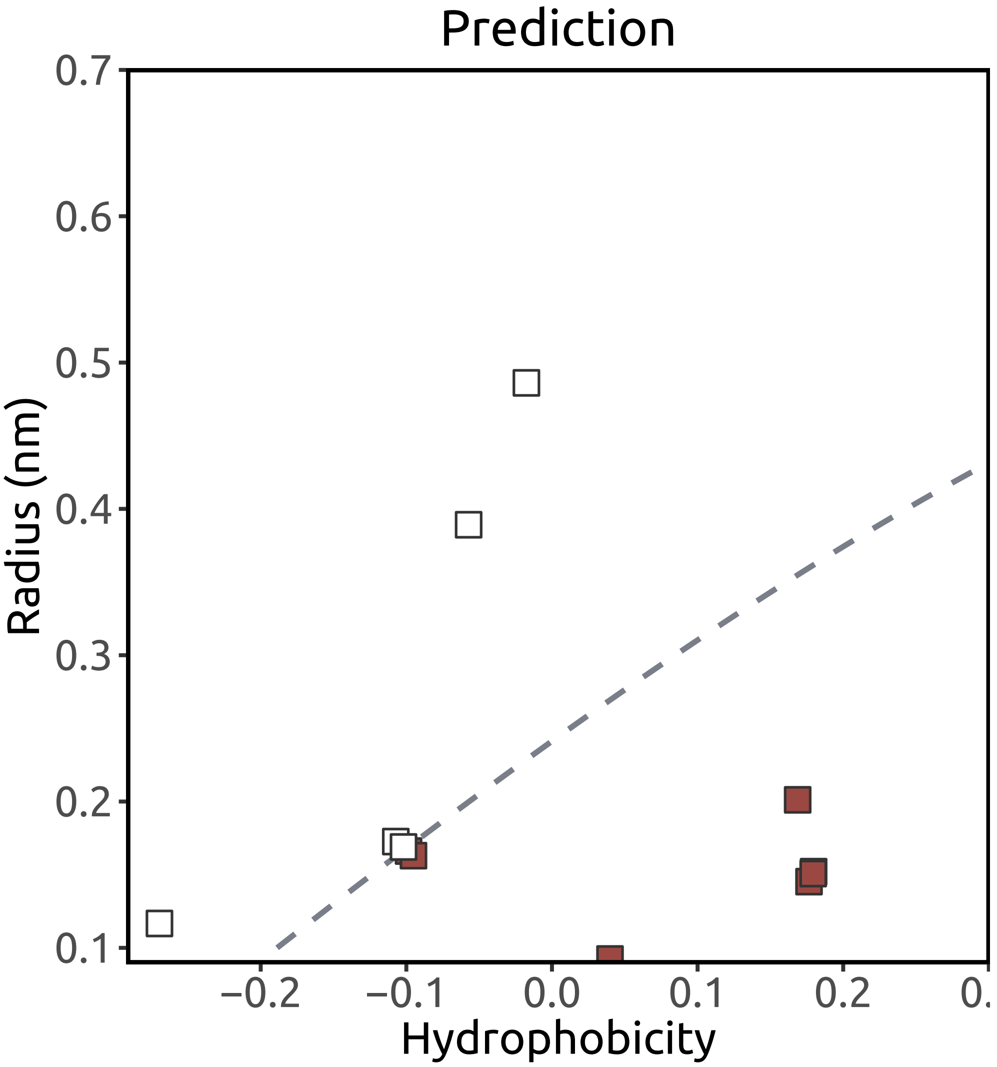


Heuristic score:
1.25 (n = 10)

Simulation result:
barrier to water

# KvLm (PDB ID: 4H37)

*Listeria monocytogenes*

X–ray (3.35 Å)

Santos et al., 2012

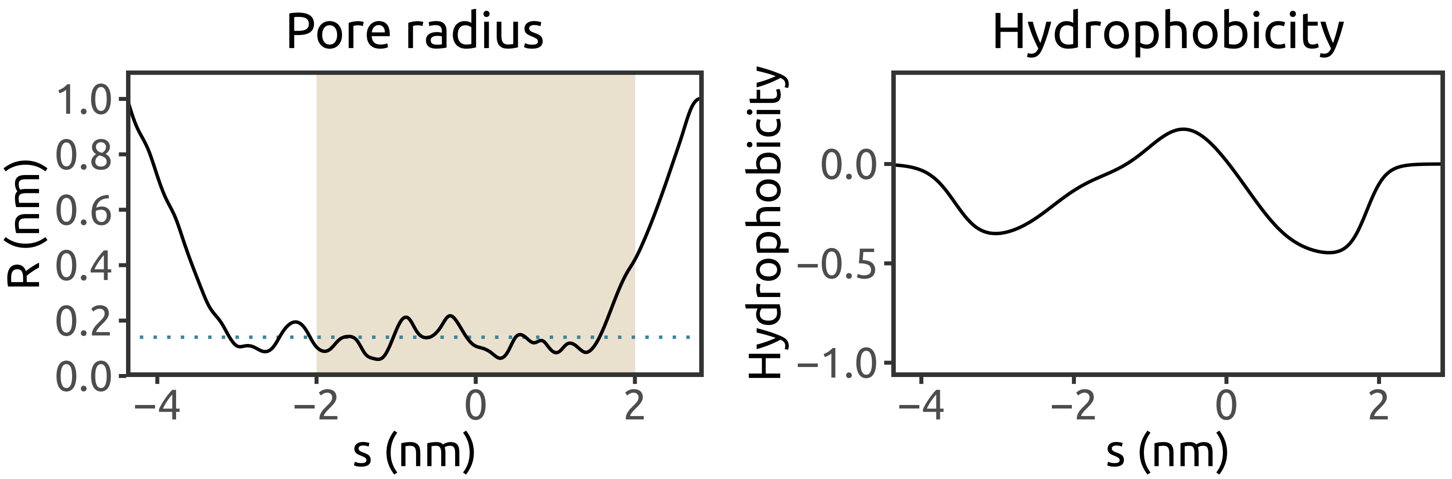


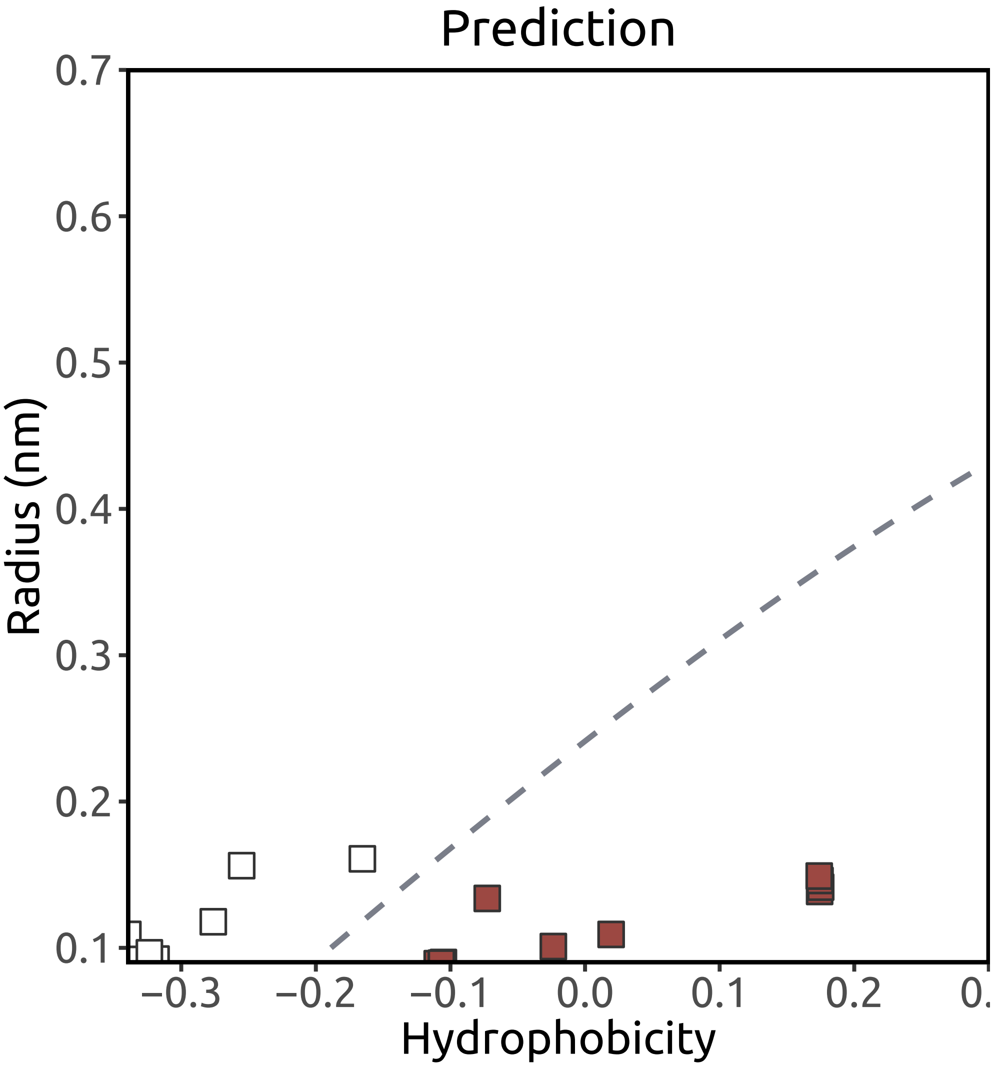


Heuristic score:

1.76 (n = 14)

Simulation result:

barrier to water

# MloK1 (PDB ID: 3BEH)

*Mesorhizobium loti*
X−ray (3.1 Å)

Clayton et al., 2008

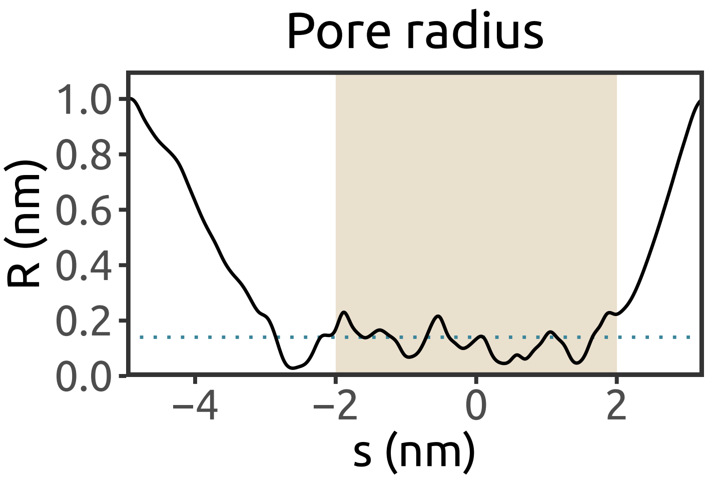


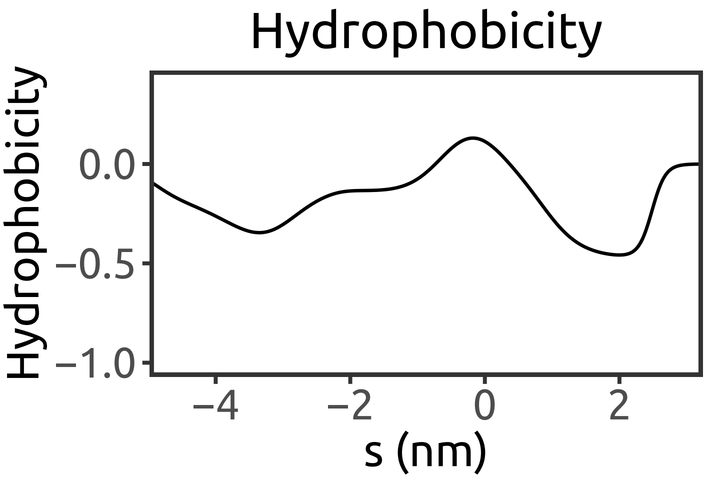


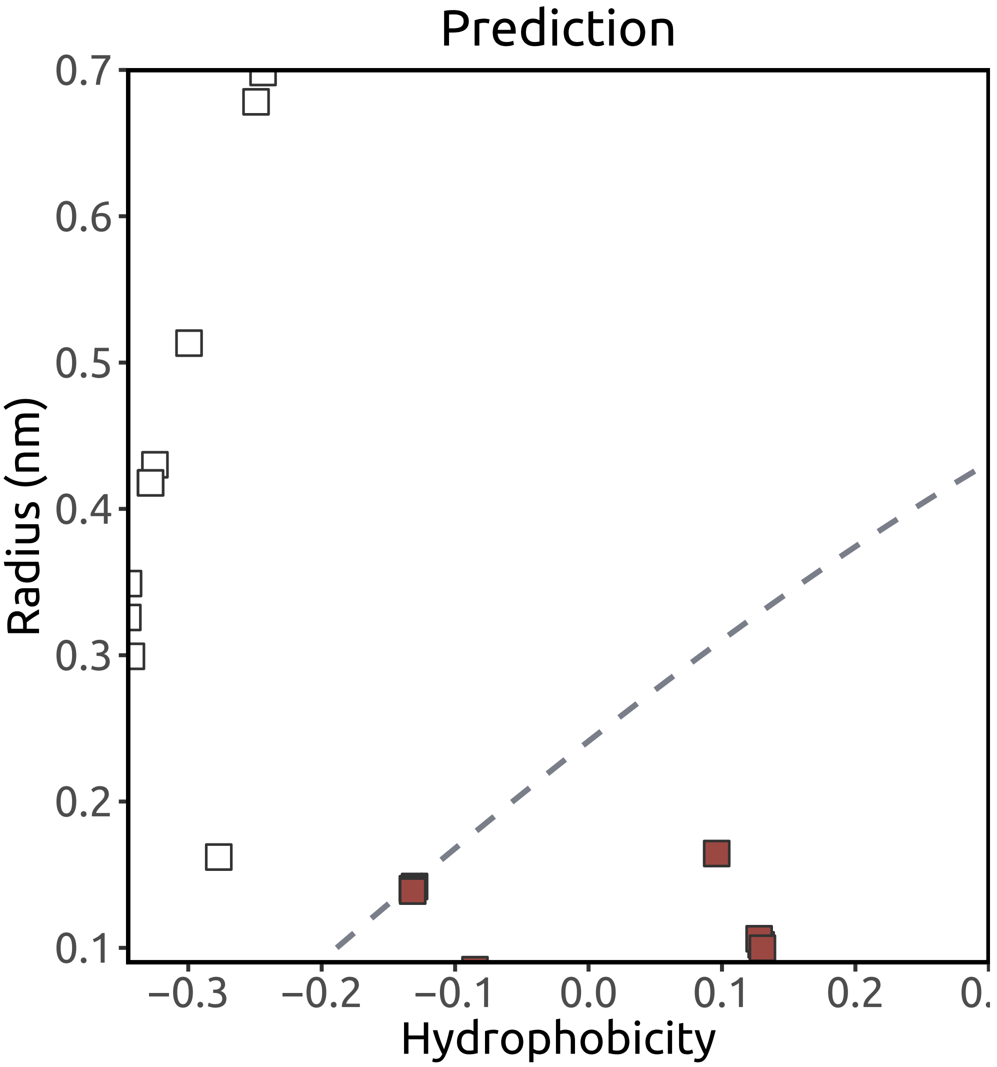


Heuristic score:
1.13 (n = 13)

Simulation result:
barrier to water

# MloK1 (PDB ID: 6EO1)

*Mesorhizobium loti*
cryo−EM (4.5 Å)

Kowal et al., 2018

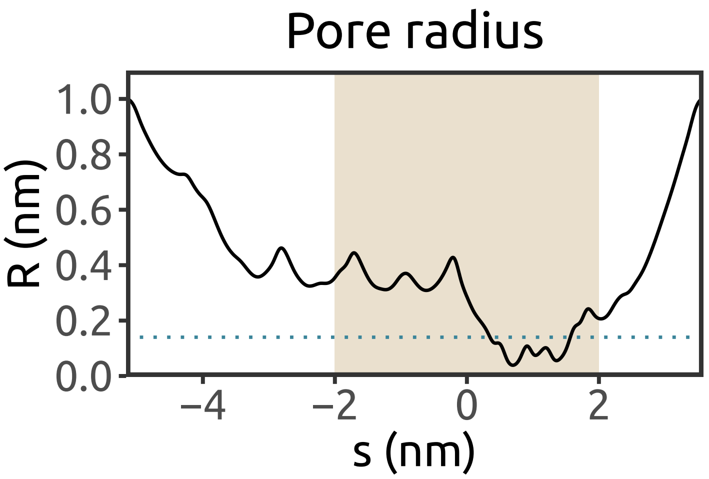


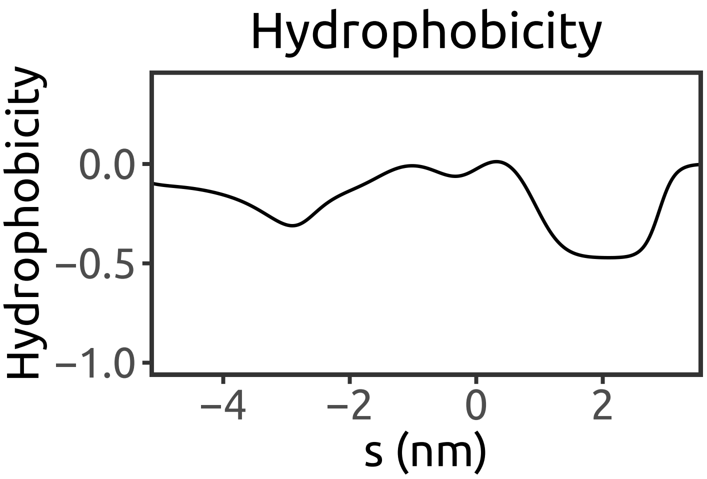


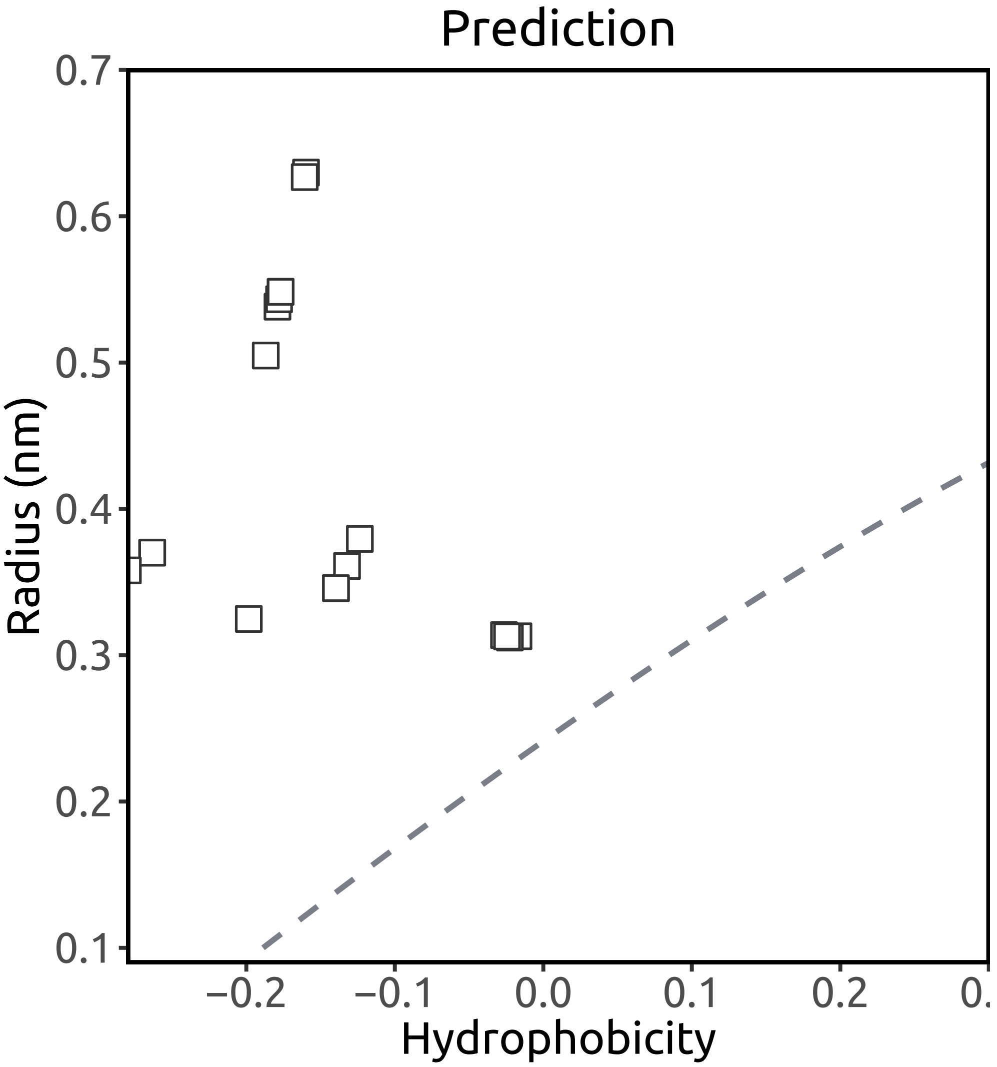


Heuristic score:
0 (n = 0)

Simulation result:
hydrated channel

# Slo1 (PDB ID: 5TJ6)

*Aplysia californica*
cryo−EM (3.5 Å)

Tao el al, 2017

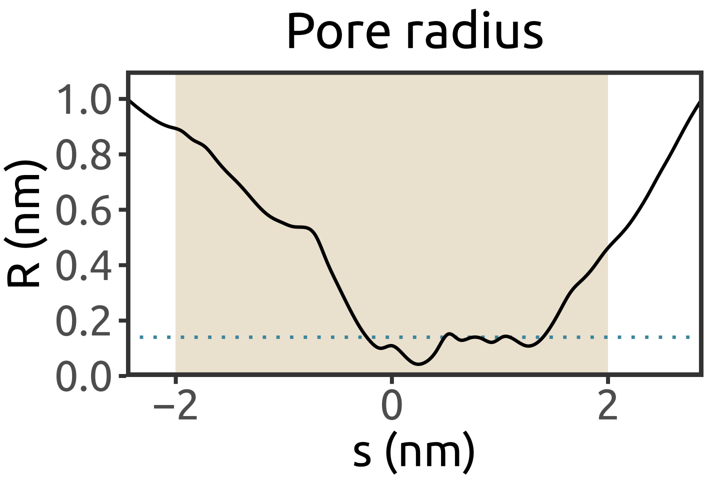


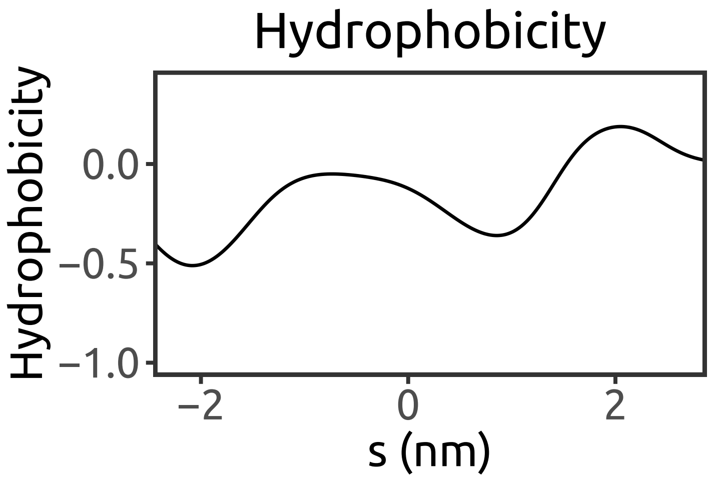


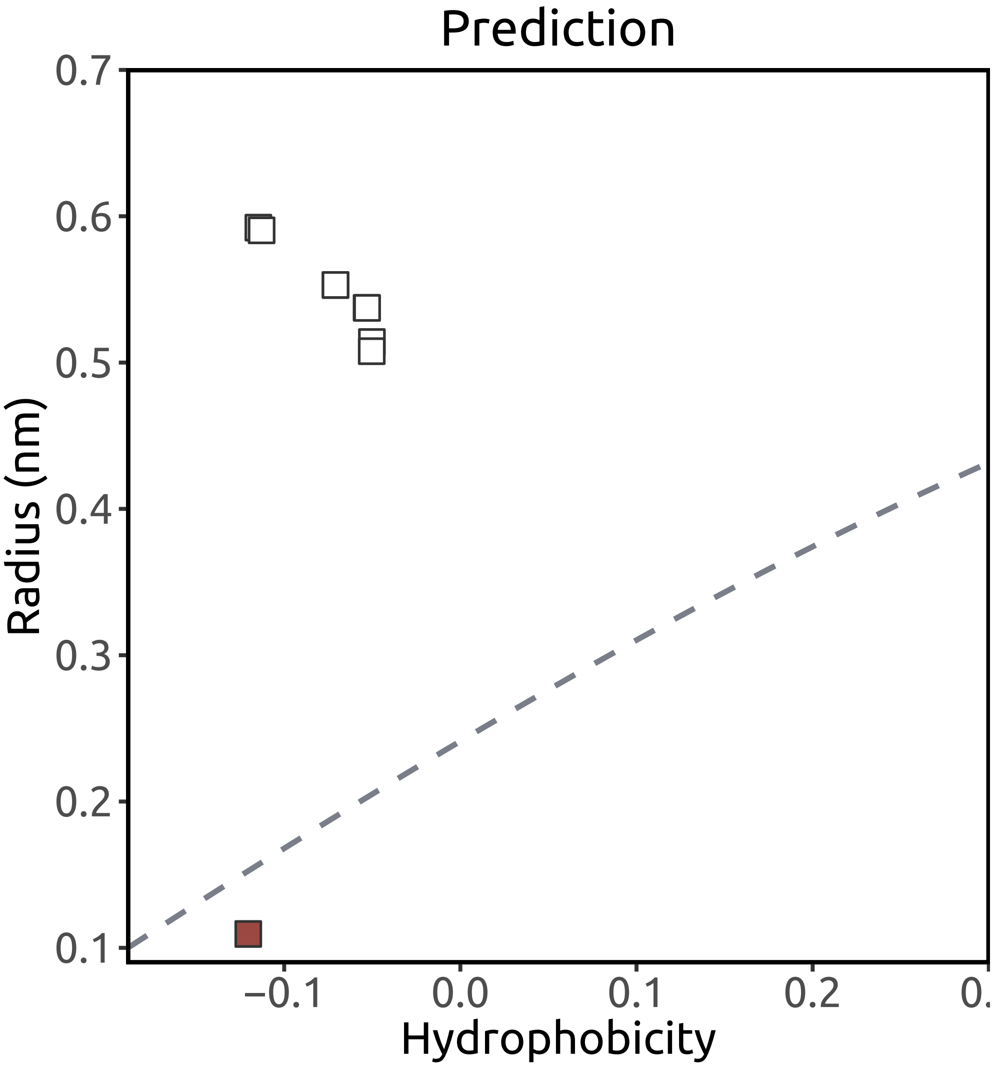


Heuristic score:
0.03 (n = 1)

Simulation result:
hydrated channel

# Slo1 (PDB ID: 5TJI)

*Aplysia californica*
cryo−EM (3.8 Å)

Hite et al., 2017

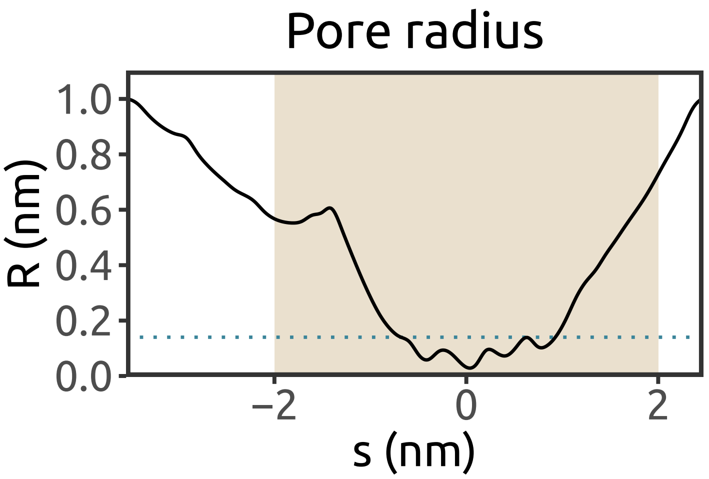


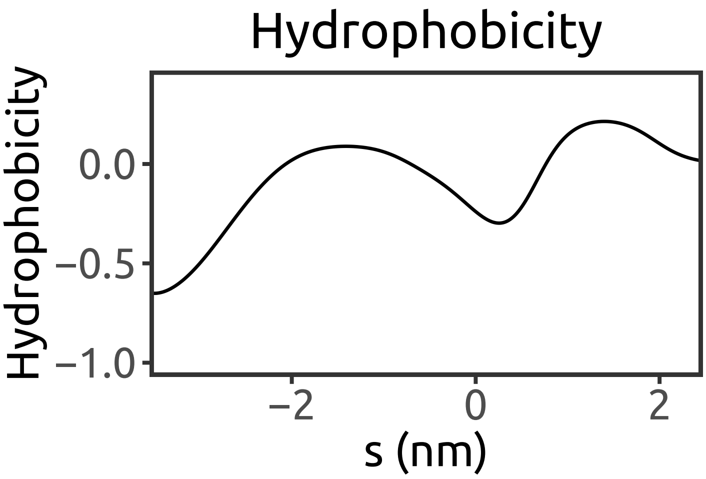


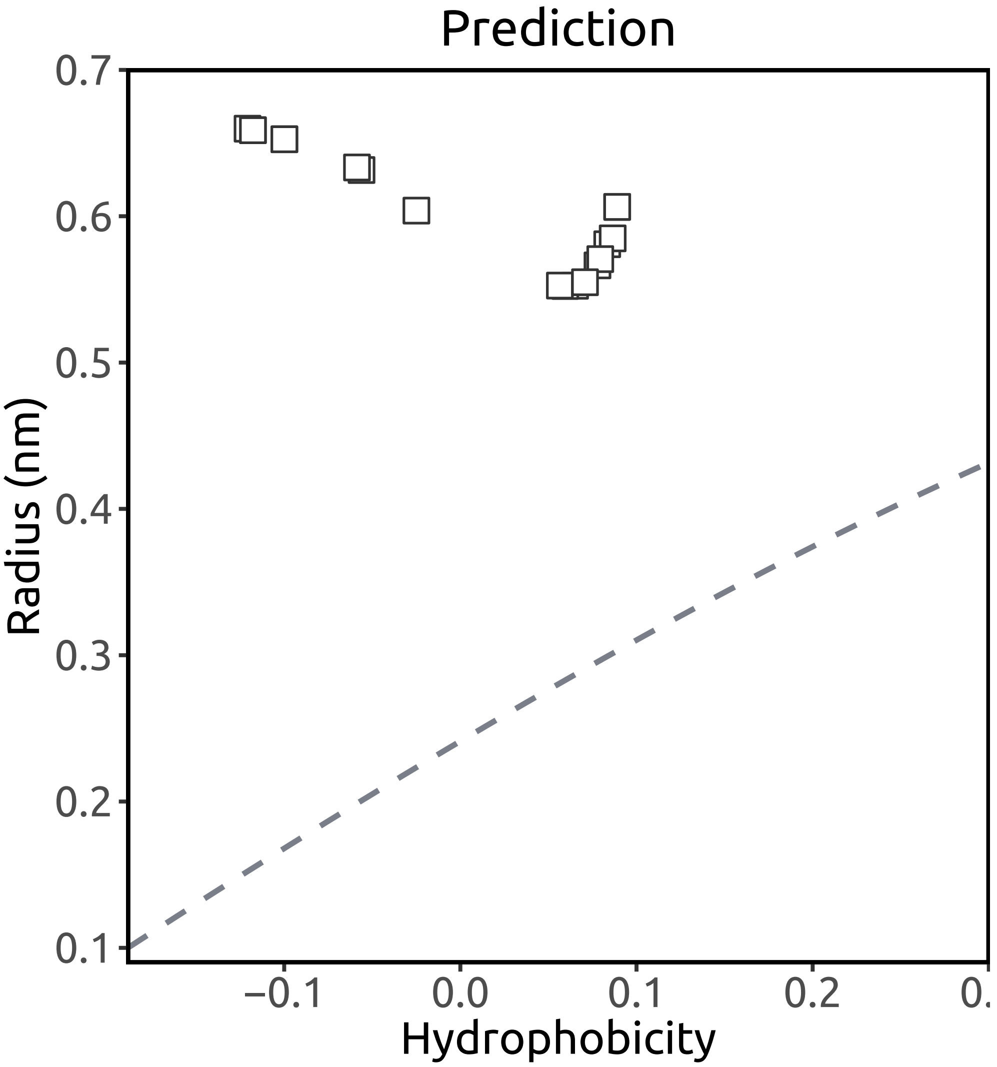


Heuristic score:
0 (n = 0)

Simulation result:
hydrated channel

# Slo2.2 (PDB ID: 5U70)

*Gallus gallus*
cryo–EM (3.76 Å)

Hite & MacKinnon, 2017

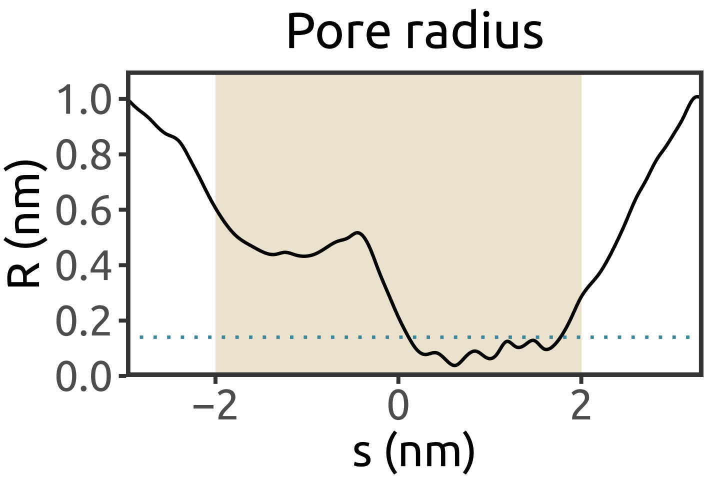


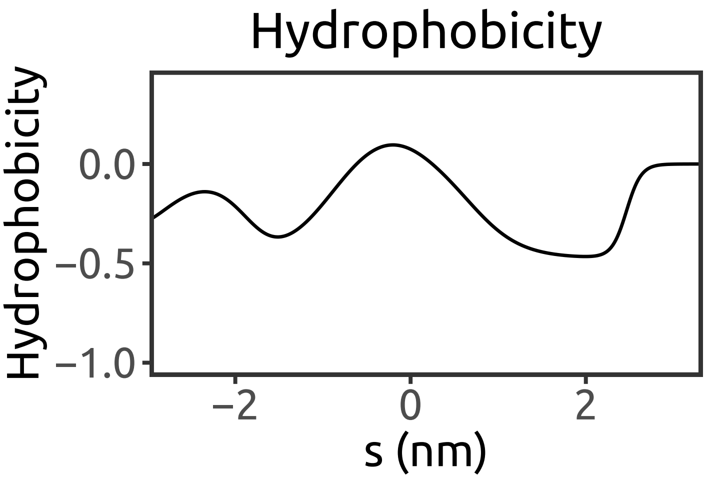


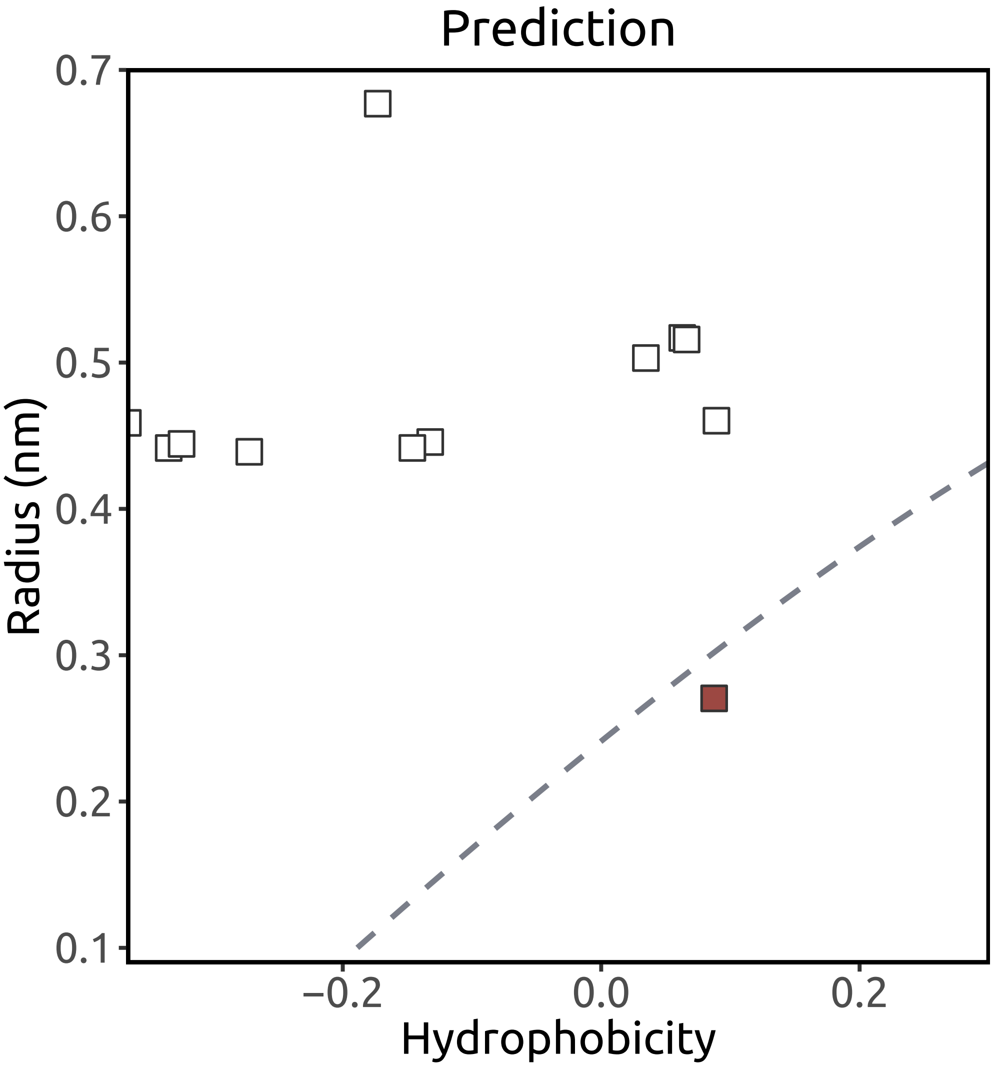


Heuristic score:
0.03 (n = 1)

Simulation result:
hydrated channel

# Slo2.2 (PDB ID: 5U76)

*Gallus gallus*
cryo–EM (3.76 Å)

Hite & MacKinnon, 2017

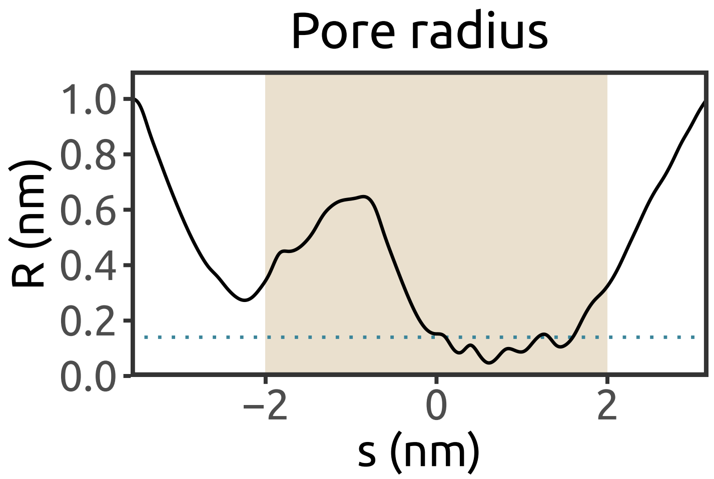


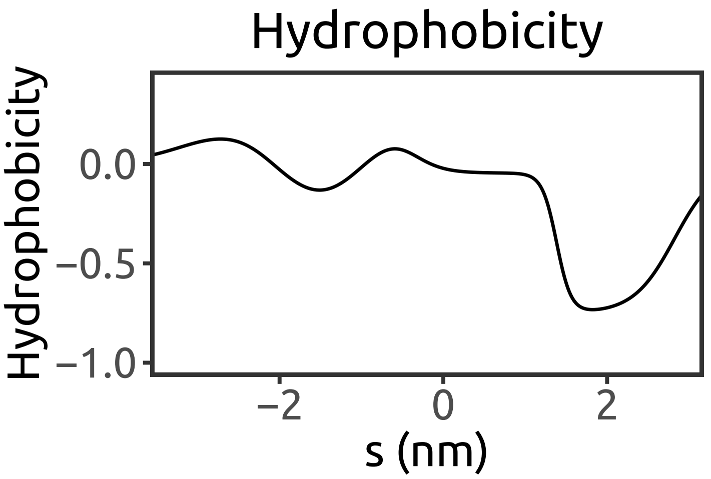


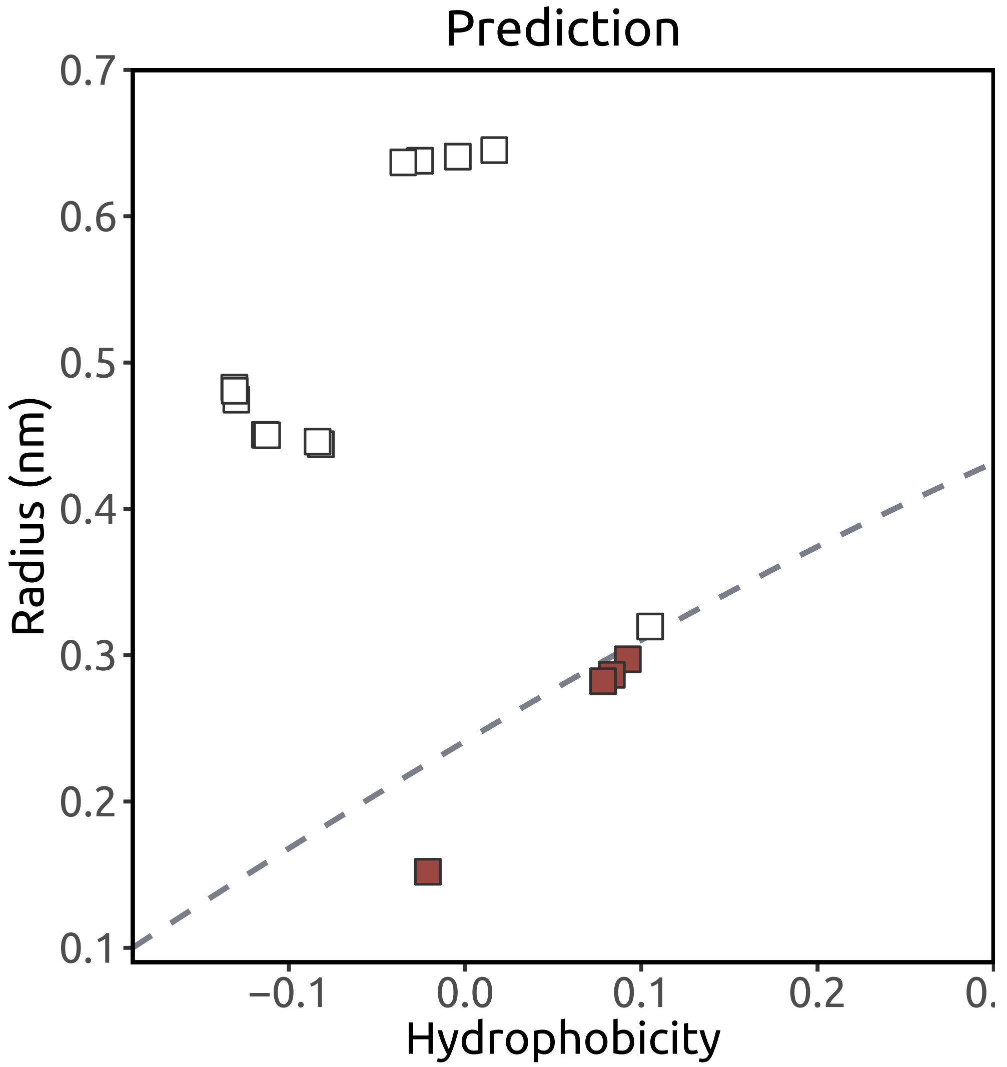


Heuristic score:
0.09 (n = 4)

Simulation result:
hydrated channel

# Cav1.1 (PDB ID: 5GJV)

*Oryctolagus cuniculus*
cryo–EM (3.6 Å)

Wu et al., 2016

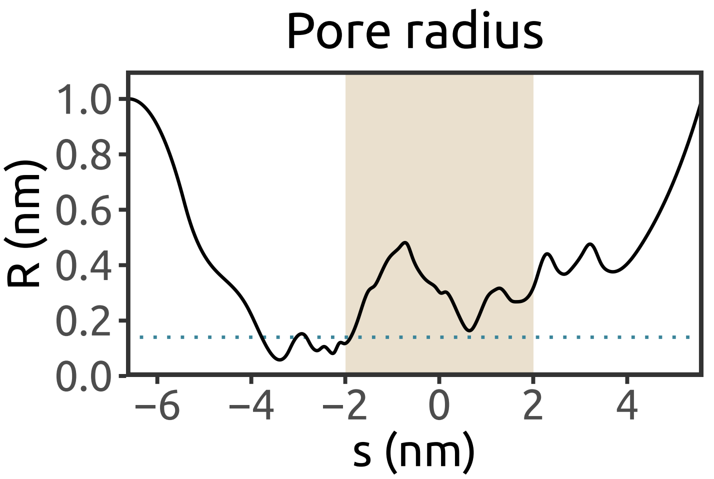


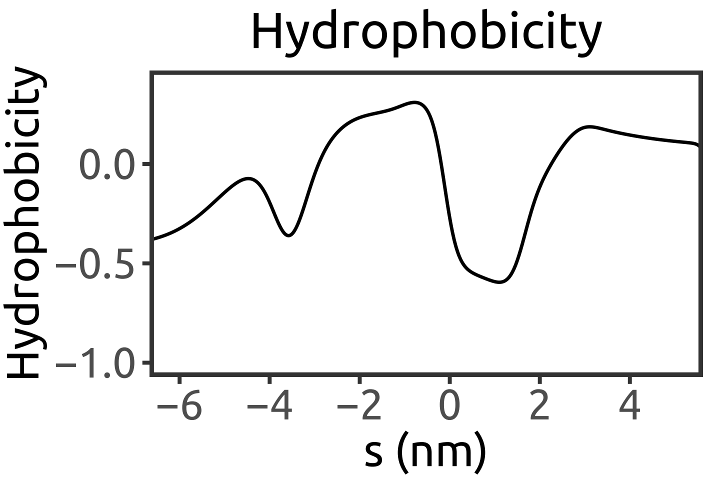


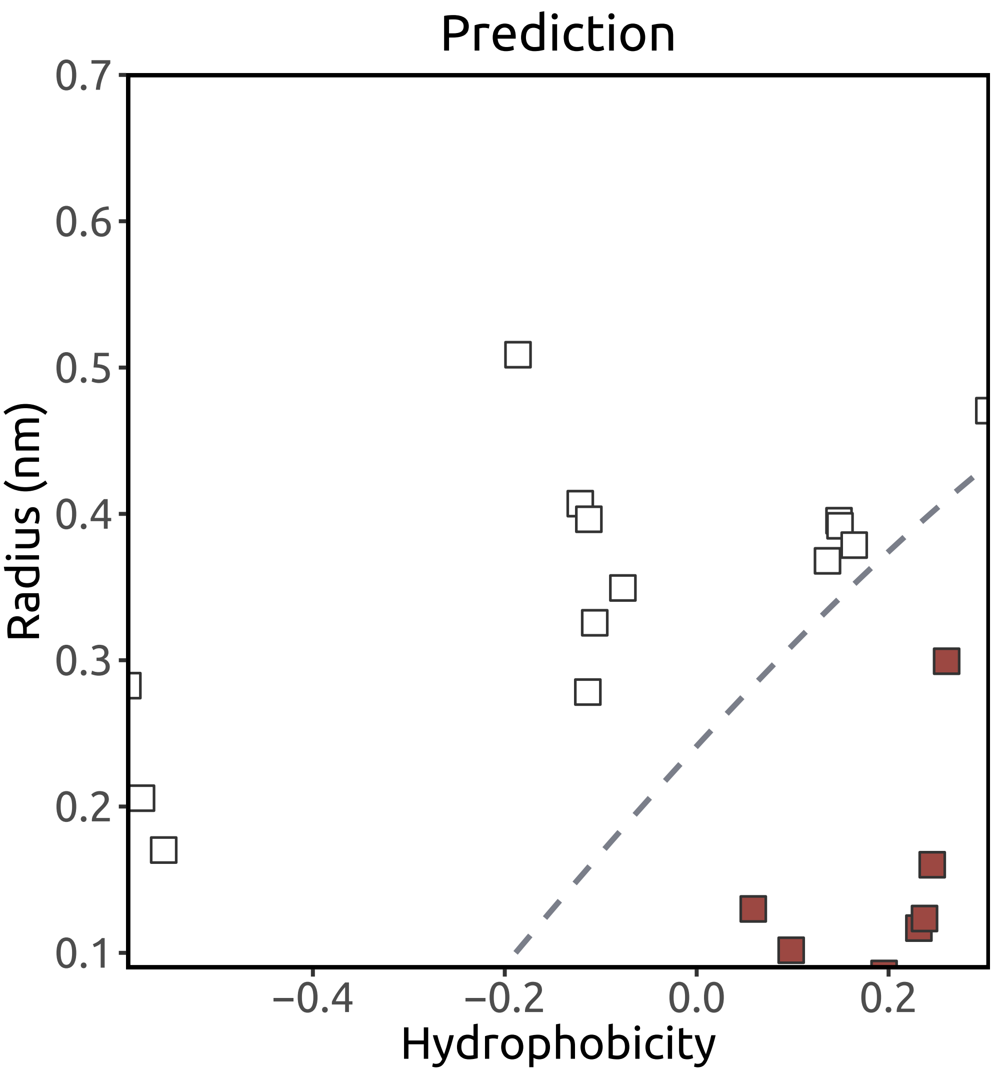


Heuristic score:
1.3 (n = 7)

Simulation result:
barrier to water

# CavAb (PDB ID: 4MVM)

*Arcobacter butzleri*
X–ray (3.20 Å)

Tang et al., 2014

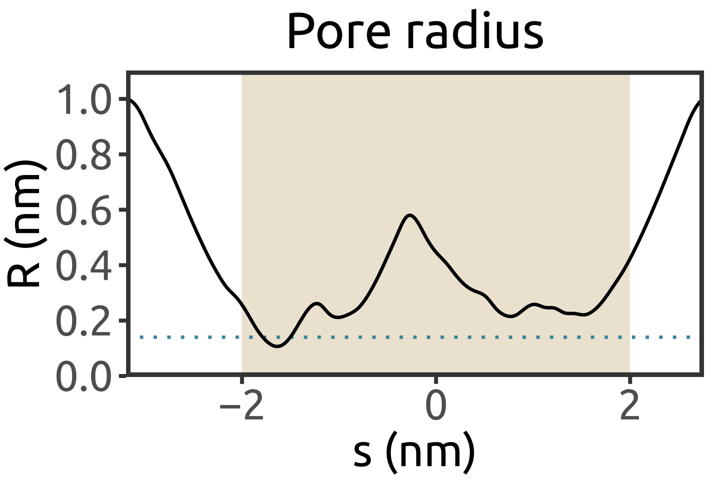


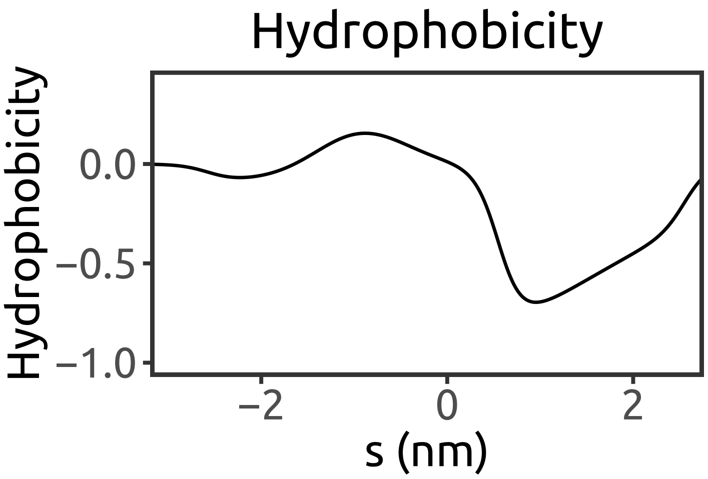


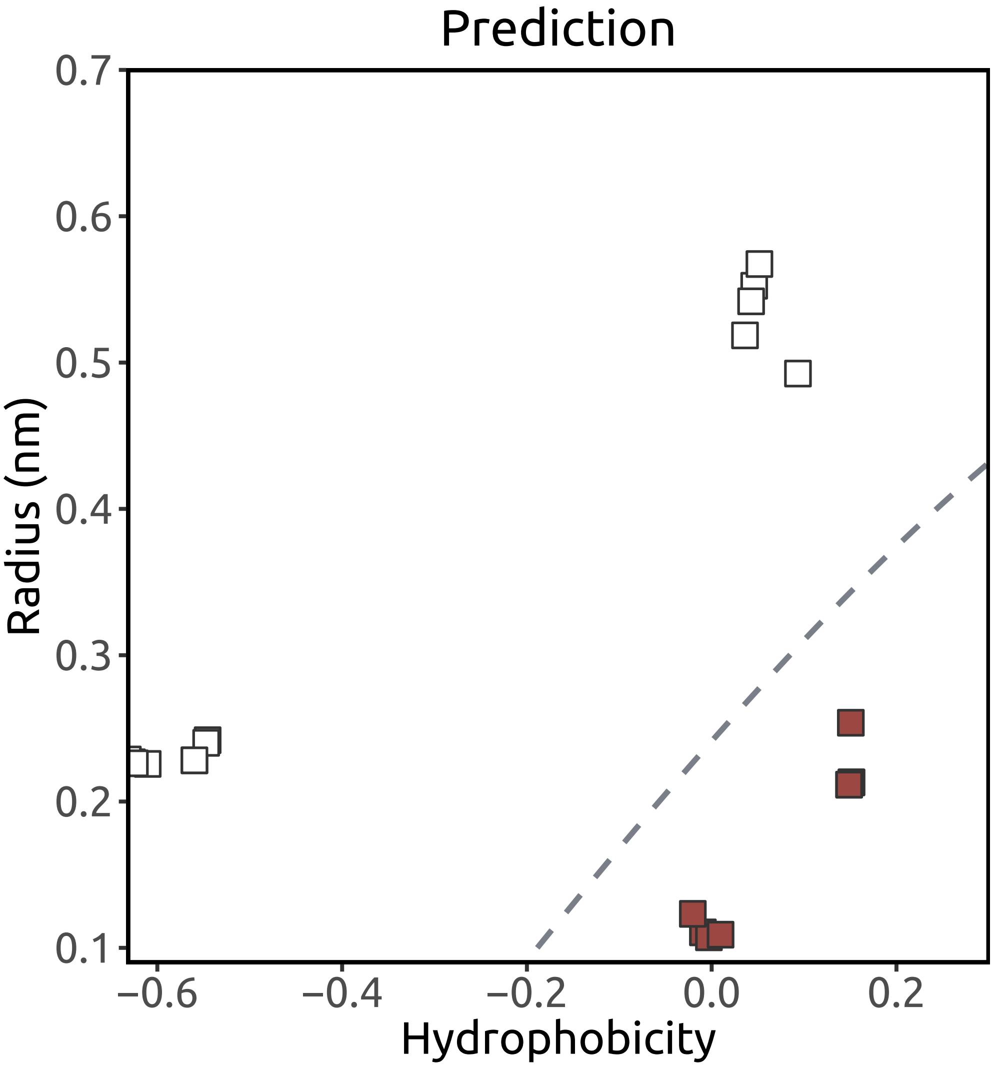


Heuristic score:
0.92 (n = 9)

Simulation result:
barrier to water

# CavAb (PDB ID: 5KLB)

*Arcobacter butzleri*
X–ray (2.7 Å)

Tang et al., 2016

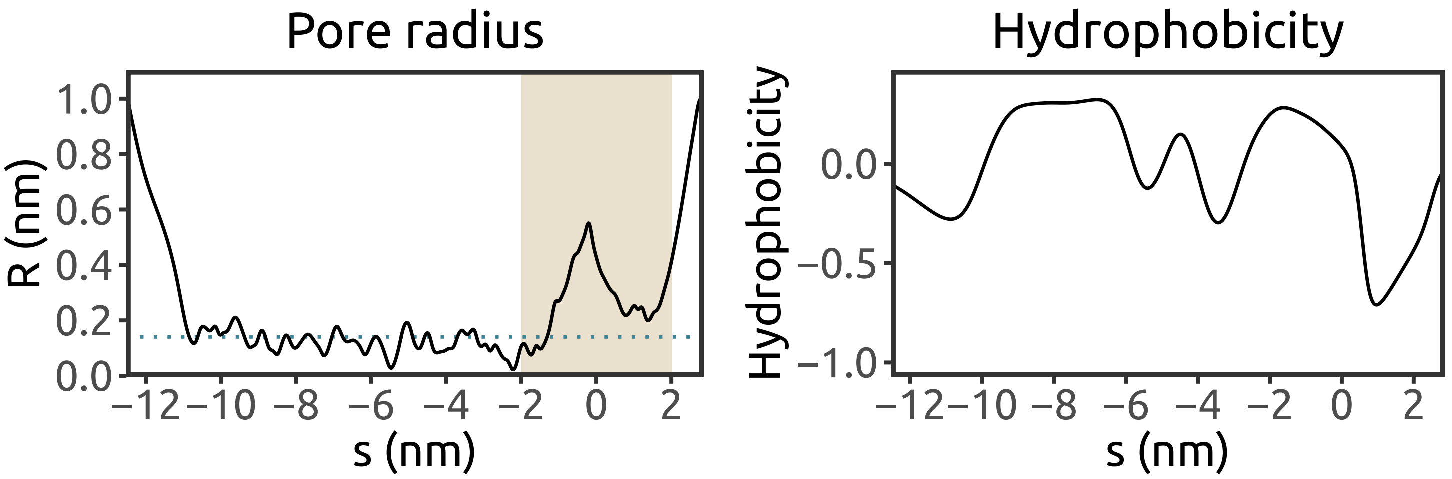


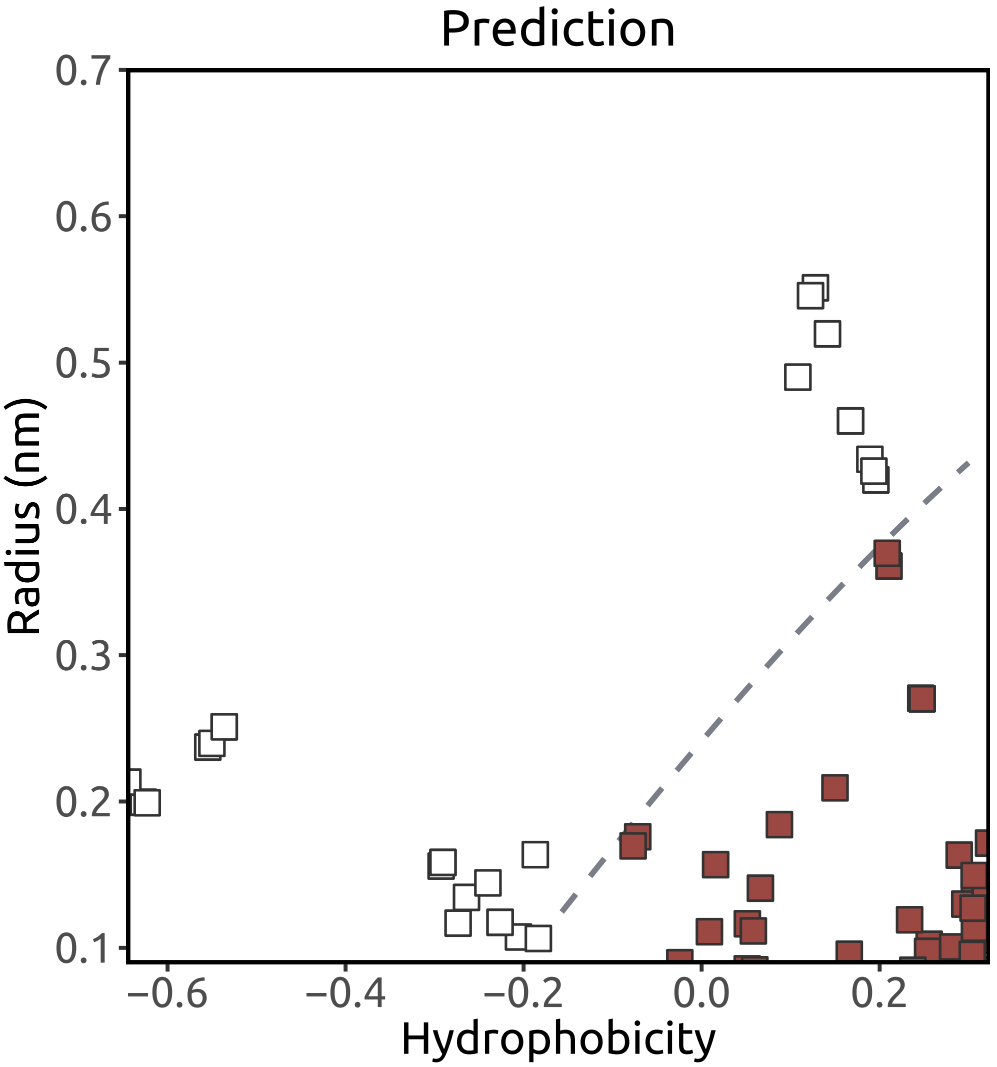


Heuristic score:
9.44 (n = 50)

Simulation result:
barrier to water

# CNG (PDB ID: 5H3O)

*Caenorhabditis elegans*

cryo−EM (3.5 Å)

Li et al., 2017

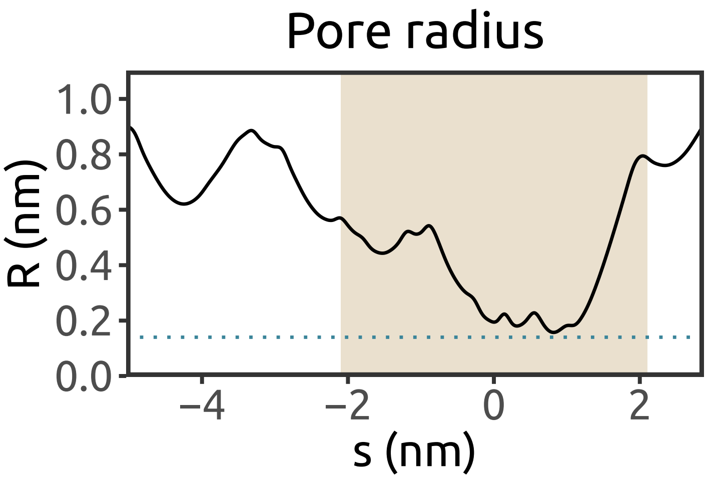


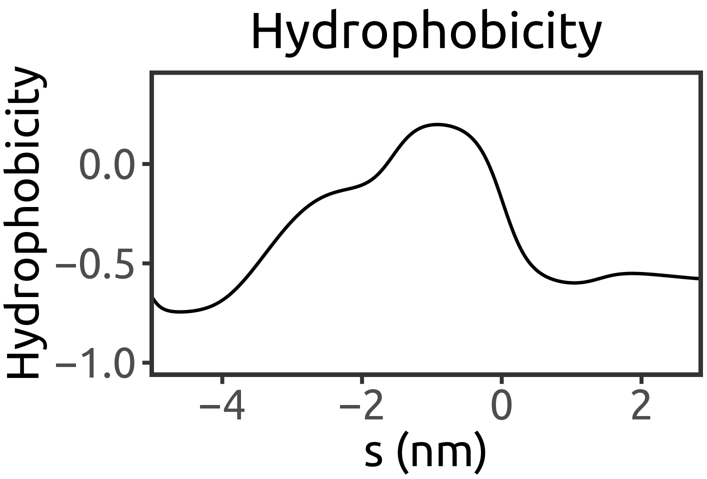


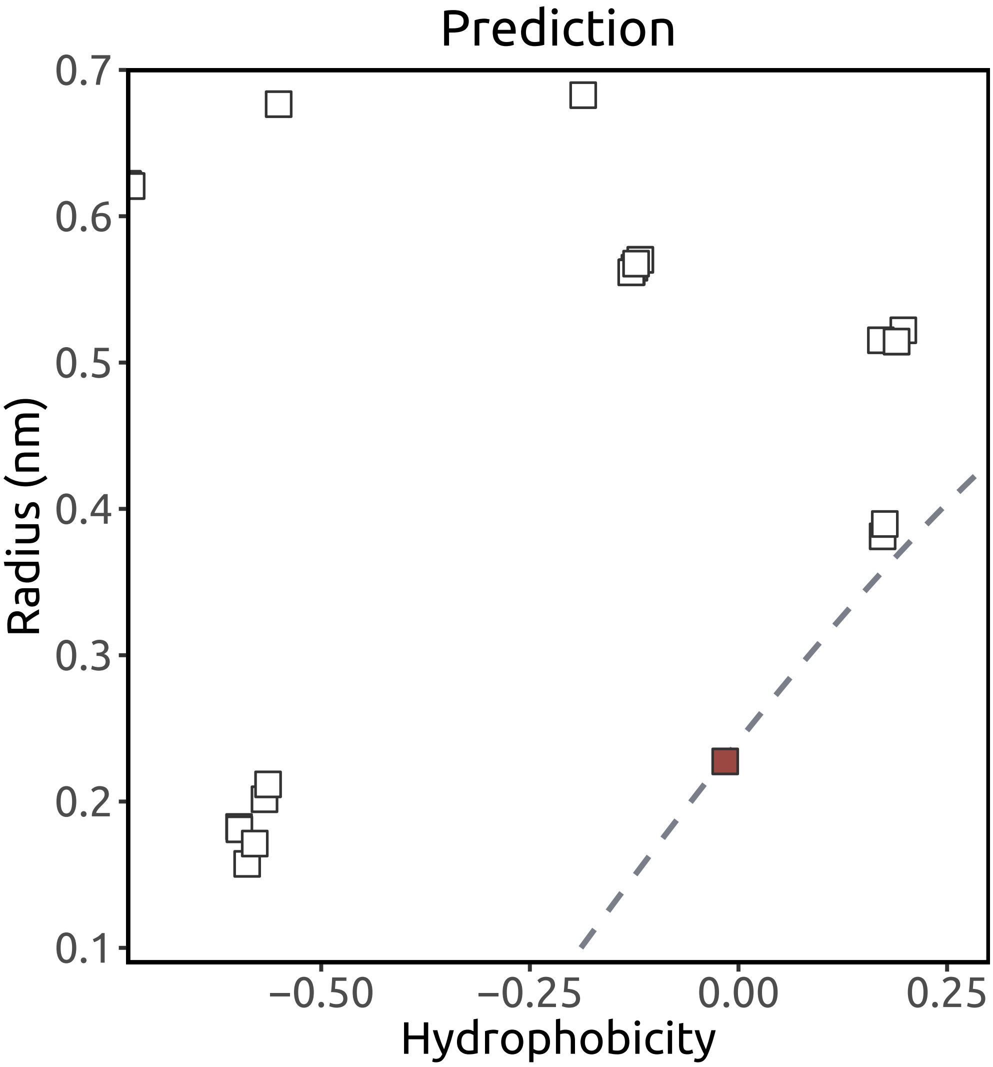


Heuristic score:

0 (n = 1)

Simulation result:

hydrated channel

# CNG (PDB ID: 5V4S)

*Leptospira licerasiae*
cryo−EM (4.2 Å)

James et al., 2017

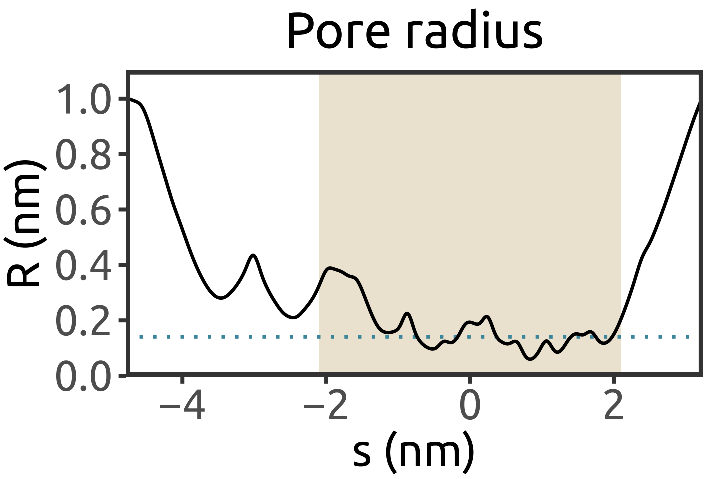


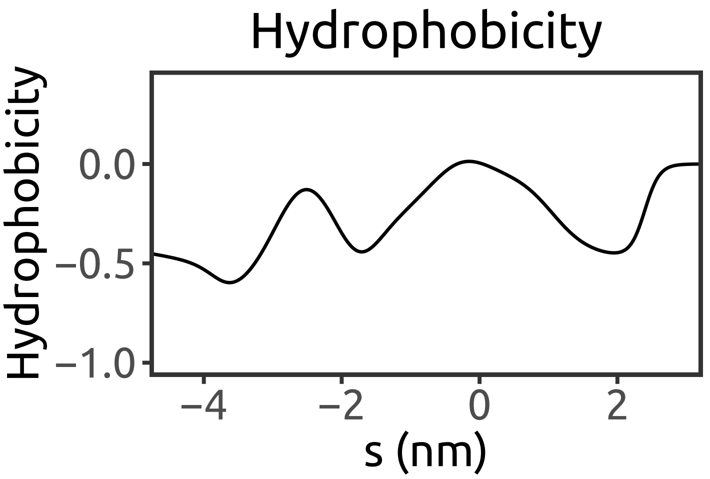


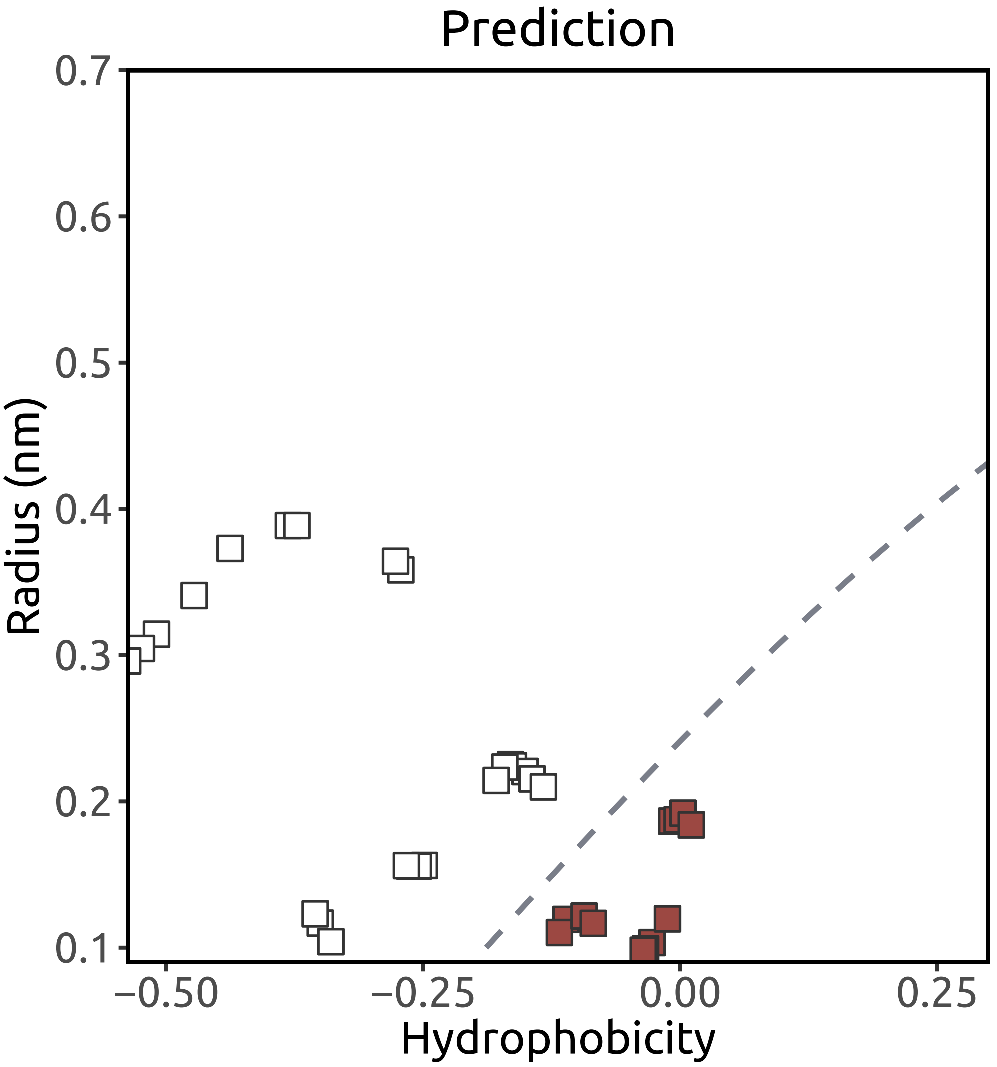


Heuristic score:
0.71 (n = 12)

Simulation result:
barrier to water

# HCN1 (PDB ID: 5U6O)

*Homo sapiens*
cryo−EM (3.5 Å)

Lee & MacKinnon, 2017

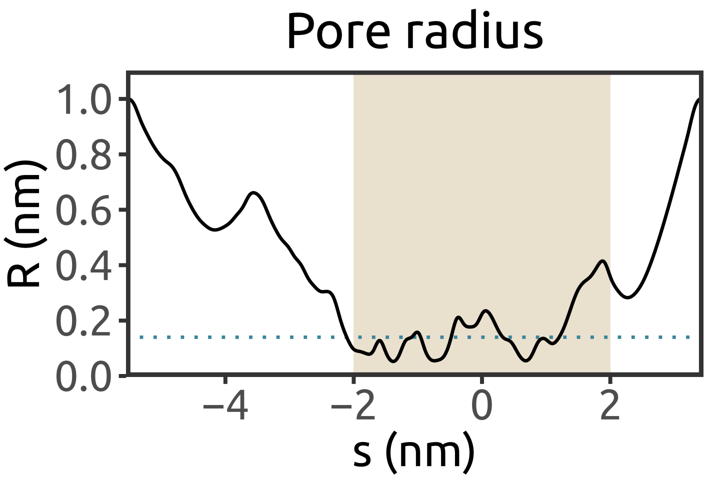


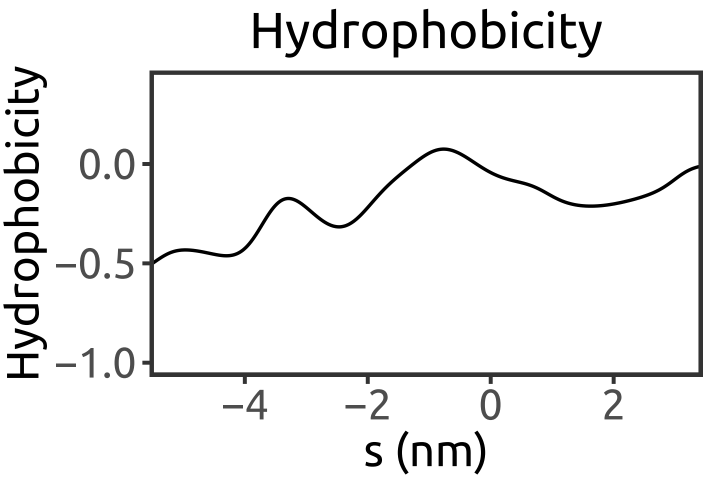


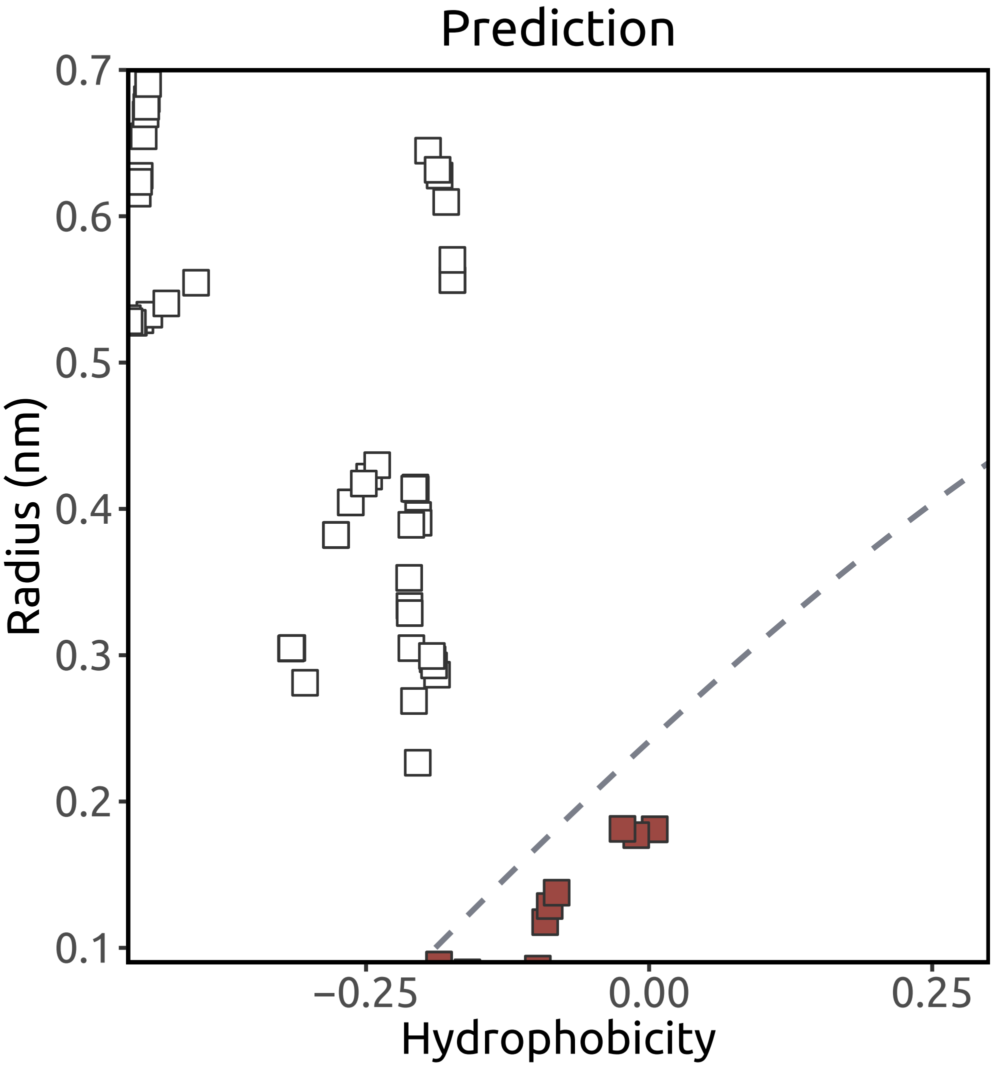


Heuristic score:
1.78 (n = 20)

Simulation result:
barrier to water

# Hv1 (PDB ID: 3WKV)

*Mus musculus*
X−ray (3.45 Å)

Takeshita et al., 2014

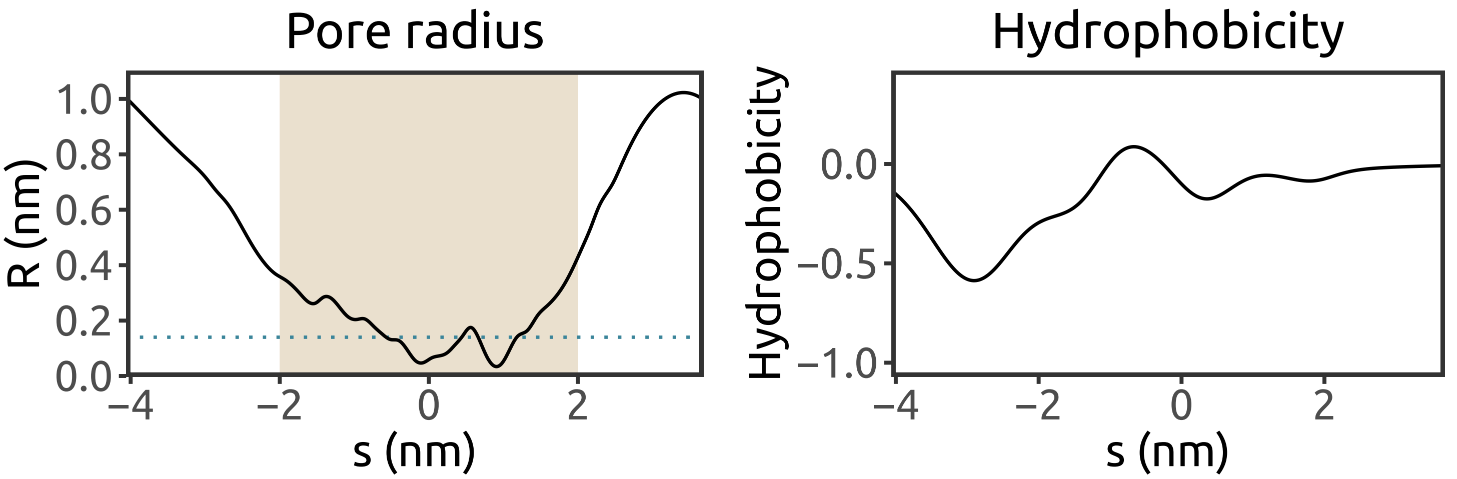


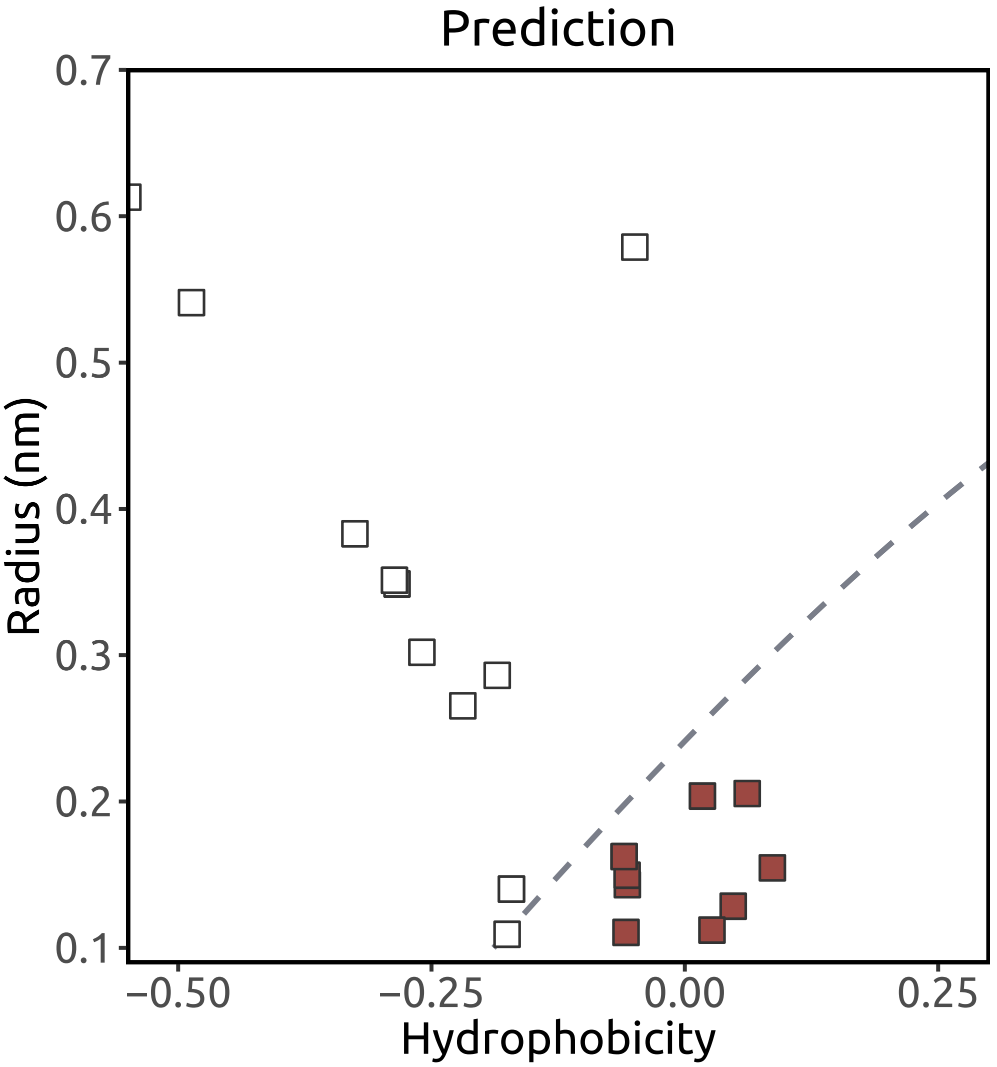


Heuristic score:
1.35 (n = 17)

Simulation result:
barrier to water

# InsP3R1 (PDB ID: 3JAV)

*Rattus norvegicus*
cryo−EM (4.7 Å)

Fan et al., 2015

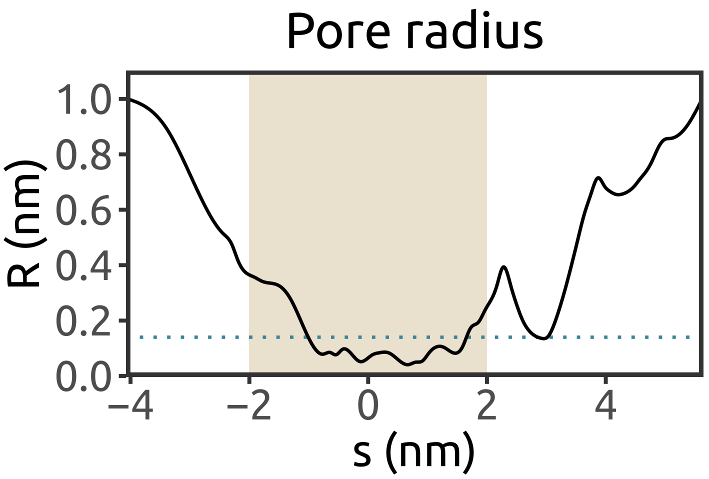


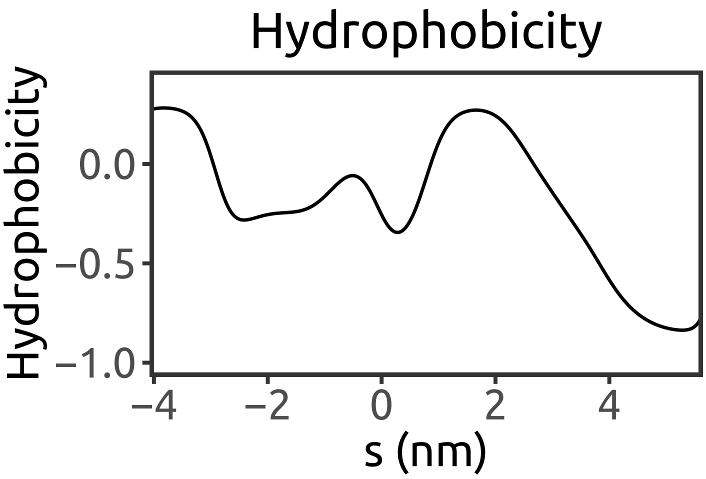


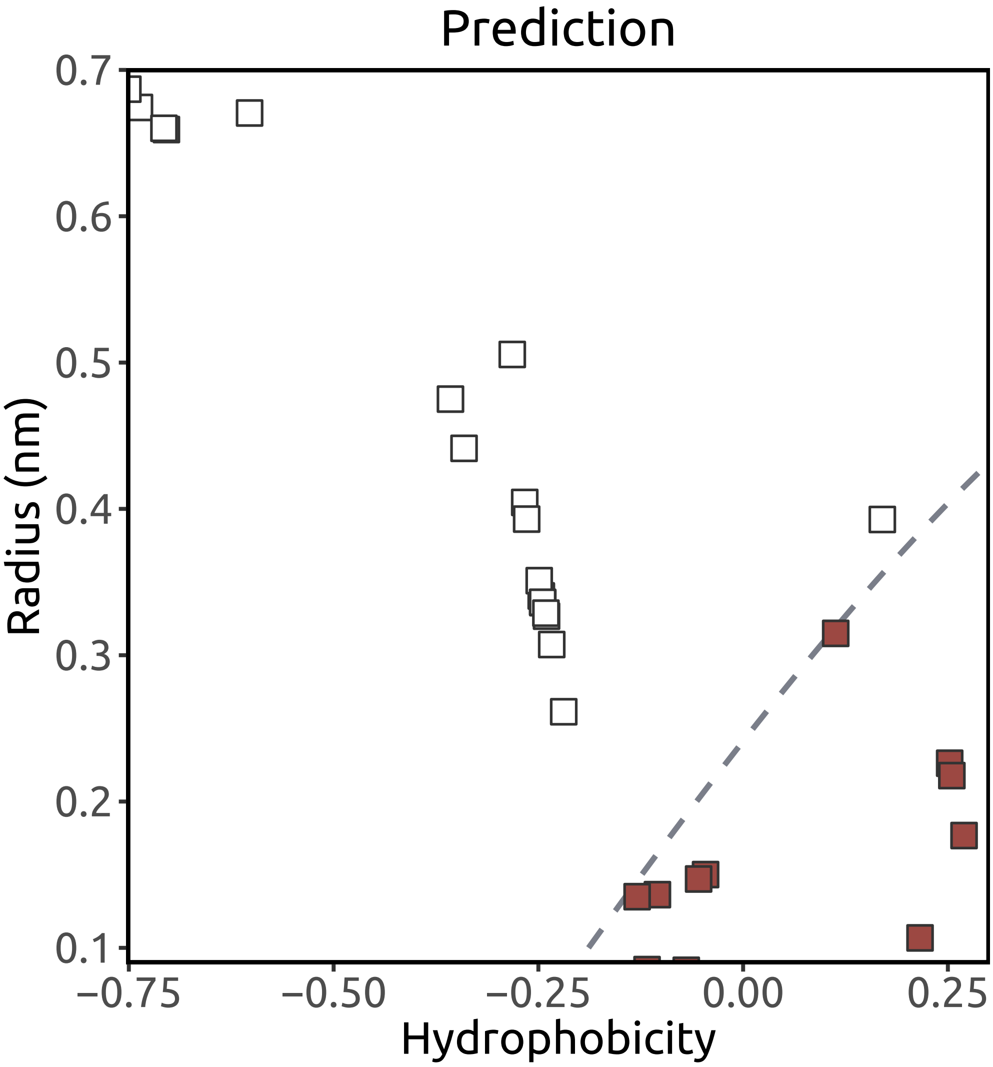


Heuristic score:
1.02 (n = 11)

Simulation result:
barrier to water

# Nav1.4 (PDB ID: 5XSY)

*Electrophorus electricus*
cryo−EM (4 Å)

Yan et al., 2017

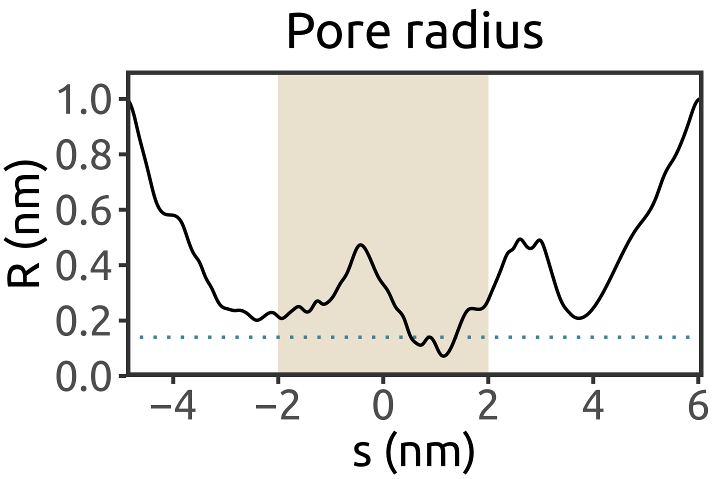


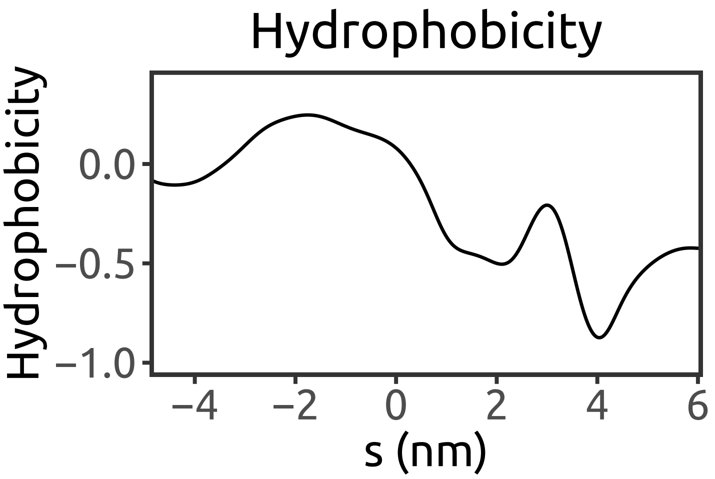


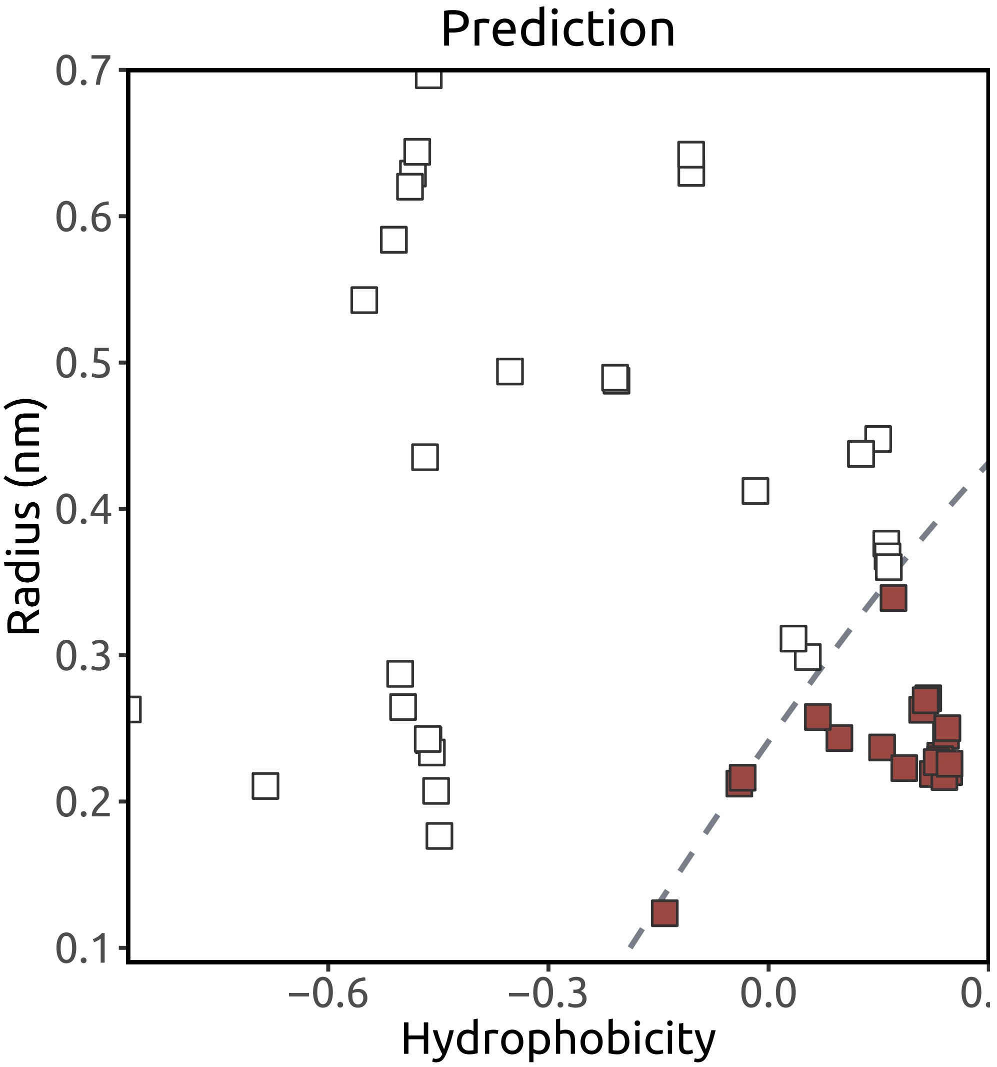


Heuristic score:
2.04 (n = 21)

Simulation result:
barrier to water

# NavAb (PDB ID: 4EKW)

*Arcobacter butzleri*
X–ray (3.21 Å)

Payandeh et al., 2012

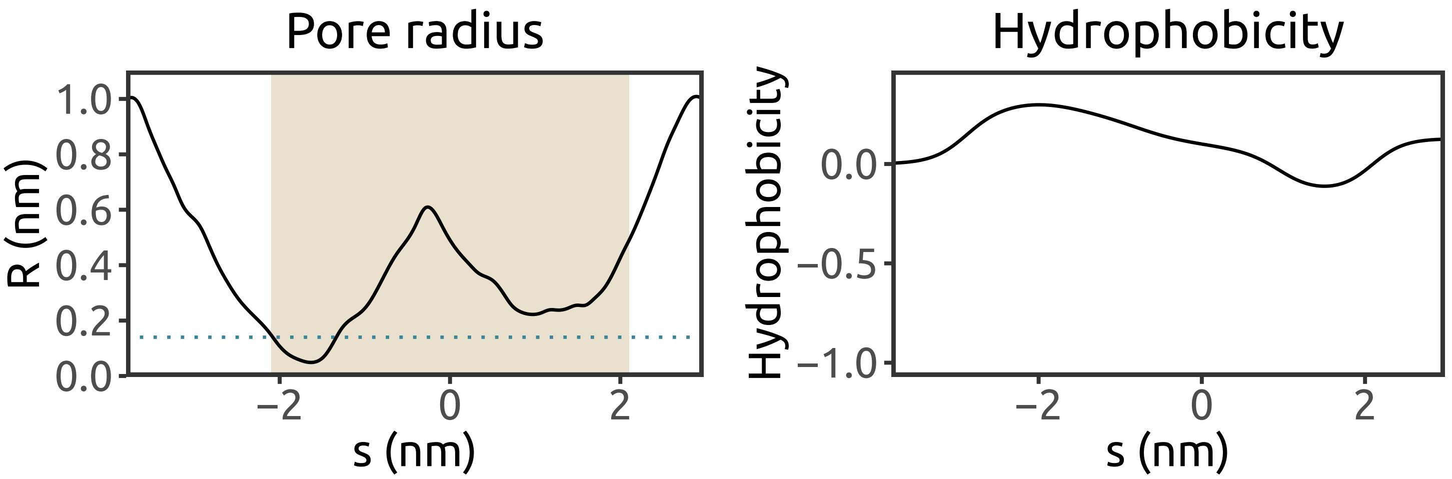


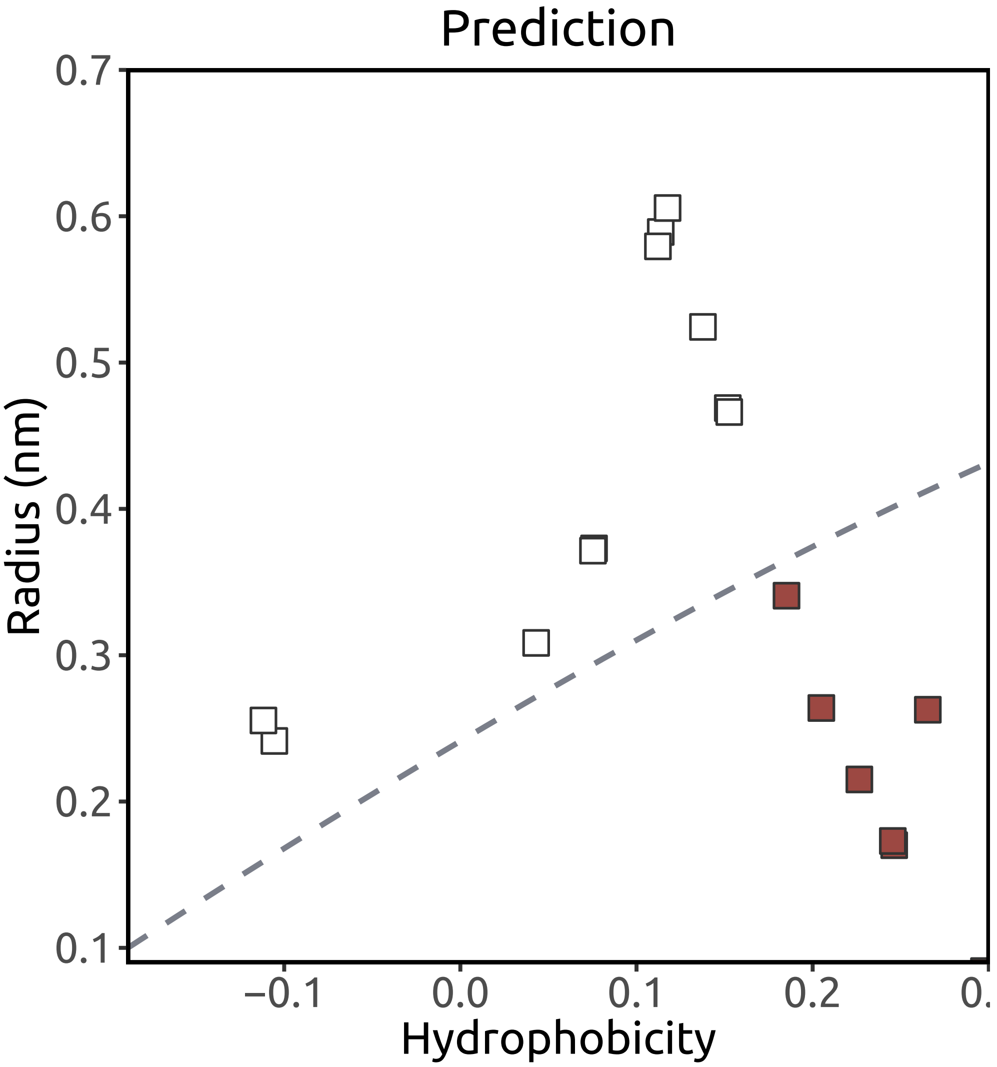


Heuristic score:
1.72 (n = 9)

Simulation result:
barrier to water

# NavAb (PDB ID: 4MW8)

*Arcobacter butzleri*
X–ray (3.26 Å)

Tang et al., 2014

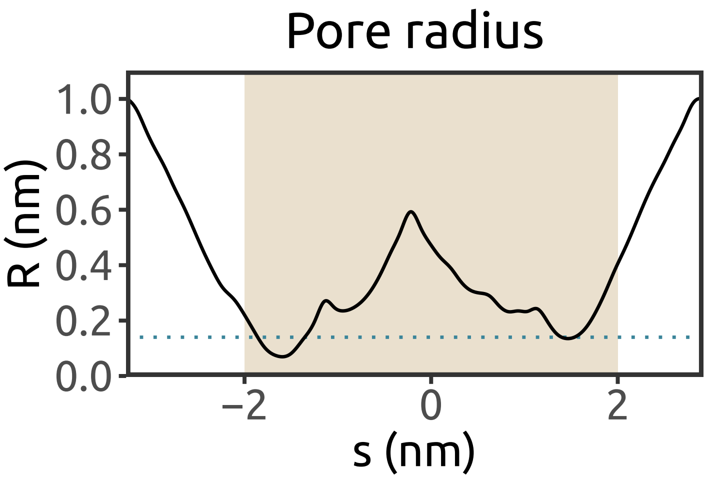


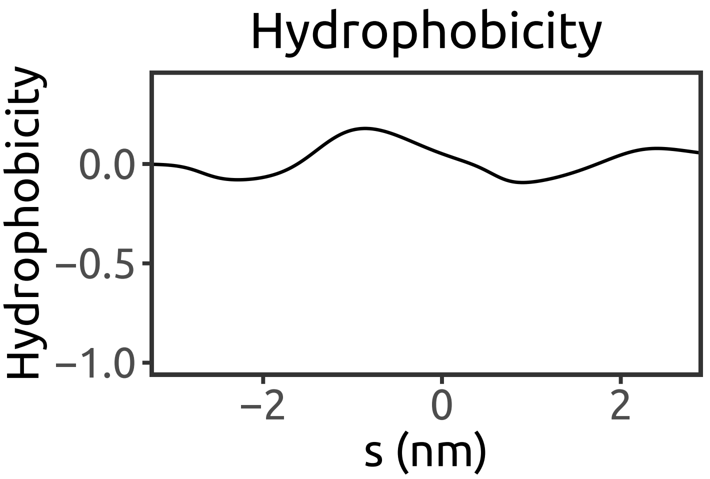


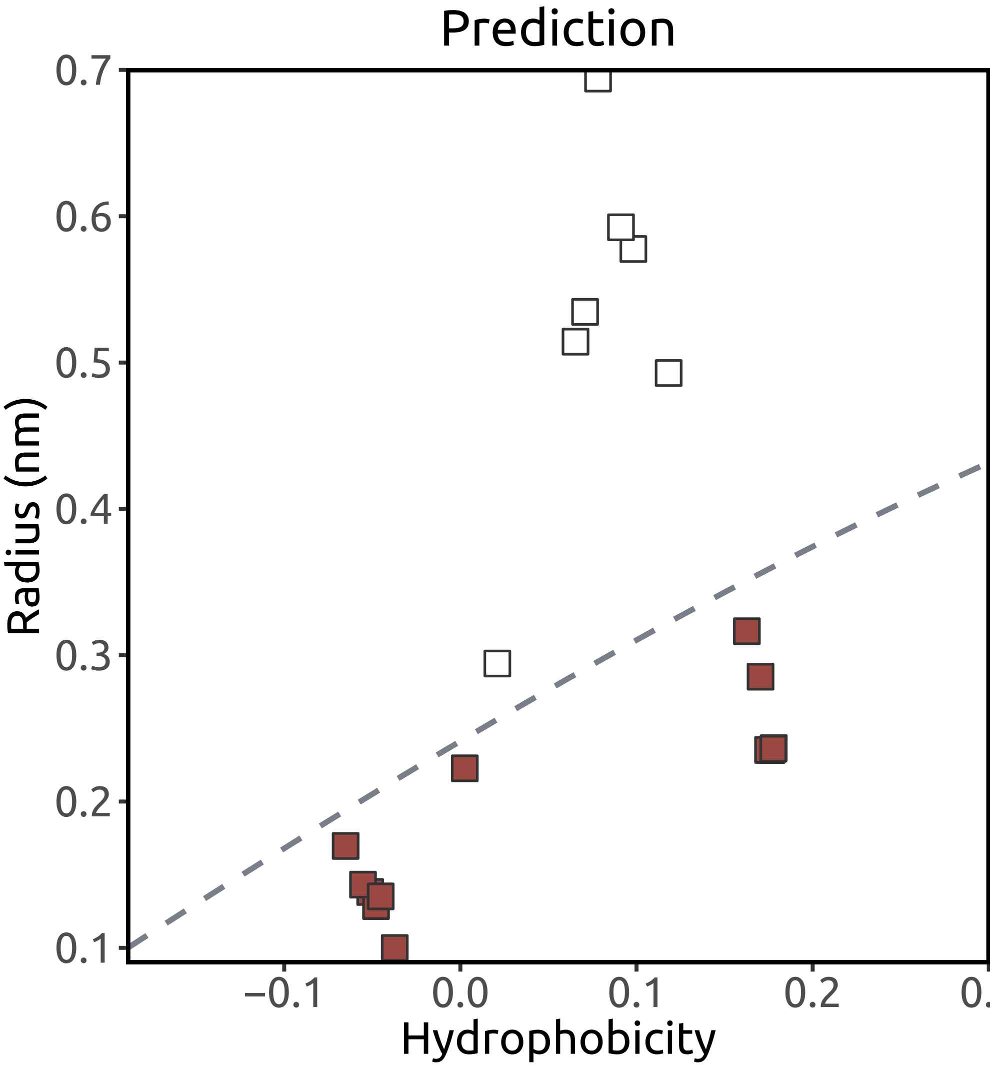


Heuristic score:
1.11 (n = 15)

Simulation result:
hydrated channel

# NavAb (PDB ID: 5EK0)

*Arcobacter butzleri*
X−ray (3.53 Å)

Ahuja et al., 2015

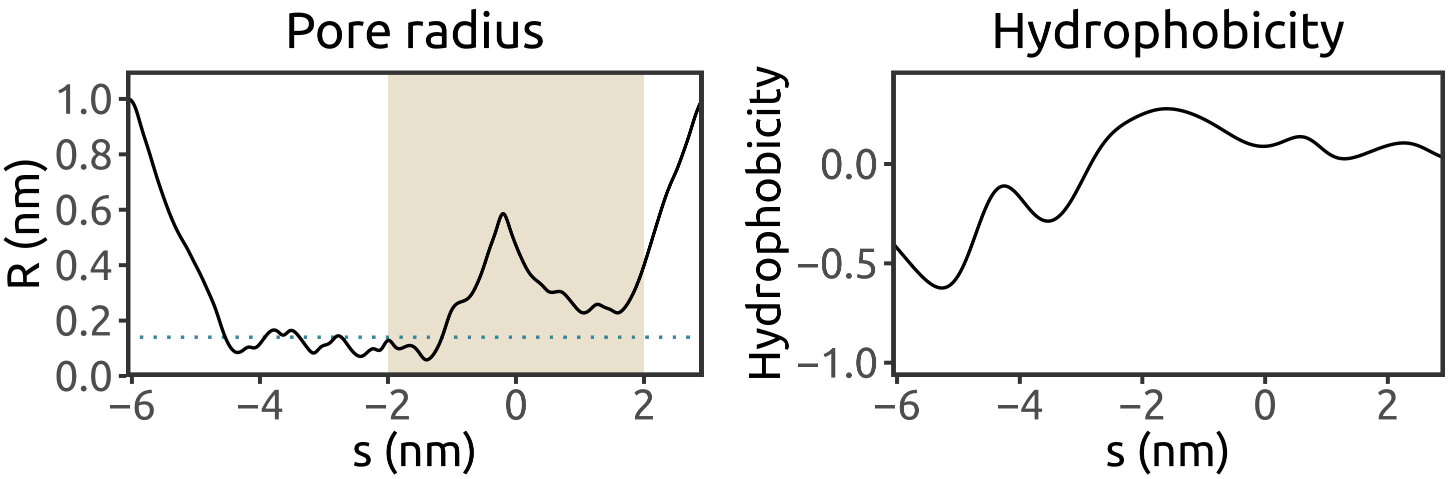


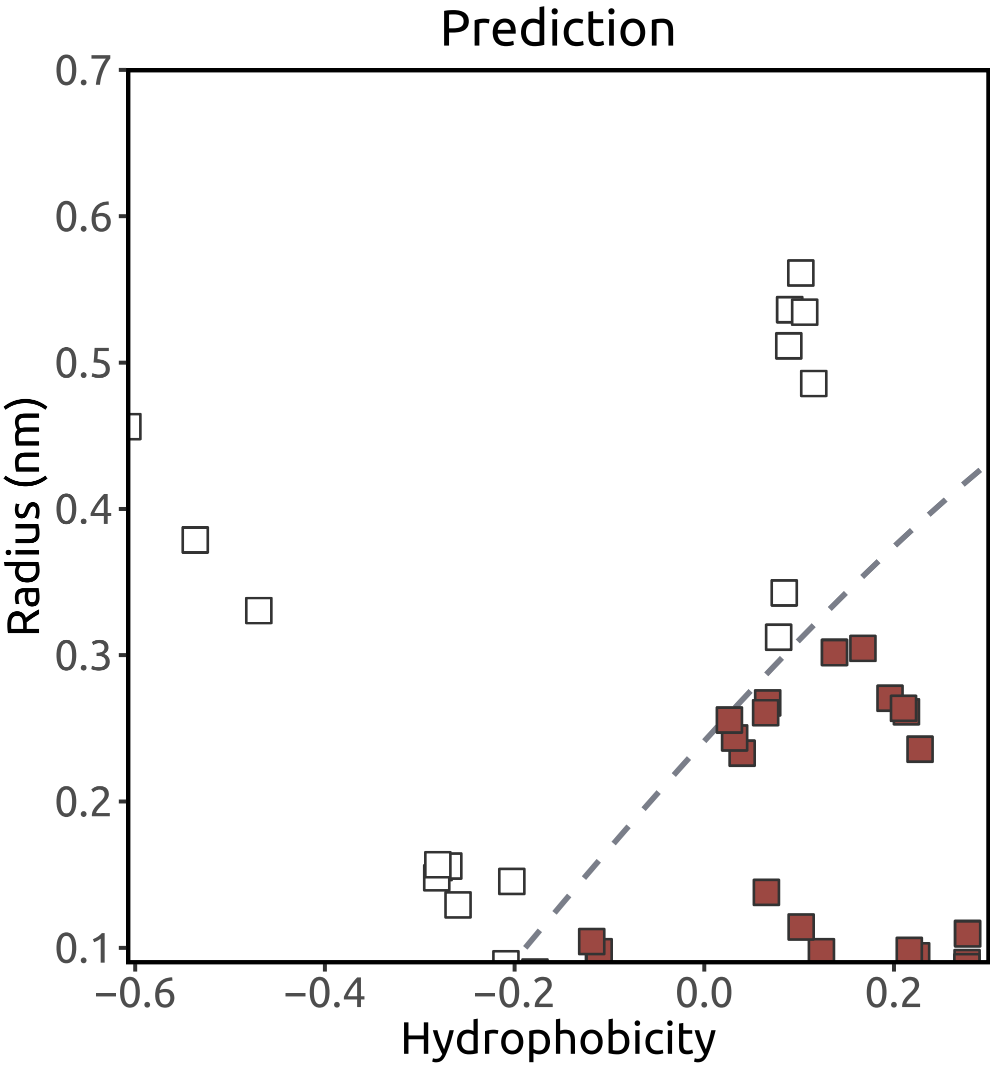


Heuristic score:

3.4 (n = 28)

Simulation result:

barrier to water

# NavAb (PDB ID: 5VB2)

*Arcobacter butzleri*
X–ray (3.2 Å)

Lenaeus et al., 2017

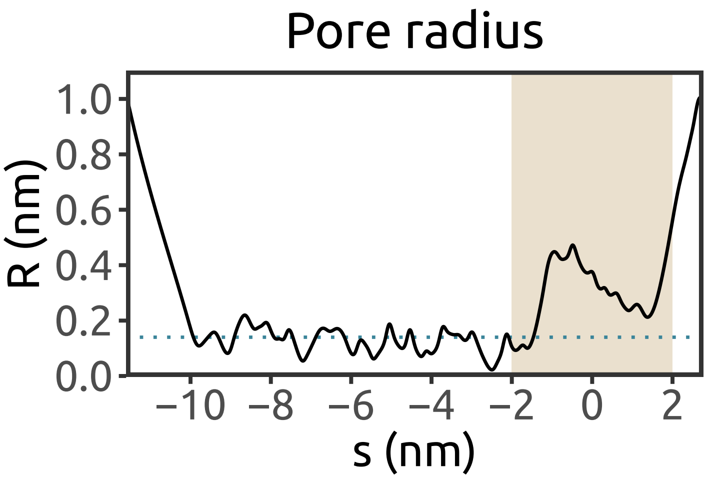


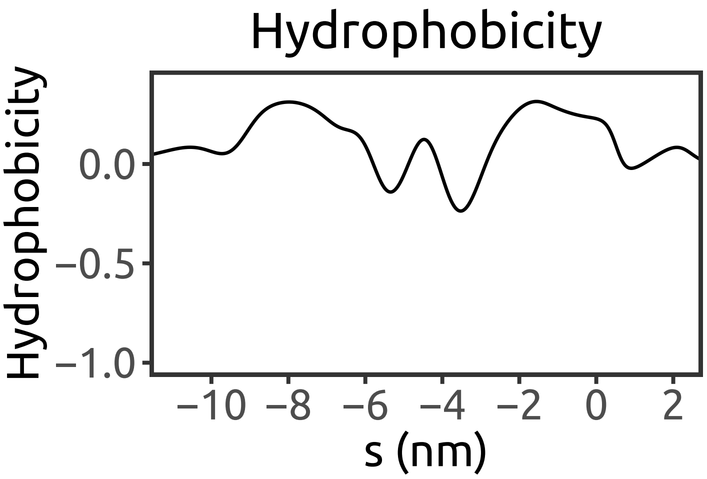


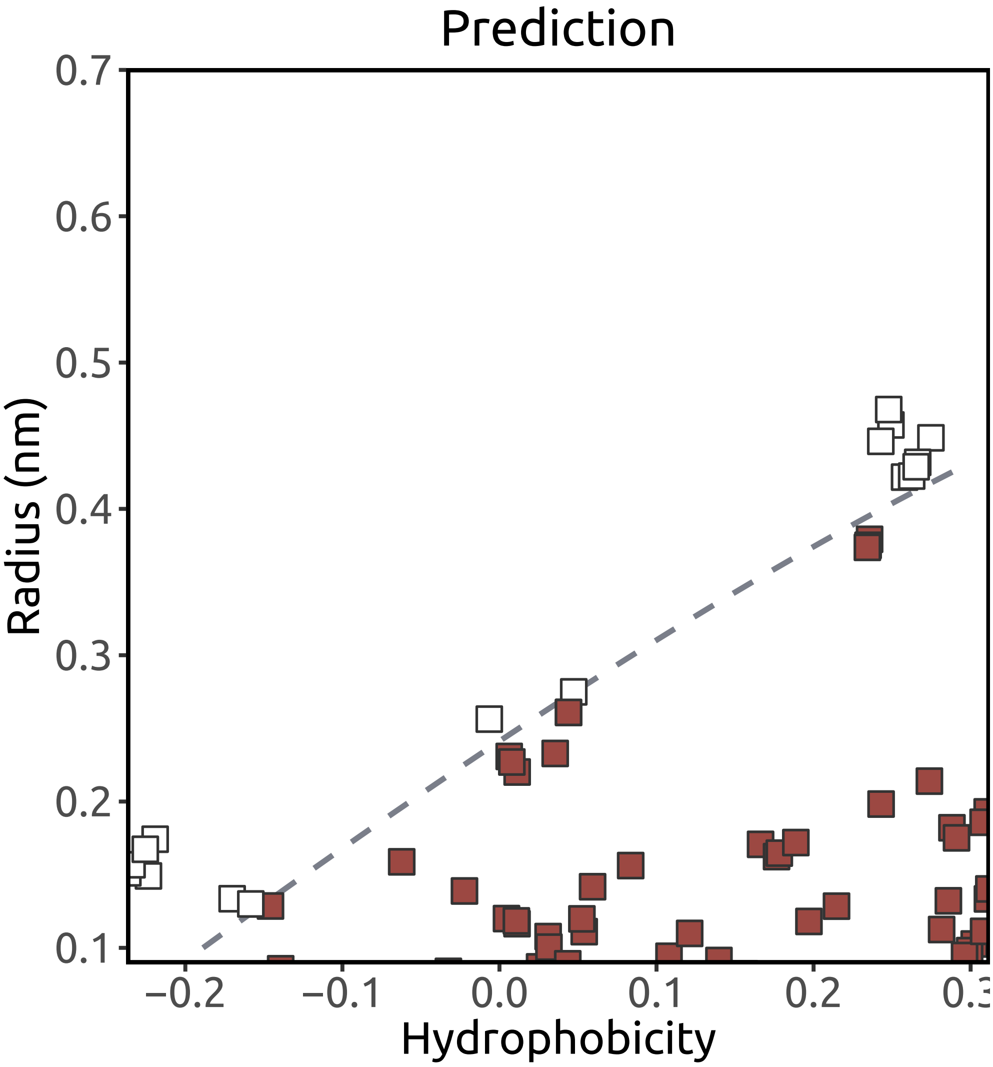


Heuristic score:
9.06 (n = 61)

Simulation result:
barrier to water

# NavAb (PDB ID: 5VB8)

*Arcobacter butzleri*
X–ray (2.85 Å)

Lenaeus et al., 2017

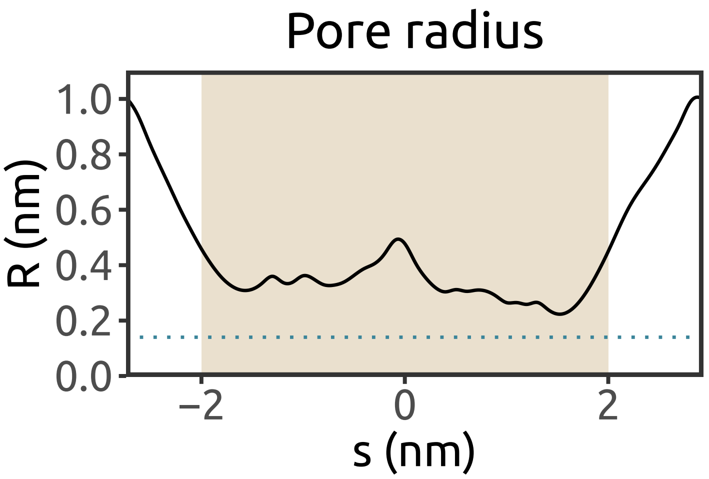


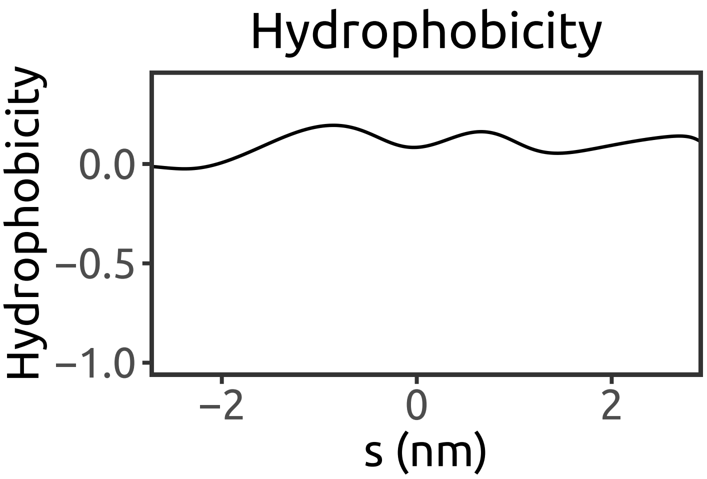


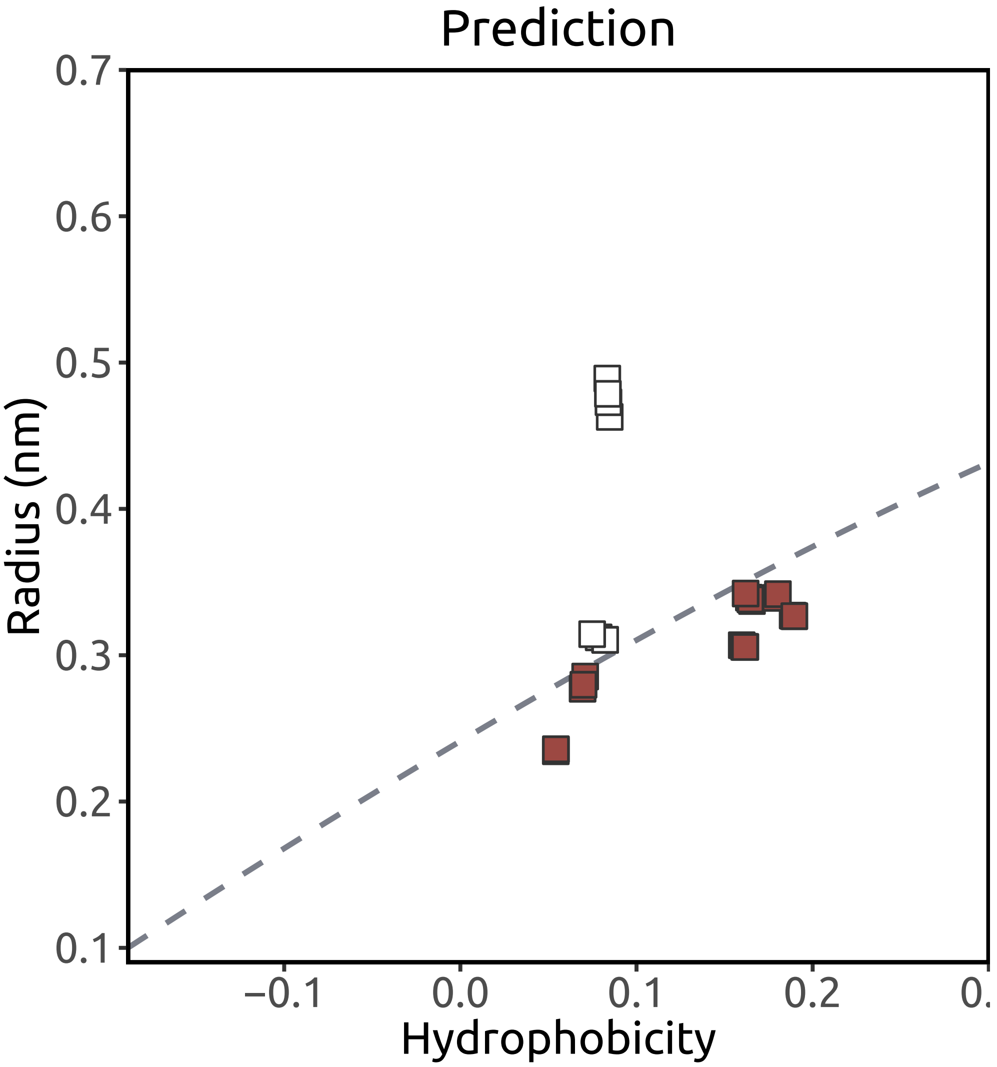


Heuristic score:

0.51 (n = 21)

Simulation result:

barrier to water

# NavAe1 (PDB ID: 4LTO)

*Alkalilimnicola ehrlichii*
X−ray (3.46 Å)

Shaya et al., 2014

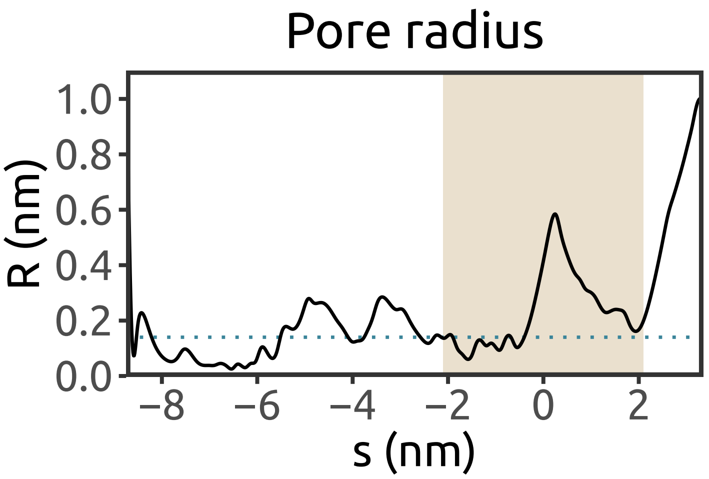


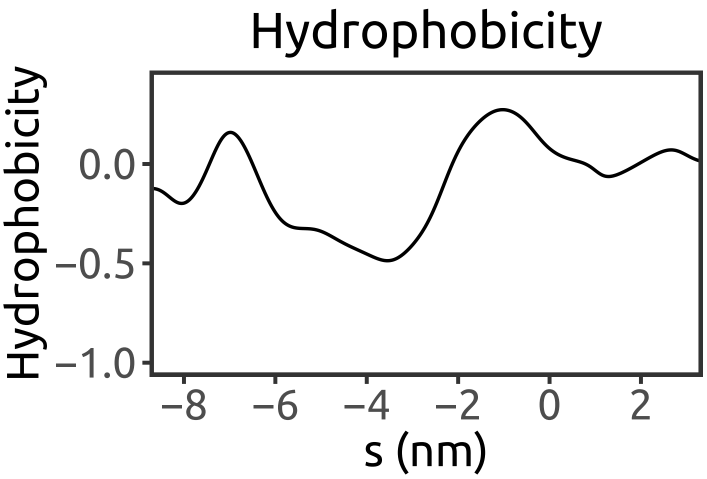


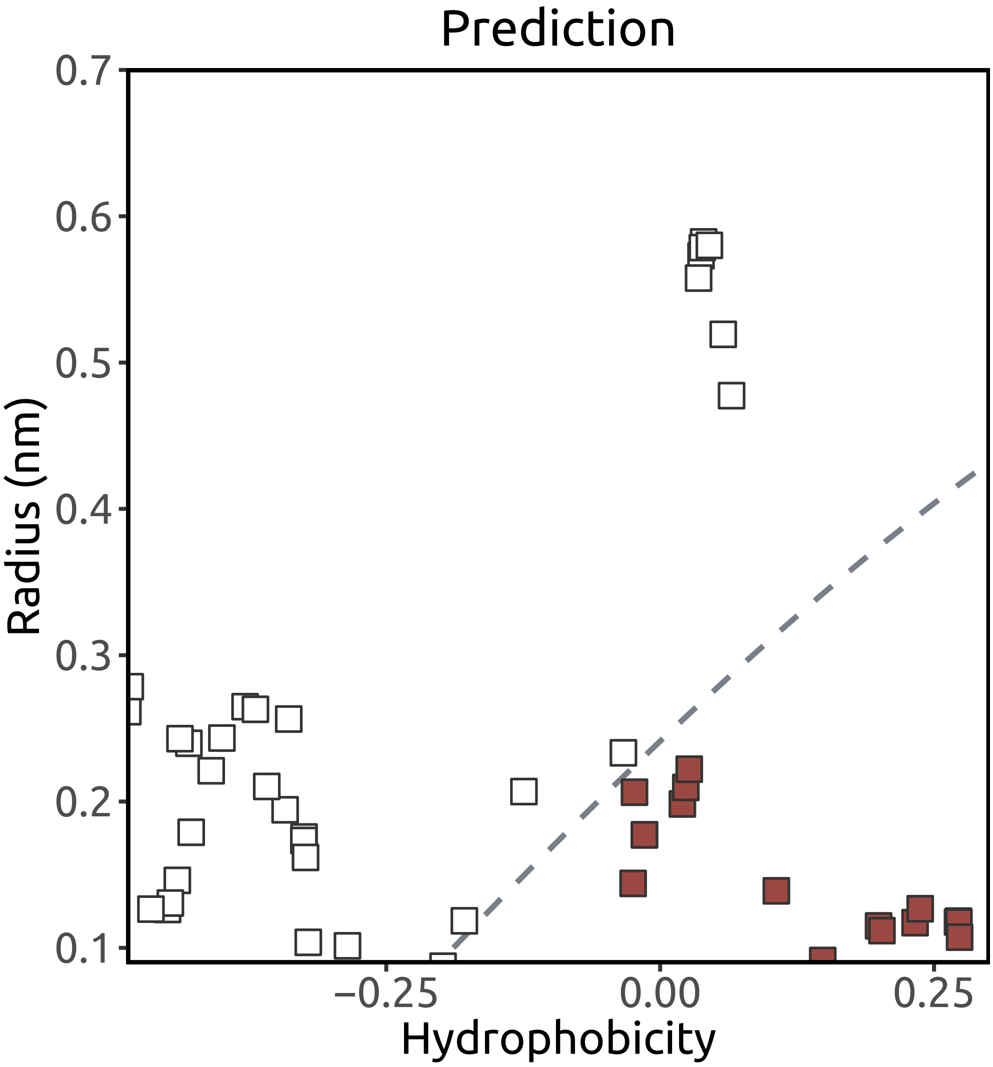


Heuristic score:
3.76 (n = 24)

Simulation result:
barrier to water

# NavMm (PDB ID: 4CBC)

*Magnetococcus marinus*
X–ray (2.66 Å)

Bagnéris et al., 2014

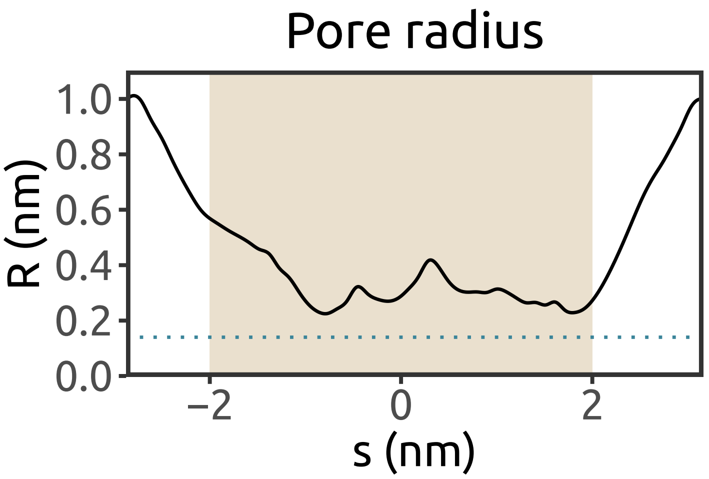


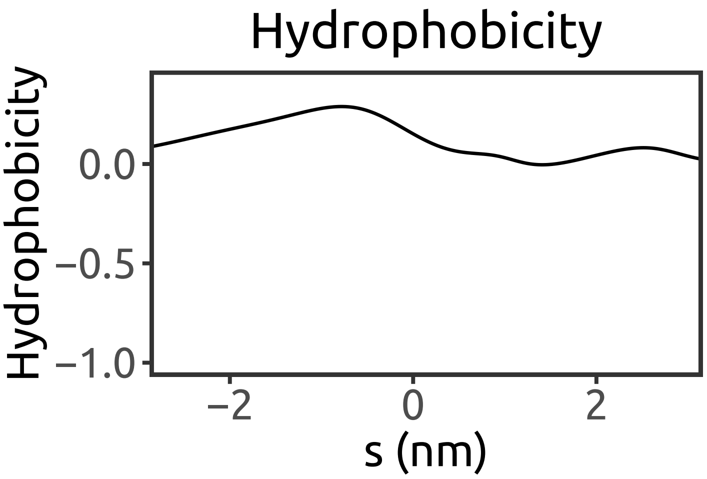


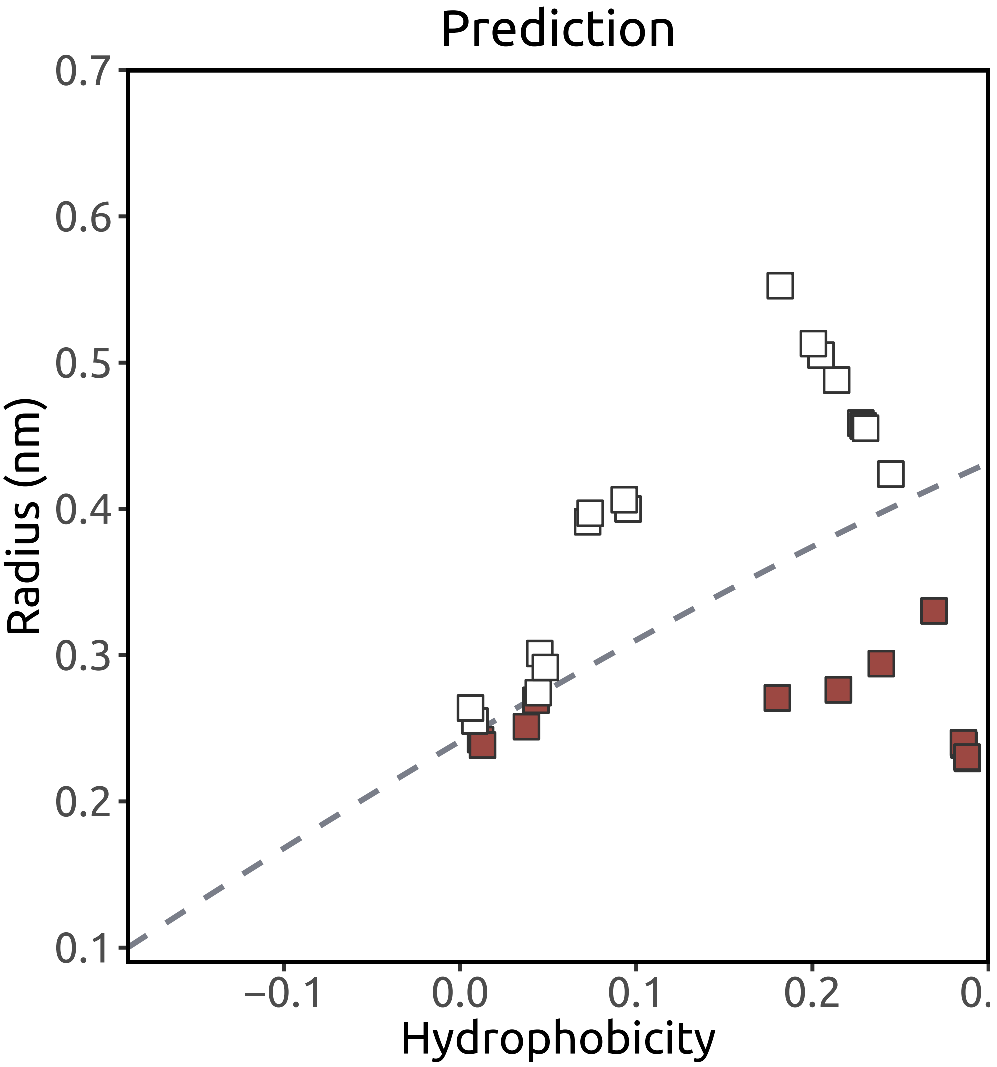


Heuristic score:
1.11 (n = 13)

Simulation result:
barrier to water

# NavMm (PDB ID: 4OXS)

*Magnetococcus marinus*
X–ray (2.8 Å)

Bagnéris et al., 2014

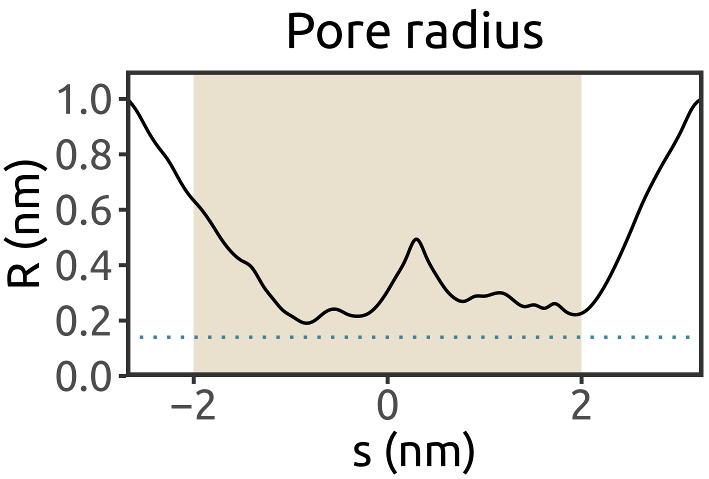


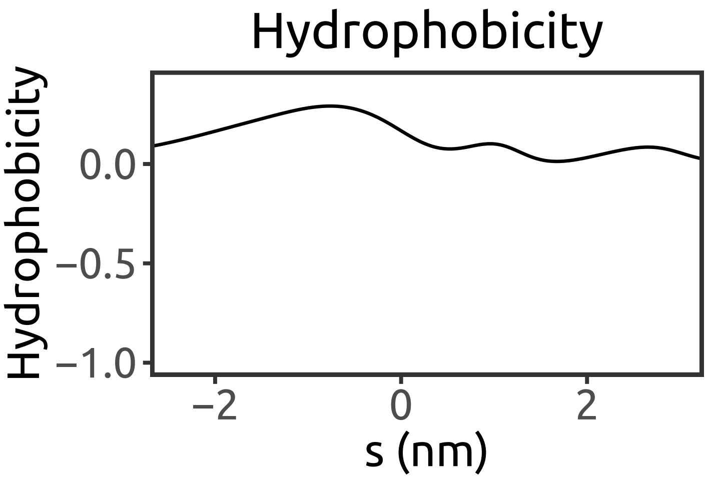


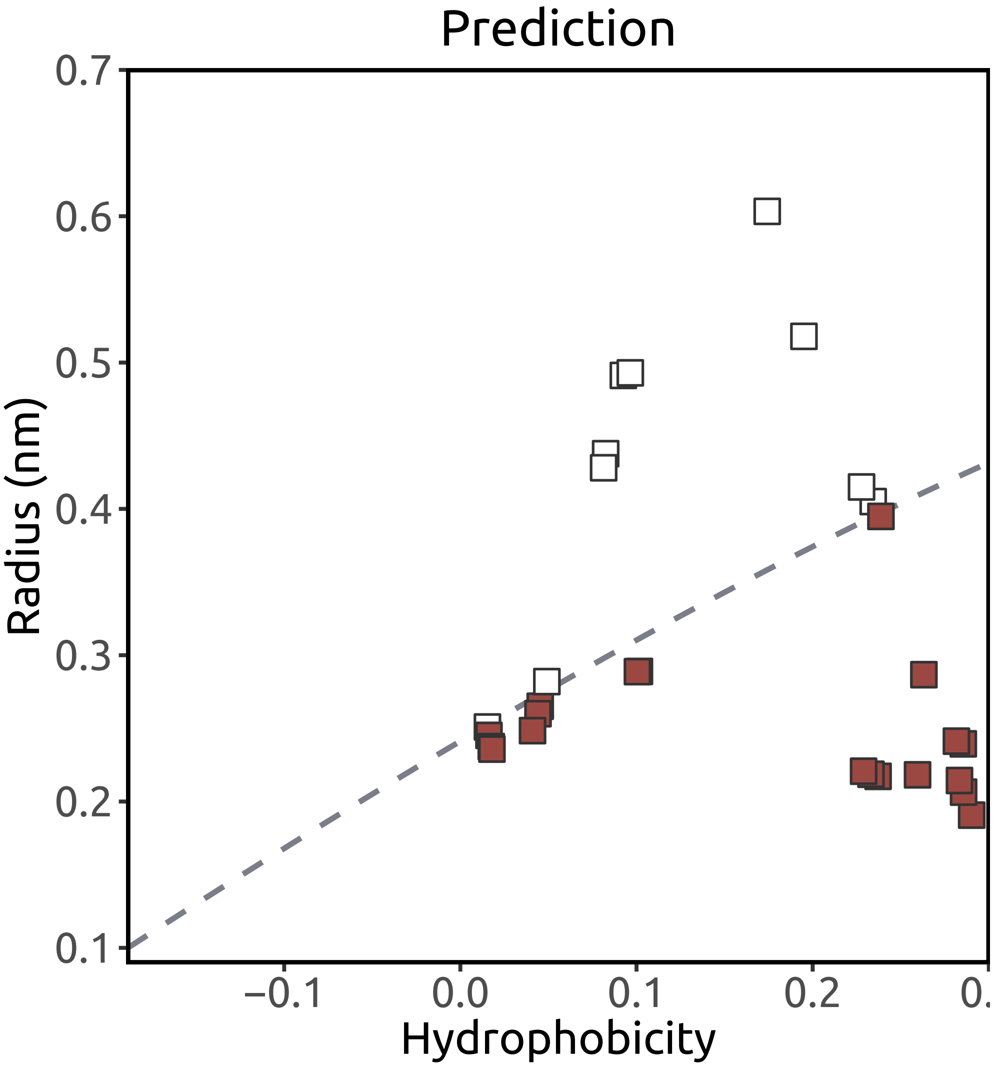


Heuristic score:
1.72 (n = 19)

Simulation result:
barrier to water

# NavPa (PDB ID: 5X0M)

*Periplaneta americana*
cryo–EM (3.8 Å)

Shen et al., 2017

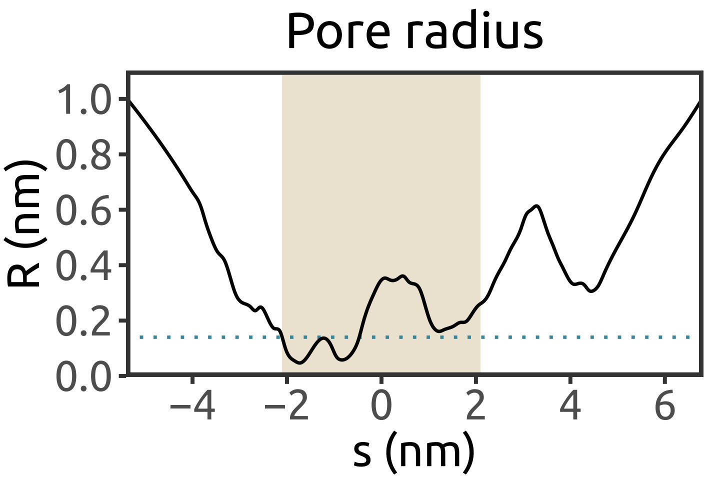


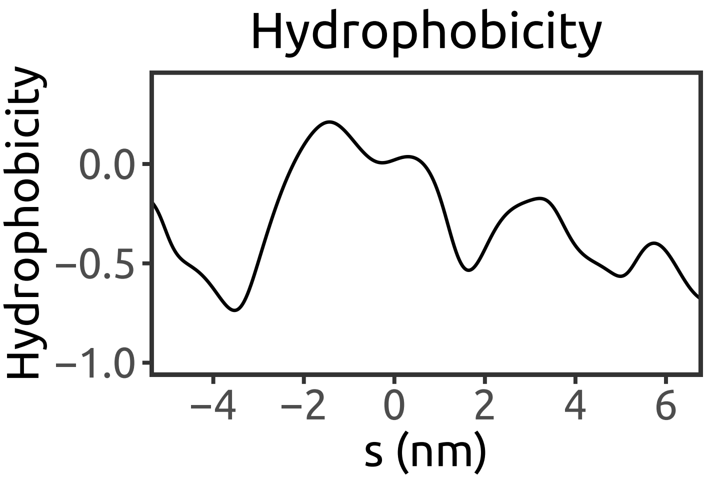


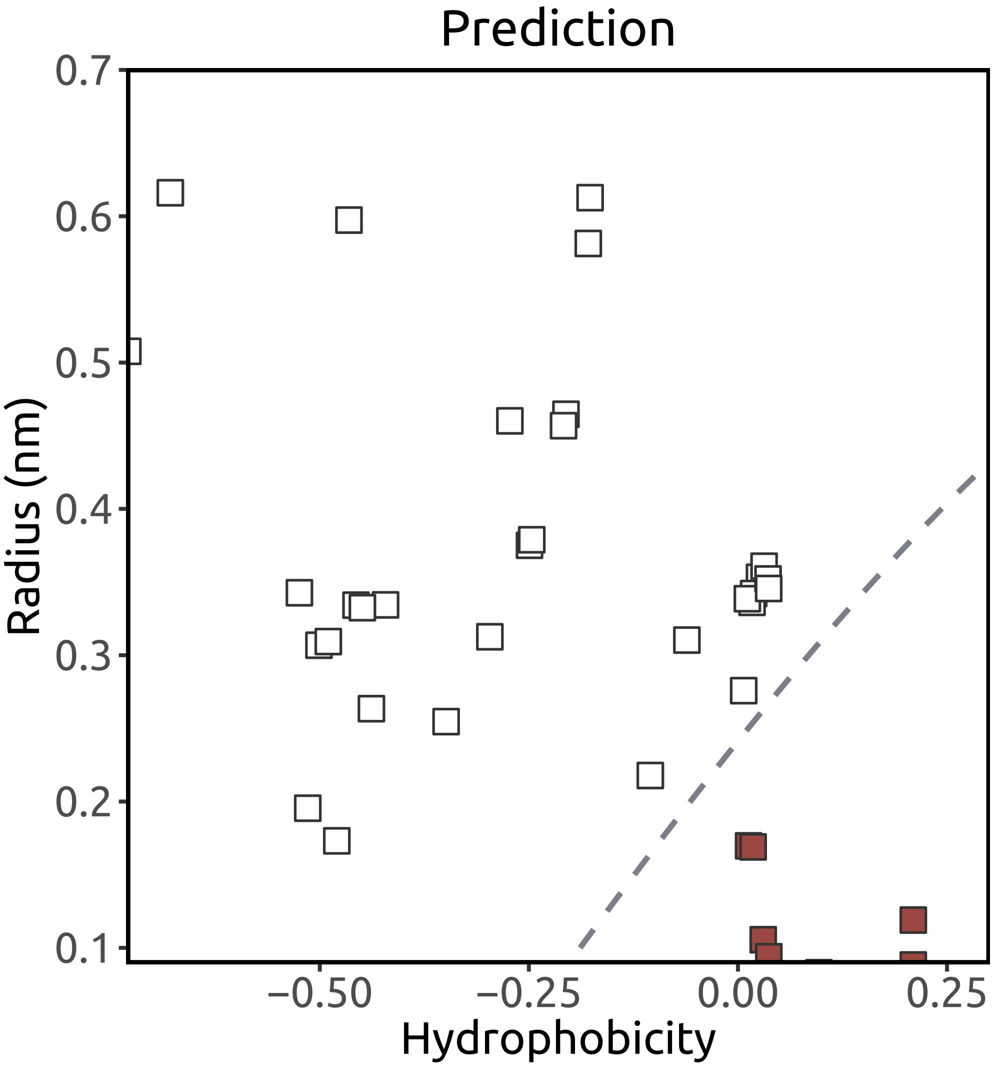


Heuristic score:
1.91 (n = 11)

Simulation result:
barrier to water

# NavRh (PDB ID: 4DXW)

*Rickettsiales sp. HIMB114*
X−ray (3.05 Å)

Zhang et al., 2012

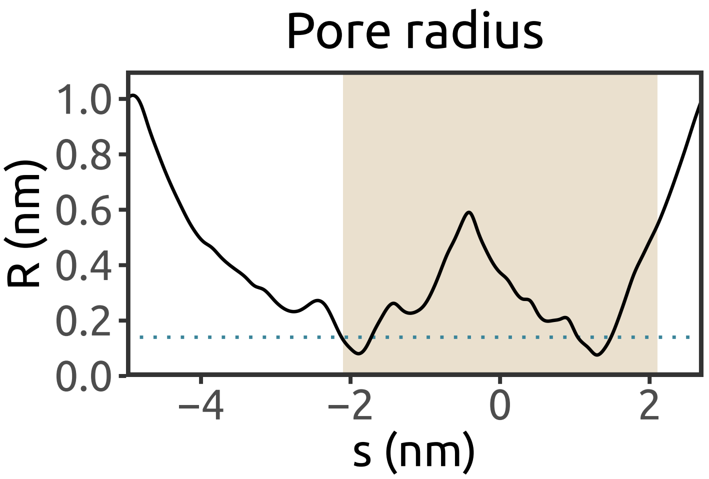


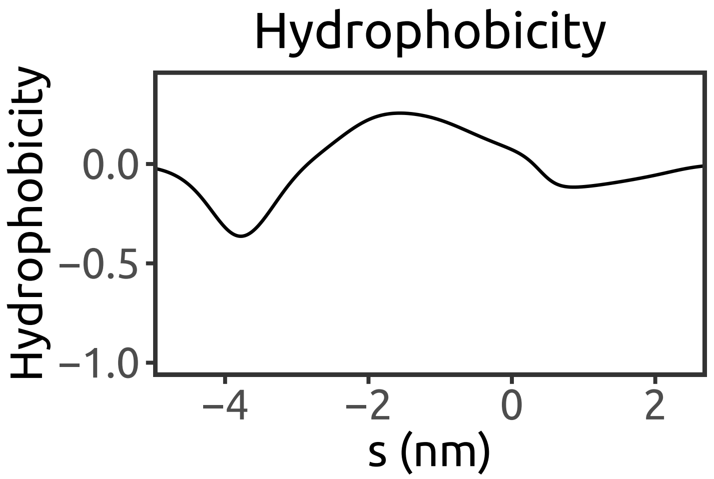


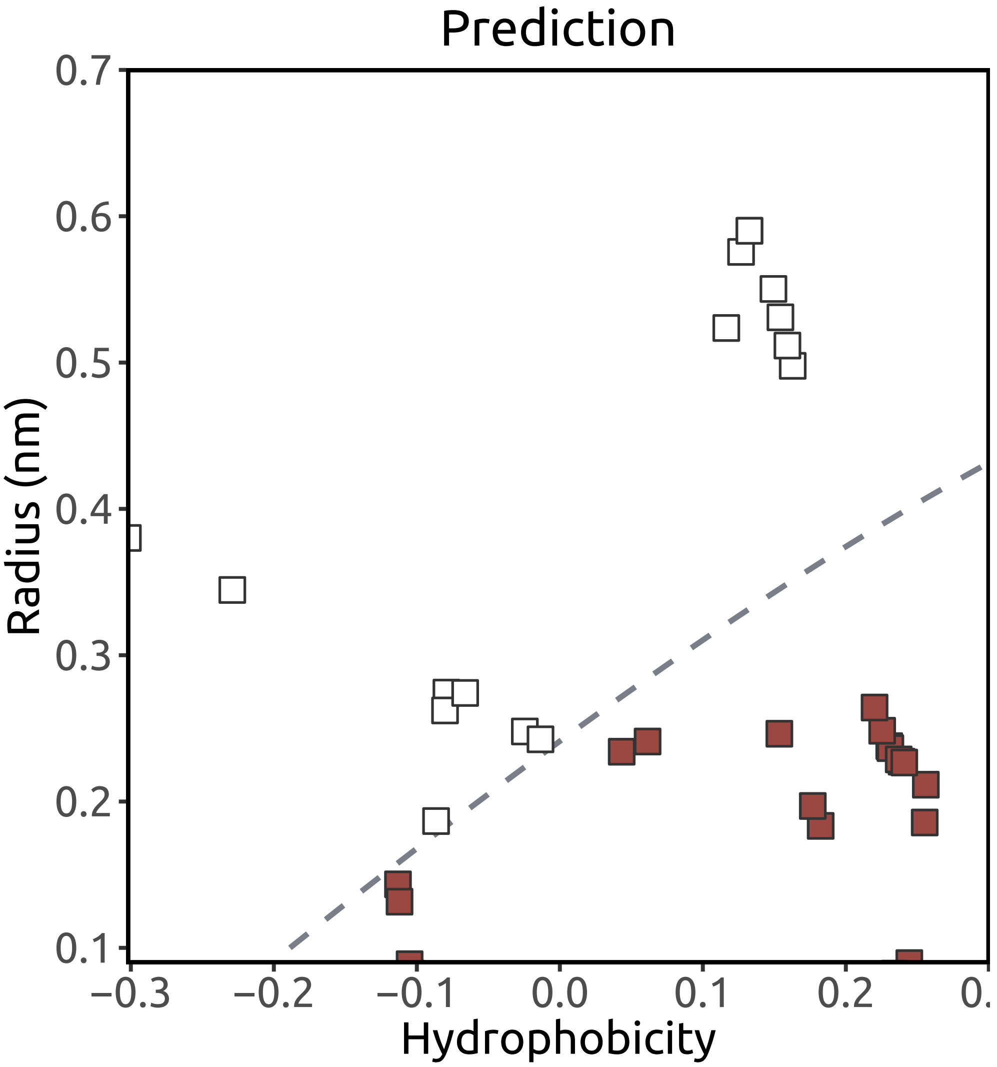


Heuristic score:

2.67 (n = 21)

Simulation result:

barrier to water

# RyR1 (PDB ID: 3J8H)

*Oryctolagus cuniculus*
cryo–EM (3.8 Å)

Yan et al., 2015

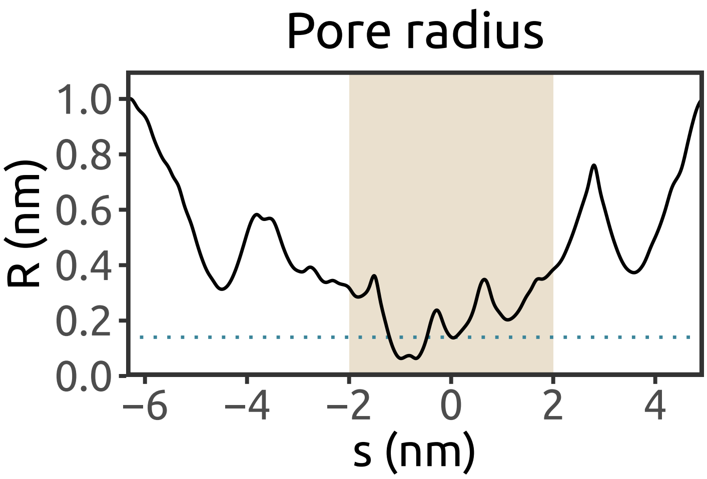


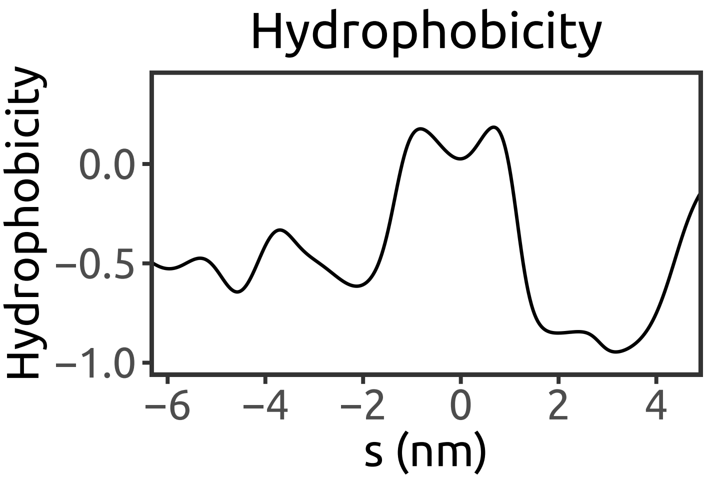


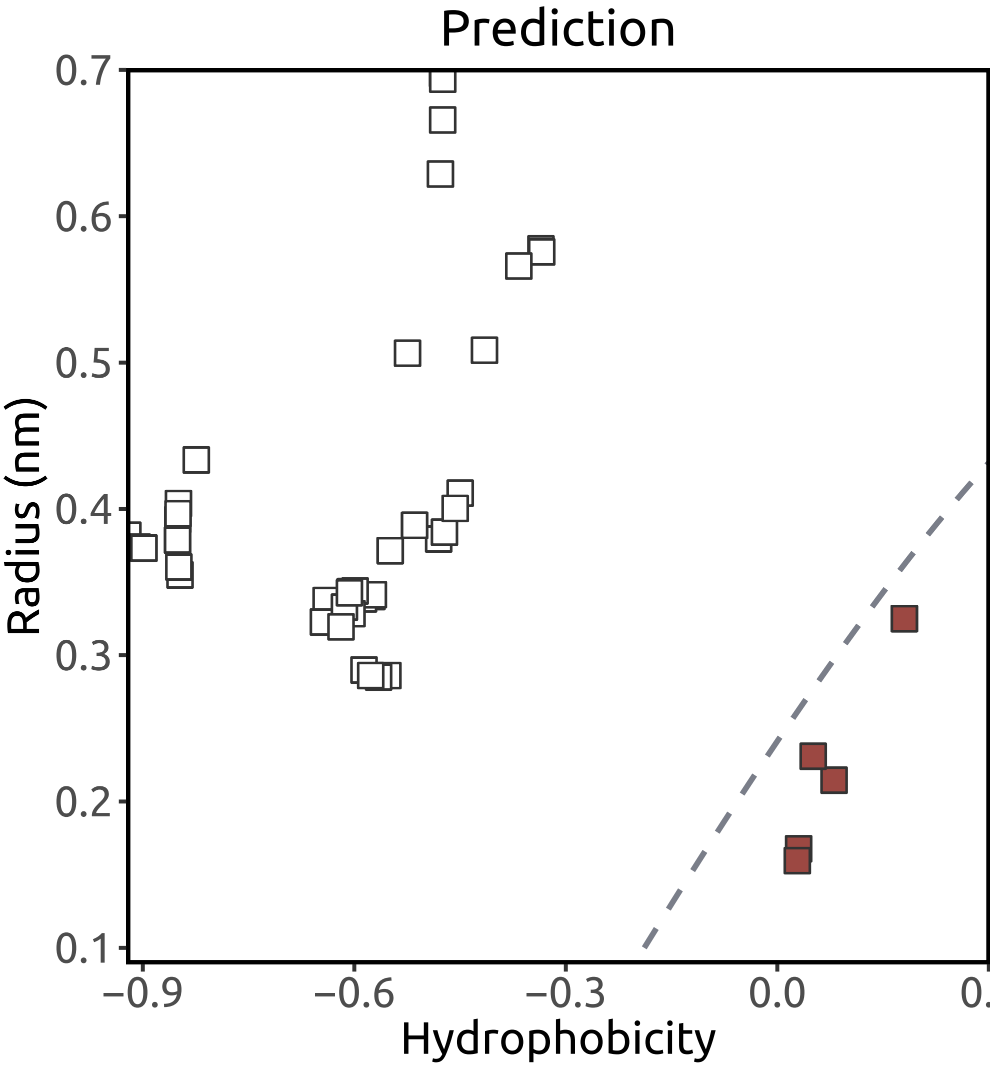


Heuristic score:
1.29 (n = 10)

Simulation result:
barrier to water

# RyR1 (PDB ID: 5TB0)

*Oryctolagus cuniculus*

cryo−EM (4.4 Å)

des et al., 2016

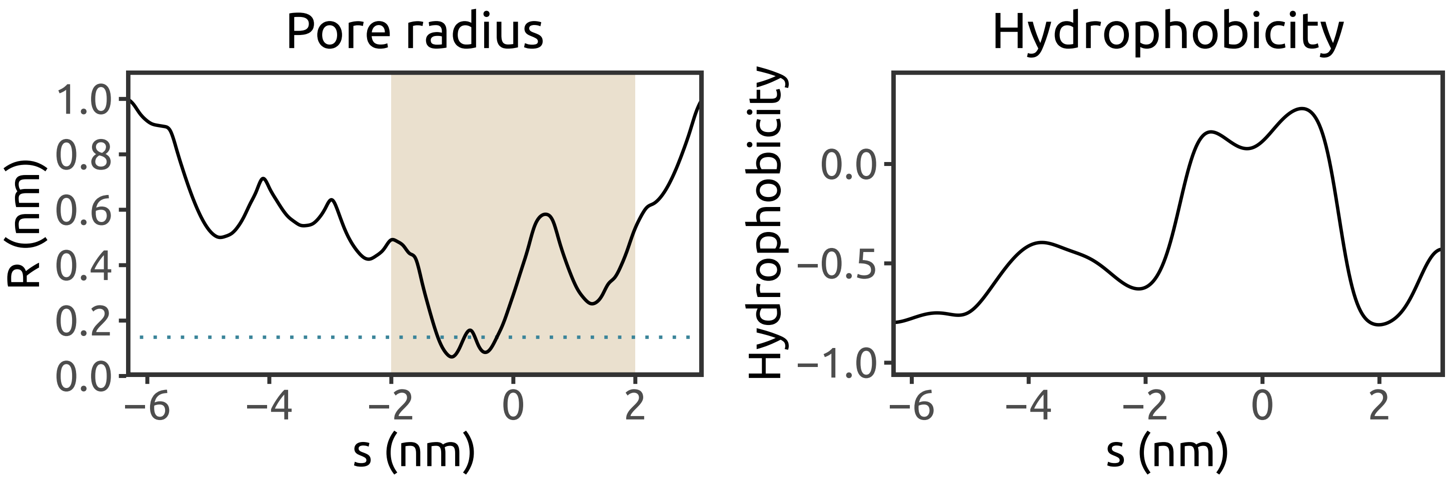


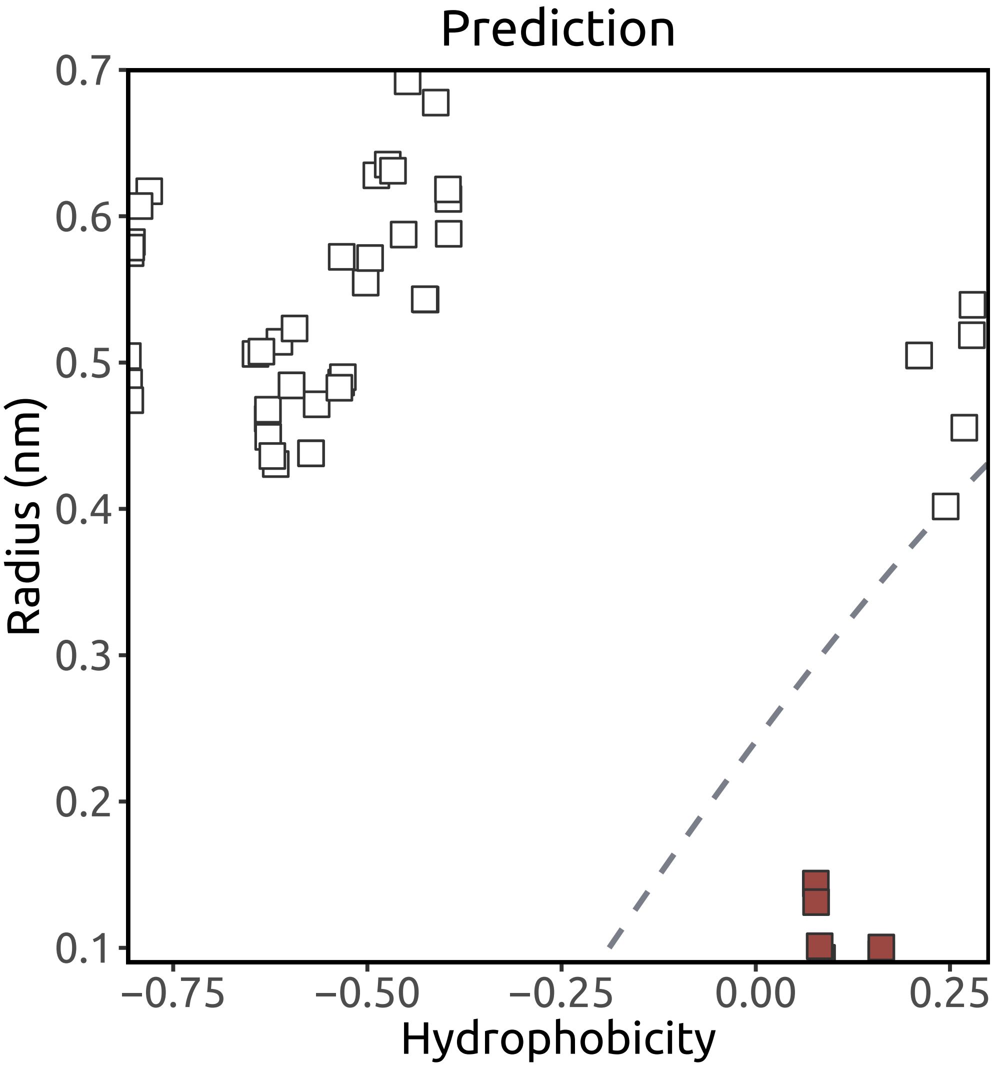


Heuristic score:

1.46 (n = 8)

Simulation result:

barrier to water

# RyR2 (PDB ID: 5GO9)

*Sus scrofa*

cryo−EM (4.4 Å)

Peng et al., 2016

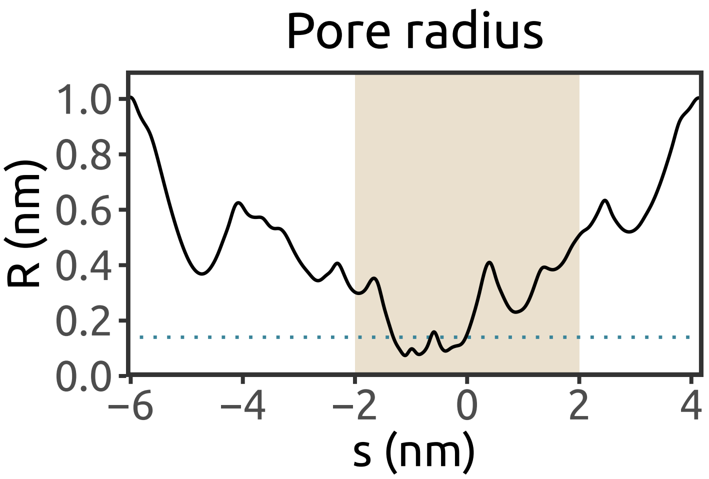


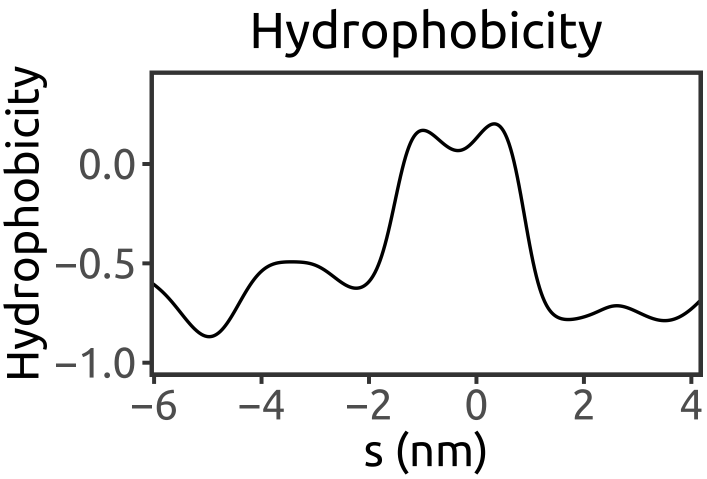


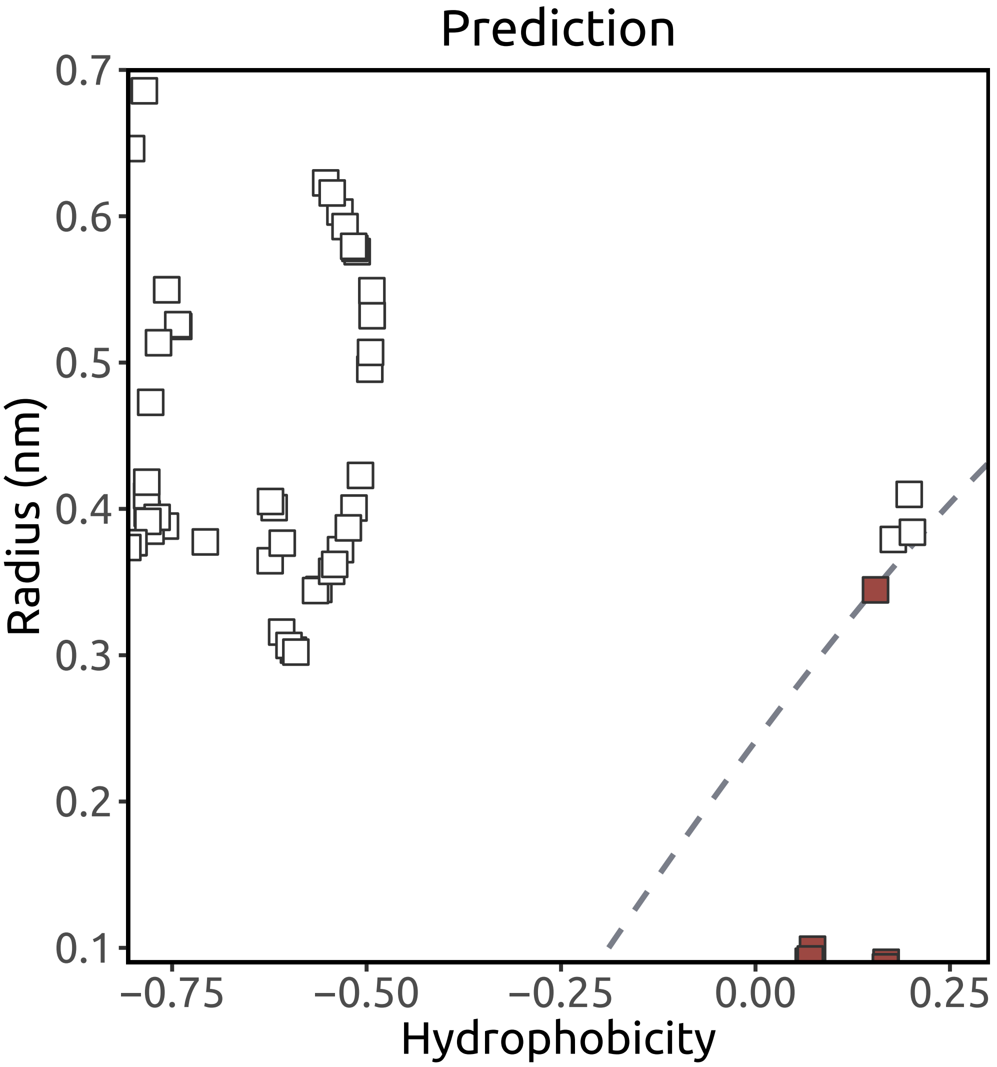


Heuristic score:

1.53 (n = 9)

Simulation result:

barrier to water

# RyR2 (PDB ID: 5GOA)

*Sus scrofa*
cryo−EM (4.2 Å)

Peng et al., 2016

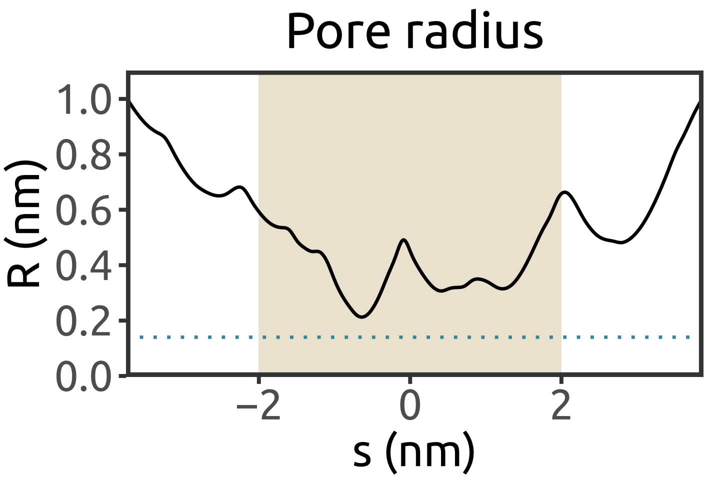


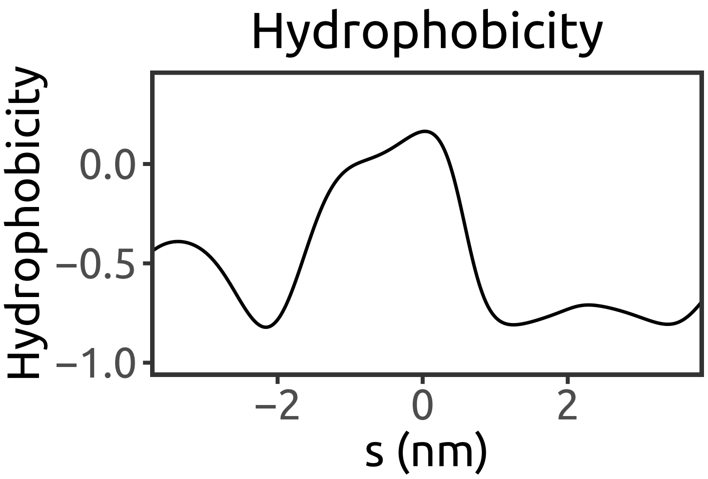


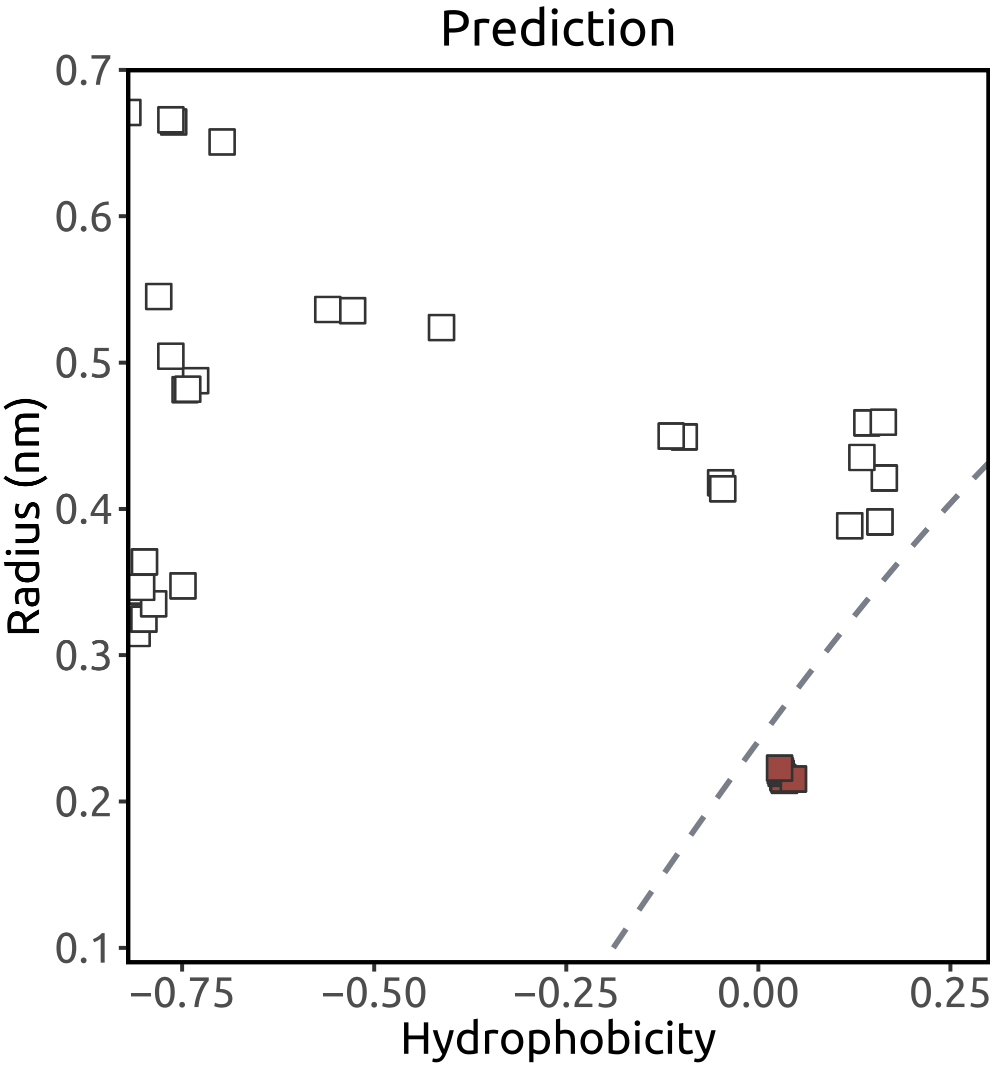


Heuristic score:
0.32 (n = 8)

Simulation result:
hydrated channel

# TPC1 (PDB ID: 5DQQ)

*Arabidopsis thaliana*
X–ray (2.87 Å)

Kintzer & Stroud, 2016

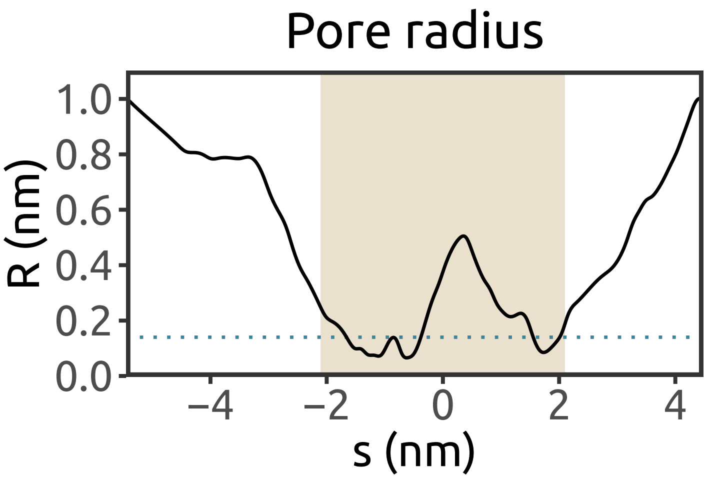


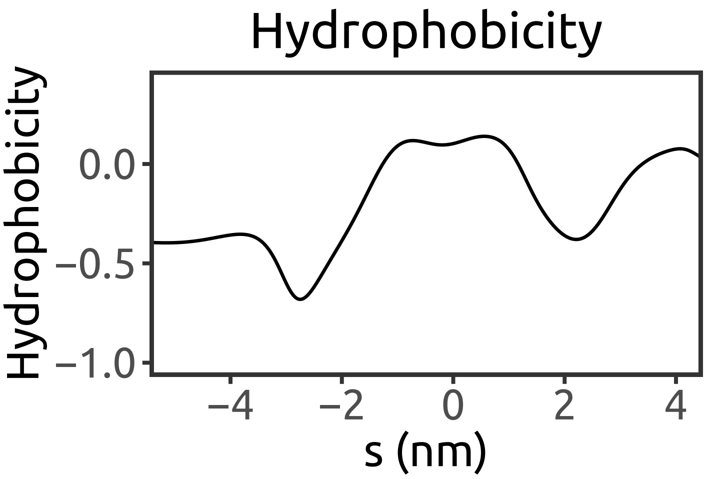


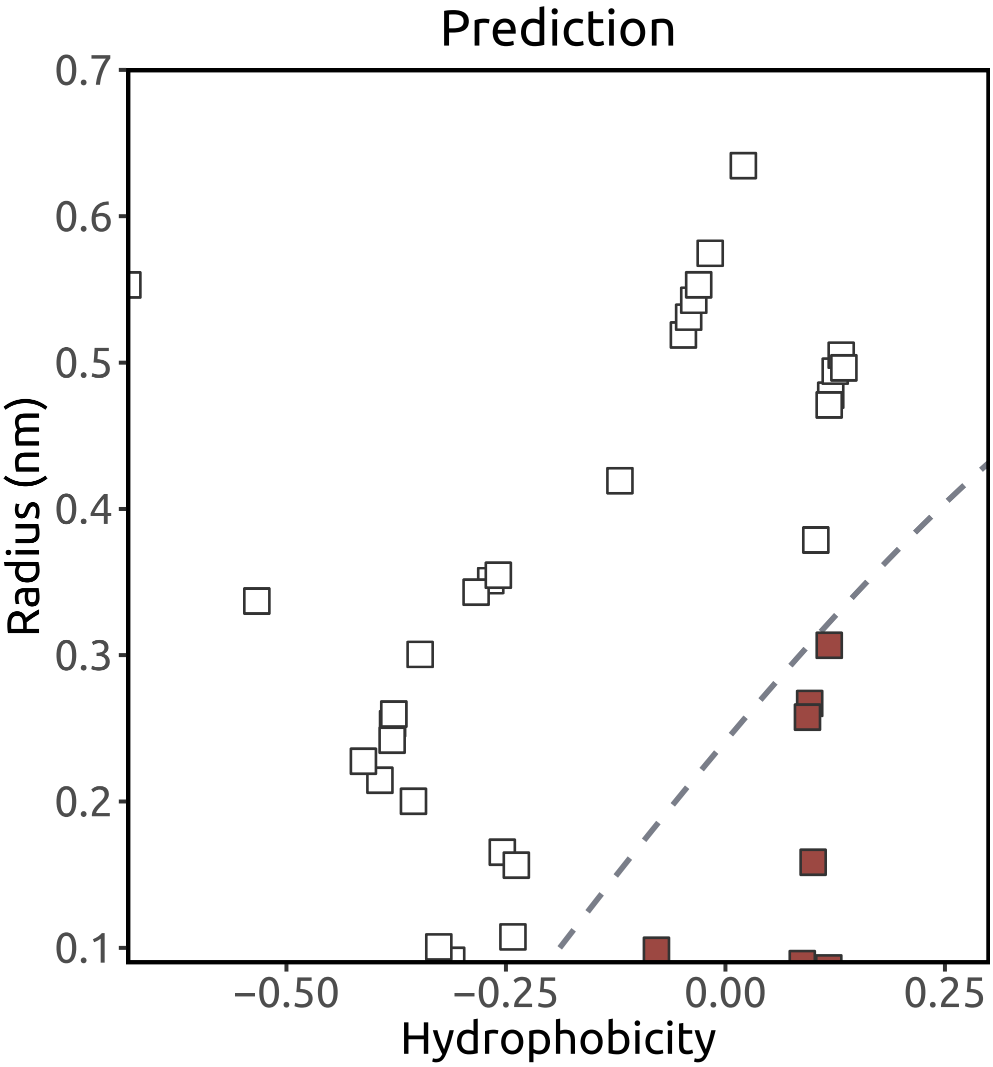


Heuristic score:

1.56 (n = 12)

Simulation result:

barrier to water

# TPC1 (PDB ID: 6C96)

*Mus musculus*
cryo−EM (3.4 Å)

She et al., 2018

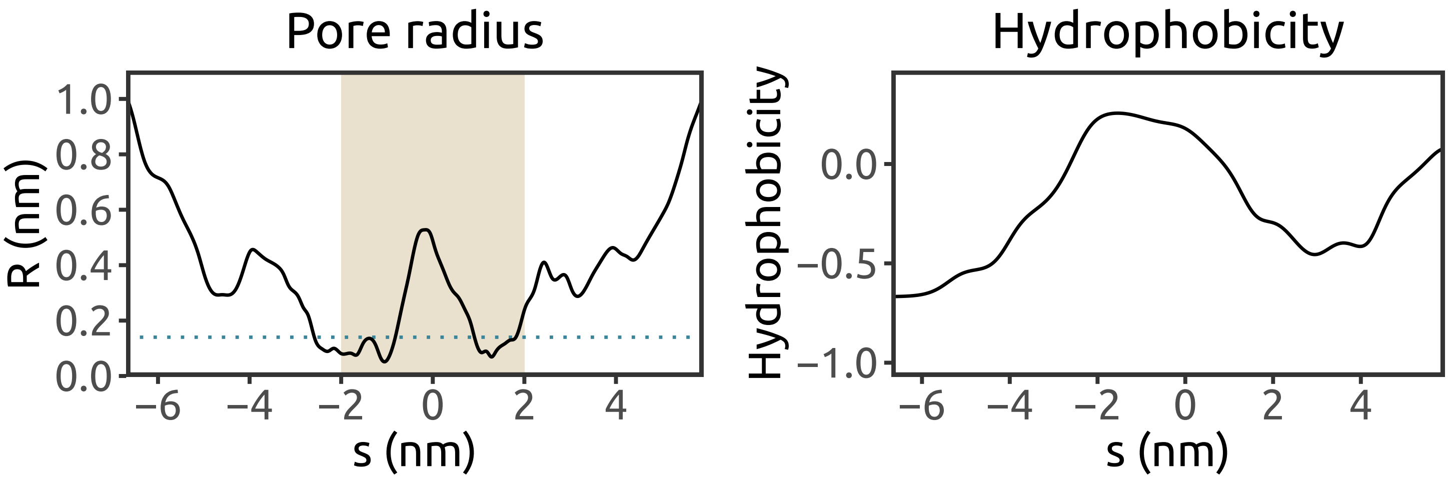


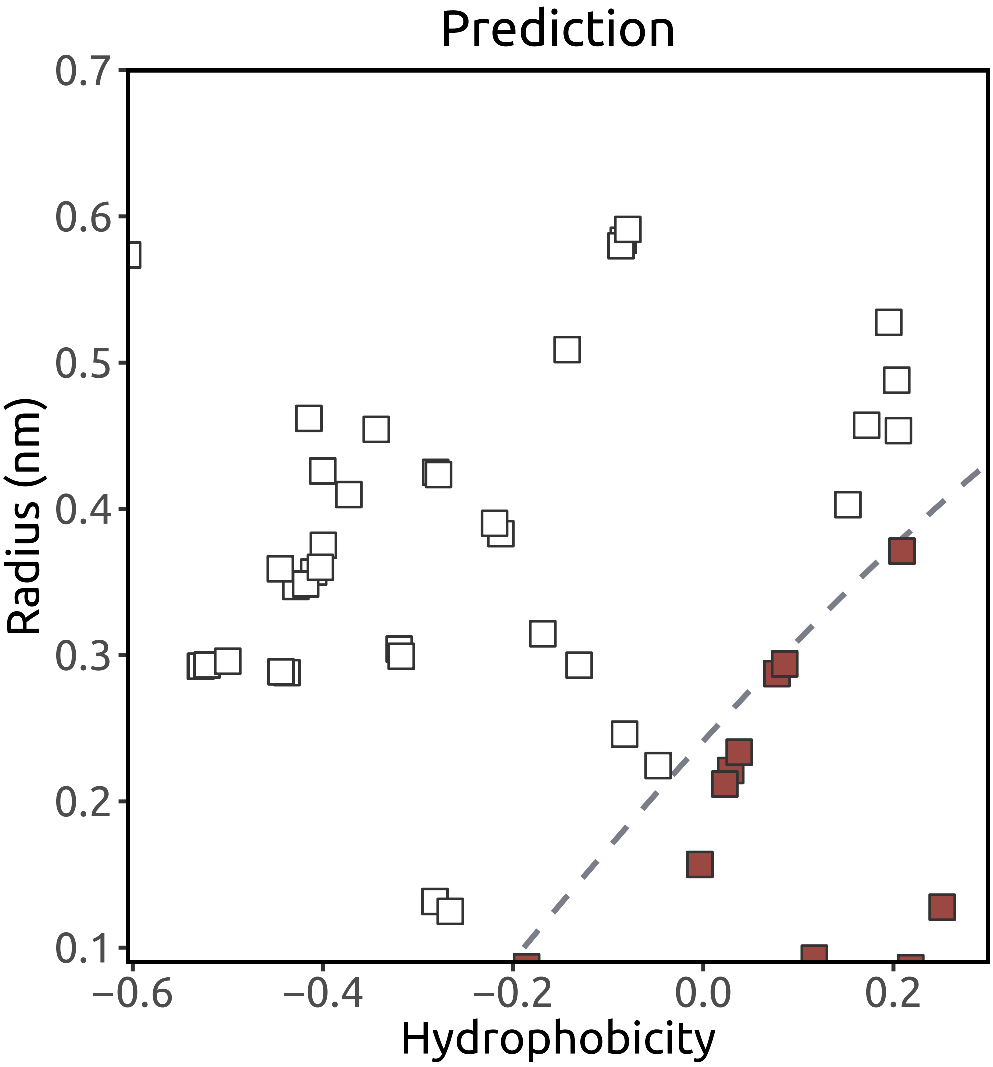


Heuristic score:
2.55 (n = 17)

Simulation result:
barrier to water

# TPC1 (PDB ID: 6C9A)

*Mus musculus*
cryo–EM (3.2 Å)

She et al., 2018

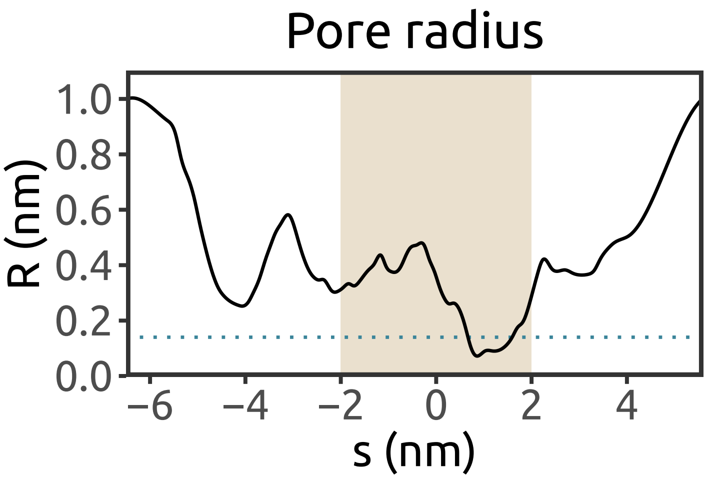


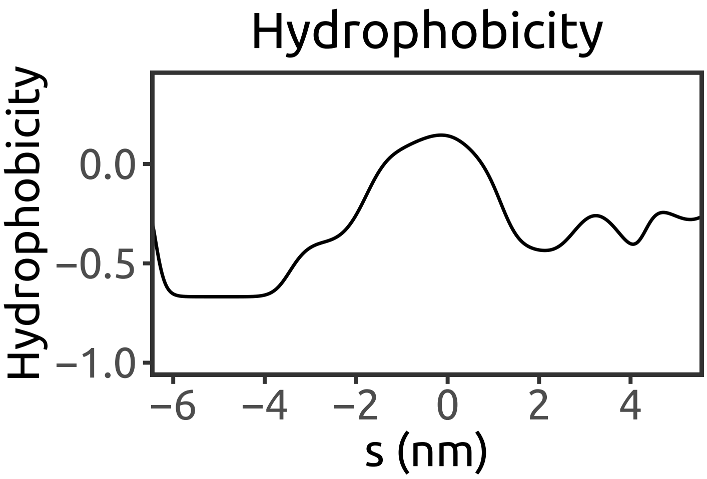


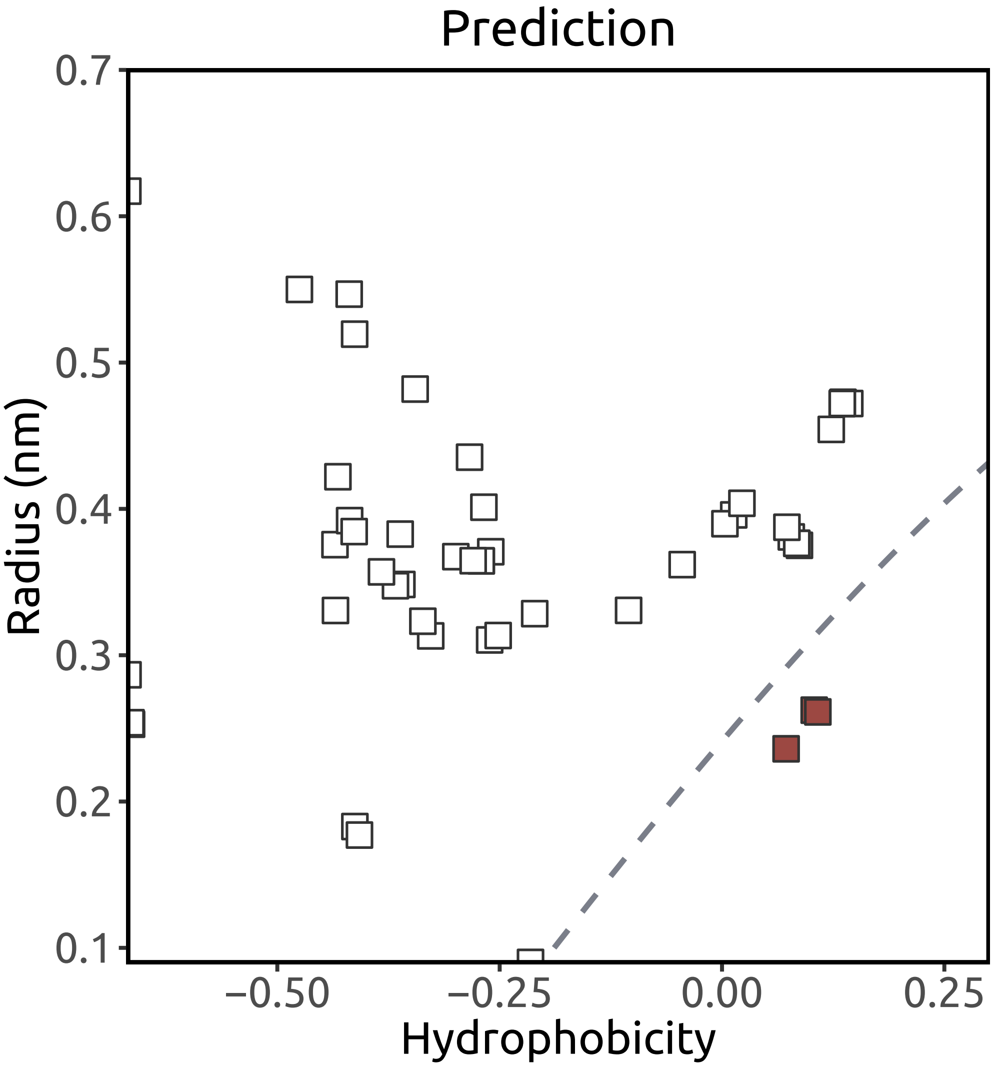


Heuristic score:
0.25 (n = 4)

Simulation result:
hydrated channel

# ASIC1 (PDB ID: 2QTS)

*Gallus gallus*
X−ray (1.9 Å)

Jasti et al., 2007

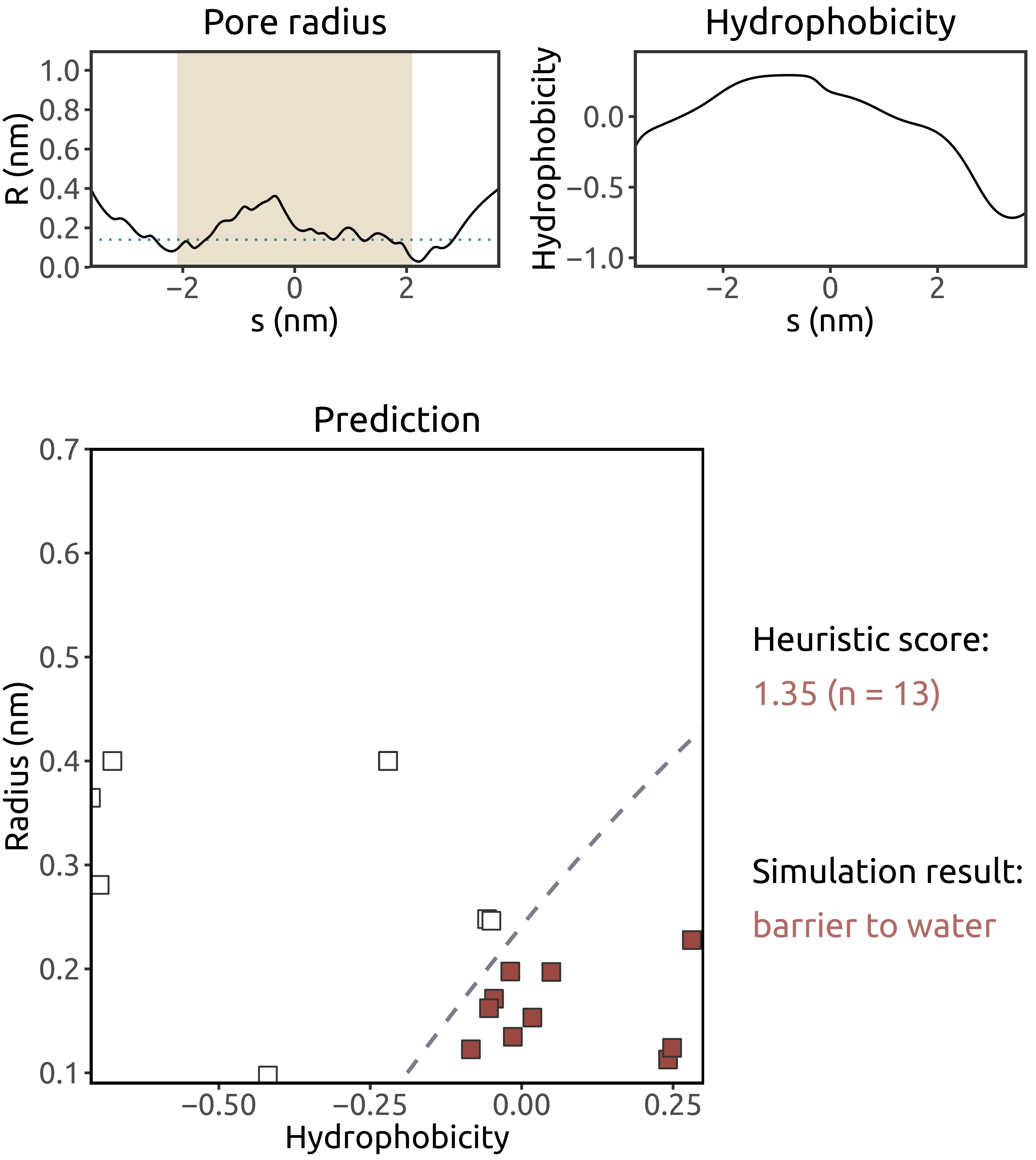


Heuristic score:
1.35 (n = 13)

Simulation result:
barrier to water

# ASIC1 (PDB ID: 3S3W)

*Gallus gallus*

X−ray (2.6 Å)

Dawson et al., 2012

Pore radius

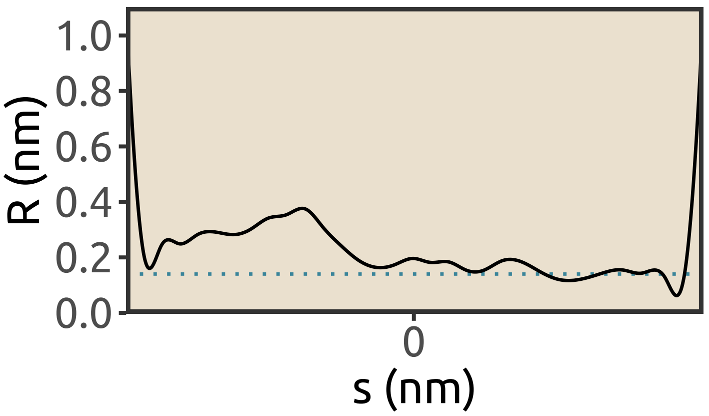


Hydrophobicity

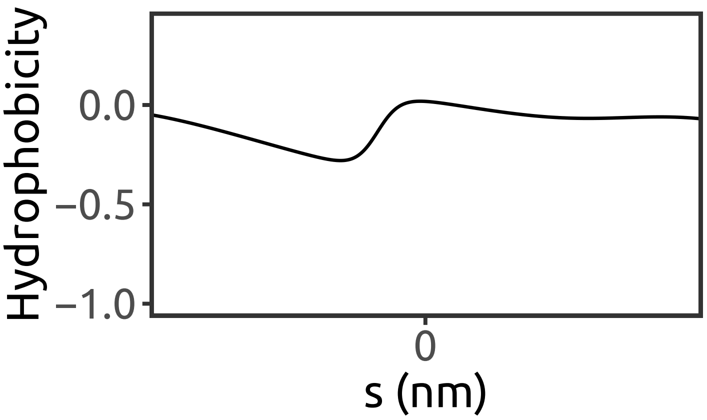


Prediction

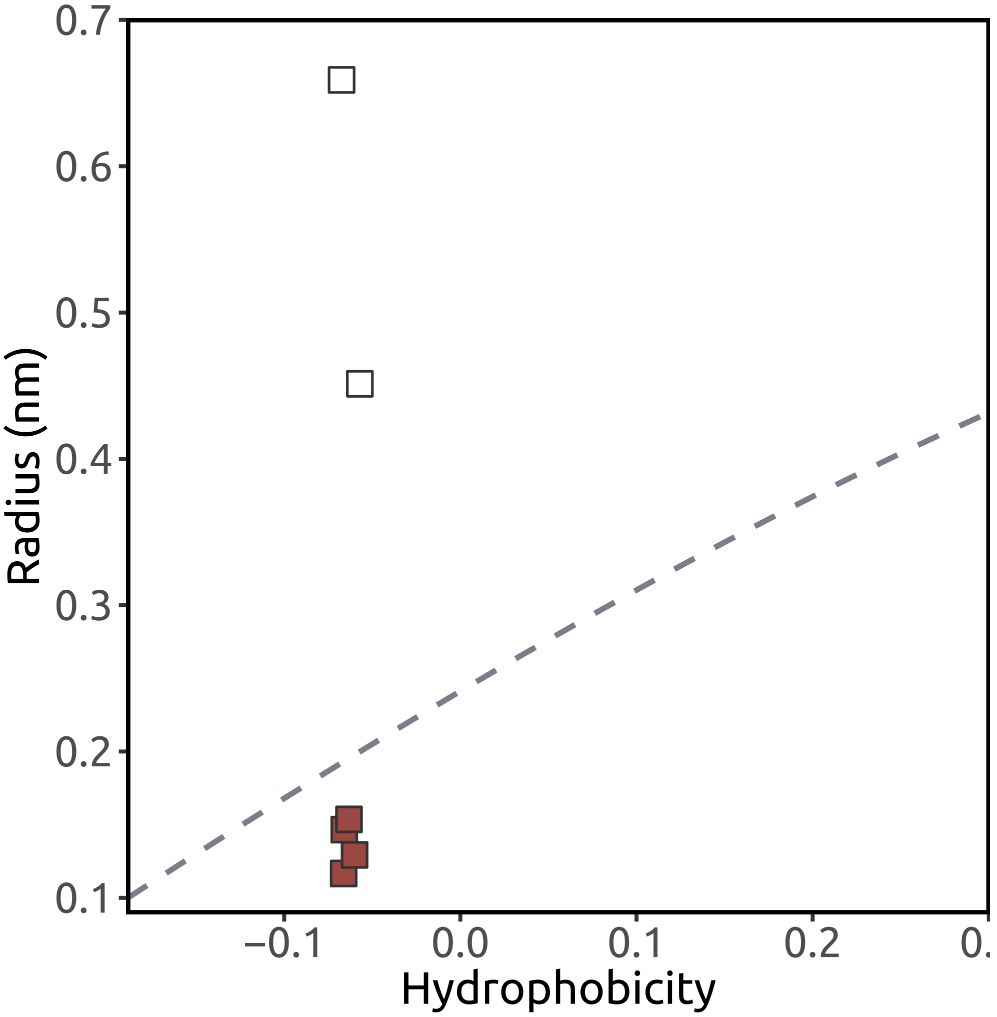


Heuristic score:

0.19 (n = 4)

Simulation result:

barrier to water

# ASIC1 (PDB ID: 4NTW)

*Gallus gallus*
X–ray (2.07 Å)

Baconguis et al., 2014

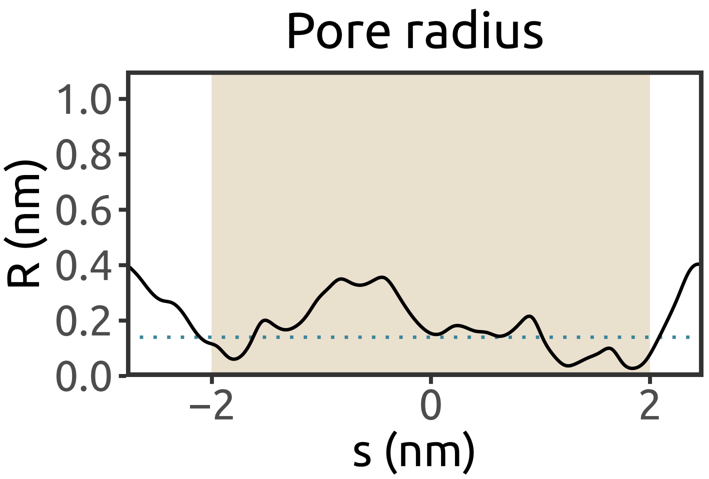


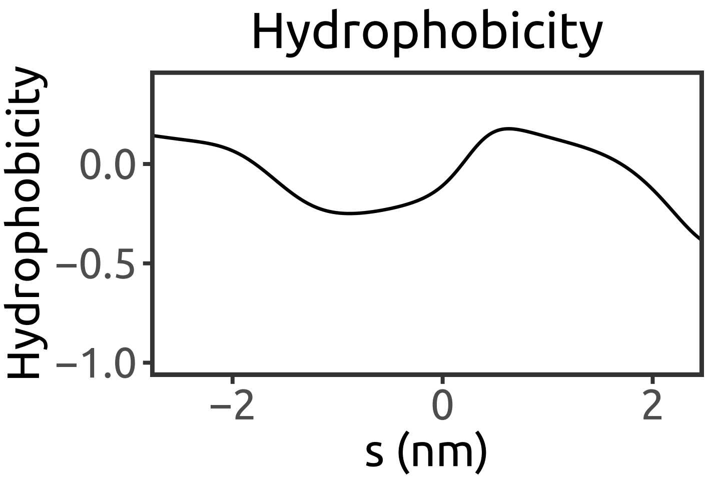


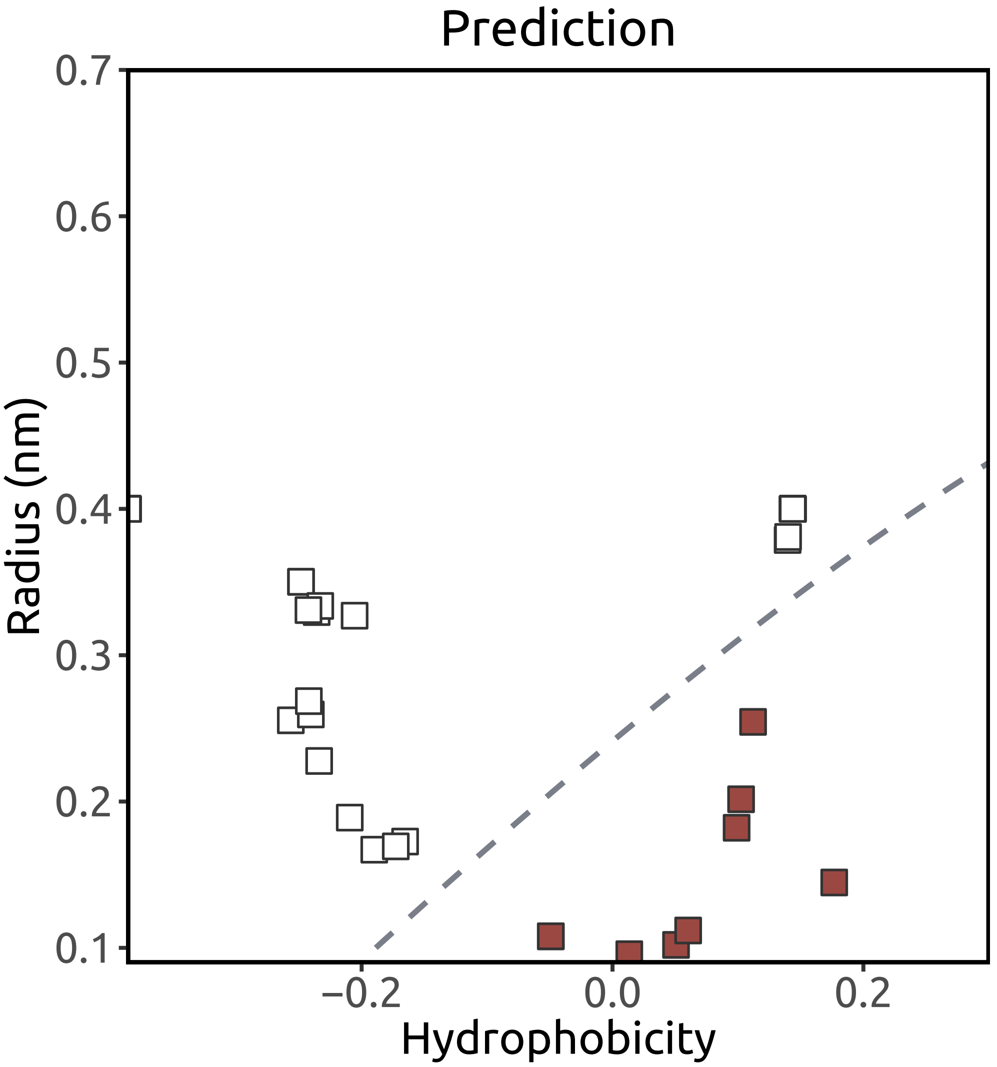


Heuristic score:
2.35 (n = 17)

Simulation result:
hydrated channel

# ASIC1 (PDB ID: 4NTY)

*Gallus gallus*
X−ray (2.65 Å)

Baconguis et al., 2014

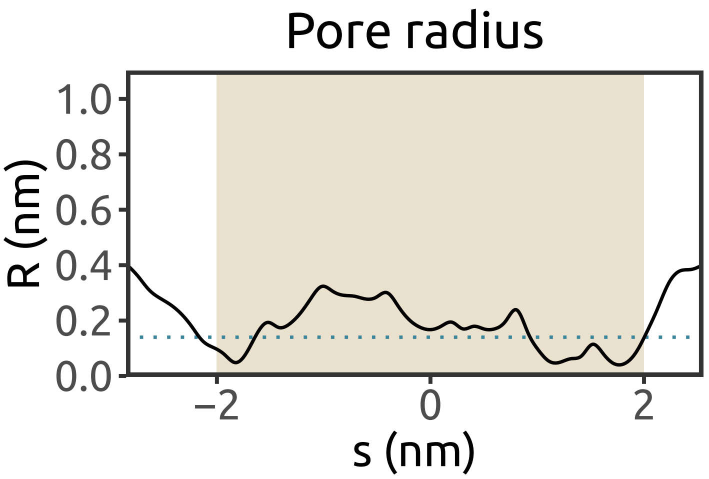


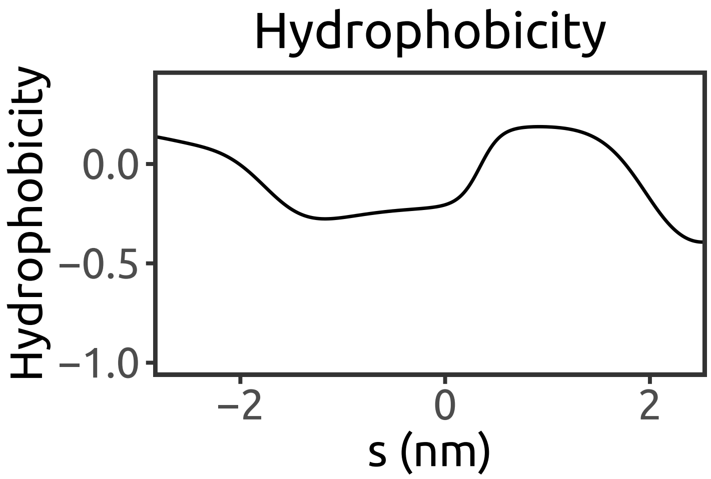


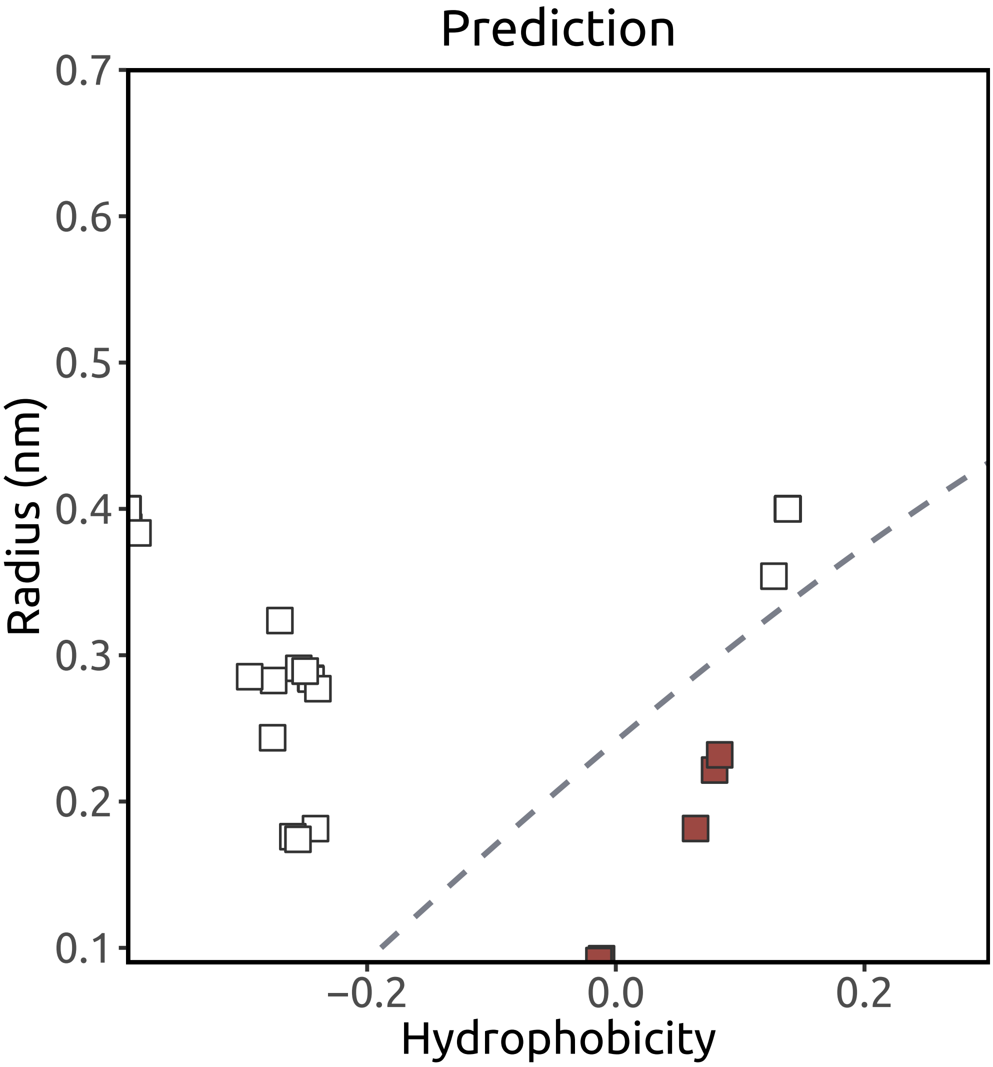


Heuristic score:
1.83 (n = 12)

Simulation result:
hydrated channel

# ASIC1 (PDB ID: 4NYK)

*Gallus gallus*
X–ray (3 Å)

Gonzales et al., 2009

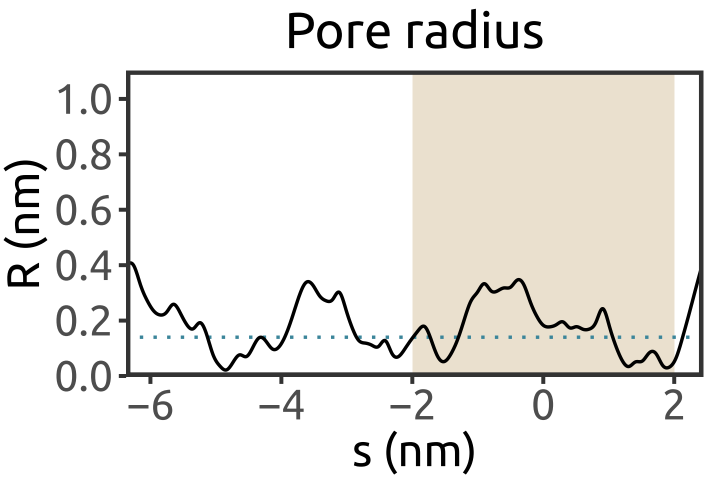


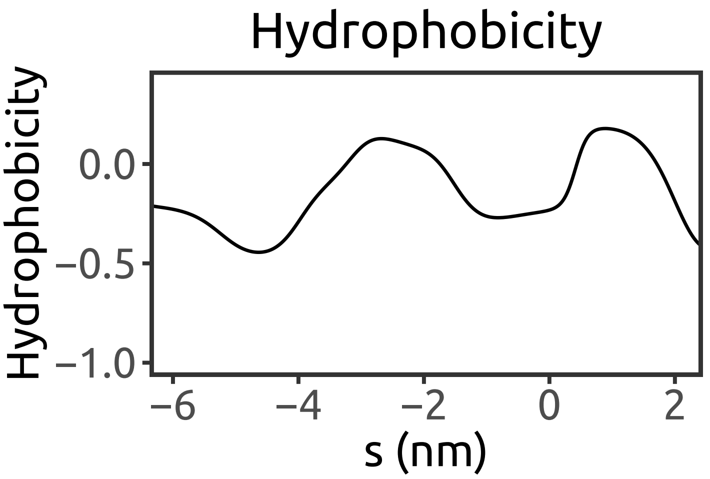


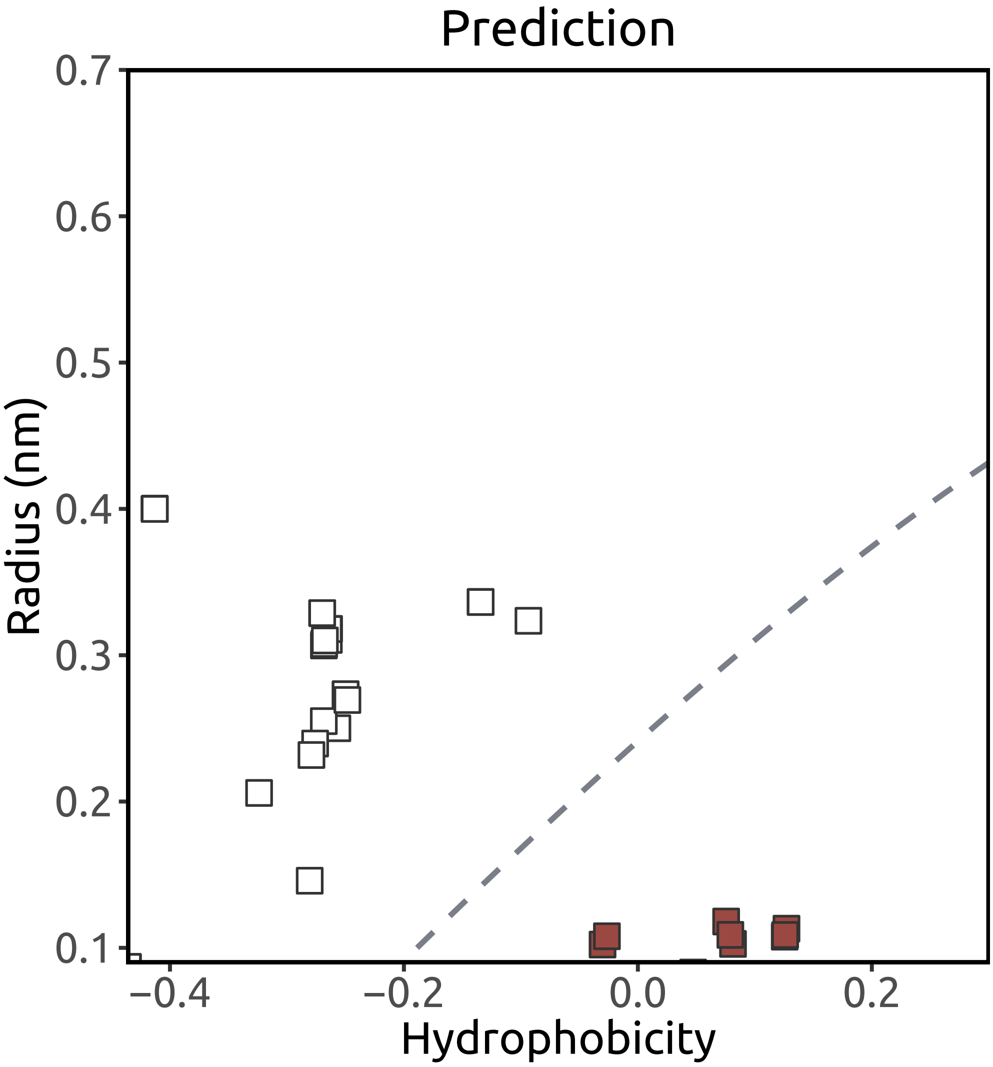


Heuristic score:
2.43 (n = 15)

Simulation result:
barrier to water

# ASIC1 (PDB ID: 5WKU)

*Gallus gallus*

X−ray (2.95 Å)

Yoder et al., 2018

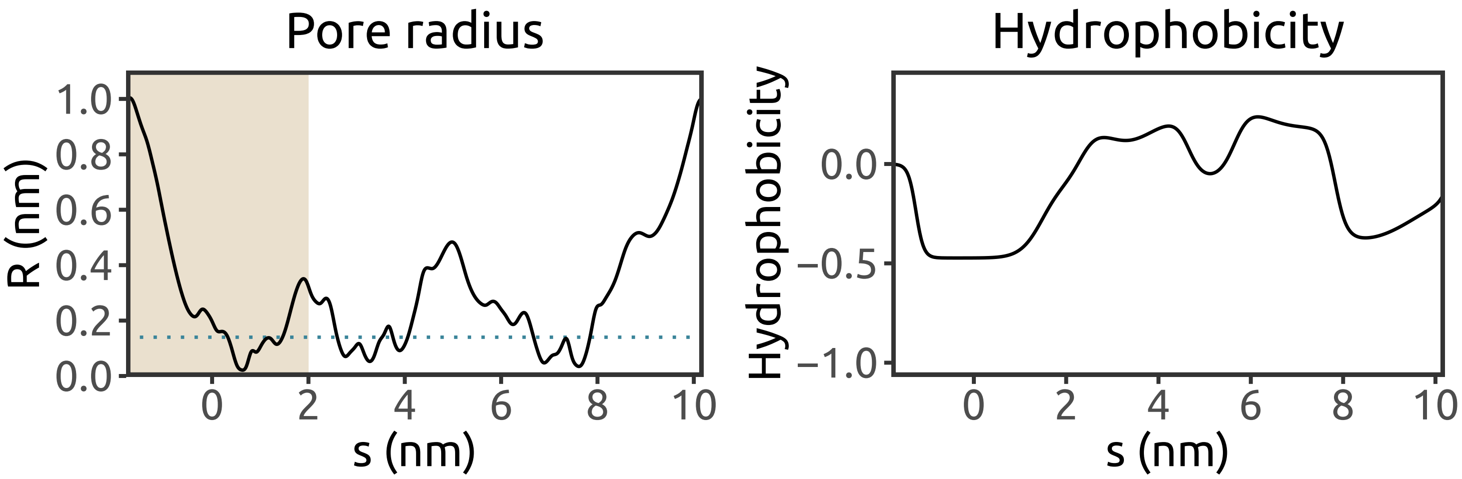


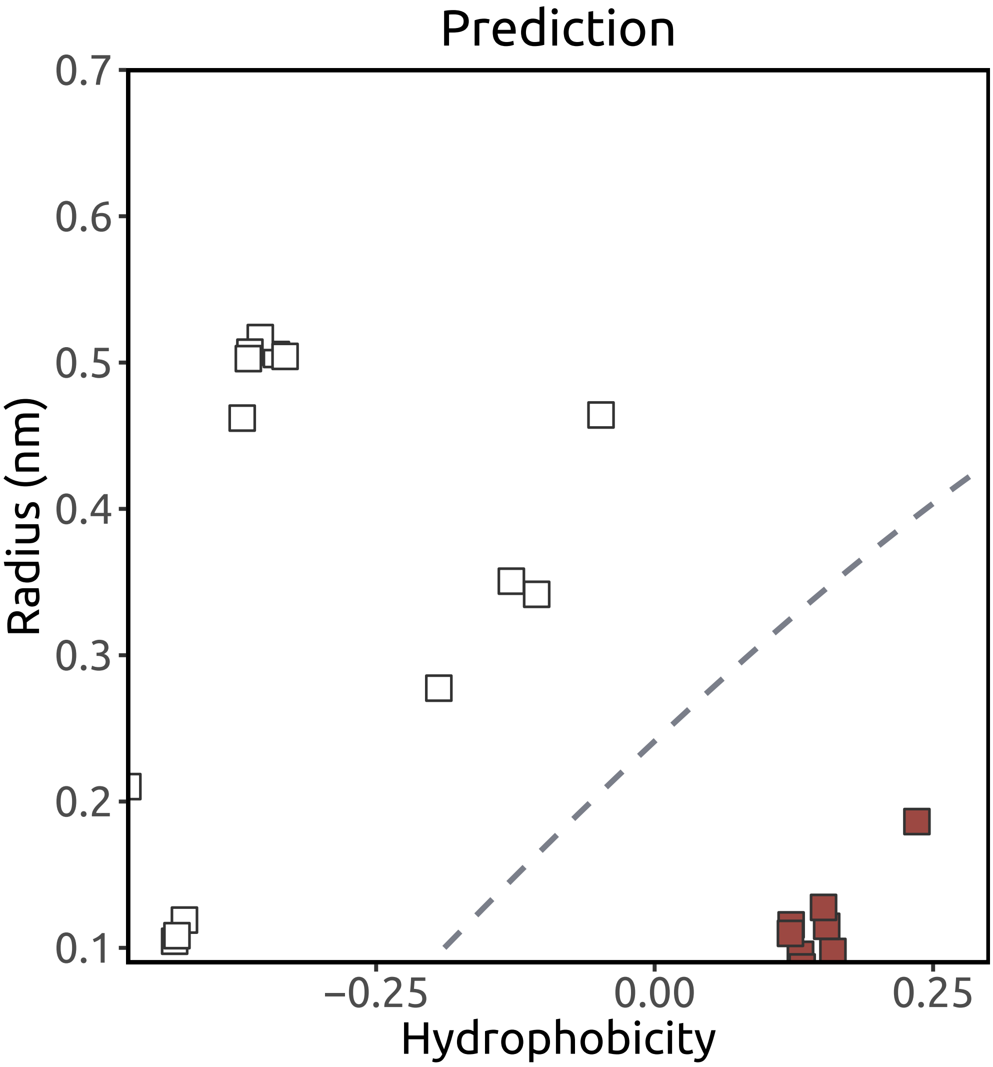


Heuristic score:

2.67 (n = 13)

Simulation result:

barrier to water

# ExbBExbD (PDB ID: 5SV0)

*Escherichia coli*
X−ray (2.6 Å)

Celia et al., 2016

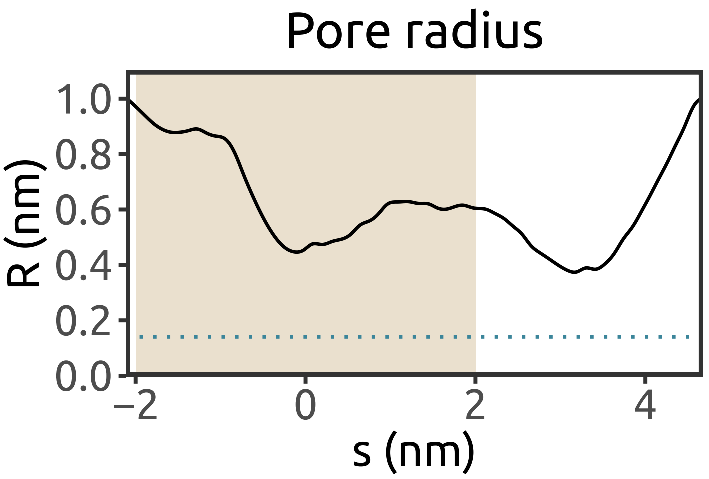


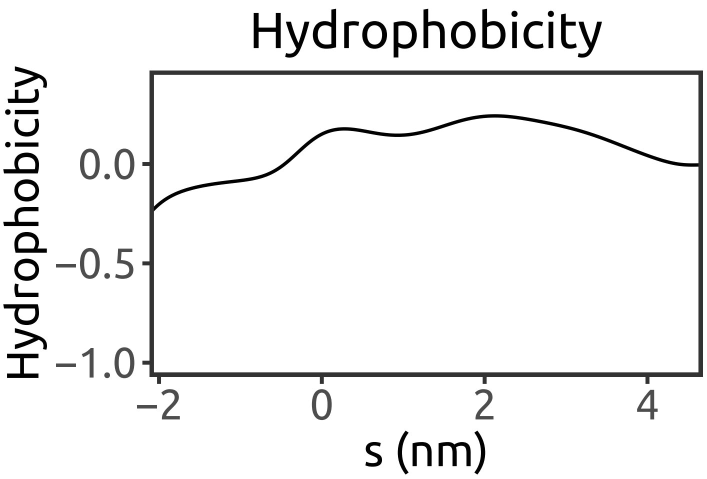


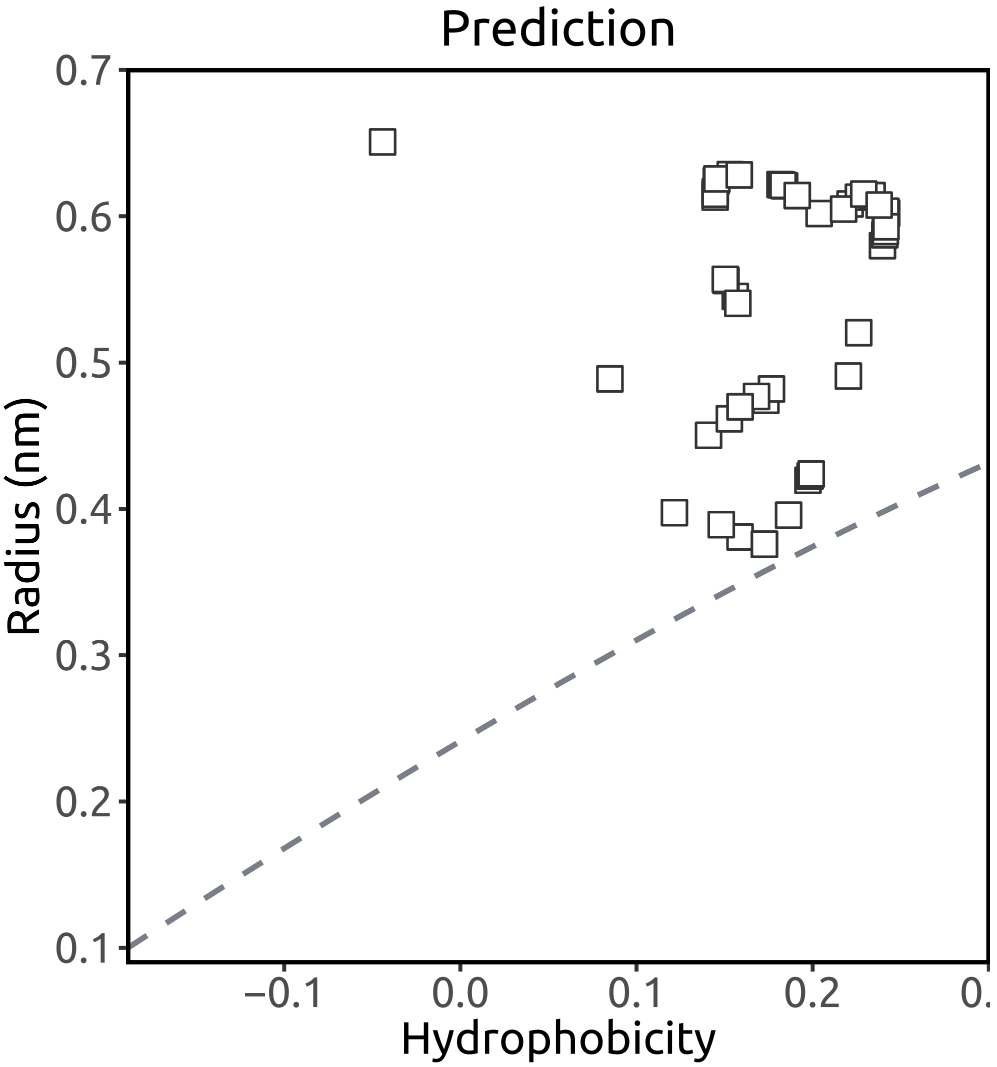


Heuristic score:
0 (n = 0)

Simulation result:
hydrated channel

# ExbBExbD (PDB ID: 5SV1)

*Escherichia coli*

X−ray (3.5 Å)

Celia et al., 2016

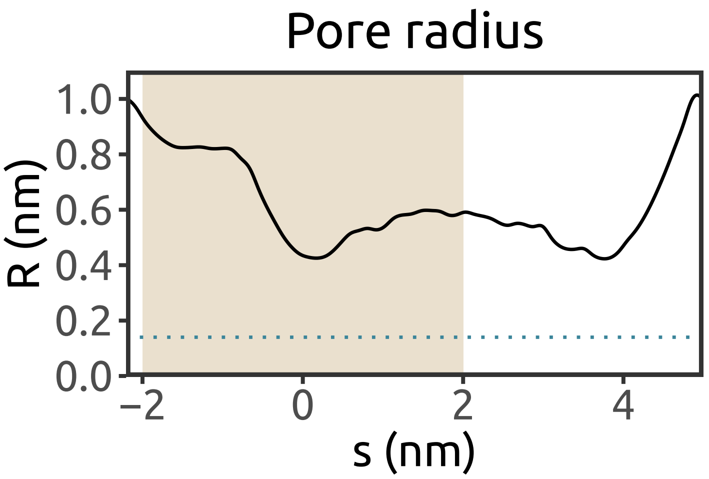


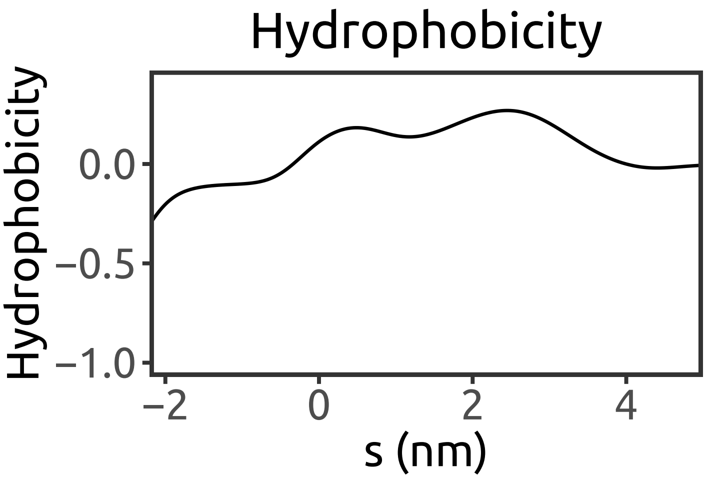


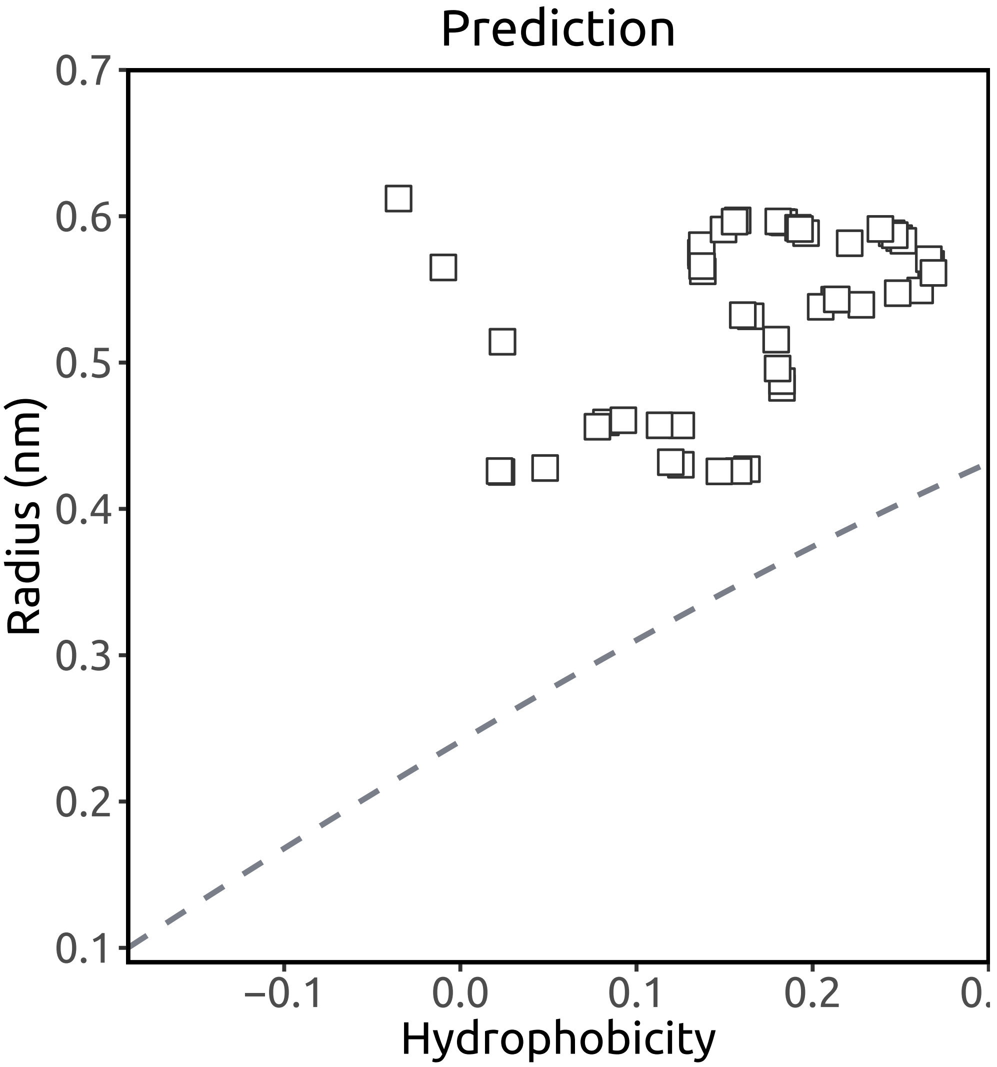


Heuristic score:

0 (n = 0)

Simulation result:

hydrated channel

# M2 (PDB ID: 2KIX)

*Influenza B*

aq NMR

Wang et al., 2009

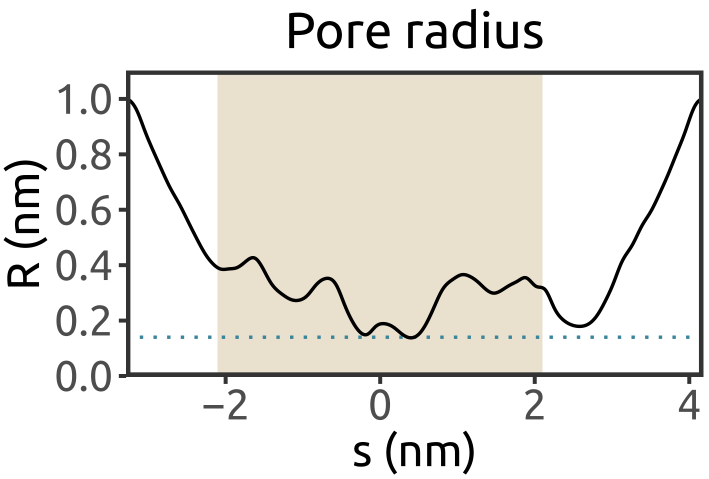


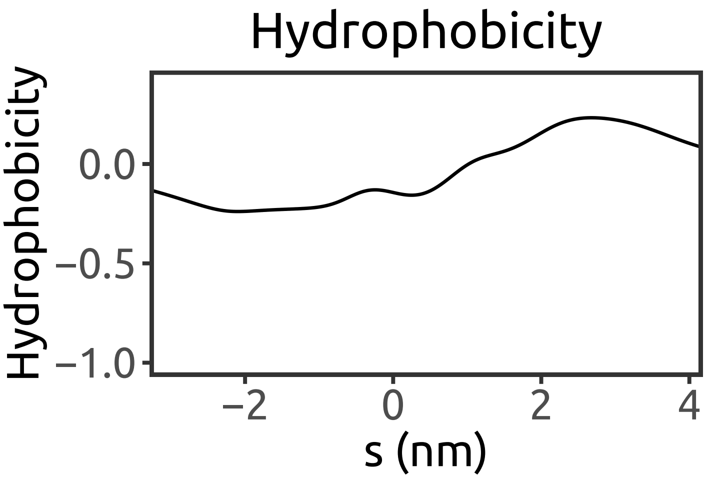


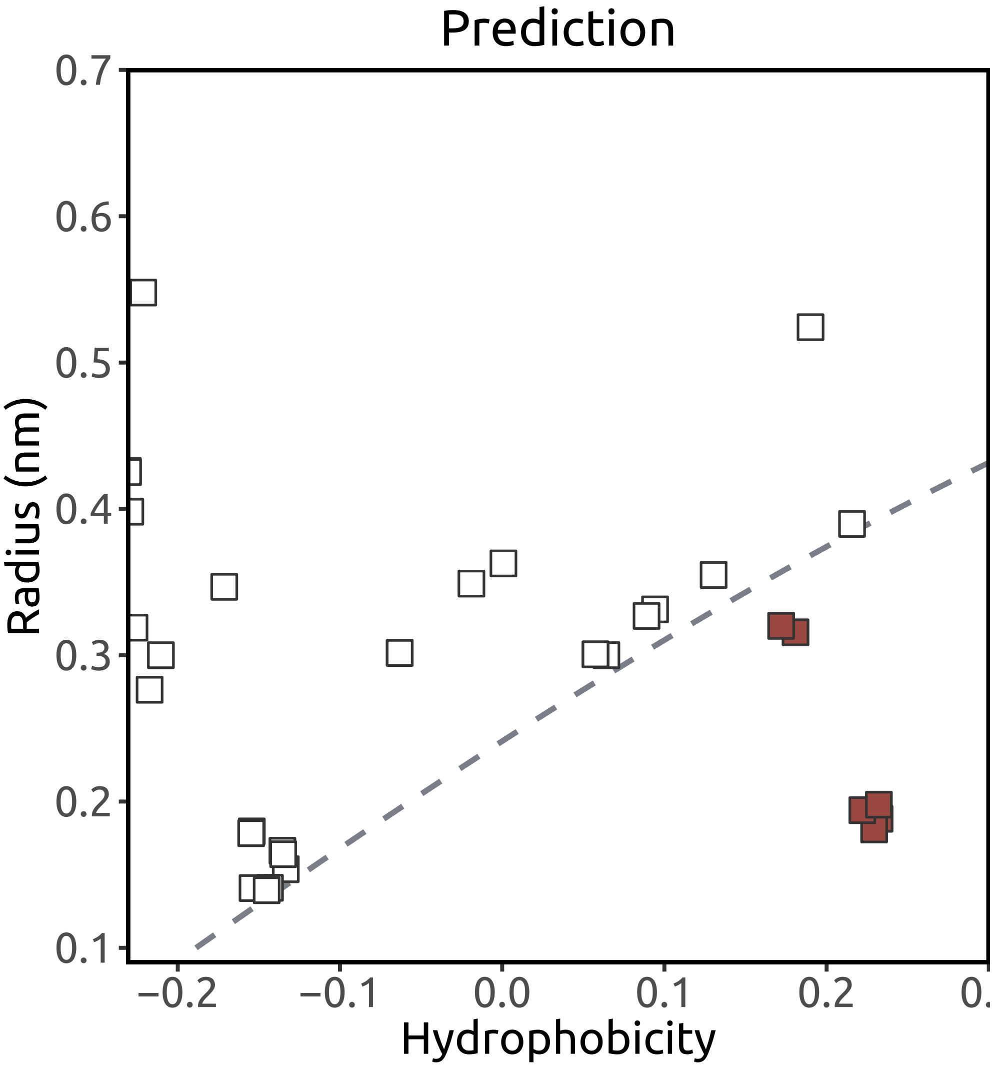


Heuristic score:

0.76 (n = 6)

Simulation result:

barrier to water

# M2 (PDB ID: 3BKD)

*Influenza A*
X−ray (2.05 Å)

Stouffer et al., 2008

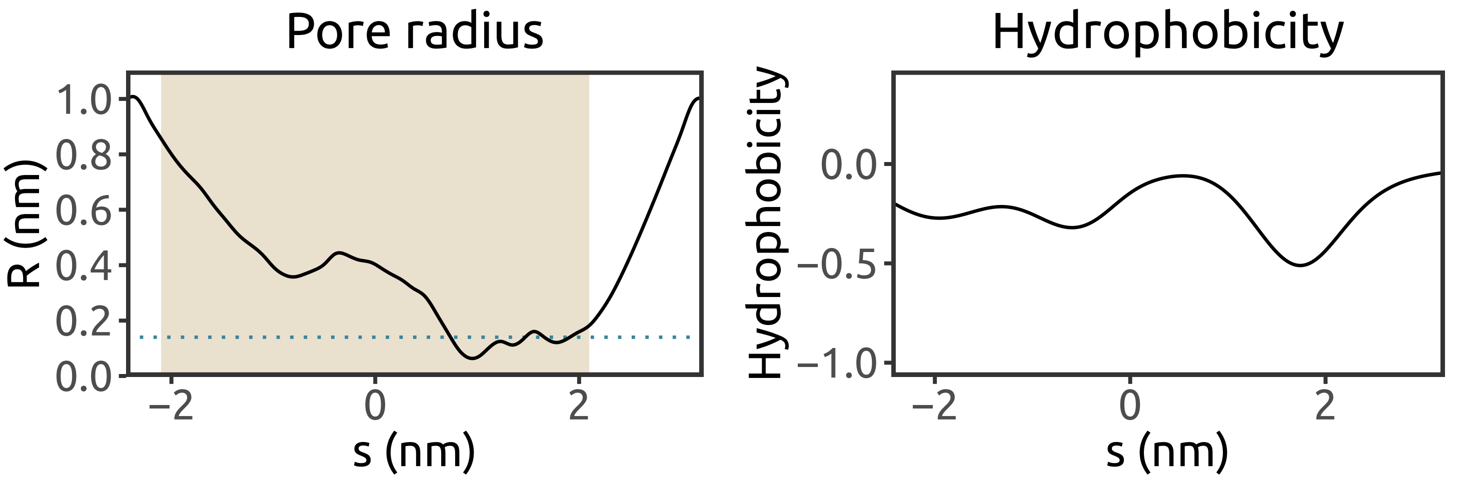


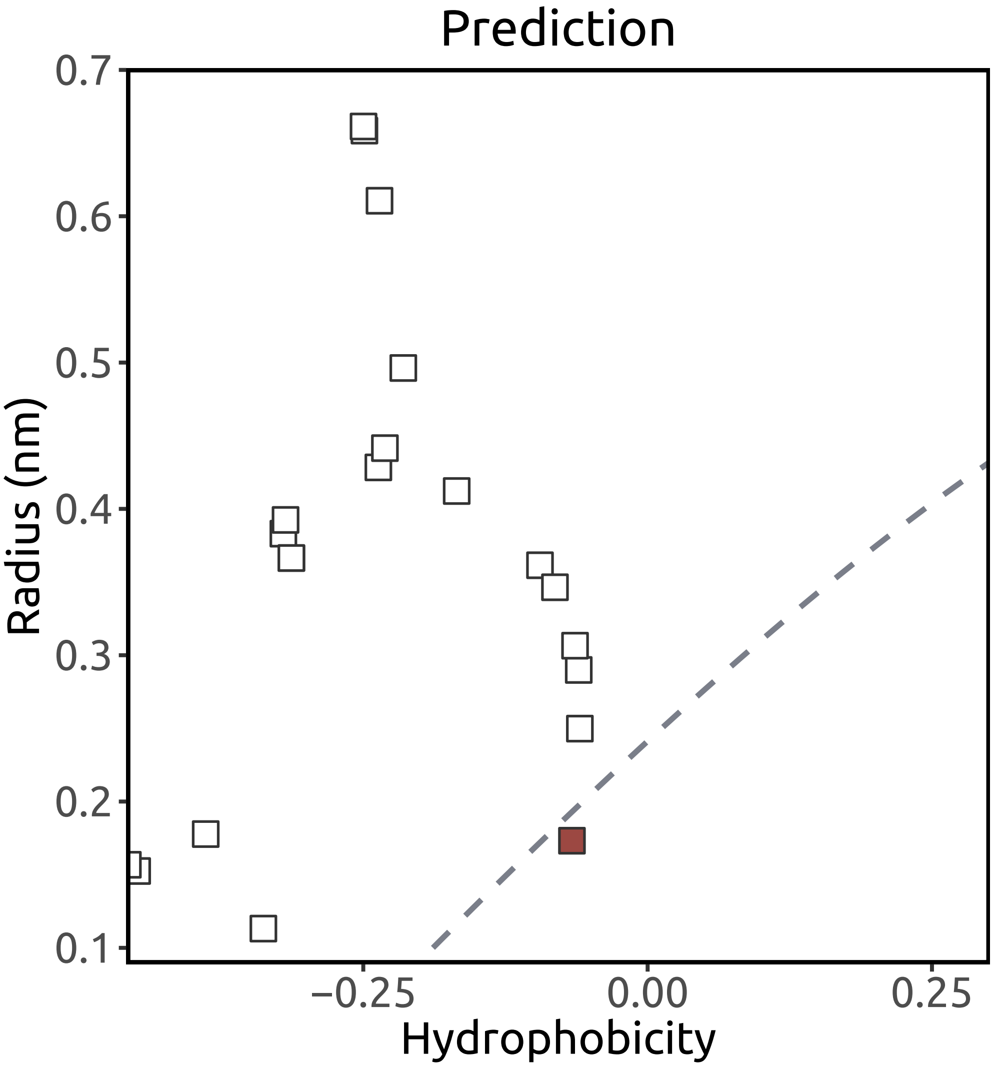


Heuristic score:
0.27 (n = 5)

Simulation result:
barrier to water

# M2 (PDB ID: 4QKC)

*Influenza A*
X–ray (1.1 Å)

Thomaston et al., 2015

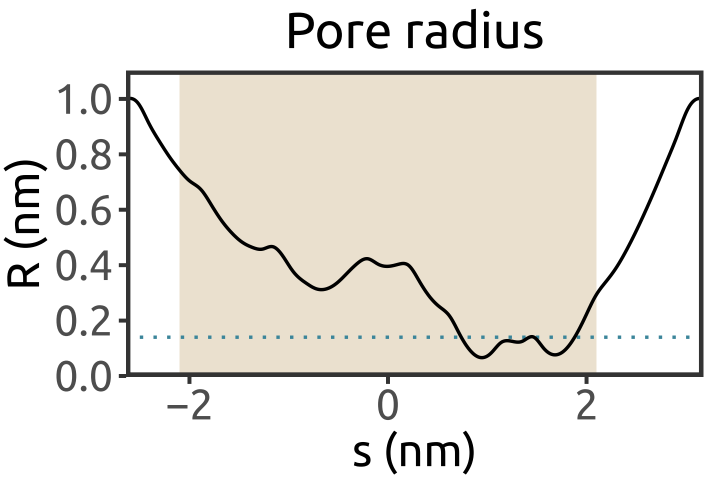


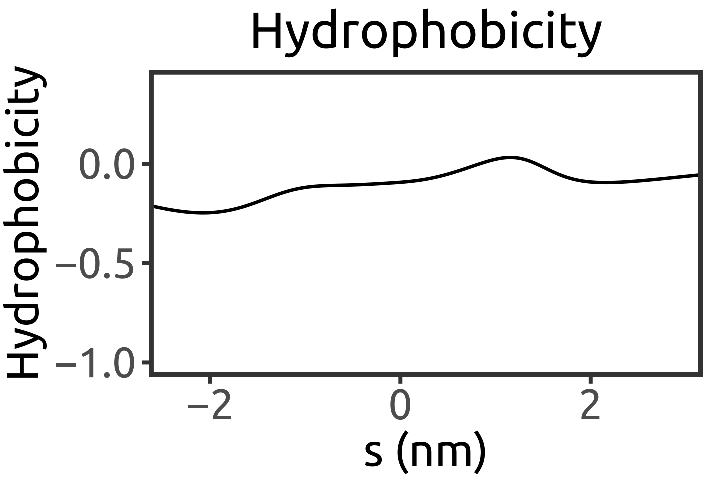


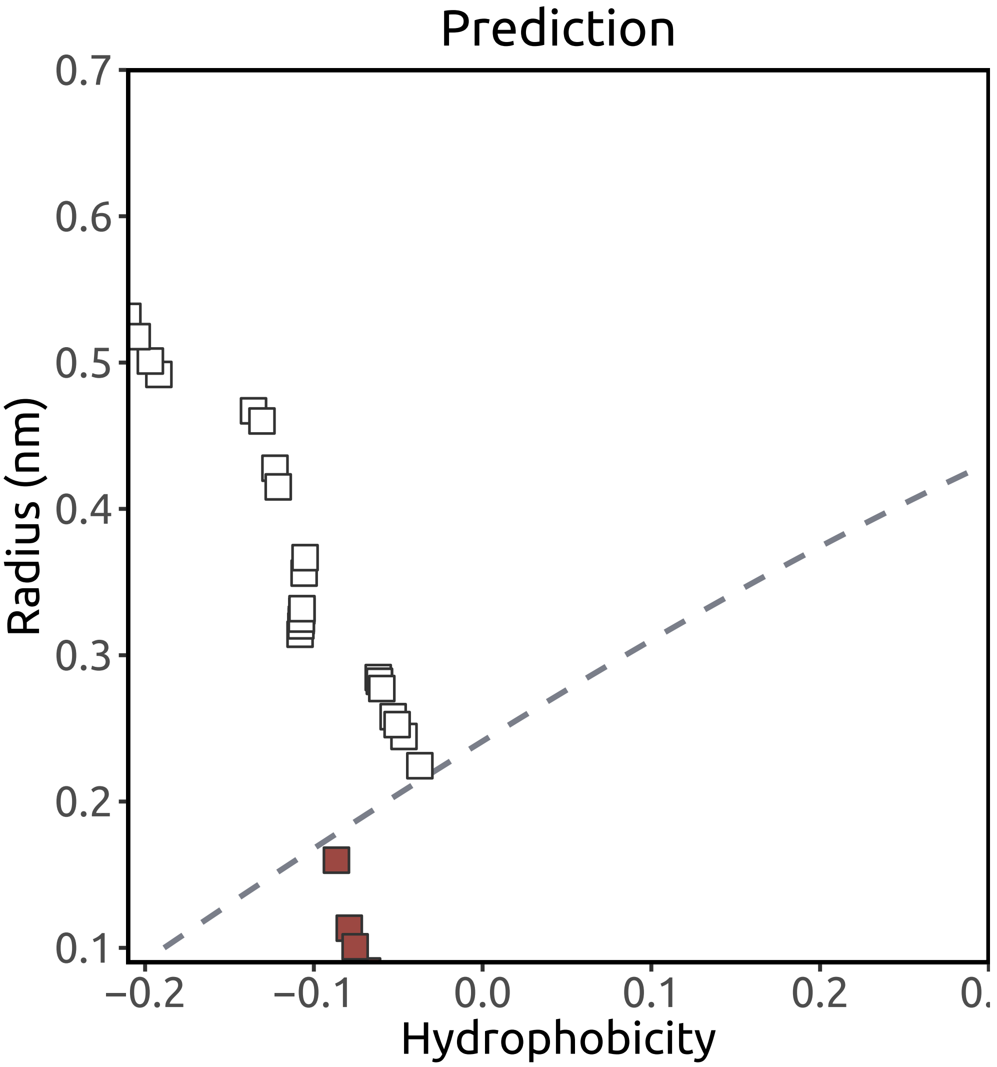


Heuristic score:
0.82 (n = 8)

Simulation result:
barrier to water

# M2 (PDB ID: 5JOO)

*Influenza A*
X–ray (1.41 Å)

Thomaston et al., 2017

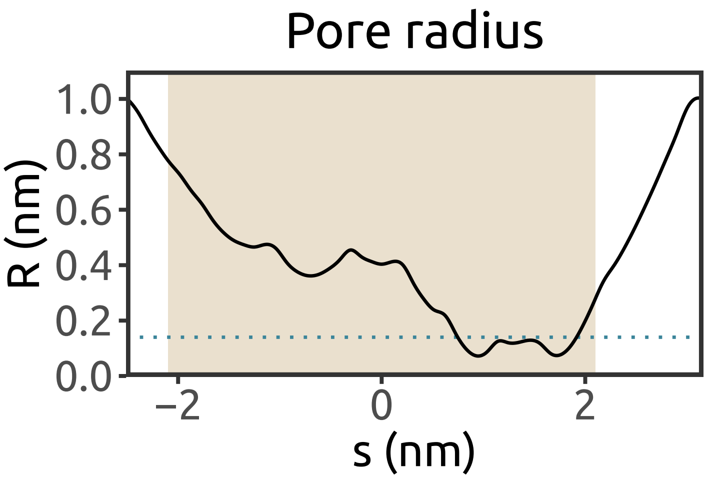


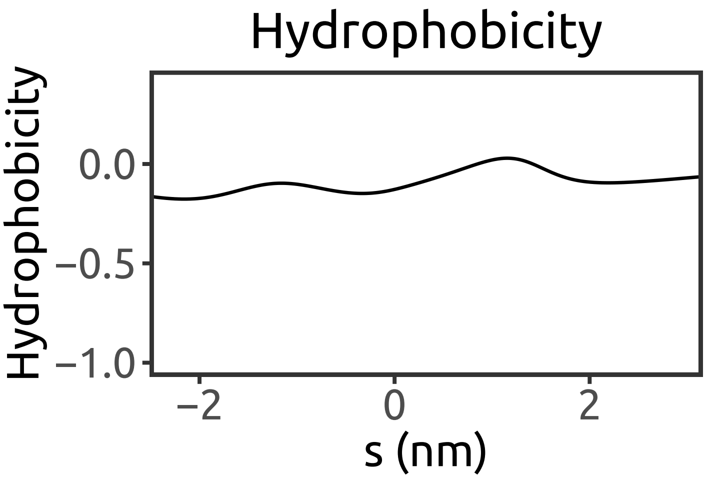


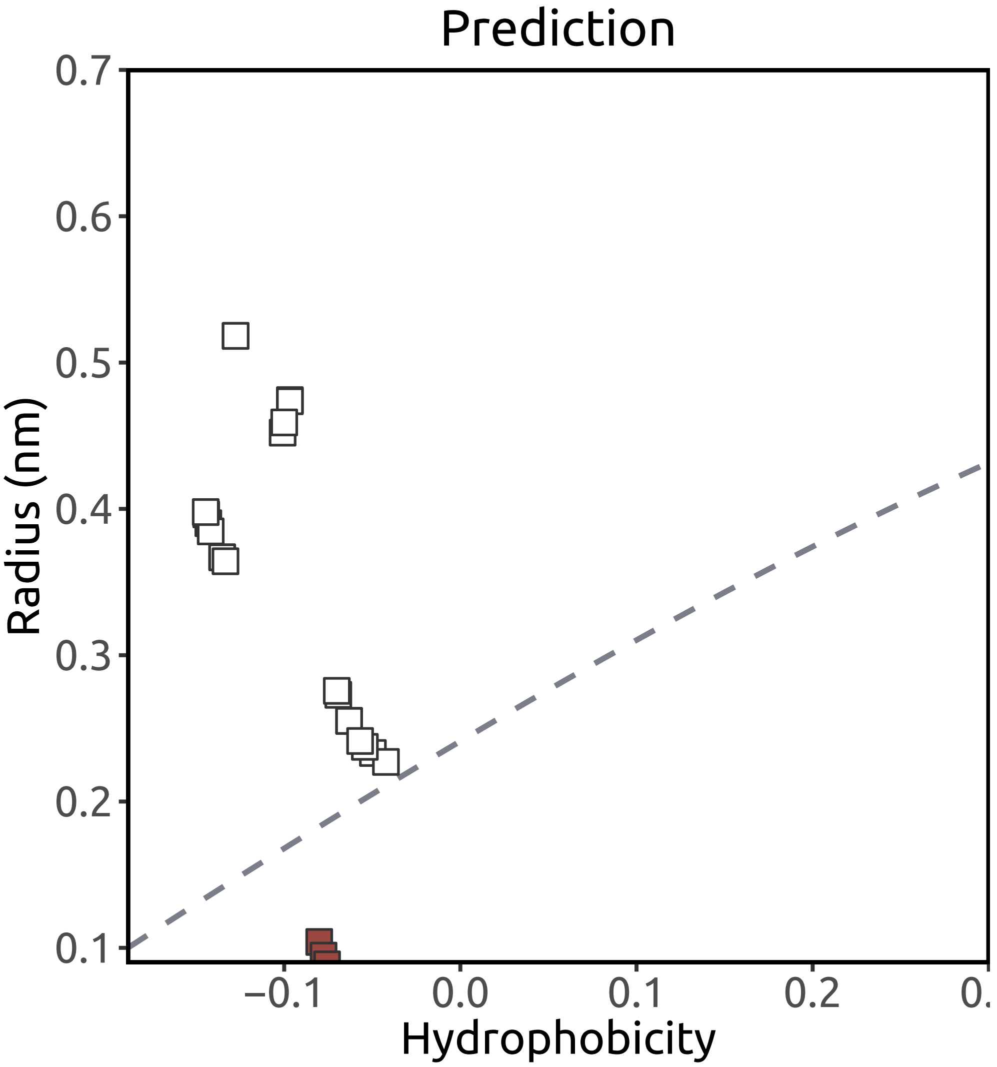


Heuristic score:
0.88 (n = 8)

Simulation result:
barrier to water

# M2 (PDB ID: 5TTC)

*Influenza A*
X–ray (1.4 Å)

Thomaston et al., 2017

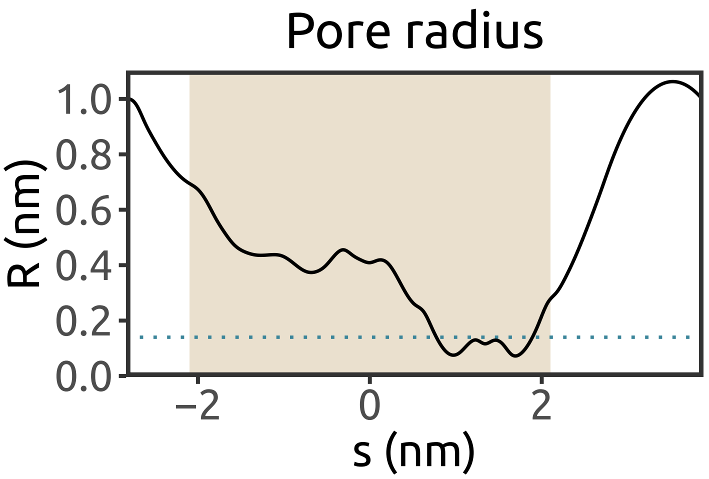


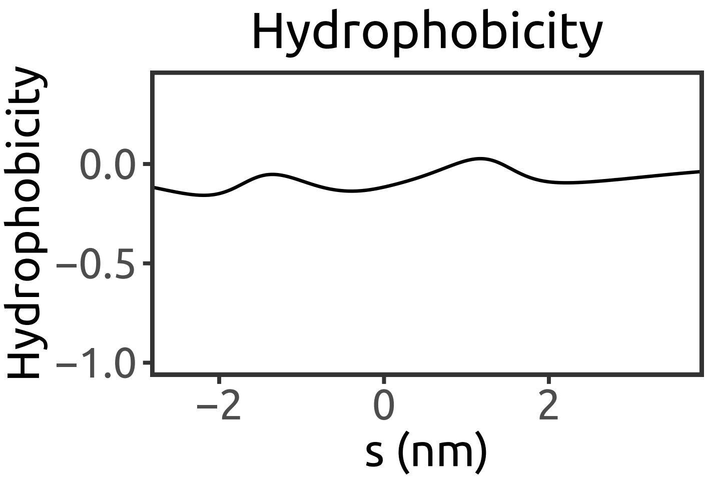


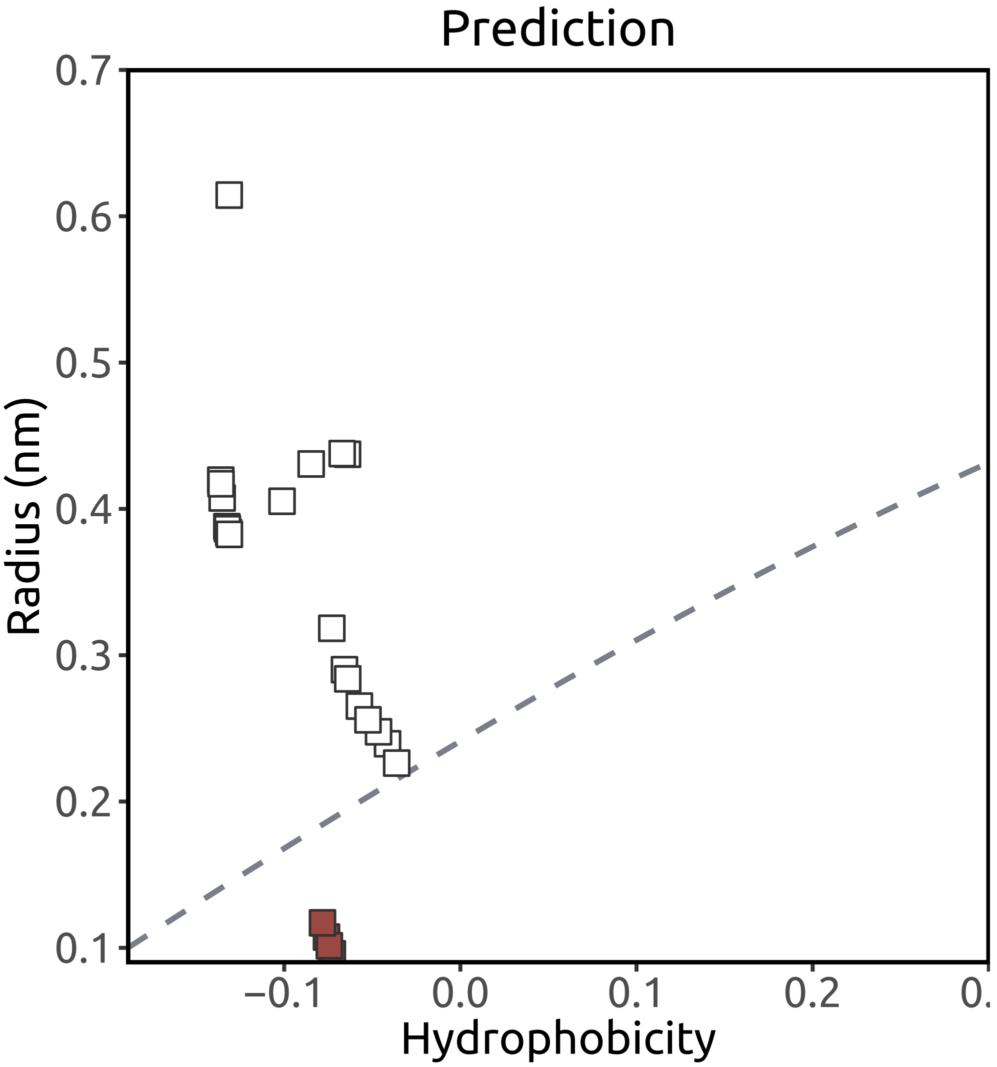


Heuristic score:
0.83 (n = 8)

Simulation result:
barrier to water

# M2 (PDB ID: 5UM1)

*Influenza A*
X–ray (1.45 Å)

Thomaston et al., 2017

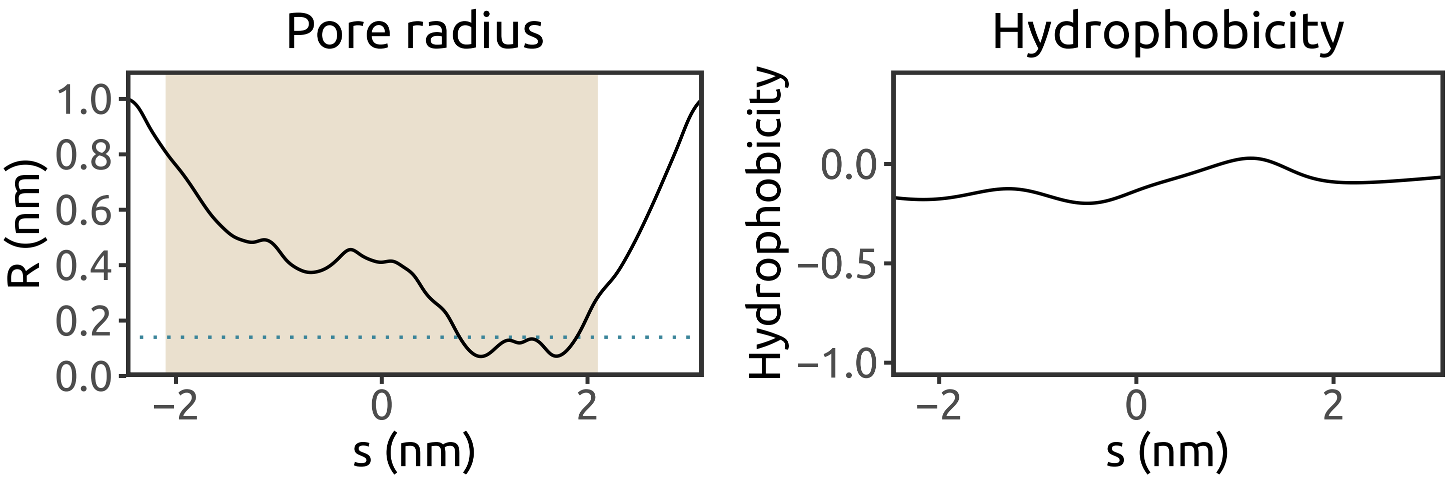


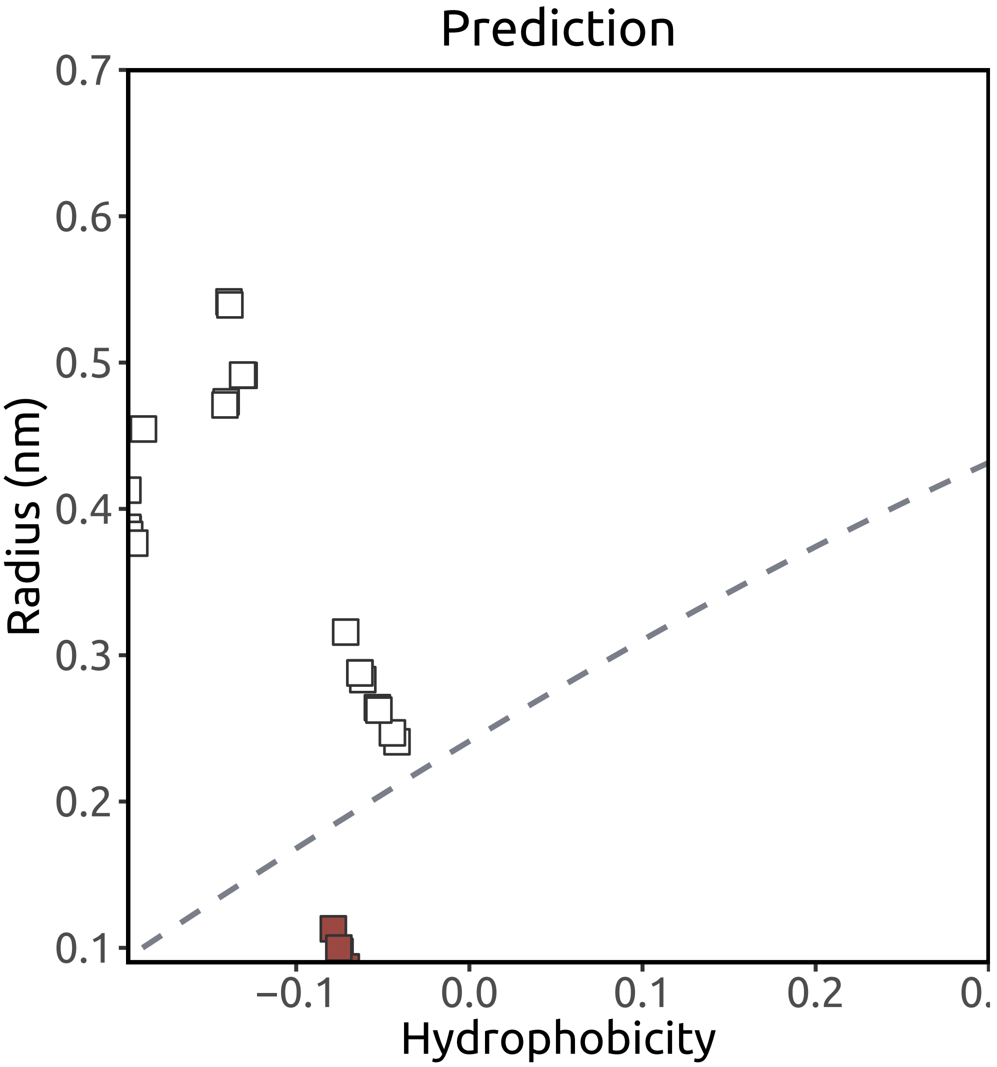


Heuristic score:
0.87 (n = 8)

Simulation result:
barrier to water

# MCU (PDB ID: 5ID3)

*Caenorhabditis elegans*

aq NMR

Oxenoid et al., 2016

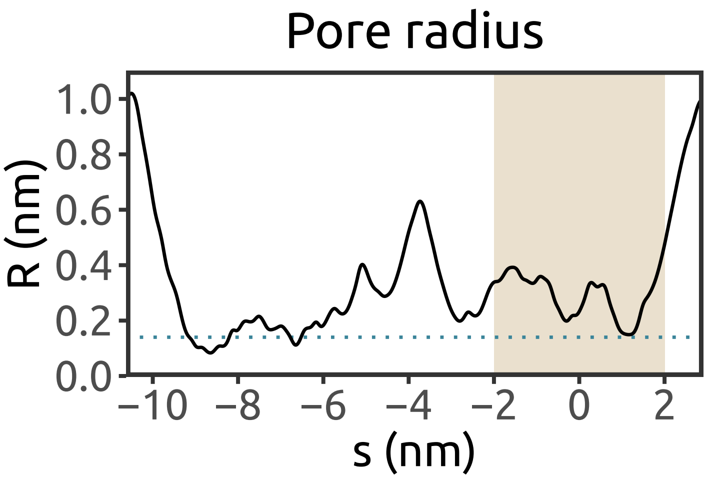


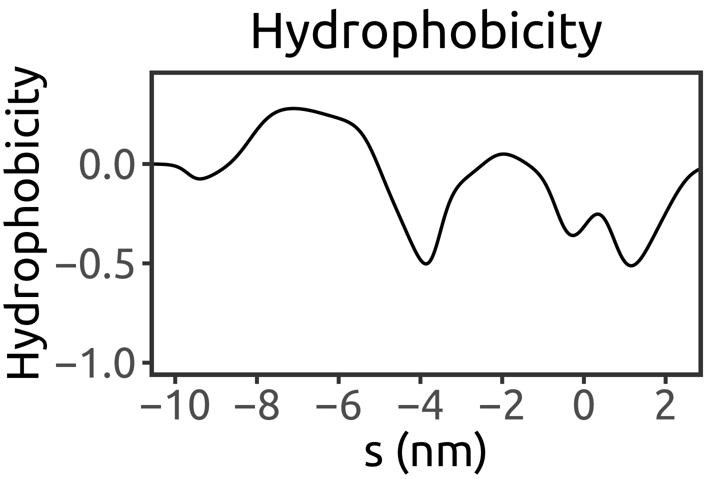


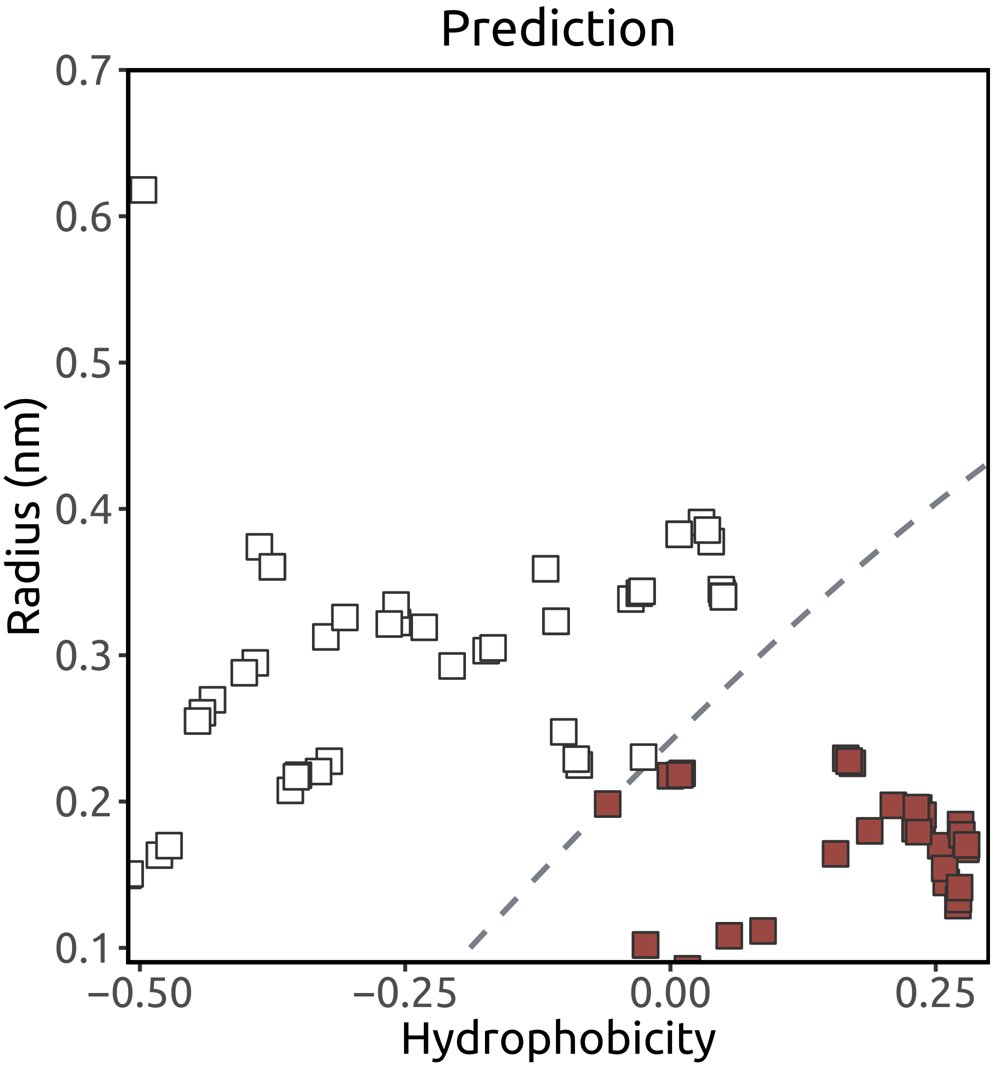


Heuristic score:

5.2 (n = 35)

Simulation result:

barrier to water

# MgtE (PDB ID: 2ZY9)

*Thermus thermophilus*
X–ray (2.94 Å)

Hattori et al., 2009

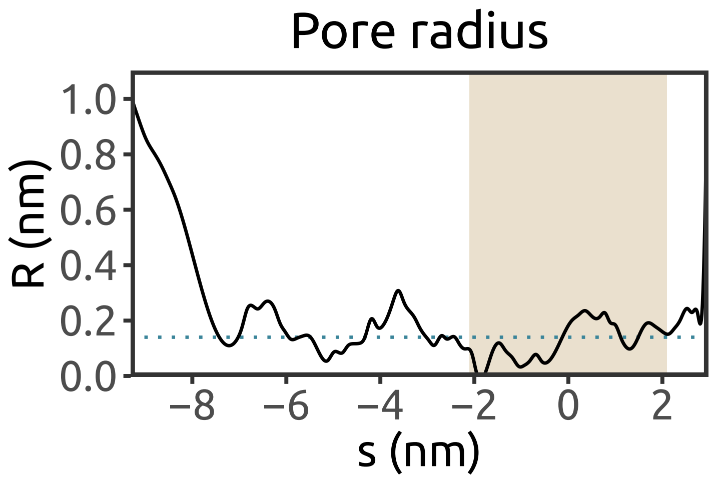


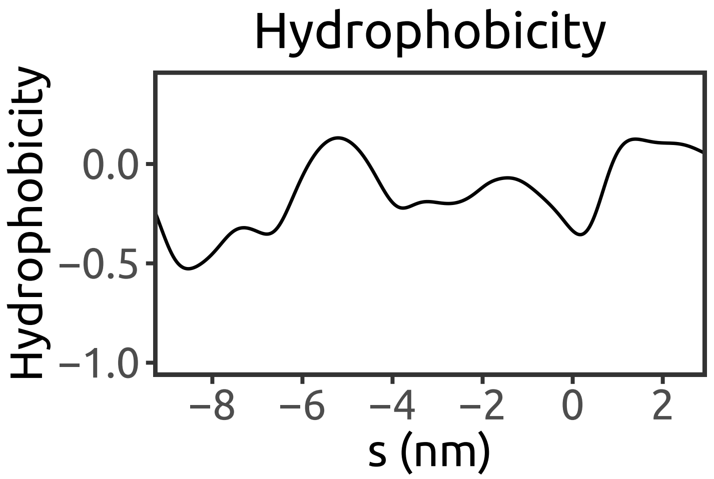


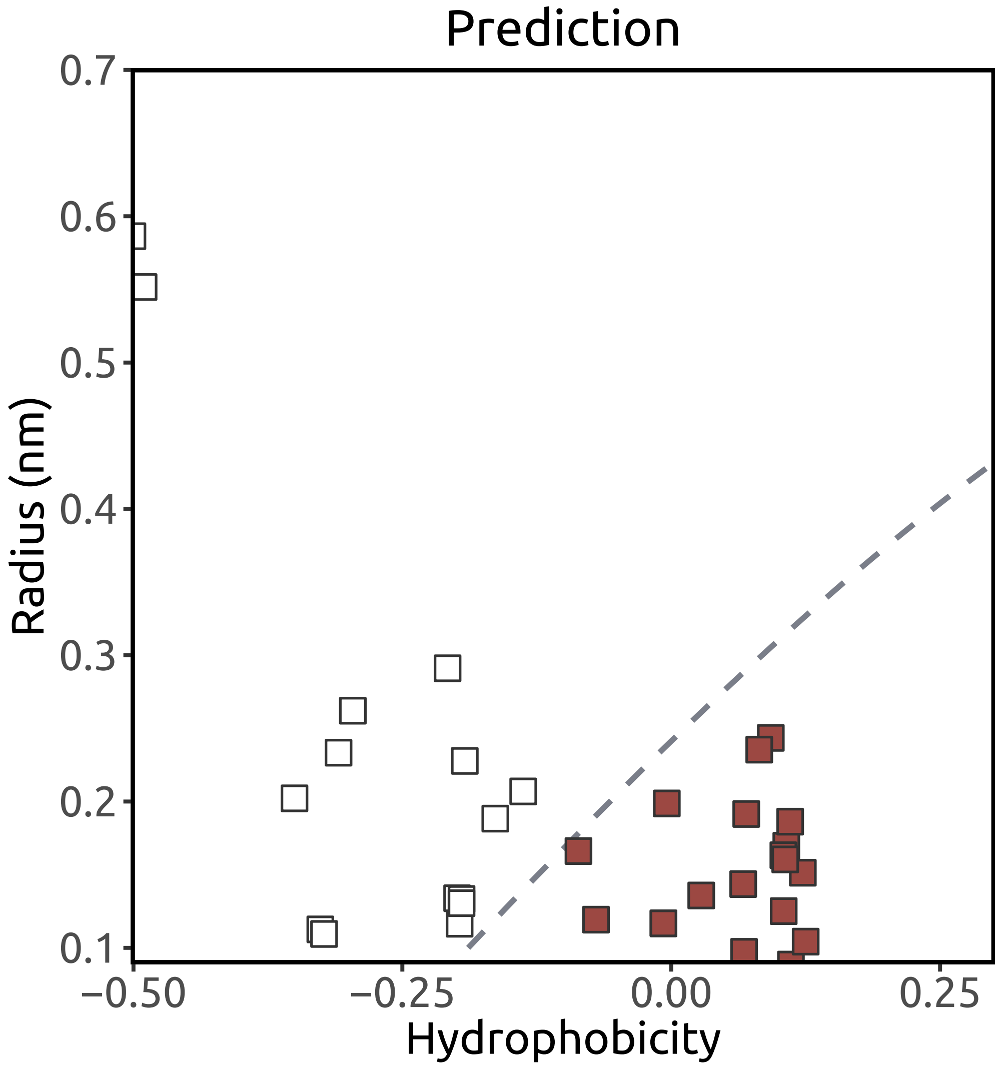


Heuristic score:
2.58 (n = 23)

Simulation result:
barrier to water

# MgtE (PDB ID: 4U9N)

*Thermus thermophilus*
X−ray (2.2 Å)

Takeda et al., 2014

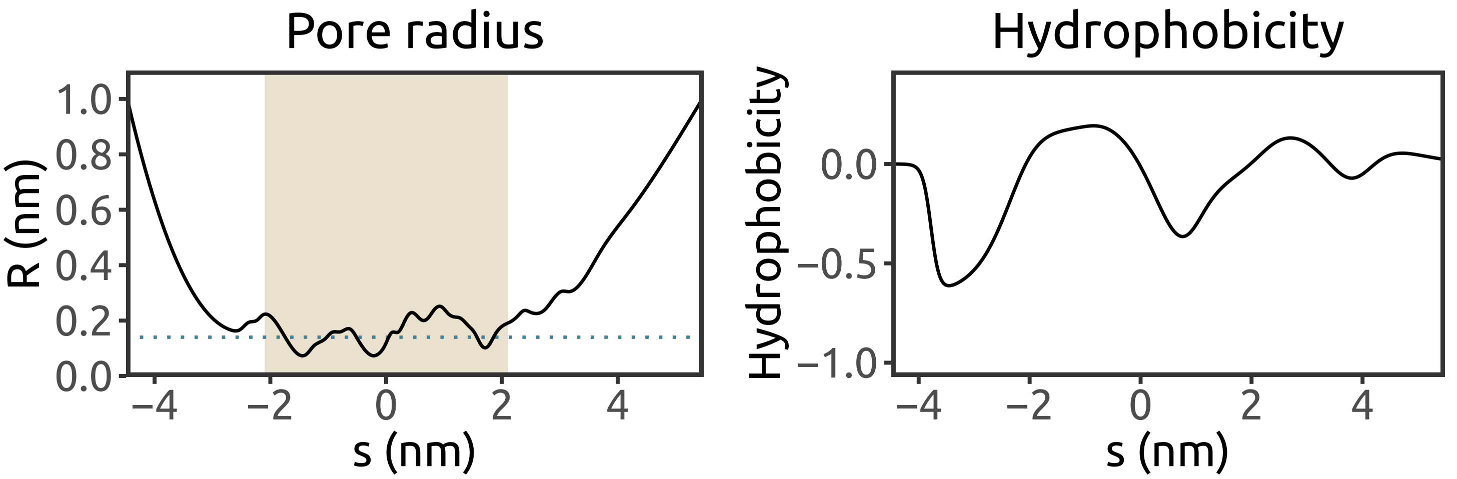


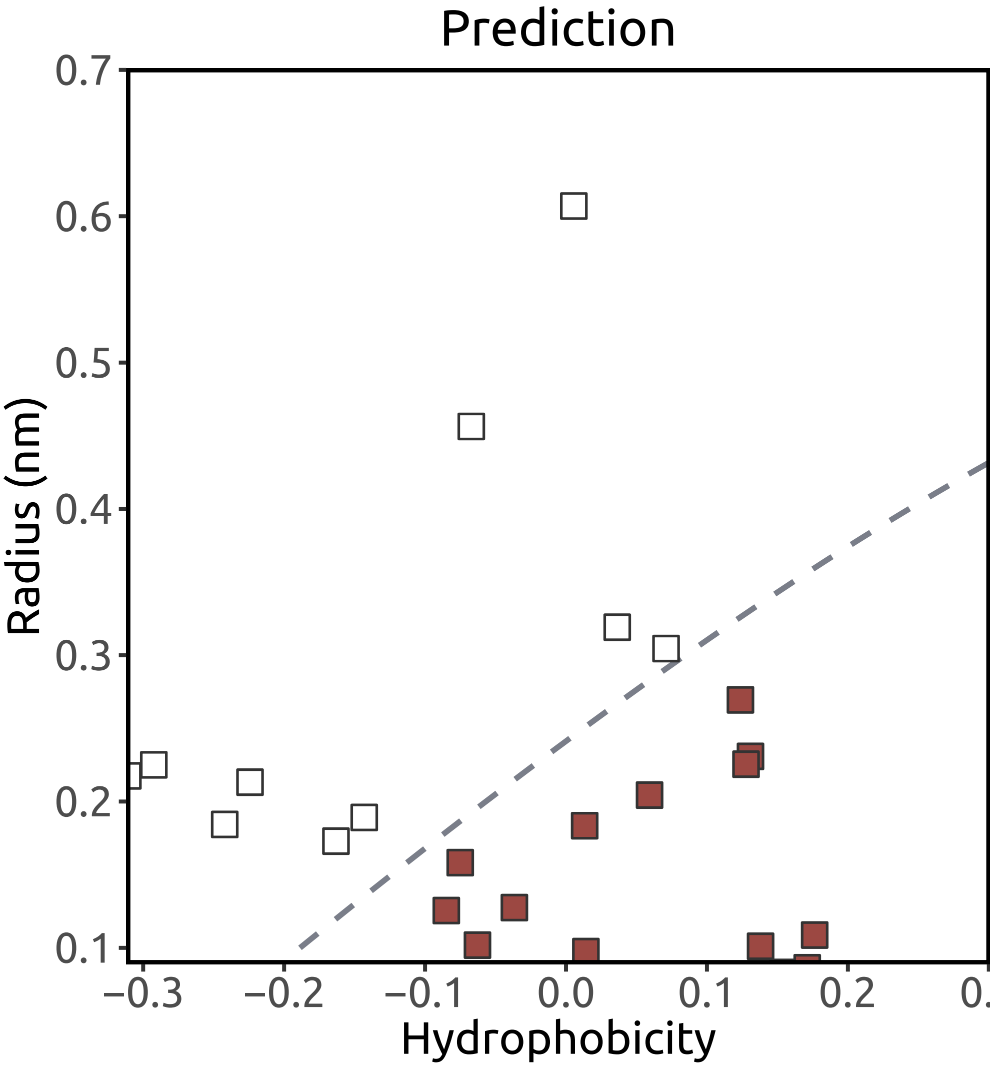


Heuristic score:
1.94 (n = 16)

Simulation result:
barrier to water

# MscL (PDB ID: 2OAR)

*Mycobacterium tuberculosis*
X−ray (3.5 Å)

Chang et al., 1998

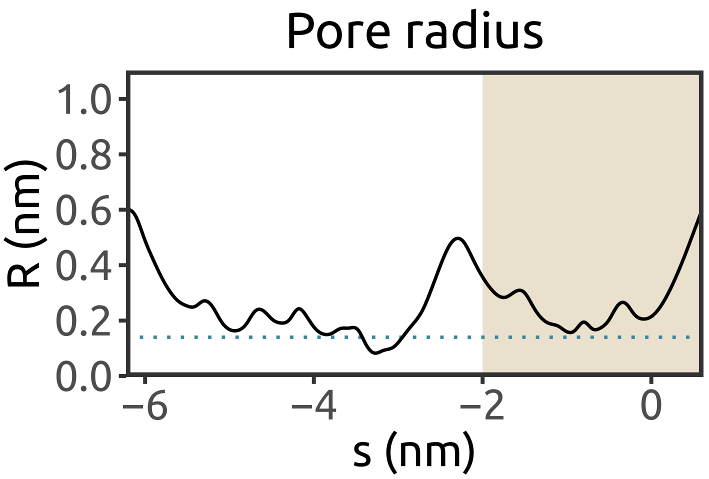


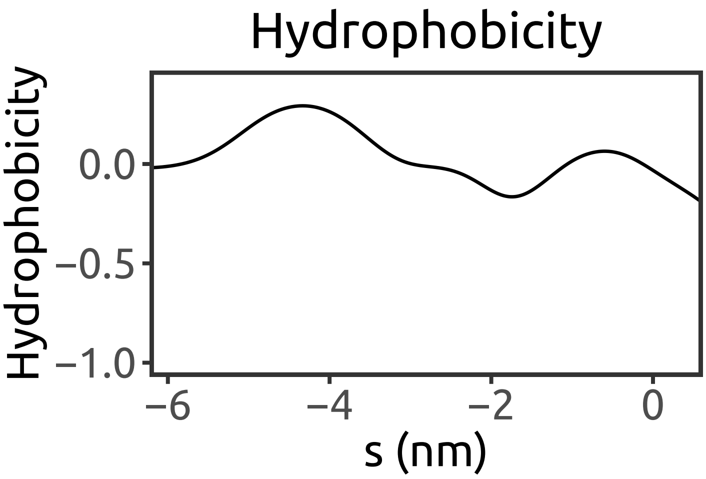


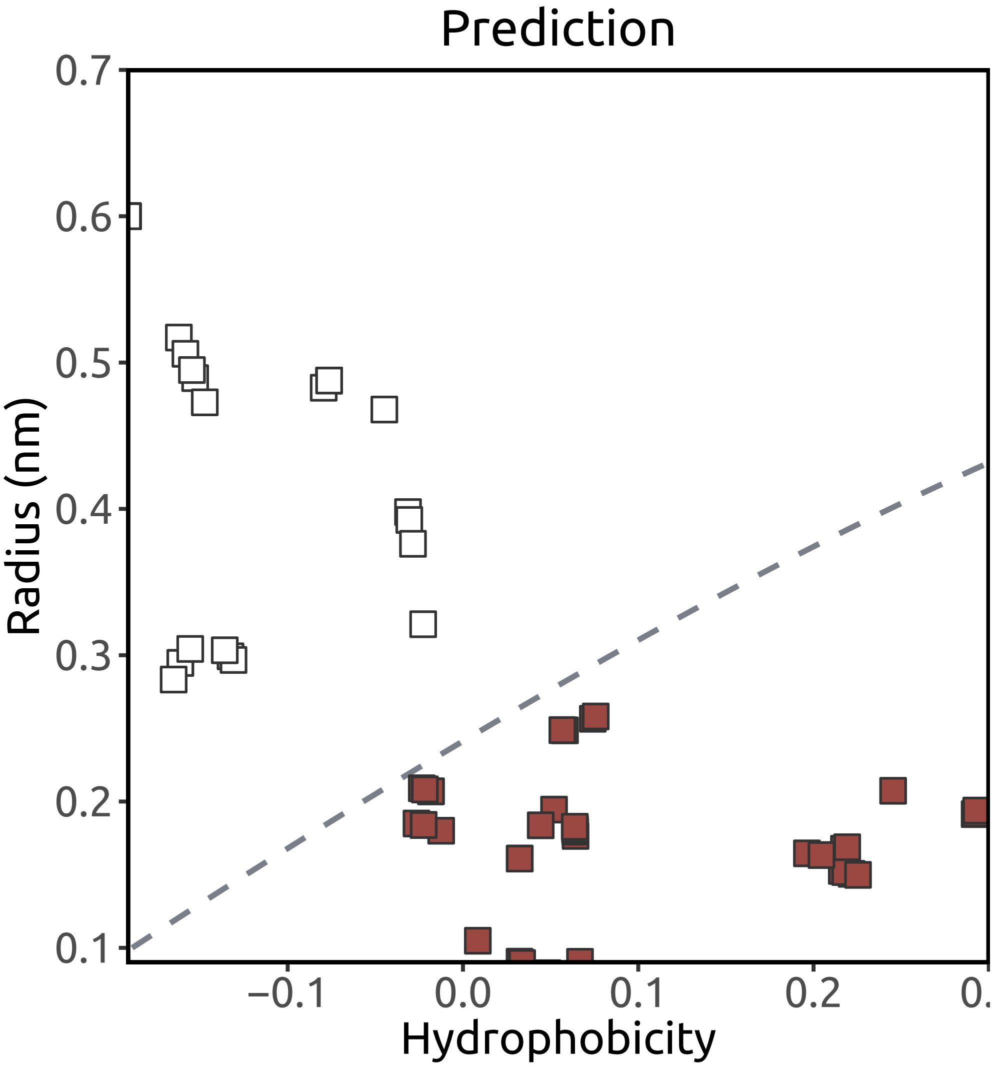


Heuristic score:
4.73 (n = 44)

Simulation result:
barrier to water

# MscL (PDB ID: 3HZQ)

*Staphylococcus aureus*

X−ray (3.82 Å)

Liu et al., 2009

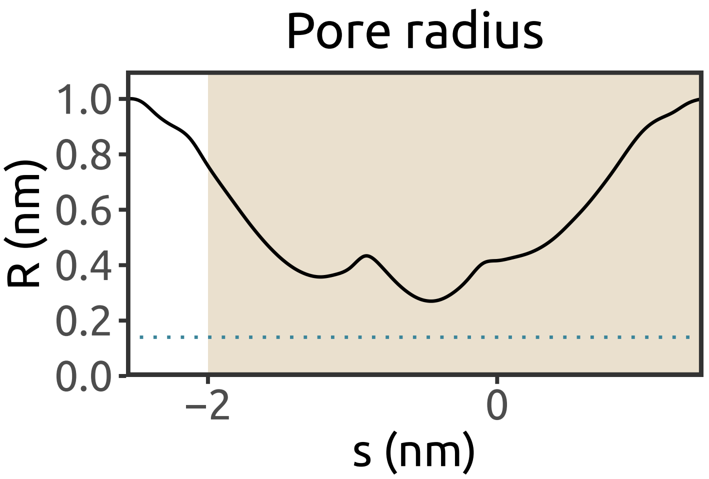


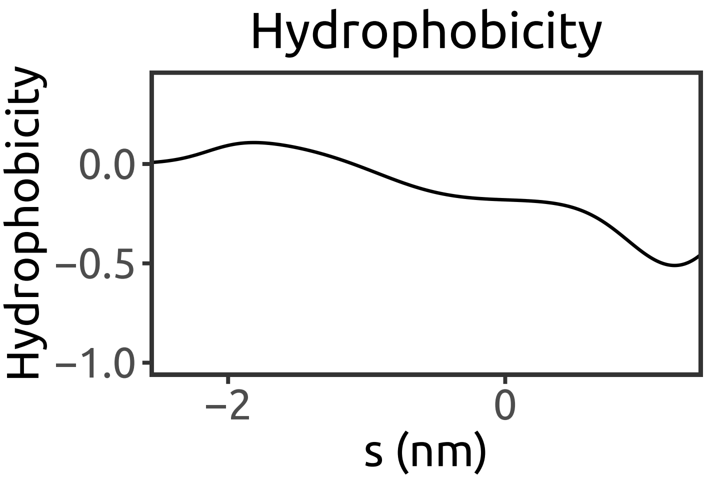


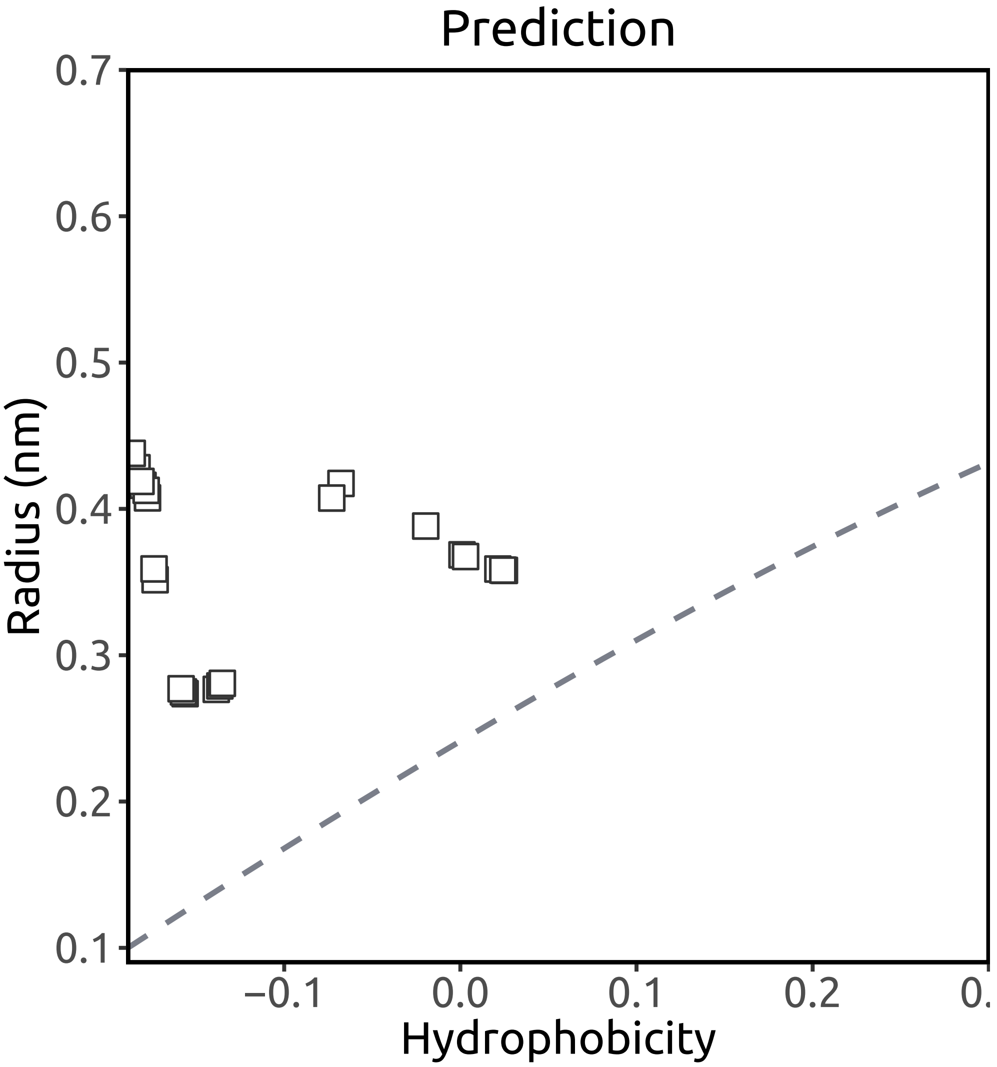


Heuristic score:

0 (n = 0)

Simulation result:

hydrated channel

# MscL (PDB ID: 4Y7J)

*Methanosarcina acetivorans*
X−ray (4.1 Å)

Li et al., 2015

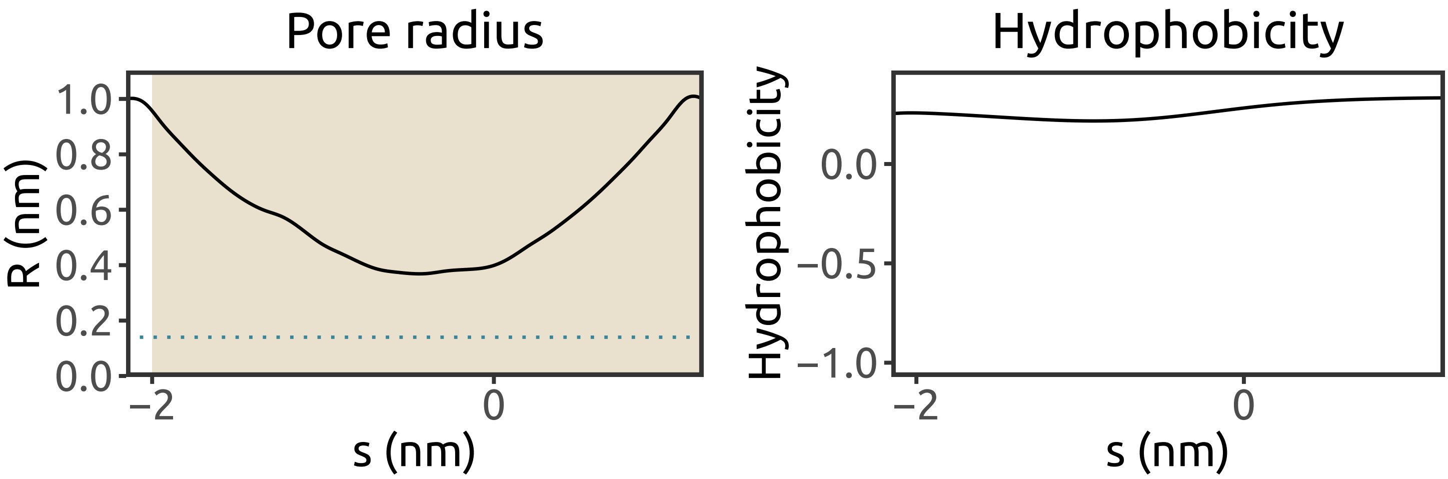


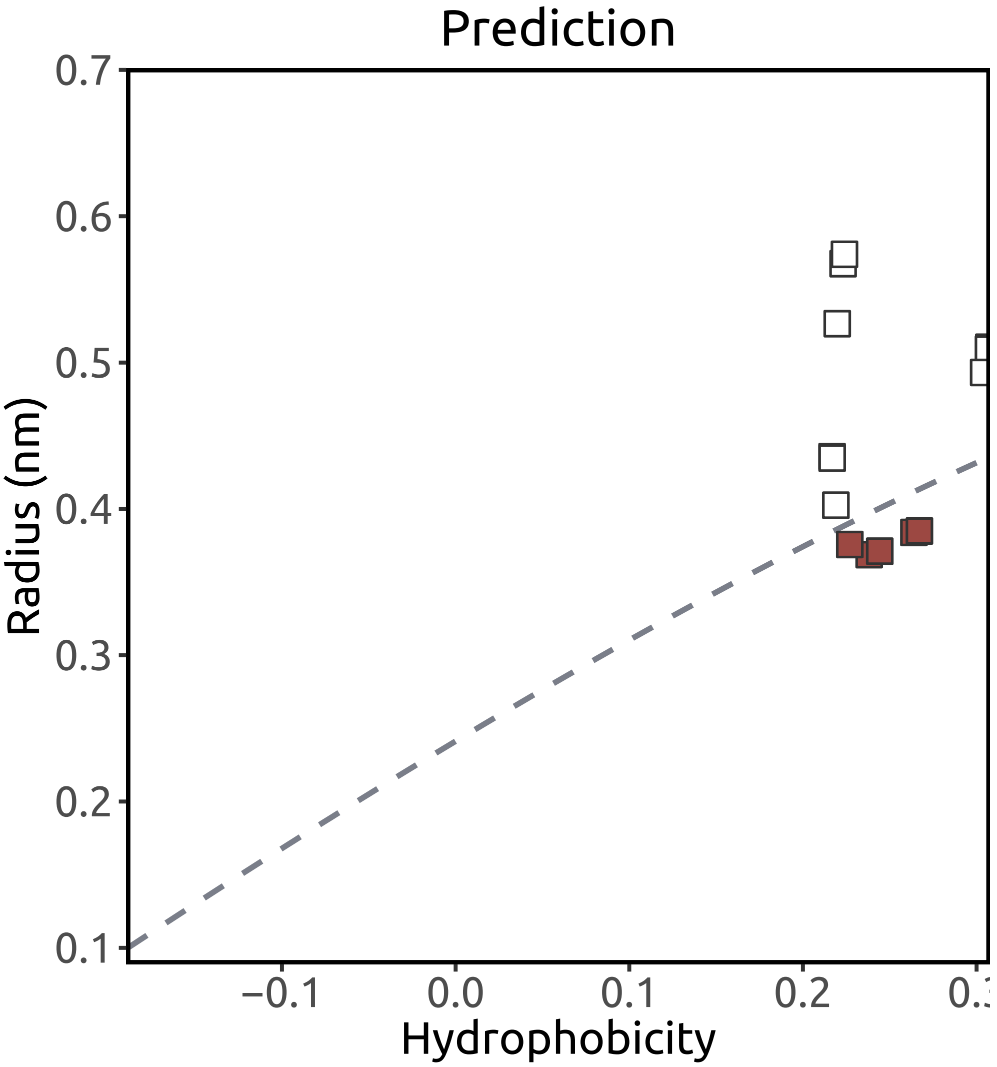


Heuristic score:
0.11 (n = 5)

Simulation result:
hydrated channel

# MscL (PDB ID: 4Y7K)

*Methanosarcina acetivorans*
X−ray (3.5 Å)

Li et al., 2015

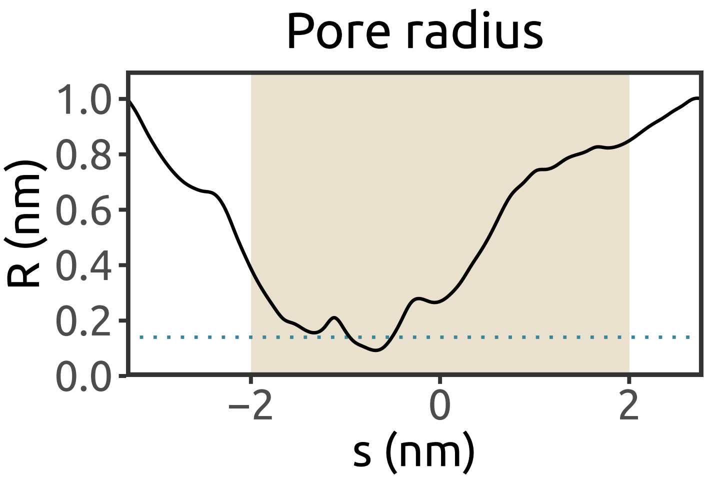


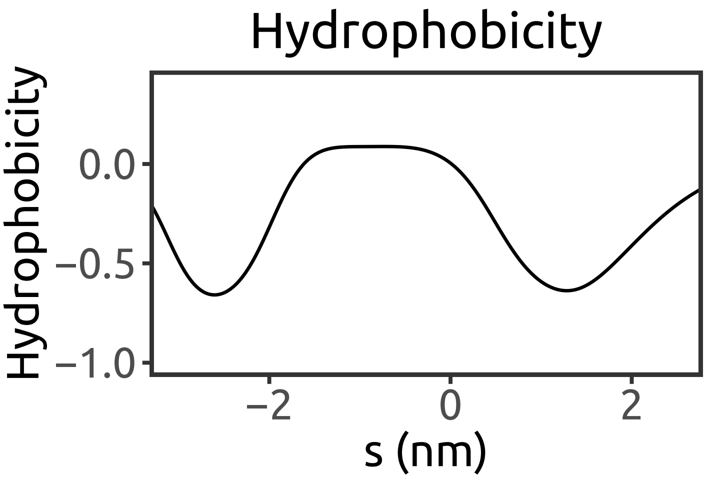


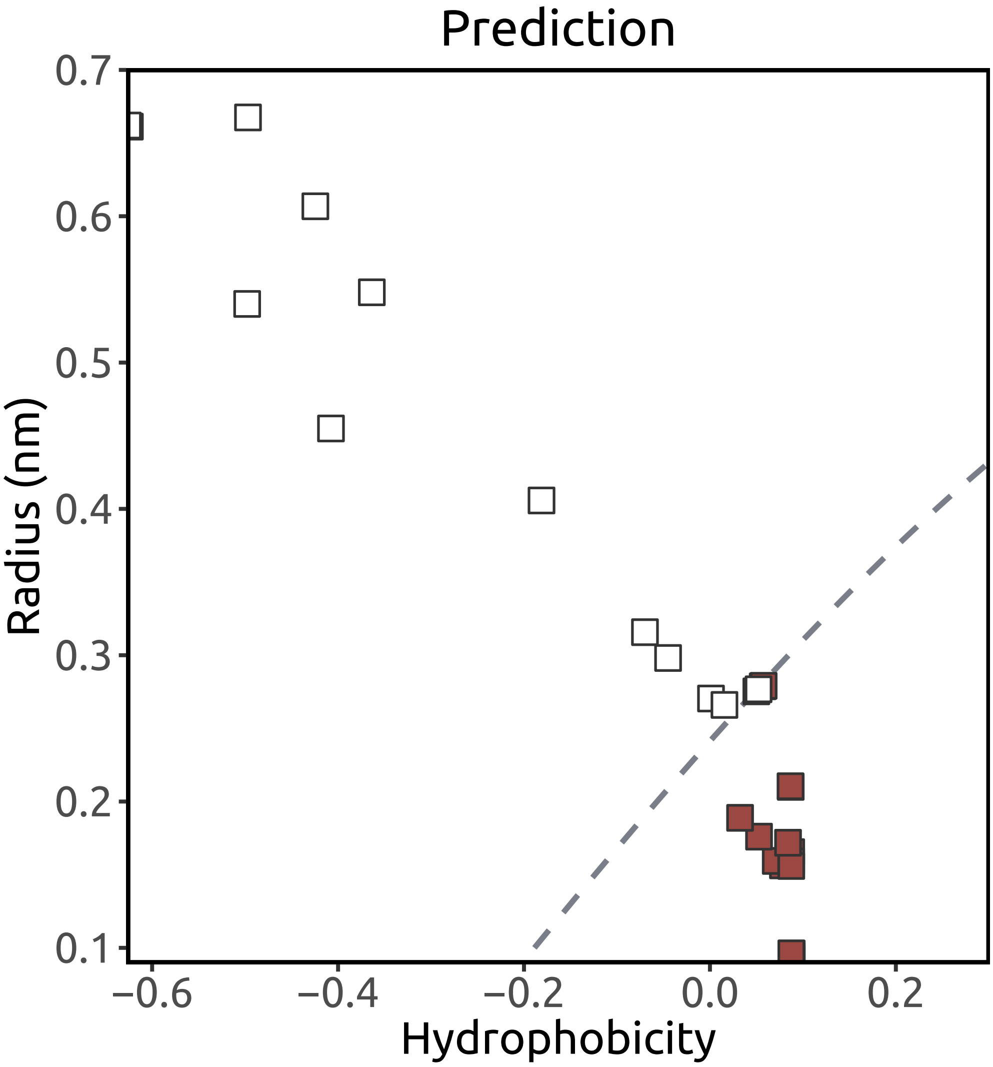


Heuristic score:
1.83 (n = 17)

Simulation result:
barrier to water

# MscS (PDB ID: 2OAU)

*Escherichia coli*
X−ray (3.7 Å)

Bass et al., 2002

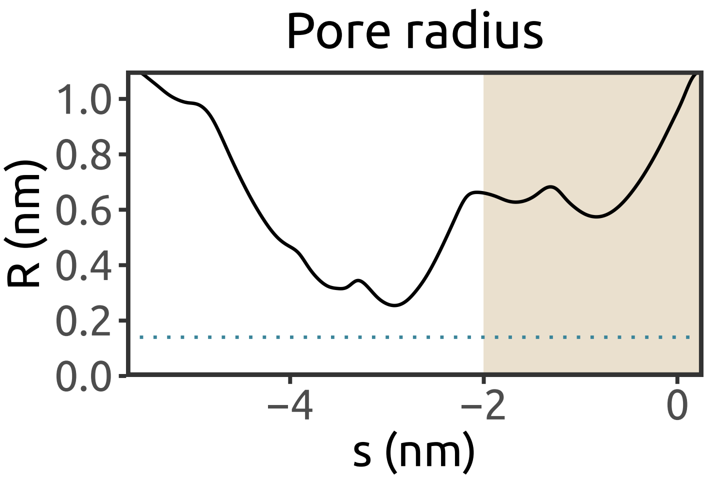


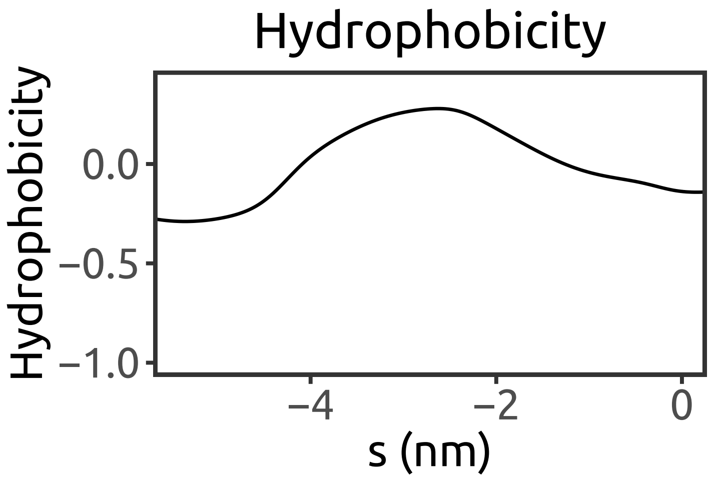


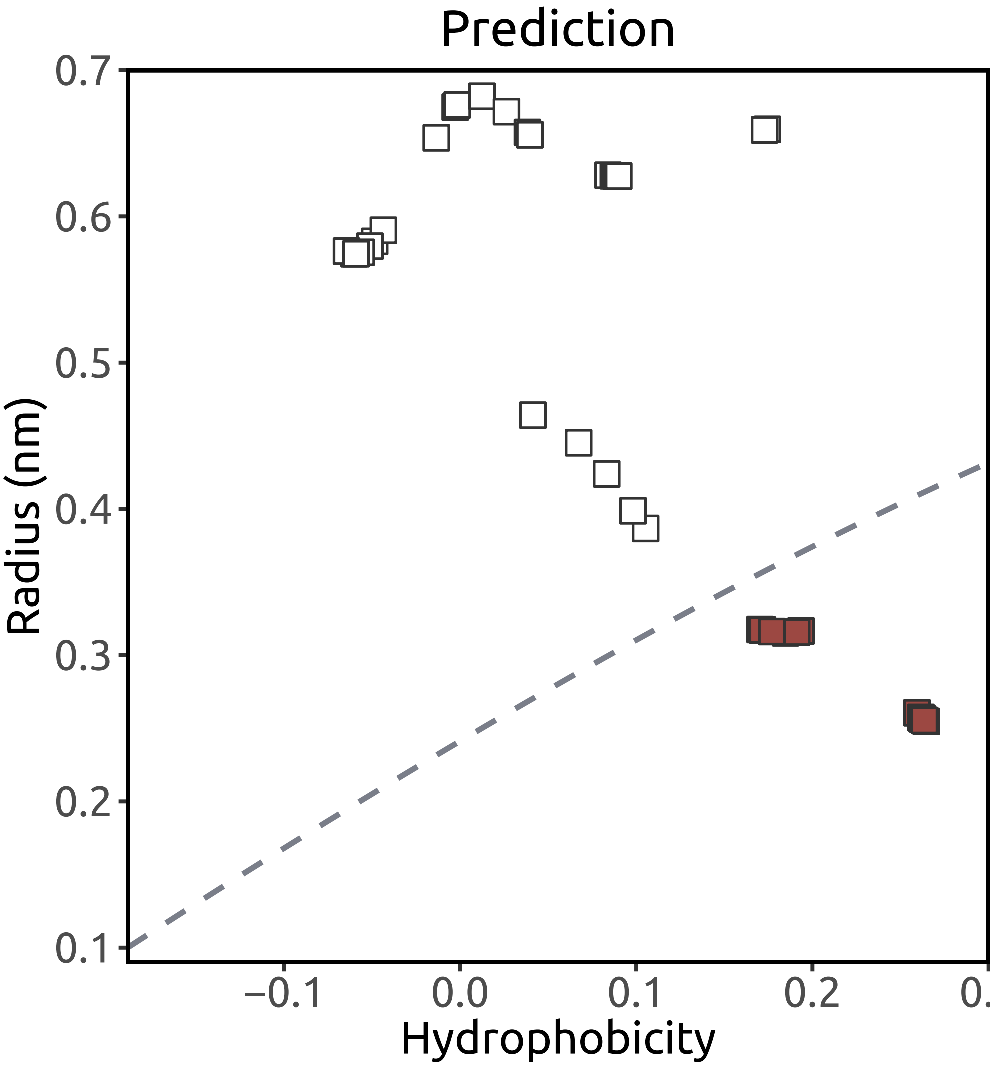


Heuristic score:
1.03 (n = 12)

Simulation result:
barrier to water

# MscS (PDB ID: 2VV5)

*Escherichia coli*

X–ray (3.45 Å)

Wang et al., 2008

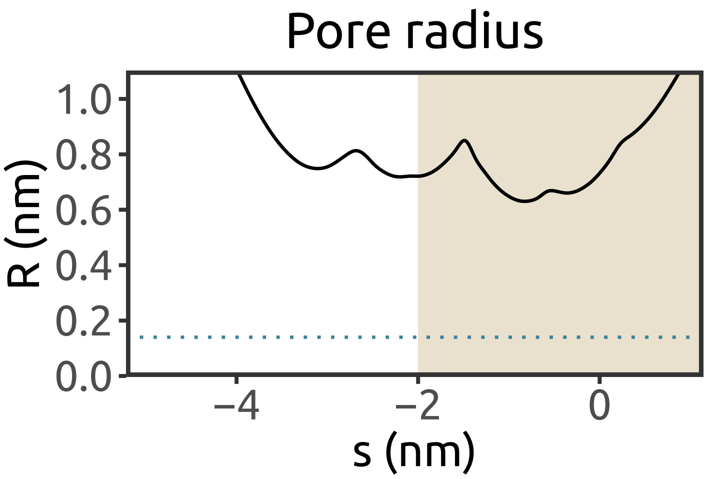


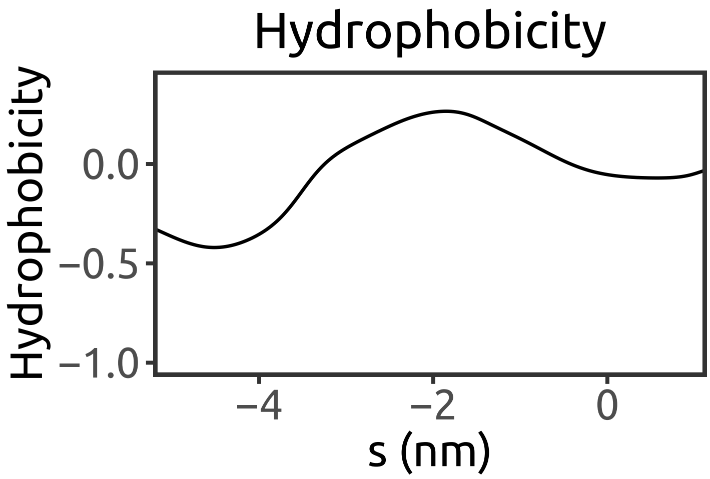


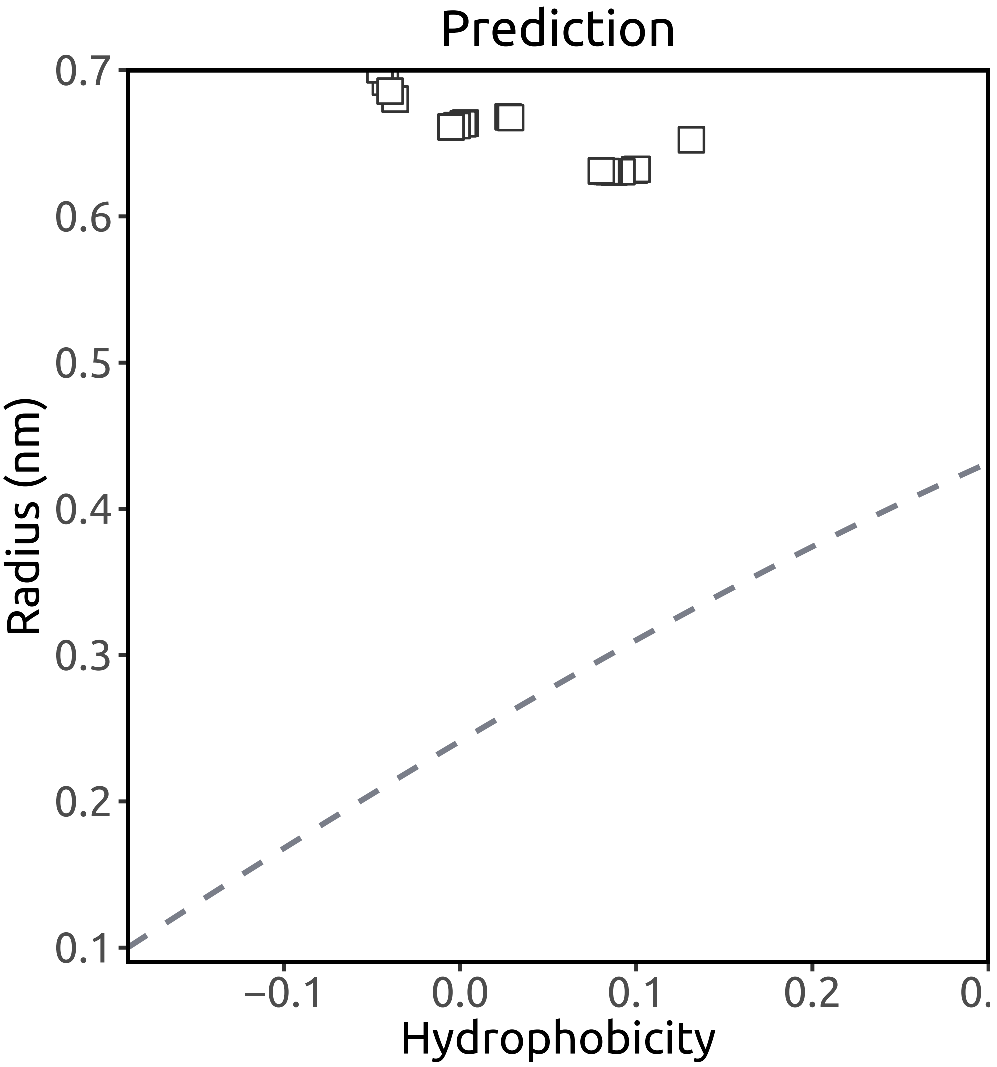


Heuristic score:

0 (n = 0)

Simulation result:

hydrated channel

# NNT (PDB ID: 5UNI)

*Thermus thermophilus*
X–ray (2.2 Å)

Padayatti et al., 2017

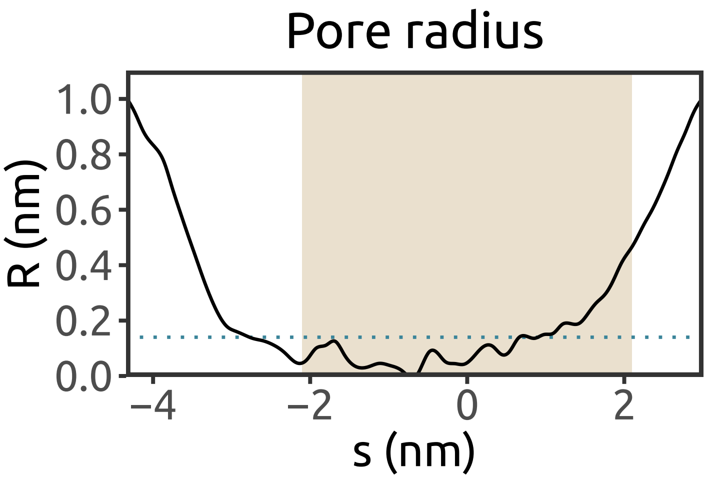


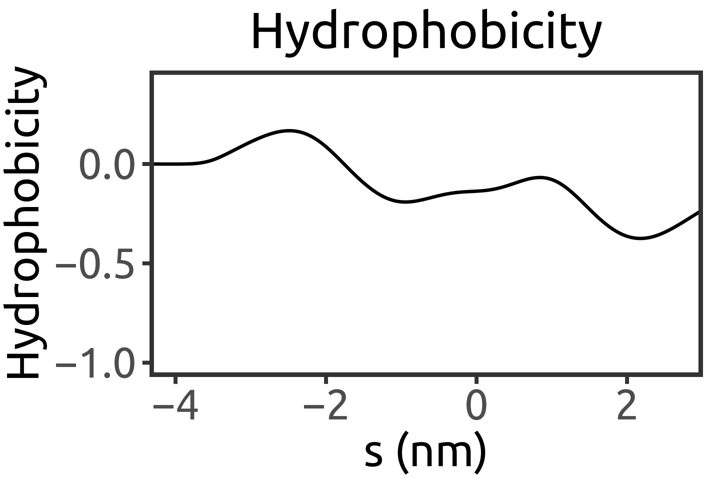


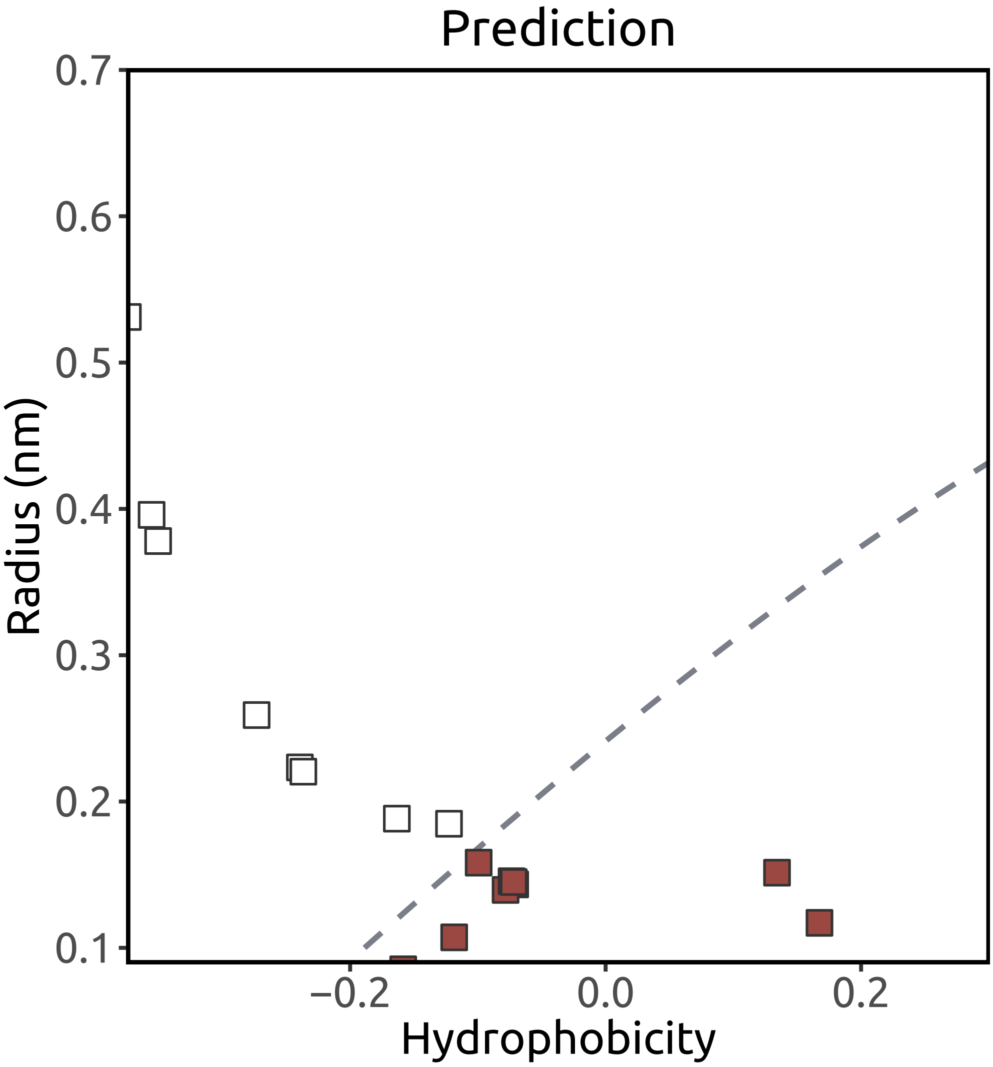


Heuristic score:

1.72 (n = 20)

Simulation result:

barrier to water

# Orai (PDB ID: 4HKR)

*Drosophila melanogaster*

X–ray (3.35 Å)

Hou et al., 2012

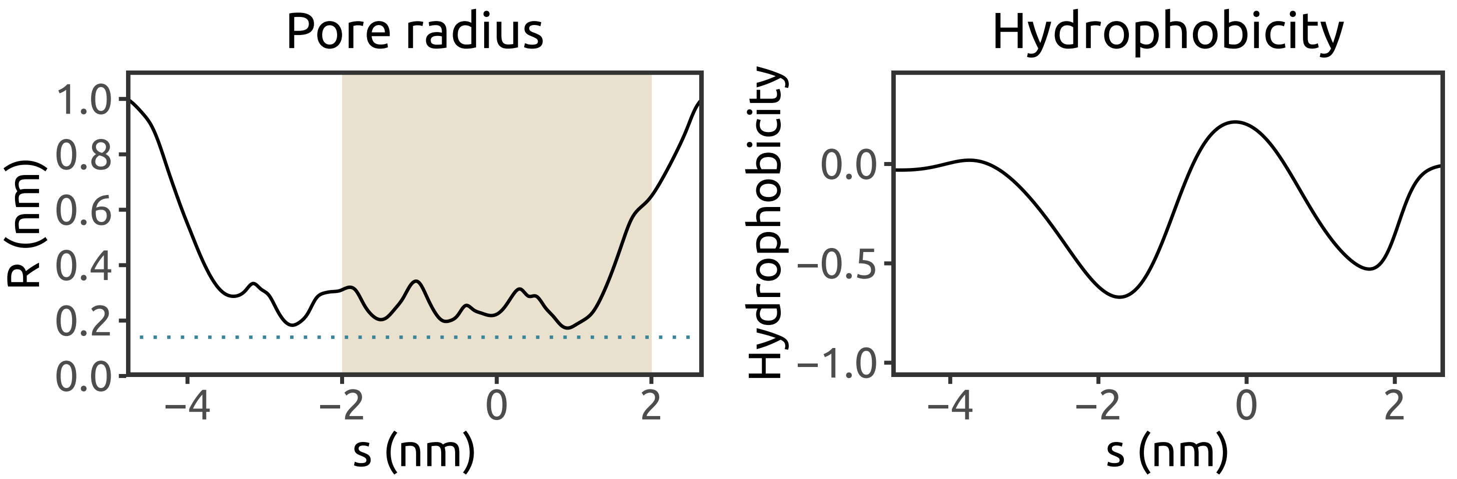


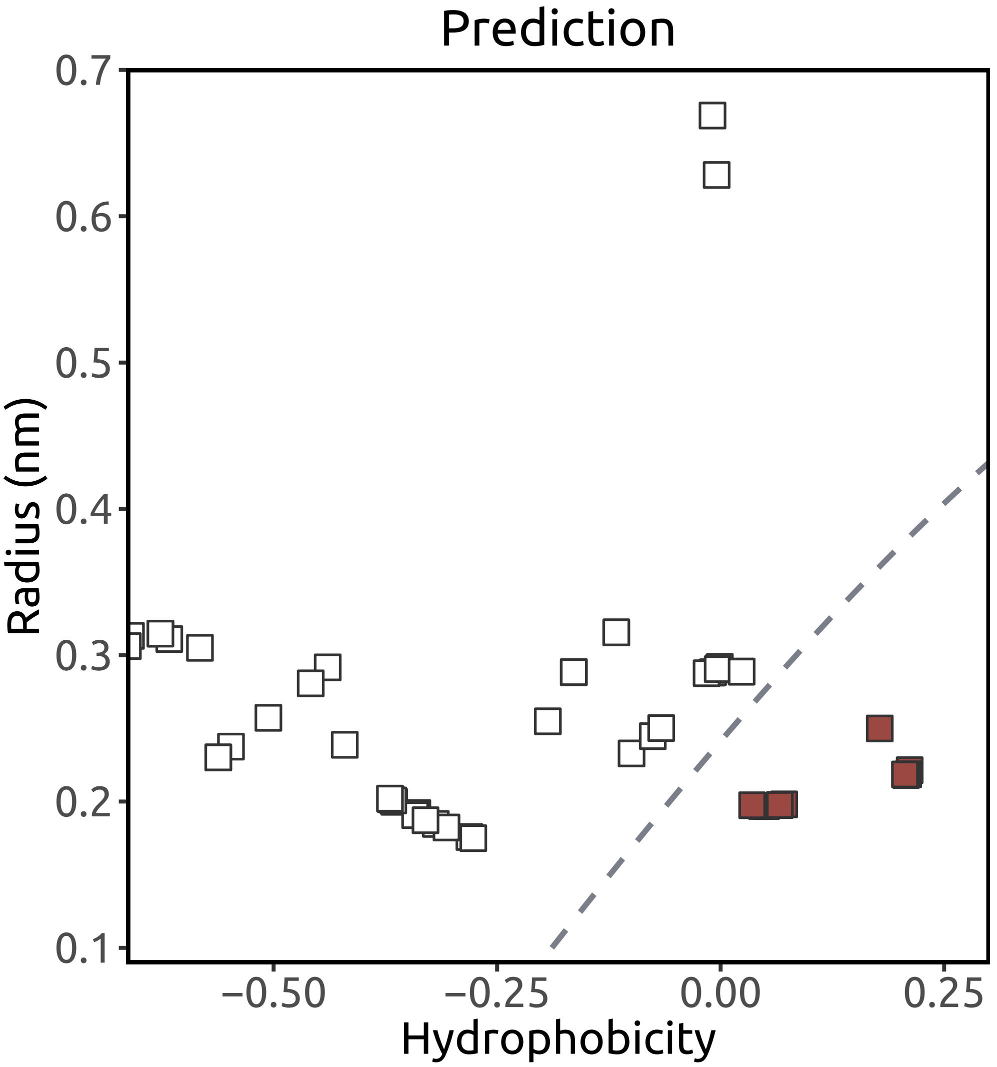


Heuristic score:

1.18 (n = 12)

Simulation result:

barrier to water

# Piezo1 (PDB ID: 6BPZ)

*Mus musculus*
cryo–EM (3.8 Å)

Saotome et al., 2017

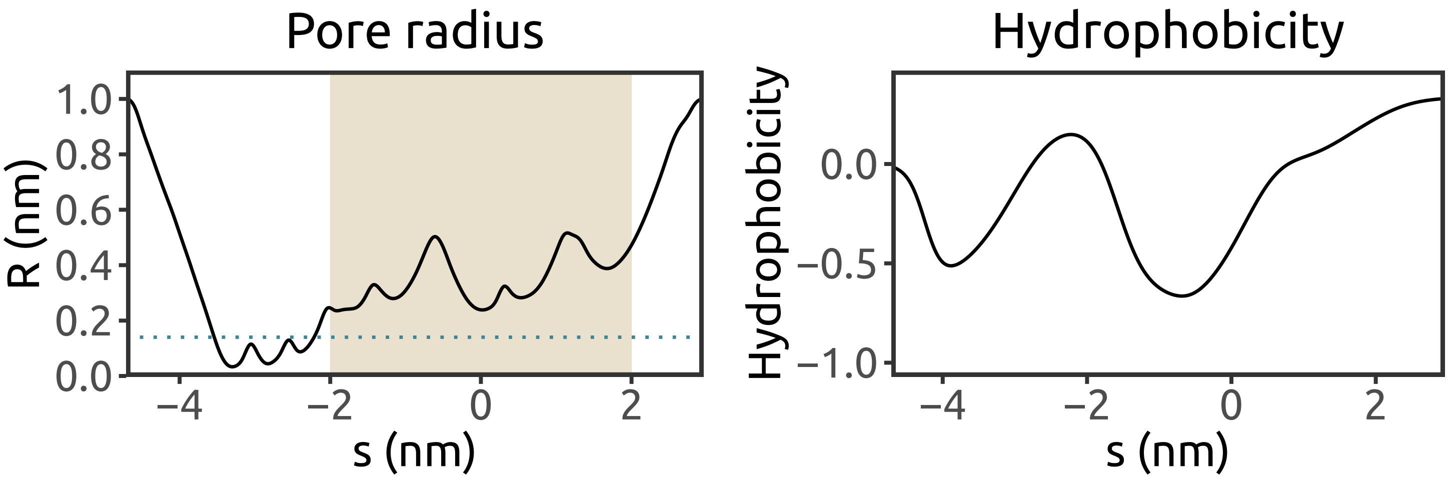


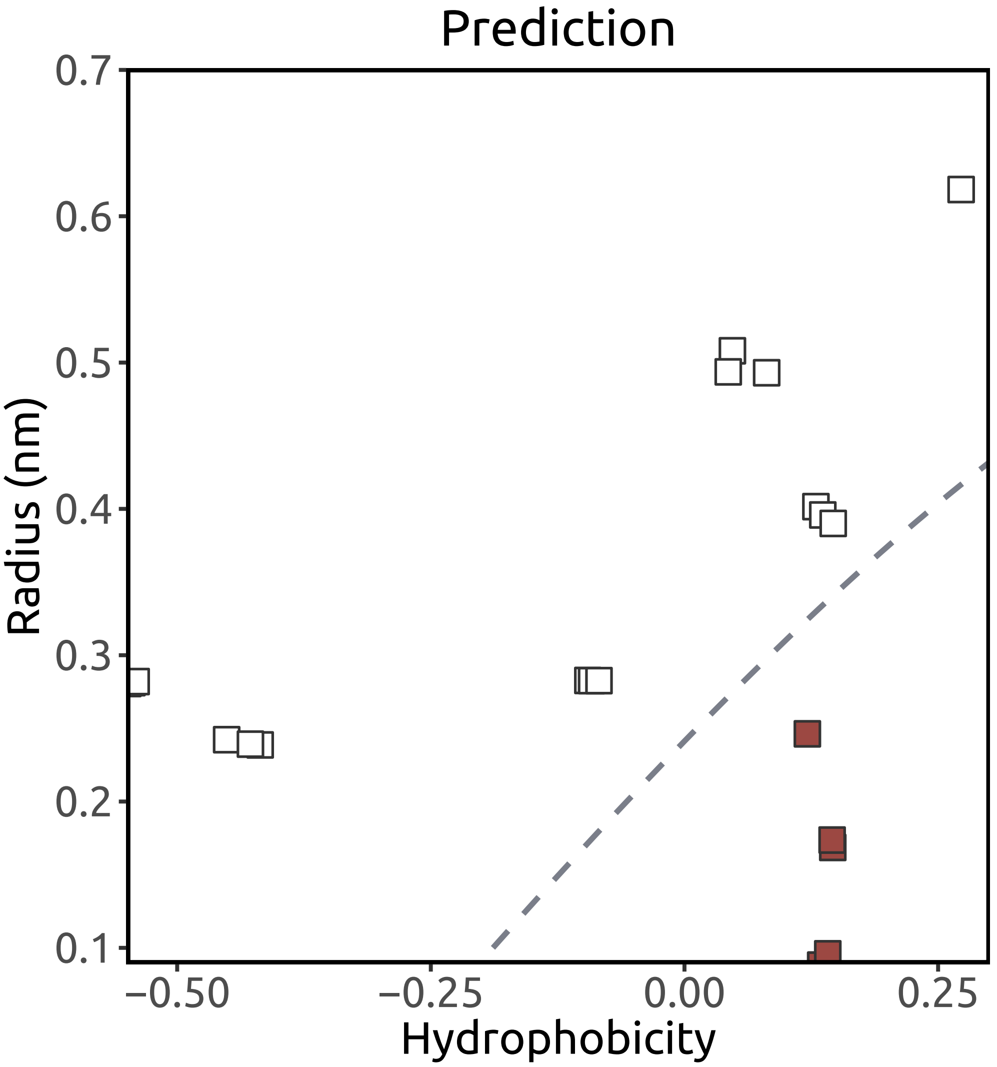


Heuristic score:
1.33 (n = 9)

Simulation result:
barrier to water

# TMEM175 (PDB ID: 5VRE)

*Chamaesiphon minutus*

X–ray (3.30 Å)

Lee et al., 2017

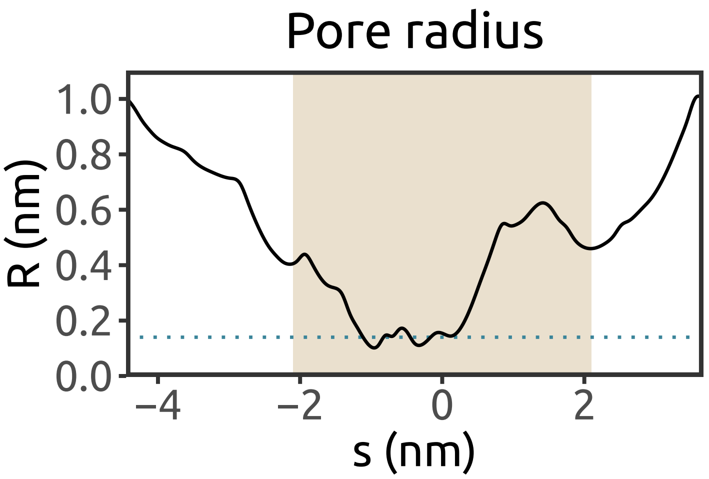


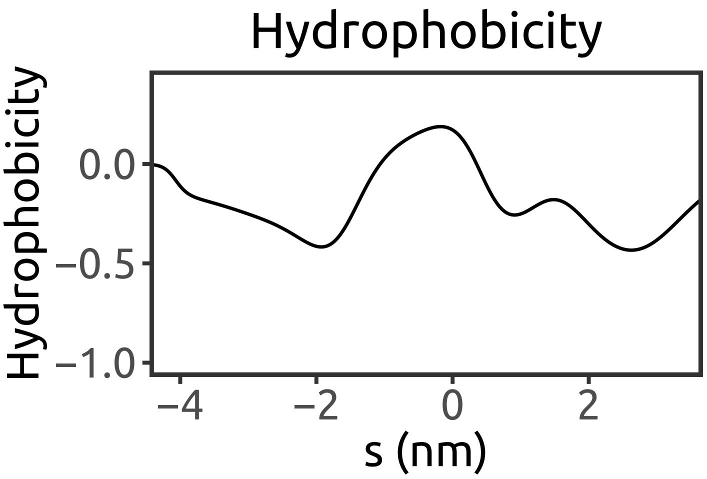


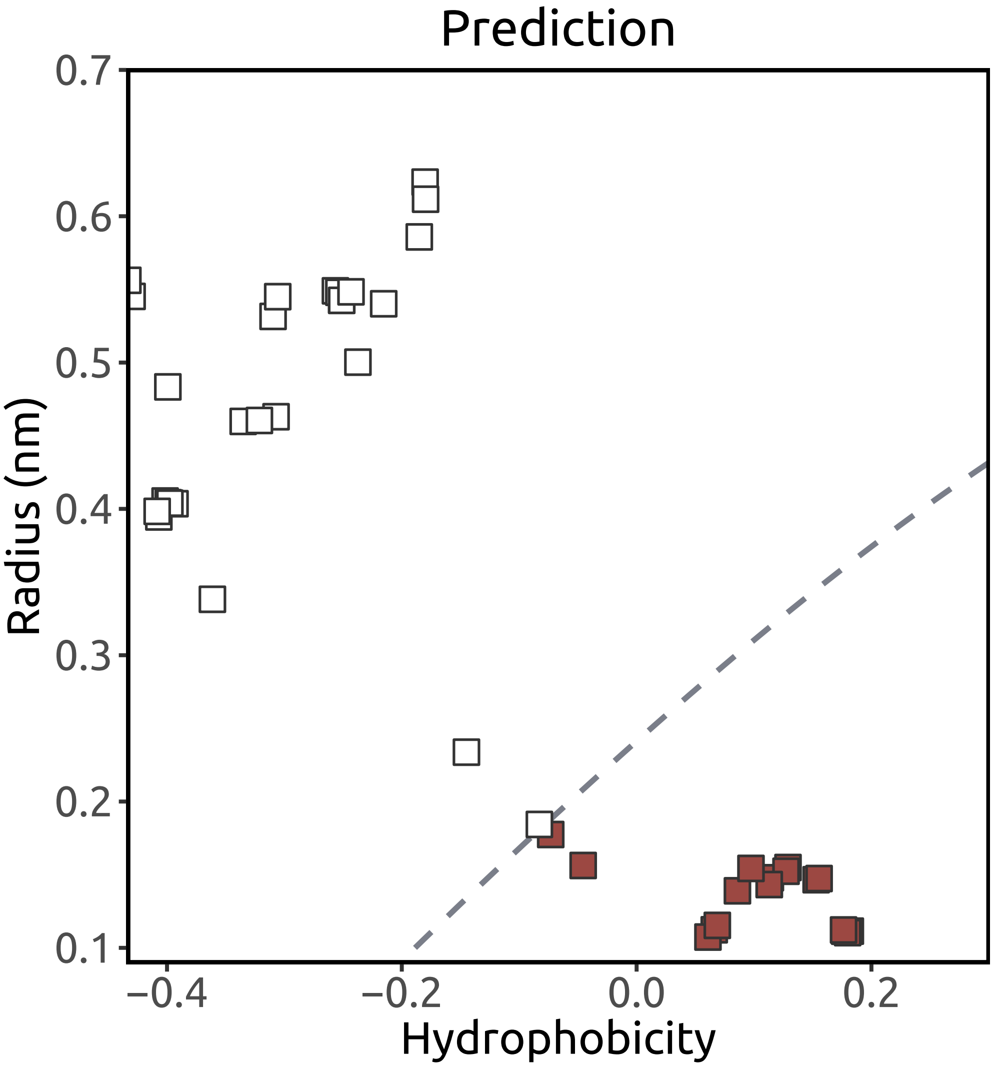


Heuristic score:

2.63 (n = 18)

Simulation result:

barrier to water

# TRIC (PDB ID: 5EGI)

*Caenorhabditis elegans*

X−ray (3.3 Å)

Yang et al., 2016

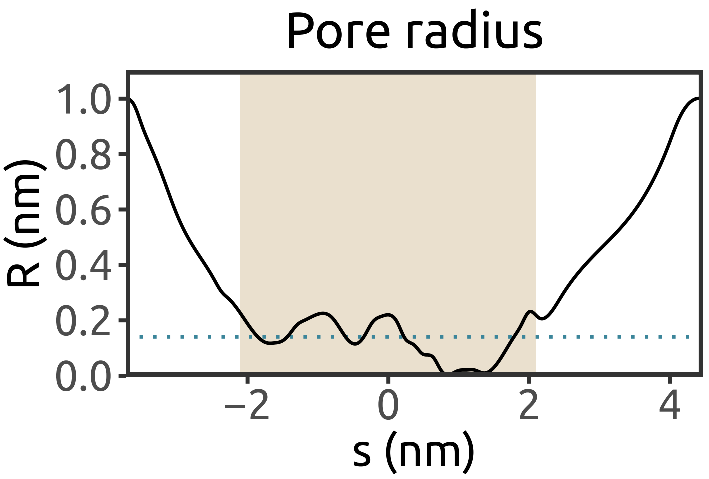


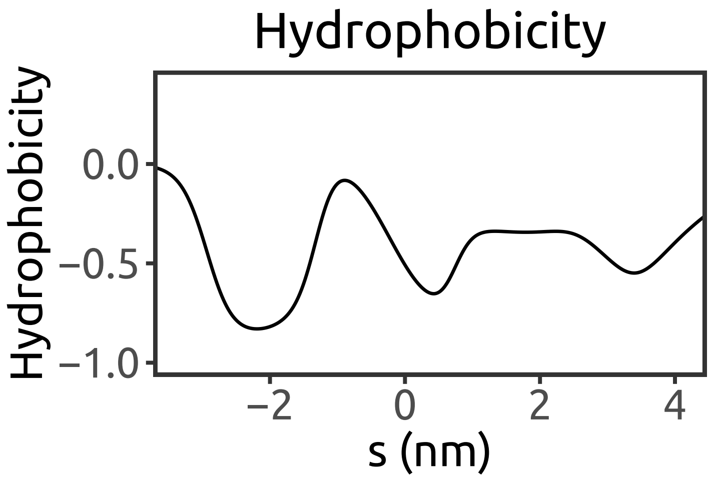


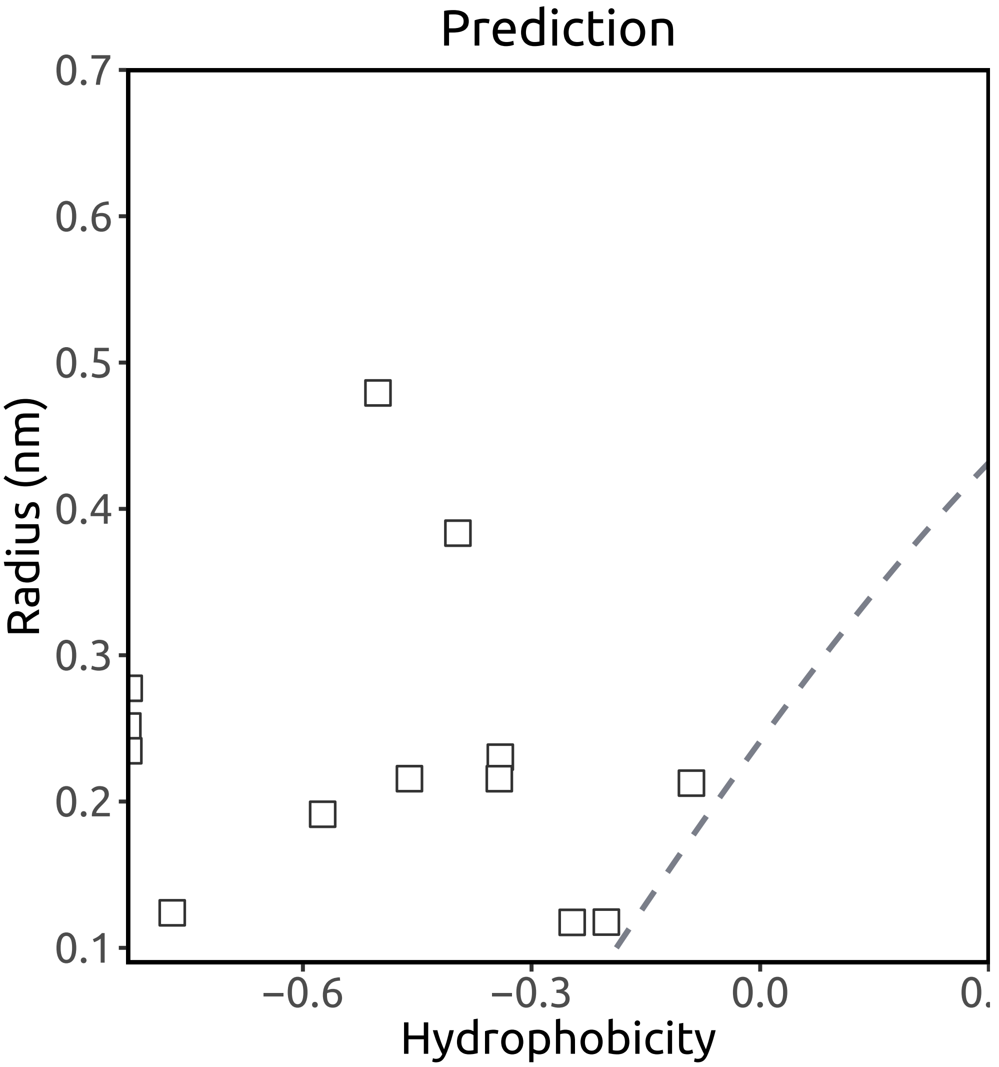


Heuristic score:

0 (n = 0)

Simulation result:

barrier to water

# TRIC (PDB ID: 5EIK)

*Caenorhabditis elegans*

X−ray (2.3 Å)

Yang et al., 2016

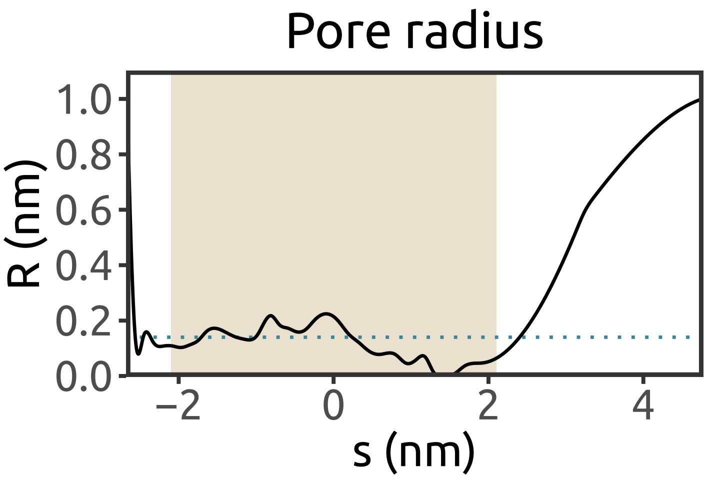


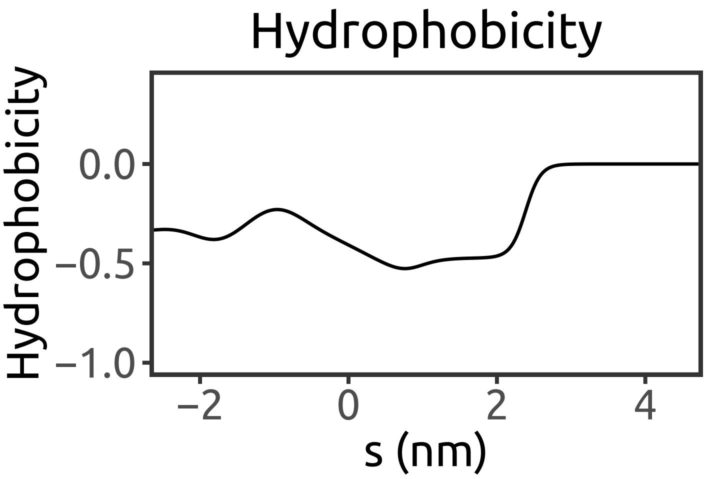


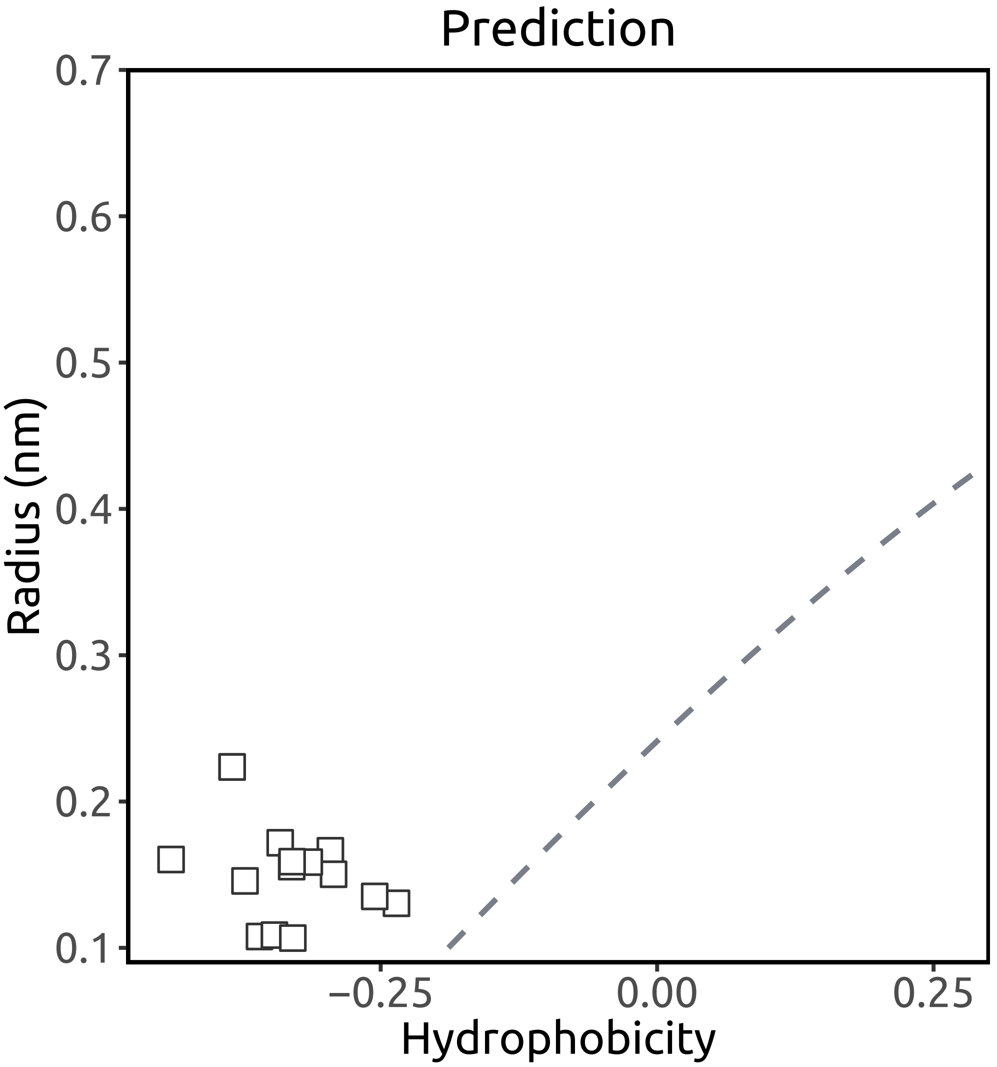


Heuristic score:

0 (n = 0)

Simulation result:

barrier to water

# TRIC (PDB ID: 5WUC)

*Sulfolobus acidocaldarius*
X−ray (1.6 Å)

Su et al., 2017

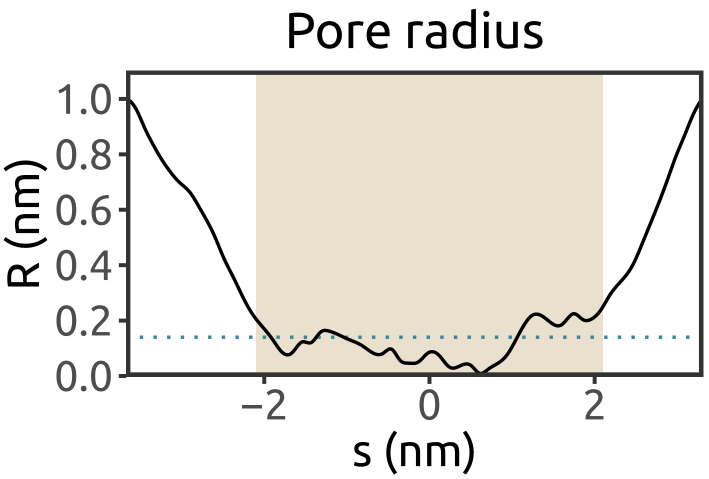


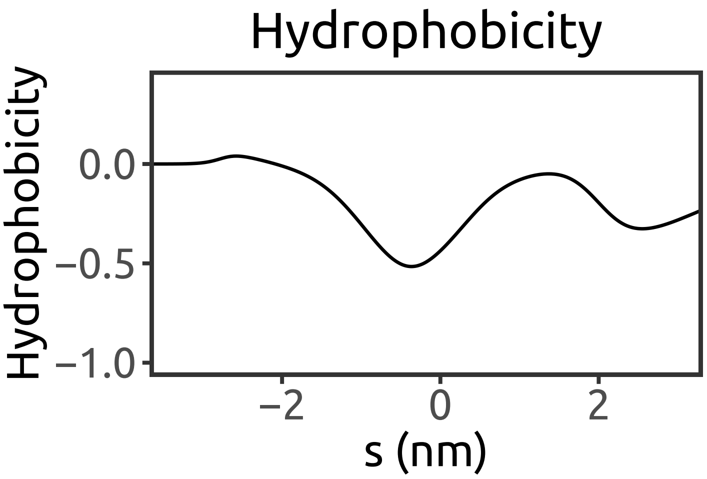


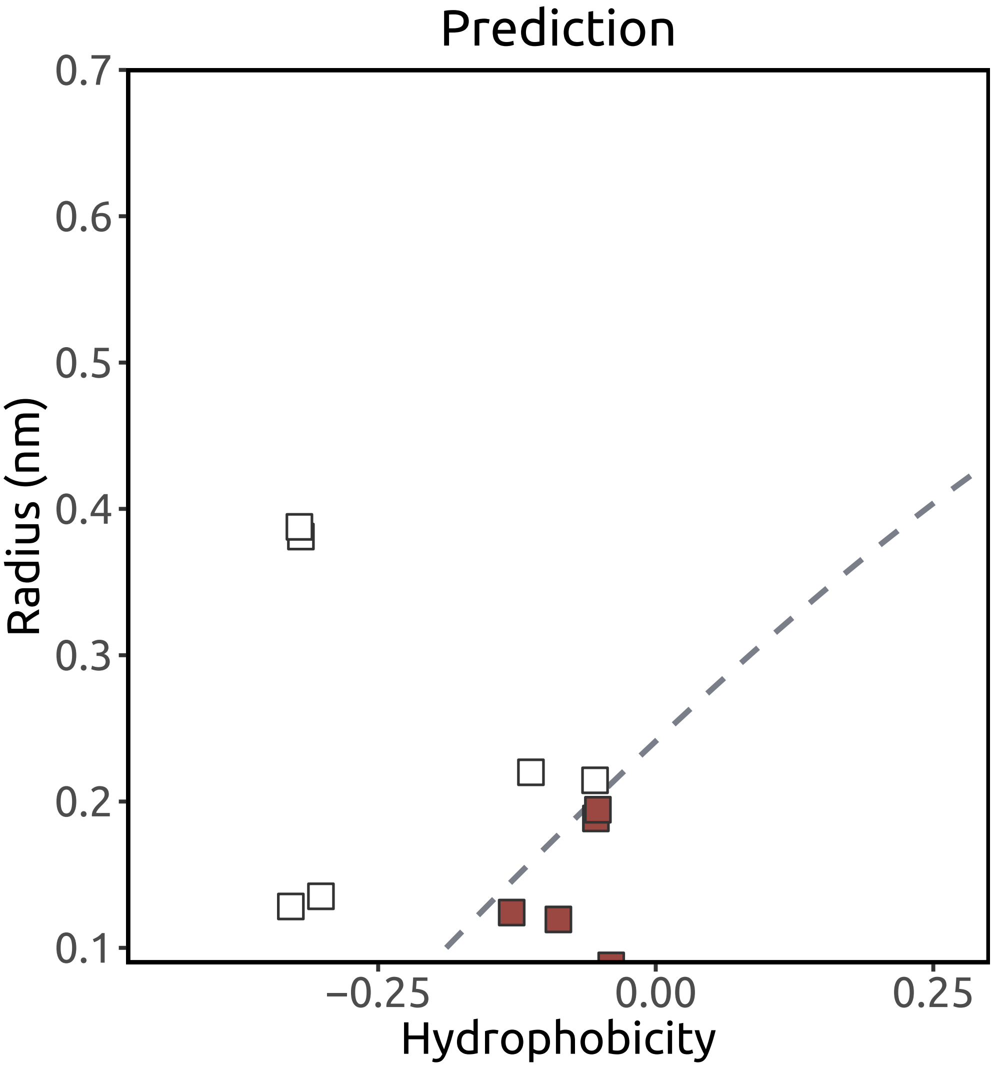


Heuristic score:
0.27 (n = 6)

Simulation result:
barrier to water

# TRIC (PDB ID: 5WUE)

*Sulfolobus acidocaldarius*

X−ray (2.4 Å)

Su et al., 2017

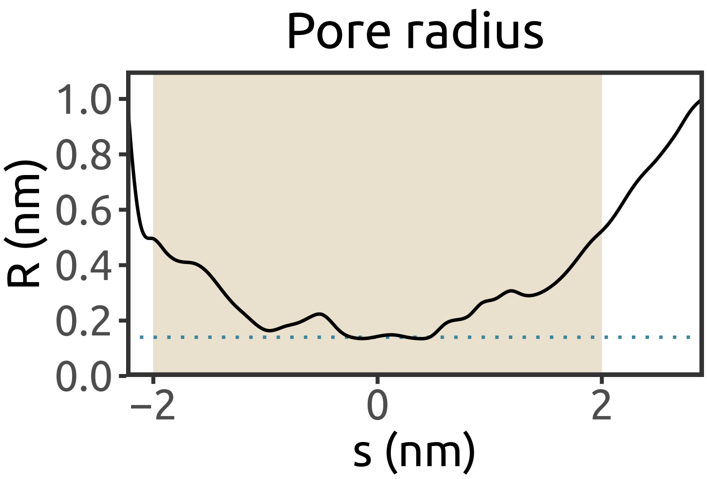


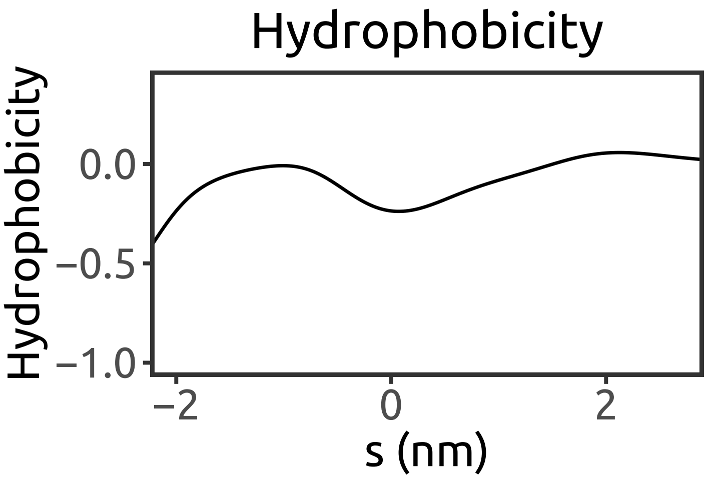


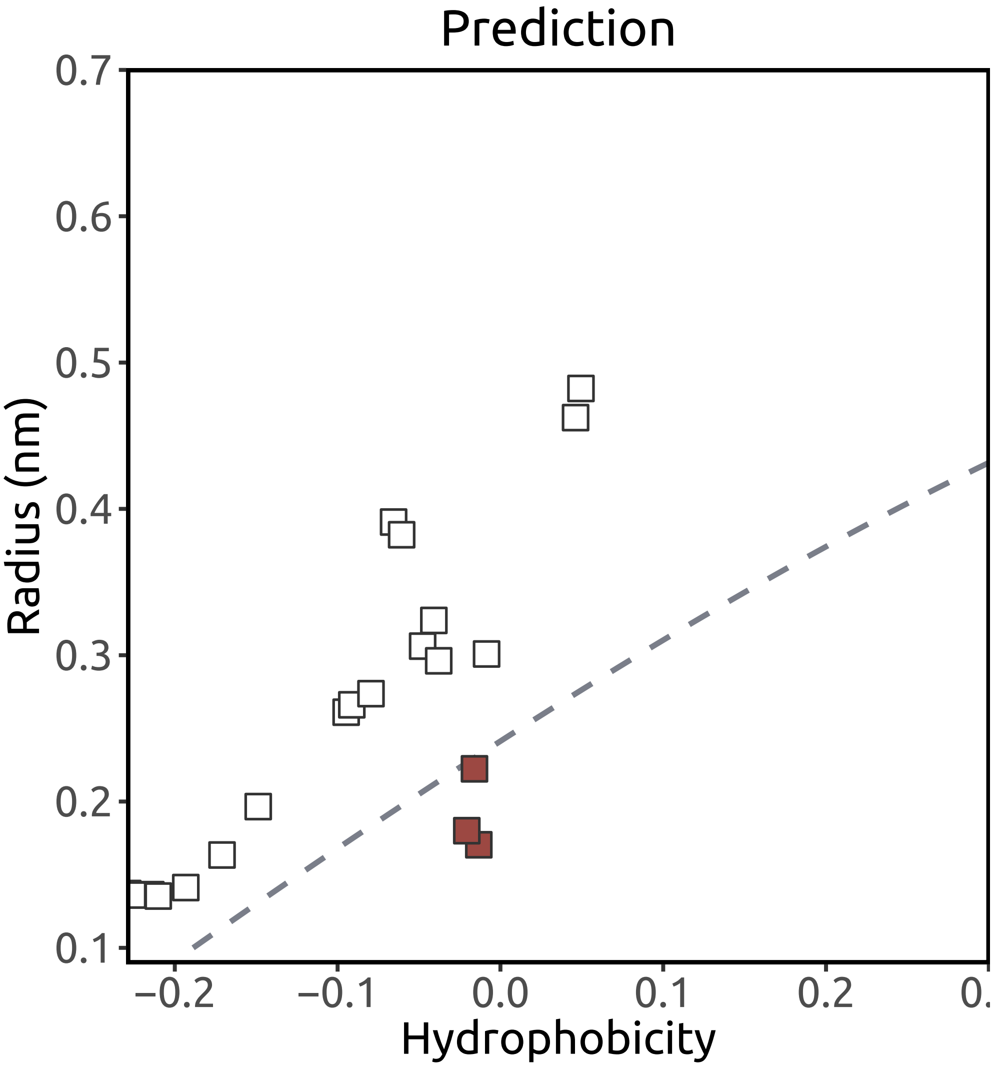


Heuristic score:

0.09 (n = 3)

Simulation result:

hydrated channel

# TRIC (PDB ID: 5WUF)

*Colwellia psychrerythraea*
X–ray (2.40 Å)

Su et al., 2017

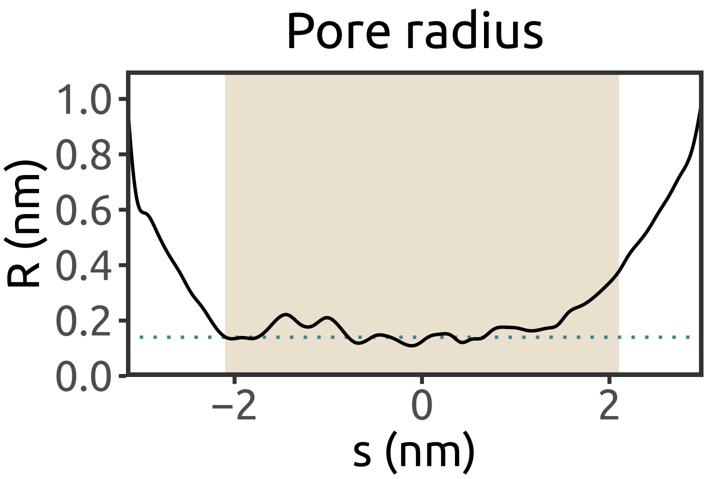


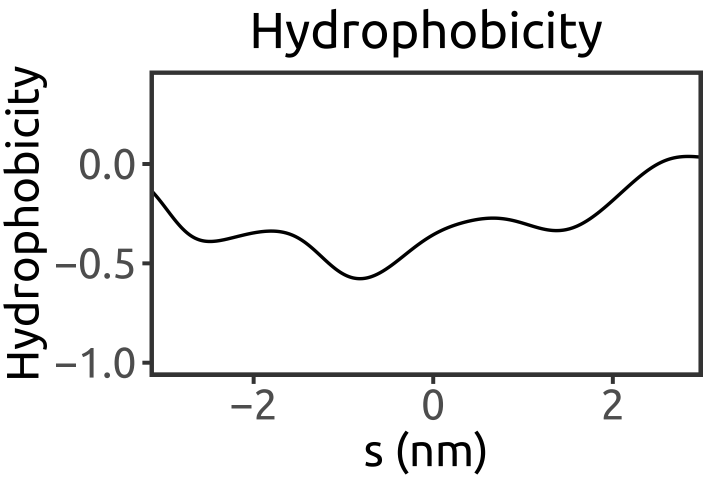


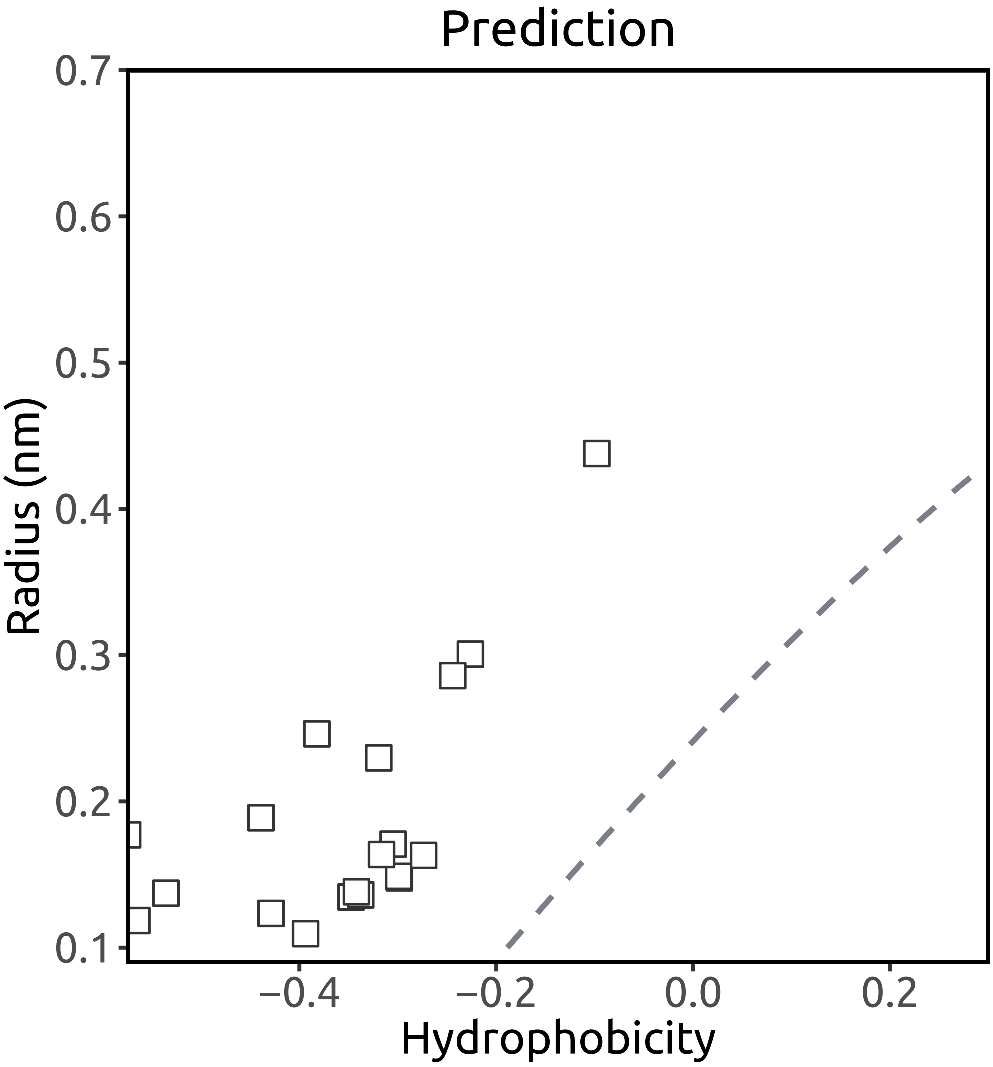


Heuristic score:
0 (n = 0)

Simulation result:
hydrated channel

# YetJ (PDB ID: 4PGR)

*Bacillus subtilis*

X−ray (1.95 Å)

Chang et al., 2014

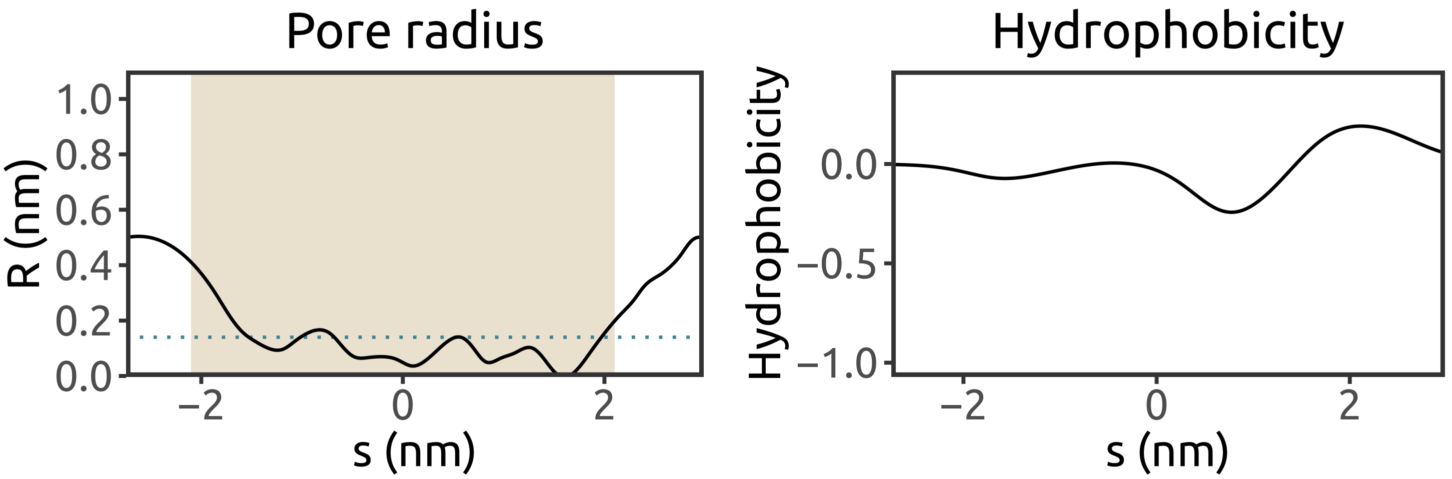


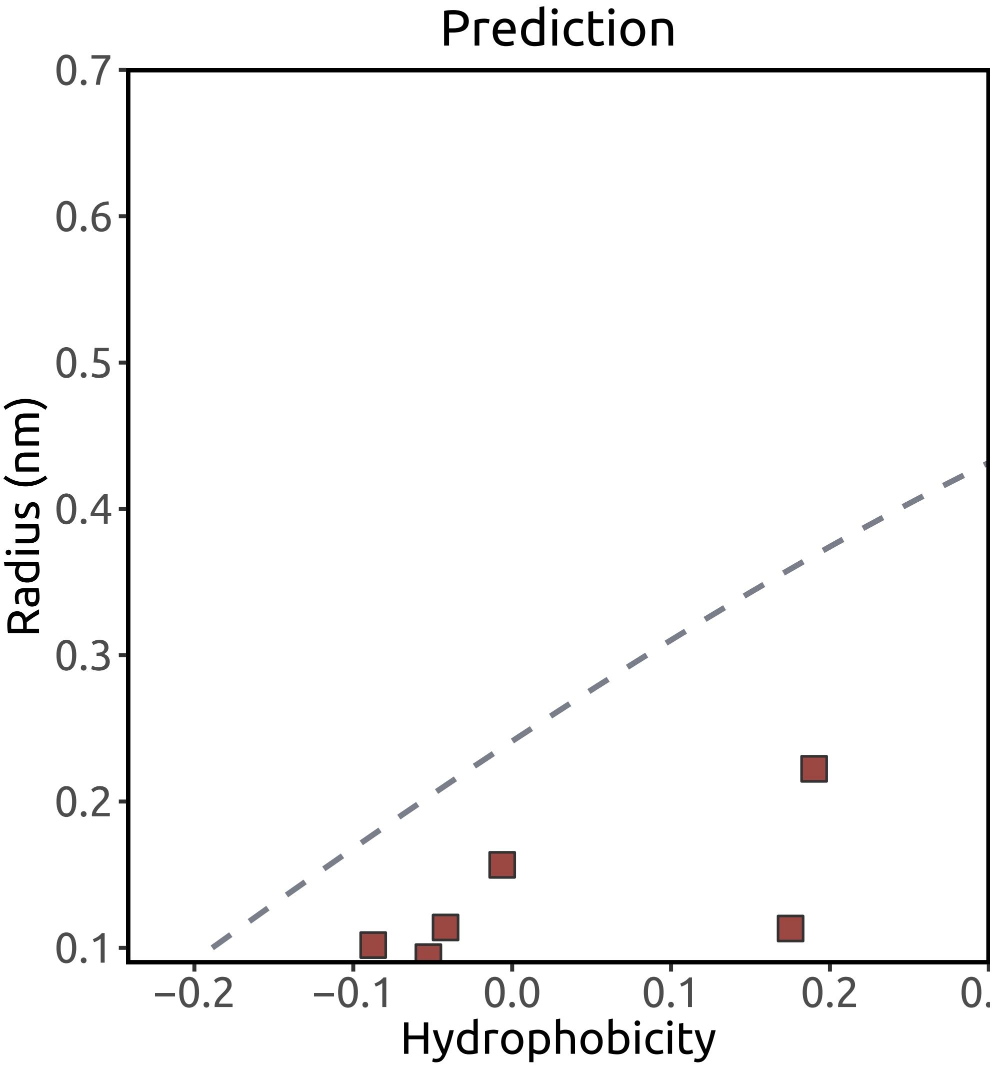


Heuristic score:

1.18 (n = 11)

Simulation result:

barrier to water

# YetJ (PDB ID: 4PGS)

*Bacillus subtilis*

X−ray (2.5 Å)

Chang et al., 2014

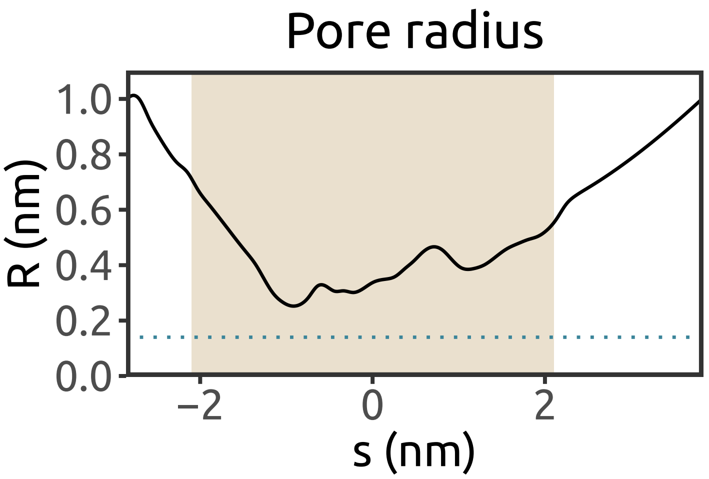


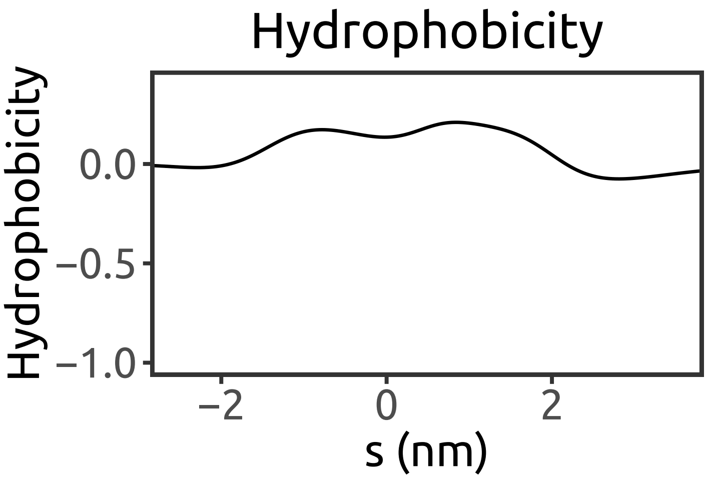


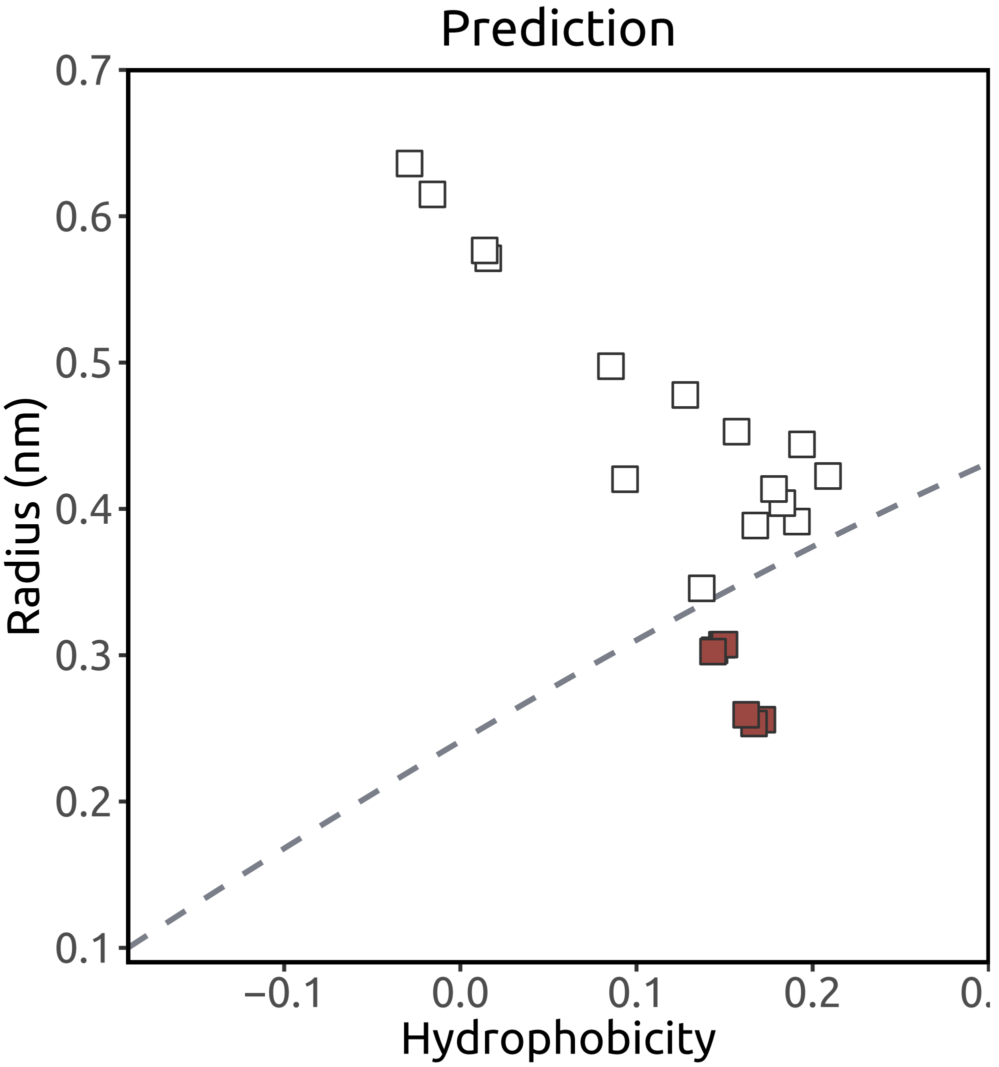


Heuristic score:

0.37 (n = 7)

Simulation result:

barrier to water

# YetJ (PDB ID: 4PGU)

*Bacillus subtilis*

X–ray (3.40 Å)

Chang et al., 2014

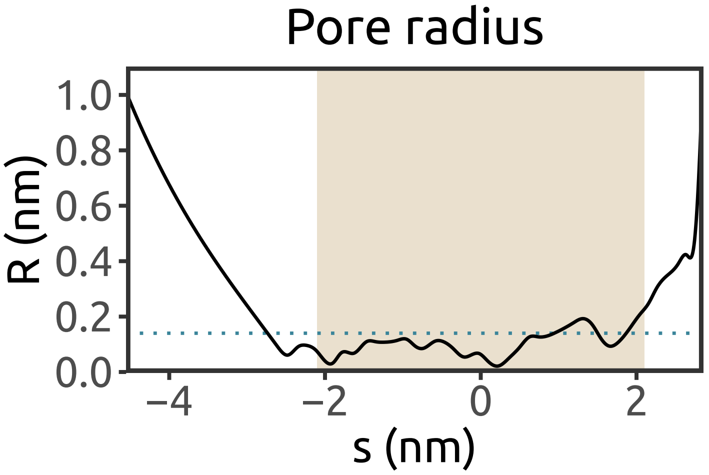


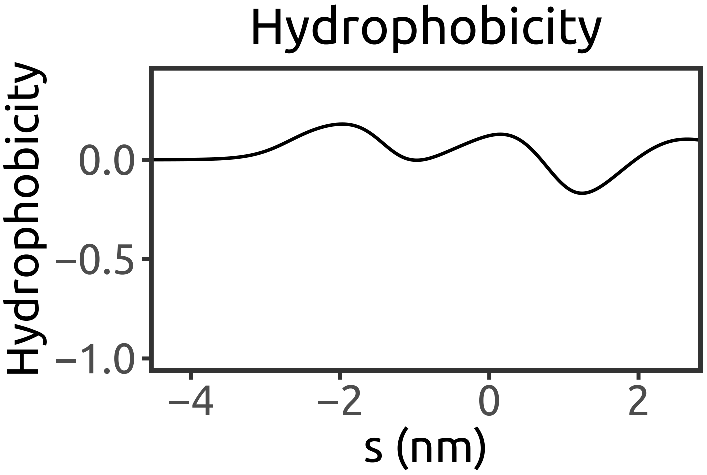


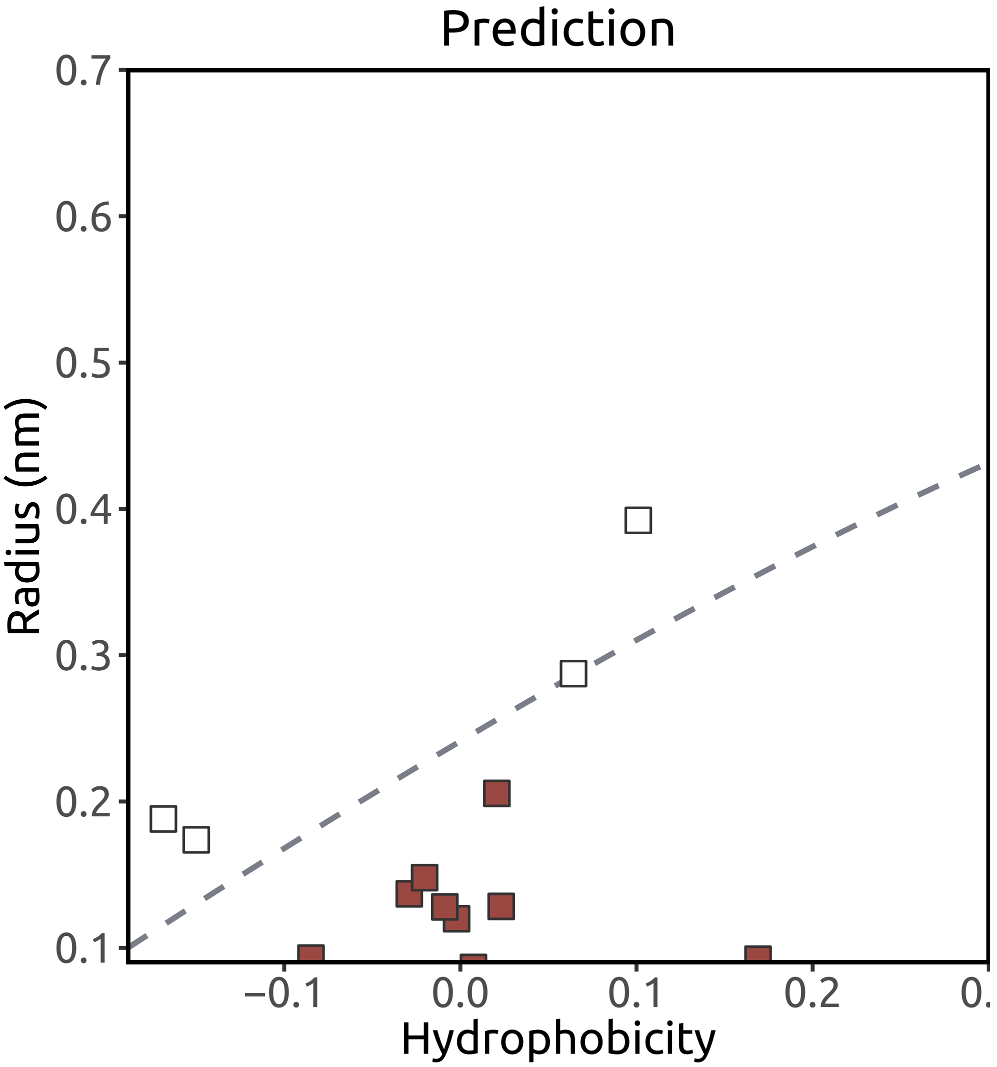


Heuristic score:

2 (n = 14)

Simulation result:

barrier to water

# BEST1 (PDB ID: 4RDQ)

*Gallus gallus*
X−ray (2.85 Å)

Kane et al., 2014

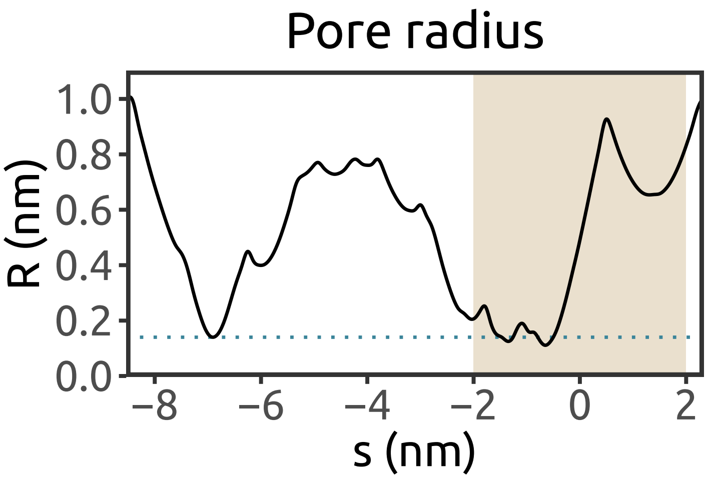


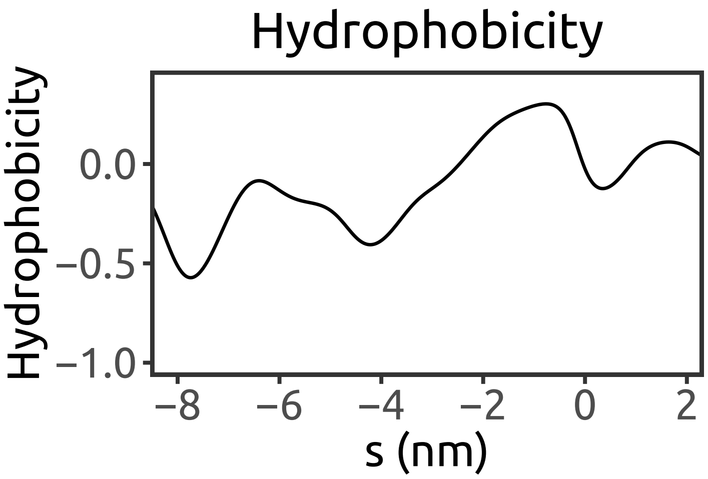


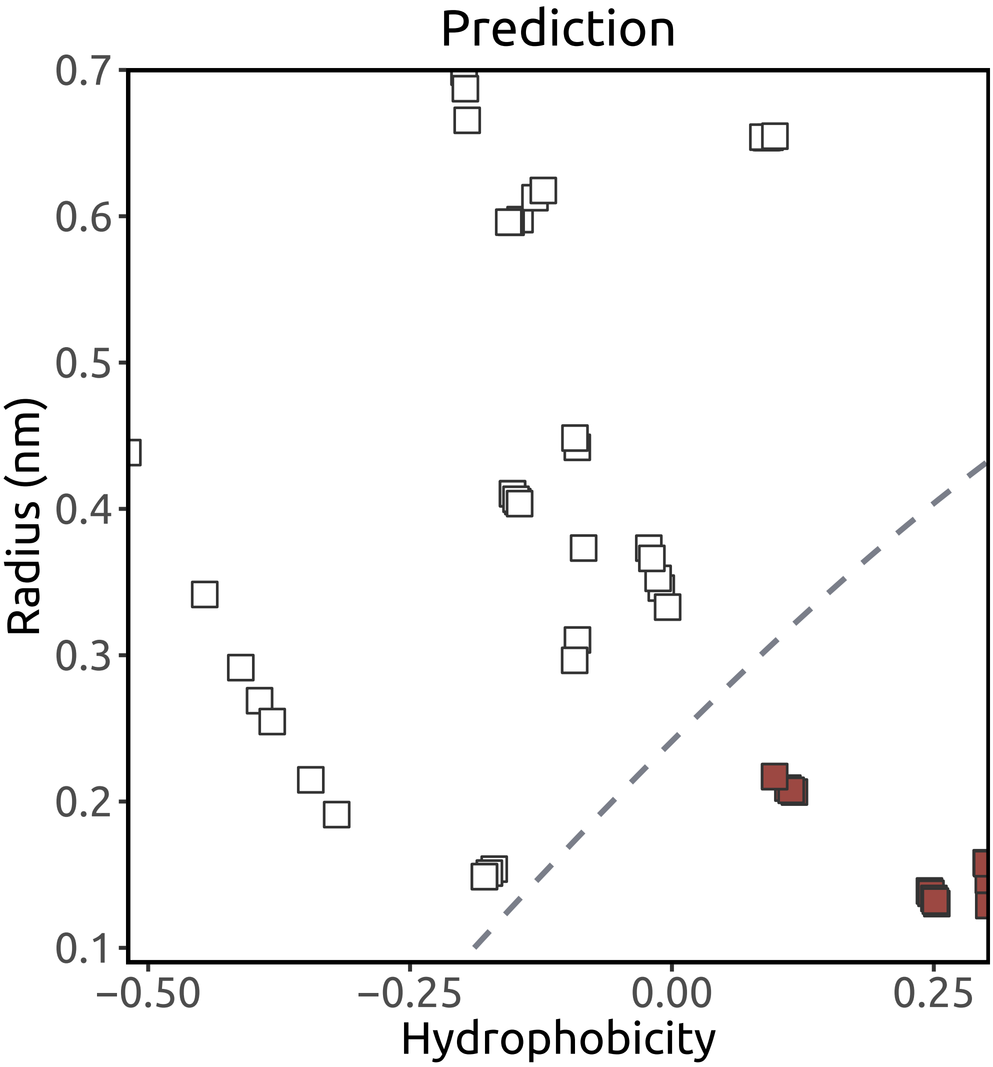


Heuristic score:
2.82 (n = 15)

Simulation result:
barrier to water

# CFTR (PDB ID: 5UAK)

*Homo sapiens*
cryo–EM (3.87 Å)

Liu et al., 2017

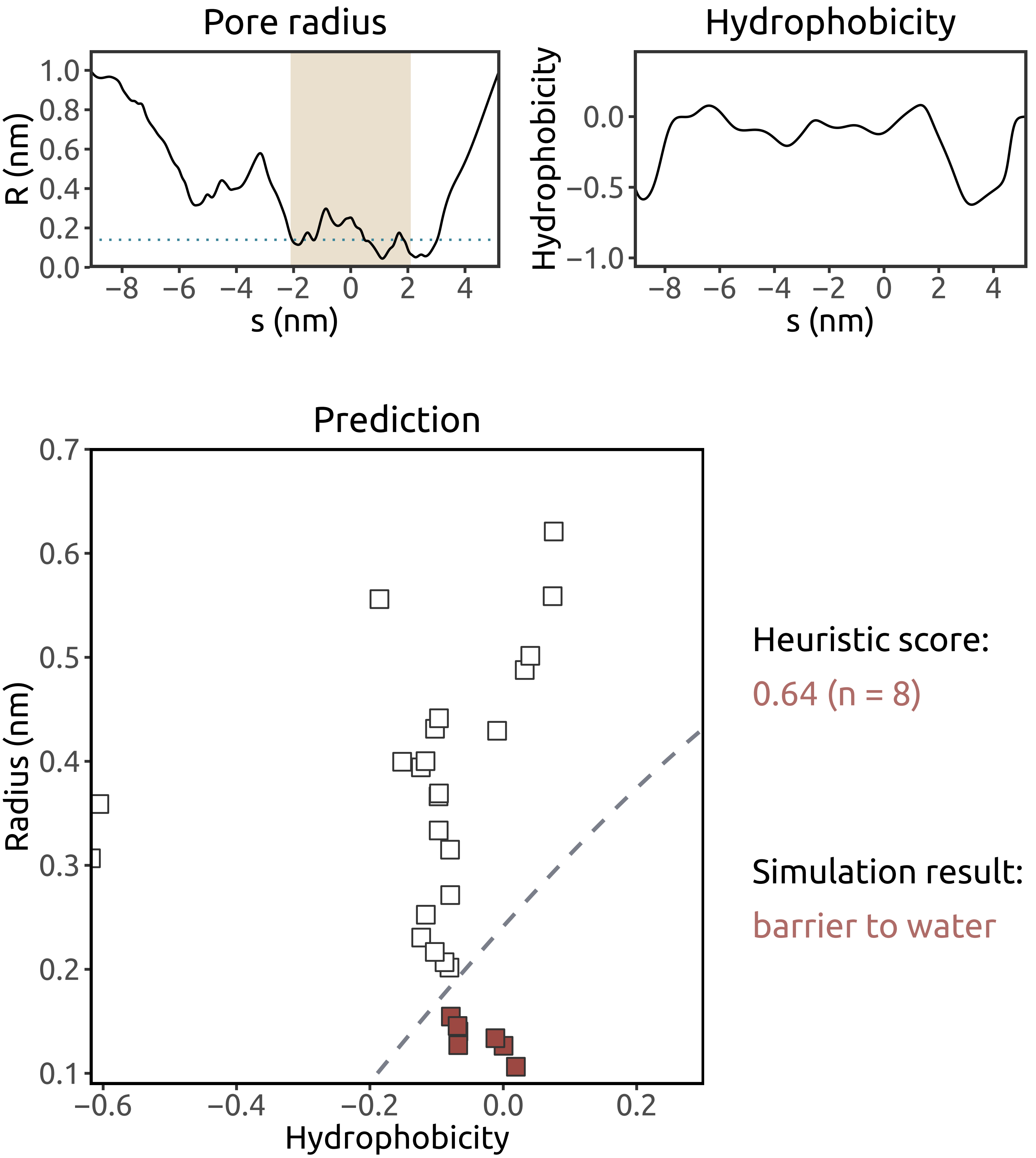


Heuristic score:

0.64 (n = 8)

Simulation result:

barrier to water

# CFTR (PDB ID: 5UAR)

*Danio rerio*
cryo−EM (3.73 Å)

Zhang & Chen, 2016

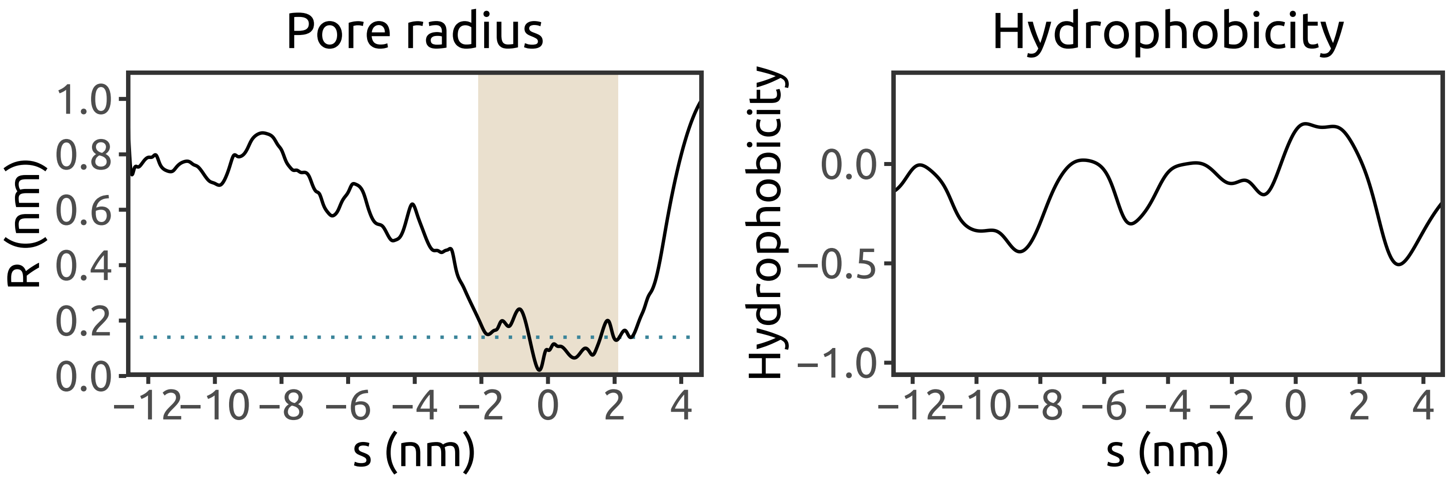


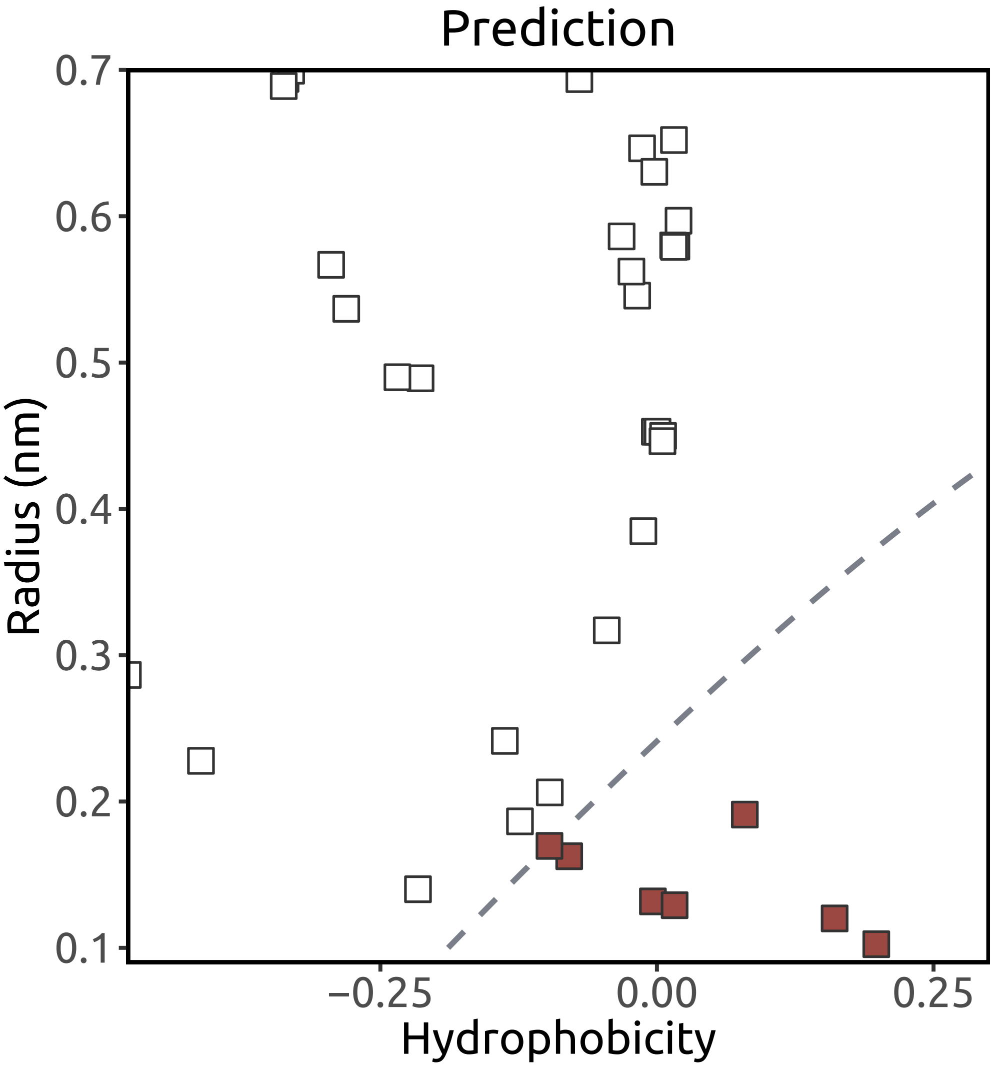


Heuristic score:
1.83 (n = 12)

Simulation result:
barrier to water

# CFTR (PDB ID: 5W81)

*Danio rerio*
cryo–EM (3.37 Å)

Zhang et al., 2017

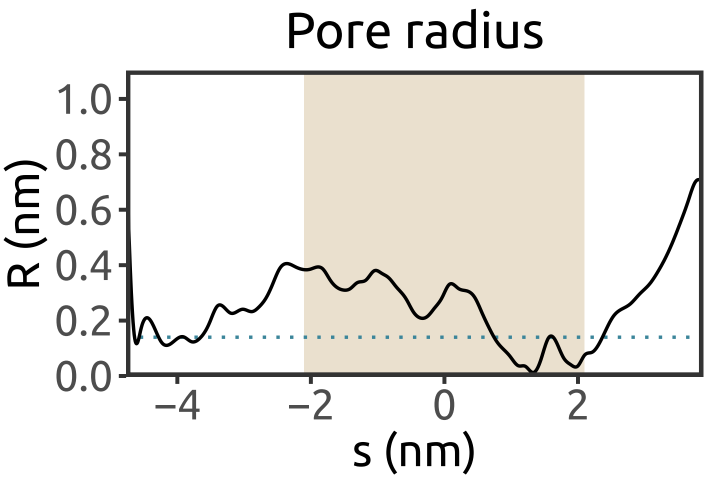


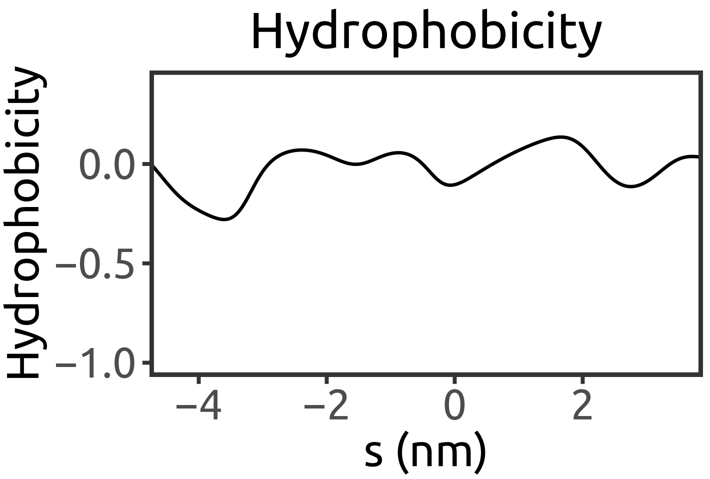


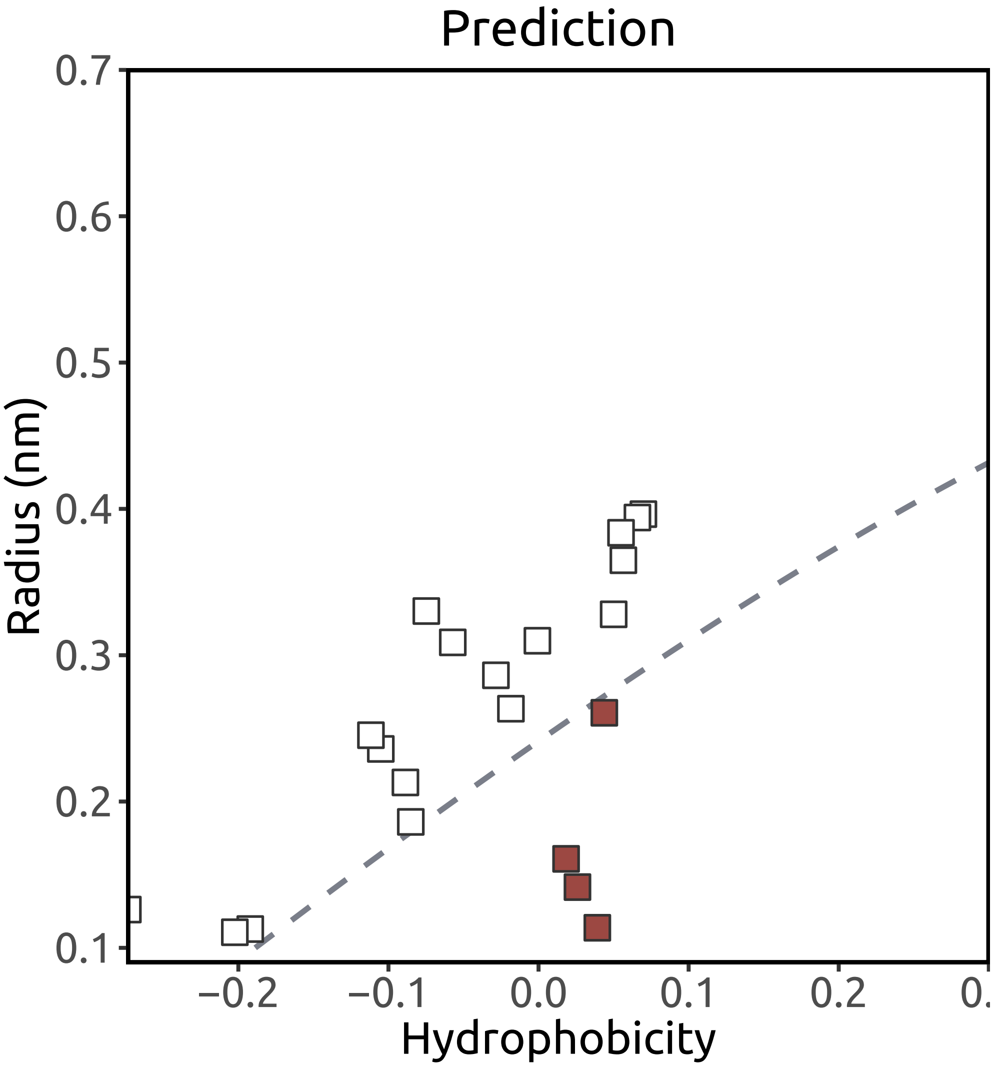


Heuristic score:
1.37 (n = 9)

Simulation result:
barrier to water

# CLC (PDB ID: 5TQQ)

*Bos taurus*

cryo−EM (3.76 Å)

Park et al., 2017

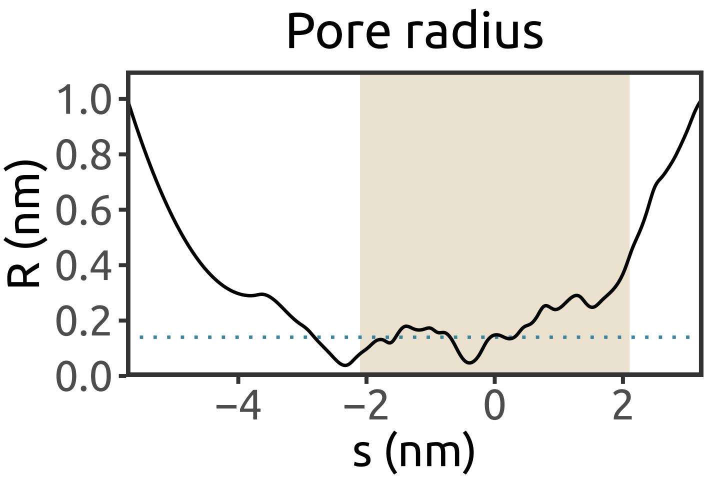


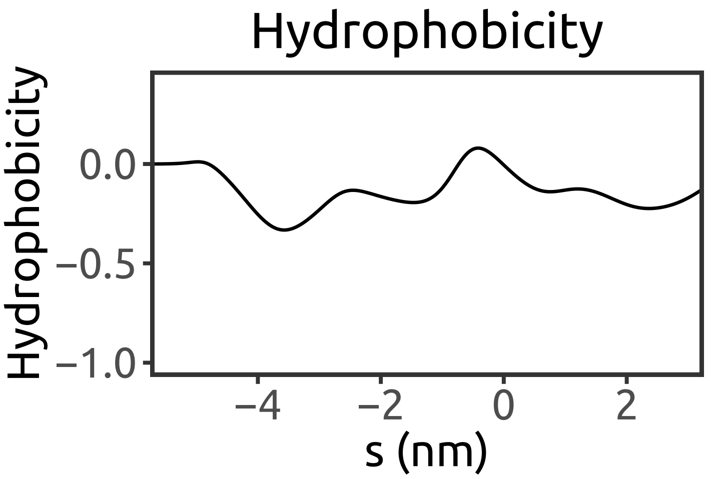


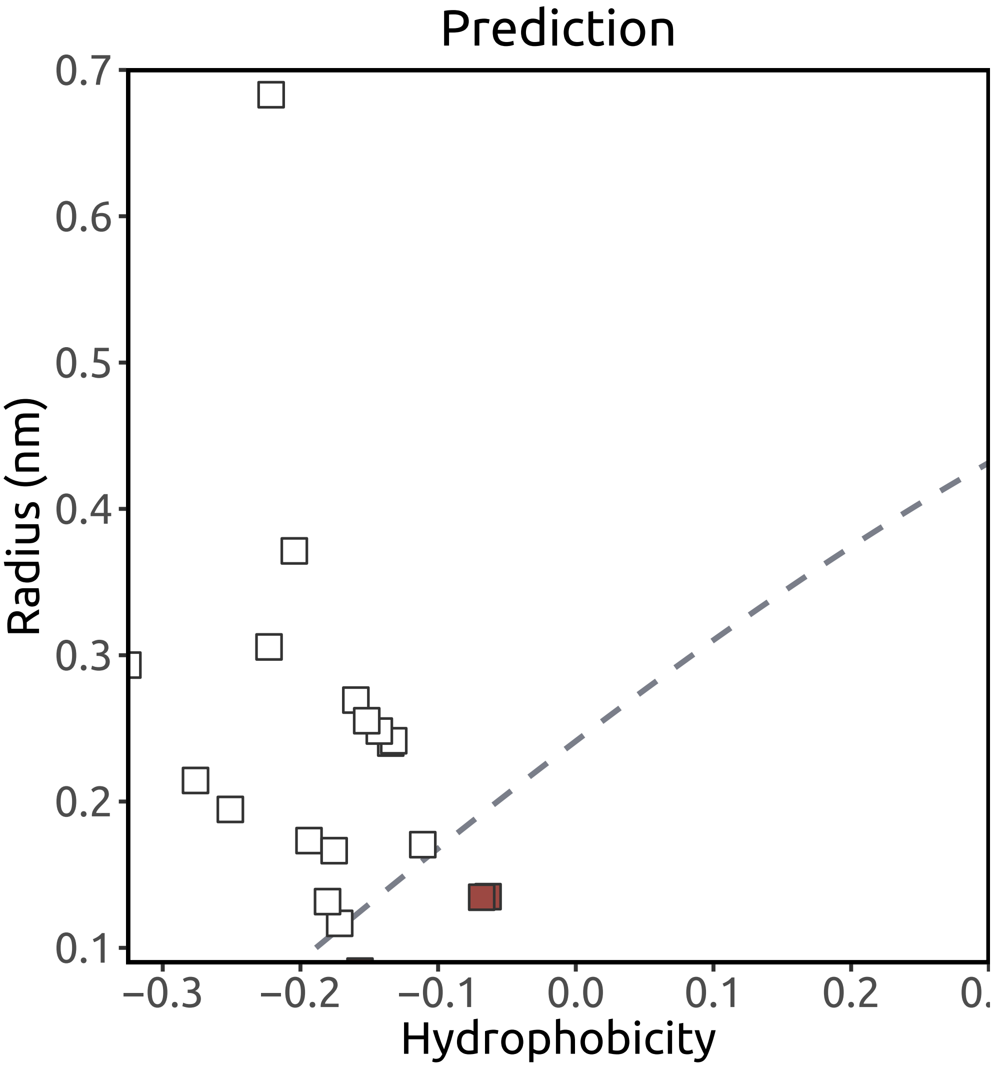


Heuristic score:

0.41 (n = 5)

Simulation result:

barrier to water

# KpBest (PDB ID: 4WD8)

*Klebsiella pneumoniae*

X−ray (2.3 Å)

Yang et al., 2014

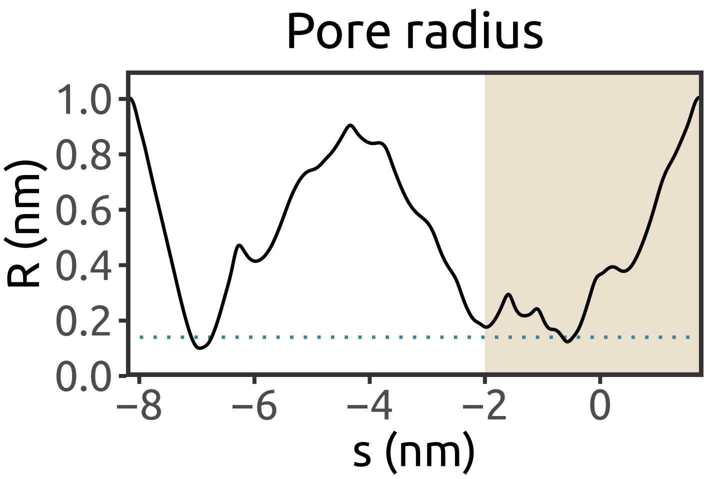


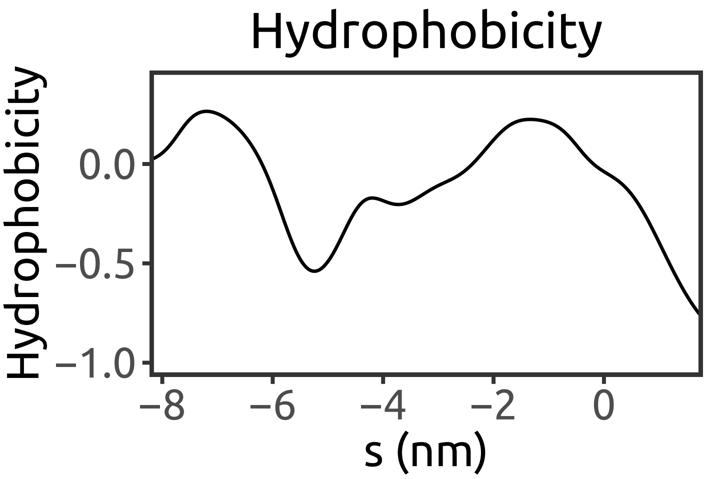


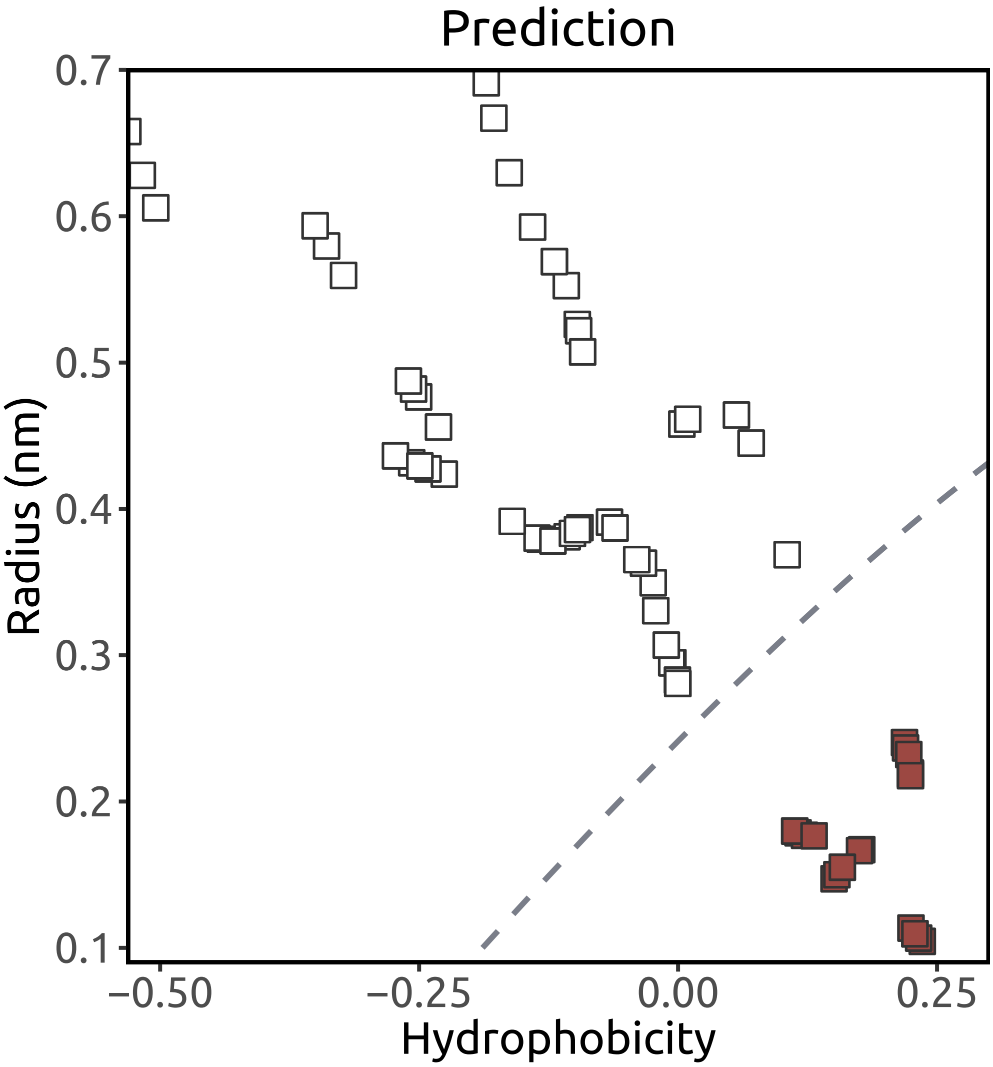


Heuristic score:

3.57 (n = 22)

Simulation result:

barrier to water

# TehA (PDB ID: 3M71)

*Haemophilus influenzae*
X–ray (1.2 Å)

Chen et al., 2010

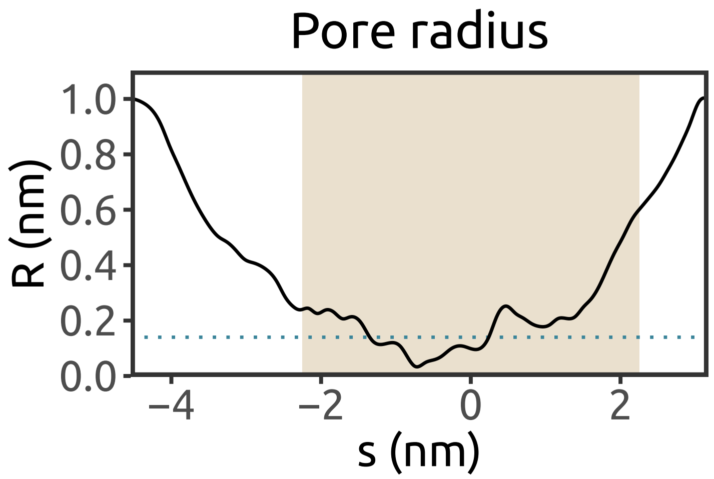


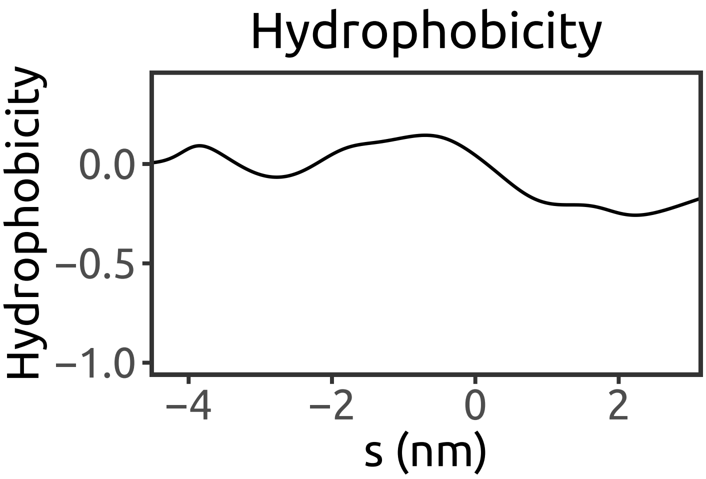


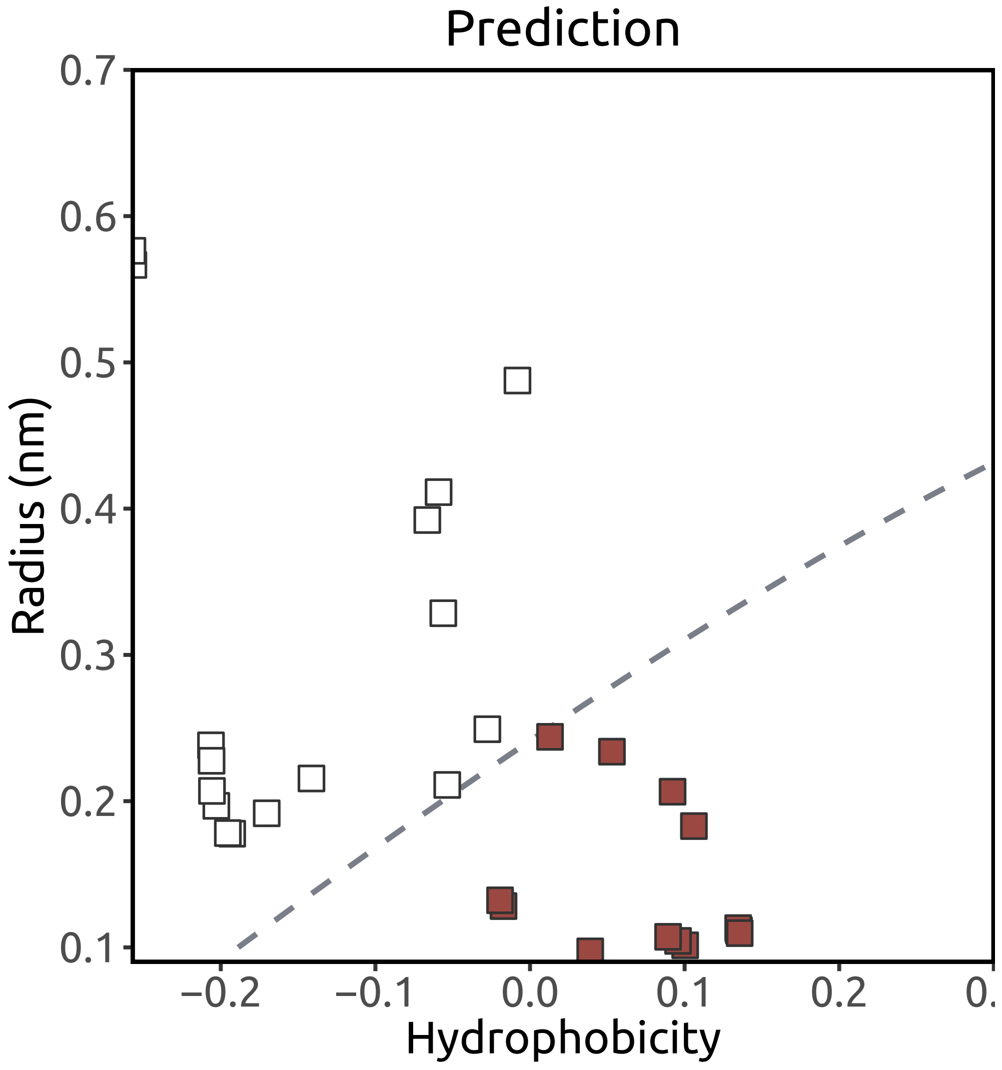


Heuristic score:
2.51 (n = 17)

Simulation result:
barrier to water

# TehA (PDB ID: 4YCR)

*Haemophilus influenzae*
X−ray (2.3 Å)

Axford et al., 2015

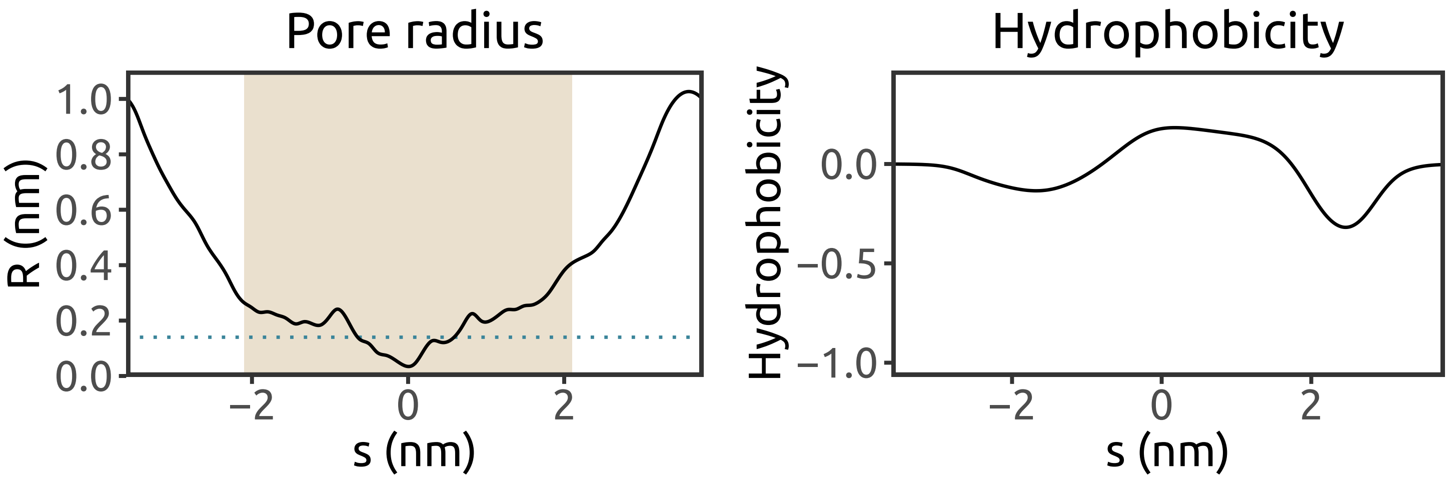


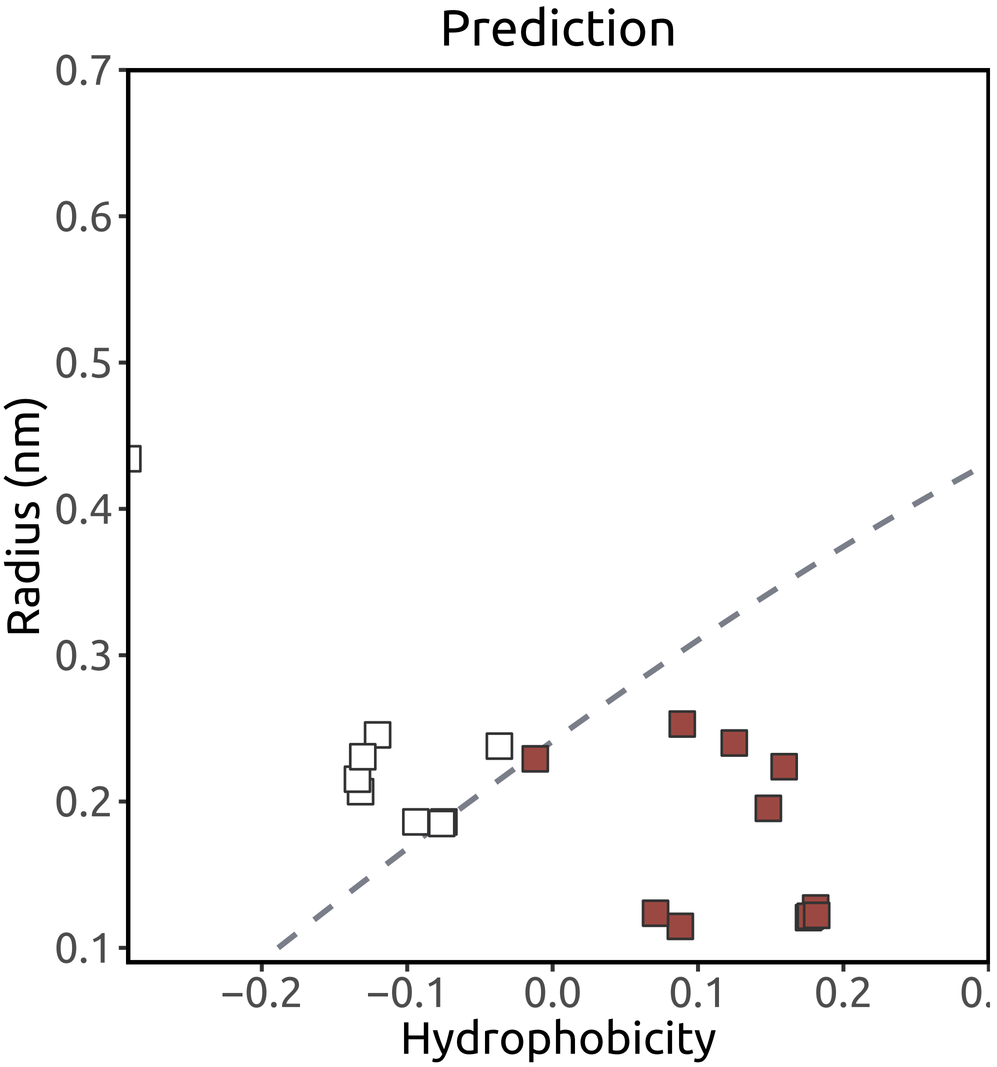


Heuristic score:

1.96 (n = 14)

Simulation result:

barrier to water

# TMEM16A (PDB ID: 5OYB)

*Mus musculus*
cryo−EM (3.75 Å)

Paulino et al., 2017

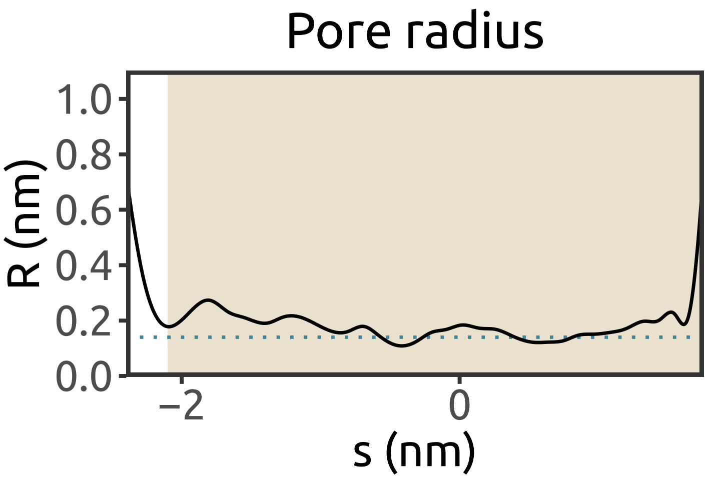


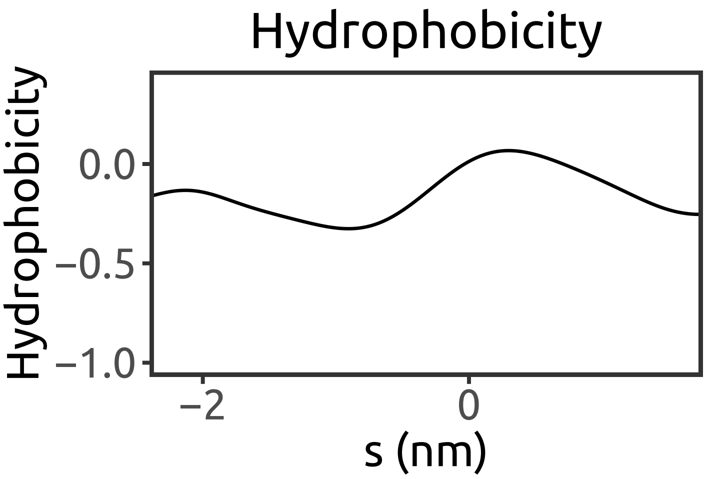


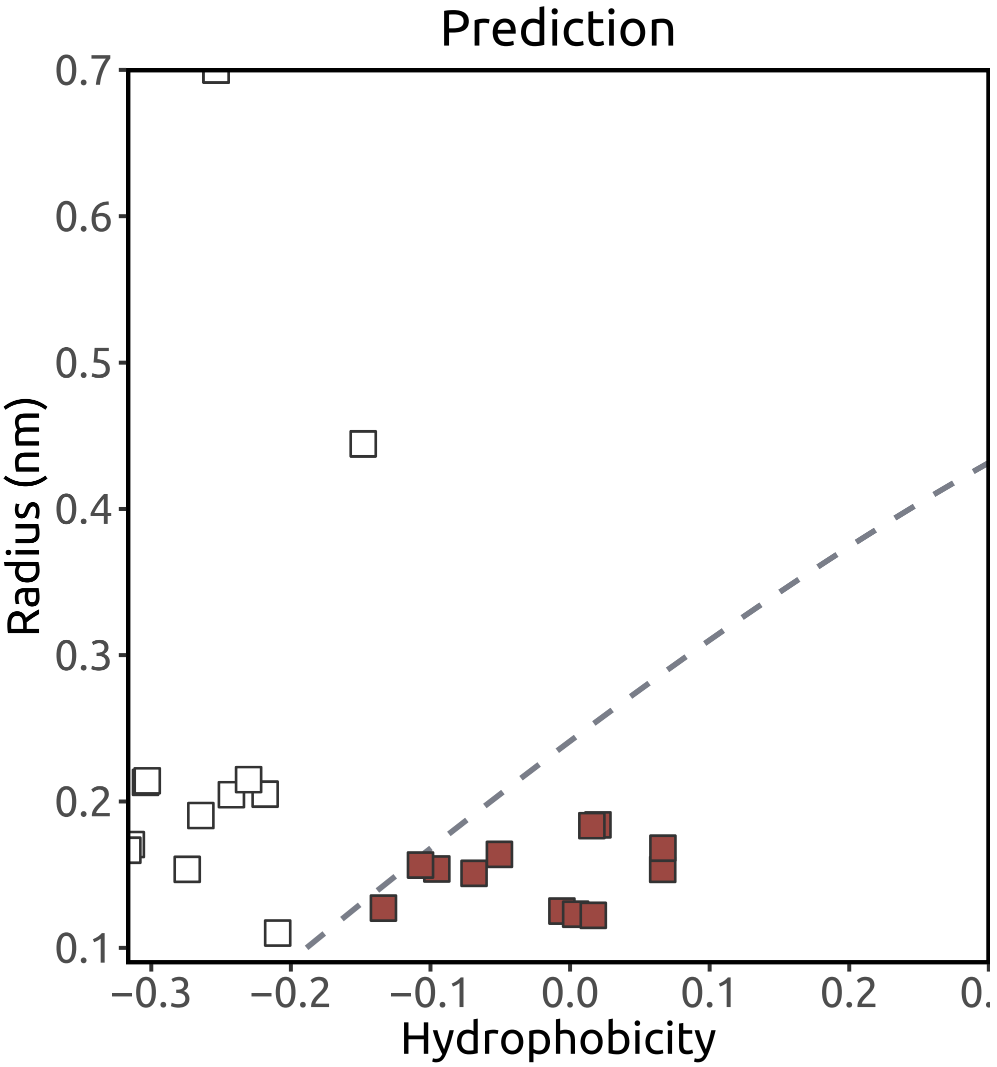


Heuristic score:
0.71 (n = 12)

Simulation result:
barrier to water

# TMEM16A (PDB ID: 5OYG)

*Mus musculus*
cryo−EM (4.06 Å)

Paulino et al., 2017

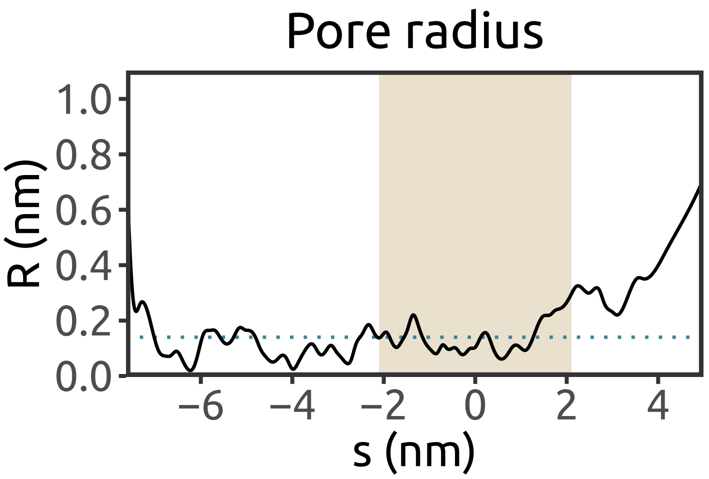


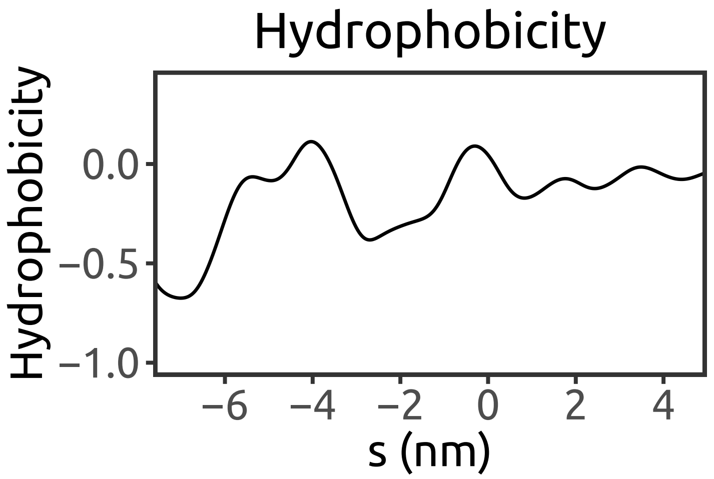


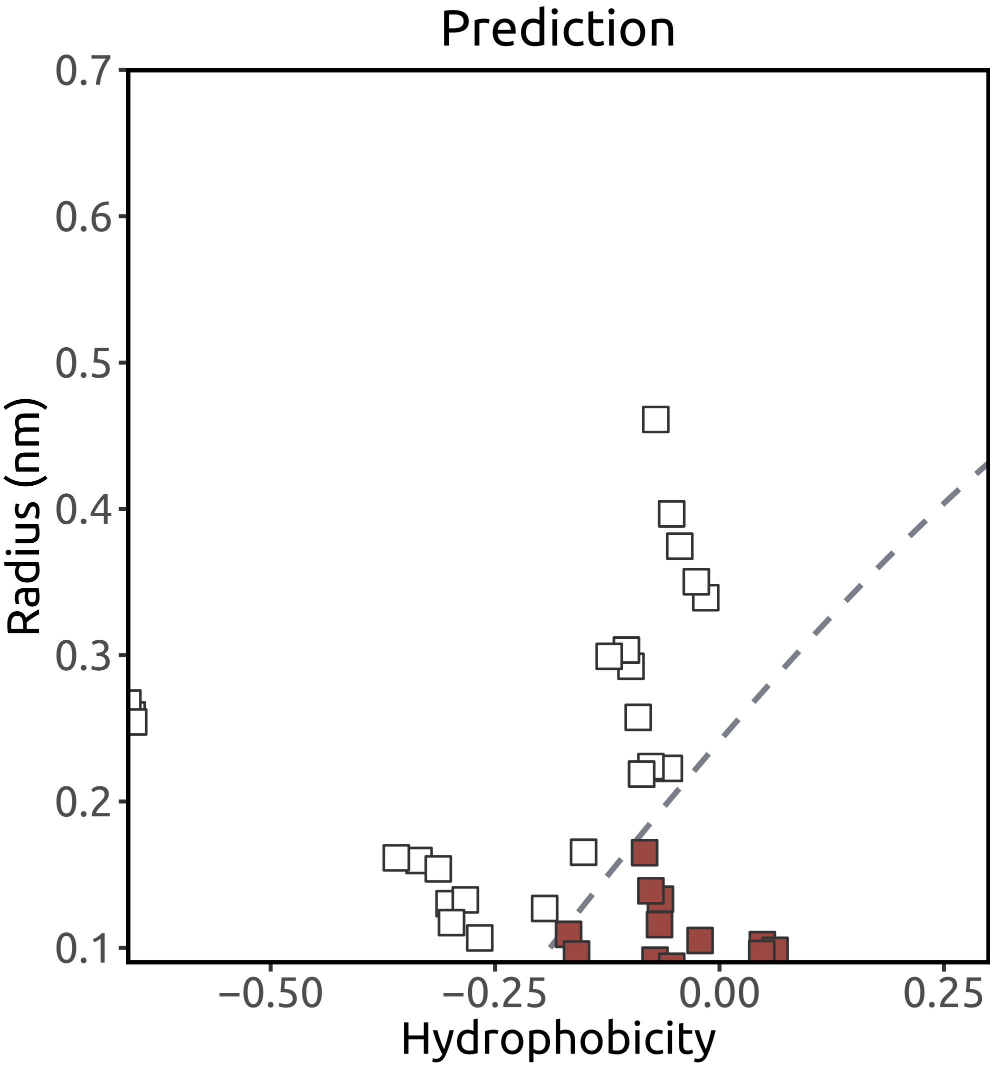


Heuristic score:
1.36 (n = 15)

Simulation result:
barrier to water

# TMEM16A (PDB ID: 6BGI)

*Mus musculus*
cryo–EM (3.8 Å)

Dang et al., 2017

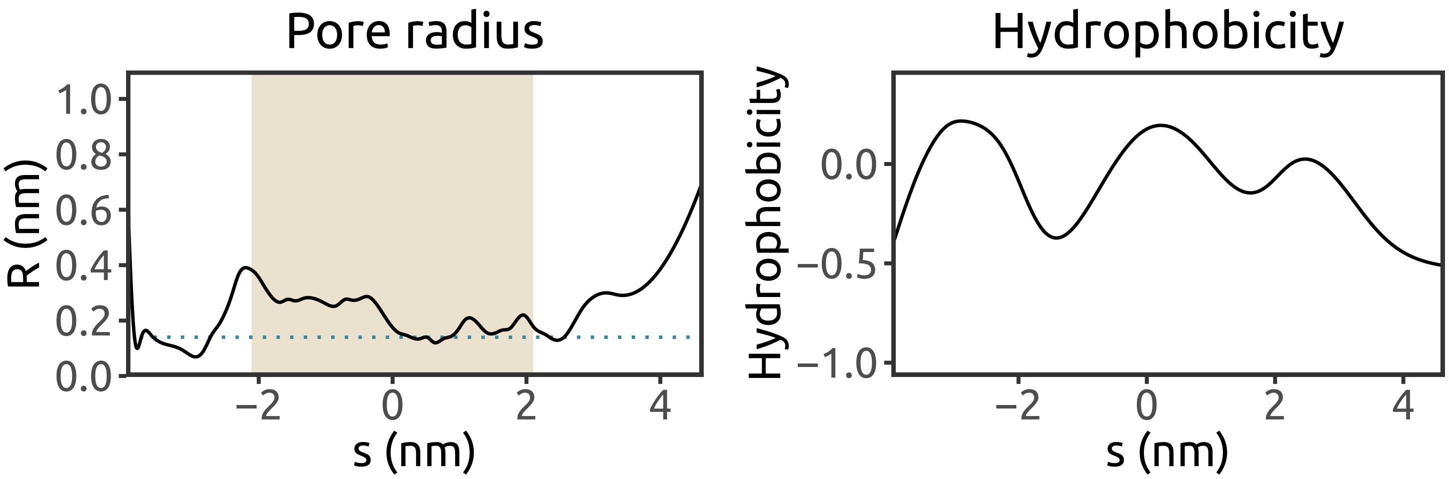


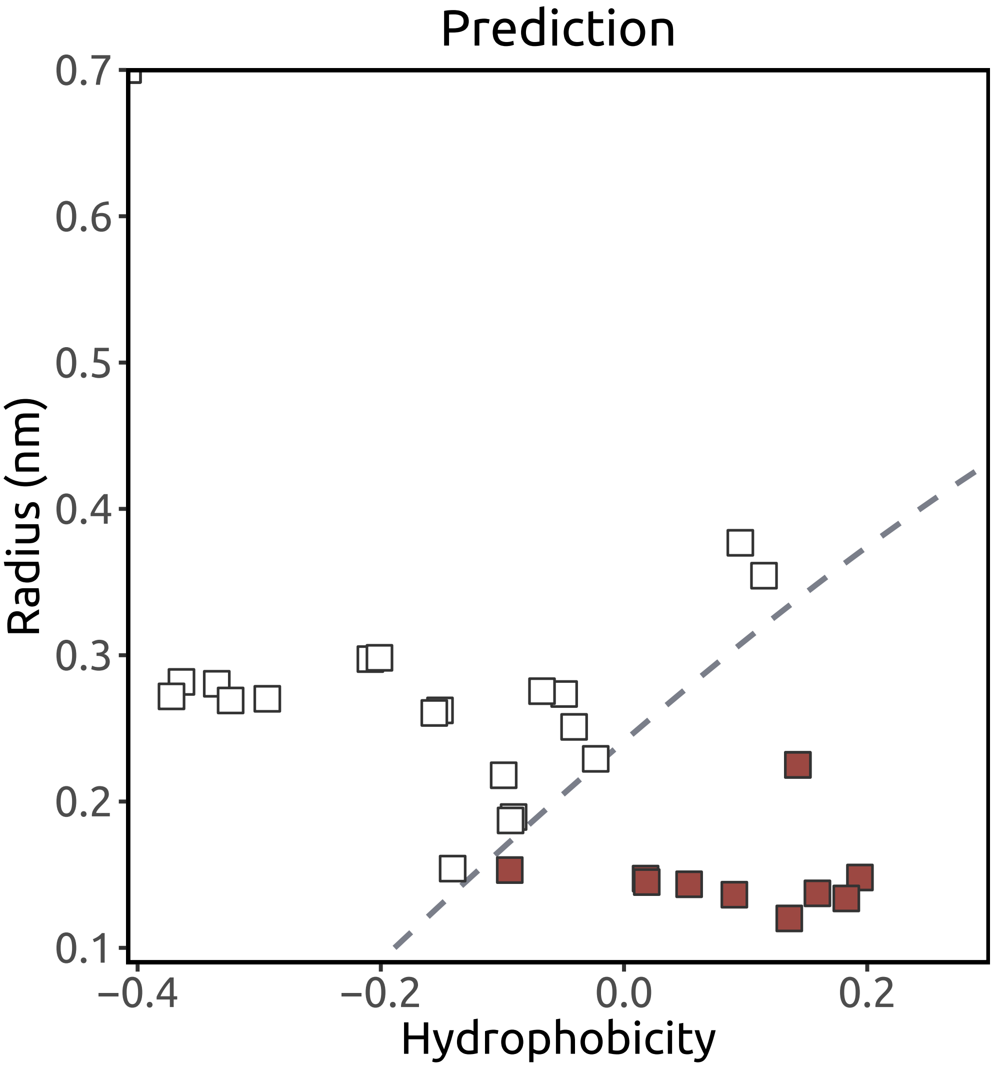


Heuristic score:
1.36 (n = 11)

Simulation result:
barrier to water

# TMEM16A (PDB ID: 6BGJ)

*Mus musculus*

cryo–EM (3.8 Å)

Dang et al., 2017

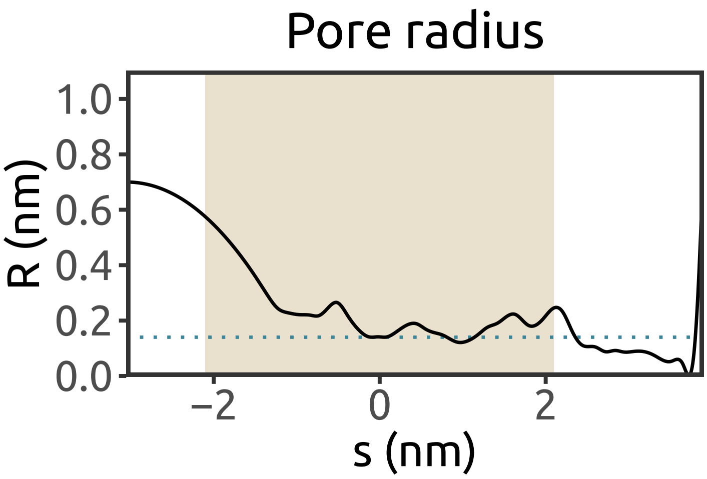


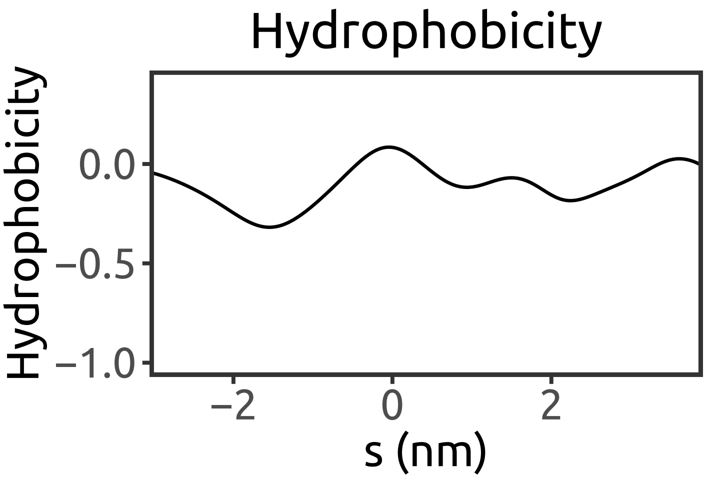


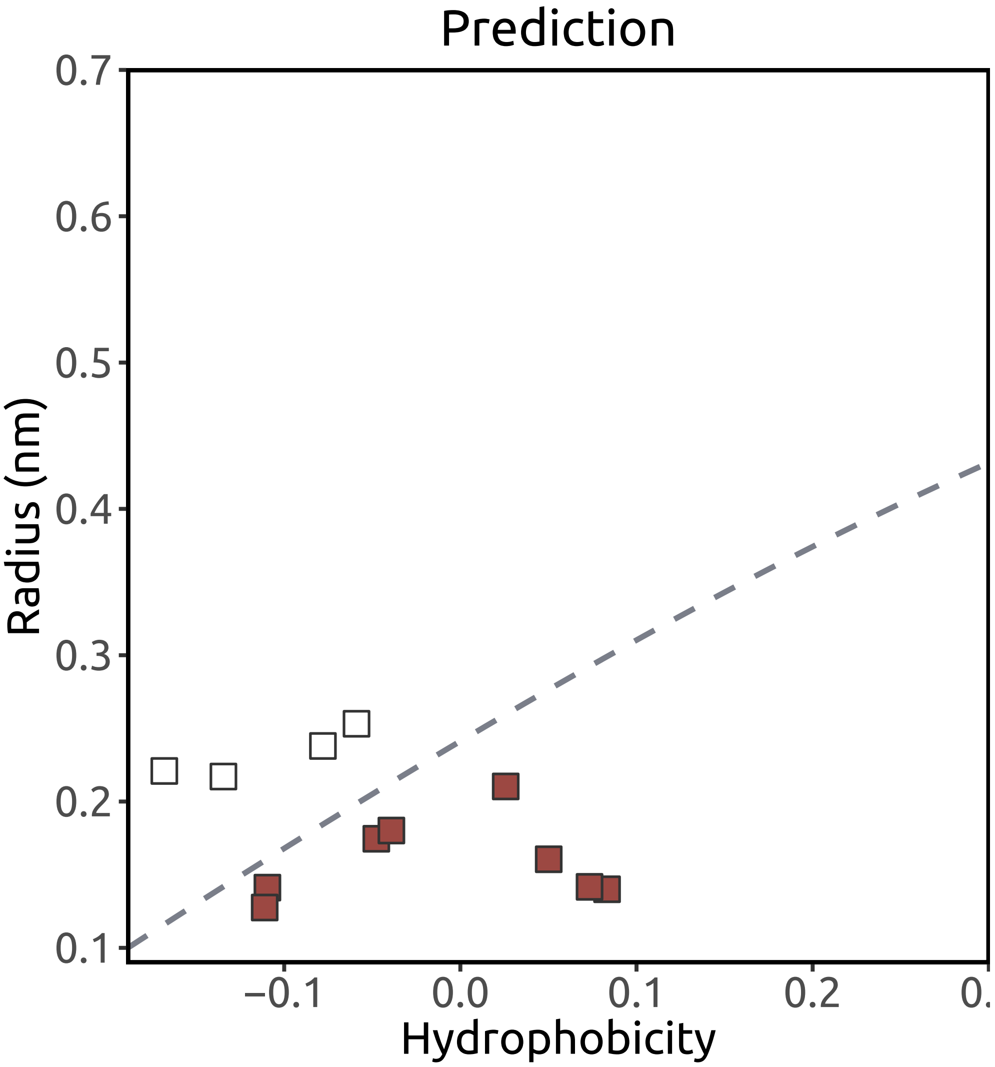


Heuristic score:

0.48 (n = 8)

Simulation result:

barrier to water
